# Bifunctional Iminophosphorane‐Catalyzed Enantioselective Nitroalkane Addition to Unactivated α,β‐Unsaturated Esters**

**DOI:** 10.1002/anie.202303391

**Published:** 2023-04-13

**Authors:** Daniel Rozsar, Alistair J. M. Farley, Iain McLauchlan, Benjamin D. A. Shennan, Ken Yamazaki, Darren J. Dixon

**Affiliations:** ^1^ Department of Chemistry University of Oxford Chemistry Research Laboratory OX1 3TA Oxford UK; ^2^ Division of Applied Chemistry Okayama University 700-8530 Tsushimanaka Okayama Japan

**Keywords:** Asymmetric Catalysis, C−C Bond Formation, Conjugate Addition, Enantioselective Synthesis, Organocatalysis

## Abstract

Herein we describe the enantioselective intermolecular conjugate addition of nitroalkanes to unactivated α,β‐unsaturated esters, catalyzed by a bifunctional iminophosphorane (BIMP) superbase. The transformation provides the most direct access to pharmaceutically relevant enantioenriched γ‐nitroesters, utilizing feedstock chemicals, with unprecedented selectivity. The methodology exhibits a broad substrate scope, including β‐(fluoro)alkyl, aryl and heteroaryl substituted electrophiles, and was successfully applied on a gram scale with reduced catalyst loading, and, additionally, catalyst recovery was carried out. The formal synthesis of a range of drug molecules, and an enantioselective synthesis of (*S*)‐rolipram were achieved. Additionally, computational studies revealed key reaction intermediates and transition state structures, and provided rationale for high enantioselectivities, in good agreement with experimental results.

## Graphical abstract

The first intermolecular enantioselective addition of nitroalkanes to unactivated α,β‐unsaturated esters is described, catalyzed by a bifunctional iminophosphorane (BIMP) superbase. This fundamental synthetically relevant transformation proceeds with high enantiomeric excesses and yields over a wide range of feedstock substrates, providing pharmaceutically relevant building blocks in a single step.

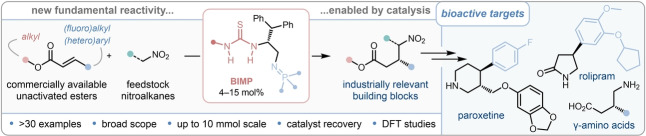

## Introduction

**Scheme 1:**
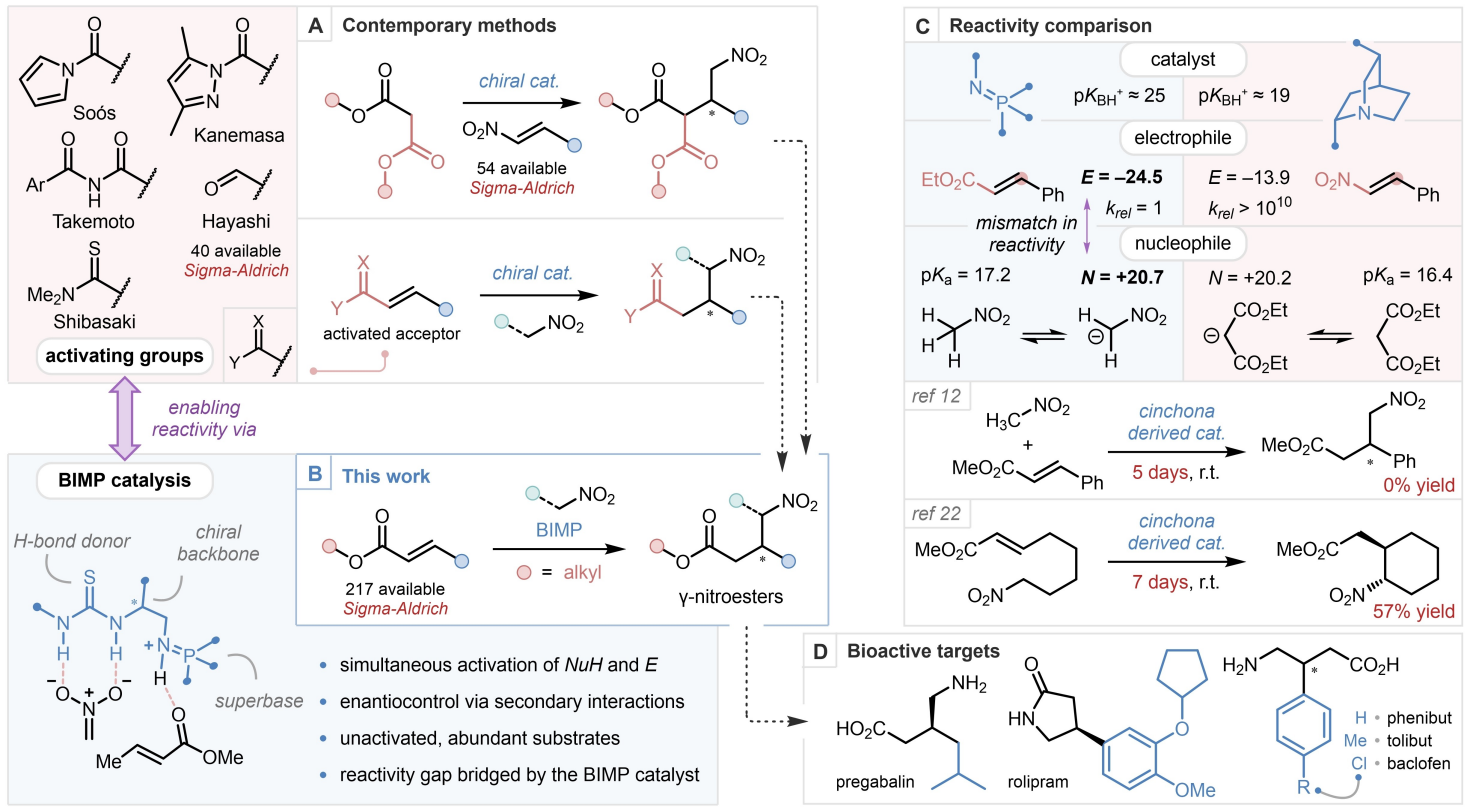
Contemporary methods for the synthesis of γ‐nitroesters (A). Streamlining the synthesis of γ‐nitroesters with BIMP catalysis (B). Reactivity comparison between the BIMP catalyzed nitromethane addition to α,β‐unsaturated esters, and the bifunctional cinchona‐derivative catalyzed malonate addition to nitroolefins based on p*K*_BH+_ values (in MeCN),^[23a]^ Mayr's reactivity parameters (*E* and *N* in DMSO)[^6^, ^21^] and p*K*_a_ values (in DMSO),[^23b^, ^23c^] and the inter‐ and intramolecular bifunctional cinchona derivative‐catalyzed enantioselective nitroalkane addition to α,β‐unsaturated ester (C). Directly available drug molecules from γ‐nitroesters (D).

Catalytic enantioselective carbon–carbon bond forming reactions are a cornerstone of contemporary organic synthesis.^[1]^ Amongst them, the venerable Michael addition reaction allows the direct formation of desirable stereogenic centres with perfect atom economy, in a single step, often using inexpensive and abundant starting materials. As such, the discovery of catalytic and enantioselective Michael additions, both metal‐catalyzed and metal‐free, have been at the forefront of organic methodology development for decades.^[2]^ The enantioselective addition of nitroalkanes, and specifically nitromethane, to electron poor olefins has received great attention, as the products obtained may be converted to γ‐nitroesters, which provide direct entry to a plethora of medicinally and industrially relevant compounds, specifically 2‐pyrrolidinones, 2‐piperidones, and γ‐amino acids.^[3]^ γ‐Aminobutyric acid (GABA), the simplest 4‐aminobutyric acid, is a primary regulator of the mammalian central nervous system.^[4]^ Synthetic analogues of GABA, substituted at the β‐position, such as pregabalin, baclofen and phenibut, exhibit significant bioactivity, including analgesic, tranquilizing, antiallodynic, anticonvulsant and anxiolytic effects (Scheme 1 D).^[5]^

The enantioselective Michael addition involving unactivated α,β‐unsaturated esters is a highly desirable transformation due to the abundance of these feedstock chemicals, however examples of such reactions remain scarce, due to the diminished reactivity of the conjugated alkene functionality as an electrophile.^[6]^ To our knowledge, the only known intermolecular organocatalytic enantioselective C−C bond forming reaction employing unactivated α,β‐unsaturated esters is a Diels–Alder reaction developed by List,^[7]^ whilst metal‐catalyzed examples are more abundant and include the enantioselective addition of organocuprates, pioneered by Feringa^[8]^ and Rh‐diene‐catalyzed addition of arylboronic acids developed by Hayashi and Miyaura.^[9]^ There are, in contrast, numerous reports of the enantioselective addition of nitromethane to *activated* α,β‐unsaturated carboxylic acid derivatives, including *N*‐acylpyrazoles by Kanemasa,^[10]^ imides by Takemoto,^[11]^ *N*‐acylpyrroles by Soós,^[12]^ and thioamides by Shibasaki.^[13]^ Jørgensen, building upon the seminal work of Hayashi,^[14]^ utilised α,β‐unsaturated aldehydes and diphenyl prolinol silyl ether catalysis to perform an enantioselective 1,4‐nitromethane addition, followed by oxidation using NBS to furnish γ‐nitroesters in a one‐pot fashion.^[15]^ An alternative strategy for the enantioselective synthesis of γ‐nitroesters can be envisaged by the conjugate addition of malonate esters to nitroolefins, followed by decarboxylation. Bifunctional tertiary amine bases can efficiently promote this addition, as demonstrated by Takemoto,^[16]^ Deng,^[17]^ Connon,^[18]^ and our own laboratory^[19]^ (Scheme 1 A). The above and related syntheses have been widely studied, and even performed in a flow setting in multiple accounts by Kobayashi and Kappe, and in a domino fashion by Corma, underpinning the importance of GABA analogues.^[20]^ While elegant and often efficient, the above methods necessitate the installation of bespoke activating groups which require the downstream manipulation of the obtained enantioenriched material to achieve the synthesis of γ‐nitroesters. Synthetic efficiency has thus been compromised to compensate for the low reactivity of existing catalysts. A more straightforward entry to enantioenriched γ‐nitroesters would entail the direct, intermolecular enantioselective addition of nitromethane to unactivated α,β‐unsaturated esters. However, due to the mismatch in reactivity between the nitronate nucleophile and unactivated α,β‐unsaturated esters (as indicated by Mayr's reactivity scale), this desirable disconnection is currently out of reach (Scheme 1 B, C).[^6^, ^21^]

Interestingly, the intramolecular enantioselective addition of certain tethered nitroalkanes to unactivated α,β‐unsaturated esters has been reported, using bifunctional cinchona‐derived catalysts.^[22]^ However, even in these kinetically favoured 6‐membered ring‐forming systems, applicability is plagued by long reaction times (≈7 days) necessary to achieve moderate to high yields (Scheme 1 C). In 2013, our group introduced a new family of bifunctional superbases,[^24^, ^25^] the bifunctional iminophosphorane (BIMP) catalysts,[^26^, ^27^] which have been demonstrably successful in promoting highly challenging enantioselective conjugate additions.^[28]^ Seeking to overcome the limitations present in contemporary methods, and to unlock the synthetic potential of two abundant, yet underutilized classes of feedstock chemicals, we aimed to develop the first enantioselective intermolecular addition of nitroalkanes to unactivated α,β‐unsaturated esters. If successful, our methodology would provide the most straightforward, single‐step, entry to valuable enantioenriched γ‐nitroesters. To achieve this, we hoped to identify a BIMP catalyst capable of the simultaneous activation of the electrophile and the pronucleophile and furnish the desired products with high levels of enantiocontrol (Scheme 1 B).

## Results and Discussion

### Reaction Optimization

To establish BIMP catalyst‐enabled reactivity in the enantioselective addition of nitromethane to α,β‐unsaturated esters, we selected commercially available (*E*)‐methyl‐crotonate **1 a**, and nitromethane **2 a** as the model system. Reactions were run at 0.1 mmol scale with 10 mol % catalyst loading, using 10 equivalents of **2 a**, and were quenched after 24 or 96 hours by passing the reaction mixture through a short silica plug. An initial catalyst screen revealed that first generation BIMP catalysts bearing a thiourea hydrogen bond donor (HBD) and a single stereocenter were significantly superior to others in our library.^[29]^ Catalyst **B1** provided the desired γ‐nitroester **3 a** in 69 % yield and 80 : 20 enantiomeric ratio (e.r.), albeit over 4 days of reaction time, even under neat conditions (Table 1, entry 1). With the HBD moiety established, the substituent effects of the chiral backbone were investigated, and found to be substantial. L‐Serine derived catalyst **B2**, bearing a benzhydryl sidechain, provided **3 a** in 84.5 : 15.5 e.r., while other substituents in the same position proved to be detrimental to selectivity (entry 2).^[29]^ In a bid to increase selectivity and reactivity, the late stage tunability of the BIMP catalysts was exploited, and a thorough investigation of the iminophosphorane substituent was performed by varying the trivalent phosphine deployed in the Staudinger reaction. It was found that replacing the *para*‐methoxyphenyl (PMP) substituents with peripherally bulky and electron‐rich 3,5‐*di*‐*tert*‐butylphenyl substituents gave a slight increase in selectivity (**B3**, 86.5 : 13.5 e.r.), however conversion remained at only 47 % over a 24‐period (entry 3). Finally, the fine tuning of the HBD revealed that a tetra‐trifluoromethylated terphenyl substituent in **B4** gave a notable uplift in selectivity (90.5 : 9.5 e.r.), and crucially in reactivity, providing **3 a** in 73 % yield in only 24 hours (entry 4). Further structural modifications to catalyst **B4** led to no improvements in the outcome of the reaction. At this stage, a thorough solvent screen was performed at high concentrations (3.3 M, 30 μL solvent), to circumvent potential decrease in reactivity due to over‐dilution. It was found that most solvents provided only a slight increase in selectivity, and low variation in reactivity (entries 5, 6).^[29]^ Switching the reaction medium to cyclohexane, however, afforded a surprising and sharp increase in both reactivity and selectivity, providing **3 a** in 88 % yield and 95 : 5 e.r. in 24 hours (entry 7). Notably, this reaction mixture was biphasic due to the immiscibility of cyclohexane and nitromethane, however crucially all components were soluble in one of the two phases, including catalyst **B4**. Finally, to increase the isolated yield of product **3 a**, the catalyst loading was adjusted to 15 mol %, providing **3 a** in 99 % isolated yield and 95 : 5 e.r. on a 0.3 mmol scale (entry 8).^[30]^ Importantly, the transformation could be conducted under air with no detrimental effects to yield or enantioselectivity, as a testament to the robustness of our catalytic system.

**Table 1:**
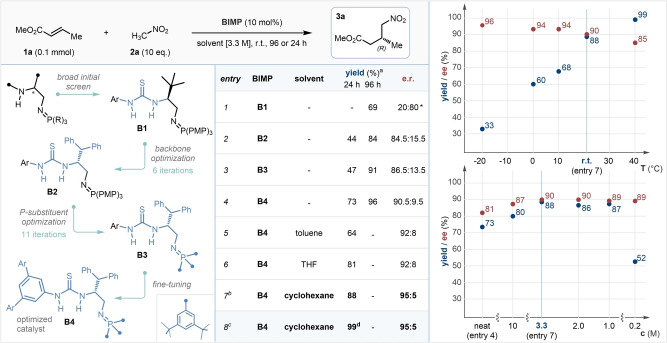
Catalyst development and solvent screen (left), condition optimization (right; unchanged conditions are identical to those in entry 7). 0.1 mmol scale.

[a] Determined by ^1^H NMR by comparing starting material and product peaks. [b] Average result of two experiments. [c] Optimized reaction conditions: 15 mol % **B5**, 3.0 M, 3.0 equiv MeNO_2_, 0.3 mmol scale, under air. [d] Isolated yield. * The opposite enantiomer, (*S*)‐**3 a**, was obtained. e.r. determined by HPLC on a chiral stationary phase. PMP: *para*‐methoxyphenyl. Ar: 3,5‐bis‐(CF)_3_‐phenyl.

### Scope and Limitations

**Scheme 2:**
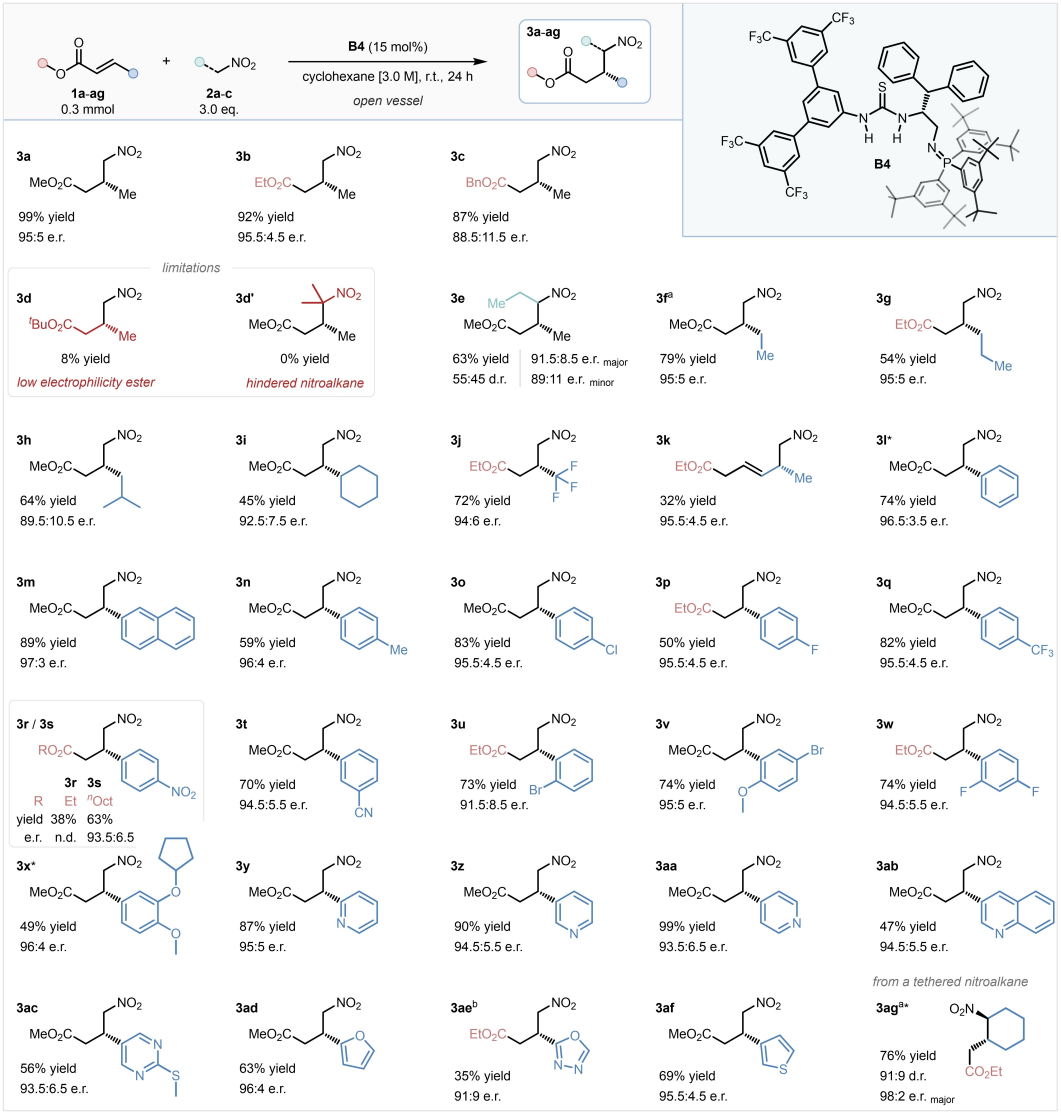
Substrate scope. * Absolute stereochemical configuration was determined by chemical correlation. Isolated yields. e.r. determined by HPLC on a chiral stationary phase.^[a]^ e.r. determined by supercritical fluid chromatography (SFC) on a chiral stationary phase.^[b]^ 48 h reaction time.

After establishing the optimal conditions for the model reaction, the generality of the methodology was explored. Reactions were conducted on a synthetically useful, 0.3 mmol scale (Scheme 2). First, the effects of the alkoxy‐substituents of the ester were investigated, using substrates **1 a**–**d**. Switching the model substrate to ethyl substituted analogue **1 b** had no considerable effect on the outcome of the transformation, which importantly allows use of commercially widely available ethyl esters. A bulkier benzyl group reduced the isolated yield to 87 % and the e.r. to 88.5 : 11.5, while *tert*‐butyl crotonate **1 d** was incompatible with the system, providing product **3 d** in only 8 % yield, likely due to the substrate's decreased electrophilicity.^[6]^ When 2‐nitropropane was employed as a pro‐nucleophile, no product formation was observed, most probably owing to the pronucleophile's increased steric hinderance (**3 d′**). 1‐Nitropropane, however, was a suitable substrate, and product **3 e** was obtained as a 55 : 45 mixture of diastereomers (with 91.5 : 8.5 e.r. and 89 : 11 e.r., respectively) in 63 % yield. Next, various alkyl substituents in the β‐position were investigated (**3 f**–**j**). Product **3 f** was obtained in excellent 79 % yield and 95 : 5 e.r., and product **3 g** in 54 % yield and 95 : 5 e.r. Pregabalin precursor **3 h** was synthesized from commercially available substrates in 64 % yield and slightly diminished 89.5 : 10.5 e.r., probably due to increased steric hinderance, while cyclohexyl substituted ester **1 i** underwent the transformation smoothly, furnishing **3 i** in 45 % yield and 92.5 : 7.5 e.r. Product **3 j**, bearing a medicinally relevant trifluoromethylated stereocenter, was obtained in an excellent 72 % yield and 94 : 6 e.r. from commercially available ester **1 j**.^[31]^ Doubly‐unsaturated ethyl sorbate **1 h** underwent the transformation in a moderate yield and excellent enantioselectivity, and provided unconjugated product **3 k** after a selective 1,6 addition as a single regioisomer. Next, a series of substituted cinnamate esters were evaluated (**1 l**–**x**). Pleasingly, an impressive range of aryl substituents were tolerated with very little variation in selectivity. Electronically neutral methyl cinnamate and 2‐naphthyl acrylate (**1 l**, **1 m**) provided **3 l** (the precursor of phenibut; previously unavailable under bifunctional cinchona‐derived catalysis),^[12]^ and **3 m** in excellent yield and selectivity. Next, *para*‐substituted aromatic substrates were investigated (**1 n**–**s**), and to our delight both electron donating and electron withdrawing substituents were tolerated exceptionally well with consistently high selectivity, however electronically rich substrates provided the corresponding products in slightly diminished yields due to lower electrophilicity. Crucially, the precursors of tolibut (**3 n**), baclofen (**3 o**) and paroxetine (**3 p**) were obtained in good to excellent yields, and high selectivity from simple, and in the case of baclofen, inexpensive, and commercially available, starting materials. 4‐Nitroethyl cinnamate (**1 r**) gave a surprisingly low, 38 % isolated yield, which we speculated was due to its low solubility in cyclohexane. Exploiting the catalytic system's ability to tolerate *n*‐alkyl chains on the ester's *O* substituent, a lipophilic *n*‐octyl analogue of **1 r** was prepared, which was fully soluble in the reaction mixture, and underwent the transformation smoothly, furnishing **3 s** in 63 % yield and 93.5 : 6.5 e.r. Substrate **1 t** bearing a nitrile group in the *meta* position yielded **3 t** in 70 % yield and 94.5 : 5.5 e.r., and even *ortho*‐bromo substituted product **3 u** was obtained in 73 % yield and 91.5 : 8.5 e.r. Disubstituted cinnamate analogues, **3 v**–**x**, underwent the transformation in good to excellent yields, and with no change in selectivity, and importantly **3 x**, a direct precursor of (*S*)‐rolipram, was obtained in 49 % yield and 96 : 4 e.r. Finally, a set of heteroaromatic α,β‐unsaturated esters was investigated. All three isomers of pharmaceutically relevant products bearing a pyridine substituted stereocenter **3 y**–**aa** were obtained in excellent enantioselectivities and yields, without the Lewis basic nitrogen compromising the catalytic process. Quinoline **1 ab** was also tolerated, however product **3 ab** was obtained in a slightly diminished yield, likely due the substrate's poor solubility in cyclohexane. Even pyrimidine **1 ac**, bearing an electron donating 4‐thioether moiety, was smoothly converted to the corresponding γ‐nitroester, providing **3 ac** in 56 % yield and 93.5 : 6.5 e.r. 5‐Membered heteroaromatics **1 ad**–**af** were similarly well tolerated, albeit oxadiazole **3 ae** was obtained in a slightly decreased 91 : 9 e.r., and moderate 35 % yield. Finally, tethered substrate **1 ag** was prepared and investigated under the optimized conditions, and the corresponding 1,2‐disubstituted cyclohexane **3 ag** was furnished in 76 % yield as a 91 : 9 mixture of diastereomers with nearly perfect enantioselectivity (98 : 2 e.r. of the major diastereomer), demonstrating a considerable improvement in reactivity compared with the earlier reported synthesis of the same product (87 % yield over 7 days under bifunctional cinchona‐derived catalysis).^[22]^

### Scale up and Derivatization

**Scheme 3:**
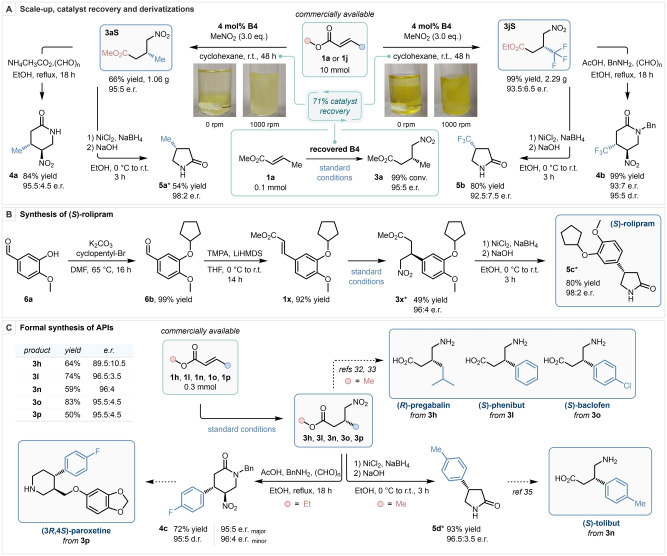
Preparative scale synthesis and catalyst recovery (A, photos of the reaction involving **1 a** were taken 5 minutes after set up, photos of the reaction involving **1 j** were taken 24 hours after set up). Synthesis of (*S*)‐rolipram (B). Formal synthesis of APIs (C). TMPA: Trimethyl phosphonoacetate. * Absolute stereochemical configuration was determined by chemical correlation.

After demonstrating the scope and limitations of our methodology, its synthetic applicability was established by scaling up both the model reaction and that employing trifluoromethylated substrate **1 j** to a 10 mmol scale (Scheme 3). Both reactions were performed under identical reaction conditions. For these transformations, the catalyst loading was reduced to 4 mol % (from 15 mol % previously), and reaction times were increased to 48 h. Product **3 aS** was isolated in 66 % yield and 95 : 5 e.r., and product **3 jS** was obtained in quantitative yield and 93.5 : 6.5 e.r. After the isolation of both products by flash column chromatography, the eluent polarity was increased to elute catalyst **B4** in both cases. The obtained catalyst was dissolved in *n*‐hexane and purified by means of an aqueous wash with 3 M NaOH. The recovered catalyst was then resubjected to the model reaction under the optimized reaction conditions, delivering the desired product in quantitative yield and with the same level of enantiopurity as previously established, demonstrating the robustness of the BIMP catalytic system. Subsequently, product **3 aS** was reacted with ammonium acetate and paraformaldehyde to furnish *trans*‐2‐ piperidinone **4 a** in 84 % yield, 95.5 : 4.5 e.r. with perfect diastereoselectivity, and the same product was reductively cyclized using in situ generated nickel boride to obtain 2‐pyrrolidinone **5 a** in a 54 % yield and 98 : 2 e.r. Trifluoromethylated γ‐nitroester **3 jS** was subjected to similar reaction conditions in the presence of benzylamine, and *N*‐benzyl protected *trans*‐2‐piperidinone **4 b** was obtained in quantitative yield, 93 : 7 e.r. and 95 : 5 d.r., while trifluoromethylated pyrrolidinone **5 b** was synthesized in 80 % yield and 92.5 : 7.5 e.r., yielding high‐value enantioenriched trifluoromethylated heterocycles in only two steps from commercially available starting materials.^[31]^ Next, the enantioselective synthesis of (*S*)‐rolipram was achieved by converting aldehyde **6 a** to α,β‐unsaturated ester **1 x** by alkylation of the phenolic OH, and subsequent HWE reaction in 92 % yield over two steps. The obtained product was then submitted to our methodology to furnish γ‐nitroester **3 x** in 49 % yield and 96 : 4 e.r., which was reductively converted to (*S*)‐rolipram (**5 c**) in a single step in 80 % yield and 98 : 2 e.r. Finally, a series of active pharmaceutical ingredients (API) were formally synthesized. With the synthesis of **3 h**, **3 l** and **3 o**, the enantioselective formal synthesis of (*R*)‐pregabalin,^[32]^ (*S*)‐phenibut and (*S*)‐baclofen^[33]^ was achieved. Additionally, **3 p** was converted to **4 c** in 72 % yield, 95 : 5 e.r. (major enantiomer) and 95 : 5 d.r. in a single step, en route to (3*R*,4*S*)‐paroxetine,[^15^, ^34^] and finally **3 n** was reductively cyclized to furnish 2‐pyrrolidinone **5 d** in 93 % yield and 96.5 : 3.5 e.r., achieving the formal synthesis of (*S*)‐tolibut.^[35]^

### Computational Study

**Figure 1:**
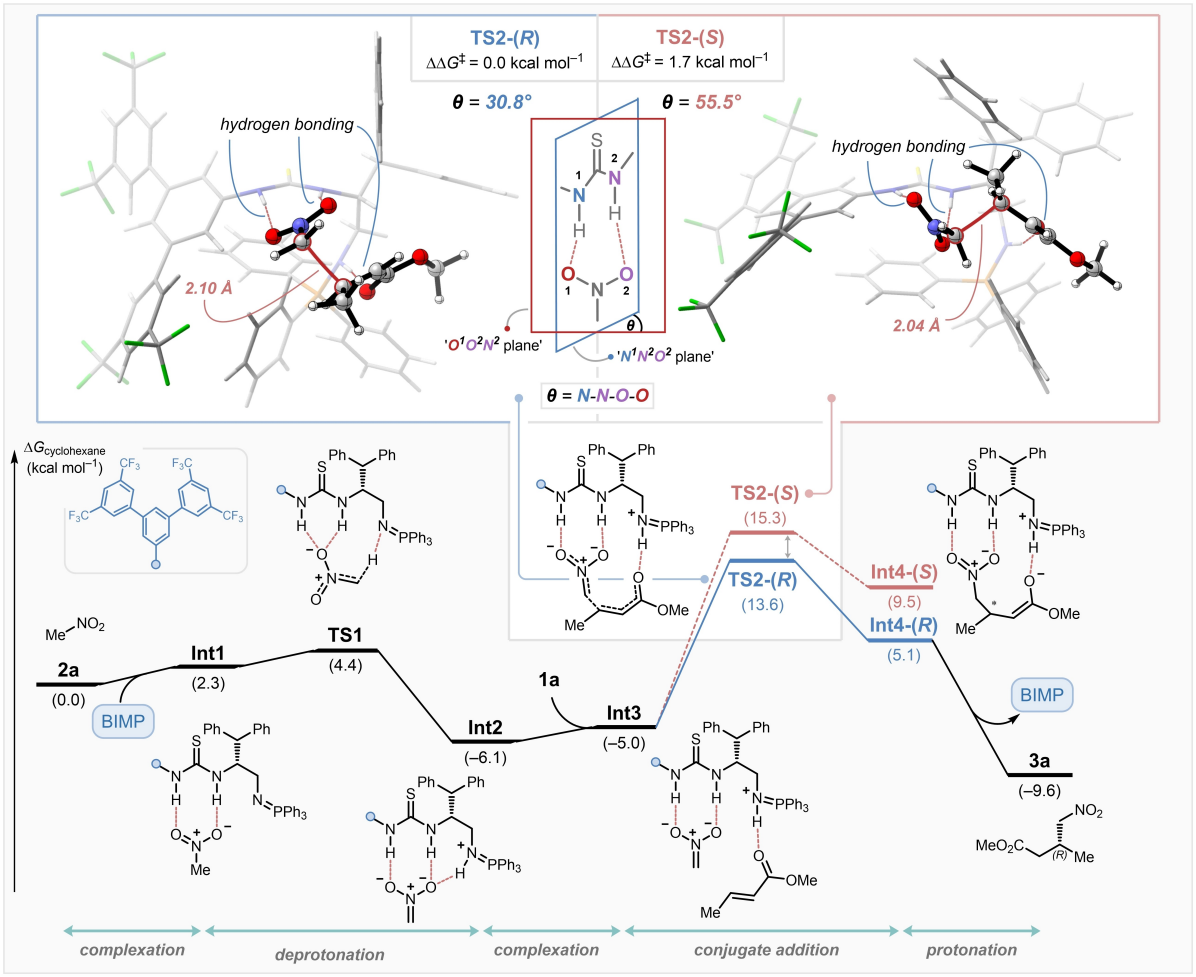
Nucleophilic attack transition state structures en route to (*R*)‐**3 a** and (*S*)‐**3 a** (top). Potential energy surface (Δ*G* [kcal mol^−1^])) for the enantioselective conjugate addition of nitroalkanes to unactivated α,β‐unsaturated esters at SMD(cyclohexane)/M062X/6‐311+G(d,p)//SMD(cyclohexane)/B3LYP‐D3/6‐31G(d) level of theory. Energies (kcal mol^−1^) and bond lengths [Å] of the transition state structures are provided in the insert (bottom).

To elucidate the mechanism and establish the origins of enantioselectivity, a DFT study was performed on the full catalytic cycle (Figure 1, bottom). Initially, complex **Int1** is formed by the hydrogen bonding interaction between the thiourea moiety of the BIMP catalyst and nitromethane (**2 a**). The following intramolecular deprotonation occurs rapidly through transition state structure (TS) **TS1** to generate a thermodynamically stable nitronate–protonated iminophosphorane ion pair, **Int2**. Prior to the enantio‐determining conjugate addition step, α,β‐unsaturated ester **1 a** coordinates to **Int2** and generates intermediate **Int3**. A conformational search of the conjugate addition transition state structures was then conducted based on the activation mode and the side‐chain conformation,^[29]^ and the lowest‐energy TS was found to be **TS2‐(*R*)** that forms the (*R*) enantiomer of product **3 a**, which is consistent with the experimentally confirmed absolute stereochemical outcome of the reaction. The energy difference with the second‐lowest transition state structure **TS2‐(*S*)** for the formation of the (*S*)‐product is 1.7 kcal mol^−1^, strongly supported by the experimentally observed enantioselectivity. Finally, the protonation of the enolate intermediate **Int4‐(*R*)** furnishes desired product **3 a** and regenerates the BIMP catalyst. The origin of the enantioselectivity arises from the TS geometry that benefits from the tight‐binding interaction between the thiourea moiety and the nitro group (Figure 1, top left). The nitro group in the disfavored transition state structure **TS2‐(*S*)** must rotate from the plane of the thiourea HBD to approach the ester substrate in which the free rotation is limited due to the steric repulsion. This is evidenced by the larger dihedral angle (*θ*=N−N−O−O) in **TS2‐(*S*)** compared to **TS2‐(*R*)**. Therefore, the conjugate addition of nitroalkanes to unactivated α,β‐unsaturated esters proceeds with high enantioselectivity.

## Conclusion

In summary, we have developed the first intermolecular enantioselective conjugate addition of nitroalkanes to unactivated α,β‐unsaturated esters to yield high‐value enantioenriched γ‐nitroesters from commercially widely available starting materials, streamlining their synthesis thanks to the power of bifunctional iminophosphorane catalysis. The methodology tolerates a larger range of substituents compared to previously reported strategies and can be applied to up to 10 mmol scale, with catalyst recovery. Computational studies shed light on the relevant transition state structures and provided an explanation for the high stereoselectivity of the developed transformation in agreement with experimental findings.

## Conflict of interest

The authors declare no conflict of interest.

1

## Supporting information

As a service to our authors and readers, this journal provides supporting information supplied by the authors. Such materials are peer reviewed and may be re‐organized for online delivery, but are not copy‐edited or typeset. Technical support issues arising from supporting information (other than missing files) should be addressed to the authors.

Supporting Information

## Data Availability

The data that support the findings of this study are available in the Supporting Information of this article.
