# Supplementary material for: Bifunctional Iminophosphorane‐Catalyzed Enantioselective Nitroalkane Addition to Unactivated α,β‐Unsaturated Esters
Source: Angew Chem Int Ed Engl. 2023 Apr 13;62(21):e202303391. doi: 10.1002/anie.202303391 (PMC10946890; doi:10.1002/anie.202303391)
Supplement: Supplementary file 1 — Supporting Information [file ANIE-62-0-s001.pdf]

Angewandte Chemie
Eine Zeitschrift der Gesellschaft Deutscher Chemiker

Supporting Information

Bifunctional Iminophosphorane-Catalyzed Enantioselective Nitroalkane Addition to Unactivated α,β-Unsaturated Esters

D. Rozsar, A. J. M. Farley, I. McLauchlan, B. D. A. Shennan, K. Yamazaki, D. J. Dixon**

Bifunctional Iminophosphorane-Catalyzed Enantioselective Nitroalkane Addition to Unactivated α,β-Unsaturated Esters

Daniel Rozsar, Alistair J. M. Farley,‡ Iain McLauchlan‡, Benjamin D. A. Shennan, Ken Yamazaki* and Darren J. Dixon*

Supporting Information

TABLE OF CONTENTS

General Experimental **1**

Model Reaction Optimization **3**

Catalyst Screen 3

Optimization of Conditions 5

Preparative Scale Syntheses and Catalyst Recovery **6**

Synthetic Procedures **7**

Catalyst Synthesis 7

Starting Material Synthesis 10

γ-Nitroester Synthesis 16

γ-Nitroester Derivative Synthesis 17

Analytical Data **19**

Catalyst Precursors 19

α,β-Unsaturated Esters and Intermediates 22

γ-Nitroesters 28

γ-Nitroester Derivatives 44

NMR Spectra **48**

Catalyst Precursors 48

α,β-Unsaturated Esters 52

γ-Nitroesters 55

γ-Nitroester Derivatives 91

HPLC and SFC Traces **101**

γ-Nitroesters 101

γ-Nitroester Derivatives 133

Computational Studies **140**

Computational Methods 140

Computational Details 141

Computational Data 143

References **186**

[ToC]

General Experimental

Solvents and Reagents

Bulk solutions were evaporated under reduced pressure using a Büchi rotary evaporator. All solvents were commercially supplied or dried by filtration through activated alumina (powder ~150 mesh, pore size 58 Å, basic, Sigma-Aldrich) columns. Petroleum ether refers to the fraction collected between 30-40 °C. All water used was purified *via* a Merck Millipore reverse osmosis purification system prior to use. All reagents were obtained from commercial suppliers and used without further purification. All reactions were performed under an inert atmosphere using oven-dried glassware and standard Schlenk technique, unless otherwise stated. Conjugate addition reactions were conducted using Sigma-Aldrich ReagentPlus® nitromethane (≥99.0).

Chromatography

Manual flash column chromatography (FCC) was carried out using Merck Silicagel 60, particle size 40-63 µm. Automated FCC was carried out using a Biotage Isolera® One Intrument. Eluent systems are provided in v/v% with respect to the more polar component. All reactions were followed by thin-layer chromatography (TLC) when practical, using Merck aluminium-backed Silicagel 60 F254 fluorescent treated silica which was visualised under UV light (λ_{max} = 254 or 365 nm) or by staining with aqueous basic $KMnO_4$, I_2, aqueous acidic vanillin or acidic ninhydrin in *n*-butanol.

Enantiomeric ratios (e.r.) were determined by chiral high-performance liquid chromatography (HPLC) and chiral supercritical fluid chromatography (SFC).

Chiral HPLC analysis was performed on an Agilent 1200 series instrument using an appropriate chiral stationary phase column, specified in the individual experiment, and by comparing the samples with the appropriate racemic mixtures.

Chiral SFC analysis was performed on a Waters Acquity UPC2 instrument using an appropriate chiral stationary phase column, specified in the individual experiment, and by comparing the samples with the appropriate racemic mixtures.

Spectroscopy and Spectrometry

^{1}H and ^{13}C and NMR spectra were recorded using Bruker AVIII HD 400, and Bruker AVII 500 spectrometers using $CDCl_3$ and DMSO-d_6. Chemical shifts (δ) are quoted in parts per million (ppm) relative to tetramethylsilane (δ_{TMS} 0.00 ppm) and referenced to the solvent residual peak (^{1}H: δ_{CDCl3} 7.26 ppm, $\delta_{DMSO\text{-}d6}$ 2.50 ppm; ^{13}C: δ_{CDCl3} 77.16 ppm, $\delta_{DMSO\text{-}d6}$ 39.52 ppm). Coupling constants (*J*) are quoted in Hertz (Hz), rounded to the nearest 0.1 Hz. The ^{1}H NMR spectra are reported as follows: ppm (multiplicity, coupling constants, number of protons, assignment). Two-dimensional (COSY, HSQC, HMBC) NMR spectroscopy was utilised to assist the assignment. Spectra were analyzed using Mestrelab MestReNova 14.2.0 software.

Low resolution mass spectra (LRMS) were recorded on a Waters LCT Premier mass spectrometer operating in positive and negative ionisation modes. High resolution mass spectra (HRMS) were recorded on a Bruker µTOF mass spectrometer. IR spectra were recorded on a Bruker Tensor 27 FT-IR spectrometer on a diamond ATR module. Absorption maxima (ν_{max}) are reported in wavenumbers (cm^{-1}). Only selected absorption maxima are reported.

Melting Points and Specific Rotations

Melting points were recorded in degrees Celsius (°C), using a Leica Galen III hot-stage microscope apparatus and are reported uncorrected.

Specific rotations ($[\alpha]_D^T$) are reported in 10^{-1} deg·cm^2 g^{-1}; D refers to the D-line of sodium (589 nm); temperatures (T) are given in degrees Celsius (°C). Specific rotations were calculated from optical rotations

measured using a Perkin Elmer Model 341 polarimeter with a sodium lamp and a cell length of 1 dm, concentrations (*c*) are reported in g/100 mL.

Naming of Compounds

Compound names were generated using MarvinSketch 20.21 following IUPAC nomenclature.

Model Reaction Optimization

Catalyst Screen

BIMP catalysts were prepared according to **Procedure 18**. To the crude BIMP catalyst was added solvent (if applicable), $MeNO_2$, and methyl-(*E*)-crotonate. Reactions were quenched by passing the reaction mixture through a short silica plug, eluting with $CDCl_3$. Yields were determined by ^{1}H NMR by comparing multiple product and starting material peaks, unless otherwise noted. Enantiomeric excesses were determined by chiral HPLC.

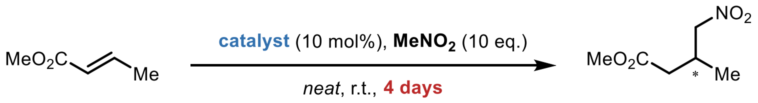

1a 0.1 mmol — **3a**

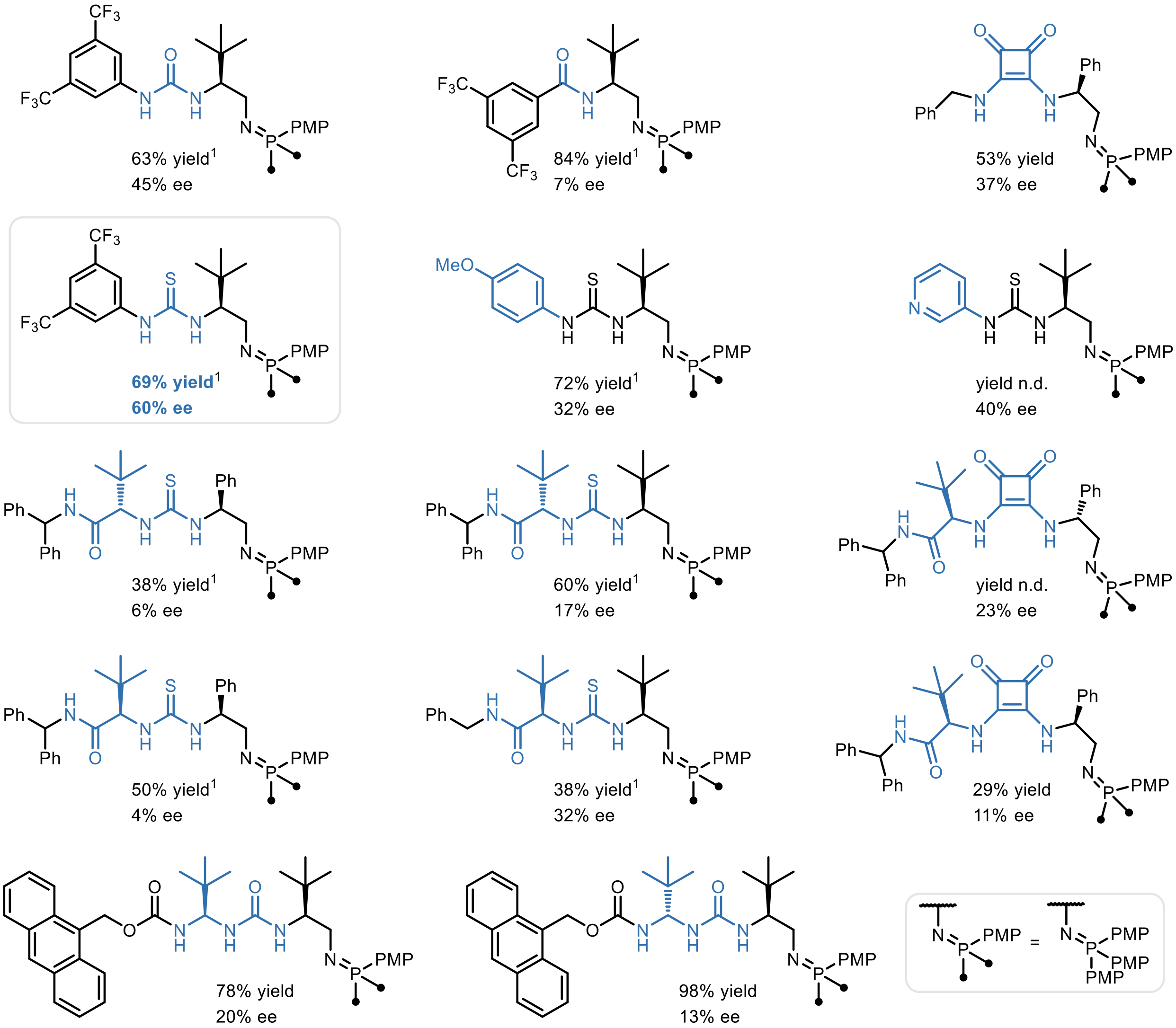

Scheme S1. Initial broad BIMP catalyst screen. PMP = 4-methoxyphenyl. [1]Isolated yield.

[ToC]

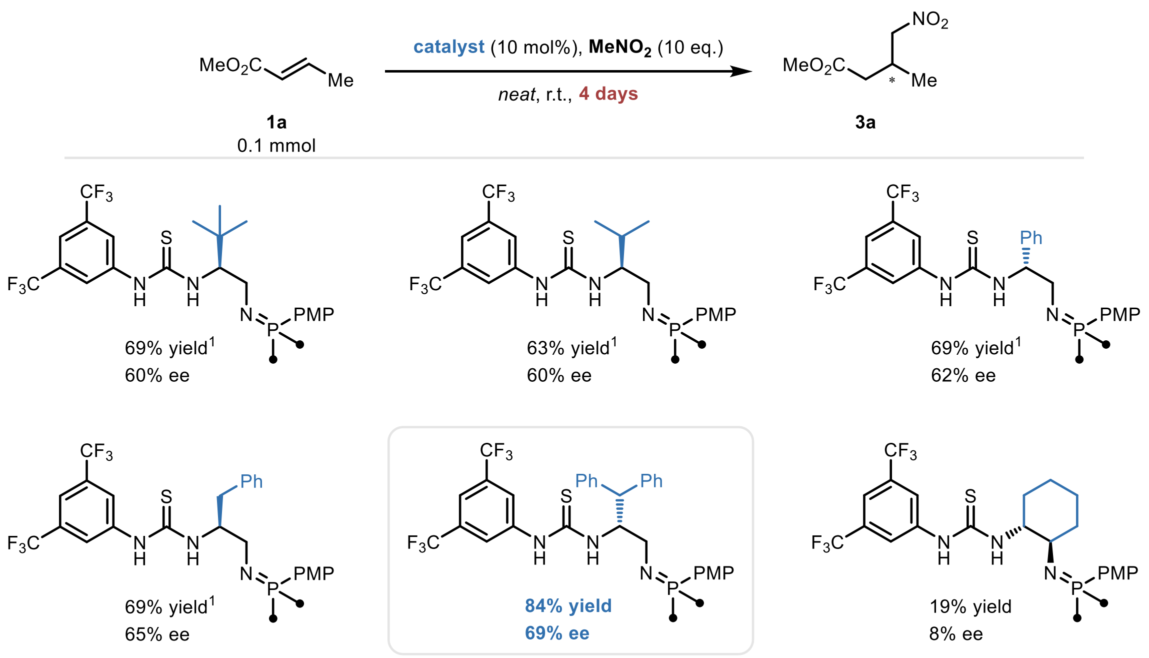

Scheme S2. Stereocenter-substituent screen. PMP = *para*-methoxyphenyl. [1]Isolated yield.

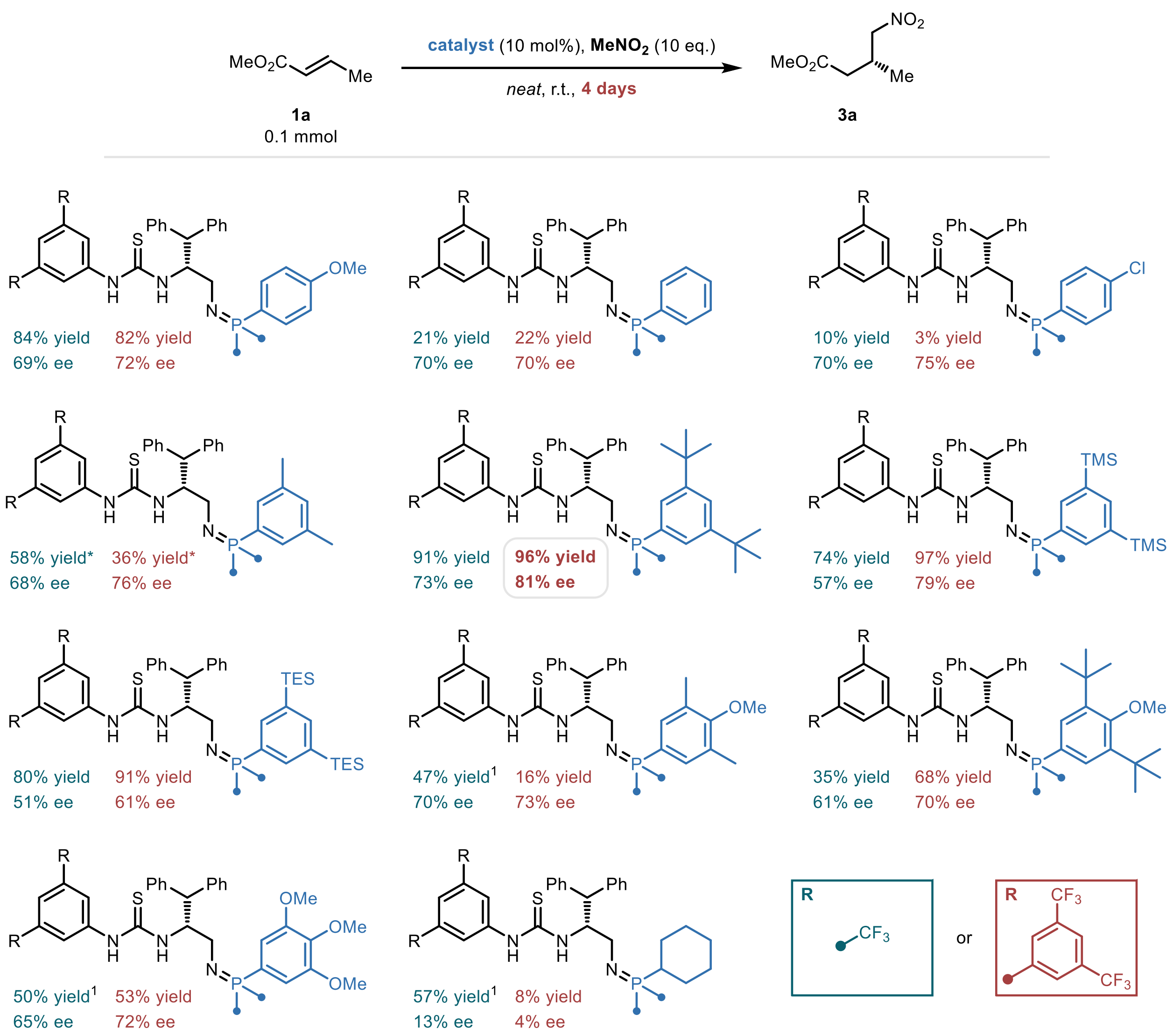

Scheme S3. Catalyst fine-tuning. *Determined after 2 days. [1]Isolated yield.

Optimization of Conditions

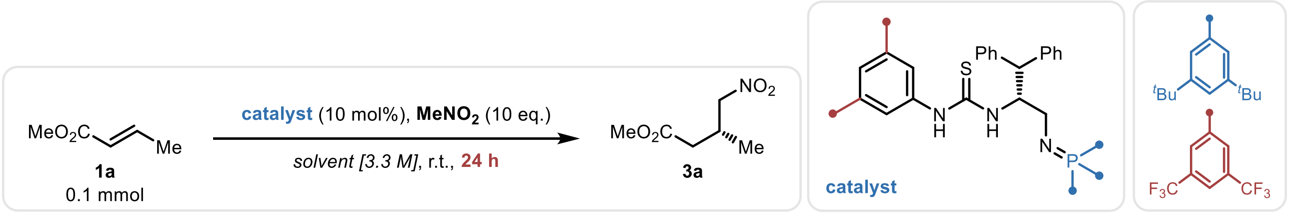

entry	solvent	yield	ee
1[1]	-	71%	81%
2	benzene	72%	86%
3	toluene	64%	84%
4	xylene	68%	84%
5	chlorobenzene	68%	84%
6	trifluorotoluene	64%	83%
7	Et_2O	66%	84%
8	TBME	75%	85%
9	CPME	76%	85%
10	1,2-DME	65%	84%
11	THF	81%	84%
12	2-Me-THF	67%	86%
13	dioxane	78%	87%
14	CH_2Cl_2	48%	80%
15	$CHCl_3$	34%	80%
16	EtOAc	70%	82%
17	acetone	37%	n.d.
18	MeCN	55%	81%
19	EtOH	21%	65%
**20[2]**	**cyclohexane**	**88%**	**90%**
**21[1]**	**Me-cyclohexane**	**78%**	**90%**
22	cyclopentane	72%	86%
**23**	***trans*-decalin**	**79%**	**89%**
24	*n*-pentane	61%	85%
25	*n*-hexane	58%	85%
26	*n*-heptane	62%	85%
27	*n*-octane	59%	84%
28	*n*-decane	62%	84%
29	perfluorohexane	72%	83%
30	perfluorodecalin	74%	82%

Scheme S4. Solvent screen. [1]Average result of 3 experiments. [2]Average result of 2 experiments.

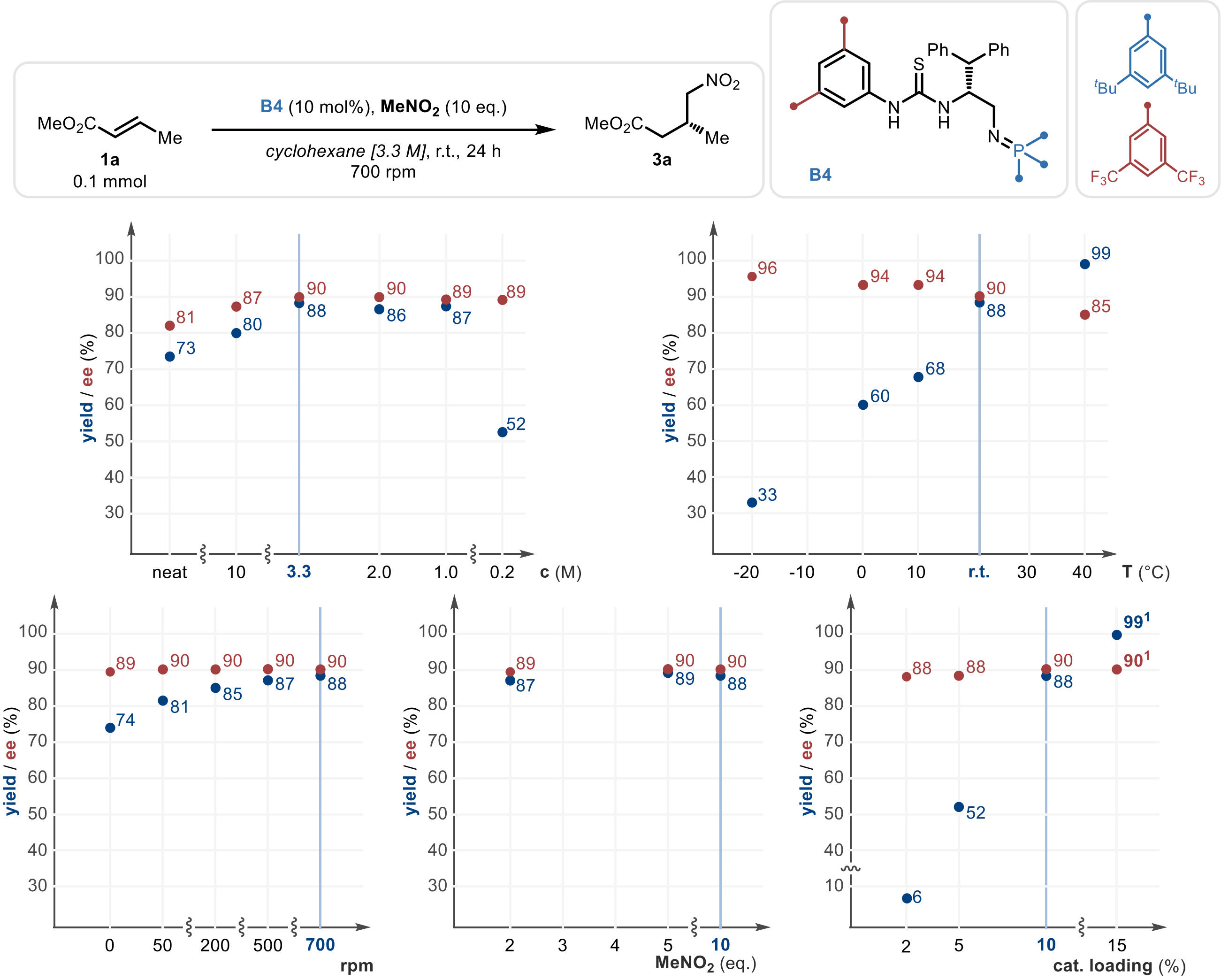

Scheme S5. Condition screen. Each graph shows the effects of changing one condition, other conditions are fixed (as shown in the reaction scheme). Unchanged conditions are highlighted. [1]Optimized conditions: 3.0 eq. $MeNO_2$, 15 mol% catalyst, 3.0 M concentration, r.t., 24 h, 700 rpm; 0.3 mmol scale, isolated yield.

[ToC]

PREPARATIVE SCALE SYNTHESES AND CATALYST RECOVERY

The two gram scale reactions were set up and purified in an analogous manner. Azide **S5** (324 mg, 0.40 mmol, 4.0 mol%) and phosphine **S7** (239 mg, 0.40 mmol, 4.0 mol%) were weighed in a 15 mL vial, then anhydrous THF (8.0 mL, 0.05 M) was added. The solution was stirred for 24 hours at r.t., volatiles were removed under a stream of N_2, then the vial was placed under vacuum for 20 minutes. To the crude catalyst was added cyclohexane (3.33 mL, 3.0 M), nitromethane (1.62 mL, 30 mmol, 3.0 eq.), then the corresponding ester (**1a**: 1.06 mL, 10 mmol, 1.0 eq; **1j**: 1.49 mL, 10 mmol, 1.0 eq.). The reaction mixtures were stirred at 1000 rpm for 48 hours, then a minimal amount of CH_2Cl_2 was added to homogenise the suspensions. The solutions were directly loaded onto the column. Product **3aS** was obtained as a colorless oil after FCC (Biotage ZIP® 120 g cartridge, [pentane : CH_2Cl_2 25%] : EtOAc 0% to 10%, 1.06 g, 6.58 mmol, 66%, 95:5 er). Analytical data were identical with those of product **3a**. Product **3aS** was obtained as pale yellow oil after FCC (Biotage ZIP® 120 g cartridge, [pentane : CH_2Cl_2 25%] : EtOAc 0% to 10%, 2.29 g, 9.99 mmol, 99%, 93.5:6.5 er). Analytical data were identical with those of product **3j.**

Catalyst recovery: After the isolation of both products, the solvent polarity was increased ([pentane : CH_2Cl_2 25%] : EtOAc 10% to 100%) to elute BIMP catalyst **B4**. Volatiles were removed *in vacuo*, then the solids were dissolved in 100 mL pentane. The solution was washed with 3.0 M aqueous NaOH (2 × 100 mL) and the organic phase was dried over anhydrous Na_2SO_4. Volatiles were removed *in vacuo* to afford **B4** (787 mg, 0.57 mmol, 71% recovery). The recovered catalyst was resubjected to the optimized reaction conditions according to modified **Procedure 19** on a 0.1 mmol scale using substrate **1a** (99% conversion to product **3a**, 95:5 er).

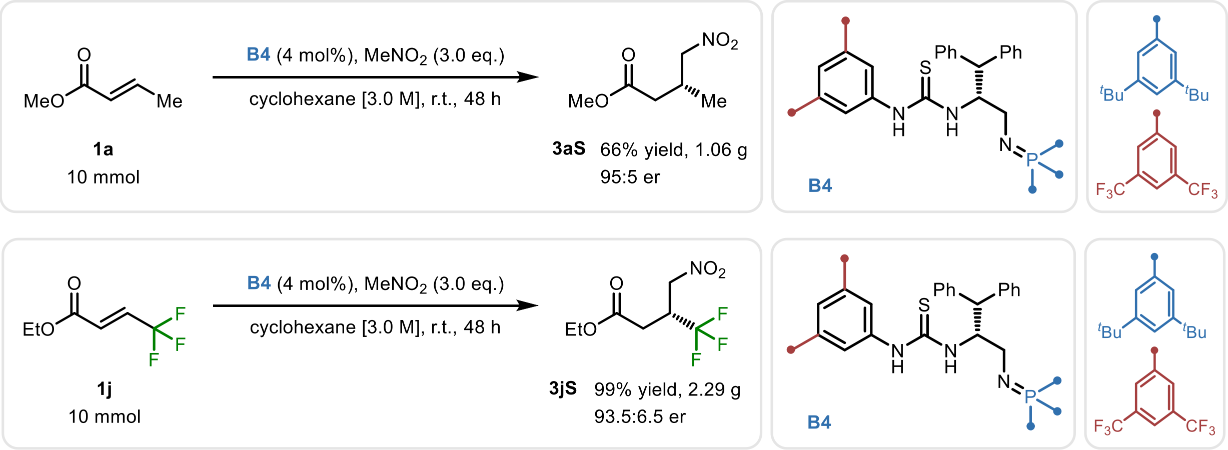

Scheme S6. Preparative scale synthesis of **3aS** and **3jS**.

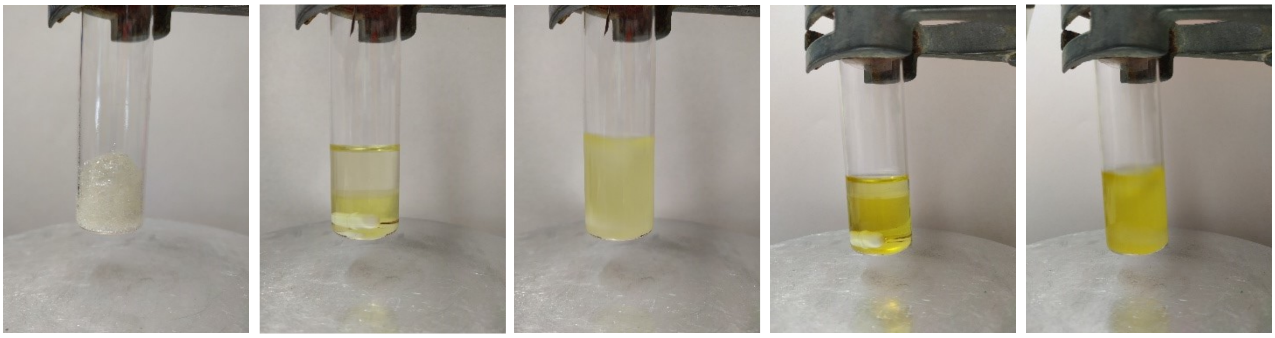

Figure S1. Preparative scale synthesis photos. Photos from left to right: activated crude BIMP catalyst; reaction mixture (**3aS**, 5 minutes after set-up, 0 rpm); reaction mixture (**3aS**, 5 minutes after set-up, 1000 rpm); reaction mixture (**3jS**, 24 hours after set-up, 0 rpm); reaction mixture (**3jS**, 24 hours after set-up, 1000 rpm).

SYNTHETIC PROCEDURES

Catalyst Synthesis

Procedure 01

[Suzuki-Miyaura coupling]

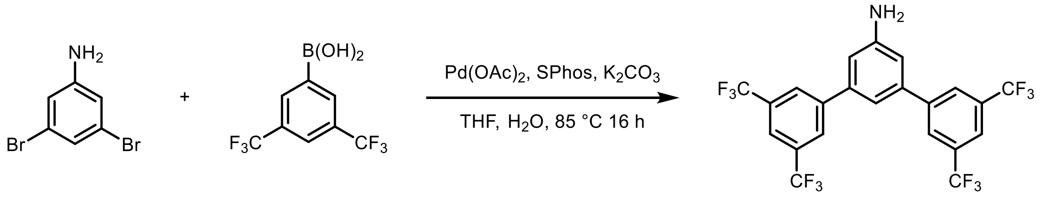

S1

Based on a modified literature procedure,[1] 3,5-dibromoaniline (2.0 g, 7.9 mmol, 1.0 eq.), (3,5-bis(trifluoromethyl)phenyl)boronic acid (7.2 g, 27.9 mmol, 3.5 eq.), SPhos (326 mg, 0.79 mmol, 0.1 eq.) and K_2CO_3 (5.46 g, 39.5 mmol, 5.0 eq.) were suspended in degassed THF : H_2O (4:1, 100 mL, 0.08 M). $Pd(OAc)_2$ (89 mg, 0.395 mmol, 0.5 mol %) was then added to the mixture. The reaction was then stirred and refluxed for 16 hours. The mixture was cooled to r.t. and was diluted with 20 mL H_2O. The mixture was extracted with CH_2Cl_2 (3 x 30 mL). The combined organic layers were washed with brine (15 mL), dried over anhydrous $MgSO_4$ and filtered on a Celite® column eluting with CH_2Cl_2. The solvent was removed *in vacuo*. Purification by FCC (pentane : EtOAc 0% to 10%) yielded product **S1** (3.88 g, 7.5 mmol, 95%, colorless solid).

Procedure 02

[isothiocyanate synthesis]

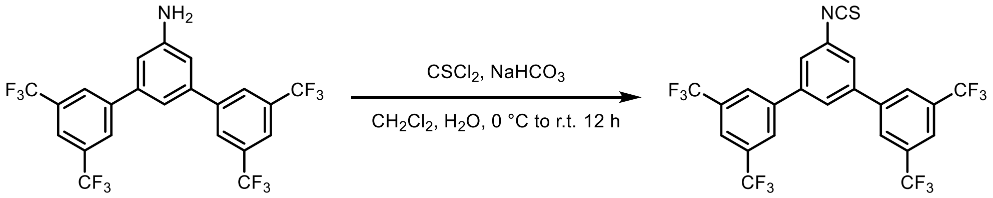

To a suspension of **S1** (1.85 g, 3.6 mmol, 1.0 eq.) in CH_2Cl_2 (6 mL, 0.6 M) and H_2O (6 mL, 0.6 M) was added $NaHCO_3$ (1.51 g, 18 mmol, 5.0 eq.). The mixture was cooled to 0 °C and, while stirring vigorously, thiophosgene (0.28 mL, 3.6 mmol, 1.0 eq.) was added portionwise. The mixture was warmed to r.t. and stirred for 12 hours. The reaction was quenched by the addition of 25 mL sat. aq. $NaHCO_3$. The mixture was extracted with CH_2Cl_2 (3 x 20 mL). The combined organic layers were washed with brine, dried over Na_2SO_4 and filtered. The filtrate was concentrated *in vacuo*. Purification by passing the crude mixture through a short silica plug eluting with CH_2Cl_2 (50 mL) yielded product **S2** (1.79 g, 3.20 mmol, 89%, yellow solid).

[[ToC](#)]

Procedure 03

[BIMP precursor syntheses]

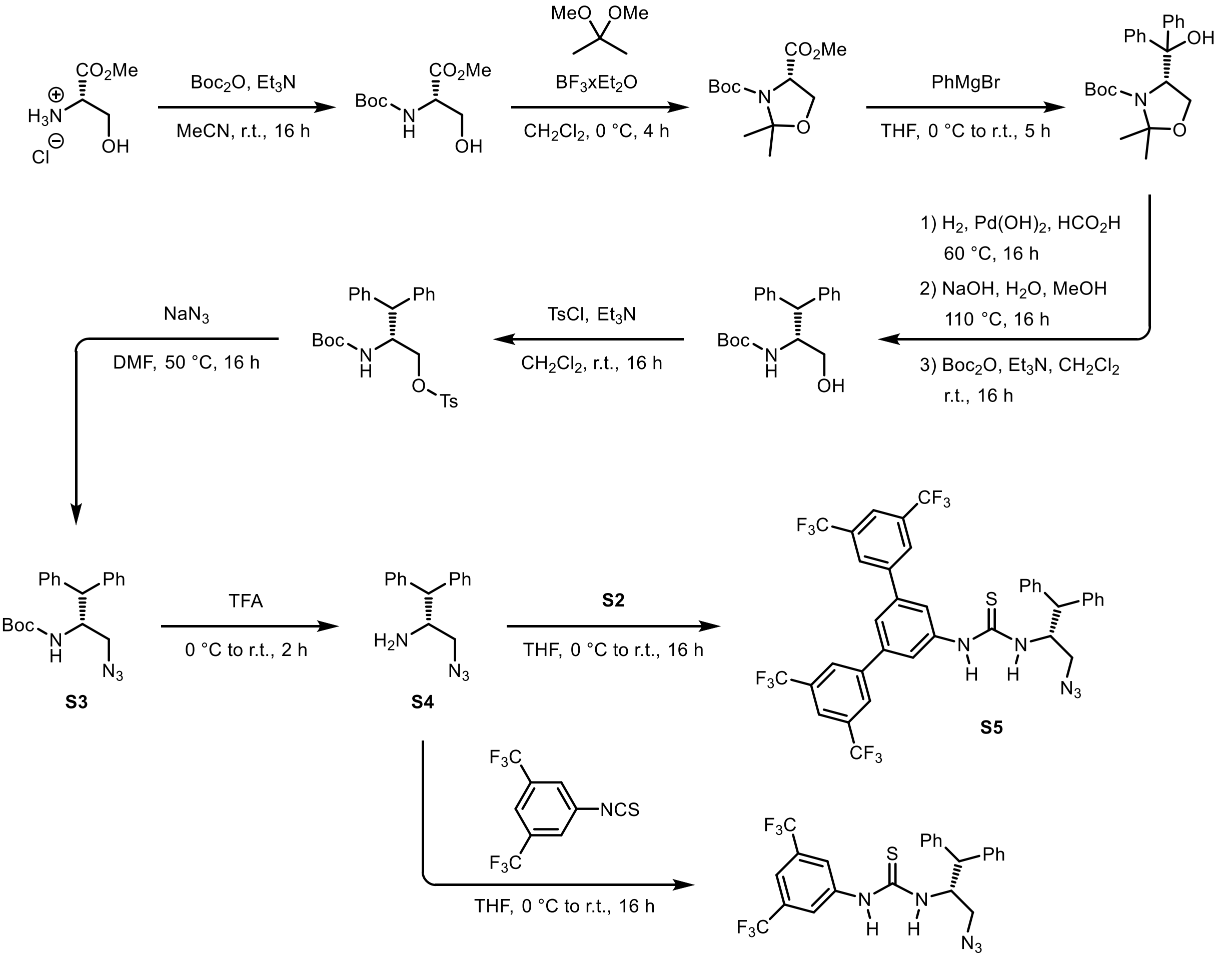

N-Boc protected aminoazide **S3** was synthesized following a procedure previously reported by our group.[2]

S3 (352 mg, 1.0 mmol, 1.0 eq.) was cooled to 0 °C, then TFA (1.0 mL, 1.0 M) was added dropwise. The mixture was allowed to warm to r.t., then was stirred for 2 h. Excess TFA was removed with a stream of N_2. The reaction was quenched with aq. NaOH (10 mL, 1.0 M) and was extracted with Et_2O (3 x 10 mL). Volatiles were removed *in vacuo* to yield amino azide **S4**. Product **S4** was dissolved in dry THF (3.3 mL, 0.3 M) and was added to isothiocyanate **S2** (559 mg, 1.0 mmol, 1.0 eq.) portionwise. The reaction mixture was stirred under N_2 for 16 hours at r.t. Volatiles were then removed *in vacuo*. Purification by FCC (pentane : EtOAc 0% to 7%) yielded azide **S5** (557 mg, 0.69 mmol, 69%, colorless solid). The synthesis was repeated multiple times with similar results.

Azide **S6** was synthesized in an analogous manner.[2]

[ToC]

Procedure 04

[phosphine synthesis]

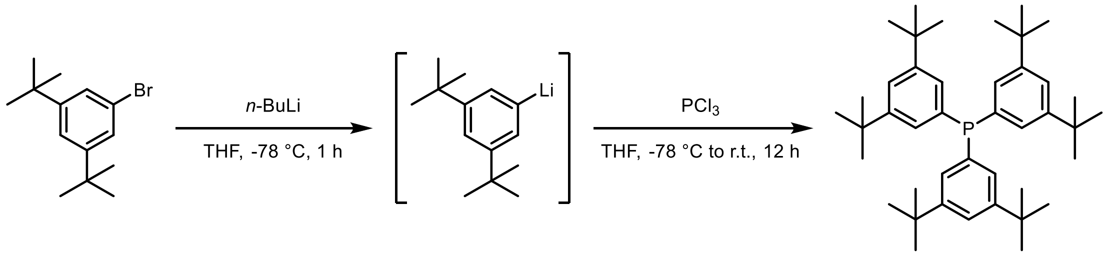

S7

According to a modified literature procedure,[3] 1-bromo-3,5-di-*tert*-butylbenzene (10.0 g, 37.1 mmol, 5.0 eq.) was dissolved in degassed THF (85 mL, 0.44 M) then the solution was cooled to -78 °C. To the solution was added *n*-BuLi (2.5 M in hexanes, 15.0 mL, 37.1 mmol, 5.0 eq.) dropwise over 20 minutes. The mixture was stirred for 1 hour with the temperature not exceeding -70 °C. PCl_3 (0.645 mL, 7.4 mmol, 1.0 eq.) dissolved in degassed THF (5.0 mL, 90 mL in total, 0.082 M) was added to the solution dropwise over 20 minutes at -78 °C. The reaction mixture was stirred at -78 °C for 1 hour, then it was slowly warmed up to room temperature, and was stirred for an additional 12 hours. Water (20 mL) was added to the mixture, then it was extracted with pentane (3 × 40 mL). The combined organic layers were washed with concentrated aqueous $NaHCO_3$ and brine (30 mL both), then were dried over anhydrous $MgSO_4$. Volatiles were removed *in vacuo*. To the residue was added MeOH (15 mL), then the mixture was cooled and kept at -20 °C for 8 hours. The product was filtered, washed with -20 °C MeOH (2 × 10 mL). The residue was dissolved in refluxing MeOH (40 mL), then was cooled to -20 °C. The solids were filtered and washed with -20 °C MeOH (2 × 5 mL). Both recrystallizations were performed in degassed MeOH. Phosphine **S7** was obtained as a colorless powder (3.57 g, 5.97 mmol, 81%).

[ToC]

Starting Material Synthesis

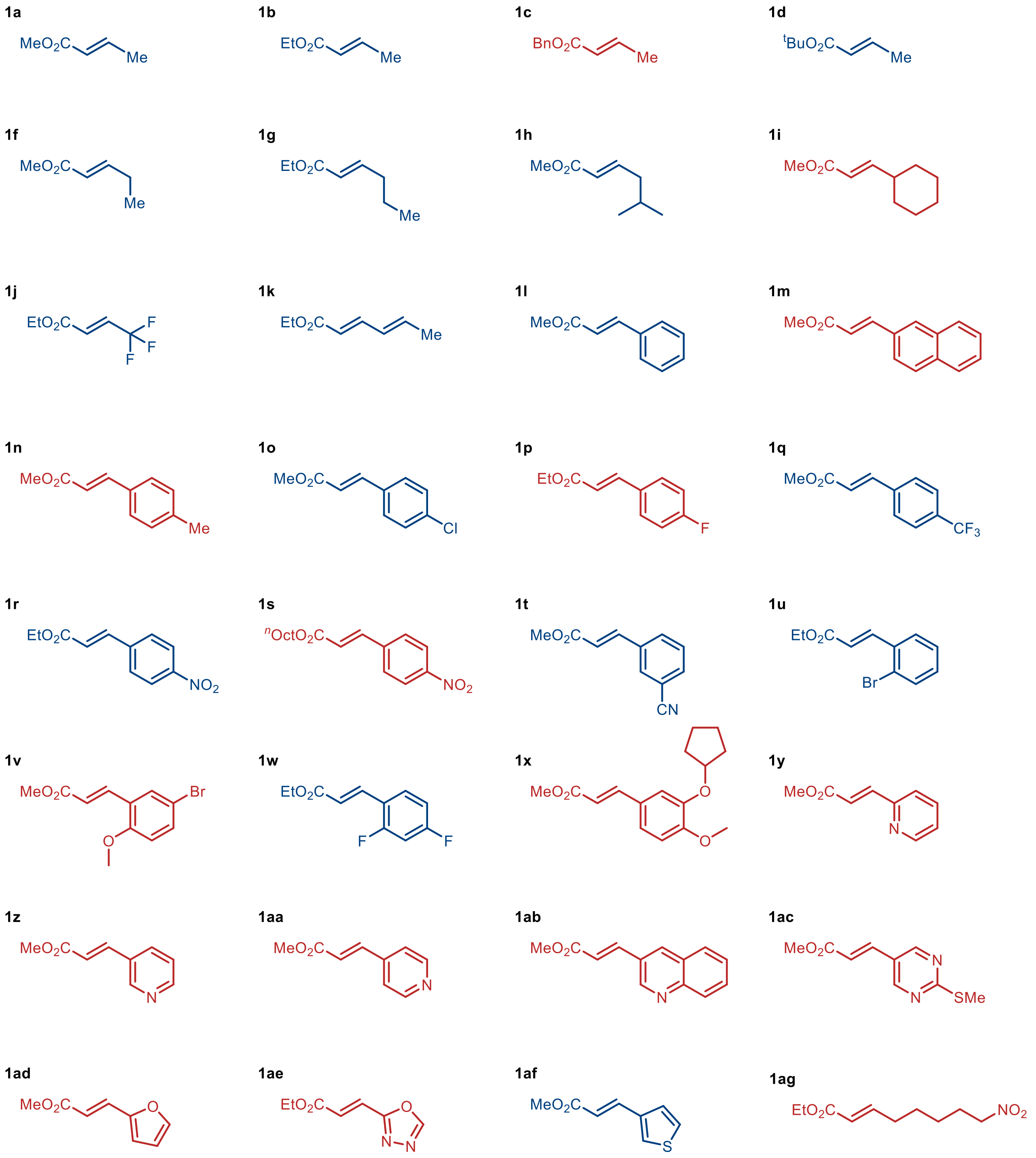

Figure S2. α,β-Unsaturated ester starting materials. Compounds denoted in blue were purchased from commercial suppliers, and used without further purification. Compounds denoted in red were synthesized according to synthetic procedures described below.

[ToC]

Procedure 05

[benzylation]

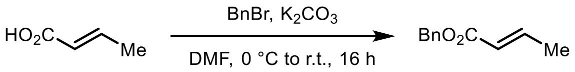

1c

Crotonic acid (1.81 g, 21 mmol, 1.1 eq.) was dissolved in DMF (12 mL, 1.65 M), then the mixture was cooled to 0 °C. To this solution was added K_2CO_3 (1.66 g, 12 mmol, 0.6 eq.), and benzyl bromide (2.38 mL, 20 mmol, 1.0 eq.). The mixture was warmed to r.t. and stirred for 16 hours. The crude mixture was filtered on a Celite® column eluting with Et_2O (150 mL), then the obtained solution was extracted with H_2O (80 mL), $NaHCO_3$ (cc., aq., 80 mL) and brine (80 mL), and was dried over anhydrous $MgSO_4$. Volatiles were removed *in vacuo*, and the product was used without further purification (3.19 g, 18.1 mmol, 91%, colorless oil).

Procedure 06

[methylation using MeI]

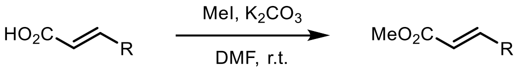

1aa, **1ac**

The corresponding α,β-unsaturated carboxylic acid (1.0 eq.) and K_2CO_3 (1.1 eq.) were suspended in DMF (0.5 M) under a N_2 atmosphere. MeI (1.1 eq.) was added in one portion, and the reaction was stirred under N_2 at r.t. until the consumption of the starting material, as indicated by TLC. The reaction was quenched by the addition of sat. aq. NH_4Cl (5 mL / 1 mmol s. m.) and was stirred for 30 minutes. The mixture was extracted with Et_2O (2 x 10 mL / 1 mmol s. m.). The combined organic layers were washed with brine (4 mL / 1 mmol s. m.), dried over anhydrous $MgSO_4$ and filtered. The solvent was removed *in vacuo*, and purification by FCC, as specified in the individual experiment, yielded the product.

Procedure 07

[methylation using TMS-CHN_2]

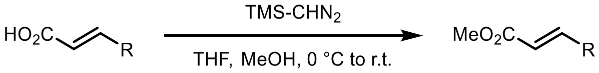

1n, **1v**, **1y**, **1z**

A solution of the corresponding α,β-unsaturated carboxylic acid (1.0 eq.) in THF:MeOH 4:1 (0.3 M) was cooled to 0 °C under a N_2 atmosphere. (Trimethylsilyl)diazomethane (2.4 eq., 2.0 M solution in Et_2O) was added dropwise and the solution was stirred for 1 hour or until all the starting material was consumed, as indicated by TLC. The reaction was quenched by the addition of sat. aq. NH_4Cl (5 mL / 1 mmol s. m.). The mixture was extracted with EtOAc (2 x 10 mL / 1 mmol s. m.). The combined organic layers were washed with brine (4 mL / 1 mmol s. m.), dried over $MgSO_4$ and filtered. The solvent was removed under reduced pressure to afford the products which were used without further purification.

[ToC]

Procedure 08

[alkenylation]

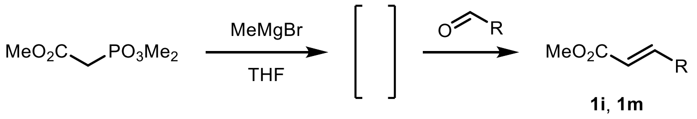

To a solution of trimethyl phosphonoacetate (1.0 eq.) in THF (0.14 M), at r.t. and under N_2, was added MeMgBr (0.86 eq.) dropwise. The solution was stirred at r.t. for 20 minutes before the aldehyde (0.94 eq.) was added in one portion. The solution was stirred at r.t. for 1 hour. The reaction was quenched by the addition of sat. aq. NH_4Cl (5 mL / 1 mmol s. m.) and was stirred for 20 minutes. The mixture was extracted with Et_2O (2 x 10 mL / 1 mmol s. m.). The combined organic layers were washed with brine (15 mL), dried over anhydrous Na_2SO_4 and filtered. The solvent was removed *in vacuo*. Purification by vacuum distillation or FCC, as specified in the individual experiment, yielded the product.

Procedure 09

[alkenylation]

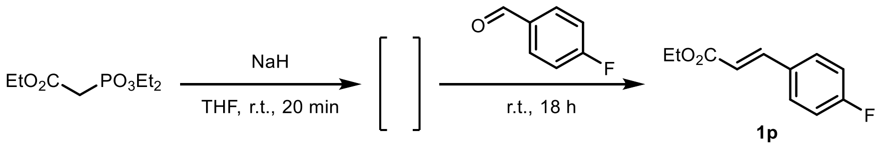

NaH (600 mg, 15 mmol, 1.5 eq., 60% in mineral oil) was suspended in THF (67 mL, 0.15 M) at r.t., then triethyl phosphonoacetate (3.0 mL, 15 mmol, 1.5 eq.) was added dropwise. The suspension was stirred for 10 minutes at r.t., then 4-fluorobenzaldehyde (1.55 mL, 10 mmol, 1.0 eq.) was added. The reaction mixture was stirred at r.t. for 18 hours, then 100 mL H_2O was added and the mixture was extracted with Et_2O (3 x 50 mL). The combined organic layers were washed with brine (100 mL), then were dried over anhydrous Na_2SO_4. Purification by FCC (pentane : EtOAc 0% to 20%) yielded **1p** (1.92 g, 9.90 mmol, 99%, colorless solid).

Procedure 10

[alkylation of **6a**]

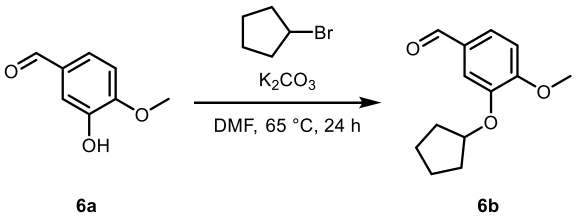

Isovanilin **6a** (1.52 g, 10 mmol, 1.0 eq.) and K_2CO_3 (2.07 g, 15 mmol, 1.5 eq.) were mixed under a N_2 atmosphere. Cyclopentyl bromide (1.60 mL, 15 mmol, 1.5 eq.) was dissolved in degassed DMF (10 mL, 1.0 M). This solution was added to the solids, then the suspension was warmed to 65 °C and was stirred for 24 h under N_2. The mixture was then cooled to r.t., then it was filtered on a Celite® column eluting with Et_2O (25 mL). The solution was extracted with H_2O (100 mL). The aqueous layer was washed with Et_2O (2 x 50 mL), then the combined organic layers were washed with brine (70 mL) and were dried over anhydrous Na_2SO_4. Volatiles were removed *in vacuo* and **6b** was used in the next step without further purification (2.25 g, 10 mmol, quant., yellow oil).

[ToC]

Procedure 11

[alkenylation of **6b**]

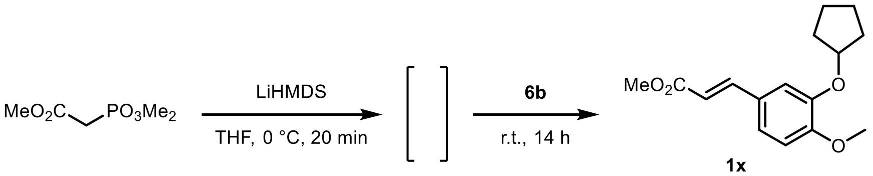

1x

According to a modified literature procedure,[4] to a solution of trimethyl phosphonoacetate (1.32 mL, 12 mmol, 1.2 eq.) in THF (6 mL, 2 M) was added LiHMDS solution (1 M in THF, 12.0 mL, 1.0 eq.) dropwise at 0 °C, then the mixture was stirred for 20 minutes. To this solution was added **S8** (2.25 g, 10.0 mmol in 5 mL THF) dropwise, then the reaction mixture was stirred at r.t. for 14 hours, then 50 mL H_2O was added and the mixture was extracted with Et_2O (3 x 50 mL). The combined organic layers were washed with brine and dried over anhydrous $MgSO_4$, then volatiles were removed *in vacuo*. Purification by FCC (pentane : EtOAc 0% to 30%) yielded **1x** (2.54 g, 9.21 mmol, 92%, colorless solid).

Procedure 12

[alkenylation of furfural]

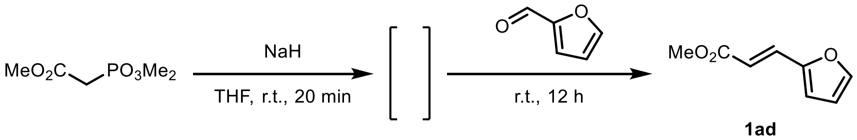

1ad

A suspension of NaH (0.88 g, 22 mmol, 1.1 eq.) in THF (60 ml ,~ 4 M) under N_2 was cooled to 0 °C. Trimethyl phosphonoacetate (3.56 ml, 3.0 M in Et_2O, 1.1 eq.) was added dropwise. The resulting slurry was stirred for 20 minutes before diluting with 20 mL of THF. Furfural (1.92 g, 20 mmol, 1.0 eq.) was added in one portion before stirring for 12 hours. The mixture was then cooled to 0 °C before quenching by the addition of 30 mL of sat. aq. NH_4Cl and was stirred for 1 hour. The 43 mixture was extracted with Et2O (3 x 20 mL). The combined organic layers were washed with brine (20 mL), dried with Na2SO4 and filtered. The solvent was removed under reduced pressure. Purification by FCC (pentane : EtOAc, 10%) yielded ester **1ad** (1.85 g, 12.2 mmol, 61%, yellow solid).

Procedure 13

[Fischer esterification]

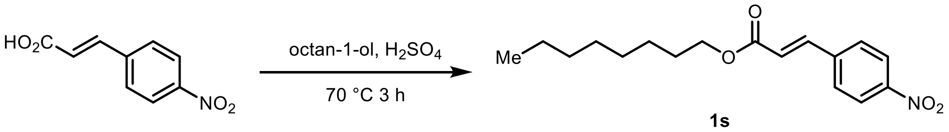

1s

4-Nitrocinnamic acid (1.93 g, 10 mmol, 1.0 eq.) was suspended in octan-1-ol (20 mL, 127 mmol, 13 eq.), then concentrated sulfuric acid (1 mL, 19 mmol, 1.9 eq.) was added. The mixture was warmed up to 70 °C, then was stirred for 3 hours. The mixure was cooled to r.t., then 100 mL 1.0 M aqueous NaOH was added. The solution was extracted with pentane (3 x 100 mL), then the combined organic layers were washed with brine (150 mL), dried over anhydrous $MgSO_4$, and filtered on a Celite® column eluting with Et_2O (50 mL). Volatiles were removed *in vacuo*, then most of the remaining octan-1-ol was removed by vacuum distillation. The residue was then warmed to 60 °C and was stirred under N_2 stream for 48 hours. The resulting crude product

was recrystallyzed from MeOH (10 mL 60 °C to 0 °C), the solids were filtered and washed with cold MeOH (4 x 2 mL) to yield **1s** (550 mg, 1.80 mmol, 18%, colorless solid).

Procedure 14

[Heck-coupling]

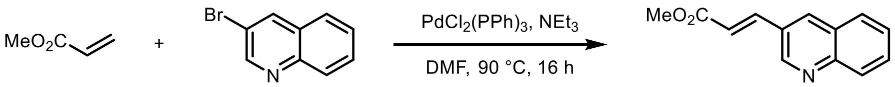

1ab

3-Bromoquinoline (0.62 g, 3.42 mmol, 1.0 eq.), methyl acrylate (2.58 g, 30 mmol, 10 eq.) and triethylamine (1.21 g, 12 mmol, 4.0 eq.) were dissolved in dry degassed DMF (18 mL), then bis(triphenylphosphine)palladium(**II**) dichloride (0.21 g, 0.3 mmol, 10 mol %) was added. The mixture was refluxed at 90 °C for 16 hours. Upon consumption of all starting material, as indicated by TLC, the mixture was diluted with 30 mL of H_2O. After extraction with EtOAc (3 x 20 mL), the combined organic layers were dried over Na_2SO_4 and were concentrated *in vacuo*. Purification by FCC (pentane : EtOAc 20%) afforded **1ab** (178 mg, 2.87 mmol, 84%, yellow solid).

Procedure 15

[1,3,4-oxadiazole synthesis]

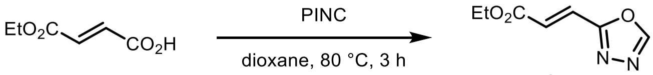

1ae

Based on a modified literature procedure,[5a] mono-ethyl fumarate (720 mg, 5.0 mmol, 1.0 eq.), and (*N*-isocyanoimino)triphenylphosphorane (PINC, 1.66 g, 5.5 mmol, 1.1 eq.) were suspended in dioxane (12.5 mL, 0.4 M). The mixture was warmed to 80 °C and was stirred for 3 hours. Volatiles were removed *in vacuo*, then FCC (pentane : EtOAc 5% to 25%) yielded **1ae** (180 mg, 1.07 mmol, 21%, yellow solid).

Procedure 16

[S_N2 reaction with $NaNO_2$]

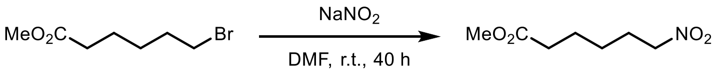

S8

Following a modified literature procedure,[5b] to a solution of methyl 6-bromohexanoate (1.58 mL, 10.0 mmol) in DMF (100 mL) was added $NaNO_2$ (1.04 g, 15.0 mmol). The reaction was stirred for 40 hours and poured onto ice water. The resulting biphasic mixture was extracted with Et_2O (3 x 150 mL) and the organics were combined, washed with brine (2 x 50 mL), dried with Na_2SO_4, filtered and concentrated under reduced pressure. The crude residue was purified by FCC (17:3 pentane:Et_2O) to yield **S8** (696 mg, 4.00 mmol, 40%, colorless oil).

[ToC]

Procedure 17

[Tethered unsaturated nitroester synthesis]

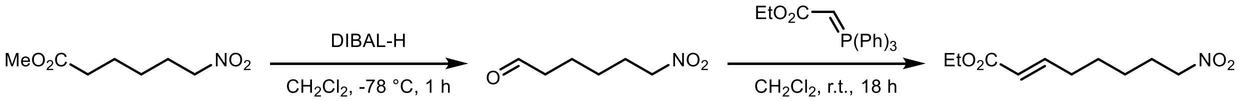

Following a modified literature procedure,[5b] to a solution of compound **S8** (696 mg, 4.0 mmol) in CH_2Cl_2 (28 ml), cooled to -78 °C, was added DIBAL (1.0 M in hexanes, 4.37 mL, 4.37 mmol). The solution was stirred for 1 hour until the starting material was consumed as indicated by TLC (1:1 pentane:Et_2O) at which point 1.0 M aq. HCl (5 mL) was added, followed by H_2O (10 mL). The mixture was warmed to room temperature, diluted with H_2O and extracted with CH_2Cl_2 (3 x 50 mL). The combined organics were dried with Na_2SO_4, filtered and concentrated under reduced pressure.

To crude **S9** was added CH_2Cl_2 (40 mL) and (carbethoxymethylene)triphenylphosphorane (1.5 g, 4.4 mmol). The mixture was stirred at room temperature for 18 hours before the addition of sat. aq. NH_4Cl. The resulting biphasic mixture was extracted with CH_2Cl_2 (3 x 50 mL) and the combined organics were dried with Na_2SO_4, filtered and concentrated under reduced pressure. The crude residue was purified by FCC (1:1 pentane:Et_2O) to yield **1ag** (570 mg, 2.65 mmol, 67%, colorless oil).

[ToC]

γ-Nitroester Synthesis

Procedure 18

[*in situ* generation of BIMP catalysts]

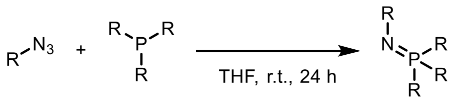

To the corresponding organoazide (1.0 eq.) and trivalent phosphine (1.0 eq.) under argon atmosphere was added THF (0.05 M) and the reaction mixture was stirred at room temperature for 24 hours. Volatiles were removed under a stream of nitrogen, then the crude BIMP catalyst was dried under vacuum for 15 minutes, and was used without further purification.

Procedure 19

[synthesis of enantiomerically enriched γ-nitroesters]

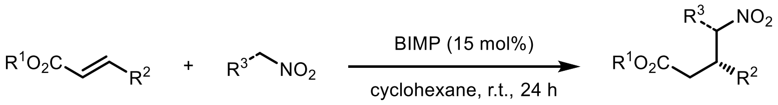

Solid substrates: The corresponding α,β-unsaturated ester (0.3 mmol, 1.0 eq.), cyclohexane (100 μL, 3.0 M), then the corresponding nitroalkane (0.9 mmol, 3.0 eq.) were added to the BIMP catalyst (0.045 mmol, 15 mol%) under air in a 1.5 mL vial.

Liquid substrates: Cyclohexane (100 μL, 3.0 M), the corresponding nitroalkane (0.9 mmol, 3.0 eq.), then the corresponding α,β-unsaturated ester (0.3 mmol, 1.0 eq.), were added to the BIMP catalyst (0.045 mmol, 15 mol%) under air in a 1.5 mL vial.

Reactions were typically stirred for 24 hours at 700 rpm, and were monitored by TLC. Crude products were purified by FCC as specified in the individual experiment. The two enantiomers were separated by analytical chiral HPLC using conditions specified in the individual experiment.

Procedure 20

[synthesis of racemic γ-nitroesters]

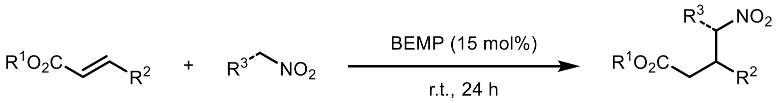

The corresponding α,β-unsaturated ester (0.1 mmol, 1.0 eq.) and nitroalkane (1.0 mmol, 10 eq.) were added to a 1.5 mL vial under air. BEMP (2-tert-butylimino-2-diethylamino-1,3-dimethylperhydro-1,3,2-diazaphosphorine) achiral superbase (4.4 μL, 0.015 mmol, 15 mol%) was added, then the mixture was stirred for 24 h. Reaction progress was monitored by TLC, and crude products were isolated by preparative TLC. The two enantiomers were separated by analytical chiral HPLC using conditions specified in the individual experiment.

γ-Nitroester Derivative Synthesis

Procedure 21

[δ-lactam synthesis]

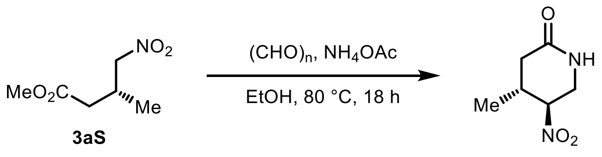

Following a modified literature procedure,[6] to a solution of product **3aS** (161 mg, 1.0 mmol, 1.0 eq.) and NH_4OAc (154 mg, 2.0 mmol, 2.0 eq.) in EtOH (0.33 mL, 3.0 M) was added paraformaldehyde (30 mg, 1.0 mmol, 1.0 eq.). The mixture was warmed to 80 °C for 24 hours, then was cooled to r.t. Volatiles were removed *in vacuo*, and purification by FCC (pentane : EtOAc : EtOH, 6 : 3 : 1) yielded product **4a** as a yellow solid (86%, 136 mg, 0.86 mmol, 95.5:4.5 e.r.).

Procedure 22

[*N*-benzyl δ-lactam synthesis]

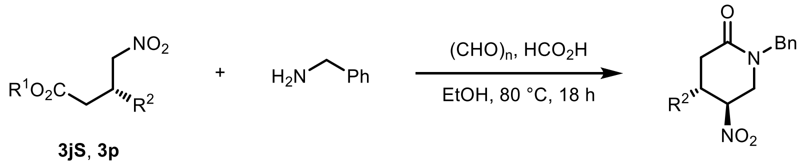

The corresponding γ-nitroester (1.0 eq.), benzylamine (2.0 eq.), paraformaldehyde (1.0 eq.) and AcOH (0.2 mL / 1.0 mmol γ-nitroester) were dissolved in EtOH (0.2 M). The mixture was warmed to 80 °C and was stirred for 18 hours.

In case of product **4b** CH_2Cl_2 was added to the reaction mixture and the organic phase was washed with 1 M aq. HCl, cc. $NaHCO_3$ and brine, and was dried over anhydrous $MgSO_4$. Volatiles were removed *in vacuo* to yield product **4b** as a brown oil (99%, 150 mg, 0.50 mmol, 95:5 d.r., 93:7 e.r.; the product decomposes on silica gel).

In case of product **4c**, to the reaction mixture was added water, the it was extracted with CH_2Cl_2. The combined organics were washed with brine, dried over anhydrous $MgSO_4$, and volatiles were removed *in vacuo*. Preparative TLC (eluent: hexane : EtOAc 60%) yielded **4c** as a colorless oil (72%, 29.6 mg, 0.09 mmol, 95:5 d.r., 95:5 e.r. [major diastereomr], 96:4 e.r. [minor diastereomer]).

[ToC]

Procedure 23

[synthesis of γ-lactams]

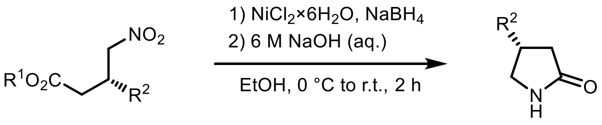

3aS, **3jS**, **3n**, **3x** **5a-c**

The corresponding γ-nitroester (1.0 eq.) and $NiCl \times 6H_2O$ (1.0 eq.) were suspended in EtOH (0.2 M), then the mixture was cooled to 0 °C. $NaBH_4$ (11.0 eq.) was slowly added, then the mixture was stirred for 1 h at 0 °C. EtOH (3.0 mL / 1.0 mmol γ-nitroester) was added, then NaOH (6 M aqueous solution, 1.3 mL / 1.0 mmol γ-nitroester) was added dropwise, then the mixture was allowed to warm to r.t., then was stirred for 30 minutes. Concentrated NH_4Cl (20 mL / 1.0 mmol γ-nitroester) was added and the mixture was extracted with CH_2Cl_2 (3×10 mL / 1.0 mmol γ-nitroester). The combined organics were extracted with brine and dried over anhydrous Na_2SO_4 and filtered on a Celite® column eluting with CH_2Cl_2. Volatiles were removed *in vacuo* to yield the desired 2-pyrrolidinone.

Analytical Data

Catalyst Precursors

5-[3,5-bis(Trifluoromethyl)phenyl]-3',5'-bis(trifluoromethyl)-[1,1'-biphenyl]-3-amine (**S1**)

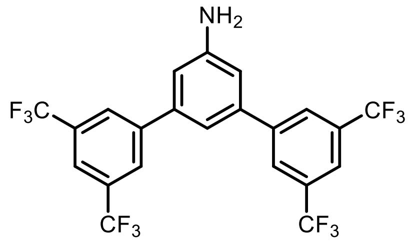

Product **S1** was prepared according to **Procedure 01**.

yield: 3.88 g (7.50 mmol, 95%, colorless solid).

^{1}H NMR (400 MHz, $CDCl_3$) δ 8.02 (s, 4H), 7.89 (s, 2H), 7.11 (t, *J* = 1.5 Hz, 1H), 6.95 (d, *J* = 1.6 Hz, 2H), 4.02 (br. s, 2H).

^{13}C NMR (101 MHz, $CDCl_3$) δ 148.1, 143.2, 140.9, 132.3 (q, *J* = 33.4 Hz), 127.5, 123.5 (q, *J* = 272.9 Hz), 121.5, 116.5, 114.1.

^{19}F NMR (377 MHz, $CDCl_3$) δ -62.8.

Analytical data were consistent with those reported in the literature.[1]

3-[3,5-bis(Trifluoromethyl)phenyl]-5-isothiocyanato-3',5'-bis(trifluoromethyl)-1,1'-biphenyl (**S2**)

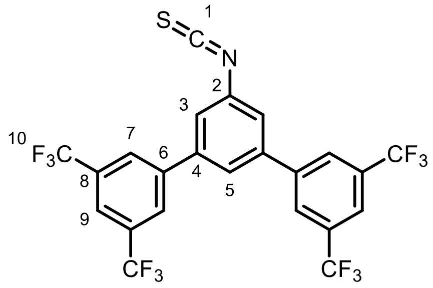

Product **S2** was prepared according to **Procedure 02**.

yield: 1.79 g (3.20 mmol, 89%, yellow solid).

^{1}H NMR (400 MHz, $CDCl_3$) δ 8.08 – 8.00 (m, 4H, ***C7***), 7.99 – 7.93 (m, 2H, ***C9***), 7.65 (t, *J* = 1.6 Hz, 1H, ***C5***), 7.52 (d, *J* = 1.6 Hz, 2H, ***C3***).

^{13}C NMR (101 MHz, $CDCl_3$) δ 141.4 (***C4*** or ***C6***), 141.4 (***C4*** or ***C6***), 138.4 (***C1***), 133.9 (***C2***), 132.8 (q, *J* = 33.6 Hz, ***C8***), 127.8 – 127.4 (m, ***C7***), 125.0 (***C5***), 124.8 (***C3***), 123.3 (q, *J* = 272.9 Hz, ***C10***), 122.4 (p, *J* = 3.6 Hz, ***C9***).

^{19}F NMR (376 MHz, $CDCl_3$) δ -62.8.

HRMS (ESI+, m/Z): ion not found.

m. p.: 142 – 144 °C.

FT-IR (thin film): ν_{max} (cm^{-1}) = 2131, 1376, 1193, 1131, 1121, 1006, 873, 704, 683.

[ToC]

1-[(2*R*)-3-Azido-1,1-diphenylpropan-2-yl]-3-{5-[3,5-bis(trifluoromethyl)phenyl]-3',5'-bis(trifluoromethyl)-[1,1'-biphenyl]-3-yl}thiourea (**S5**)

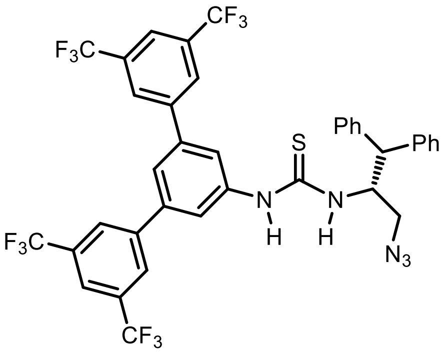

Product **S5** was prepared according to **Procedure 03**.

yield: 557 mg (0.69 mmol, 69%, colorless solid).

^{1}H NMR (400 MHz, $CDCl_3$) δ 8.00 – 7.96 (m, 2H), 7.94 – 7.88 (m, 5H), 7.59 (t, *J* = 1.6 Hz, 1H), 7.30 – 7.23 (m, 4H), 7.22 – 7.16 (m, 3H), 7.05 (d, *J* = 1.6 Hz, 2H), 6.98 – 6.92 (m, 2H), 6.90 – 6.85 (m, 1H), 5.74 (d, *J* = 8.1 Hz, 1H), 5.45 (ddt, *J* = 11.0, 8.2, 2.8 Hz, 1H), 4.12 (d, *J* = 11.5 Hz, 1H), 4.01 (dd, *J* = 12.5, 3.3 Hz, 1H), 3.18 (dd, *J* = 12.6, 2.1 Hz, 1H).

^{13}C NMR (101 MHz, $CDCl_3$) δ 180.3, 141.9, 141.5, 140.6, 140.4, 137.4, 132.7 (q, *J* = 33.5 Hz), 129.3, 128.7, 128.1, 127.9, 127.8 – 127.6 (m), 127.6, 127.1, 125.8, 124.9, 123.3 (q, *J* = 273.0 Hz), 122.5 – 122.2 (m), 57.4, 52.9, 51.8.

^{19}F NMR (376 MHz, $CDCl_3$) δ -62.7.

HRMS (ESI+, m/Z): exact mass calculated for $C_{38}H_{26}F_{12}N_5S$ $[M+H]^+$ 812.1712, found 812.1706.

m. p.: 100 – 102 °C.

$[\alpha]_D^{25}$ = -69.6 (c = 0.96, $CHCl_3$).

FT-IR (thin film): ν_{max} (cm^{-1}) = 2104, 1599, 1526, 1396, 1368, 1279, 1220, 1179, 1136, 903, 845, 757, 705, 683.

1-[(2*R*)-3-Azido-1,1-diphenylpropan-2-yl]-3-[3,5-bis(trifluoromethyl)phenyl]thiourea (**S6**)

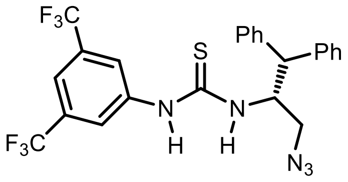

Product **S6** was prepared according to **Procedure 03**.

yield: 425 mg (0.81 mmol, 81%, colorless solid).

^{1}H NMR (400 MHz, $CDCl_3$) δ 8.10 (s, 1H), 7.71 (s, 1H), 7.37 – 7.27 (m, 10H), 7.25 – 7.18 (m, 2H), 5.92 (d, *J* = 8.4 Hz, 1H), 5.50 (s, 1H), 4.20 (d, *J* = 11.4 Hz, 1H), 3.91 (dd, *J* = 12.5, 3.4 Hz, 1H), 3.27 (dd, *J* = 12.5, 2.3 Hz, 1H).

^{13}C NMR (101 MHz, $CDCl_3$) δ 180.0, 140.6, 140.1, 137.8, 133.4 (q, *J* = 33.9 Hz), 129.2, 129.1, 128.0, 127.8, 127.5, 127.3, 124.3, 122.6 (q, *J* = 273.0 Hz) 120.1, 57.1, 52.9, 51.8.

^{19}F NMR (376 MHz, $CDCl_3$) δ -63.0.

Analytical data were consistent with those reported in the literature.[2]

[ToC]

Tris(3,5-di-*tert*-butylphenyl)phosphane (**S7**)

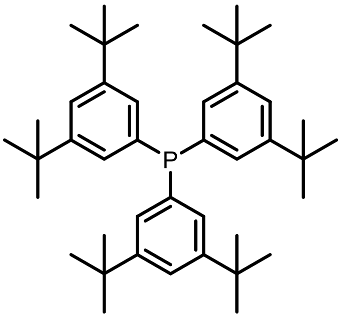

Product **S7** was prepared according to **Procedure 04**.

yield: 3.57 g (5.97 mmol, 81%, colorless solid).

^{1}H NMR (400 MHz, $CDCl_3$) δ 7.35 (td, *J* = 1.8, 0.5 Hz, 3H), 7.08 (dd, J = 8.1, 1.9 Hz, 6H), 1.22 (s, 54H).

^{13}C NMR (101 MHz, $CDCl_3$) δ 150.5 (d, *J* = 6.5 Hz), 137.0 (d, *J* = 8.8 Hz), 128.1 (d, *J* = 19.3 Hz), 122.4, 35.0, 31.5.

^{31}P NMR (162 MHz, $CDCl_3$) δ -3.3.

Analytical data were consistent with those reported in the literature.[3]

[ToC]

α,β-Unsaturated Esters and Intermediates

Benzyl (2*E*)-but-2-enoate (**1c**)

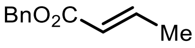

Product **1c** was prepared according to **Procedure 05**.

yield: 3.19 g (18.1 mmol, 91%, colorless oil).

^{1}H NMR (400 MHz, $CDCl_3$) δ 7.4 – 7.3 (m, 5H), 7.0 (dq, *J* = 15.5, 6.9 Hz, 1H), 5.9 (dq, *J* = 15.5, 1.7 Hz, 1H), 5.2 (s, 2H), 1.9 (dd, *J* = 6.9, 1.7 Hz, 3H).

^{13}C NMR (101 MHz, $CDCl_3$) δ 166.4, 145.3, 136.4, 128.7, 128.3 (d, *J* = 1.5 Hz), 122.7, 66.1, 18.1.

Analytical data were consistent with those reported in the literature.[7]

Methyl (2*E*)-3-cyclohexylprop-2-enoate (**1i**)

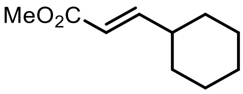

Product **1i** was prepared according to **Procedure 08**, and was obtained as a colorless oil after vacuum distillation.

yield: 404 mg (2.40 mmol, 14%).

^{1}H NMR (400 MHz, $CDCl_3$) δ 6.91 (dd, *J* = 15.8, 6.8 Hz, 1H), 5.75 (dd, *J* = 15.8, 1.5 Hz, 1H), 3.71 (s, 3H), 2.19 – 2.05 (m, 1H), 1.79 – 1.62 (m, 4H), 1.37 – 1.05 (m, 6H).

^{13}C NMR (101 MHz, $CDCl_3$) δ 167.7, 154.7, 118.6, 51.5, 40.5, 31.8, 26.0, 25.8.

Analytical data were consistent with those reported in the literature.[8]

Methyl (2*E*)-3-(naphthalen-2-yl)prop-2-enoate (**1m**)

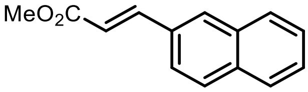

Following the work-up described in **Procedure 08**, the crude product was diluted with Et_2O (25 mL) and was cooled to 0 °C. Sat. aq. $NaHSO_4$ (50 mL) was then added. The mixture was stirred vigorously at 0 °C for 4 hours. The white slurry formed was filtered under reduced pressure and was washed with ice-cold Et_2O (25 mL). The organic layer in the filtrate was separated and the remaining aqueous layer was extracted with Et_2O (2 x 25 mL). The combined organic layers were washed with brine (15 mL), dried over Na_2SO_4 and filtered. Removal of the solvent under reduced pressure yielded product **1m** as a yellow solid.

yield: 519 mg (2.45 mmol, 24%).

^{1}H NMR (400 MHz, $CDCl_3$) δ 7.94 (s, 1H), 7.90 – 7.81 (m, 4H), 7.67 (dd, *J* = 8.6, 1.8 Hz, 1H), 7.55 – 7.48 (m, 2H), 6.56 (d, *J* = 16.0 Hz, 1H), 3.84 (s, 3H).

^{13}C NMR (101 MHz, $CDCl_3$) δ 167.6, 145.1, 134.4, 133.4, 132.0, 130.1, 128.8, 128.7, 127.9, 127.4, 126.9, 123.6, 118.1, 51.9.

Analytical data were consistent with those reported in the literature.[9]

[ToC]

Methyl (2*E*)-3-(4-methylphenyl)prop-2-enoate (**1n**)

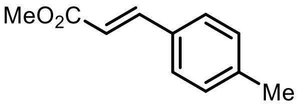

Product **1n** was prepared according to **Procedure 07**, and was obtained as a colorless solid after FCC (pentane : EtOAc 10%).

yield: 456 mg (2.59 mmol, 86%).

^{1}H NMR (400 MHz, $CDCl_3$) δ 7.67 (d, *J* = 16.0 Hz, 1H), 7.42 (d, *J* = 8.2 Hz, 2H), 7.19 (d, *J* = 7.8 Hz, 2H), 6.40 (d, *J* = 16.0 Hz, 1H), 3.80 (s, 3H), 2.37 (s, 3H).

^{13}C NMR (101 MHz, $CDCl_3$) δ 167.8, 145.0, 140.9, 131.8, 129.8, 128.2, 116.9, 51.8, 21.6.

Analytical data were consistent with those reported in the literature.[10]

Ethyl (2*E*)-3-(4-fluorophenyl)prop-2-enoate (**1p**)

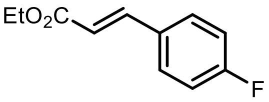

Product **1p** was prepared according to **Procedure 09**.

yield: 1.92 g (9.90 mmol, 99%, colorless solid).

^{1}H NMR (400 MHz, $CDCl_3$) δ 7.6 (d, *J* = 16.0 Hz, 1H), 7.5 – 7.5 (m, 2H), 7.1 – 7.0 (m, 2H), 6.4 (dd, *J* = 16.0, 0.6 Hz, 1H), 4.3 (q, *J* = 7.1 Hz, 2H), 1.3 (t, *J* = 7.1 Hz, 3H).

^{13}C NMR (101 MHz, $CDCl_3$) δ 167.0, 164.0 (d, *J* = 251.1 Hz), 143.4, 130.9 (d, *J* = 3.5 Hz), 130.0 (d, *J* = 8.4 Hz), 118.2 (d, *J* = 2.4 Hz), 116.1 (d, *J* = 21.9 Hz), 60.7, 14.4.

^{19}F NMR (376 MHz, $CDCl_3$) δ -109.8 (tt, *J* = 8.5, 5.4 Hz).

Analytical data were consistent with those reported in the literature.[11]

Octyl (2*E*)-3-(4-nitrophenyl)prop-2-enoate (**1s**)

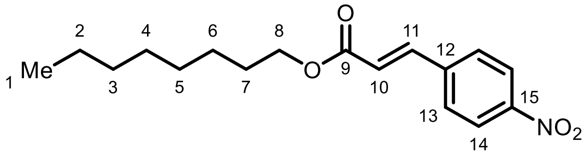

Product **1s** was prepared according to **Procedure 13**.

yield: 550 mg (1.80 mmol, 18%, colorless solid).

^{1}H NMR (400 MHz, $CDCl_3$) δ 8.24 (d, *J* = 8.4 Hz, 2H, ***C14***), 7.80 – 7.58 (m, 3H, ***C11***, ***C13***), 6.56 (d, *J* = 16.0 Hz, 1H, ***C10***), 4.22 (t, *J* = 6.7 Hz, 2H, ***C8***), 1.71 (p, *J* = 6.9 Hz, 2H, ***C7***), 1.51 – 1.18 (m, 10H, ***C2*** – ***C6***), 0.88 (t, *J* = 6.4 Hz, 3H, ***C1***).

^{13}C NMR (101 MHz, $CDCl_3$) δ 166.2 (***C9***), 148.6 (***C15***), 141.7 (***C11***), 140.8 (***C12***), 128.7 (***C13***), 124.3 (***C14***), 122.8 (***C10***), 65.4 (***C8***), 31.9 (***C2*** – ***C6***), 29.4 (***C2*** – ***C6***), 29.3 (***C2*** – ***C6***), 28.8 (***C7***), 26.1 (***C2*** – ***C6***), 22.8 (***C2*** – ***C6***), 14.2 (***C1***).

HRMS (ESI+, m/Z): exact mass calculated for $C_{17}H_{24}NO_4$ $[M+H]^+$ 306.1700, found 306.1694.

m. p.: 28 – 30 °C.

FT-IR (thin film): ν_{max} (cm^{-1}) = 2927, 1708, 1639, 1594, 1516, 1340, 1278, 1174, 832, 760.

[ToC]

Methyl (2*E*)-3-(5-bromo-2-methoxyphenyl)prop-2-enoate (**1v**)

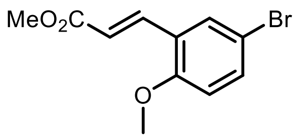

Product **1v** was prepared according to **Procedure 07**, and was obtained as a colorless solid after FCC (pentane : EtOAc 10%).

yield: 592 mg (2.18 mmol, 73%).

^{1}H NMR (400 MHz, $CDCl_3$) δ 7.89 (d, *J* = 16.2 Hz, 1H), 7.59 (d, *J* = 2.5 Hz, 1H), 7.42 (dd, *J* = 8.8, 2.5 Hz, 1H), 6.79 (d, *J* = 8.8 Hz, 1H), 6.49 (d, *J* = 16.2 Hz, 1H), 3.86 (s, 3H), 3.80 (s, 3H).

^{13}C NMR (101 MHz, $CDCl_3$) δ 167.6, 157.4, 138.8, 133.9, 131.3, 125.5, 119.7, 113.1, 113.0, 55.9, 51.8.

Analytical data were consistent with those reported in the literature.[12]

Methyl (2*E*)-3-[3-(cyclopentyloxy)-4-methoxyphenyl]prop-2-enoate (**1x**)

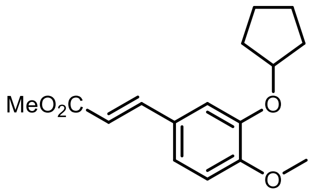

Product **1x** was prepared according to **Procedure 11**.

yield: 2.54 g (9.21 mmol, 92%, colorless solid).

^{1}H NMR (400 MHz, $CDCl_3$) δ 7.62 (d, *J* = 15.9 Hz, 1H), 7.08 (dd, *J* = 8.3, 2.0 Hz, 1H), 7.05 (d, *J* = 2.0 Hz, 1H), 6.85 (d, *J* = 8.3 Hz, 1H), 6.28 (d, *J* = 15.9 Hz, 1H), 4.79 (tt, *J* = 6.4, 3.2 Hz, 1H), 3.87 (s, 3H), 3.79 (s, 3H), 2.01 – 1.78 (m, 6H), 1.68 – 1.57 (m, 2H).

^{13}C NMR (101 MHz, $CDCl_3$) δ 167.8, 152.3, 148.0, 145.1, 127.4, 122.4, 115.4, 113.5, 111.7, 80.7, 56.2, 51.7, 32.9, 24.2.

Analytical data were consistent with those reported in the literature.[13]

Methyl (2*E*)-3-(pyridin-2-yl)prop-2-enoate (**1y**)

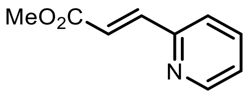

Product **1y** was prepared according to **Procedure 07**, and was obtained as a yellow solid after FCC (pentane : EtOAc 5% to 10%).

yield: 254 mg (1.56 mmol, 52%).

^{1}H NMR (400 MHz, $CDCl_3$) δ 8.69 – 8.60 (m, 1H), 7.75 – 7.64 (m, 2H), 7.44 (d, *J* = 7.8 Hz, 1H), 7.34 – 7.21 (m, 1H), 6.95 (d, *J* = 15.7 Hz, 1H), 3.84 (s, 3H).

^{13}C NMR (101 MHz, $CDCl_3$) δ 167.3, 153.0, 150.3, 143.7, 136.9, 124.4, 124.3, 122.1, 52.0.

Analytical data were consistent with those reported in the literature.[8]

Methyl (2*E*)-3-(pyridin-3-yl)prop-2-enoate (**1z**)

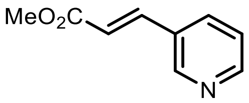

Product **1z** was prepared according to **Procedure 07**, and was obtained as a yellow solid after FCC (pentane : EtOAc 40%).

yield: 289 mg (1.77 mmol, 59%).

^{1}H NMR (400 MHz, $CDCl_3$) δ 8.65 (d, *J* = 2.2 Hz, 1H), 8.51 (dd, *J* = 4.8, 1.7 Hz, 1H), 7.77 – 7.73 (m, 1H), 7.59 (d, *J* = 16.3 Hz, 1H), 7.24 (ddt, *J* = 8.0, 4.9, 0.6 Hz, 1H), 6.43 (d, *J* = 16.1 Hz, 1H), 3.73 (s, 3H).

^{13}C NMR (101 MHz, $CDCl_3$) δ 166.6, 151.0, 149.7, 141.1, 134.1, 130.1, 123.7, 119.9, 51.8.

Analytical data were consistent with those reported in the literature.[14]

Methyl (2*E*)-3-(pyridin-3-yl)prop-2-enoate (**1aa**)

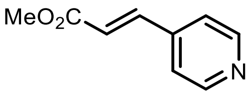

Product **1aa** was prepared according to **Procedure 06**, and was obtained as a pink solid after FCC (pentane : EtOAc 30%).

yield: 234 mg (1.41 mmol, 21%).

^{1}H NMR (400 MHz, $CDCl_3$) δ 8.65 – 8.62 (m, 2H), 7.59 (d, *J* = 16.0 Hz, 1H), 7.37 – 7.32 (m, 2H), 6.58 (d, *J* = 16.1 Hz, 1H), 3.81 (s, 3H).

^{13}C NMR (101 MHz, $CDCl_3$) δ 166.5, 150.7, 142.1, 141.6, 122.5, 121.9, 52.1.

Analytical data were consistent with those reported in the literature.[8]

Methyl (2*E*)-3-(quinolin-3-yl)prop-2-enoate (**1ab**)

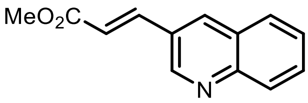

Product **1ab** was prepared according to **Procedure 14**.

yield: 178 mg (2.87 mmol, 84%, yellow solid).

^{1}H NMR (400 MHz, $CDCl_3$) δ 9.09 (d, *J* = 2.3 Hz, 1H), 8.24 (d, *J* = 2.2 Hz, 1H), 8.11 (d, *J* = 8.4 Hz, 1H), 7.88 – 7.82 (m, 2H), 7.80 – 7.72 (m, 1H), 7.59 (t, *J* = 8.2 Hz, 1H), 6.67 (d, *J* = 16.1 Hz, 1H), 3.85 (s, 3H).

^{13}C NMR (101 MHz, $CDCl_3$) δ 167.0, 149.4, 148.7, 141.6, 135.6, 130.8, 129.6, 128.5, 127.7, 127.6, 127.5, 119.9, 52.1.

Analytical data were consistent with those reported in the literature.[15]

Methyl (2*E*)-3-[2-(methylsulfanyl)pyrimidin-5-yl]prop-2-enoate (**1ac**)

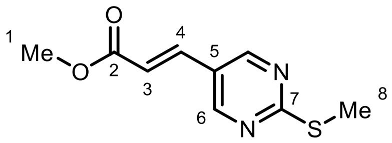

Product **1ac** was prepared according to **Procedure 06**, and was obtained as a colorless solid after FCC (pentane : EtOAc 30%).

yield: 344 mg (1.64 mmol, 82%).

^{1}H NMR (400 MHz, $CDCl_3$) δ 8.64 (s, 2H, ***C6***), 7.55 (d, *J* = 16.3, Hz, 1H, ***C4***), 6.49 (d, *J* = 16.2 Hz, 1H, ***C3***), 3.82 (s, 3H, ***C1***), 2.59 (s, 3H, ***C8***).

^{13}C NMR (101 MHz, $CDCl_3$) δ 174.5 (***C7***), 166.6 (***C2***), 156.0 (***C6***), 137.8 (***C4***), 123.3 (***C5***), 119.6 (***C3***), 52.1 (***C1***), 14.4 (***C8***).

HRMS (ESI+, m/Z): exact mass calculated for $C_9H_{11}N_2O_2S$ $[M+H]^+$ 211.0536, found 211.0536.

m. p.: 102 – 104 °C.

FT-IR (thin film): ν_{max} (cm^{-1}) = 1711, 1640, 1581, 1530, 1432, 1413, 1386, 1323, 1197, 1172, 981, 859, 772, 720.

Methyl (2*E*)-3-(furan-2-yl)prop-2-enoate (**1ad**)

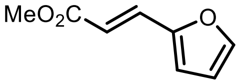

Product **1ad** was prepared according to **Procedure 12**.

yield: 1.85 g (12.2 mmol, 61%, yellow solid).

^{1}H NMR (400 MHz, $CDCl_3$) δ 7.50 – 7.38 (m, 2H), 6.60 (d, *J* = 3.4 Hz, 1H), 6.51 – 6.42 (m, 1H), 6.31 (d, *J* = 15.7 Hz, 1H), 3.78 (s, 3H).

^{13}C NMR (101 MHz, $CDCl_3$) δ 167.6, 151.0, 144.9, 131.3, 115.6, 114.9, 112.4, 51.8.

Analytical data were consistent with those reported in the literature.[16]

Ethyl (2*E*)-3-(1,3,4-oxadiazol-2-yl)prop-2-enoate (**1ae**)

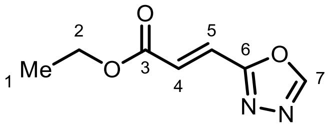

Product **1ae** was prepared according to **Procedure 15**.

yield: 180 mg (1.07 mmol, 21%, yellow solid).

^{1}H NMR (400 MHz, $CDCl_3$) δ 8.48 – 8.43 (m, 1H, ***C7***), 7.59 (dd, *J* = 16.1, 0.8 Hz, 1H, ***C5***), 6.85 (d, *J* = 16.1 Hz, 1H, ***C4***), 4.30 (q, *J* = 7.1 Hz, 2H, ***C2***), 1.34 (t, *J* = 7.1 Hz, 3H, ***C1***).

^{13}C NMR (101 MHz, $CDCl_3$) δ 164.7 (***C3***), 162.6 (***C6***), 153.2 (***C7***), 129.2 (***C4***), 124.6 (***C5***), 61.7 (***C2***), 14.3 (***C1***).

HRMS (ESI+, m/Z): exact mass calculated for $C_7H_9N_2O_3$ $[M+H]^+$ 169.0608, found 169.0606.

m. p.: 46 – 48 °C.

FT-IR (thin film): ν_{max} (cm^{-1}) = 3124, 1712, 1527, 1368, 1308, 1268, 1187, 1095, 980, 957, 752, 690.

[ToC]

Methyl 6-nitrohexanoate (**S8**)

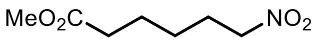

Product **S8** was prepared according to **Procedure 16**.

yield: 696 mg (4.00 mmol, 40%, colorless oil).

^{1}H NMR (400 MHz, $CDCl_3$) δ 4.39 (t, *J* = 7.0 Hz, 2H), 3.67 (s, 3H), 2.34 (t, *J* = 7.4 Hz, 2H), 2.09 – 1.97 (m, 2H), 1.80 – 1.62 (m, 2H), 1.49 – 1.36 (m, 2H).

^{13}C NMR (101 MHz, $CDCl_3$) δ 173.8, 75.5, 51.8, 33.7, 27.2, 25.9, 24.3.

Analytical data were consistent with those reported in the literature.[5b]

Ethyl (*E*)-8-nitrooct-2-enoate (**1ag**)

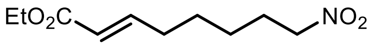

Product **1ag** was prepared according to **Procedure 17**.

yield: 570 mg (2.65 mmol, 67%, colorless oil).

^{1}H NMR (400 MHz, $CDCl_3$) δ 6.92 (dt, *J* = 15.6, 7.0 Hz, 1H), 5.82 (dt, *J* = 15.6, 1.6 Hz, 1H), 4.38 (t, *J* = 7.0 Hz, 2H), 4.18 (q, *J* = 7.1 Hz, 2H), 2.22 (qd, *J* = 7.1, 1.6 Hz, 2H), 2.08 – 1.96 (m, 2H), 1.59 – 1.35 (m, 4H), 1.29 (t, *J* = 7.1 Hz, 3H).

^{13}C NMR (101 MHz, $CDCl_3$) δ 166.7, 148.3, 122.0, 75.6, 60.4, 31.9, 27.4, 27.3, 25.9, 14.4.

Analytical data were consistent with those reported in the literature.[5b]

[ToC]

γ-Nitroesters

Methyl (3*R*)-3-methyl-4-nitrobutanoate (**3a**)

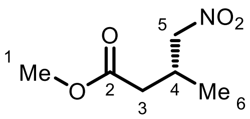

Product **3a** was prepared according to **Procedure 19**, and was obtained as a colorless oil after FCC (pentane : Et_2O 0% to 15%).

yield: 48.0 mg (0.298 mmol, 99%).

e.r.: 95:5 [HPLC CHIRALPAK® AS-H, hexane/IPA = 95/05, 1 mL/min, λ = 220 nm, $t_{(major)}$ = 16.1 min, $t_{(minor)}$ = 19.5 min].

^{1}H NMR (400 MHz, $CDCl_3$) δ 4.47 (dd, *J* = 12.1, 6.3 Hz, 1H, ***C5***), 4.34 (dd, *J* = 12.1, 7.1 Hz, 1H, ***C5***), 3.69 (s, 3H, ***C1***), 2.84 – 2.72 (m, 1H, ***C4***), 2.45 (dd, *J* = 16.2, 6.7 Hz, 1H, ***C3***), 2.36 (dd, *J* = 16.2, 6.9 Hz, 1H, ***C3***), 1.10 (d, *J* = 6.9 Hz, 3H, ***C6***).

^{13}C NMR (101 MHz, $CDCl_3$) δ 171.8 (***C2***), 80.3 (***C5***), 51.9 (***C1***), 37.8 (***C3***), 29.5 (***C4***), 17.4 (***C6***).

HRMS (ESI+, m/Z): exact mass calculated for $C_6H_{11}NNaO_4$ $[M+Na]^+$ 184.0580, found 184.0577.

$[\alpha]_D^{25}$ = -3.2 (*c* = 2.10, $CHCl_3$).

FT-IR (thin film): ν_{max} (cm^{-1}) = 1738, 1553, 1438, 1382, 1282, 1222, 1178, 1108, 1010, 892, 648.

Rthyl (3*R*)-3-methyl-4-nitrobutanoate (**3b**)

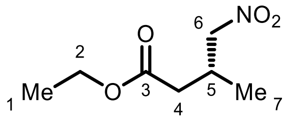

Product **3b** was prepared according to **Procedure 19**, and was obtained as a colorless oil after FCC (pentane : EtOAc 0% to 10%).

yield: 50.6 mg (0.289 mmol, 96%).

e.r.: 95.5:4.5 [HPLC CHIRALPAK® AS-H, hexane/IPA = 95/05, 1 mL/min, λ = 220 nm, $t_{(major)}$ = 10.3 min, $t_{(minor)}$ = 12.2 min].

^{1}H NMR (400 MHz, $CDCl_3$) δ 4.52 – 4.29 (m, 2H, ***C6***), 4.15 (q, *J* = 7.1 Hz, 2H, ***C2***), 2.86 – 2.70 (m, 1H, ***C5***), 2.48 – 2.29 (m, 2H, ***C4***), 1.26 (t, *J* = 7.1 Hz, 3H, ***C1***), 1.10 (d, *J* = 6.8 Hz, 3H, ***C7***).

^{13}C NMR (101 MHz, $CDCl_3$) δ 171.3 (***C3***), 80.4 (***C6***), 60.9 (***C2***), 38.1 (***C4***), 29.6 (***C5***), 17.4 (***C7***), 14.3 (***C1***).

HRMS (ESI+, m/Z): exact mass calculated for $C_7H_{13}NNaO_4$ $[M+Na]^+$ 198.0737, found 198.0738.

$[\alpha]_D^{25}$ = -2.1 (*c* = 4.17, $CHCl_3$).

FT-IR (thin film): ν_{max} (cm^{-1}) = 1731, 1552, 1377, 1181, 1030.

[ToC]

Benzyl (3*R*)-3-methyl-4-nitrobutanoate (**3c**)

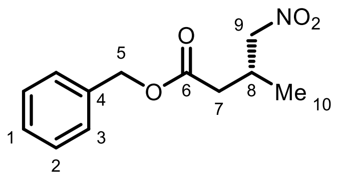

Product **3c** was prepared according to **Procedure 19**, and was obtained as a colorless oil after FCC (pentane : EtOAc 0% to 10%).

yield: 62.0 mg (0.262 mmol, 87%).

e.r.: 88.5:11.5 [HPLC CHIRALPAK® IA, hexane/IPA = 99/01, 1 mL/min, λ = 220 nm, $t_{(minor)}$ = 22.7 min, $t_{(major)}$ = 24.5 min].

^{1}H NMR (400 MHz, $CDCl_3$) δ 7.42 – 7.29 (m, 5H, ***C1*** – ***C3***), 5.14 (s, 2H, ***C5***), 4.46 (dd, *J* = 12.1, 6.2 Hz, 1H, ***C9***), 4.33 (dd, *J* = 12.1, 7.1 Hz, 1H, ***C9***), 2.89 – 2.72 (m, 1H, ***C8***), 2.54 – 2.37 (m, 2H, ***C7***), 1.09 (d, *J* = 6.8 Hz, 3H, ***C10***).

^{13}C NMR (101 MHz, $CDCl_3$) δ 171.1 (***C6***), 135.7 (***C4***), 128.7 (***C2***), 128.5 (***C1***), 128.4 (***C3***), 80.3 (***C9***), 66.7 (***C5***), 38.0 (***C7***), 29.6 (***C8***), 17.4 (***C10***).

HRMS (ESI+, m/Z): exact mass calculated for $C_{12}H_{16}NO_4$ $[M+H]^+$ 238.1074, found 238.1068.

$[\alpha]_D^{25}$ = +0.3 (*c* = 3.11, $CHCl_3$).

FT-IR (thin film): ν_{max} (cm^{-1}) = 1733, 1552, 1457, 1381, 1171, 1106, 999, 741, 698.

Methyl (3*R*)-3-methyl-4-nitrohexanoate (**3e**)

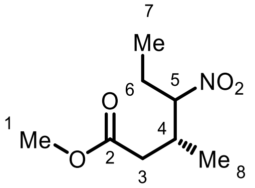

Product **3e** was prepared according to **Procedure 19**, and was obtained as a colorless oil after preparative TLC (toluene : EtOAc 10%).

yield: 35.9 mg (0.190 mmol, 63%).

d.r.: 55:45.

e.r.: 91.5:8.5 (major diastereomer); 89:11 (minor diastereomer) [HPLC CHIRALPAK® IA, hexane/IPA = 98/02, 1 mL/min, λ = 220 nm, *major diastereomer:* $t_{(minor)}$ = 7.8 min, $t_{(major)}$ = 8.7 min; *minor diastereomer:* $t_{(minor)}$ = 9.4 min, $t_{(major)}$ = 10.2 min].

^{1}H NMR (400 MHz, $CDCl_3$) δ 4.49 – 4.33 (m, 1H, ***C5***), 3.72 – 3.65 (m, 3H, ***C1***), 2.60 – 2.41 (m, 2H, ***C3***, ***C4***), 2.32 – 2.16 (m, 1H, ***C3***), 2.12 – 1.90 (m, 1H, ***C6***), 1.88 – 1.69 (m, 1H, ***C6***), 1.08 – 0.92 (m, 6H, ***C7***, ***C8***).

^{13}C NMR (101 MHz, $CDCl_3$) δ 172.2 (***C2***), 94.2 (***C5***), 93.6 (***C5***), 52.0 (***C1***), 51.9 (***C1***), 37.6 (***C3***), 37.2 (***C3***), 33.7 (***C4***), 33.5 (***C4***), 24.3 (***C6***), 24.2 (***C6***), 16.4 (***C8***), 15.6 (***C8***), 10.7 (***C7***), 10.5 (***C7***).

HRMS (ESI+, m/Z): exact mass calculated for $C_8H_{15}NNaO_4$ $[M+Na]^+$ 212.0893, found 212.0893.

$[\alpha]_D^{25}$ = +2.1 (*c* = 1.74, $CHCl_3$).

FT-IR (thin film): ν_{max} (cm^{-1}) = 1738, 1548, 1450, 1438, 1372, 1263, 1197, 1179, 1005, 809.

[ToC]

Methyl (3*R*)-3-(nitromethyl)pentanoate (3f)

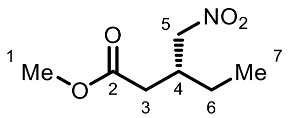

Product **3f** was prepared according to **Procedure 19**, and was obtained as a colorless oil after FCC (Biotage ZIP® 10 g cartridge, [pentane : CH_2Cl_2 25%] : EtOAc 0% to 5%).

yield: 41.6 mg (0.238 mmol, 79%).

e.r.: 95:5 [SFC CHIRALPAK® IG, from 1% to 20% MeOH in 7 min, then from 20% to 50% in 1 min, 1 mL/min, λ = 220 nm, $t_{(major)}$ = 2.7 min, $t_{(minor)}$ = 2.8 min].

^{1}H NMR (400 MHz, $CDCl_3$) δ 4.48 (qd, *J* = 12.3, 6.3 Hz, 2H, ***C5***), 3.70 (s, 3H, ***C1***), 2.57 (hept, *J* = 6.5 Hz, 1H, ***C4***), 2.49 – 2.42 (m, 2H, ***C3***), 1.56 – 1.40 (m, 2H, ***C6***), 0.98 (t, *J* = 7.5 Hz, 3H, ***C7***).

^{13}C NMR (101 MHz, $CDCl_3$) δ 172.1 (***C2***), 78.3 (***C5***), 52.0 (***C1***), 35.8 (***C4***), 35.4 (***C3***), 24.5 (***C6***), 11.0 (***C7***).

HRMS (ESI+, m/Z): exact mass calculated for $C_7H_{13}NNaO_4$ $[M+Na]^+$ 198.0737, found 198.0738.

$[\alpha]_D^{25}$ = -5.4 (*c* = 0.73, $CHCl_3$).

FT-IR (thin film): ν_{max} (cm^{-1}) = 1736, 1552, 1437, 1380, 1175, 1017.

Ethyl (3*R*)-3-(nitromethyl)hexanoate (3g)

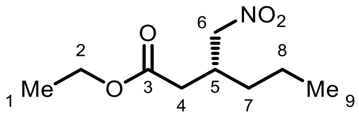

Product **3g** was prepared according to **Procedure 19**, and was obtained as a colorless oil after FCC (pentane : EtOAc 0% to 10%).

yield: 30.9 mg (0.163 mmol, 54%).

e.r.: 95:5 [HPLC CHIRALPAK® IA, hexane/IPA = 99/01, 1 mL/min, λ = 222 nm, $t_{(major)}$ = 11.3 min, $t_{(minor)}$ = 12.2 min].

^{1}H NMR (400 MHz, $CDCl_3$) δ 4.53 – 4.39 (m, 2H, ***C6***), 4.14 (q, *J* = 7.1 Hz, 2H, ***C2***), 2.62 (p, *J* = 6.4 Hz, 1H, ***C5***), 2.41 (d, 2H, ***C4***), 1.43 – 1.32 (m, 4H, ***C7***, ***C8***), 1.25 (t, *J* = 7.1 Hz, 3H, ***C1***), 0.95 – 0.87 (m, 3H, ***C9***).

^{13}C NMR (101 MHz, $CDCl_3$) δ 171.6 (***C3***), 78.7 (***C6***), 60.8 (***C2***), 36.0 (***C4***), 34.1 (***C5***), 33.7 (***C7***), 19.7 (***C8***), 14.3 (***C1***), 14.0 (***C9***).

HRMS (ESI+, m/Z): ion not found.

$[\alpha]_D^{25}$ = -19.6 (*c* = 0.38, $CHCl_3$).

FT-IR (thin film): ν_{max} (cm^{-1}) = 1733, 1553, 1467, 1369, 1280, 1181, 1138, 1031, 706.

[ToC]

Methyl (3*R*)-5-methyl-3-(nitromethyl)hexanoate (**3h**)

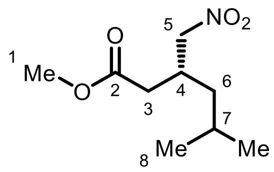

Product **3h** was prepared according to **Procedure 19**, and was obtained as a colorless oil after FCC (pentane : EtOAc 0% to 8%).

yield: 39.3 mg (0.193 mmol, 64%).

e.r.: 89.5:10.5 [HPLC CHIRALPAK® AS-H, hexane/IPA = 90/10, 1 mL/min, λ = 220 nm, $t_{(minor)}$ = 7.2 min, $t_{(major)}$ = 8.2 min].

^{1}H NMR (400 MHz, $CDCl_3$) δ 4.55 – 4.39 (m, 2H), 3.70 (s, 3H), 2.74 – 2.61 (m, 1H), 2.45 (d, *J* = 6.4 Hz, 2H), 1.64 (dh, *J* = 13.4, 6.7 Hz, 1H), 1.32 – 1.21 (m, 2H), 0.97 – 0.87 (m, 6H).

^{13}C NMR (101 MHz, $CDCl_3$) δ 172.1 (***C2***), 78.9 (***C5***), 51.9 (***C1***), 40.7 (***C6***), 35.9 (***C3***), 32.3 (***C4***), 25.2 (***C7***), 22.7 (***C8***), 22.4 (***C8***).

HRMS (ESI+, m/Z): exact mass calculated for $C_9H_{17}NNaO_4$ $[M+Na]^+$ 226.1050, found 226.1050.

$[\alpha]_D^{25}$ = -7.2 (*c* = 1.31, $CHCl_3$).

FT-IR (thin film): ν_{max} (cm^{-1}) = 2958, 1737, 1552, 1437, 1383, 1175, 1005.

Methyl (3*S*)-3-cyclohexyl-4-nitrobutanoate (**3i**)

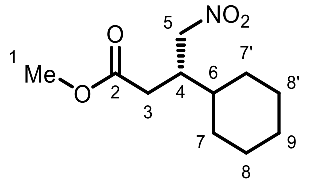

Product **3i** was prepared according to **Procedure 19**, and was obtained as a colorless oil after FCC (pentane : EtOAc 0% to 6%).

yield: 30.9 mg (0.135 mmol, 45%).

e.r.: 92.5:7.5 [HPLC CHIRALPAK® AD-H, hexane/IPA = 92/08, 1 mL/min, λ = 220 nm, $t_{(major)}$ = 5.9 min, $t_{(minor)}$ = 6.8 min].

^{1}H NMR (400 MHz, $CDCl_3$) δ 4.48 (d, *J* = 6.5 Hz, 2H, ***C5***), 3.69 (s, 3H, ***C1***), 2.64 – 2.46 (m, 2H, ***C3***, ***C4***), 2.37 (dd, *J* = 16.2, 7.7 Hz, 1H, ***C3***), 1.82 – 1.62 (m, 5H, ***C7*** – ***C9***), 1.51 – 1.37 (m, 1H, ***C6***), 1.31 – 0.91 (m, 5H, ***C7*** – ***C9***).

^{13}C NMR (101 MHz, $CDCl_3$) δ 172.5 (***C2***), 77.2 (***C5***), 52.0 (***C1***), 39.5 (***C4***), 39.1 (***C6***), 33.5 (***C3***), 30.0 (***C7***), 29.5 (***C7'***), 26.4 (***C9***), 26.4 (***C8***), 26.3 (***C8'***).

HRMS (ESI+, m/Z): exact mass calculated for $C_{11}H_{19}NNaO_4$ $[M+Na]^+$ 252.1206, found 252.1205.

$[\alpha]_D^{25}$ = -6.2 (*c* = 1.72, $CHCl_3$).

FT-IR (thin film): ν_{max} (cm^{-1}) = 2927, 2854, 1736, 1552, 1437, 1378, 1172.

[ToC]

Ethyl (3*R*)-4,4,4-trifluoro-3-(nitromethyl)butanoate (**3j**)

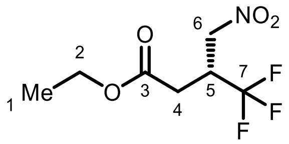

Product **3j** was prepared according to **Procedure 19**, and was obtained as a colorless oil after FCC (pentane : EtOAc 0% to 10%).

yield: 49.2 mg (0.210 mmol, 72%).

e.r.: 94:6 [HPLC CHIRALPAK® IA, hexane/IPA = 98/02, 1 mL/min, λ = 220 nm, $t_{(minor)}$ = 13.0 min, $t_{(major)}$ = 14.2 min].

^{1}H NMR (400 MHz, $CDCl_3$) δ 4.65 (qd, *J* = 14.1, 6.0 Hz, 2H, ***C6***), 4.20 (q, *J* = 7.1 Hz, 2H, ***C2***), 3.75 – 3.58 (m, 1H, ***C5***), 2.85 – 2.75 (m, 1H, ***C4***), 2.65 – 2.53 (m, 1H, ***C4***), 1.28 (t, *J* = 7.1 Hz, 3H, ***C1***).

^{13}C NMR (101 MHz, $CDCl_3$) δ 169.3 (***C3***), 125.7 (q, *J* = 280.0 Hz, ***C7***), 72.4 (q, *J* = 2.8 Hz, ***C6***), 61.9 (***C2***), 39.1 (q, *J* = 28.7 Hz, ***C5***), 30.8 (q, *J* = 2.4 Hz, ***C4***), 14.2 (***C1***).

^{19}F NMR (377 MHz, $CDCl_3$) δ -71.3 (d, *J* = 8.5 Hz).

HRMS (ESI+, m/Z): exact mass calculated for $C_7H_{10}F_3NNaO_4$ $[M+Na]^+$ 262.1074, found 262.1074.

$[\alpha]_D^{25}$ = -3.9 (*c* = 2.21, $CHCl_3$).

FT-IR (thin film): ν_{max} (cm^{-1}) = 2989, 1736, 1567, 1379, 1256, 1221, 1175, 1126, 1025.

Ethyl (3*E*,5*R*)-5-methyl-6-nitrohex-3-enoate (**3k**)

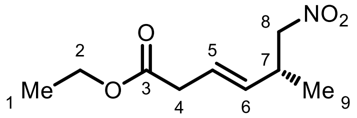

Product **3k** was prepared according to **Procedure 19**, and was obtained as a colorless oil after FCC (Biotage ZIP® 10 g cartridge, [pentane : CH_2Cl_2 15%] : EtOAc 0% to 8%).

yield: 19.2 mg (0.096 mmol, 32%).

e.r.: 95.5:4.5 [HPLC CHIRALPAK® AS-H, hexane/IPA = 95/05, 1 mL/min, λ = 220 nm, $t_{(major)}$ = 12.7 min, $t_{(minor)}$ = 14.0 min].

^{1}H NMR (400 MHz, $CDCl_3$) δ 5.69 (dtd, *J* = 15.2, 6.9, 1.1 Hz, 1H, ***C5***), 5.45 (ddt, *J* = 15.4, 7.6, 1.5 Hz, 1H, ***C6***), 4.35 – 4.21 (m, 2H, ***C8***), 4.13 (q, *J* = 7.1 Hz, 2H, ***C2***), 3.11 – 2.96 (m, 3H, ***C4***, ***C7***), 1.25 (t, *J* = 7.1 Hz, 3H, ***C1***), 1.12 (d, *J* = 6.8 Hz, 3H, ***C9***).

^{13}C NMR (101 MHz, $CDCl_3$) δ 171.4 (***C3***), 133.2 (***C6***), 124.6 (***C5***), 80.8 (***C8***), 60.8 (***C2***), 37.8 (***C4***), 35.9 (***C7***), 17.3 (***C9***), 14.2 (***C1***).

HRMS (ESI+, m/Z): exact mass calculated for $C_9H_{16}NO_4$ $[M+H]^+$, 202.1074 found 202.1070.

$[\alpha]_D^{25}$ = +34.5 (*c* = 1.29, $CHCl_3$).

FT-IR (thin film): ν_{max} (cm^{-1}) = 2983, 1734, 1552, 1383, 1324, 1176, 1027, 974.

[ToC]

Methyl (3*S*)-4-nitro-3-phenylbutanoate (**3l**)

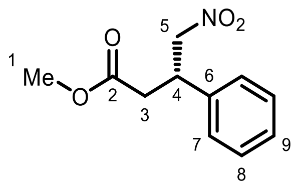

Product **3l** was prepared according to **Procedure 19**, and was obtained as a colorless oil after preparative TLC ($CHCl_3$).

yield: 49.8 mg (0.223 mmol, 74%).

e.r.: 96.5:3.5 [HPLC CHIRALPAK® AS-H, hexane/IPA = 95/05, 1 mL/min, λ = 220 nm, $t_{(major)}$ = 28.3 min, $t_{(minor)}$ = 35.0 min].

^{1}H NMR (400 MHz, $CDCl_3$) δ 7.38 – 7.18 (m, 5H, ***C7*** – ***C9***), 4.74 (dd, *J* = 12.6, 7.0 Hz, 1H, ***C5***), 4.64 (dd, *J* = 12.6, 7.9 Hz, 1H, ***C5***), 3.99 (p, *J* = 7.4 Hz, 1H, ***C4***), 3.63 (s, 3H, ***C1***), 2.78 (dd, *J* = 7.4, 1.0 Hz, 2H, ***C3***).

^{13}C NMR (101 MHz, $CDCl_3$) δ 171.2 (***C2***), 138.4 (***C6***), 129.2 (***C8***), 128.2 (***C9***), 127.4 (***C7***), 79.5 (***C5***), 52.0 (***C1***), 40.3 (***C3***), 37.6 (***C3***).

HRMS (ESI+, m/Z): exact mass calculated for $C_{11}H_{13}NNaO_4$ $[M+Na]^+$ 246.0737, found 246.0737.

$[\alpha]_D^{25}$ = -10.6 (*c* = 1.19, $CHCl_3$). {lit.: $[\alpha]_D^{25}$ = +8.7 (*c* = 2.00, $CHCl_3$, (*R*)-enantiomer)}[17]

FT-IR (thin film): ν_{max} (cm^{-1}) = 2955, 1734, 1552, 1437, 1378, 1199, 1171, 767, 701.

Methyl (3*S*)-3-(naphthalen-2-yl)-4-nitrobutanoate (**3m**)

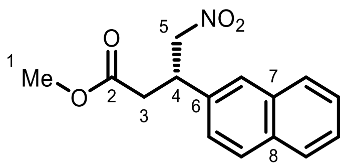

Product **3m** was prepared according to **Procedure 19**, and was obtained as a colorless oil after FCC (pentane : EtOAc 0% to 20%).

yield: 73.2 mg (0.267 mmol, 89%).

e.r.: 97:3 [HPLC CHIRALPAK® AS-H, hexane/IPA = 85/15, 1 mL/min, λ = 240 nm, $t_{(major)}$ = 17.4 min, $t_{(minor)}$ = 22.7 min].

^{1}H NMR (400 MHz, $CDCl_3$) δ 7.87 – 7.77 (m, 3H, ***ArH***), 7.72 – 7.67 (m, 1H, ***ArH***), 7.54 – 7.44 (m, 2H, ***ArH***), 7.34 (dd, *J* = 8.5, 1.9 Hz, 1H, ***ArH***), 4.82 (dd, *J* = 12.7, 7.1 Hz, 1H, ***C5***), 4.74 (dd, *J* = 12.7, 7.8 Hz, 1H, ***C5***), 4.17 (p, *J* = 7.4 Hz, 1H, ***C4***), 3.63 (s, 3H, ***C1***), 2.88 (d, *J* = 7.4 Hz, 2H, ***C3***).

^{13}C NMR (101 MHz, $CDCl_3$) δ 171.2 (***C2***), 135.8 (***C6***), 133.5 (***C7***), 133.0 (***C8***), 129.2 (***ArC***), 128.0 (***ArC***), 127.8 (***ArC***), 126.7 (***ArC***), 126.5 (***ArC***), 125.0 (***ArC***), 79.5 (***C5***), 52.1 (***C1***), 40.4 (***C4***), 37.7 (***C3***).

HRMS (ESI+, m/Z): exact mass calculated for $C_{15}H_{15}NNaO_4$ $[M+Na]^+$ 296.0893, found 296.0893.

$[\alpha]_D^{25}$ = -4.8 (*c* = 3.38, $CHCl_3$).

FT-IR (thin film): ν_{max} (cm^{-1}) = 2919, 1734, 1552, 1436, 1377, 1216, 1173, 860, 821, 751.

[ToC]

Methyl (3*S*)-3-(4-methylphenyl)-4-nitrobutanoate (**3n**)

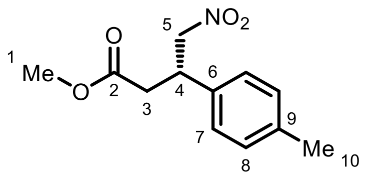

Product **3n** was prepared according to **Procedure 19**, and was obtained as a colorless oil after FCC (Biotage ZIP® 10 g cartridge, pentane : EtOAc 0% to 10%).

yield: 41.7 mg (0.176 mmol, 59%).

e.r.: 96:4 [HPLC CHIRALPAK® AS-H, hexane/IPA = 85/15, 1 mL/min, λ = 220 nm, $t_{(major)}$ = 13.0 min, $t_{(minor)}$ = 16.3 min].

^{1}H NMR (400 MHz, $CDCl_3$) δ 7.19 – 7.07 (m, 4H, ***C7***, ***C8***), 4.71 (dd, *J* = 12.5, 7.1 Hz, 1H, ***C5***), 4.61 (dd, *J* = 12.5, 7.9 Hz, 1H, ***C5***), 3.95 (p, *J* = 7.4 Hz, 1H, ***C4***), 3.63 (s, 3H, ***C10***), 2.76 (dd, *J* = 7.4, 1.4 Hz, 2H, ***C3***), 2.32 (s, 3H, ***C1***).

^{13}C NMR (101 MHz, $CDCl_3$) δ 171.3 (***C2***), 137.9 (***C6***), 135.3 (***C9***), 129.9 (***C8***), 127.2 (***C7***), 79.6 (***C5***), 52.0 (***C1***), 39.9 (***C4***), 37.7 (***C3***), 21.2 (***C10***).

HRMS (ESI+, m/Z): exact mass calculated for $C_{12}H_{15}NNaO_4$ $[M+Na]^+$ 260.0893, found 260.1856.

$[\alpha]_D^{25}$ = -9.34 (*c* = 1.06, $CHCl_3$).

FT-IR (thin film): ν_{max} (cm^{-1}) = 1735, 1552, 1436, 1378, 1169, 996, 818.

Methyl (3*S*)-3-(4-chlorophenyl)-4-nitrobutanoate (**3o**)

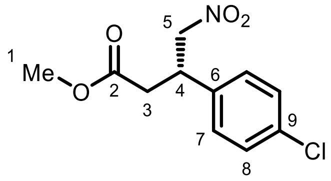

Product **3o** was prepared according to **Procedure 19**, and was obtained as a yellow oil after FCC (Biotage ZIP® 10 g cartridge, [pentane : CH_2Cl_2 33%] : EtOAc 0% to 10%).

yield: 64.1 mg (0.249 mmol, 83%).

e.r.: 95.5:4.5 [HPLC CHIRALPAK® AS-H, hexane/IPA = 85/15, 1 mL/min, λ = 220 nm, $t_{(major)}$ = 17.7 min, $t_{(minor)}$ = 24.5 min].

^{1}H NMR (400 MHz, $CDCl_3$) δ 7.35 – 7.29 (m, 2H, ***C7***), 7.20 – 7.14 (m, 2H, ***C8***), 4.72 (dd, *J* = 12.7, 6.8 Hz, 1H, ***C5***), 4.61 (dd, *J* = 12.7, 8.1 Hz, 1H, ***C5***), 3.97 (p, *J* = 7.4 Hz, 1H, ***C4***), 3.64 (s, 3H, ***C1***), 2.82 – 2.68 (m, 2H, ***C3***).

^{13}C NMR (101 MHz, $CDCl_3$) δ 170.9 (***C2***), 136.9 (***C6***), 134.1 (***C9***), 129.4 (***C7***), 128.9 (***C8***), 79.3 (***C5***), 52.2 (***C1***), 39.7 (***C4***), 37.5 (***C3***).

HRMS (ESI+, m/Z): exact mass calculated for $C_{11}H_{12}ClNNaO_4$ $[M+Na]^+$, 280.0347 found 280.0343.

$[\alpha]_D^{25}$ = -7.31 (*c* = 1.00, $CHCl_3$).

FT-IR (thin film): ν_{max} (cm^{-1}) = 1736, 1554, 1495, 1437, 1377, 1201, 1172, 1096, 1015, 831, 762.

[ToC]

Ethyl (3*S*)-3-(4-fluorophenyl)-4-nitrobutanoate (**3p**)

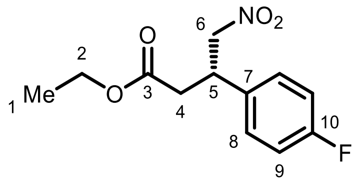

Product **3p** was prepared according to **Procedure 19**, and was obtained as a colorless oil after FCC (Biotage ZIP® 10 g cartridge, [pentane : CH_2Cl_2 25%] : EtOAc 0% to 8%).

yield: 46.5 mg (0.182 mmol, 61%).

e.r.: 95.5:4.5 [HPLC CHIRALPAK® AS-H, hexane/IPA = 90/10, 1 mL/min, λ = 220 nm, $t_{(major)}$ = 18.8 min, $t_{(minor)}$ = 22.0 min].

^{1}H NMR (400 MHz, $CDCl_3$) δ 7.24 – 7.17 (m, 2H, ***C8***), 7.06 – 6.97 (m, 2H, ***C9***), 4.71 (dd, *J* = 12.6, 6.8 Hz, 1H, ***C6***), 4.60 (dd, *J* = 12.6, 8.2 Hz, 1H, ***C6***), 4.08 (qd, *J* = 7.1, 1.0 Hz, 2H, ***C2***), 3.97 (p, *J* = 7.5 Hz, 1H, ***C5***), 2.81 – 2.64 (m, 2H, ***C4***), 1.17 (t, *J* = 7.1 Hz, 3H, ***C1***).

^{13}C NMR (101 MHz, $CDCl_3$) δ 170.5 (***C3***), 162.4 (d, *J* = 246.8 Hz, ***C10***), 134.2 (d, *J* = 3.4 Hz, ***7***, 129.2 (d, *J* = 8.2 Hz, ***C8***), 116.1 (d, *J* = 21.5 Hz, ***C9***), 79.5 (***C6***), 61.1 (***C2***), 39.7 (***C5***), 37.9 (***C4***), 14.2 (***C1***).

^{19}F NMR (376 MHz, $CDCl_3$) δ -114.0 (tt, *J* = 8.4, 5.1 Hz).

HRMS (ESI+, m/Z): exact mass calculated for $C_{12}H_{15}FNO_4$ $[M+H]^+$, 256.0980 found 256.0982.

$[\alpha]_D^{25}$ = -14.0 (*c* = 1.12, $CHCl_3$).

FT-IR (thin film): ν_{max} (cm^{-1}) = 1732, 1606, 1556, 1513, 1434, 1378, 1228, 1191, 1163, 1103, 1026, 838, 767.

Methyl (3*S*)-4-nitro-3-[4-(trifluoromethyl)phenyl]butanoate (**3q**)

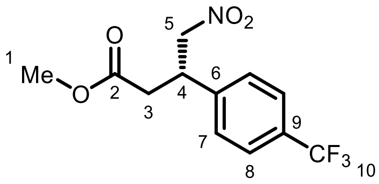

Product **3q** was prepared according to **Procedure 19**, and was obtained as a colorless oil after FCC (pentane : EtOAc 0% to 10%).

yield: 71.6 mg (0.246 mmol, 82%).

e.r.: 95.5:4.5 [HPLC CHIRALPAK® AS-H, hexane/IPA = 80/20, 1 mL/min, λ = 220 nm, $t_{(major)}$ = 10.2 min, $t_{(minor)}$ = 12.4 min].

^{1}H NMR (400 MHz, $CDCl_3$) δ 7.65 – 7.57 (m, 2H, ***C7***), 7.40 – 7.34 (m, 2H, ***C8***), 4.77 (dd, *J* = 12.9, 6.7 Hz, 1H, ***C5***), 4.67 (dd, *J* = 12.9, 8.2 Hz, 1H, ***C5***), 4.07 (p, *J* = 7.4 Hz, 1H, ***C4***), 3.65 (s, 3H, ***C1***), 2.87 – 2.72 (m, 2H, ***C3***).

^{13}C NMR (101 MHz, $CDCl_3$) δ 170.8 (***C2***), 142.5 (***C6***), 130.5 (q, J^2_{CF3} = 32.6 Hz, ***C9***), 128.0 (***C7***), 126.2 (q, J^3_{CF3} = 3.8 Hz, ***C8***), 124.0 (d, J^1_{CF3} = 272.2 Hz, ***C10***), 78.9 (***C5***), 52.2 (***C1***), 40.0 (***C4***), 37.3 (***C3***).

^{19}F NMR (376 MHz, $CDCl_3$) δ -62.7.

HRMS (ESI+, m/Z): exact mass calculated for $C_{12}H_{11}F_3NO_4$ $[M-H]^-$ 290.0646, found 290.0639.

$[\alpha]_D^{25}$ = -11.8 (*c* = 3.10, $CHCl_3$).

FT-IR (thin film): ν_{max} (cm^{-1}) = 1736, 1621, 1556, 1438, 1378, 1326, 1167, 1116, 1071, 1018, 842, 652.

[ToC]

Ethyl (3*S*)-4-nitro-3-(4-nitrophenyl)butanoate (**3r**)

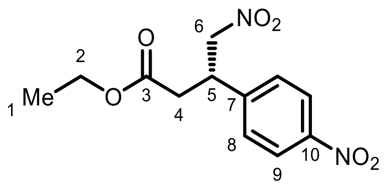

Product **3r** was prepared according to **Procedure 19**, and was obtained as a yellow solid after preparative TLC (toluene : EtOAc 10%).

yield: 32.4 mg (0.121 mmol, 38%).

^{1}H NMR (400 MHz, $CDCl_3$) δ 8.25 – 8.14 (m, 2H, ***C9***), 7.48 – 7.40 (m, 2H, ***C8***), 4.79 (dd, *J* = 13.0, 6.4 Hz, 1H, ***C6***), 4.69 (dd, *J* = 13.0, 8.5 Hz, 1H, ***C6***), 4.17 – 4.03 (m, 3H, ***C2***, ***C5***), 2.87 – 2.71 (m, 2H, ***C4***), 1.18 (t, *J* = 7.2 Hz, 3H, ***C1***).

^{13}C NMR (101 MHz, $CDCl_3$) δ 170.0 (***C3***), 147.8 (***C10***), 145.9 (***C7***), 128.7 (***C8***), 124.4 (***C9***), 78.7 (***C6***), 61.4 (***C2***), 40.0 (***C5***), 37.4 (***C4***), 14.2 (***C1***).

HRMS (ESI+, m/Z): exact mass calculated for $C_{12}H_{14}N_2NaO_6$ $[M+Na]^+$ 305.0744, found 305.0746.

m. p.: 84 – 90 ºC

$[\alpha]_D^{25}$ = -5.4 (*c* = 2.90, $CHCl_3$).

FT-IR (thin film): ν_{max} (cm^{-1}) = 1731, 1555, 1522, 1377, 1348, 1180, 857.

Octanoyl (3*S*)-4-nitro-3-(4-nitrophenyl)butanoate (**3s**)

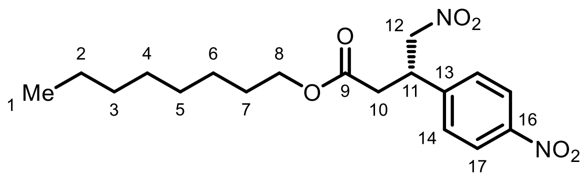

Product **3s** was prepared according to **Procedure 19**, and was obtained as a yellow oil after FCC (Biotage ZIP® 10 g cartridge, [pentane : CH_2Cl_2 25%] : EtOAc 0% to 10%).

yield: 68.0 mg (0.186 mmol, 62%).

e.r.: 93.5:6.5 [HPLC CHIRALPAK® AS-H, hexane/IPA = 85/15, 1 mL/min, λ = 220 nm, $t_{(major)}$ = 19.5 min, $t_{(minor)}$ = 23.7 min].

^{1}H NMR (400 MHz, $CDCl_3$) δ 8.25 – 8.15 (m, 2H, ***C15***), 7.50 – 7.39 (m, 2H, ***C14***), 4.90 – 4.60 (m, 2H, ***C12***), 4.11 (p, 1H, ***C11***), 4.02 (t, *J* = 6.7 Hz, 2H, ***C8***), 2.87 – 2.71 (m, 2H, ***C10***), 1.53 (p, *J* = 6.9 Hz, 2H, ***C7***), 1.36 – 1.16 (m, 10H, ***C2*** – ***C6***), 0.87 (t, *J* = 1.3 Hz, 3H, ***C1***).

^{13}C NMR (101 MHz, $CDCl_3$) δ 170.1 (***C9***), 147.8 (***C16***), 145.9 (***C13***), 128.7 (***C14***), 124.3 (***C15***), 78.7 (***C12***), 65.6 (***C8***), 40.0 (***C11***), 37.4 (***C10***), 31.8 (***C2*** – ***C6***), 29.2 (***C2*** – ***C6***), 28.6 (***C7***), 25.9 (***C2*** – ***C6***), 22.7 (***C2*** – ***C6***), 14.2 (***C1***).

HRMS (ESI+, m/Z): exact mass calculated for $C_{18}H_{26}N_2NaO_6$ $[M+Na]^+$ 389.1683, found 389.1685.

$[\alpha]_D^{25}$ = -3.4 (*c* = 1.46, $CHCl_3$).

FT-IR (thin film): ν_{max} (cm^{-1}) = 2925, 2856, 1733, 1557, 1542, 1348, 1177, 856, 613.

[[ToC]]

Methyl (3*S*)-3-(3-cyanophenyl)-4-nitrobutanoate (**3t**)

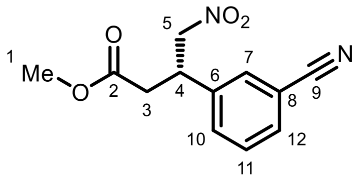

Product **3t** was prepared according to **Procedure 19**, and was obtained as a yellow oil after FCC (Biotage ZIP® 5 g cartridge, pentane : CH_2Cl_2 0% to 80%).

yield: 52.0 mg (0.210 mmol, 70%).

e.r.: 94.5:5.5 [HPLC CHIRALPAK® IB, hexane/IPA = 80/20, 1 mL/min, λ = 220 nm, $t_{(minor)}$ = 19.7 min, $t_{(major)}$ = 25.2 min].

^{1}H NMR (400 MHz, $CDCl_3$) δ 7.59 (dt, *J* = 7.2, 1.6 Hz, 1H, ***C12***), 7.56 – 7.53 (m, 1H, ***C7***), 7.53 – 7.43 (m, 2H, ***C10***, ***C11***), 4.81 – 4.61 (m, 2H, ***C5***), 4.02 (p, *J* = 7.1 Hz, 1H, ***C4***), 3.64 (s, 3H, ***C1***), 2.86 – 2.69 (m, 2H, ***C3***).

^{13}C NMR (101 MHz, $CDCl_3$) δ 170.6 (***C2***), 140.1 (***C6***), 132.2 (***C11***), 131.9 (***C12***), 131.2 (***C7***), 130.1 (***C10***), 118.4 (***C9***), 113.4 (***C8***), 78.7 (***C5***), 52.3 (***C1***), 39.7 (***C4***), 37.1 (***C3***).

HRMS (ESI+, m/Z): exact mass calculated for $C_{12}H_{13}N_2O_4$ $[M+H]^+$ 249.0870, found 249.0870.

$[\alpha]_D^{25}$ = +6.4 (*c* = 4.68, $CHCl_3$).

FT-IR (thin film): ν_{max} (cm^{-1}) = 2231, 1734, 1553, 1437, 1378, 1215, 1174, 804, 756, 694.

Ethyl (3*S*)-3-(2-bromophenyl)-4-nitrobutanoate (**3u**)

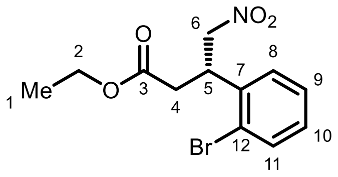

Product **3u** was prepared according to **Procedure 19**, and was obtained as a colorless oil after FCC (pentane : EtOAc 10% to 20%).

yield: 69.5 mg (0.220 mmol, 73%).

e.r.: 91.5:8.5 [HPLC CHIRALPAK® IA, hexane/IPA = 95/05, 1 mL/min, λ = 220 nm, $t_{(minor)}$ = 9.9 min, $t_{(major)}$ = 11.0 min].

^{1}H NMR (400 MHz, $CDCl_3$) δ 7.59 (dd, *J* = 8.0, 1.3 Hz, 1H, ***C10***), 7.34 – 7.25 (m, 1H, ***C11***), 7.24 – 7.19 (m, 1H, ***C9***), 7.18 – 7.09 (m, 1H, ***C8***), 4.83 – 4.69 (m, 2H, ***C6***), 4.48 (p, *J* = 7.1 Hz, 1H, ***C5***), 4.16 – 4.03 (m, 2H, ***C2***), 2.92 – 2.75 (m, 2H, ***C4***), 1.17 (t, *J* = 7.1 Hz, 3H, ***C1***).

^{13}C NMR (101 MHz, $CDCl_3$) δ 170.5 (***C3***), 137.3 (***C7***), 133.8 (***C11***), 129.5 (***C10***), 128.1 (***C8***), 128.0 (***C9***), 124.6 (***C12***), 77.8 (***C6***), 61.1 (***C2***), 39.0 (***C5***), 36.4 (***C4***), 14.1 (***C1***).

HRMS (ESI+, m/Z): exact mass calculated for $C_{12}H_{14}BrNNaO_4$ $[M+Na]^+$ 337.9998, found 337.9999.

$[\alpha]_D^{25}$ = -6.2 (*c* = 1.13, $CHCl_3$).

FT-IR (thin film): ν_{max} (cm^{-1}) = 2850, 2360, 1732, 1553, 1473, 1438, 1377, 1192, 1024, 759.

[ToC]

Methyl (3*S*)-3-(5-bromo-2-methoxyphenyl)-4-nitrobutanoate (**3v**)

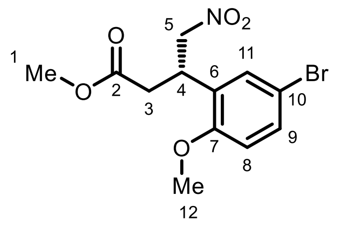

Product **3v** was prepared according to **Procedure 19**, and was obtained as a colorless oil after FCC (pentane : EtOAc 10%).

yield: 73.5 mg (0.222 mmol, 74%).

e.r.: 95:5 [HPLC CHIRALPAK® IB, hexane/IPA = 80/20, 1 mL/min, λ = 220 nm, $t_{(minor)}$ = 8.8 min, $t_{(major)}$ = 10.2 min].

^{1}H NMR (400 MHz, $CDCl_3$) δ 7.34 (dd, *J* = 8.7, 2.5 Hz, 1H, ***C11***), 7.25 (d, *J* = 2.6 Hz, 1H, ***C9***), 6.76 (d, *J* = 8.7 Hz, 1H, ***C8***), 4.81 – 4.68 (m, 2H, ***C5***), 4.11 (p, *J* = 7.1 Hz, 1H, ***C4***), 3.83 (s, 3H, ***C12***), 3.64 (s, 3H, ***C1***), 2.90 – 2.74 (m, 2H, ***C3***).

^{13}C NMR (101 MHz, $CDCl_3$) δ 171.5 (***C2***), 156.4 (***C7***), 132.2 (***C11***), 131.9 (***C9***), 128.4 (***C6***), 113.1 (***C10***), 112.9 (***C8***), 77.4 (***C5***), 55.8 (***C12***), 52.0 (***C1***), 36.5 (***C3***), 35.5 (***C4***).

HRMS (ESI+, m/Z): exact mass calculated for $C_{12}H_{14}BrNNaO_5$ $[M+Na]^+$ 353.9948, found 353.9948.

$[\alpha]_D^{25}$ = +4.3 (*c* = 1.03, $CHCl_3$).

FT-IR (thin film): ν_{max} (cm^{-1}) = 1735, 1551, 1490, 1438, 1377, 1249, 1174, 1134, 1025, 811, 625.

Ethyl (3*S*)-3-(2,4-difluorophenyl)-4-nitrobutanoate (**3w**)

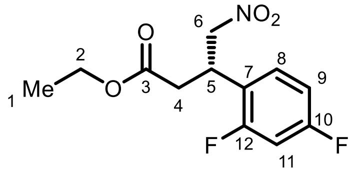

Product **3w** was prepared according to **Procedure 19**, and was obtained as a colorless oil after FCC (Biotage ZIP® 10 g cartridge, [pentane : CH_2Cl_2 25%] : EtOAc 0% to 10%).

yield: 59.2 mg (0.217 mmol, 72%).

e.r.: 94.5:5.5 [HPLC CHIRALPAK® AS-H, hexane/IPA = 85/15, 1 mL/min, λ = 220 nm, $t_{(major)}$ = 10.8 min, $t_{(minor)}$ = 12.3 min].

^{1}H NMR (400 MHz, $CDCl_3$) δ 7.25 – 7.17 (m, 1H, ***C8***), 6.89 – 6.77 (m, 2H, ***C9***, ***C11***), 4.81 – 4.66 (m, 2H, ***C6***), 4.19 – 4.03 (m, 3H, ***C2***, ***C5***), 2.88 – 2.70 (m, 2H, ***C4***), 1.19 (t, *J* = 7.1 Hz, 3H, ***C1***).

^{13}C NMR (101 MHz, $CDCl_3$) δ 170.5 (***C3***), 163.2 (dd, *J* = 162.2, 12.3 Hz, ***C10***), 160.7 (dd, *J* = 161.4, 12.2 Hz, ***C12***), 130.8 (dd, *J* = 9.7, 6.2 Hz, ***C8***), 121.2 (dd, *J* = 13.7, 4.0 Hz, ***C7***), 111.9 (dd, *J* = 21.2, 3.6 Hz, ***C9***), 104.8 (app. t, *J* = 25.8 Hz, ***C11***), 77.8 (d, *J* = 2.7 Hz, ***C6***), 61.2 (***C2***), 36.3 (d, *J* = 2.0 Hz, ***C4***), 35.4 (***C5***), 14.2 (***C1***).

^{19}F NMR (376 MHz, $CDCl_3$) δ -109.6 – -110.3 (m), -112.1 – -113.1 (m).

HRMS (ESI+, m/Z): exact mass calculated for $C_{12}H_{13}F_2NNaO_4$ $[M+Na]^+$ 296.0705, found 296.0707.

$[\alpha]_D^{25}$ = -4.2 (*c* = 1.75, $CHCl_3$).

FT-IR (thin film): ν_{max} (cm^{-1}) = 1732, 1619, 1556, 1506, 1430, 1378, 1273, 1185, 1142, 1100, 1025, 967, 853, 618.

[ToC]

Methyl (3*S*)-3-[3-(cyclopentyloxy)-4-methoxyphenyl]-4-nitrobutanoate (3x)

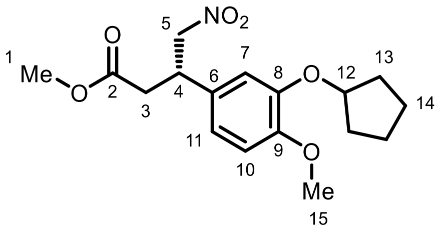

Product **3x** was prepared according to **Procedure 19**, and was obtained as a colorless powder after FCC (Biotage ZIP® 10 g cartridge, [pentane : CH_2Cl_2 25%] : EtOAc 0% to 20%).

yield: 49.1 mg (0.146 mmol, 49%).

e.r.: 96:4 [HPLC CHIRALPAK® AS-H, hexane/IPA = 80/20, 1 mL/min, λ = 220 nm, $t_{(major)}$ = 14.0 min, $t_{(minor)}$ = 16.6 min].

^{1}H NMR (400 MHz, $CDCl_3$) δ 6.83 – 6.79 (m, 1H, ***C10***), 6.75 – 6.70 (m, 2H, ***C7***, ***C11***), 4.78 – 4.73 (m, 1H, ***C12***), 4.69 (dd, *J* = 12.4, 7.1 Hz, 1H, ***C5***), 4.60 (dd, *J* = 12.5, 7.8 Hz, 1H, ***C5***), 3.90 (p, *J* = 7.4 Hz, 1H, ***C4***), 3.81 (s, 3H, ***C15***), 3.63 (s, 3H, ***C1***), 2.80 – 2.68 (m, 2H, ***C3***), 1.98 – 1.75 (m, 6H, ***C13***, ***C14***), 1.67 – 1.55 (m, 2H, ***C14***).

^{13}C NMR (101 MHz, $CDCl_3$) δ 171.3 (***C2***), 149.9 (***C9***), 148.0 (***C8***), 130.6 (***C6***), 119.3 (***C11***), 114.5 (***C7***), 112.4 (***C10***), 80.7 (***C12***), 79.8 (***C15***), 56.1 (***C15***), 52.1 (***C1***), 39.9 (***C4***), 37.8 (***C3***), 32.9 (***C13***), 32.9 (***C13'***), 24.2 (***C14***).

HRMS (ESI+, m/Z): exact mass calculated for $C_{17}H_{24}NO_6$ $[M+H]^+$, 338.1598 found 338.1599.

m. p.: 100 – 102 ºC. {lit.: m.p. = 103 – 105 ºC}[18]

$[\alpha]_D^{25}$ = -9.8 (*c* = 0.85, $CHCl_3$). {lit.: $[\alpha]_D^{25}$ = -11.8 (*c* = 1.00, $CHCl_3$)}[18]

FT-IR (thin film): ν_{max} (cm^{-1}) = 2953, 1735, 1555, 1517, 1375, 1238, 1140, 764.

Methyl (3*R*)-4-nitro-3-(pyridin-2-yl)butanoate (3y)

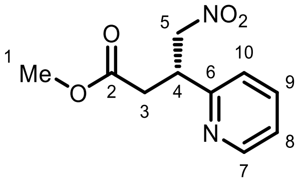

Product **3y** was prepared according to **Procedure 19**, and was obtained as a green oil after FCC (Biotage ZIP® 5 g cartridge, pentane : EtOAc 0% to 15%).

yield: 58.5 mg (0.261 mmol, 87%).

e.r.: 95:5 [HPLC CHIRALPAK® OD, hexane/IPA = 85/15, 1 mL/min, λ = 220 nm, $t_{(minor)}$ = 10.0 min, $t_{(major)}$ = 11.7 min].

^{1}H NMR (400 MHz, $CDCl_3$) δ 8.55 – 8.49 (m, 1H, ***C7***), 7.67 – 7.58 (m, 1H, ***C9***), 7.30 – 7.23 (m, 1H, ***C10***), 7.21 – 7.13 (m, 1H, ***C8***), 4.93 (dd, *J* = 13.3, 8.5 Hz, 1H, ***C5***), 4.74 (dd, *J* = 13.3, 5.9 Hz, 1H, ***C5***), 4.14 – 4.03 (m, 1H, ***C4***), 3.62 (s, 3H, ***C1***), 2.88 (dd, *J* = 16.5, 7.7 Hz, 1H, ***C3***), 2.74 (dd, *J* = 16.6, 6.7 Hz, 1H, ***C3***).

^{13}C NMR (101 MHz, $CDCl_3$) δ 171.4 (***C2***), 158.1 (***C6***), 149.8 (***C7***), 137.0 (***C9***), 123.8 (***C10***), 77.9 (***C8***), 52.0 (***C5***), 41.3 (***C1***), 36.7 (***C4***), 31.4 (***C3***).

HRMS (ESI+, m/Z): exact mass calculated for $C_{10}H_{13}N_2O_4$ $[M+H]^+$ 225.0870, found 225.0870.

$[\alpha]_D^{25}$ = +1.8 (*c* = 3.35, $CHCl_3$).

FT-IR (thin film): ν_{max} (cm^{-1}) = 1735, 1593, 1551, 1438, 1378, 1206, 1171, 1000, 789, 752.

[ToC]

Methyl (3*S*)-4-nitro-3-(pyridin-3-yl)butanoate (**3z**)

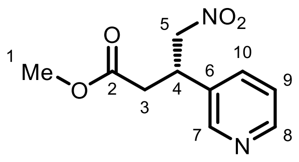

Product **3z** was prepared according to **Procedure 19**, and was obtained as a colorless oil after FCC (pentane : EtOAc 20% to 80%).

yield: 60.5 mg (0.270 mmol, 90%).

e.r.: 94.5:5.5 [HPLC CHIRALPAK® AD-H, hexane/IPA = 80/20, 1 mL/min, λ = 220 nm, $t_{(minor)}$ = 11.5 min, $t_{(major)}$ = 15.6 min].

^{1}H NMR (400 MHz, $CDCl_3$) δ 8.57 – 8.51 (m, 2H, ***C7***, ***C8***), 7.60 – 7.53 (m, 1H, ***C10***), 7.31 – 7.23 (m, 1H, ***C9***), 4.78 (dd, *J* = 12.9, 6.7 Hz, 1H, ***C5***), 4.67 (dd, *J* = 12.9, 8.1 Hz, 1H, ***C5***), 4.01 (p, *J* = 7.3 Hz, 1H, ***C4***), 3.64 (s, 3H, ***C1***), 2.88 – 2.71 (m, 2H, ***C3***).

^{13}C NMR (101 MHz, $CDCl_3$) δ 170.7 (***C2***), 149.7 (***C7***), 149.3 (***C8***), 135.0 (***C10***), 134.1 (***C6***), 123.9 (***C9***), 78.8 (***C5***), 52.2 (***C4***), 37.9 (***C4***), 37.2 (***C3***).

HRMS (ESI+, m/Z): exact mass calculated for $C_{10}H_{13}N_2O_4$ $[M+H]^+$ 225.0870, found 225.0868.

$[\alpha]_D^{25}$ = -11.2 (*c* = 5.80, $CHCl_3$).

FT-IR (thin film): ν_{max} (cm^{-1}) = 1734, 1552, 1431, 1378, 1280, 1172, 814, 715.

Methyl (3*S*)-4-nitro-3-(pyridin-4-yl)butanoate (**3aa**)

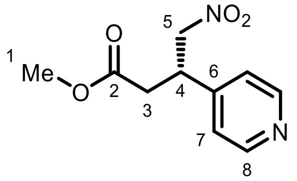

Product **3aa** was prepared according to **Procedure 19**, and was obtained as a yellow oil after preparative TLC (hexane : EtOAc 70%).

yield: 67.3 mg (0.299 mmol, 99%).

e.r.: 93.5:6.5 [HPLC CHIRALPAK® AD-H, hexane/IPA = 80/20, 1 mL/min, λ = 220 nm, $t_{(major)}$ = 10.7 min, $t_{(minor)}$ = 11.8 min].

^{1}H NMR (400 MHz, $CDCl_3$) δ 8.61 – 8.55 (m, 2H, ***C8***), 7.20 – 7.14 (m, 2H, ***C7***), 4.81 – 4.62 (m, 2H, ***C5***), 3.97 (p, *J* = 7.3 Hz, 1H, ***C4***), 3.64 (s, 3H, ***C1***), 2.85 – 2.70 (m, 2H, ***C3***).

^{13}C NMR (101 MHz, $CDCl_3$) δ 170.6 (***C2***), 150.7 (***C8***), 147.4 (***C6***), 122.6 (***C7***), 78.3 (***C5***), 52.3 (***C1***), 39.4 (***C4***), 36.8 (***C3***).

HRMS (ESI+, m/Z): exact mass calculated for $C_{10}H_{13}N_2O_4$ $[M+H]^+$ 225.0870, found 225.0870.

$[\alpha]_D^{25}$ = -5.2 (*c* = 4.00, $CHCl_3$).

FT-IR (thin film): ν_{max} (cm^{-1}) = 3033, 2954, 1734, 1600, 1553, 1437, 1417, 1378, 1203, 1173, 994, 827.

Methyl (3*S*)-4-nitro-3-(quinolin-3-yl)butanoate (**3ab**)

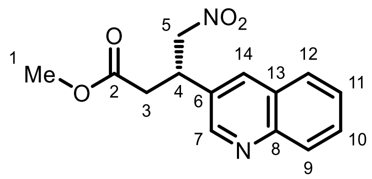

Product **3ab** was prepared according to **Procedure 19**, and was obtained as a colorless solid after FCC (CH_2Cl_2 : MeCN 0% to 15%).

yield: 38.7 mg (0.141 mmol, 47%).

e.r.: 94.5:5.5 [HPLC CHIRALPAK® IB, hexane/IPA = 70/30, 1 mL/min, λ = 220 nm, $t_{(minor)}$ = 14.8 min, $t_{(major)}$ = 30.0 min].

^{1}H NMR (400 MHz, $CDCl_3$) δ 8.84 (d, *J* = 2.4 Hz, 1H, ***C7***), 8.13 – 8.06 (m, 1H, ***C14***), 8.02 (d, *J* = 2.4 Hz, 1H, ***C9***), 7.80 (dd, *J* = 8.0, 1.4 Hz, 1H, ***C12***), 7.77 – 7.68 (m, 1H, ***C10***), 7.61 – 7.53 (m, 1H, ***C11***), 4.87 (dd, *J* = 13.0, 6.7 Hz, 1H, ***C5***), 4.79 (dd, *J* = 13.0, 8.0 Hz, 1H, ***C5***), 4.22 (p, *J* = 7.3 Hz, 1H, ***C4***), 3.64 (s, 3H, ***C1***), 2.99 – 2.83 (m, 2H, ***C3***).

^{13}C NMR (101 MHz, $CDCl_3$) δ 170.7 (***C2***), 150.1 (***C7***), 148.0 (***C8***), 134.4 (***C9***), 131.3 (***C6***), 130.1 (***C10***), 129.5 (***C14***), 127.9 (***C12***), 127.8 (***C13***), 127.4 (***C11***), 78.8 (***C5***), 52.3 (***C1***), 38.0 (***C4***), 37.4 (***C3***).

HRMS (ESI+, m/Z): exact mass calculated for $C_{14}H_{15}N_2O_4$ $[M+H]^+$ 275.1026, found 275.1026.

m. p.: 98 – 100 °C

$[\alpha]_D^{25}$ = +1.5 (*c* = 1.05, $CHCl_3$).

FT-IR (thin film): ν_{max} (cm^{-1}) = 2918, 1735, 1553, 1437, 1376, 1263, 1216, 1175, 790, 756.

Methyl (3*S*)-3-[2-(methylsulfanyl)pyrimidin-5-yl]-4-nitrobutanoate (**3ac**)

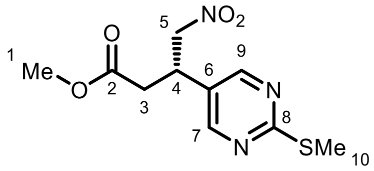

Product **3ac** was prepared according to **Procedure 19**, and was obtained as a colorless oil after FCC (Biotage ZIP® 10 g cartridge, [pentane : CH_2Cl_2 10%] : EtOAc 0% to 6%).

yield: 45.4 mg (0.167 mmol, 56%).

e.r.: 93.5:6.5 [HPLC CHIRALPAK® OD-H, hexane/IPA = 80/20, 1 mL/min, λ = 220 nm, $t_{(minor)}$ = 31.6 min, $t_{(major)}$ = 45.5 min].

^{1}H NMR (400 MHz, $CDCl_3$) δ 8.43 (s, 2H, ***C7***, ***C9***), 4.77 (dd, *J* = 13.1, 6.4 Hz, 1H, ***C5***), 4.65 (dd, *J* = 13.1, 8.3 Hz, 1H, ***C5***), 3.98 – 3.86 (m, 1H, ***C4***), 3.66 (s, 3H, ***C1***), 2.87 – 2.69 (m, 2H, ***C3***), 2.55 (s, 3H, ***C10***).

^{13}C NMR (101 MHz, $CDCl_3$) δ 173.0 (***C2***), 170.4 (***C8***), 156.5 (***C7***, ***C9***), 126.6 (***C6***), 78.2 (***C5***), 52.4 (***C1***), 36.8 (***C3***), 35.5 (***C4***), 14.3 (***C10***).

HRMS (ESI+, m/Z): exact mass calculated for $C_{10}H_{14}N_3O_4S$ $[M+H]^+$ 272.0700, found 272.0698.

$[\alpha]_D^{25}$ = +2.0 (*c* = 2.60, $CHCl_3$).

FT-IR (thin film): ν_{max} (cm^{-1}) = 1734, 1586, 1553, 1436, 1401, 1377, 1213, 1174, 777, 645.

[ToC]

Methyl (3*R*)-3-(furan-2-yl)-4-nitrobutanoate (**3ad**)

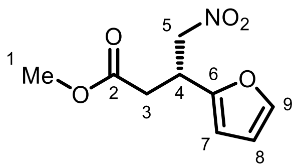

Product **3ad** was prepared according to **Procedure 19**, and was obtained as a colorless oil after FCC (Biotage ZIP® 10 g cartridge, [pentane : CH_2Cl_2 15%] : EtOAc 0% to 10%).

yield: 40.5 mg (0.190 mmol, 63%).

e.r.: 96:4 [HPLC CHIRALPAK® AS-H, hexane/IPA = 80/20, 1 mL/min, λ = 220 nm, $t_{(minor)}$ = 15.1 min, $t_{(minor)}$ = 22.6 min].

^{1}H NMR (400 MHz, $CDCl_3$) δ 7.37 – 7.32 (m, 1H, ***C9***), 6.33 – 6.26 (m, 1H, ***C8***), 6.20 – 6.14 (m, 1H, ***C7***), 4.72 (d, *J* = 6.9 Hz, 2H, ***C5***), 4.15 – 4.03 (m, 1H, ***C4***), 3.69 (s, 3H, ***C1***), 2.88 – 2.71 (m, 2H, ***C3***).

^{13}C NMR (101 MHz, $CDCl_3$) δ 171.0 (***C2***), 151.3 (***C6***), 142.6 (***C9***), 110.6 (***C8***), 107.3 (***C7***), 77.1 (***C5***), 52.2 (***C1***), 35.2 (***C3***), 34.2 (***C4***).

HRMS (ESI+, m/Z): exact mass calculated for $C_9H_{12}NO_5$ $[M+H]^+$ 214.0710, found 214.0897.

$[\alpha]_D^{25}$ = -3.2 (*c* = 1.42, $CHCl_3$).

FT-IR (thin film): ν_{max} (cm^{-1}) = 2921, 1735, 1556, 1437, 1209, 1015, 741, 668.

Ethyl (3*R*)-4-nitro-3-(1,3,4-oxadiazol-2-yl)butanoate (**3ae**)

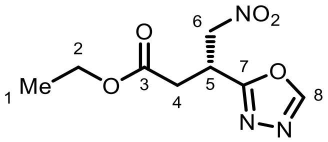

Product **3ae** was prepared according to **Procedure 19**, and was obtained as a yellow oil after FCC (pentane : EtOAc 10% to 80%), and preparative TLC (CH_2Cl_2 : MeCN 10%).

yield: 23.8 mg (0.104 mmol, 35%).

e.r.: 91:9 [HPLC CHIRALPAK® IA, hexane/IPA = 90/10, 1 mL/min, λ = 220 nm, $t_{(minor)}$ = 7.7 min, $t_{(major)}$ = 8.4 min].

^{1}H NMR (400 MHz, $CDCl_3$) δ 8.40 (s, 1H, ***C8***), 5.01 (dd, *J* = 14.2, 7.1 Hz, 1H, ***C6***), 4.89 (dd, *J* = 14.3, 5.7 Hz, 1H, ***C6***), 4.34 – 4.23 (m, 1H, ***C5***), 4.17 (q, *J* = 7.2 Hz, 2H, ***C2***), 3.05 (dd, *J* = 17.3, 6.3 Hz, 1H, ***C4***), 2.91 (dd, *J* = 17.3, 7.0 Hz, 1H, ***C4***), 1.26 (t, *J* = 7.2 Hz, 3H, ***C1***).

^{13}C NMR (101 MHz, $CDCl_3$) δ 169.6 (***C3***), 165.0 (***C7***), 153.5 (***C8***), 74.8 (***C6***), 61.8 (***C2***), 34.1 (***C4***), 31.8 (***C5***), 14.2 (***C1***).

HRMS (ESI+, m/Z): exact mass calculated for $C_8H_{12}N_3O_5$ $[M+H]^+$ 230.0771, found 230.0773.

$[\alpha]_D^{25}$ = +8.3 (*c* = 1.26, $CHCl_3$).

FT-IR (thin film): ν_{max} (cm^{-1}) = 1731, 1558, 1376, 1210, 1097, 1026, 957, 642.

[ToC]

Methyl (3*S*)-4-nitro-3-(thiophen-3-yl)butanoate (**3af**)

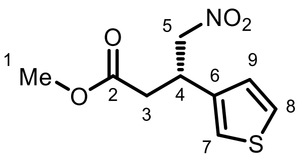

Product **3af** was prepared according to **Procedure 19**, and was obtained as a yellow oil after preparative TLC (hexane : EtOAc 20%).

yield: 47.3 mg (0.207 mmol, 69%).

e.r.: 95.5:4.5 [HPLC CHIRALPAK® OD, hexane/IPA = 85/15, 1 mL/min, λ = 220 nm, $t_{(minor)}$ = 14.6 min, $t_{(major)}$ = 17.4 min].

^{1}H NMR (400 MHz, $CDCl_3$) δ 7.23 (dd, *J* = 5.0, 1.4 Hz, 1H, ***C8***), 6.98 – 6.90 (m, 2H, ***C7***, ***C9***), 4.77 (dd, *J* = 12.7, 6.8 Hz, 1H, ***C5***), 4.66 (dd, *J* = 12.7, 7.5 Hz, 1H, ***C5***), 4.31 (p, *J* = 7.1 Hz, 1H, ***C4***), 3.68 (s, 3H, ***C1***), 2.83 (d, *J* = 7.1 Hz, 2H, ***C3***).

^{13}C NMR (101 MHz, $CDCl_3$) δ 170.9 (***C2***), 141.0 (***C6***), 127.3 (***C9***), 125.7 (***C8***), 125.1 (***C7***), 79.7 (***C5***), 52.2 (***C1***), 38.5 (***C4***), 35.8 (***C3***).

HRMS (ESI+, m/Z): ion not found.

$[\alpha]_D^{25}$ = -14.9 (*c* = 3.67, $CHCl_3$).

FT-IR (thin film): ν_{max} (cm^{-1}) = 2959, 1736, 1554, 1437, 1377, 1279, 1175, 1136, 851, 704.

Ethyl 2-((1*R*,2*S*)-2-nitrocyclohexyl)acetate (**3ag**)

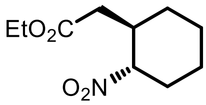

Following a modification to **Procedure 19**, to a solution of **1ag** (64.5 mg, 0.30 mmol) in cyclohexane (150 µL) was added **B4** (reisolated, 62 mg, 0.045 mmol). The resulting mixture was stirred for 24 hours before being passed through a silica plug. The volatiles were removed to yield the crude residue (d.r. 10:1). The crude residue was purified by FCC (5:1 to 3:1 pentane:EtOAc) to yield **3ag** as a colorless oil (49 mg, 76%, d.r. 11:1). Data were consistent with those reported in the literature.[5b]

yield: 49.0 mg (0.228 mmol, 76%).

e.r.: 98:2 (major diastereomer) [SFC CHIRALPAK® IF, 0% MeOH for 3 minutes; then 0% to 10% MeOH over 5 min, then from 10% to 30% MeOH in 0.5 min, then from 30% to 50% MeOH in 0.5 min, then hold 50% MeOH for 2 mins, 1.5 mL/min, λ = 215 nm, $t_{(major)}$ = 6.9 min, $t_{(minor)}$ = 7.1 min].

NMR data for major diastereomer:

^{1}H NMR (400 MHz, $CDCl_3$) δ 4.39 – 4.30 (m, 1H), 4.12 (q, *J* = 7.2 Hz, 2H), 2.47 – 2.33 (m, 2H), 2.30 – 2.13 (m, 2H), 2.00 – 1.81 (m, 3H), 1.79 – 1.67 (m, 1H), 1.42 – 1.29 (m, 2H), 1.25 (t, *J* = 7.1 Hz, 3H), 1.22 – 1.10 (m, 1H).

^{13}C NMR (101 MHz, $CDCl_3$) δ 171.3, 89.8, 60.8, 37.9, 37.5, 32.0, 30.3, 24.7, 24.4, 14.3.

$[\alpha]_D^{25}$ = +26.4 (*c* = 1.05, $CHCl_3$). {lit.: $[\alpha]_D^{25}$ = +22.5 (*c* = 1.04, $CHCl_3$)}[5b]

γ-Nitroester Derivatives

(4*R*,5*S*)-4-Methyl-5-nitropiperidin-2-one (**4a**)

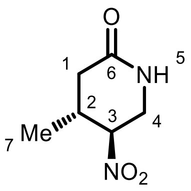

Product **4a** was prepared according to **Procedure 21**, and was obtained as a yellow solid.

yield: 136 mg (0.84 mmol, 84%).

e.r.: 95.5:4.5 [HPLC CHIRALPAK® AD, hexane/IPA = 85/15, 1 mL/min, λ = 220 nm, $t_{(minor)}$ = 10.7 min, $t_{(major)}$ = 12.4 min].

^{1}H NMR (400 MHz, $CDCl_3$) δ 6.13 (s, 1H, ***N5***), 4.58 – 4.44 (m, 1H, ***C3***), 3.98 – 3.88 (m, 1H, ***C4***), 3.80 – 3.68 (m, 1H, ***C4***), 2.84 – 2.69 (m, 1H, ***C2***), 2.61 (dd, *J* = 17.7, 5.9 Hz, 1H, ***C1***), 2.18 (dd, *J* = 17.7, 8.6 Hz, 1H, ***C1***), 1.17 (d, *J* = 6.7 Hz, 3H, ***C7***).

^{13}C NMR (101 MHz, $CDCl_3$) δ 169.8 (***C6***), 84.8 (***C3***), 43.0 (***C4***), 36.2 (***C1***), 31.6 (***C2***), 18.4 (***C7***).

HRMS (ESI+, m/Z): exact mass calculated for $C_6H_{11}N_2O_3$ $[M+H]^+$ 159.0764, found 159.0765.

m. p.: 128 – 130 ºC

$[\alpha]_D^{25}$ = -5.8 (*c* = 0.93, $CHCl_3$).

FT-IR (thin film): ν_{max} (cm^{-1}) = 3186, 2923, 1680, 1654, 1504, 1413, 1376, 1284, 762.

(4*R*,5*S*)-1-Benzyl-5-nitro-4-(trifluoromethyl)piperidin-2-one (**4b**)

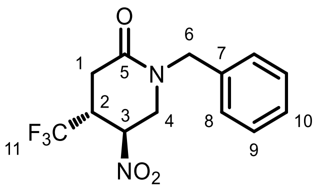

Product **4b** was prepared according to **Procedure 22**, and was obtained as a brown oil.

yield: 150 mg (0.499 mmol, 99%).

d.r.: 95:5.

e.r.: 93:7 [HPLC CHIRALPAK® AD-H, hexane/IPA = 90/10, 1 mL/min, λ = 220 nm, $t_{(minor)}$ = 14.6 min, $t_{(major)}$ = 20.6 min].

^{1}H NMR (400 MHz, $CDCl_3$) δ 7.39 – 7.29 (m, 3H, ***C8*** – **C10**), 7.25 – 7.19 (m, 2H ***C8*** – **C10**), 4.86 – 4.79 (m, 2H, ***C3***, ***C6***), 4.36 (d, *J* = 14.7 Hz, 1H, ***C6***), 3.96 (dd, *J* = 14.6, 3.9 Hz, 1H), 3.80 – 3.71 (m, 1H, ***C2***), 3.68 (dd, *J* = 14.6, 4.6 Hz, 1H, ***C4***), 2.88 (dd, *J* = 16.7, 7.1 Hz, 1H, ***C4***), 2.63 (dd, *J* = 16.7, 8.2 Hz, 1H, ***C1***).

^{13}C NMR (101 MHz, $CDCl_3$) δ 166.4 (***C5***), 135.5 (***C3***), 129.0 (***C8***), 128.3 (***C9***), 128.3 (***C10***), 125.4 (q, *J* = 279.4 Hz, ***C11***), 78.5 (q, *J* = 2.1 Hz ,***C3***), 50.1 (***C6***), 47.2 (***C4***), 40.1 (q, *J* = 29.1 Hz, ***C2***), 28.9 (q, *J* = 2.3 Hz, ***C1***).

^{19}F NMR (376 MHz, $CDCl_3$) δ -71.8 (d, *J* = 9.1 Hz).

HRMS (ESI+, m/Z): exact mass calculated for $C_{13}H_{14}F_3N_2O_3$ $[M+H]^+$ 303.0951, found 303.0954.

$[\alpha]_D^{25}$ = -50.6 (*c* = 0.93, $CHCl_3$).

FT-IR (thin film): ν_{max} (cm^{-1}) = 1670, 1565, 1261, 1180, 1078, 700, 658.

(4*S*,5*S*)-1-benzyl-4-(4-fluorophenyl)-5-nitropiperidin-2-one (**4c**)

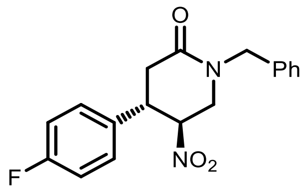

Product **4c** was prepared according to **Procedure 22**, and was obtained as a colorless oil.

yield: 29.6 mg (0.090 mmol, 72%).

d.r.: 95:5.

e.r.: 95:5 (major diastereomer); 96:4 (minor diastereomer) [HPLC CHIRALPAK® AD-H, hexane/IPA = 85/15, 1 mL/min, λ = 220 nm, *major diastereomer:* $t_{(minor)}$ = 16.5 min, $t_{(major)}$ = 21.1 min; *minor diastereomer:* $t_{(major)}$ = 23.2 min, $t_{(minor)}$ = 29.3 min].

^{1}H NMR (400 MHz, $CDCl_3$) δ 7.32 – 7.18 (m, 5H), 7.15 – 7.05 (m, 2H), 6.96 (ddt, *J* = 8.6, 6.5, 2.6 Hz, 2H), 4.82 – 4.71 (m, 2H), 4.44 (d, *J* = 14.5 Hz, 1H), 3.83 – 3.69 (m, 2H), 3.49 (dd, *J* = 13.0, 5.1 Hz, 1H), 2.84 (dd, *J* = 17.9, 6.1 Hz, 1H), 2.67 (dd, *J* = 17.9, 9.0 Hz, 1H).

^{13}C NMR (101 MHz, $CDCl_3$) δ 167.3, 162.5 (d, *J* = 247.9 Hz), 135.7, 133.6 (d, *J* = 3.4 Hz), 129.0, 128.8 (d, *J* = 8.2 Hz), 128.5, 128.2, 116.4 (d, *J* = 21.6 Hz), 84.9, 50.3, 47.4, 41.8, 35.9.

^{19}F NMR (376 MHz, $CDCl_3$) δ -113.2 (tt, *J* = 8.4, 5.1 Hz).

$[\alpha]_D^{25}$ = -31.8 (*c* = 2.15, $CHCl_3$).

Analytical data were consistent with those reported in the literature.[18]

(*R*)-4-Methylpyrrolidin-2-one (**5a**)

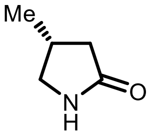

Product **5a** was prepared according to **Procedure 23**, and was obtained as a colorless solid.

yield: 26.9 mg (0.272 mmol, 54%).

e.r.: 98:2 [SFC CHIRALPAK® IG, from 1% to 20% MeOH in 7 min, then from 20% to 50% in 1 min, 1 mL/min, λ = 220 nm, $t_{(minor)}$ = 4.7 min, $t_{(major)}$ = 4.9 min].

^{1}H NMR (400 MHz, $CDCl_3$) δ 6.55 (s, 1H), 3.56 – 3.41 (m, 1H), 3.01 – 2.86 (m, 1H), 2.60 – 2.39 (m, 2H), 1.93 (dd, *J* = 16.5, 7.1 Hz, 1H), 1.12 (d, *J* = 6.7 Hz, 3H).

^{13}C NMR (101 MHz, $CDCl_3$) δ 178.8, 49.7, 38.6, 29.6, 19.7.

$[\alpha]_D^{25}$ = +11.0 (*c* = 0.48, $CHCl_3$). {lit.: $[\alpha]_D^{25}$ = +18.1 (*c* = 1.00, $CHCl_3$)}[19]

Analytical data were consistent with those reported in the literature.[19,20]

(*R*)-4-(Trifluoromethyl)pyrrolidin-2-one (**5b**)

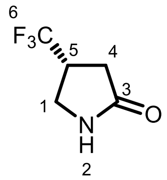

Product **5b** was prepared according to modified **Procedure 23**, and was obtained as a colorless solid after silica gel chromatography (CH_2Cl_2 : MeOH 0% to 10%).

yield: 33.2 mg (0.239 mmol, 80%).

e.r.: 92.5:7.5 [HPLC CHIRALPAK® AD-H, hexane/IPA = 90/10, 1 mL/min, λ = 220 nm, $t_{(major)}$ = 6.5 min, $t_{(minor)}$ = 7.1 min].

^{1}H NMR (400 MHz, $CDCl_3$) δ 7.14 (s, 1H, ***N2***), 3.66 – 3.56 (m, 1H, ***C1***), 3.50 (dd, *J* = 10.5, 6.0 Hz, 1H, ***C1***), 3.27 – 3.08 (m, 1H, ***C5***), 2.64 – 2.41 (m, 2H, ***C4***).

^{13}C NMR (101 MHz, $CDCl_3$) δ 175.7 (***C3***), 126.6 (q, *J* = 276.8 Hz, ***C6***), 41.5 (q, *J* = 3.4 Hz, ***C1***), 38.1 (q, *J* = 29.9 Hz, ***C56***), 30.2 (d, *J* = 2.5 Hz, ***C4***).

^{19}F NMR (376 MHz, $CDCl_3$) δ -73.1 (d, *J* = 8.9 Hz).

HRMS (ESI+, m/Z): exact mass calculated for $C_5H_7F_3NO$ $[M+H]^+$ 154.0474, found 154.0477.

m. p.: 68 – 70 ºC

$[\alpha]_D^{25}$ = +6.1 (*c* = 0.86, $CHCl_3$).

FT-IR (thin film): ν_{max} (cm^{-1}) = 3244, 1693, 1666, 1284, 1164, 1124, 1058, 673.

(*S*)-4-(3-(Cyclopentyloxy)-4-methoxyphenyl)pyrrolidin-2-one ((*S*)-rolipram, **5c**)

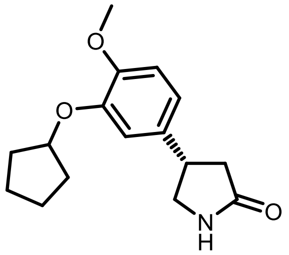

Product **5c** ((*S*)-rolipram) was prepared according to **Procedure 23**, and was obtained as a colorless solid.

yield: 20.9 mg (0.08 mmol, 80%).

e.r.: 98:2 [SFC CHIRALPAK® IG, from 1% to 20% MeOH in 7 min, then from 20% to 50% in 1 min, 1 mL/min, λ = 220 nm, $t_{(minor)}$ = 4.7 min, $t_{(major)}$ = 4.8 min].

^{1}H NMR (400 MHz, $CDCl_3$) δ 6.86 – 6.80 (m, 1H), 6.80 – 6.74 (m, 2H), 6.39 (s, 1H), 4.80 – 4.71 (m, 1H), 3.83 (s, 3H), 3.79 – 3.71 (m, 1H), 3.67 – 3.55 (m, 1H), 3.43 – 3.31 (m, 1H), 2.75 – 2.65 (m, 1H), 2.55 – 2.39 (m, 1H), 1.97 – 1.76 (m, 5H), 1.67 – 1.53 (m, 2H).

^{13}C NMR (101 MHz, $CDCl_3$) δ 177.8, 149.4, 148.1, 134.7, 118.9, 114.0, 112.4, 80.8, 56.3, 49.9, 40.1, 38.2, 32.9, 24.1.

$[\alpha]_D^{25}$ = +31.3 (*c* = 0.96, $CHCl_3$). {lit.: $[\alpha]_D^{25}$ = +27.4 (*c* = 0.14, $CHCl_3$)}[21]

Analytical data were consistent with those reported in the literature.[21]

[ToC]

(*S*)-4-(p-tolyl)pyrrolidin-2-one (**5d**)

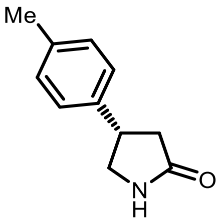

Product **5d** was prepared according to **Procedure 23**, and was obtained as a colorless solid.

yield: 22.9 mg (0.131 mmol, 93%).

e.r.: 96.5:3.5 [HPLC CHIRALPAK® AD-H, hexane/IPA = 97/3, 1 mL/min, λ = 220 nm, $t_{(minor)}$ = 25.9 min, $t_{(major)}$ = 27.0 min].

^{1}H NMR (400 MHz, $CDCl_3$) δ 7.19 – 7.10 (m, 4H), 6.74 (s, 1H), 3.81 – 3.74 (m, 1H), 3.71 – 3.61 (m, 1H), 3.40 (dd, *J* = 9.4, 7.3 Hz, 1H), 2.71 (dd, *J* = 16.9, 8.8 Hz, 1H), 2.49 (dd, *J* = 16.8, 8.9 Hz, 1H), 2.34 (s, 3H).

^{13}C NMR (101 MHz, $CDCl_3$) δ 178.0, 139.2, 136.9, 129.6, 126.8, 49.8, 40.1, 38.2, 21.1.

$[\alpha]_D^{25}$ = +40.6 (*c* = 1.75, $CHCl_3$). {lit.: $[\alpha]_D^{25}$ = +30.3 (*c* = 1.04, $CHCl_3$)}[22]

Analytical data were consistent with those reported in the literature.[23]

[ToC]

NMR Spectra
Catalyst Precursors
S2

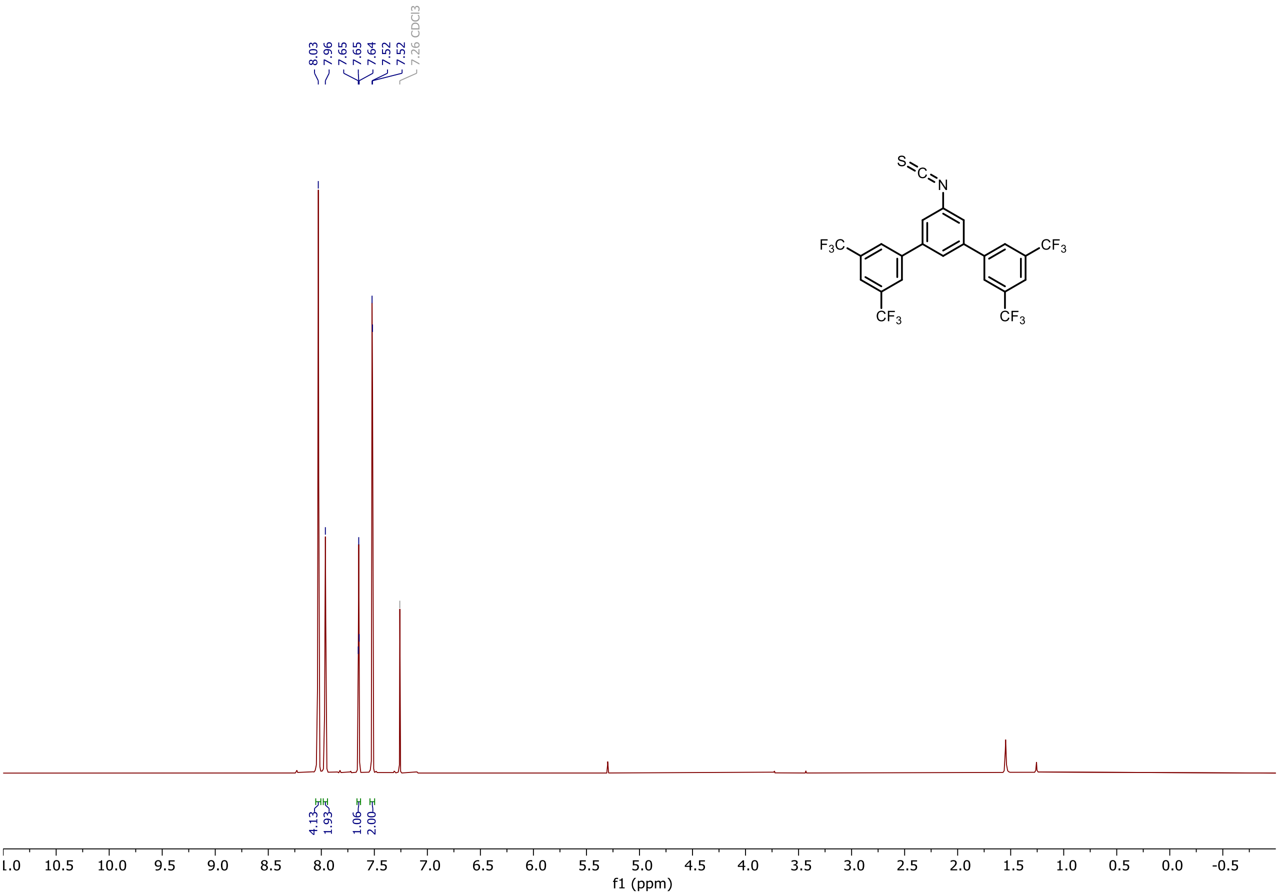

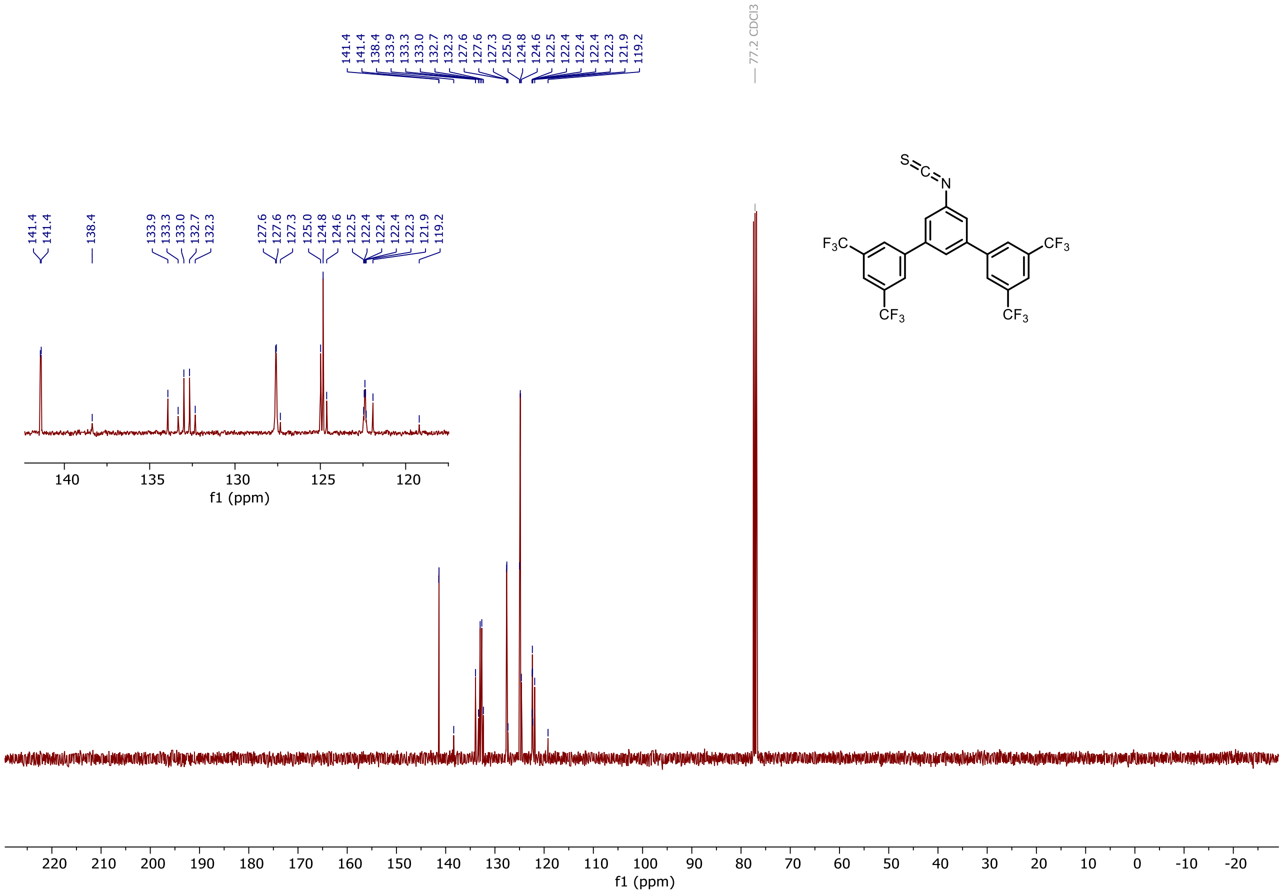

[ToC]

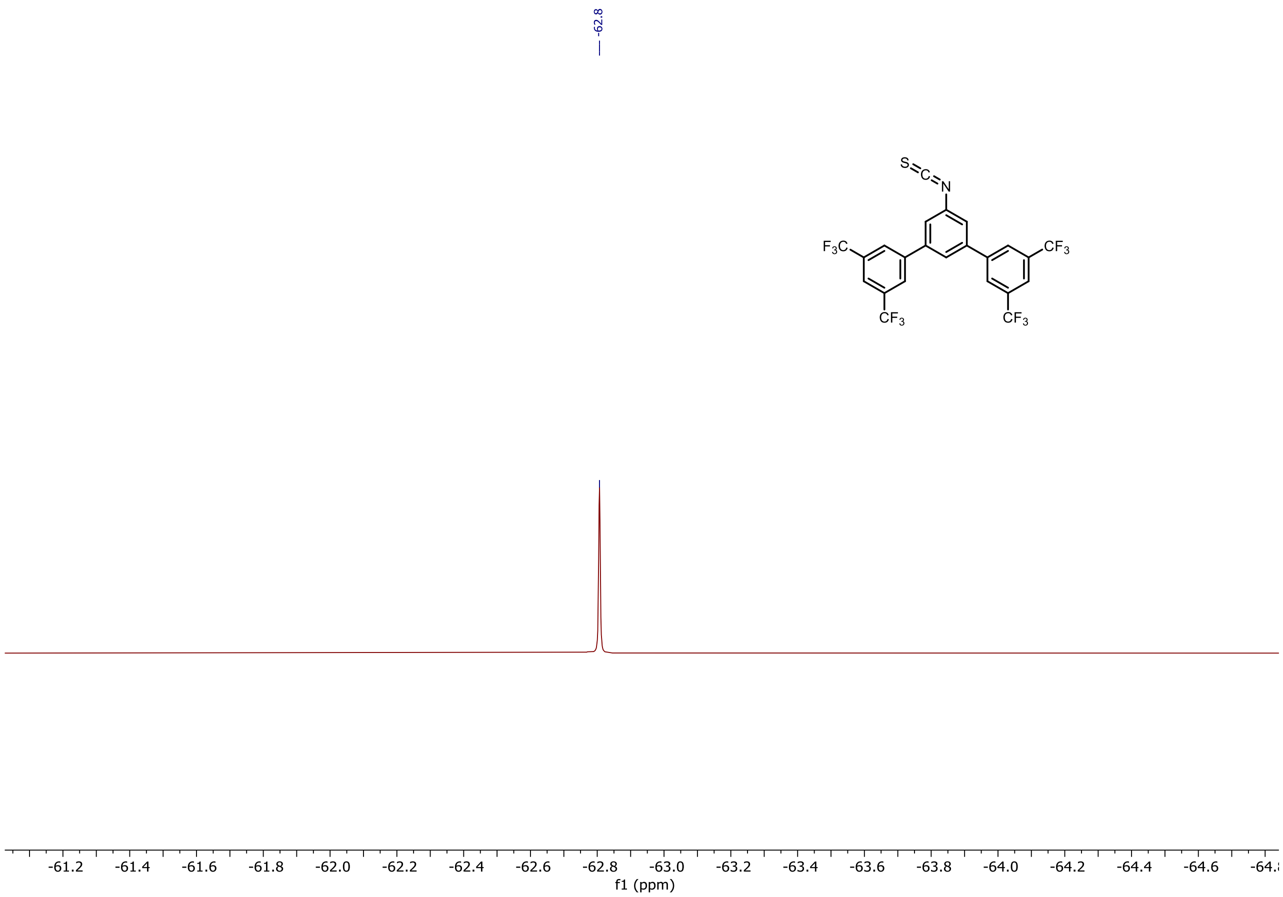
-62.8
-61.2
-61.4
-61.6
-61.8
-62.0
-62.2
-62.4
-62.6
-62.8
-63.0
-63.2
-63.4
-63.6
-63.8
-64.0
-64.2
-64.4
-64.6
f1 (ppm)
S
C
N
F3C
CF3
CF3
CF3

S5

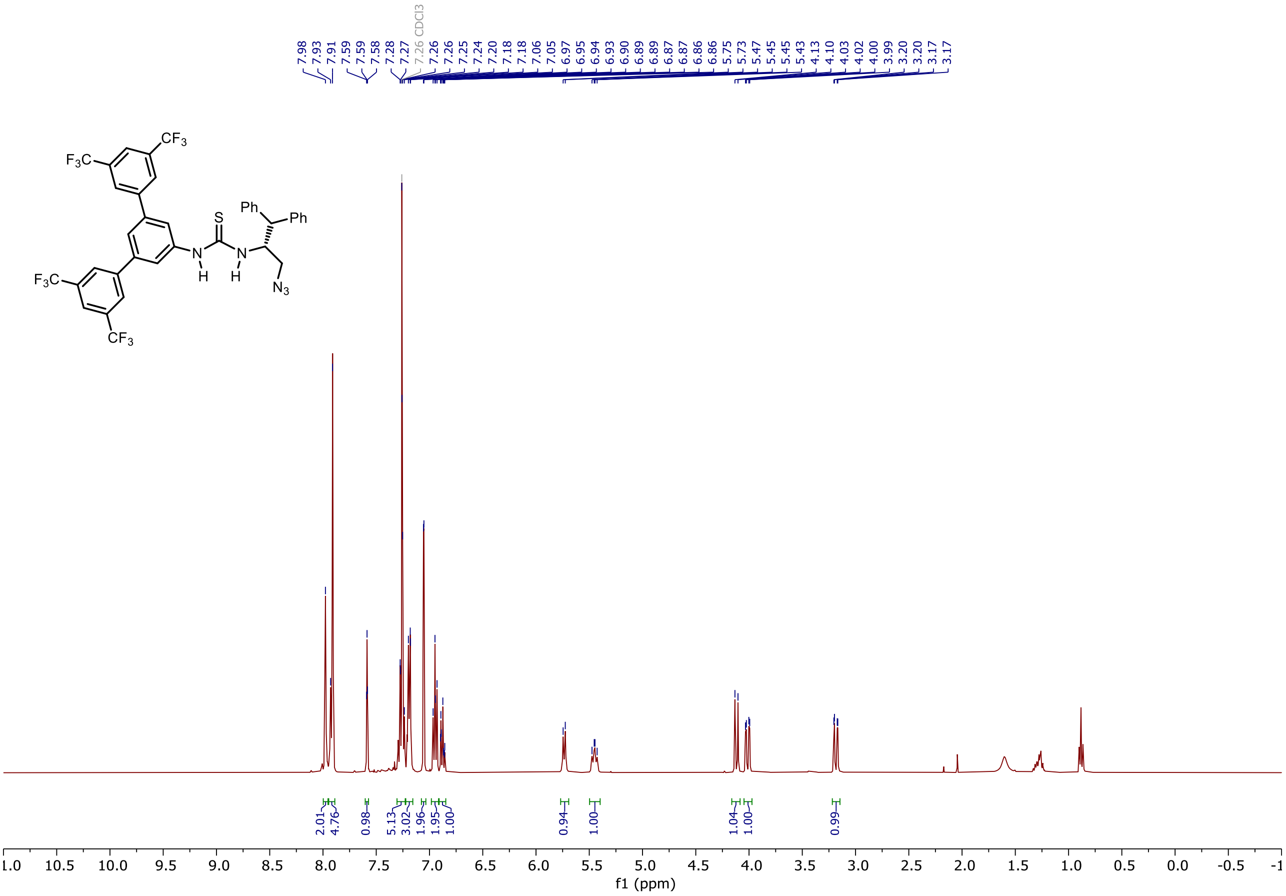
7.98
7.93
7.91
7.59
7.59
7.58
7.28
7.27
7.26 CDCl3
7.26
7.26
7.25
7.24
7.20
7.18
7.18
7.06
7.05
6.97
6.95
6.94
6.93
6.90
6.89
6.89
6.87
6.87
6.86
6.86
5.75
5.73
5.47
5.45
5.45
5.43
4.13
4.10
4.03
4.02
4.00
3.99
3.20
3.20
3.17
3.17
CF3
F3C
Ph
Ph
S
N
H
N
H
F3C
CF3
N3
2.01
4.76
0.98
5.13
3.02
1.96
1.95
1.00
0.94
1.00
1.04
1.00
0.99
f1 (ppm)

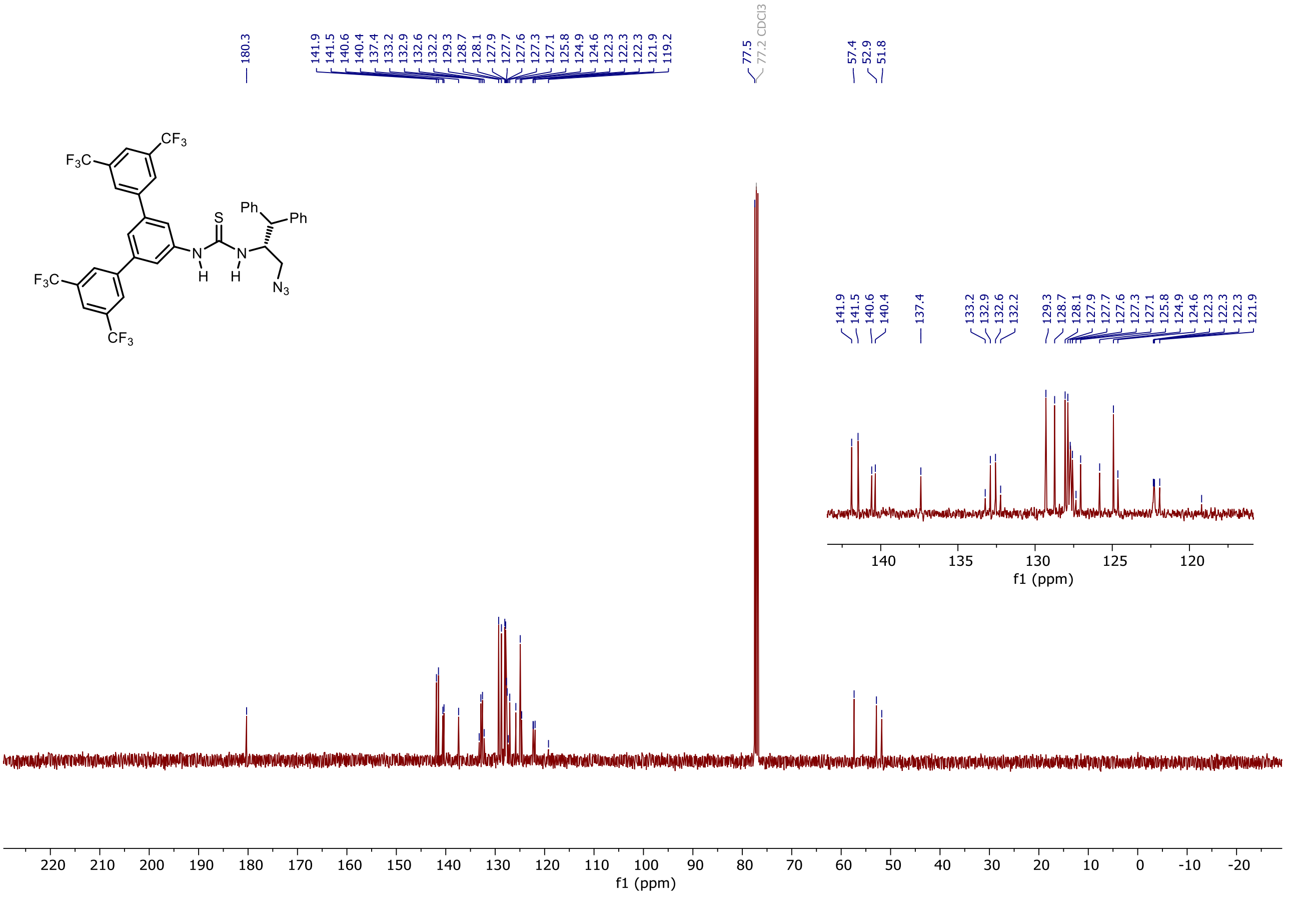
180.3
141.9
141.5
140.6
140.4
137.4
133.2
132.9
132.6
132.2
129.3
128.7
128.1
127.9
127.7
127.6
127.3
127.1
125.8
124.9
124.6
122.3
122.3
121.9
119.2
77.5
77.2 CDCl3
57.4
52.9
51.8
CF3
F3C
Ph
Ph
S
N
H
N
H
F3C
CF3
N3
f1 (ppm)

[ToC]

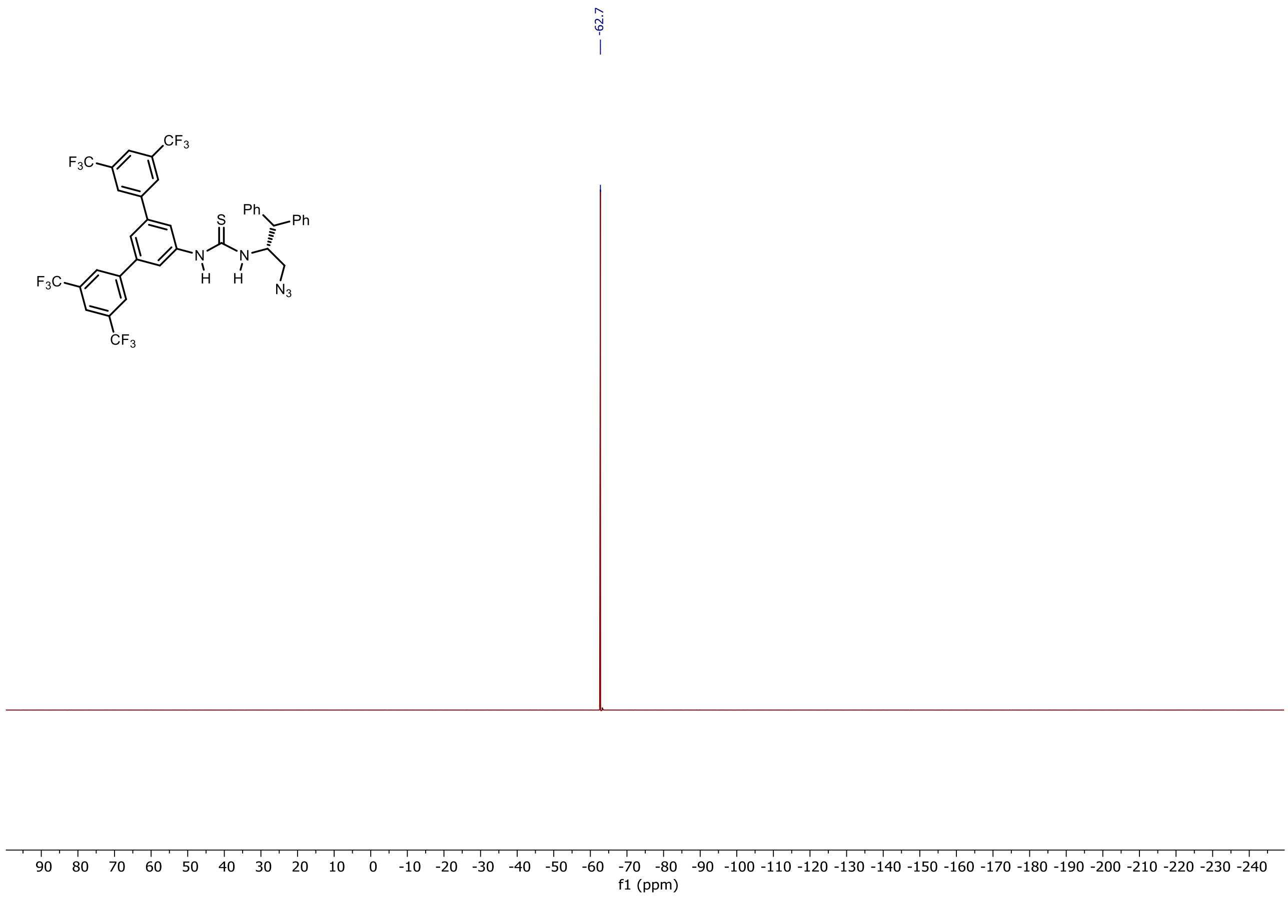

-62.7
CF3
F3C
Ph
S
Ph
N
N
H
H
N3
F3C
CF3
90 80 70 60 50 40 30 20 10 0 -10 -20 -30 -40 -50 -60 -70 -80 -90 -100 -110 -120 -130 -140 -150 -160 -170 -180 -190 -200 -210 -220 -230 -240
f1 (ppm)

α,β-Unsaturated Esters

1s

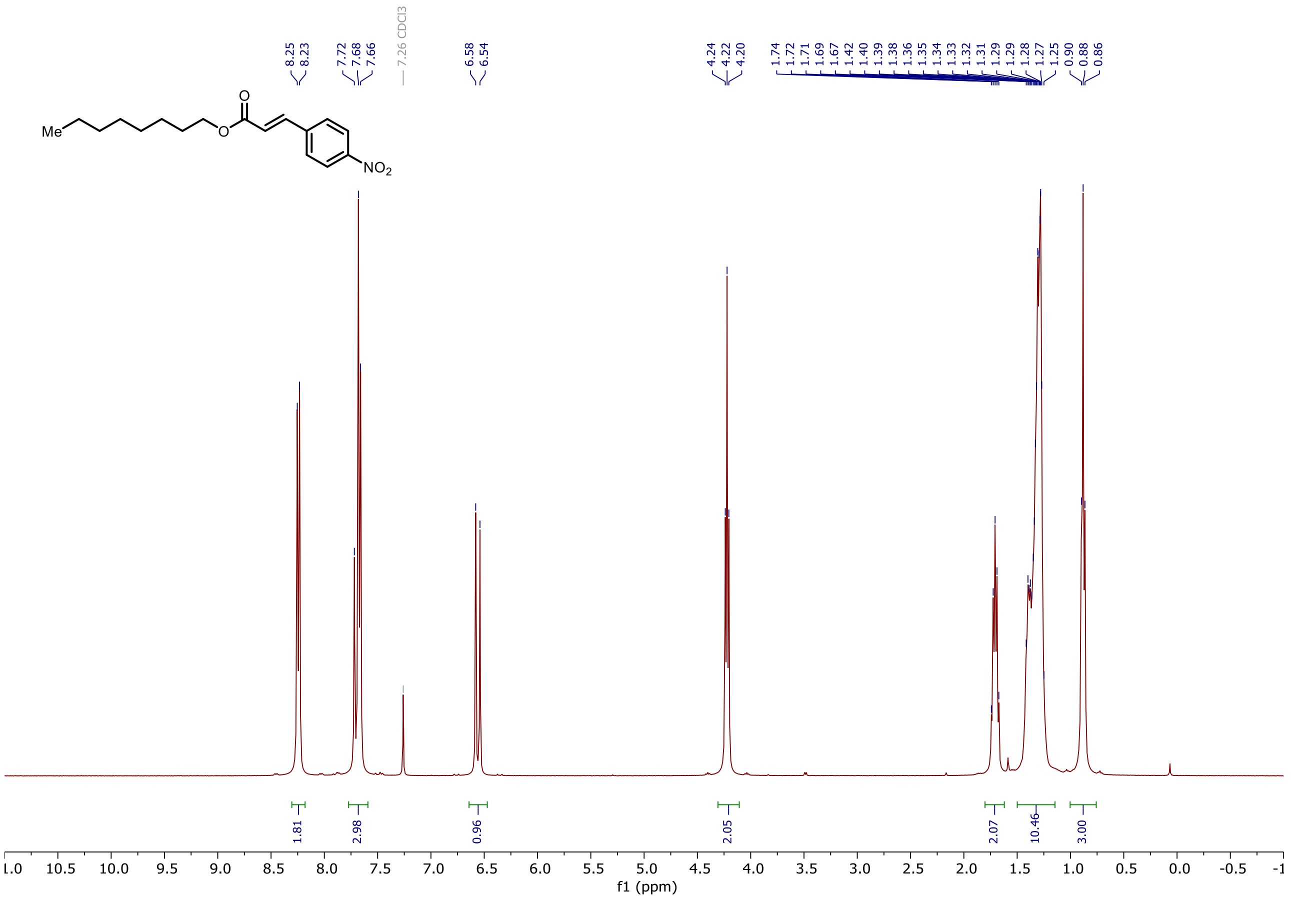

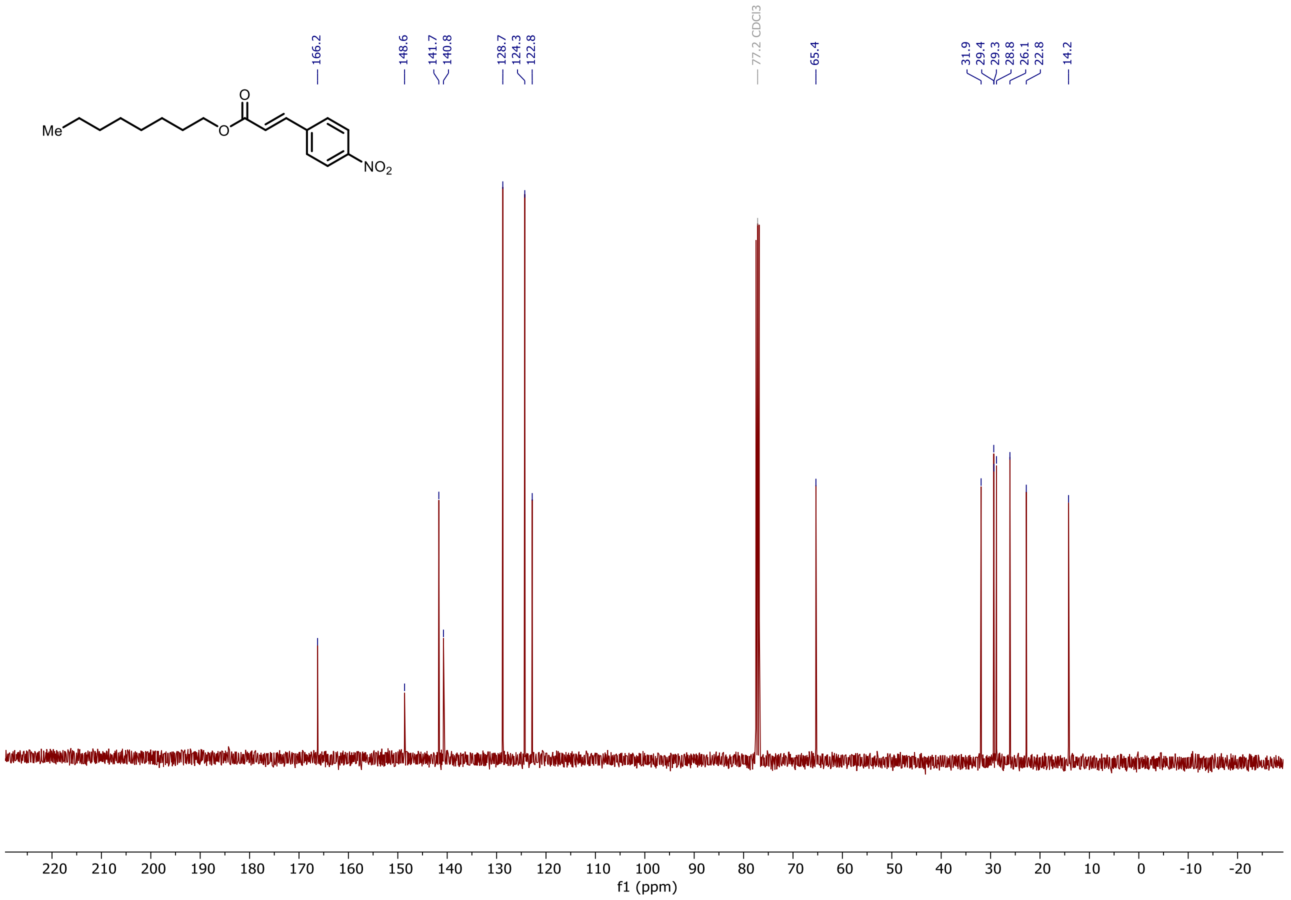

[ToC]

1ac

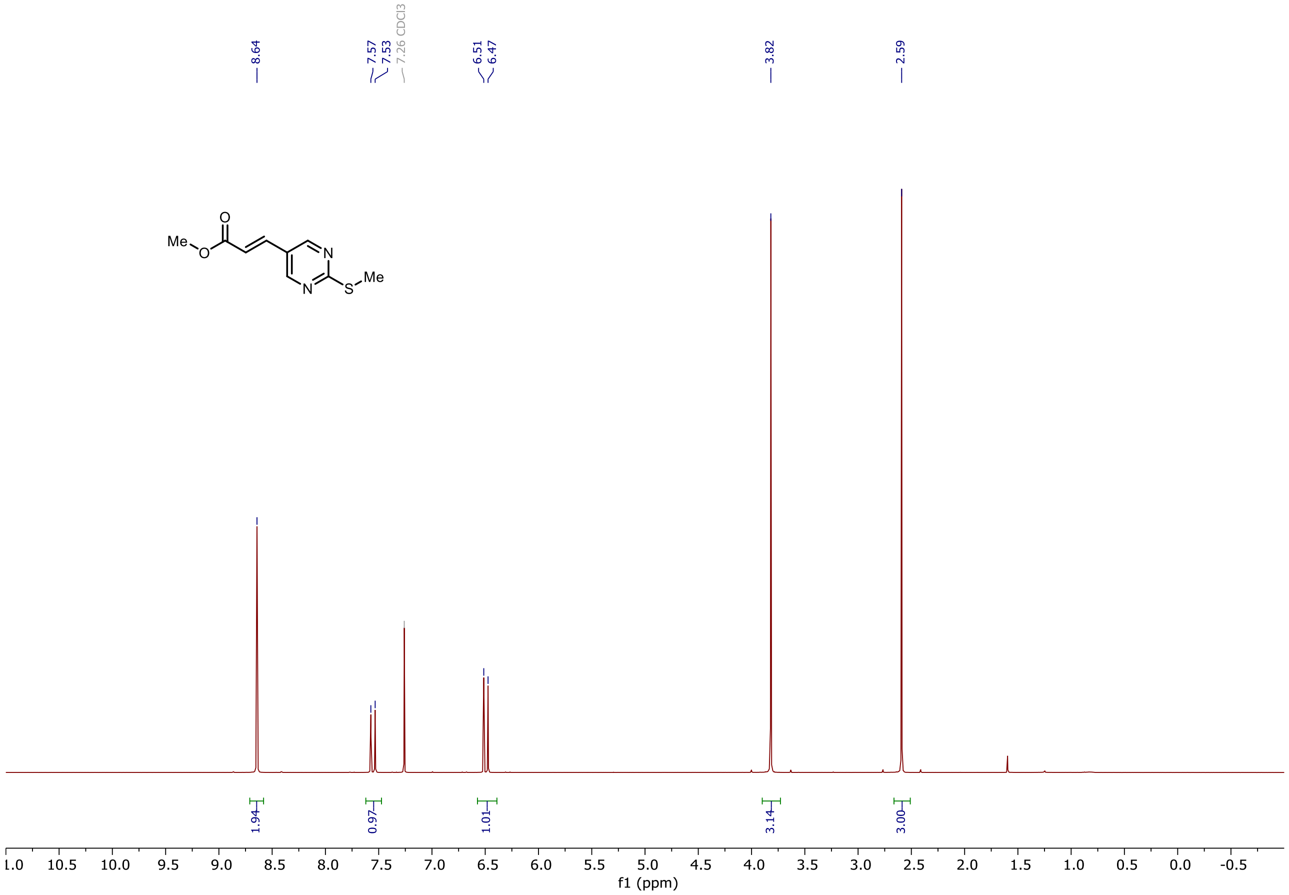
8.64
7.57
7.53
7.26 CDCl3
6.51
6.47
3.82
2.59
Me
O
O
N
N
S
Me
1.94
0.97
1.01
3.14
3.00
f1 (ppm)

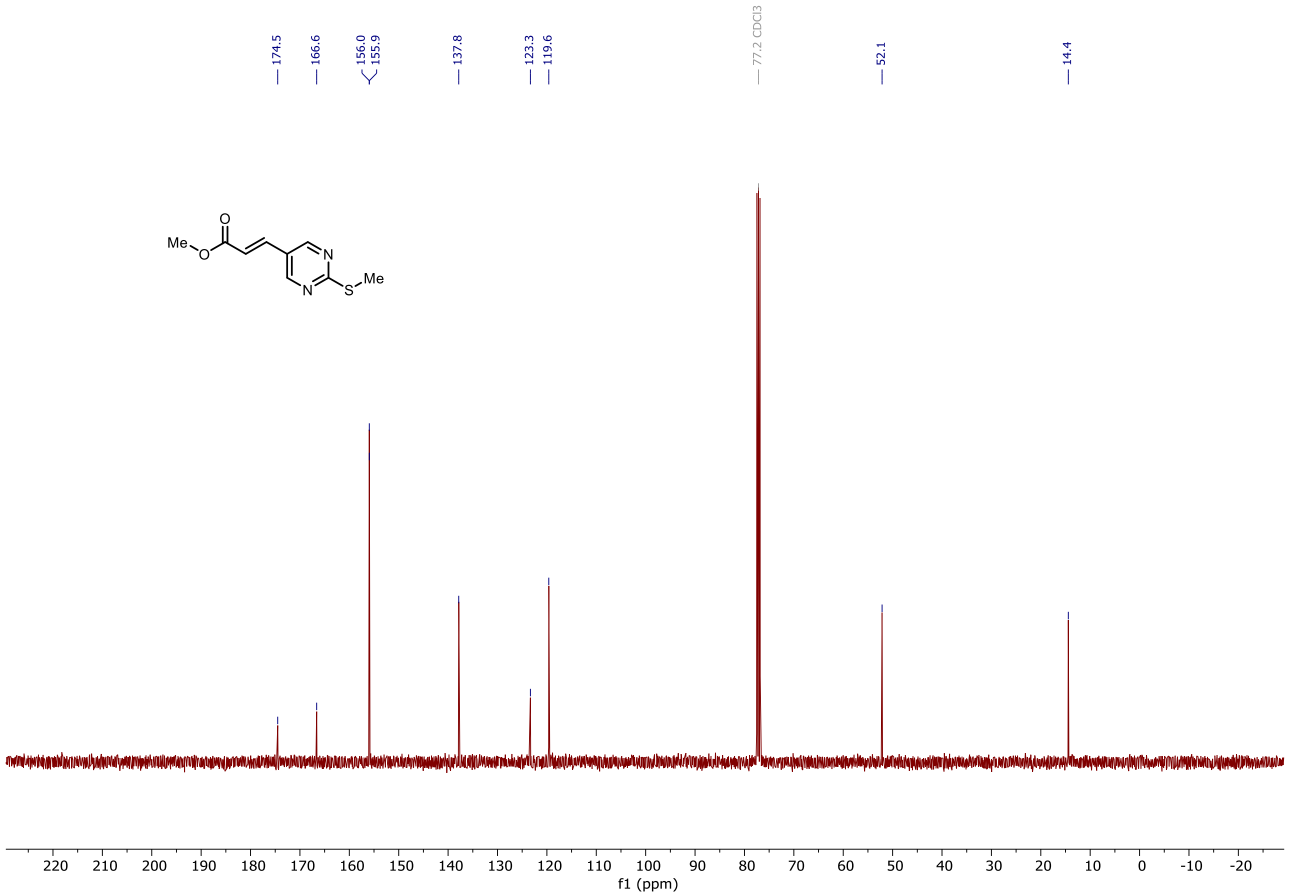
174.5
166.6
156.0
155.9
137.8
123.3
119.6
77.2 CDCl3
52.1
14.4
Me
O
O
N
N
S
Me
f1 (ppm)

[ToC]

1ae

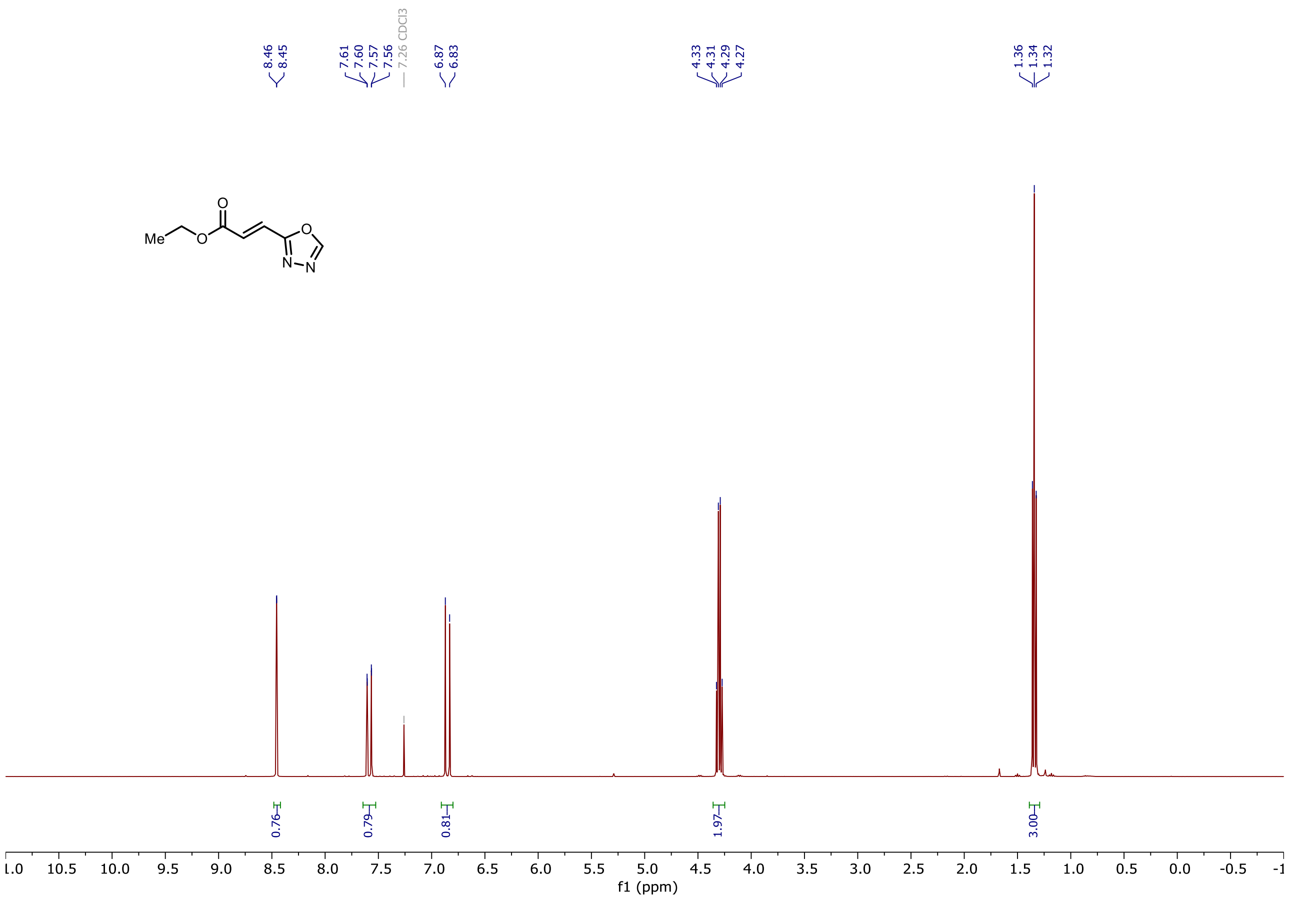

8.46
8.45
7.61
7.60
7.57
7.56
7.26 CDCl3
6.87
6.83
4.33
4.31
4.29
4.27
1.36
1.34
1.32
Me
O
O
O
N
N
0.76
0.79
0.81
1.97
3.00
f1 (ppm)

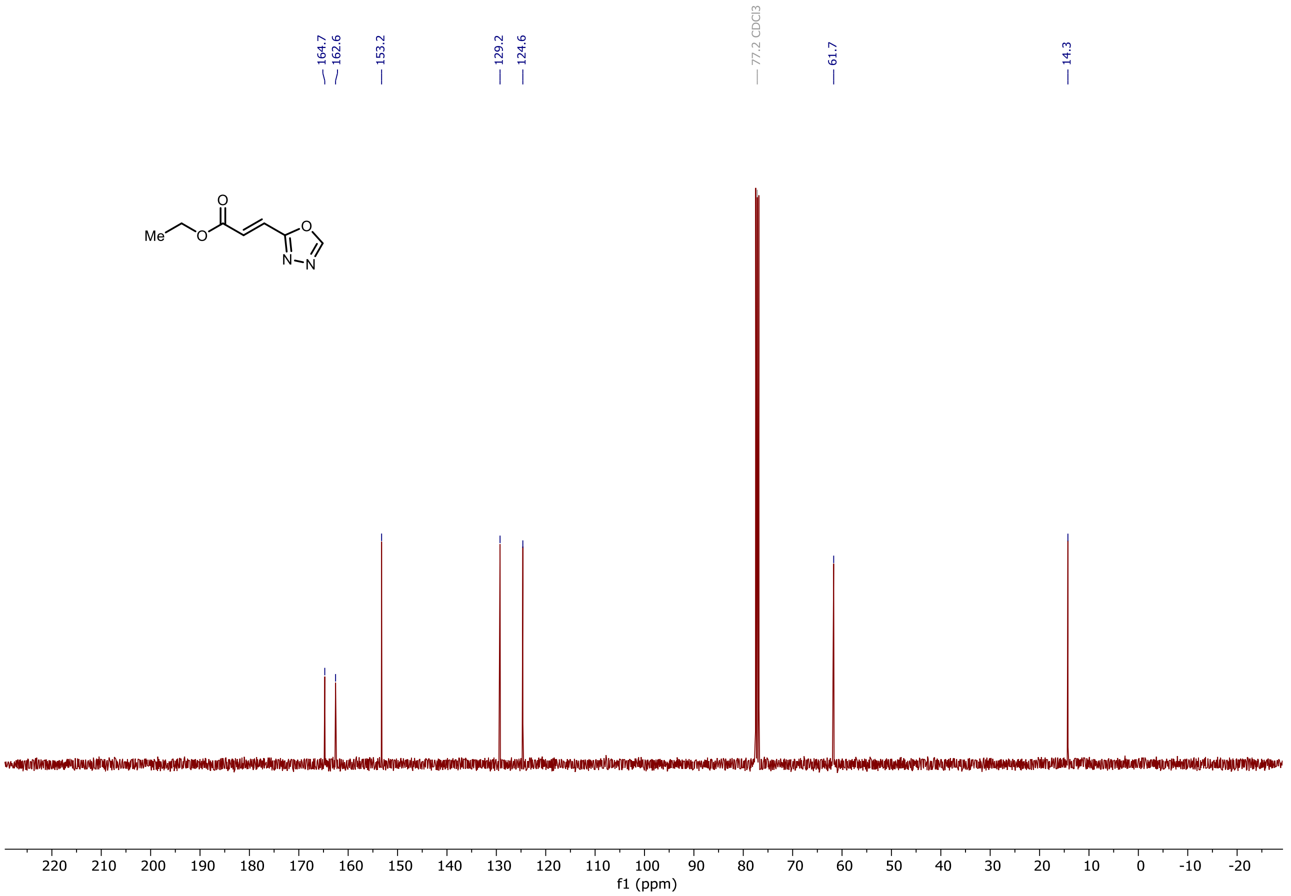

164.7
162.6
153.2
129.2
124.6
77.2 CDCl3
61.7
14.3
Me
O
O
O
N
N
f1 (ppm)

[ToC]

γ-Nitroesters

3a

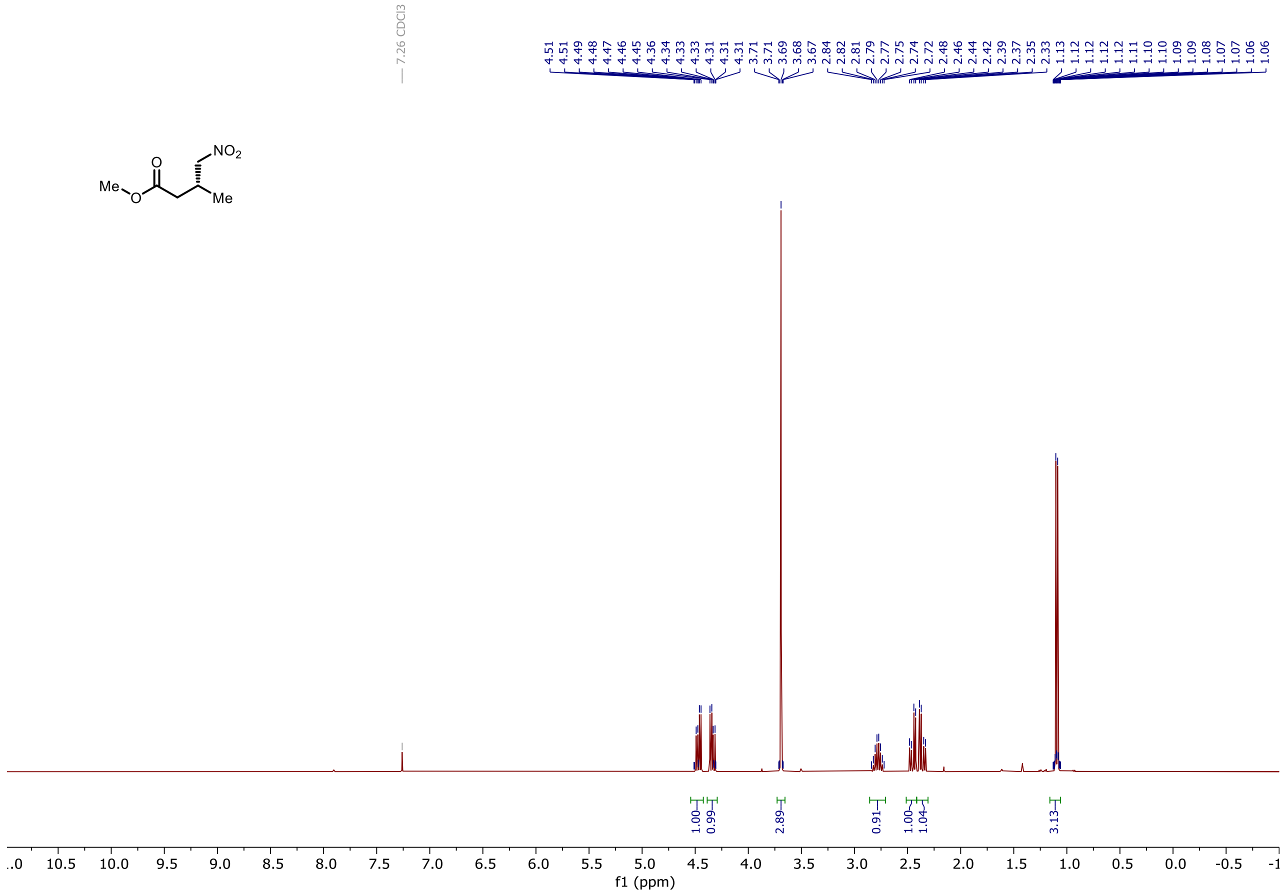

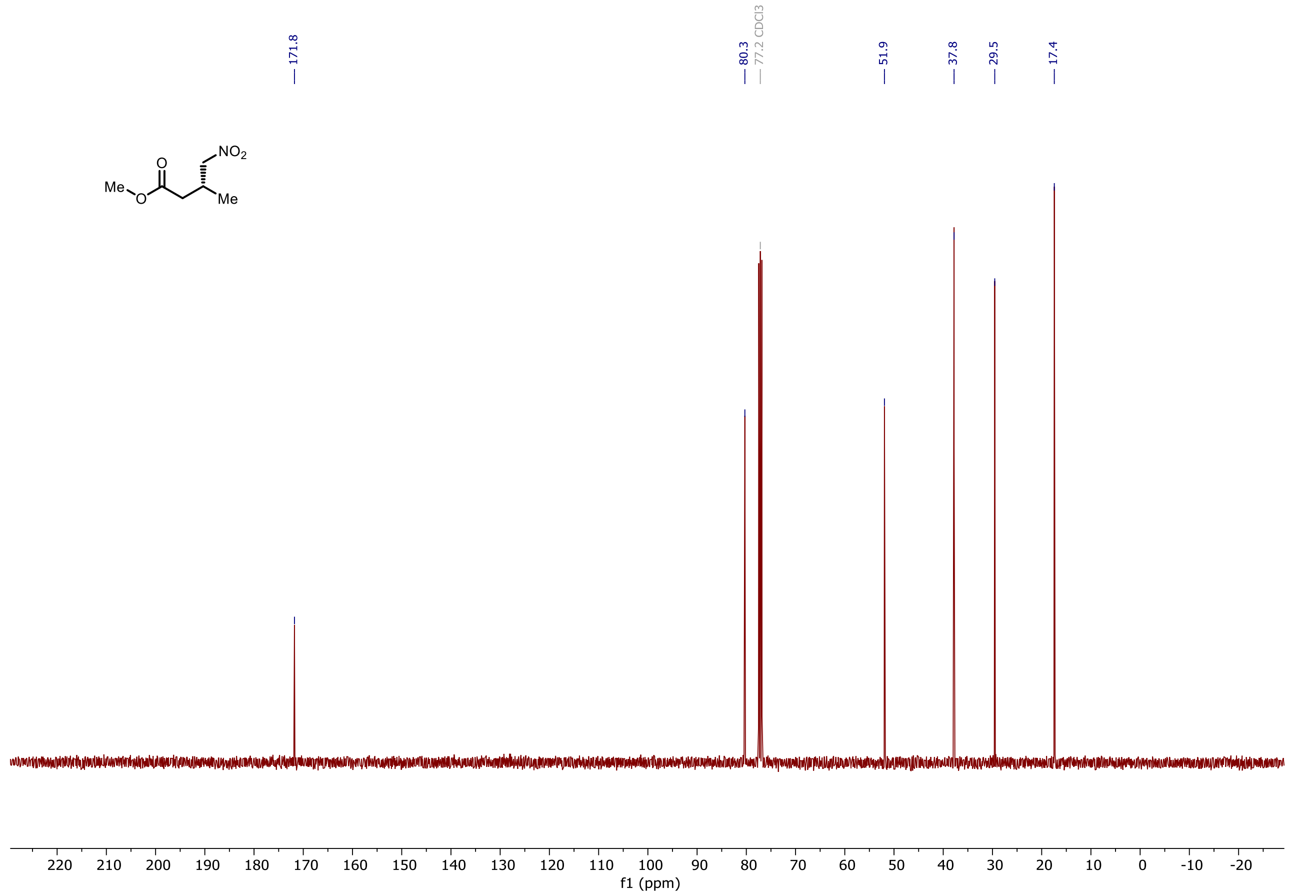

[ToC]

3b

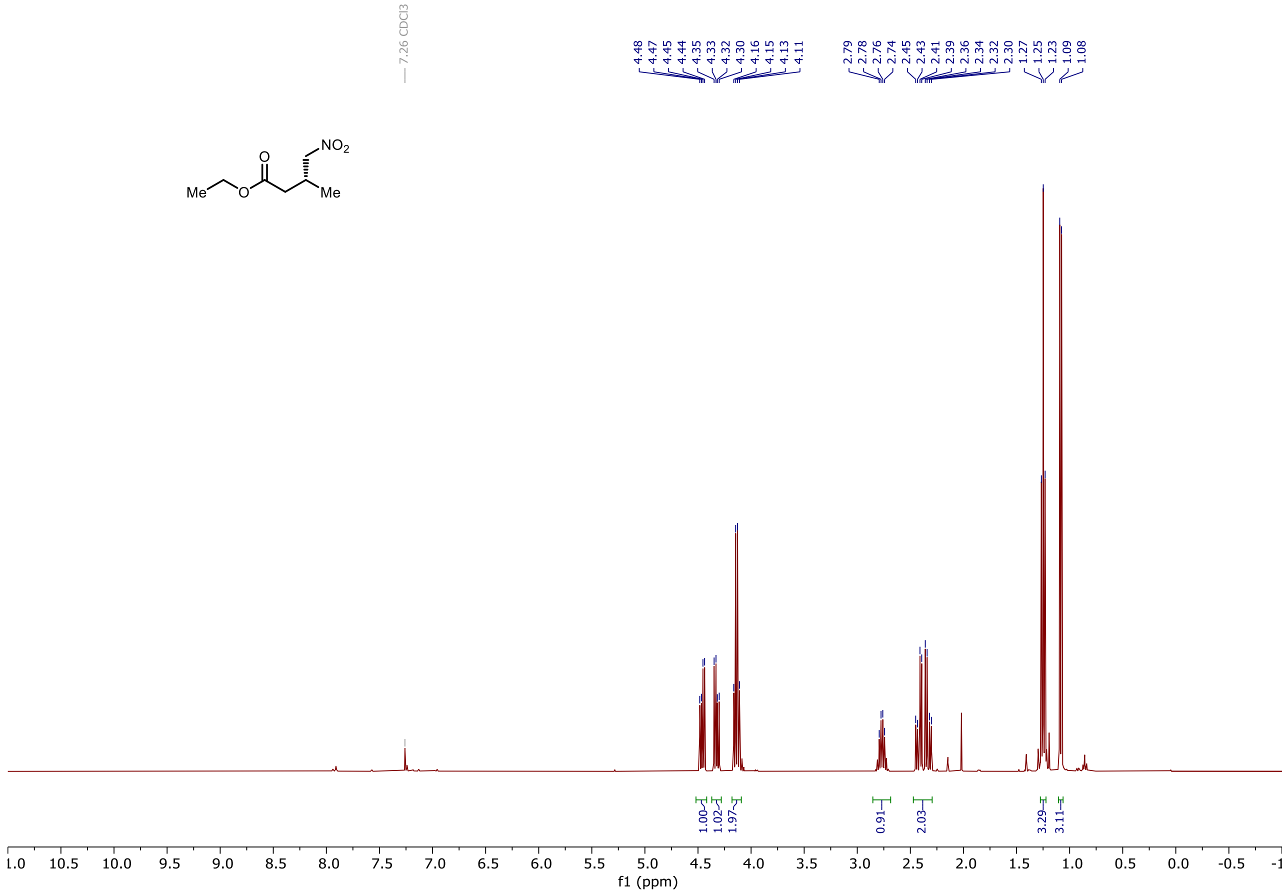
7.26 CDCl3
4.48
4.47
4.45
4.44
4.35
4.33
4.32
4.30
4.16
4.15
4.13
4.11
2.79
2.78
2.76
2.74
2.45
2.43
2.41
2.39
2.36
2.34
2.32
2.30
1.27
1.25
1.23
1.09
1.08
NO2
O
Me
O
Me
1.00
1.02
1.97
0.91
2.03
3.29
3.11
f1 (ppm)

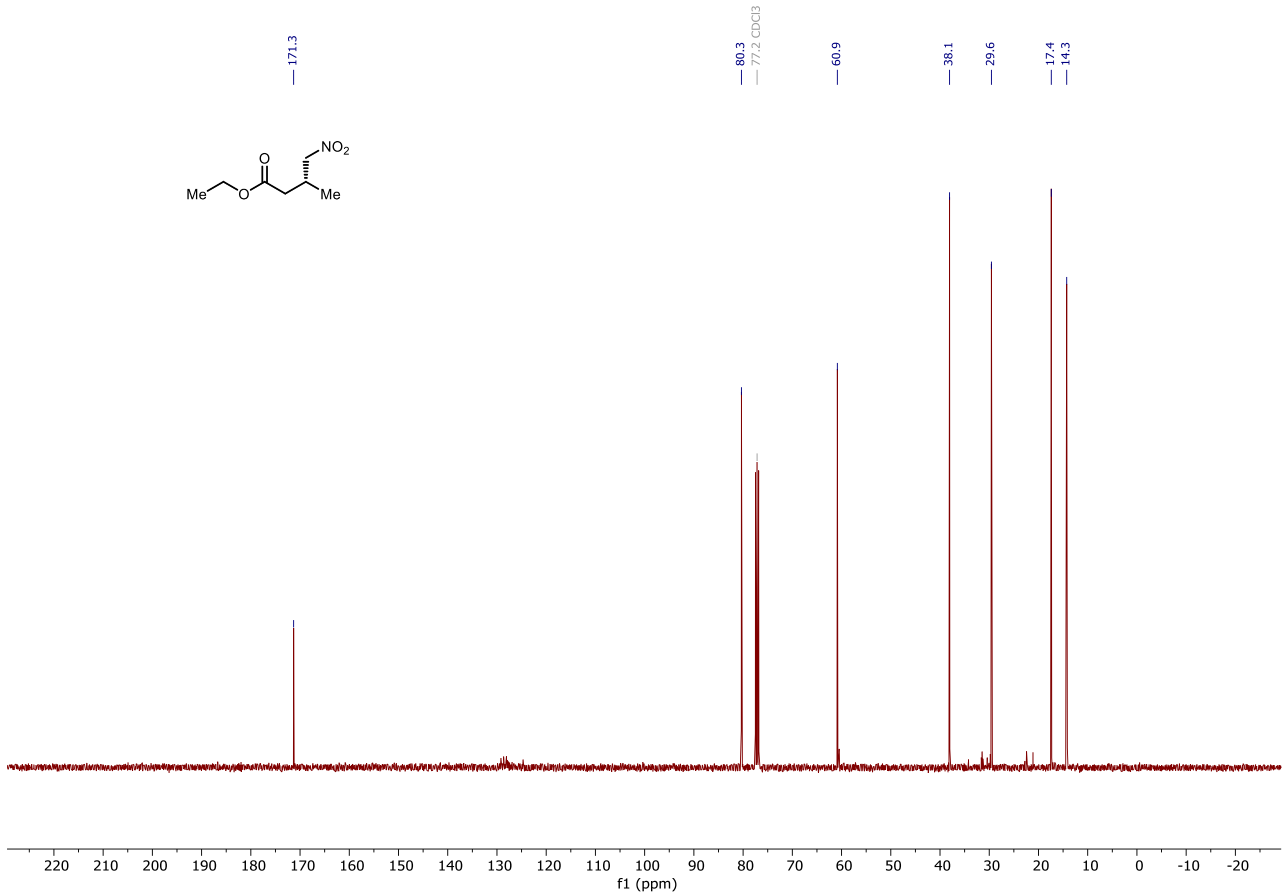
171.3
80.3
77.2 CDCl3
60.9
38.1
29.6
17.4
14.3
NO2
O
Me
O
Me
f1 (ppm)

[ToC]

3c

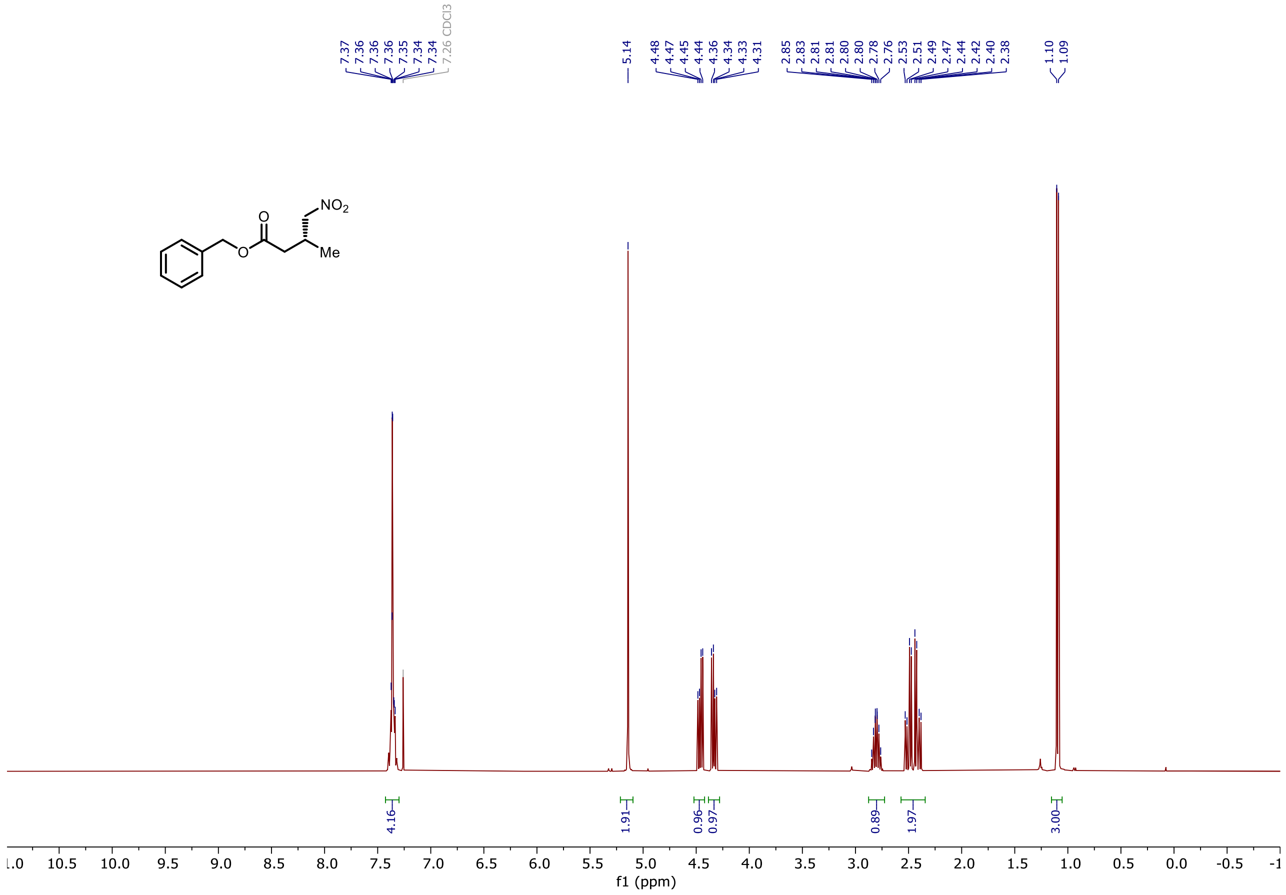

7.37
7.36
7.36
7.36
7.35
7.34
7.34
7.26 CDCl3
5.14
4.48
4.47
4.45
4.44
4.36
4.34
4.33
4.31
2.85
2.83
2.81
2.81
2.80
2.78
2.76
2.53
2.51
2.49
2.47
2.44
2.42
2.40
2.38
1.10
1.09
NO2
O
O
Me
4.16
1.91
0.96
0.97
0.89
1.97
3.00
f1 (ppm)

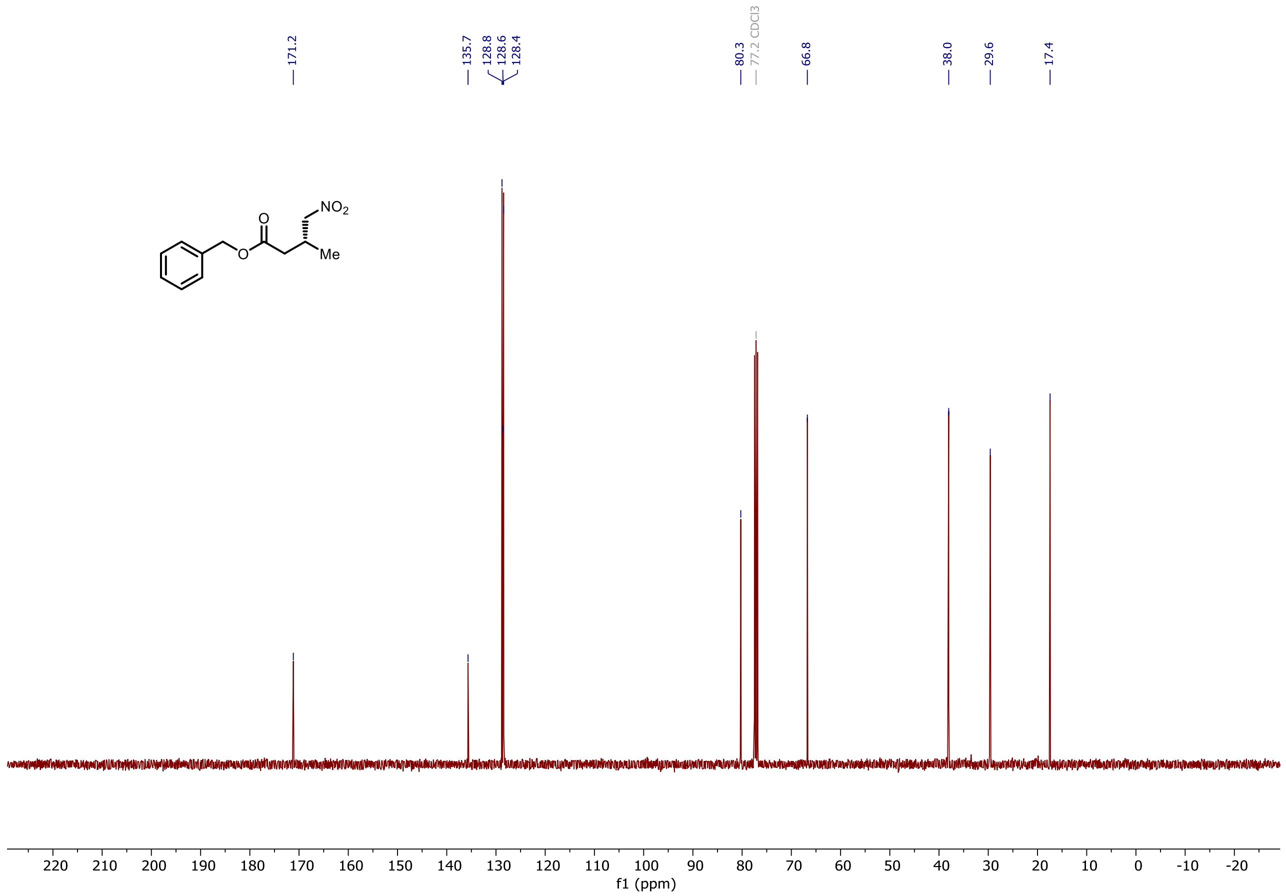

171.2
135.7
128.8
128.6
128.4
80.3
77.2 CDCl3
66.8
38.0
29.6
17.4
NO2
O
O
Me
f1 (ppm)

3e

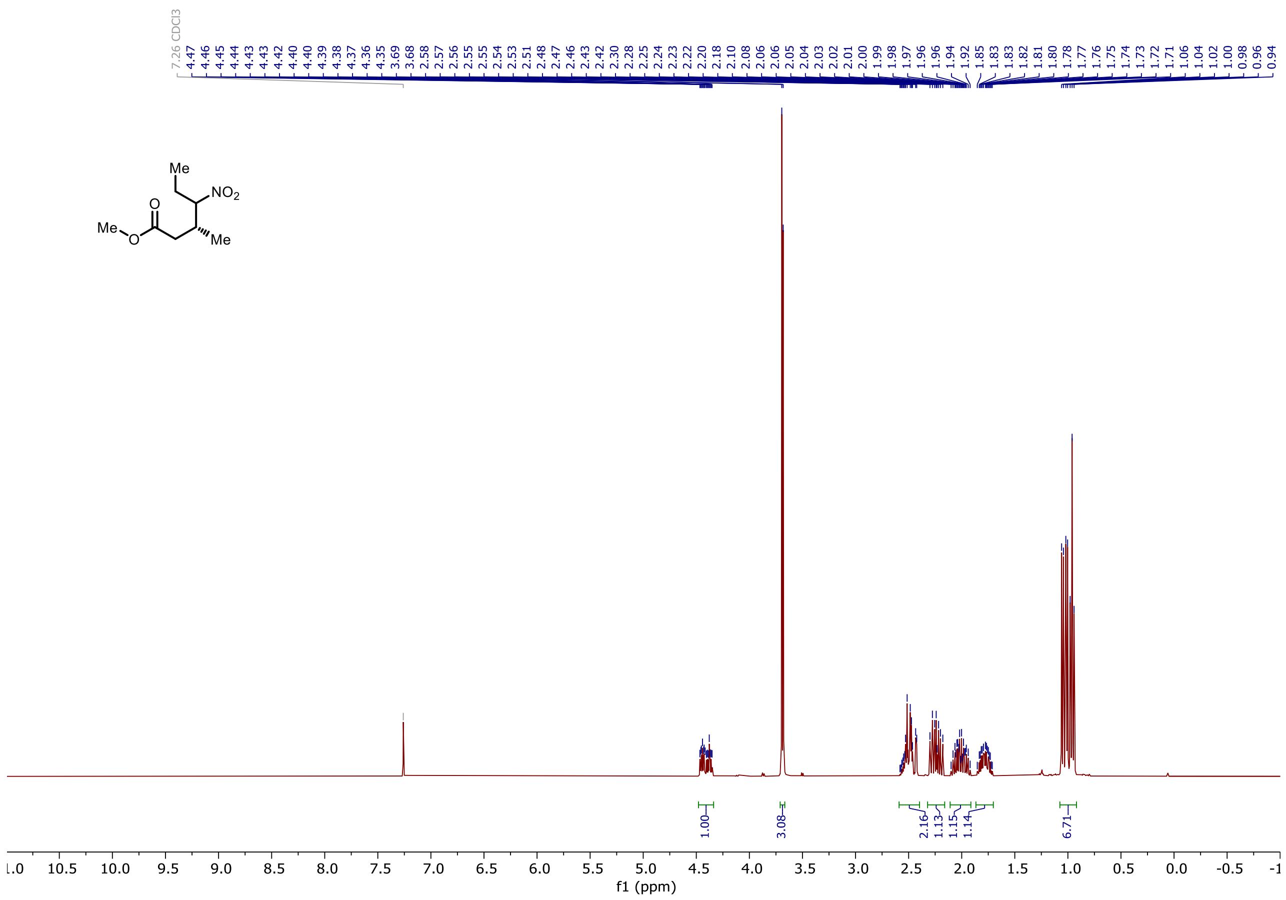
7.26 CDCl3
4.47
4.46
4.45
4.44
4.43
4.43
4.42
4.40
4.40
4.39
4.38
4.37
4.36
4.35
3.69
3.68
2.58
2.57
2.56
2.55
2.55
2.54
2.53
2.51
2.48
2.47
2.46
2.43
2.42
2.30
2.28
2.25
2.24
2.23
2.22
2.20
2.18
2.10
2.08
2.06
2.06
2.06
2.05
2.04
2.03
2.02
2.01
2.00
1.99
1.98
1.97
1.96
1.96
1.94
1.92
1.85
1.83
1.83
1.82
1.81
1.80
1.78
1.77
1.76
1.75
1.74
1.73
1.72
1.71
1.06
1.04
1.02
1.00
0.98
0.96
0.94
Me
NO2
O
Me
O
Me
1.00
3.08
2.16
1.13
1.15
1.14
6.71
f1 (ppm)

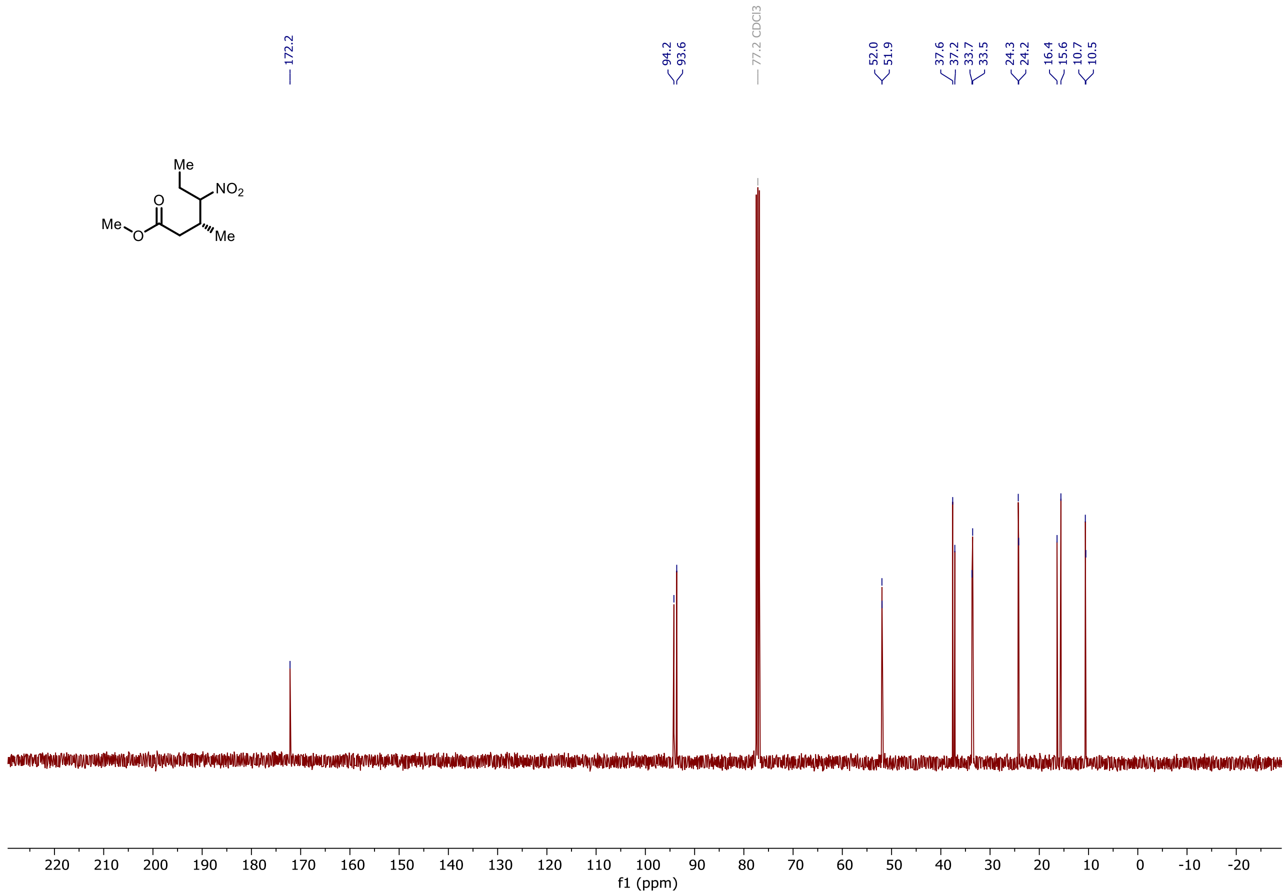
172.2
94.2
93.6
77.2 CDCl3
52.0
51.9
37.6
37.2
33.7
33.5
24.3
24.2
16.4
15.6
10.7
10.5
Me
NO2
O
Me
O
Me
f1 (ppm)

[ToC]

3f

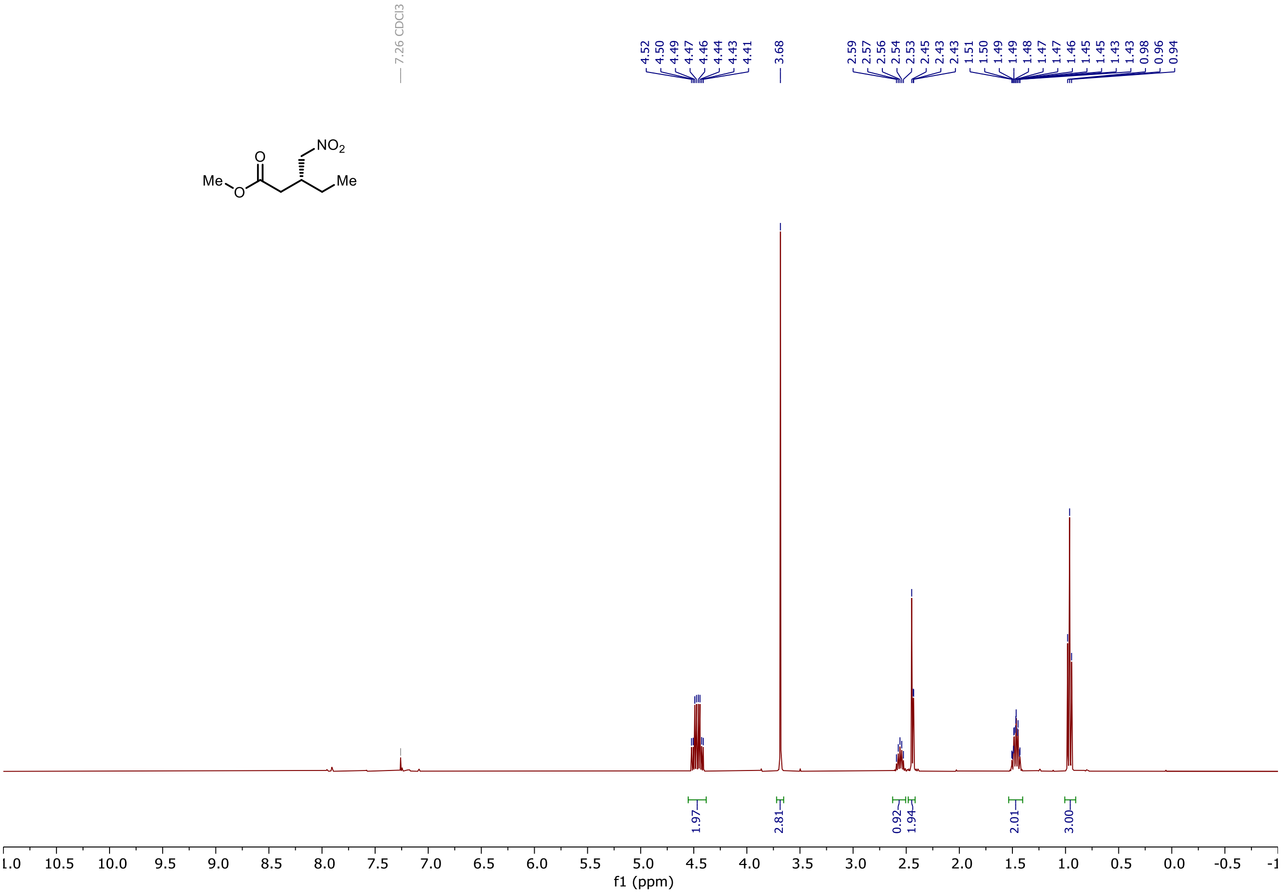
7.26 CDCl3
4.52 4.50 4.49 4.47 4.46 4.44 4.43 4.41
3.68
2.59 2.57 2.56 2.54 2.53 2.45 2.43 2.43
1.51 1.50 1.49 1.49 1.48 1.47 1.47 1.46 1.45 1.45 1.43
0.98 0.96 0.94
NO2
O
Me
O
Me
1.97
2.81
0.92
1.94
2.01
3.00
f1 (ppm)

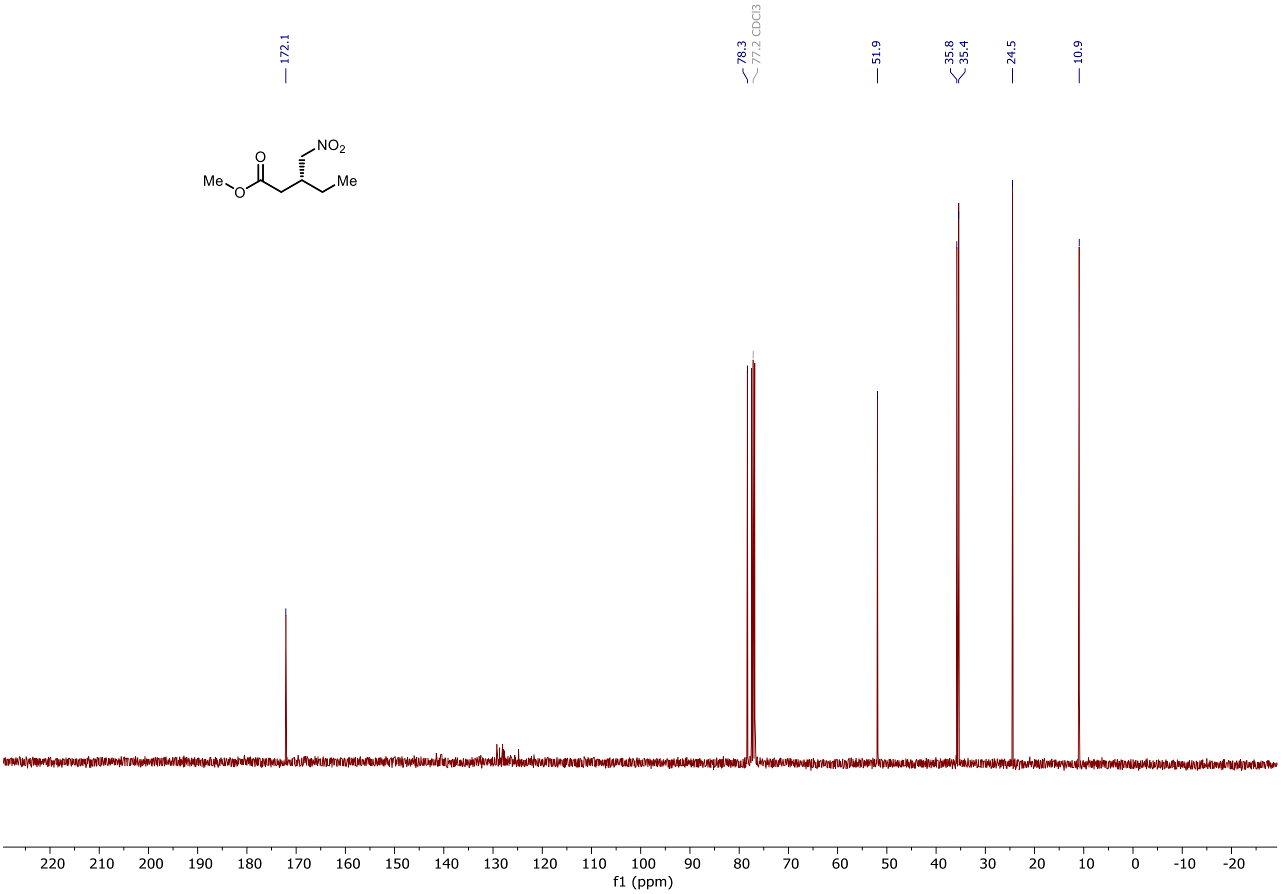
172.1
78.3
77.2 CDCl3
51.9
35.8
35.4
24.5
10.9
NO2
O
Me
O
Me
f1 (ppm)

[ToC]

3g

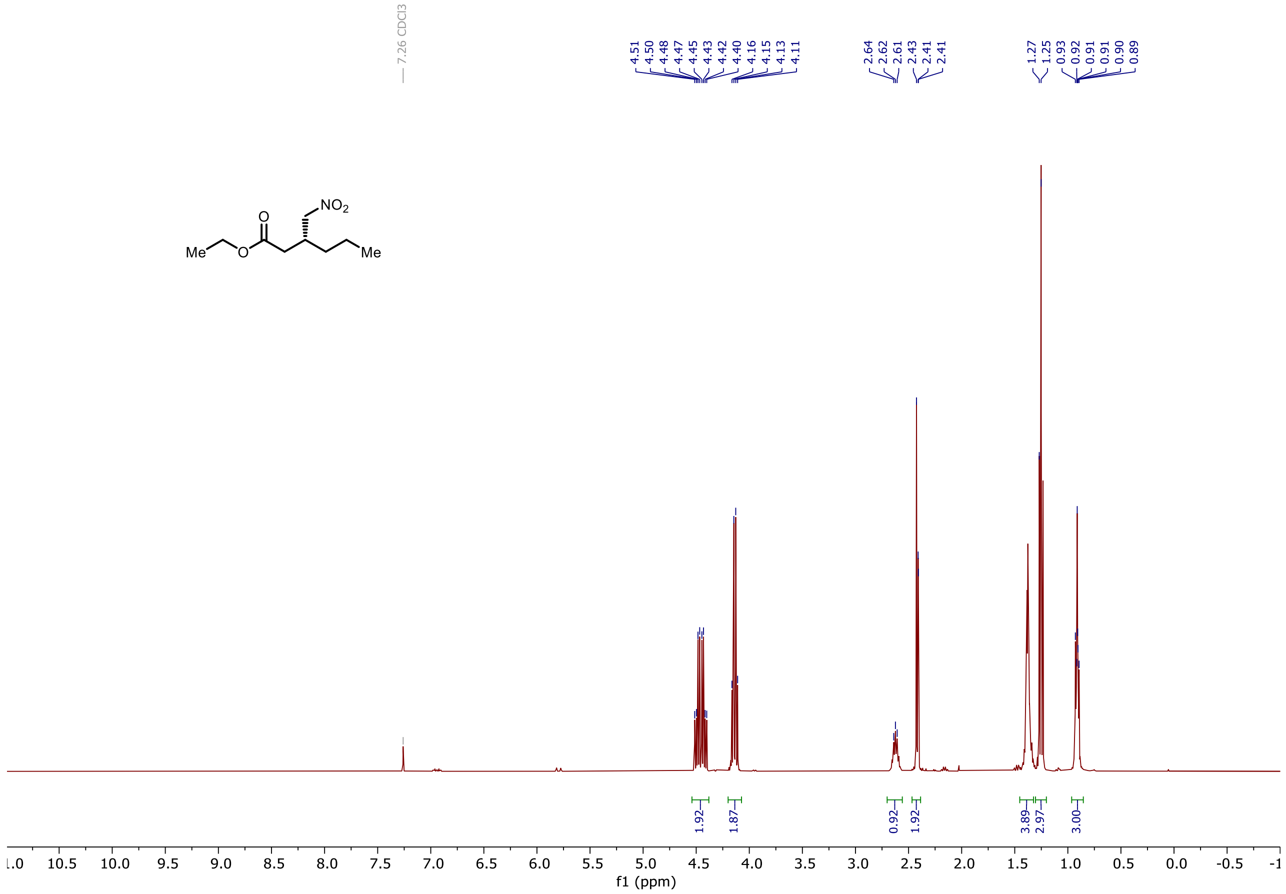
7.26 CDCl3
4.51
4.50
4.48
4.47
4.45
4.43
4.42
4.40
4.16
4.15
4.13
4.11
2.64
2.62
2.61
2.43
2.41
2.41
1.27
1.25
0.93
0.92
0.91
0.91
0.90
0.89
NO2
O
Me
O
Me
1.92
1.87
0.92
1.92
3.89
2.97
3.00
f1 (ppm)

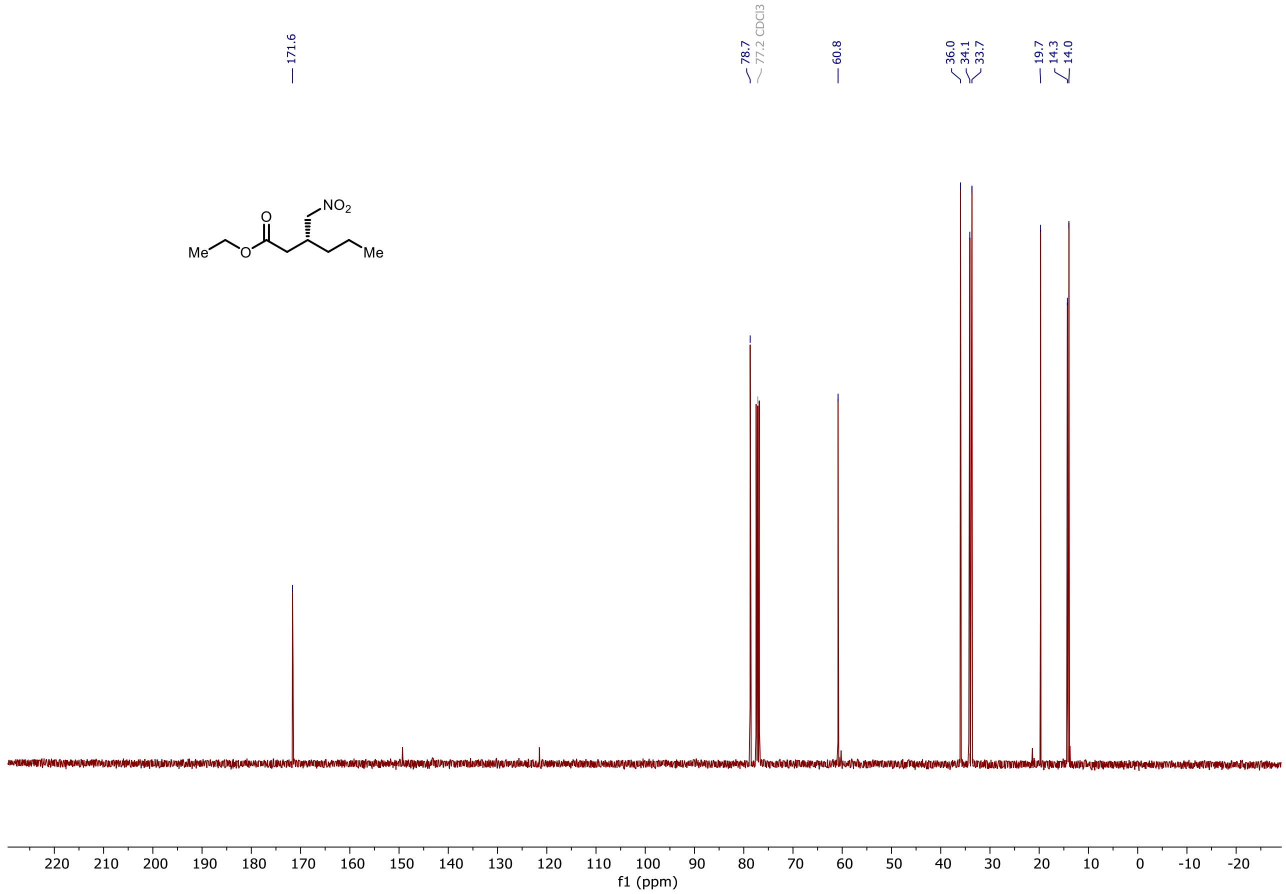
171.6
78.7
77.2 CDCl3
60.8
36.0
34.1
33.7
19.7
14.3
14.0
NO2
O
Me
O
Me
f1 (ppm)

[ToC]

3h

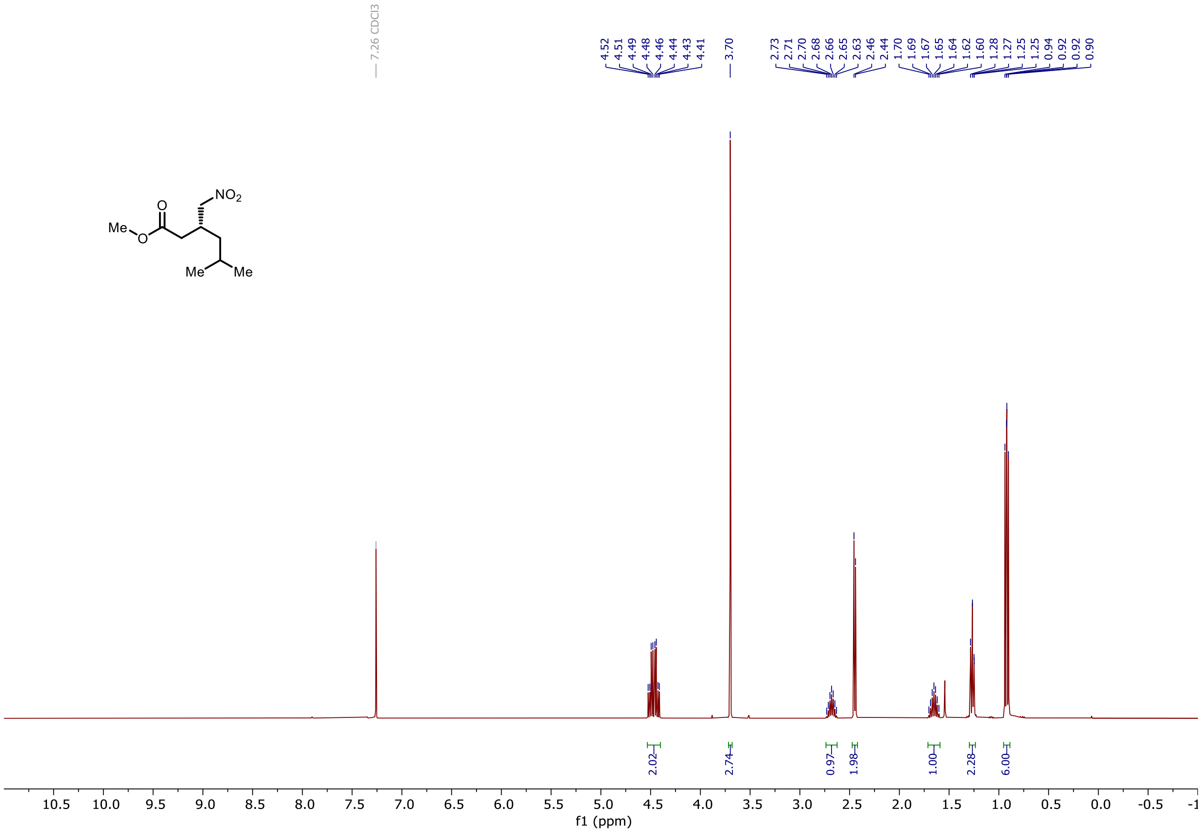
7.26 CDCl3
4.52
4.51
4.49
4.48
4.46
4.44
4.43
4.41
3.70
2.73
2.71
2.70
2.68
2.66
2.65
2.63
2.46
2.44
1.70
1.69
1.67
1.65
1.64
1.62
1.60
1.28
1.27
1.25
1.25
0.94
0.92
0.92
0.90
NO2
O
Me
O
Me
Me
2.02
2.74
0.97
1.98
1.00
2.28
6.00
f1 (ppm)

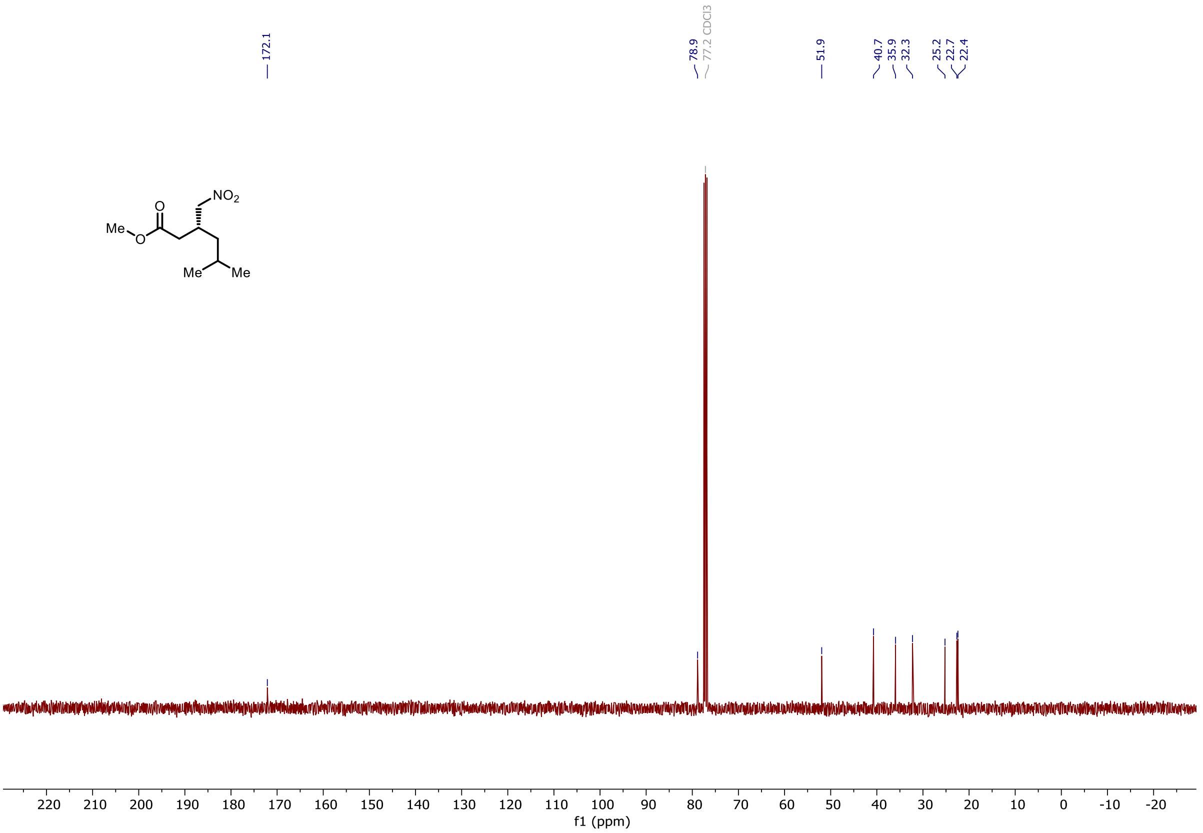
172.1
78.9
77.2 CDCl3
51.9
40.7
35.9
32.3
25.2
22.7
22.4
NO2
O
Me
O
Me
Me
f1 (ppm)

[ToC]

3i

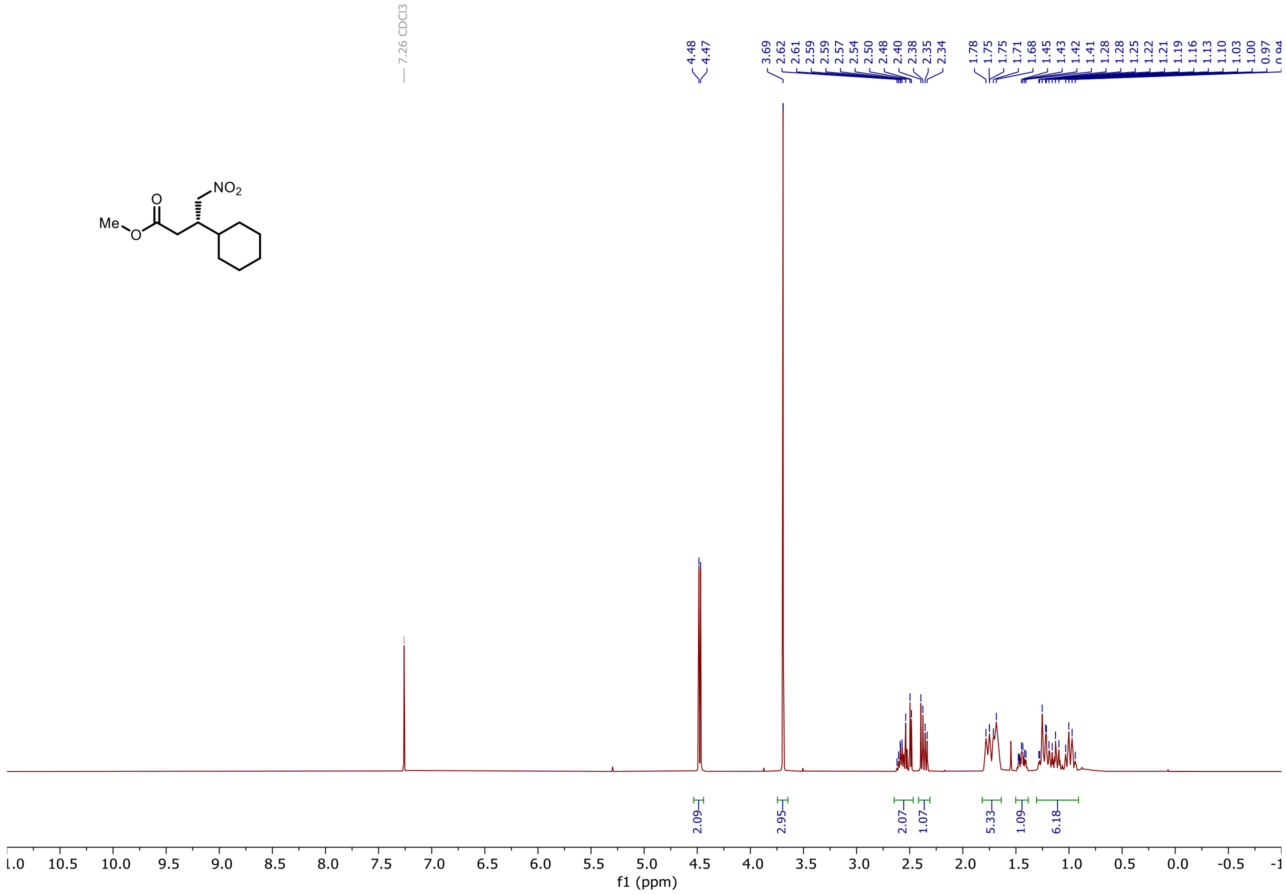
7.26 CDCl3
4.48
4.47
3.69
2.62
2.61
2.59
2.59
2.57
2.54
2.50
2.48
2.40
2.38
2.35
2.34
1.78
1.75
1.75
1.71
1.68
1.45
1.43
1.42
1.41
1.28
1.28
1.25
1.22
1.21
1.19
1.16
1.13
1.10
1.03
1.00
0.97
NO2
O
Me
O
2.09
2.95
2.07
1.07
5.33
1.09
6.18
f1 (ppm)

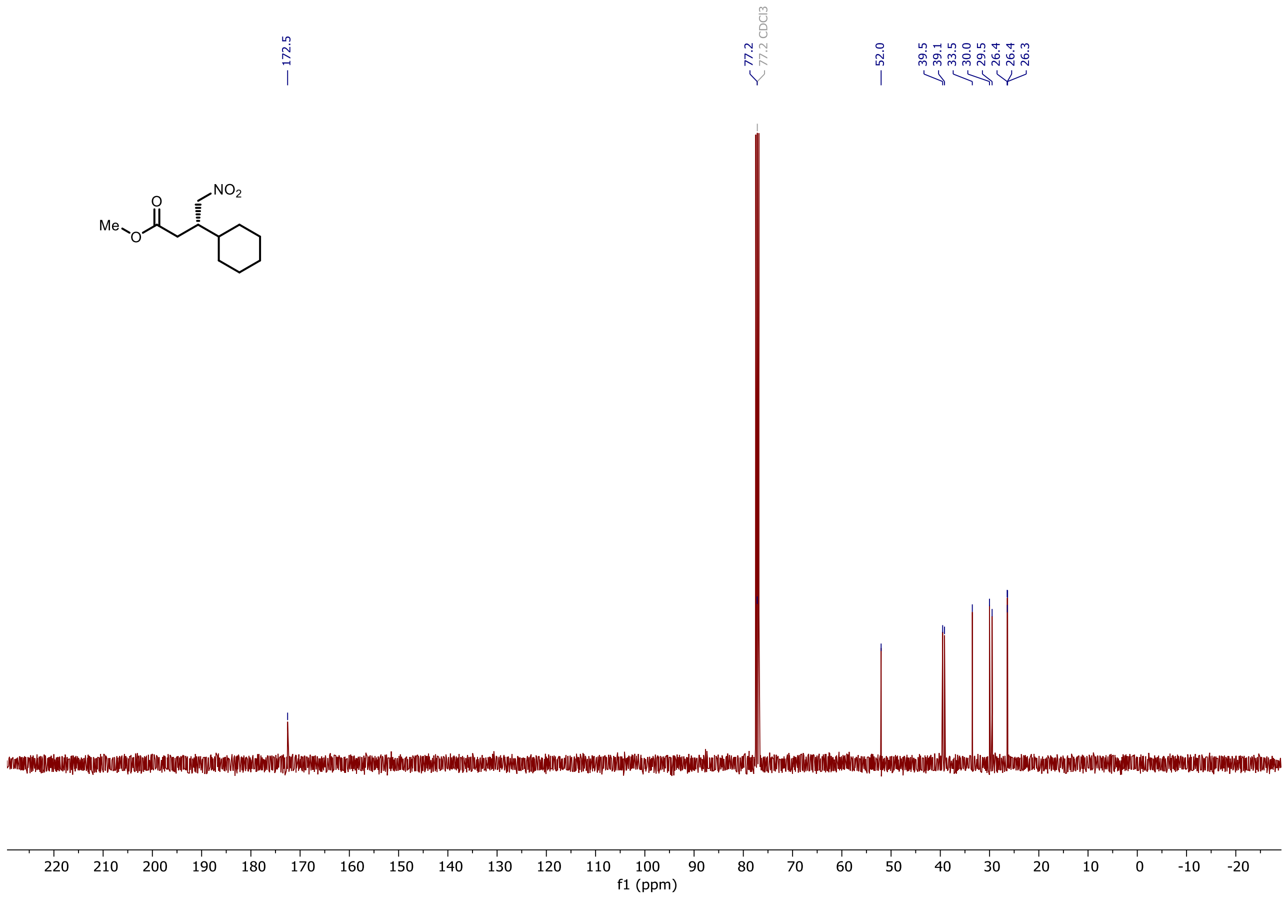
172.5
77.2
77.2 CDCl3
52.0
39.5
39.1
33.5
30.0
29.5
26.4
26.4
26.3
NO2
O
Me
O
f1 (ppm)

[ToC]

3j

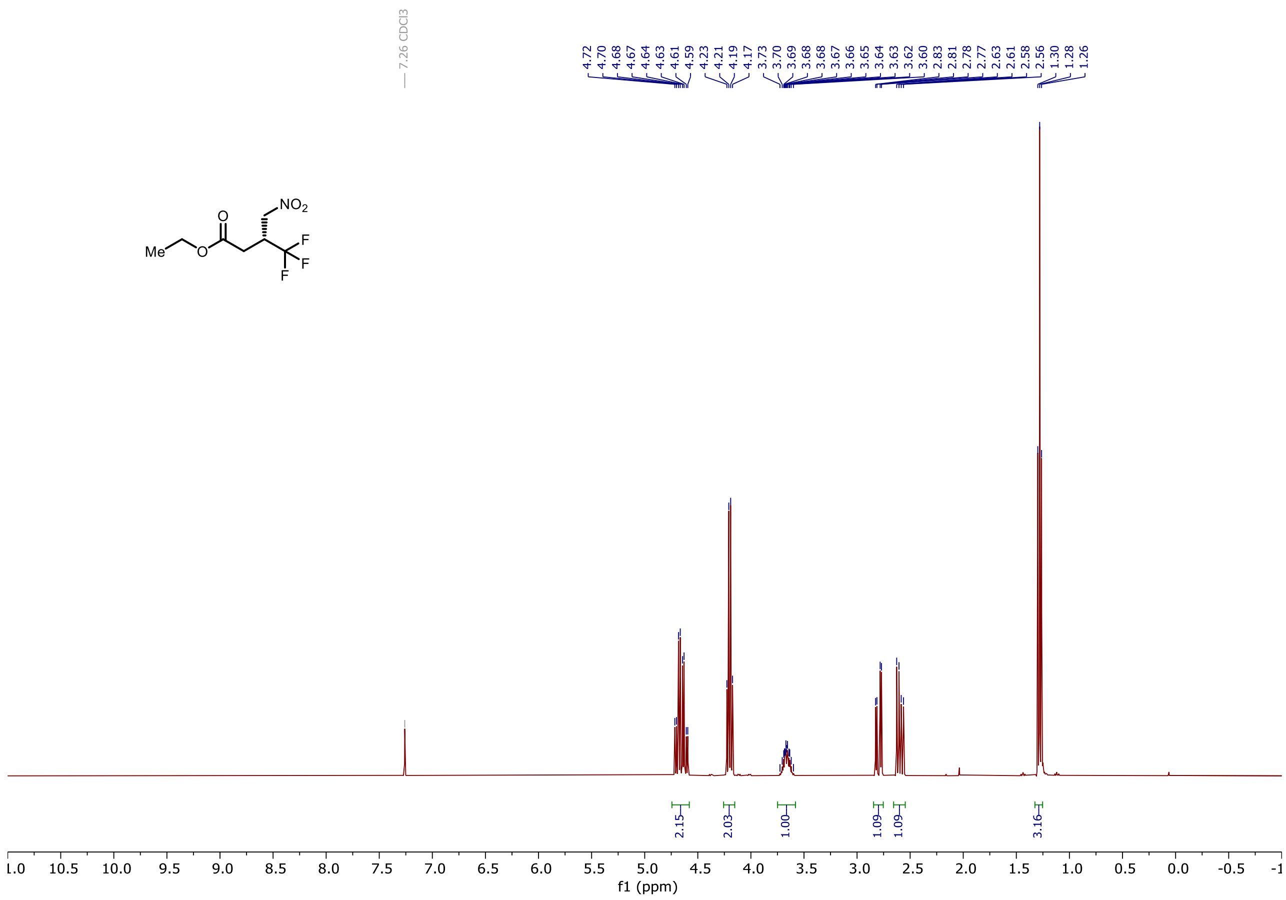
7.26 CDCl3
4.72
4.70
4.68
4.67
4.64
4.63
4.61
4.59
4.23
4.21
4.19
4.17
3.73
3.70
3.69
3.68
3.67
3.66
3.65
3.64
3.63
3.62
3.60
2.83
2.81
2.78
2.77
2.63
2.61
2.58
2.56
1.30
1.28
1.26
NO2
O
Me
O
F
F
F
2.15
2.03
1.00
1.09
1.09
3.16
f1 (ppm)

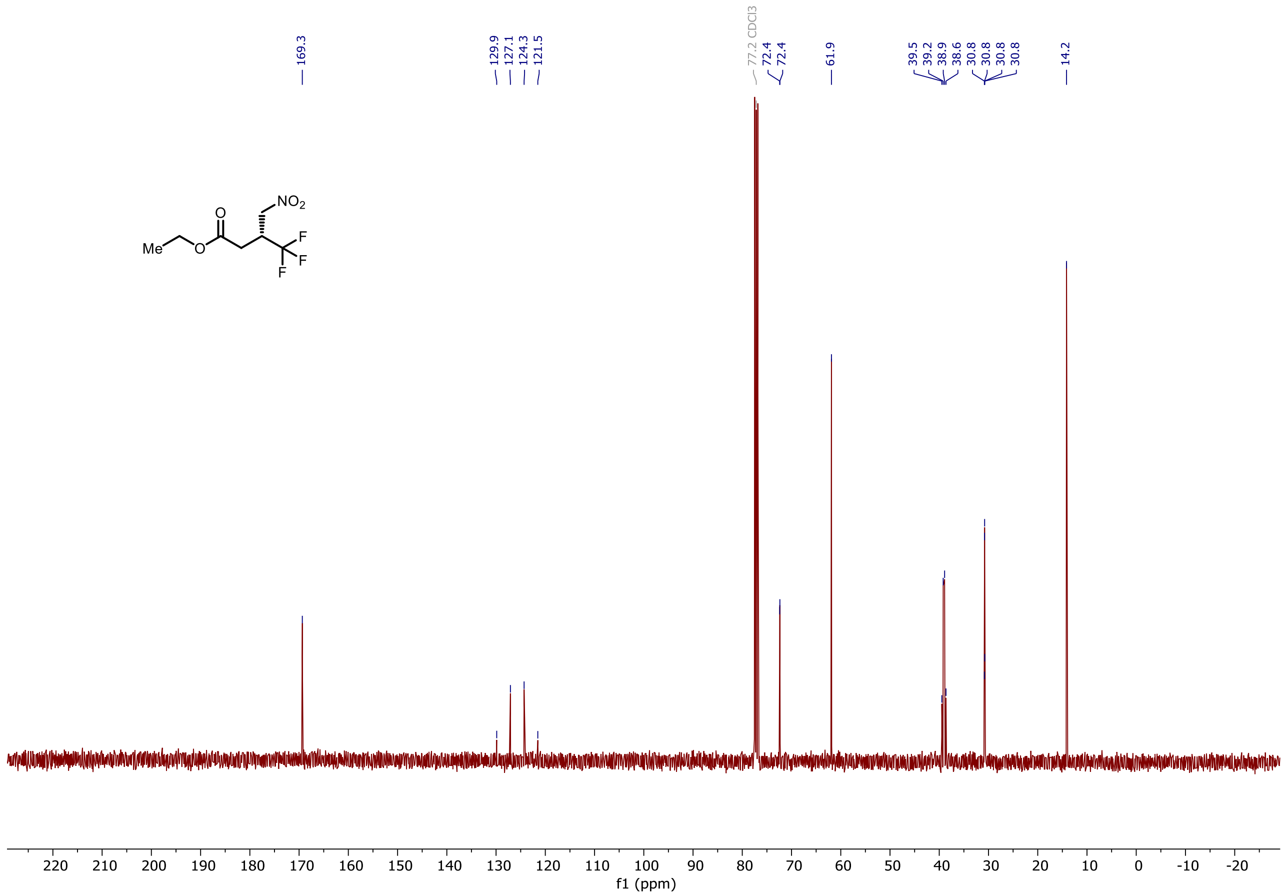
169.3
129.9
127.1
124.3
121.5
77.2 CDCl3
72.4
72.4
61.9
39.5
39.2
38.9
38.6
30.8
30.8
30.8
30.8
14.2
NO2
O
Me
O
F
F
F
f1 (ppm)

[ToC]

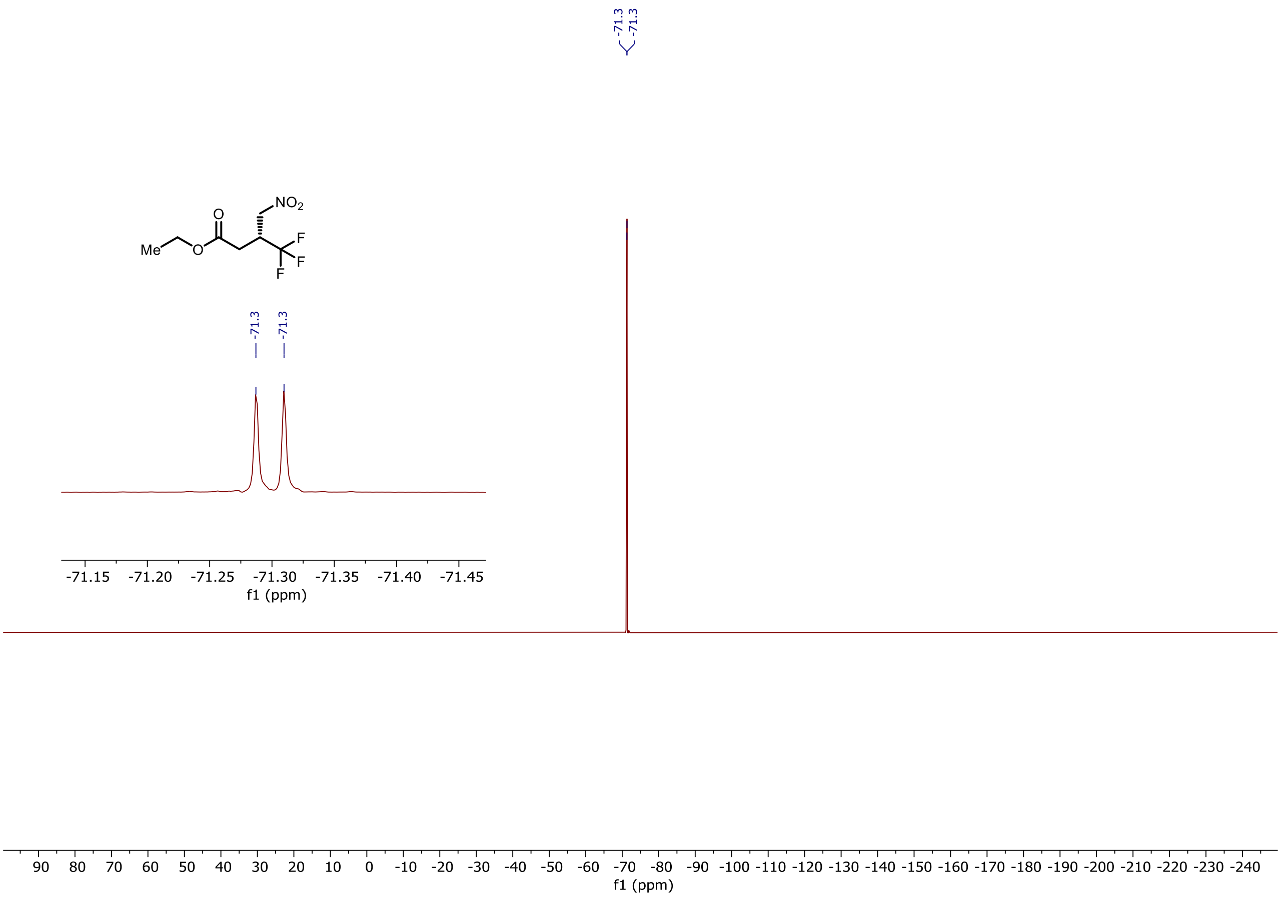

-71.3
-71.3
NO2
O
Me
O
F
F
F
-71.15 -71.20 -71.25 -71.30 -71.35 -71.40 -71.45
f1 (ppm)
90 80 70 60 50 40 30 20 10 0 -10 -20 -30 -40 -50 -60 -70 -80 -90 -100 -110 -120 -130 -140 -150 -160 -170 -180 -190 -200 -210 -220 -230 -240
f1 (ppm)

[ToC]

3k

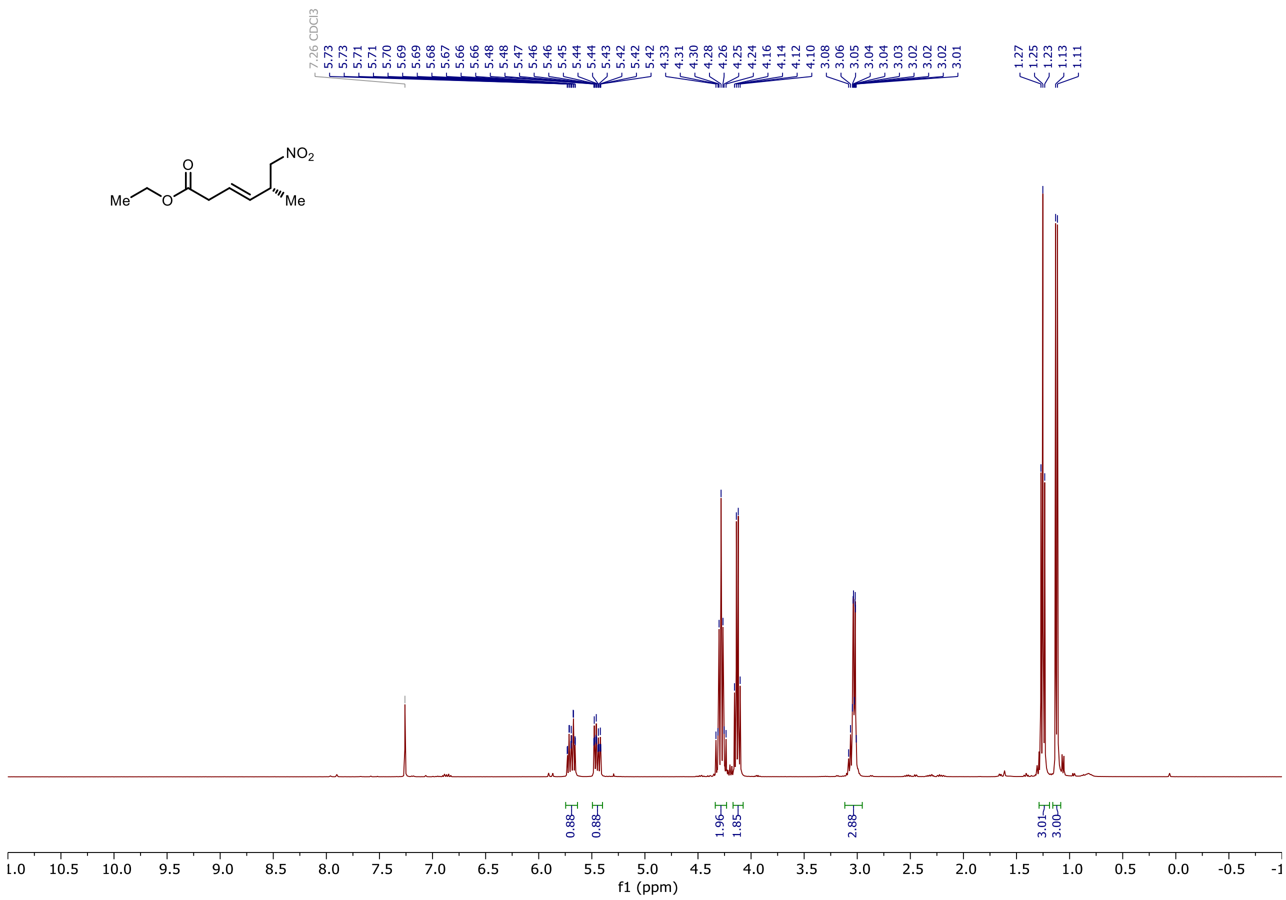
7.26 CDCl3
5.73
5.73
5.71
5.71
5.70
5.69
5.69
5.68
5.67
5.66
5.66
5.48
5.48
5.47
5.46
5.46
5.45
5.44
5.44
5.43
5.42
5.42
5.42
4.33
4.31
4.30
4.28
4.26
4.25
4.24
4.16
4.14
4.12
4.10
3.08
3.06
3.05
3.04
3.04
3.03
3.02
3.02
3.01
1.27
1.25
1.23
1.13
1.11
NO2
O
Me
O
Me
0.88
0.88
1.96
1.85
2.88
3.01
3.00
f1 (ppm)

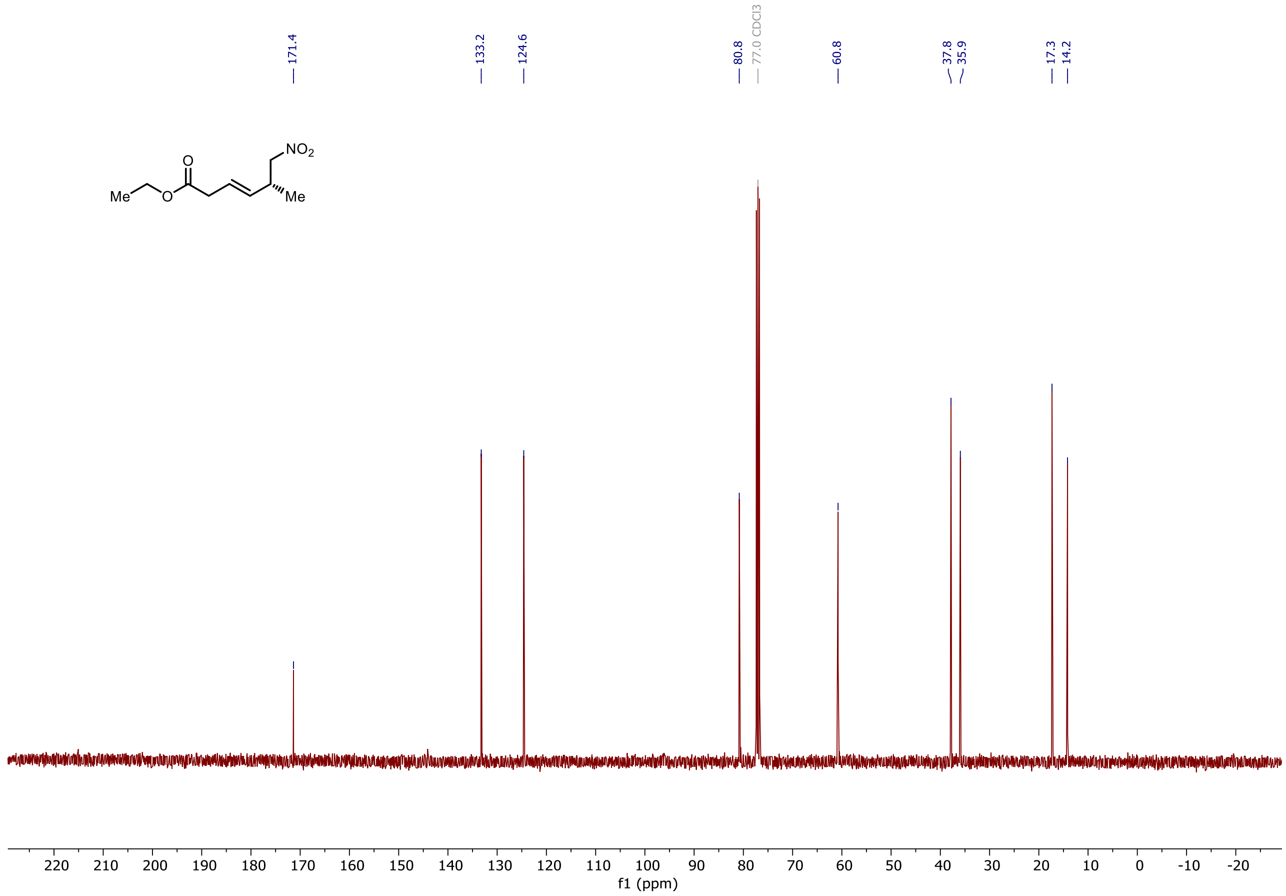
171.4
133.2
124.6
80.8
77.0 CDCl3
60.8
37.8
35.9
17.3
14.2
NO2
O
Me
O
Me
f1 (ppm)

[ToC]

3l

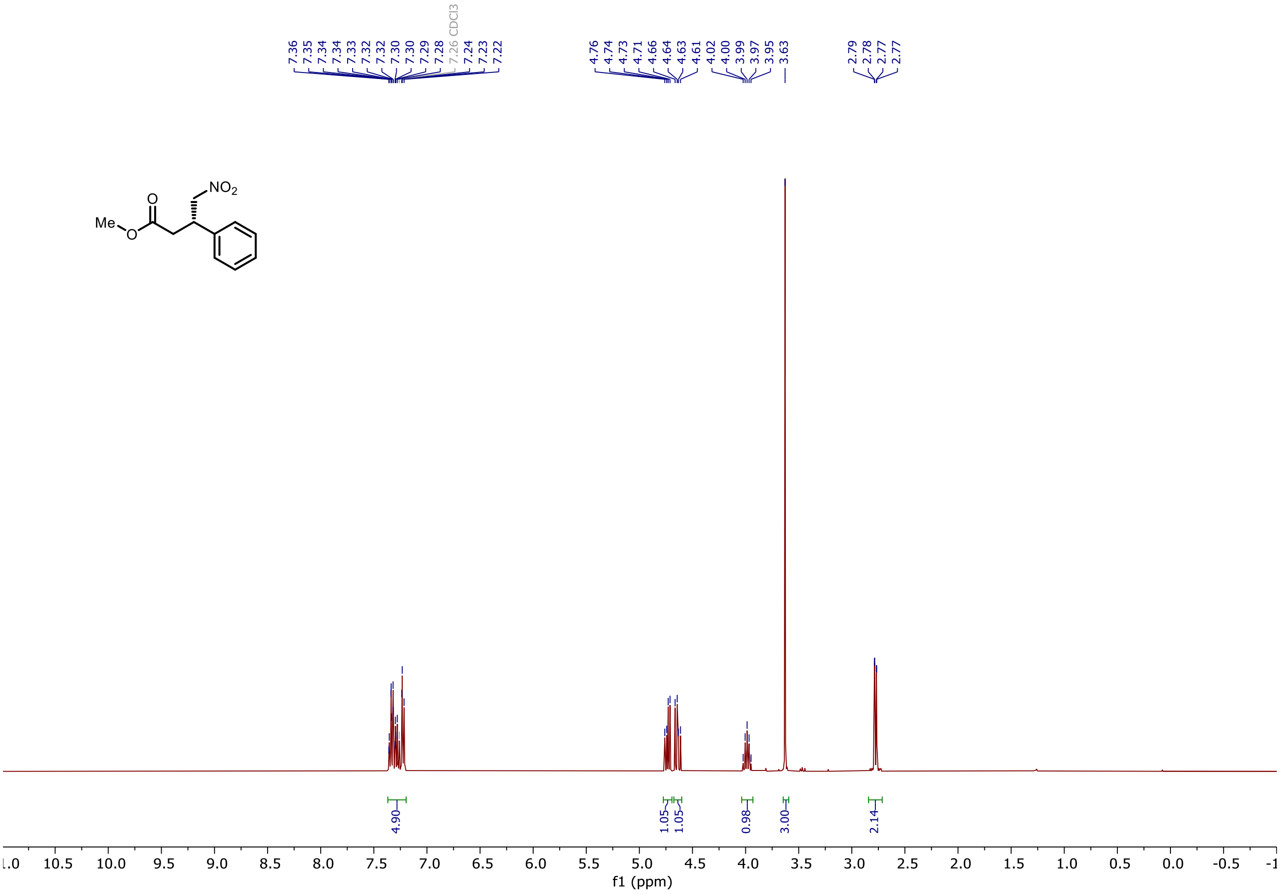

7.36
7.35
7.34
7.34
7.33
7.32
7.32
7.30
7.30
7.29
7.28
7.26 CDCl3
7.24
7.23
7.22
4.76
4.74
4.73
4.71
4.66
4.64
4.63
4.61
4.02
4.00
3.99
3.97
3.95
3.63
2.79
2.78
2.77
2.77
NO2
O
Me
O
4.90
1.05
1.05
0.98
3.00
2.14
f1 (ppm)

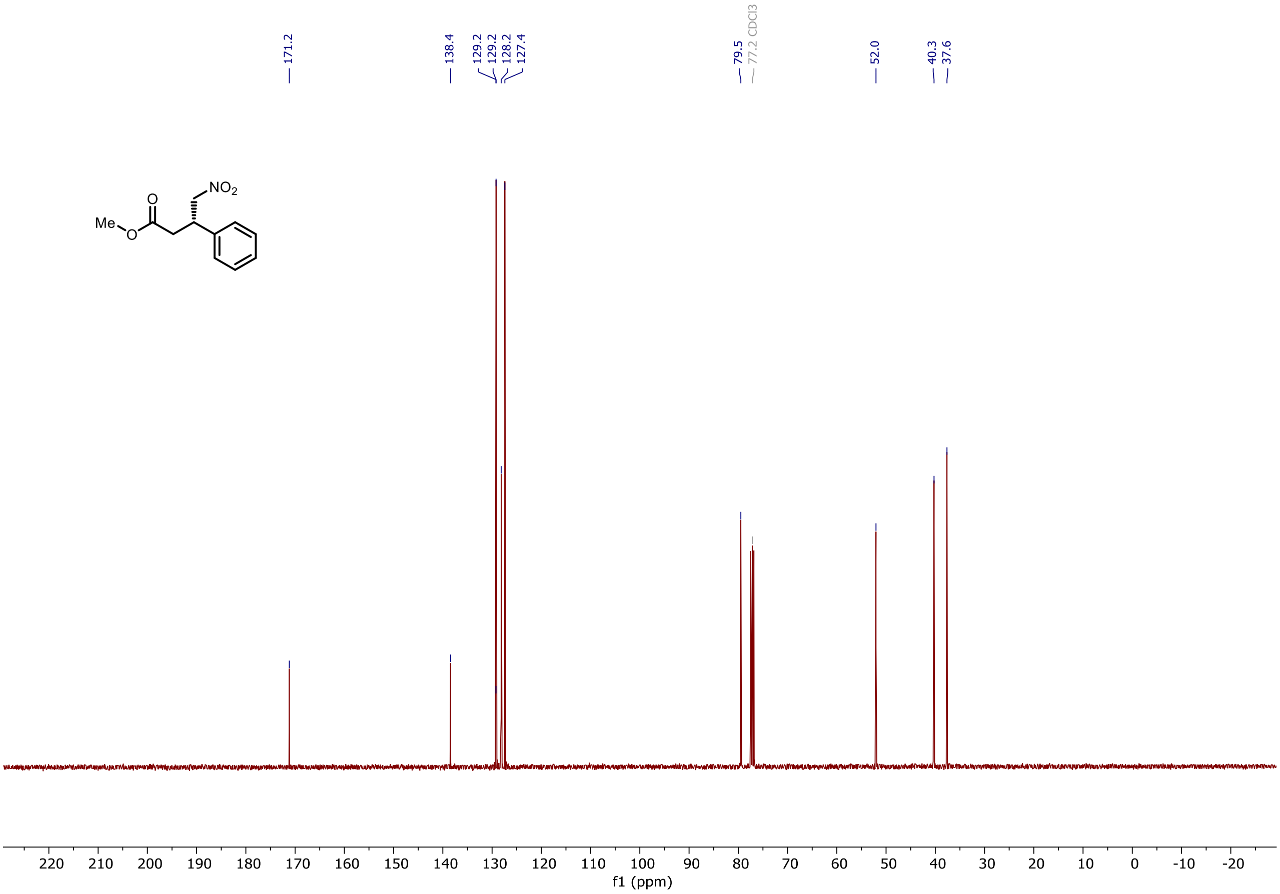

171.2
138.4
129.2
129.2
128.2
127.4
79.5
77.2 CDCl3
52.0
40.3
37.6
NO2
O
Me
O
f1 (ppm)

[ToC]

3m

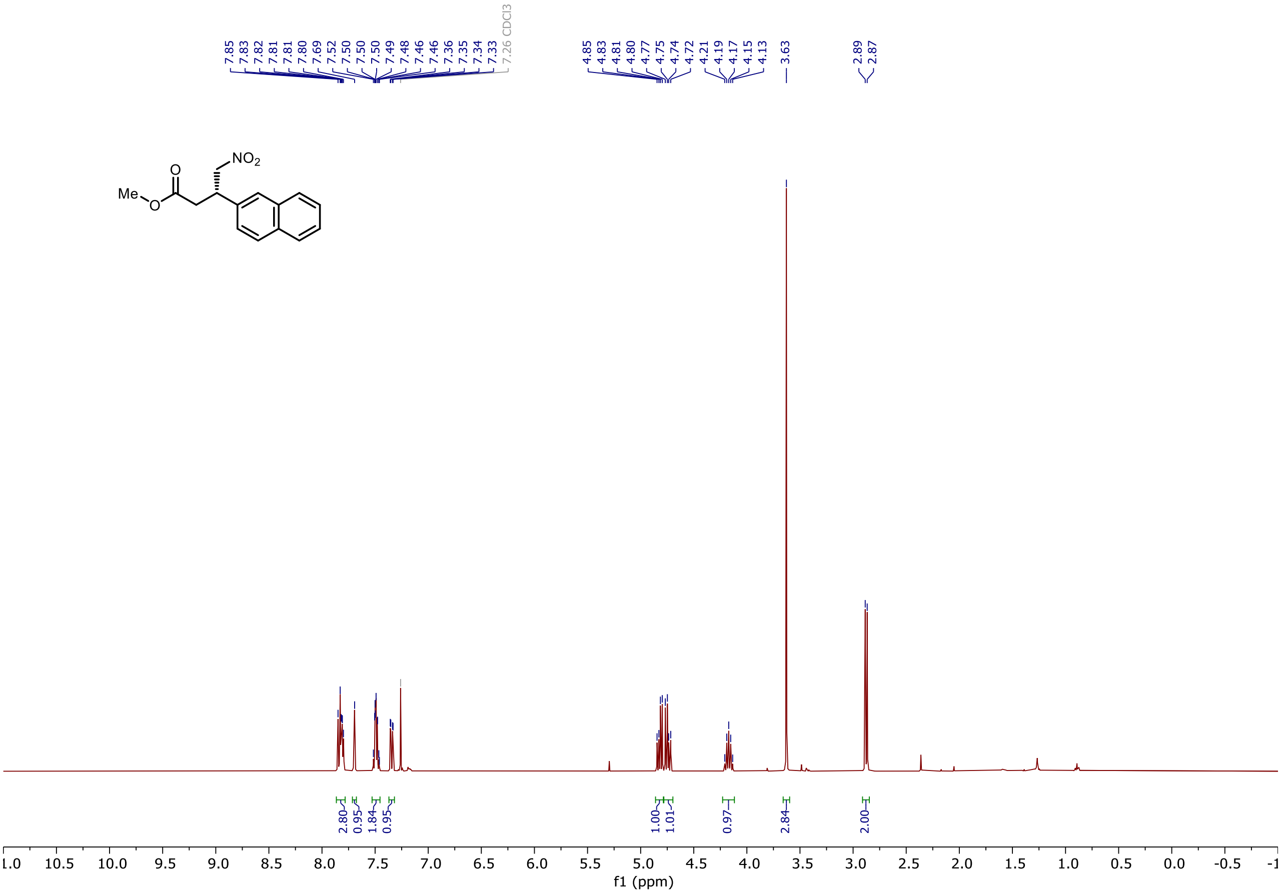

7.85 7.83 7.82 7.81 7.81 7.80 7.69 7.52 7.50 7.50 7.50 7.49 7.48 7.46 7.36 7.35 7.34 7.33 7.26 CDCl3
4.85 4.83 4.81 4.80 4.77 4.75 4.74 4.72 4.21 4.19 4.17 4.15 4.13 3.63 2.89 2.87
NO2
O
Me
O
2.80 0.95 1.84 0.95 1.00 1.01 0.97 2.84 2.00
f1 (ppm)

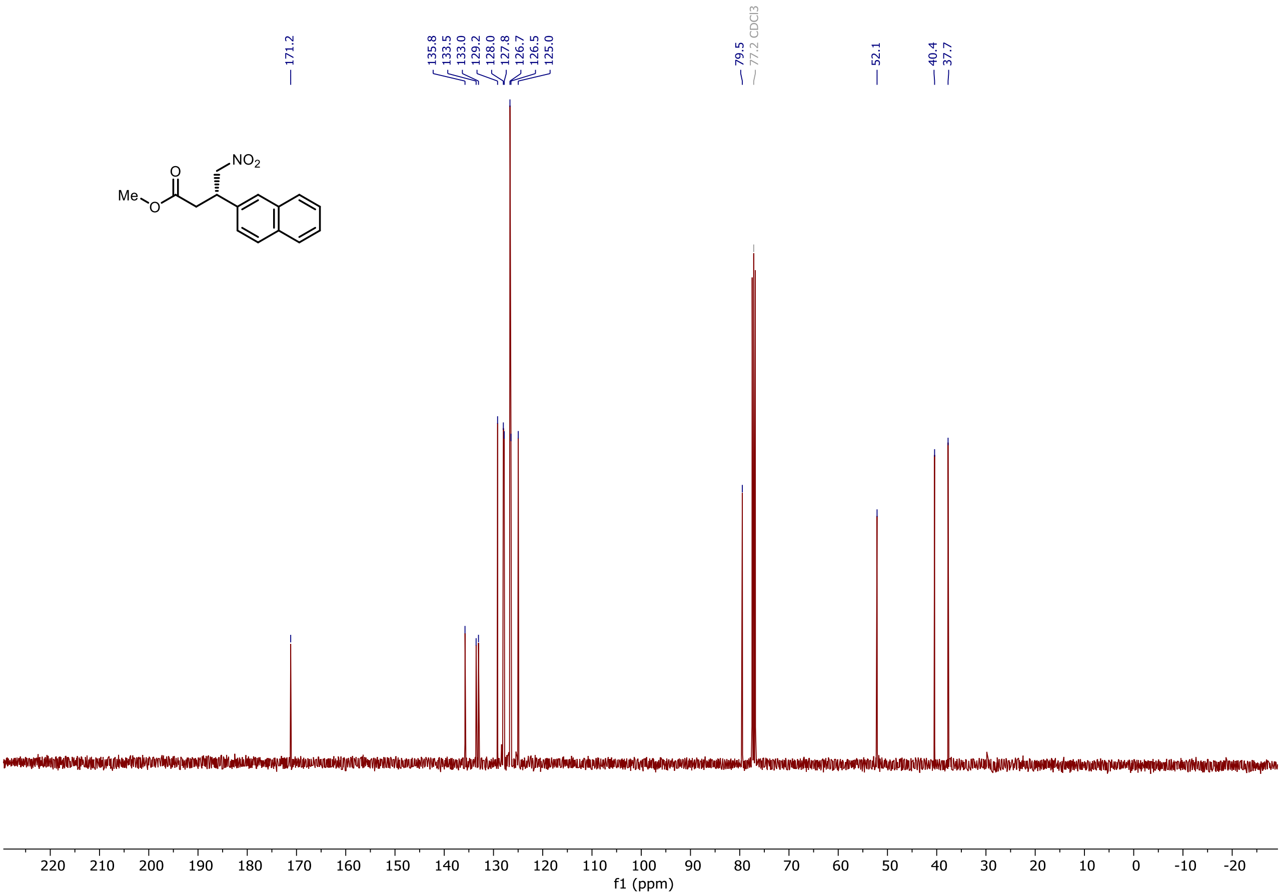

171.2 135.8 133.5 133.0 129.2 128.0 127.8 126.7 126.5 125.0 79.5 77.2 CDCl3 52.1 40.4 37.7
NO2
O
Me
O
f1 (ppm)

[ToC]

3n

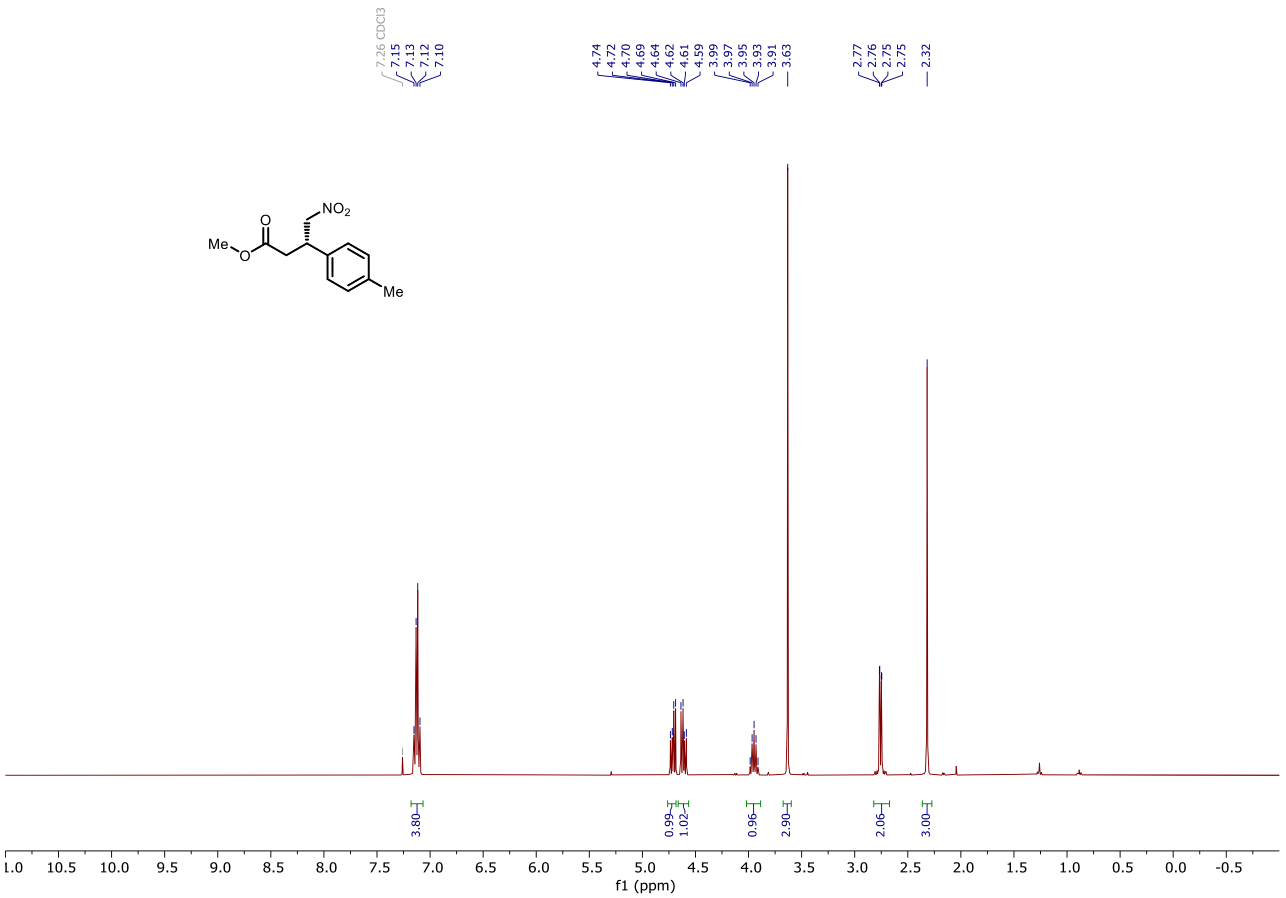

7.26 CDCl3
7.15
7.13
7.12
7.10
4.74
4.72
4.70
4.69
4.64
4.62
4.61
4.59
3.99
3.97
3.95
3.93
3.91
3.63
2.77
2.76
2.75
2.75
2.32
NO2
O
Me
O
Me
3.80
0.99
1.02
0.96
2.90
2.06
3.00
f1 (ppm)

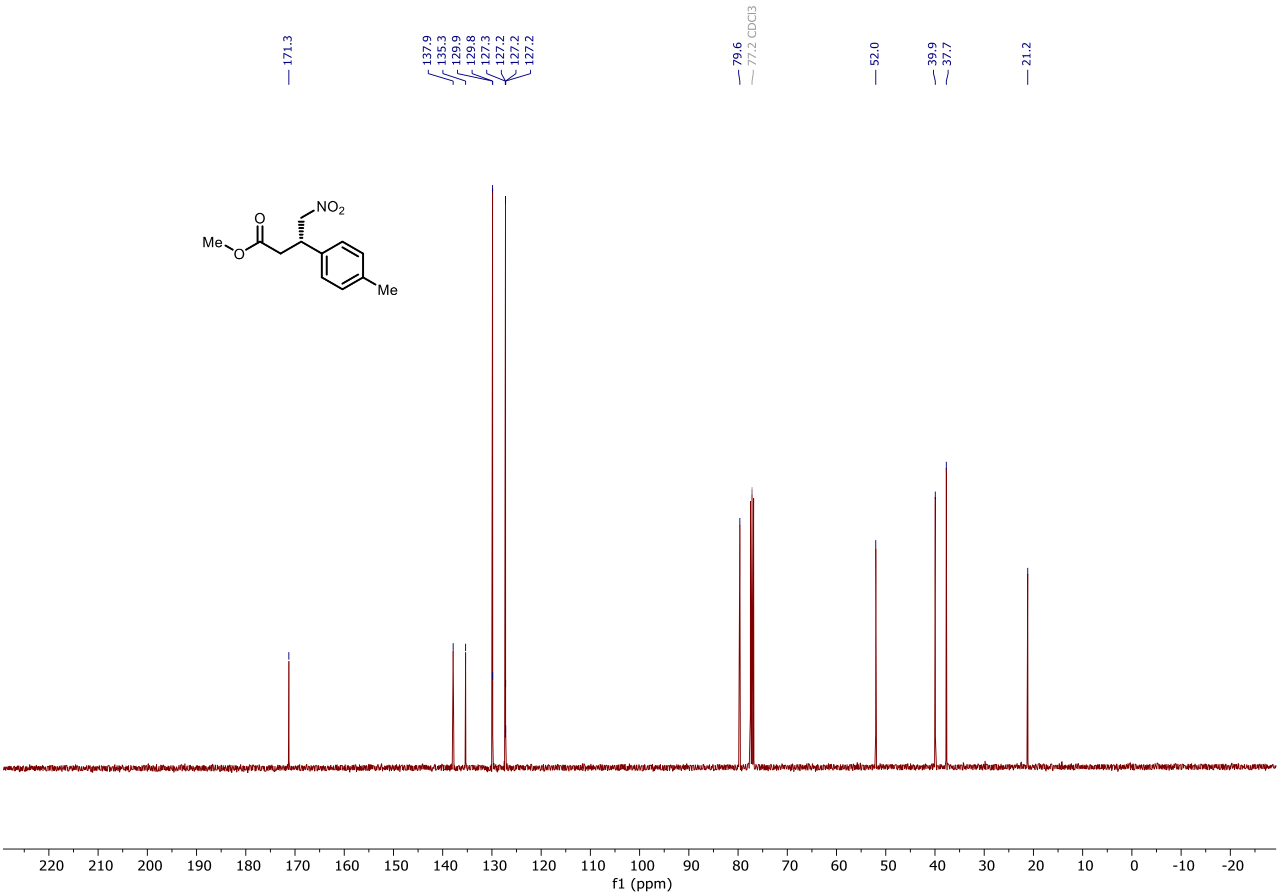

171.3
137.9
135.3
129.9
129.8
127.3
127.2
127.2
127.2
79.6
77.2 CDCl3
52.0
39.9
37.7
21.2
NO2
O
Me
O
Me
f1 (ppm)

[ToC]

3o

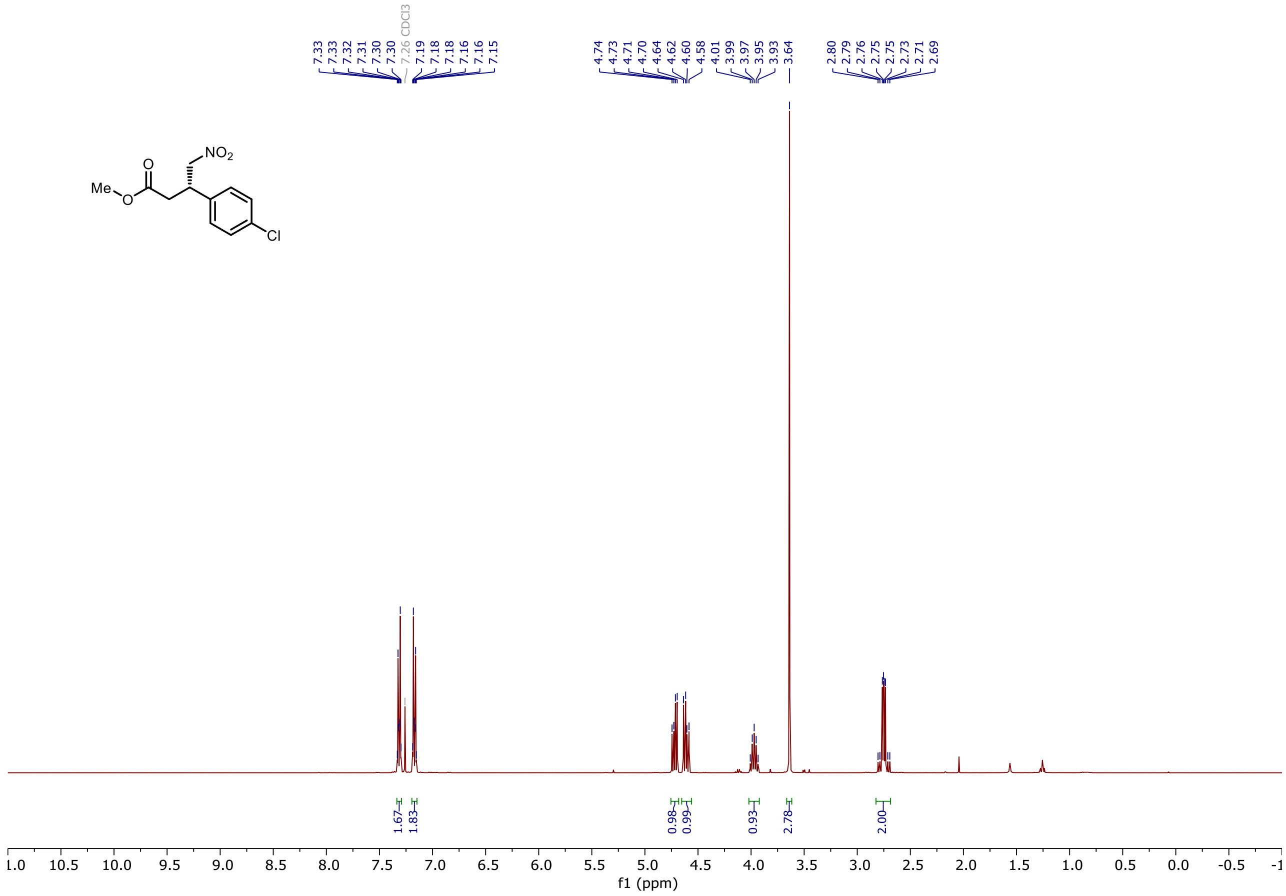
7.33
7.33
7.32
7.31
7.30
7.30
7.26 CDCl3
7.19
7.18
7.18
7.16
7.16
7.15
4.74
4.73
4.71
4.70
4.64
4.62
4.60
4.58
4.01
3.99
3.97
3.95
3.93
3.64
2.80
2.79
2.76
2.75
2.75
2.73
2.71
2.69
NO2
O
Me
O
Cl
1.67
1.83
0.98
0.99
0.93
2.78
2.00
f1 (ppm)

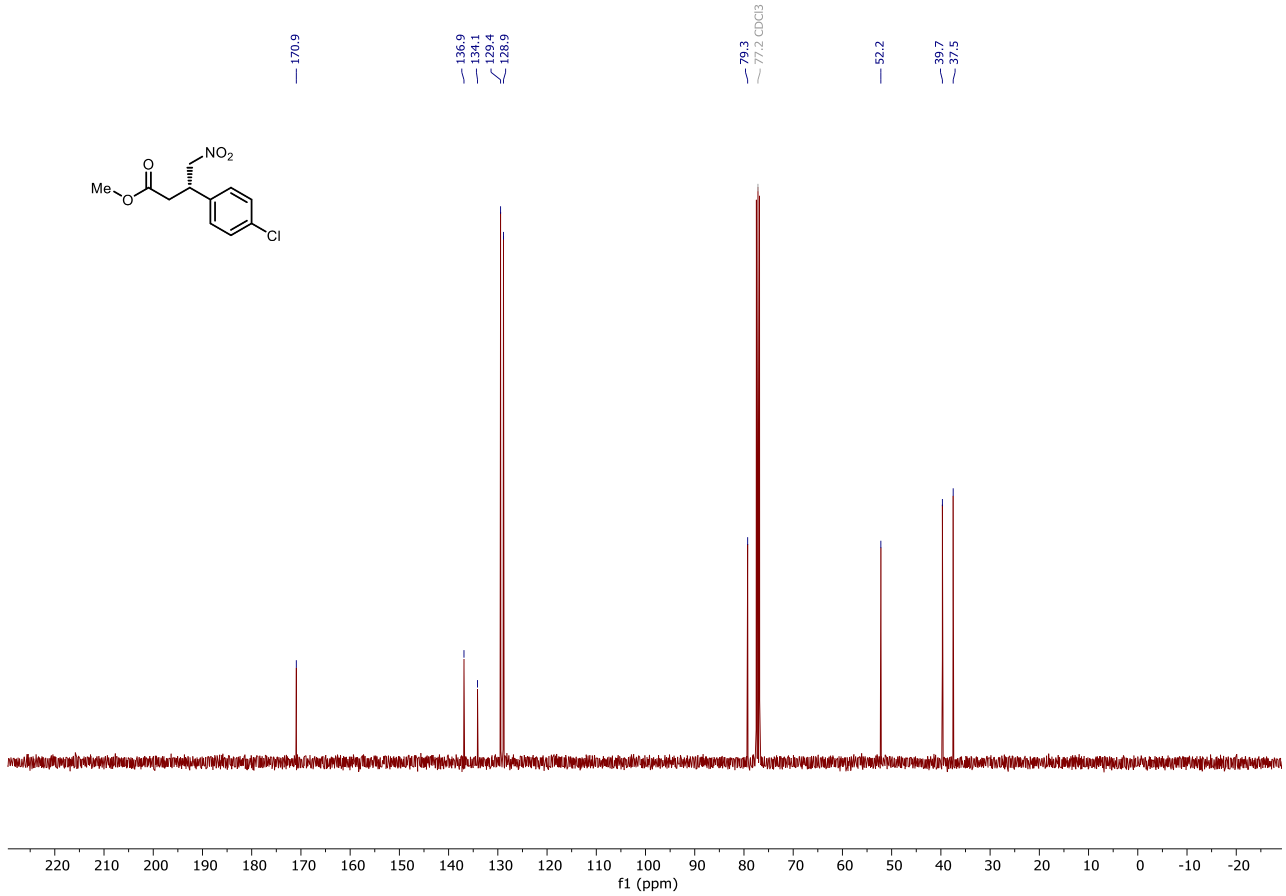
170.9
136.9
134.1
129.4
128.9
79.3
77.2 CDCl3
52.2
39.7
37.5
NO2
O
Me
O
Cl
f1 (ppm)

[ToC]

3p

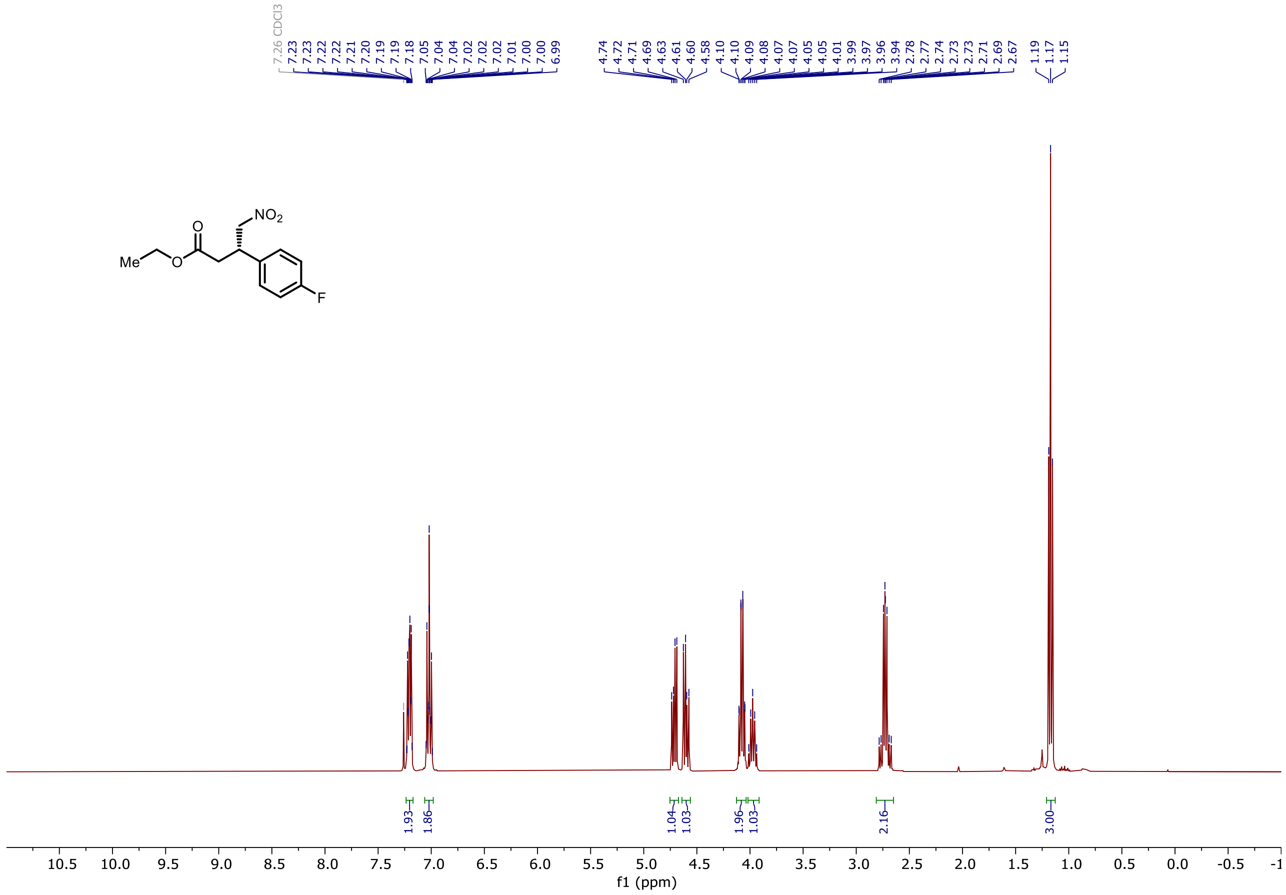

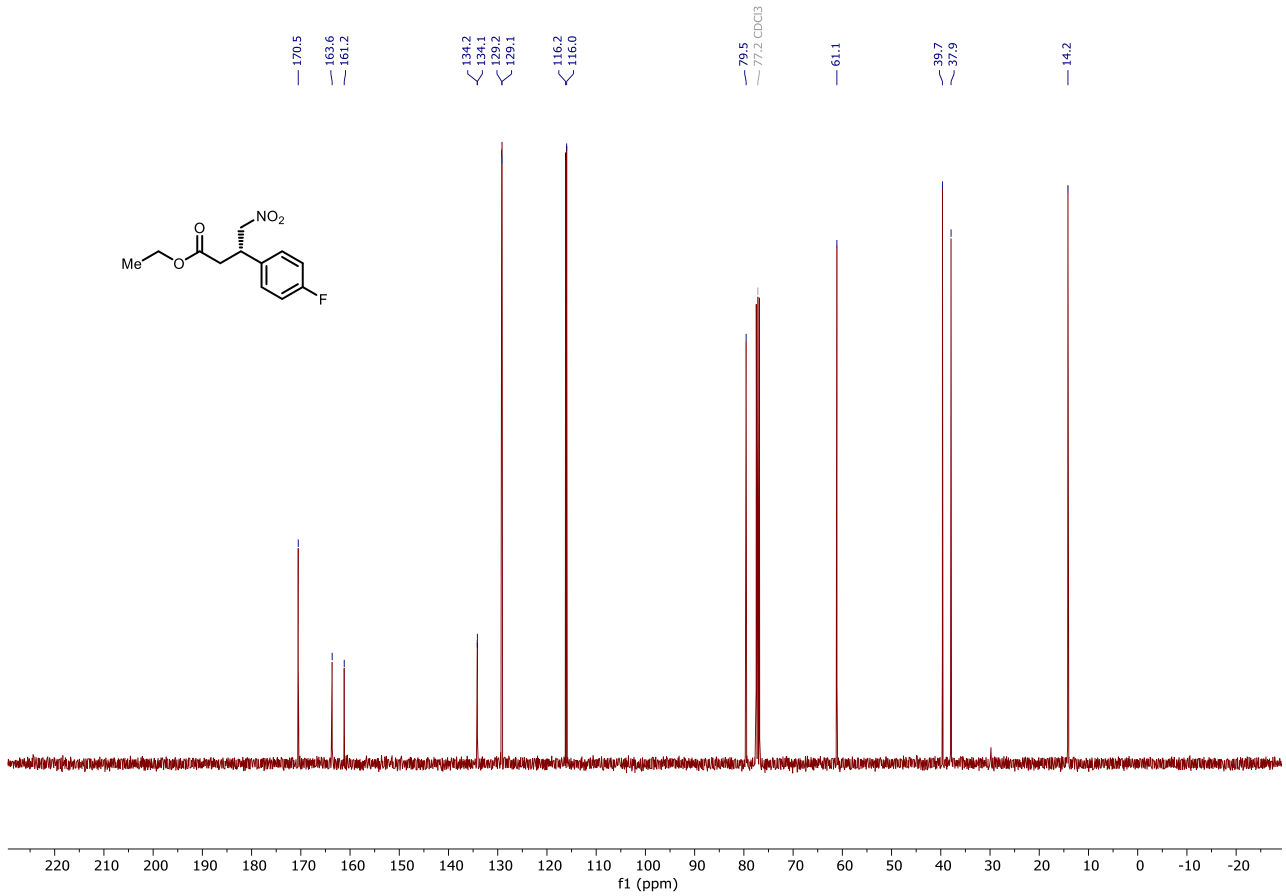

[ToC]

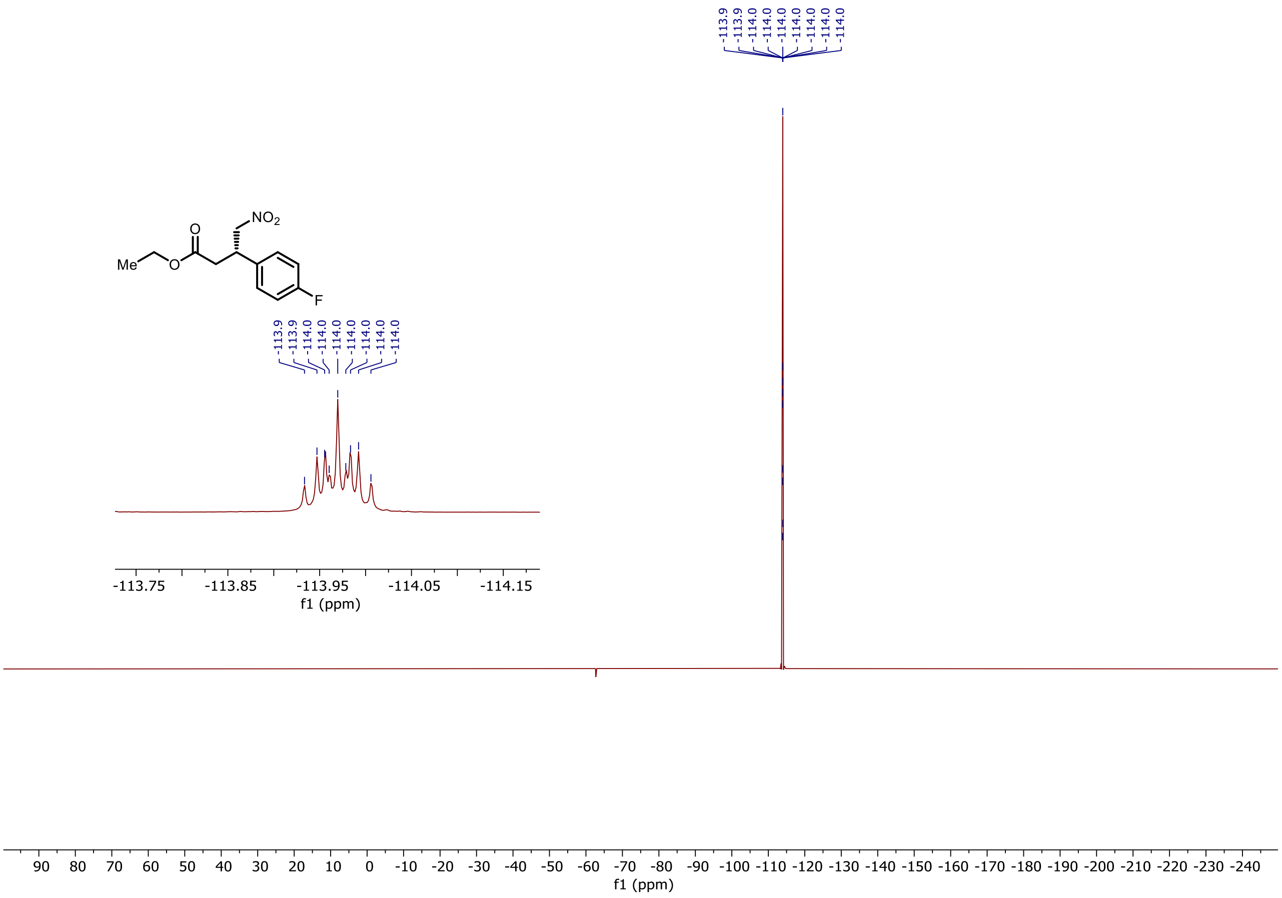
-113.9
-113.9
-114.0
-114.0
-114.0
-114.0
-114.0
-114.0
-114.0
NO2
O
Me
O
F
-113.75
-113.85
-113.95
-114.05
-114.15
f1 (ppm)
90 80 70 60 50 40 30 20 10 0 -10 -20 -30 -40 -50 -60 -70 -80 -90 -100 -110 -120 -130 -140 -150 -160 -170 -180 -190 -200 -210 -220 -230 -240
f1 (ppm)

[ToC]

3q

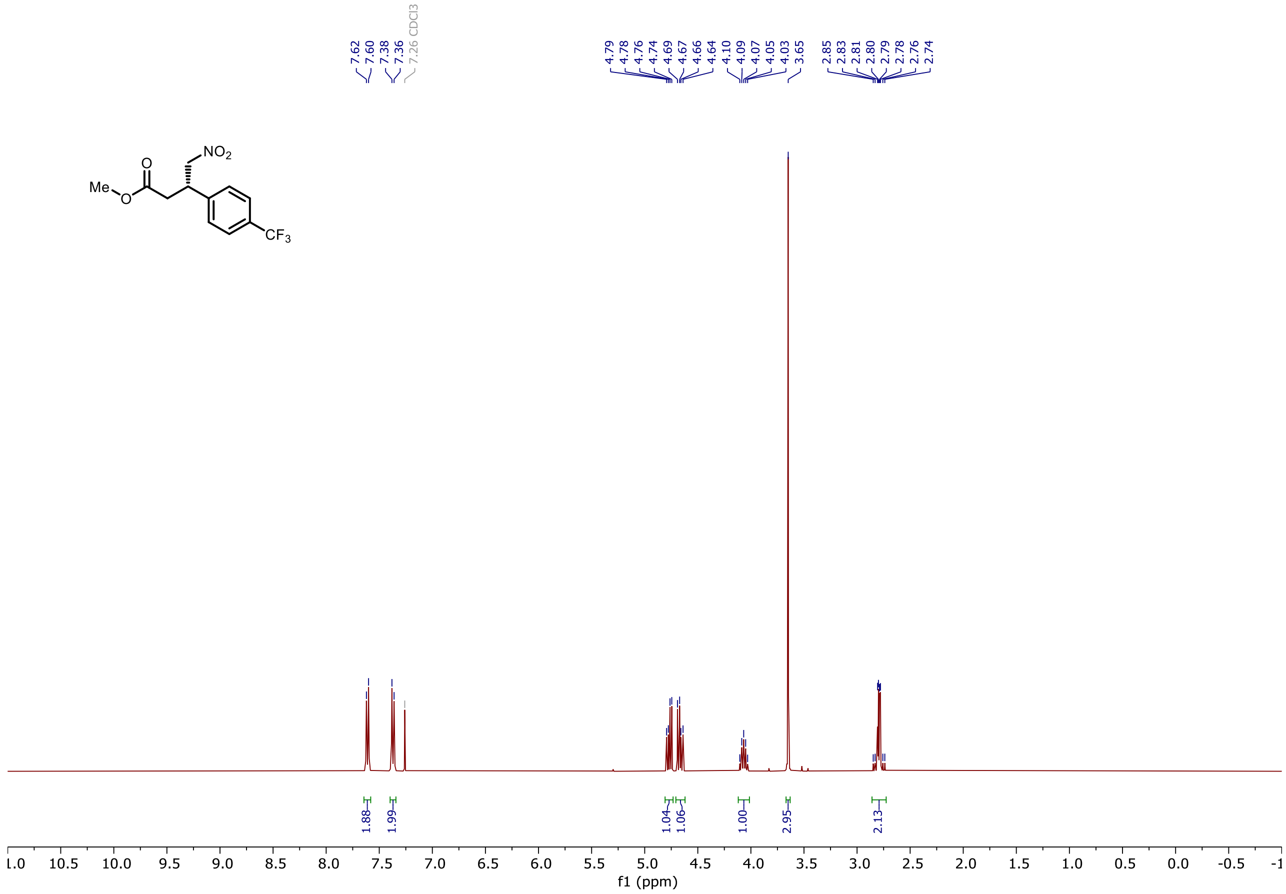
7.62
7.60
7.38
7.36
7.26 CDCl3
4.79
4.78
4.76
4.74
4.69
4.67
4.66
4.64
4.10
4.09
4.07
4.05
4.03
3.65
2.85
2.83
2.81
2.80
2.79
2.78
2.76
2.74
NO2
O
Me
O
CF3
1.88
1.99
1.04
1.06
1.00
2.95
2.13
f1 (ppm)

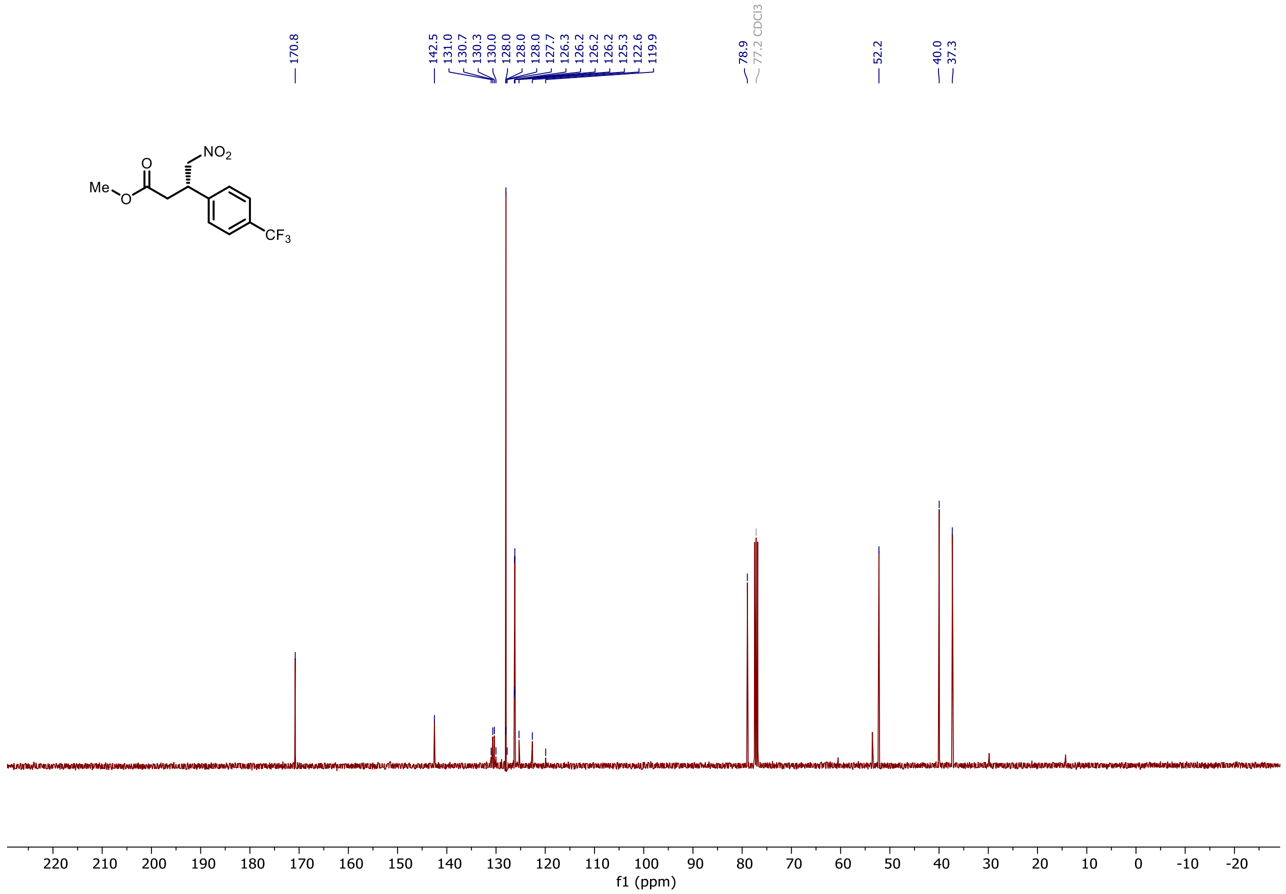
170.8
142.5
131.0
130.7
130.3
130.0
128.0
128.0
128.0
127.7
126.3
126.2
126.2
126.2
125.3
122.6
119.9
78.9
77.2 CDCl3
52.2
40.0
37.3
NO2
O
Me
O
CF3
f1 (ppm)

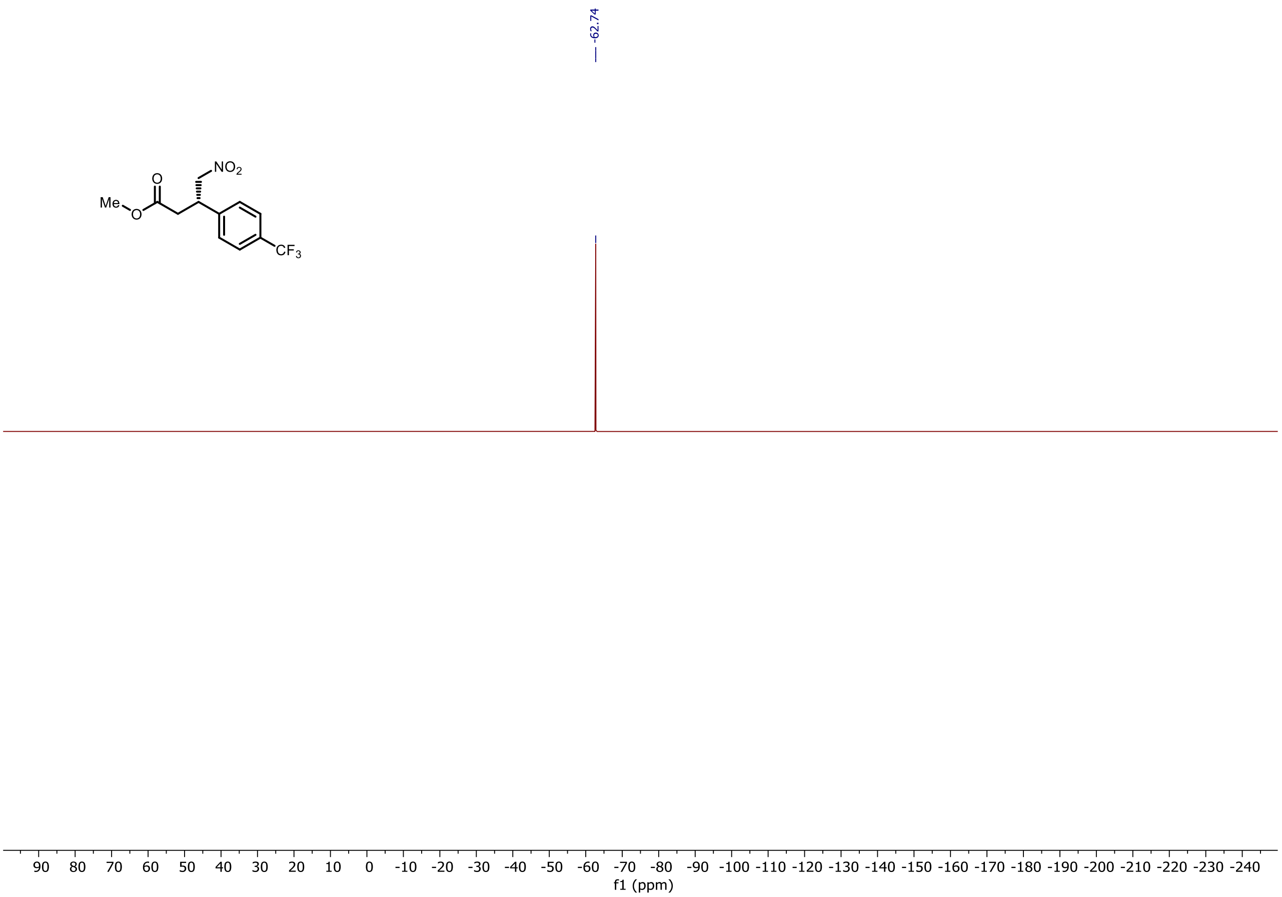
-62.74
90
80
70
60
50
40
30
20
10
0
-10
-20
-30
-40
-50
-60
-70
-80
-90
-100
-110
-120
-130
-140
-150
-160
-170
-180
-190
-200
-210
-220
-230
-240
f1 (ppm)

[[ToC](#)]

3r

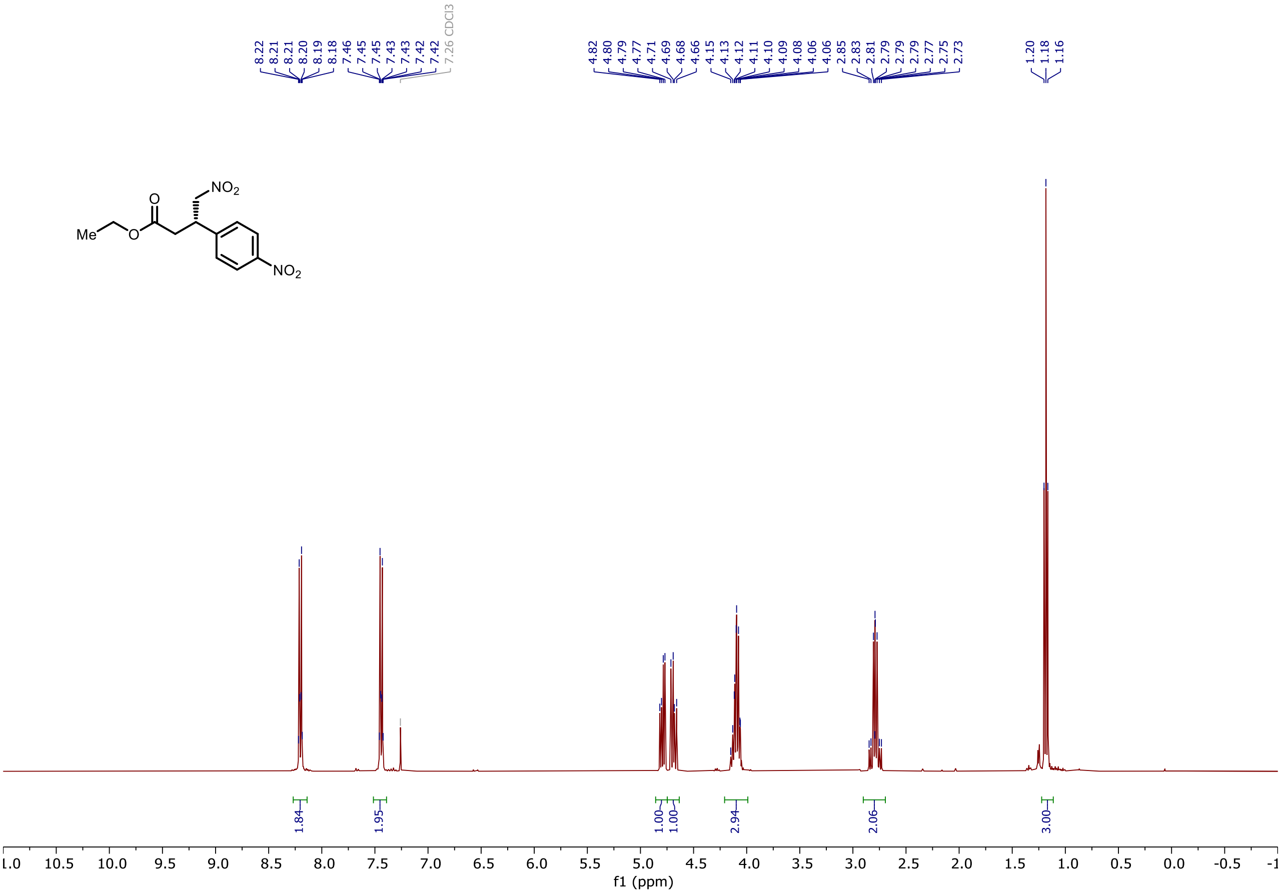

8.22
8.21
8.21
8.20
8.19
8.18
7.46
7.45
7.45
7.43
7.43
7.42
7.42
7.26 CDCl3
4.82
4.80
4.79
4.77
4.71
4.69
4.68
4.66
4.15
4.13
4.12
4.11
4.10
4.09
4.08
4.06
2.85
2.83
2.81
2.79
2.79
2.77
2.75
2.73
1.20
1.18
1.16
NO2
O
Me
O
NO2
1.84
1.95
1.00
1.00
2.94
2.06
3.00
f1 (ppm)

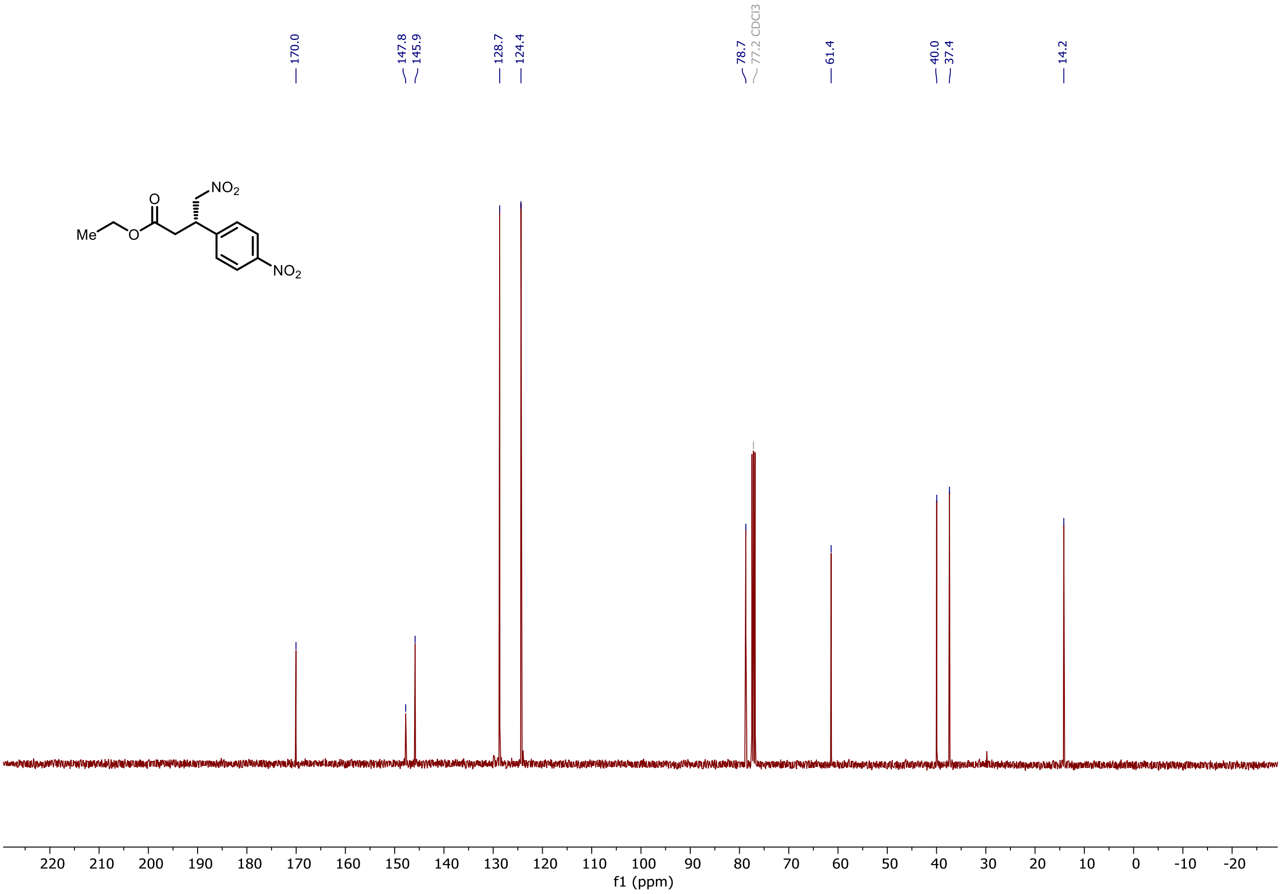

170.0
147.8
145.9
128.7
124.4
78.7
77.2 CDCl3
61.4
40.0
37.4
14.2
NO2
O
Me
O
NO2
f1 (ppm)

3s

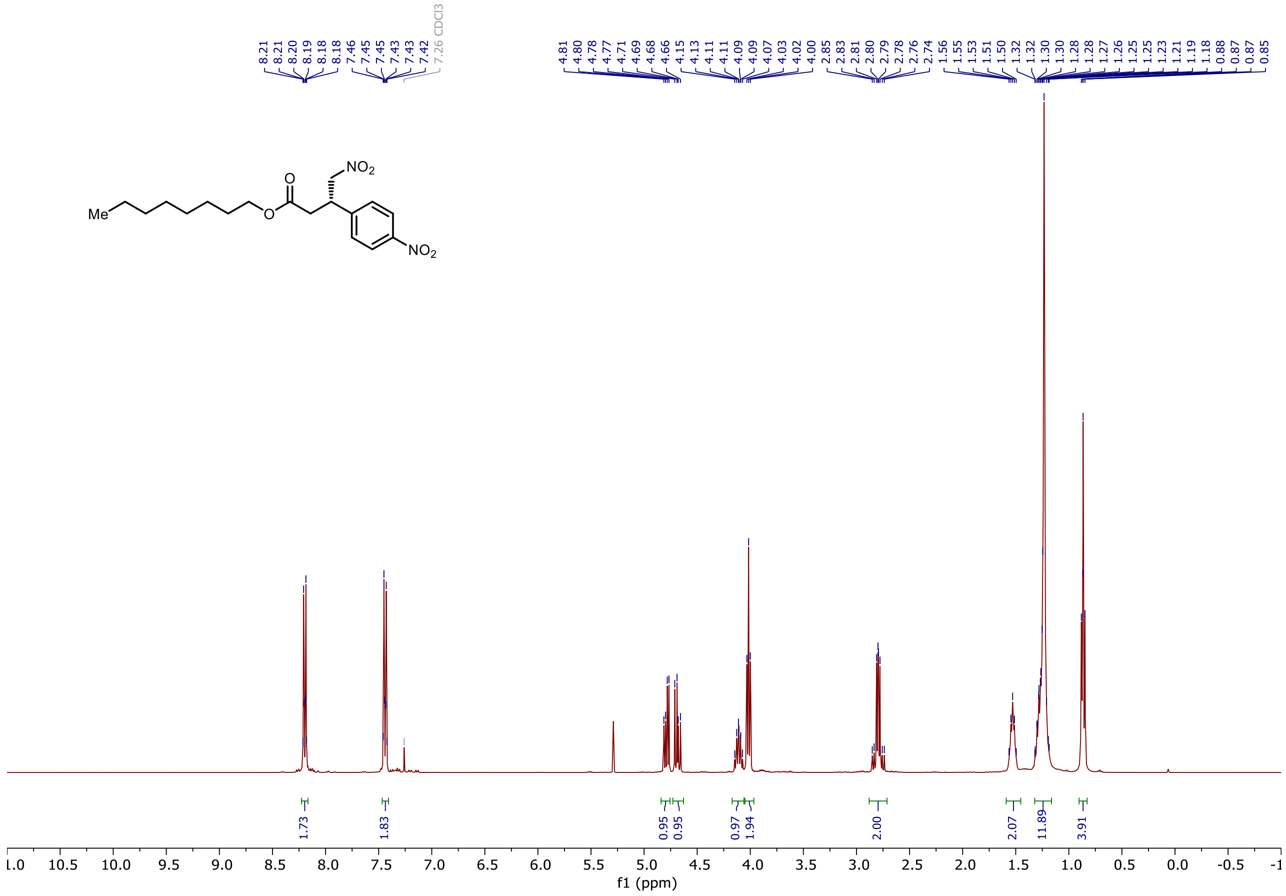

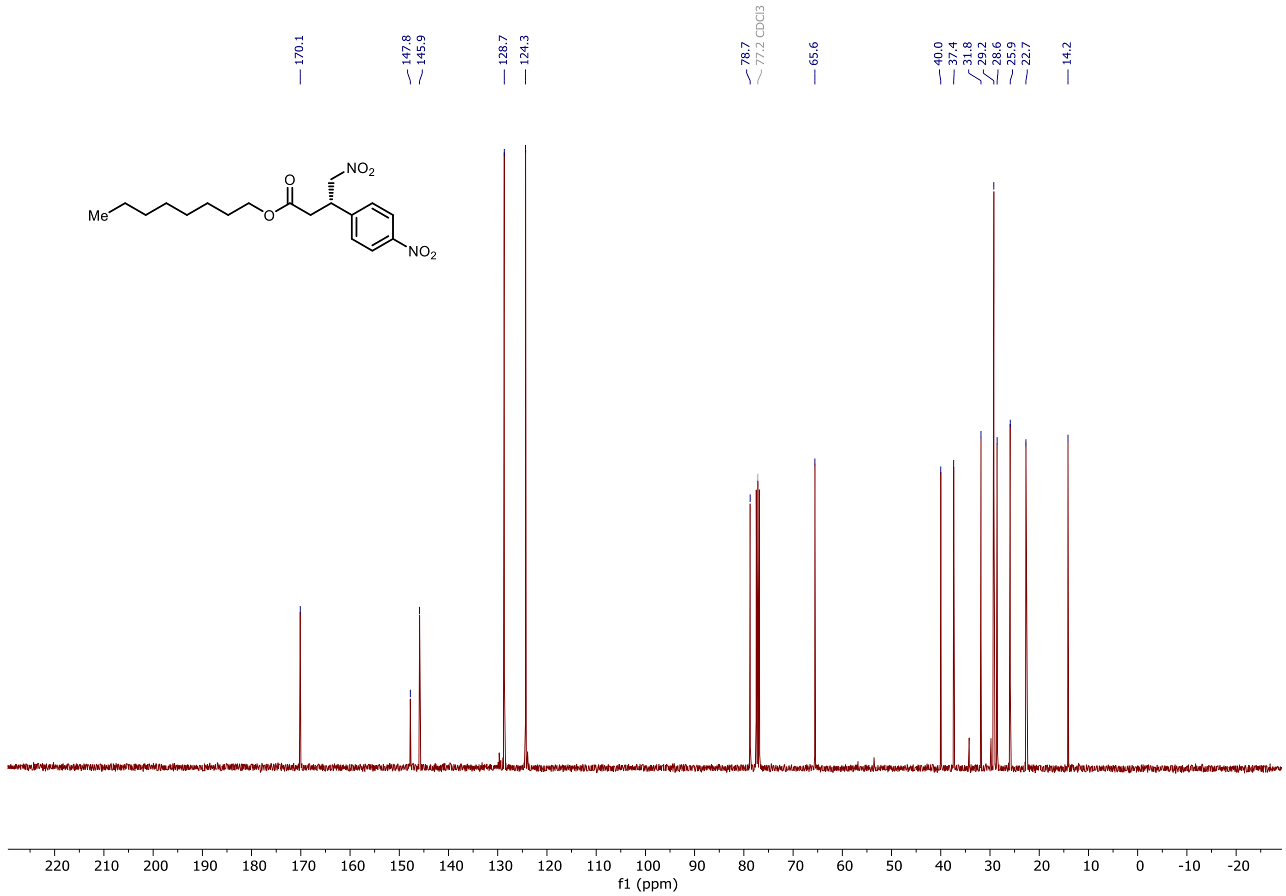

3t

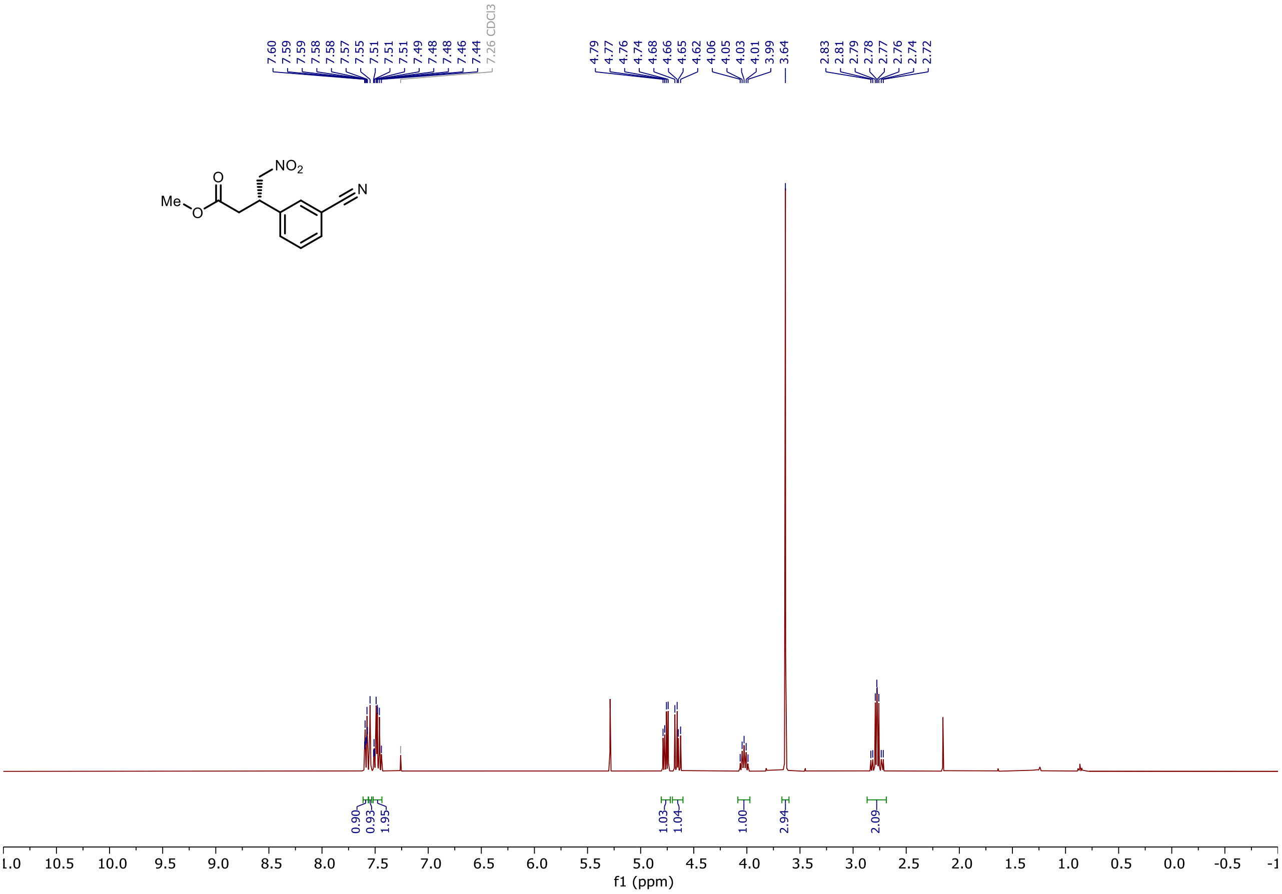

7.60 7.59 7.59 7.58 7.58 7.57 7.55 7.51 7.51 7.51 7.49 7.48 7.46 7.44 7.26 CDCl3
4.79 4.77 4.76 4.74 4.68 4.66 4.65 4.62 4.06 4.05 4.03 4.01 3.99 3.64
2.83 2.81 2.79 2.78 2.77 2.76 2.74 2.72
NO2
O
Me
O
N
0.90 0.93 1.95 1.03 1.04 1.00 2.94 2.09
f1 (ppm)

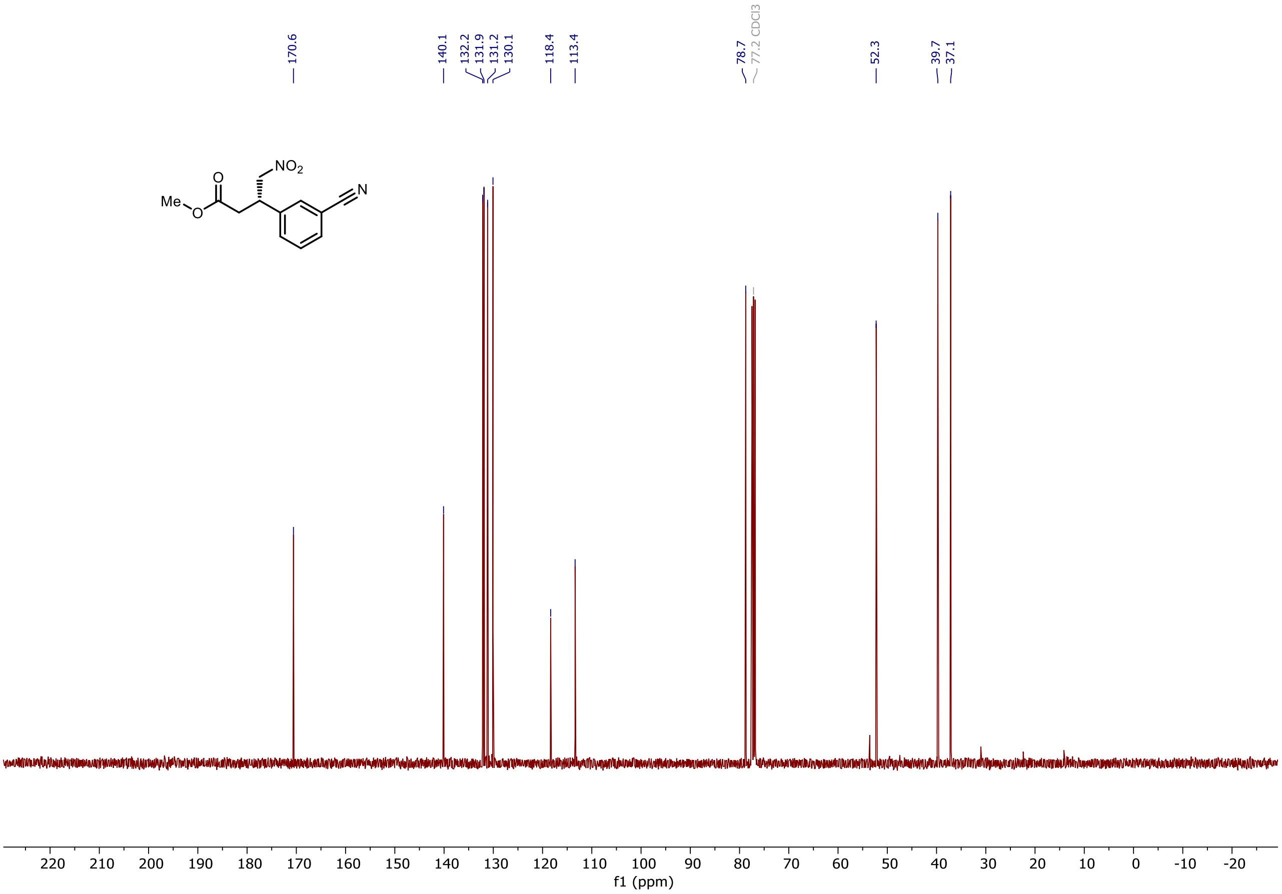

170.6 140.1 132.2 131.9 131.2 130.1 118.4 113.4 78.7 77.2 CDCl3 52.3 39.7 37.1
NO2
O
Me
O
N
f1 (ppm)

[[ToC]]

3u

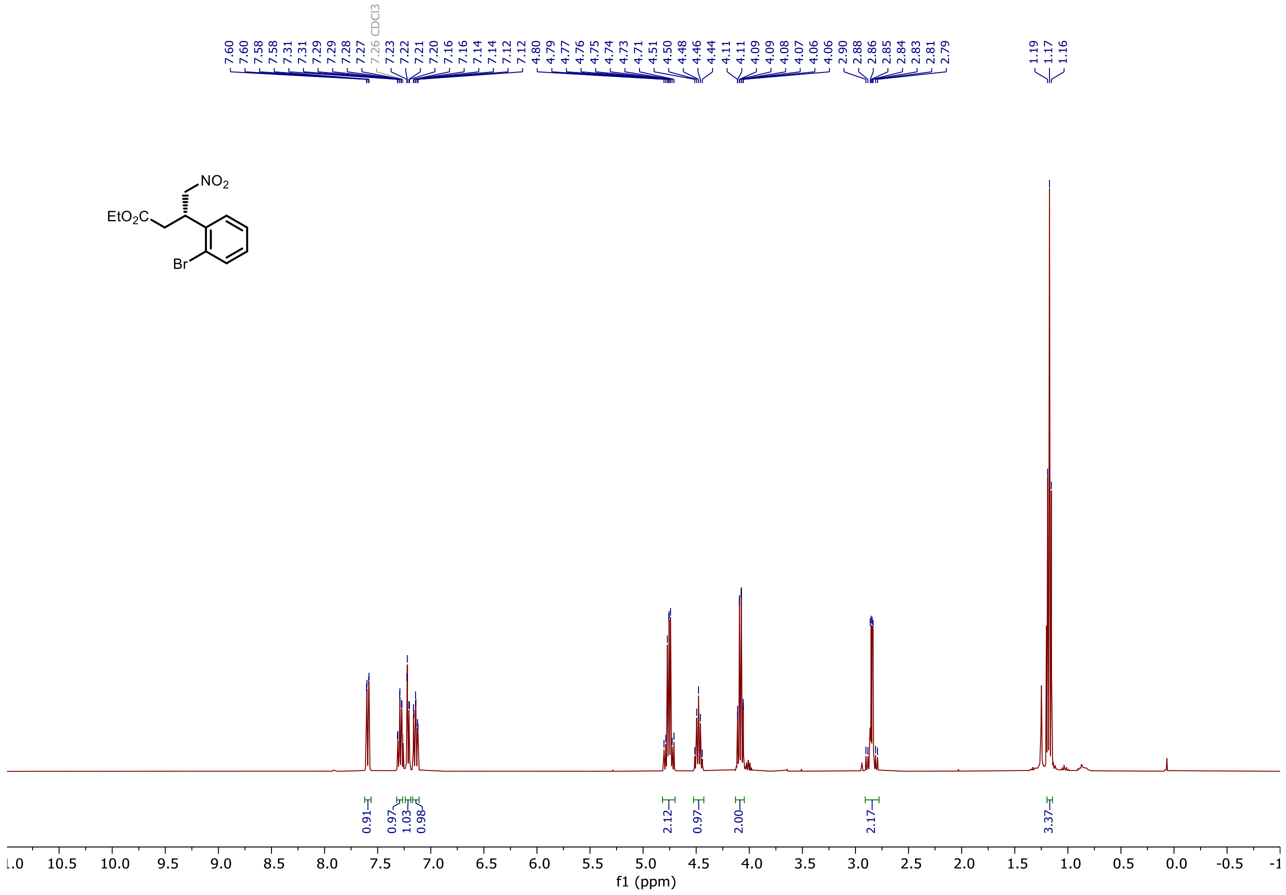

7.60 7.60 7.58 7.58 7.31 7.29 7.29 7.28 7.27 7.26 CDCl3 7.23 7.22 7.21 7.20 7.16 7.16 7.14 7.14 7.12 7.12 4.80 4.79 4.77 4.76 4.75 4.74 4.73 4.71 4.51 4.50 4.48 4.46 4.44 4.11 4.11 4.09 4.09 4.08 4.07 4.06 4.06 2.90 2.88 2.86 2.85 2.84 2.83 2.81 2.79 1.19 1.17 1.16
NO2
EtO2C
Br
0.91 0.97 1.03 0.98 2.12 0.97 2.00 2.17 3.37
f1 (ppm)

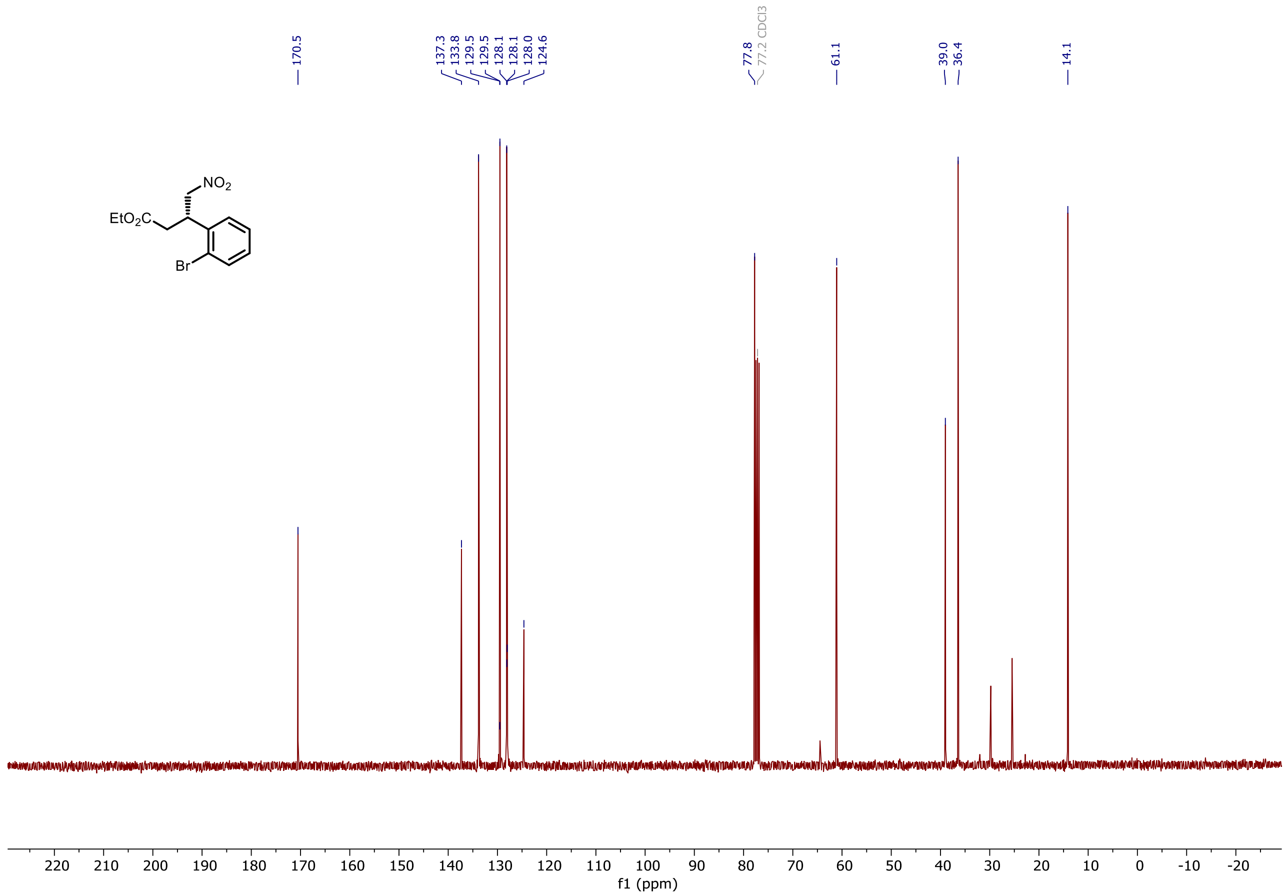

170.5 137.3 133.8 129.5 129.5 128.1 128.1 128.0 124.6 77.8 77.2 CDCl3 61.1 39.0 36.4 14.1
NO2
EtO2C
Br
f1 (ppm)

[ToC]

3v

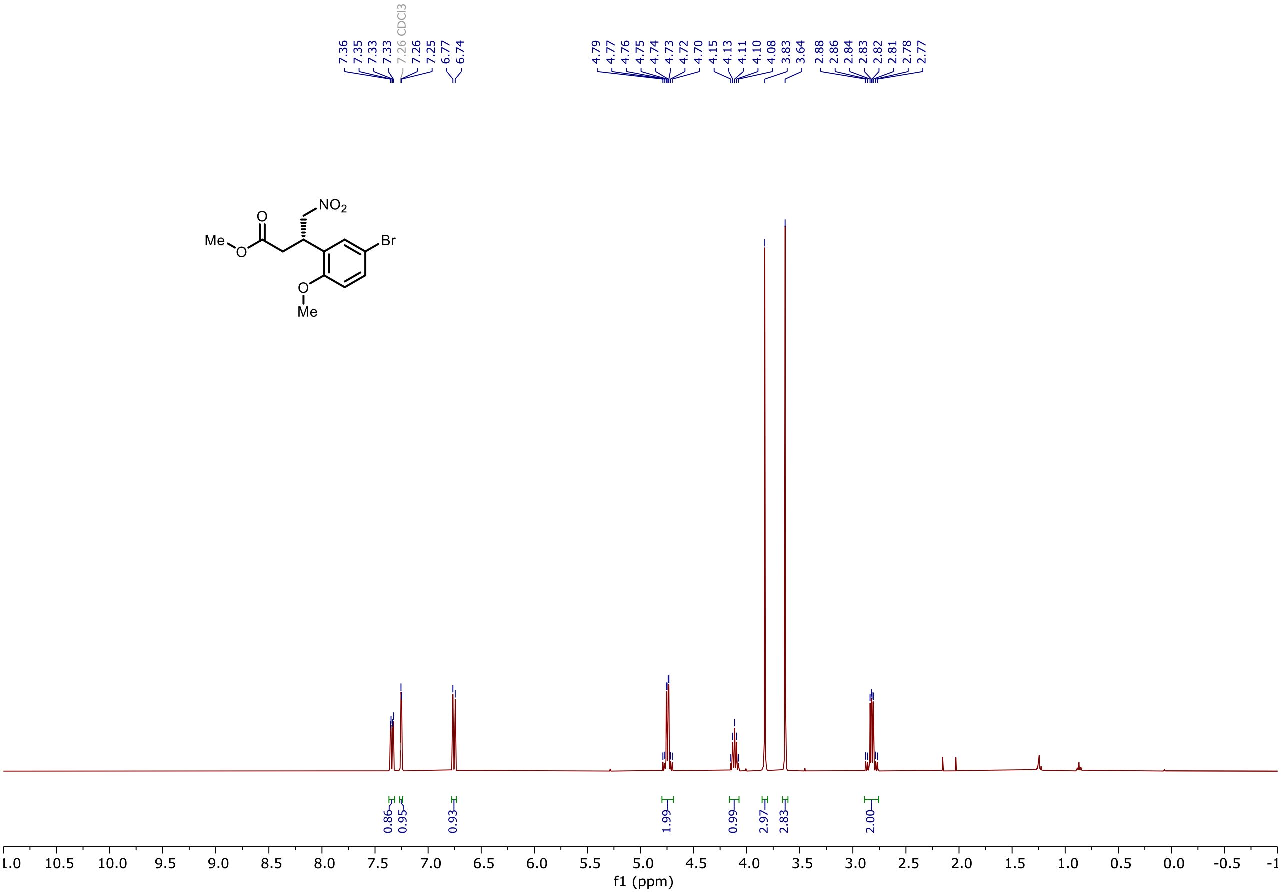

7.36
7.35
7.33
7.33
7.26 CDCl3
7.26
7.25
6.77
6.74
4.79
4.77
4.76
4.75
4.74
4.73
4.72
4.70
4.15
4.13
4.11
4.10
4.08
3.83
3.64
2.88
2.86
2.84
2.83
2.82
2.81
2.78
2.77
NO2
O
Me
O
Br
O
Me
0.86
0.95
0.93
1.99
0.99
2.97
2.83
2.00
f1 (ppm)

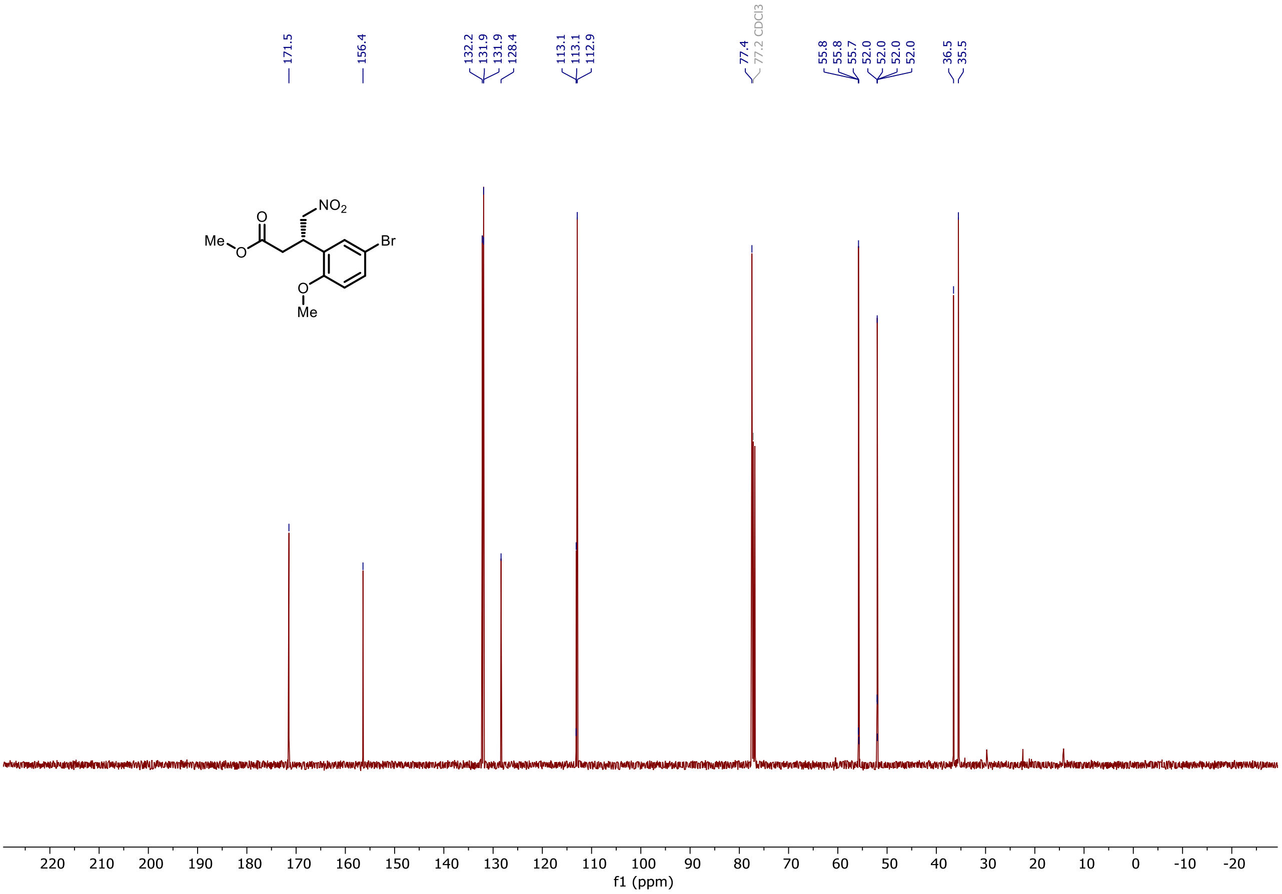

171.5
156.4
132.2
131.9
131.9
128.4
113.1
113.1
112.9
77.4
77.2 CDCl3
55.8
55.8
55.7
52.0
52.0
52.0
52.0
36.5
35.5
NO2
O
Me
O
Br
O
Me
f1 (ppm)

[ToC]

3w

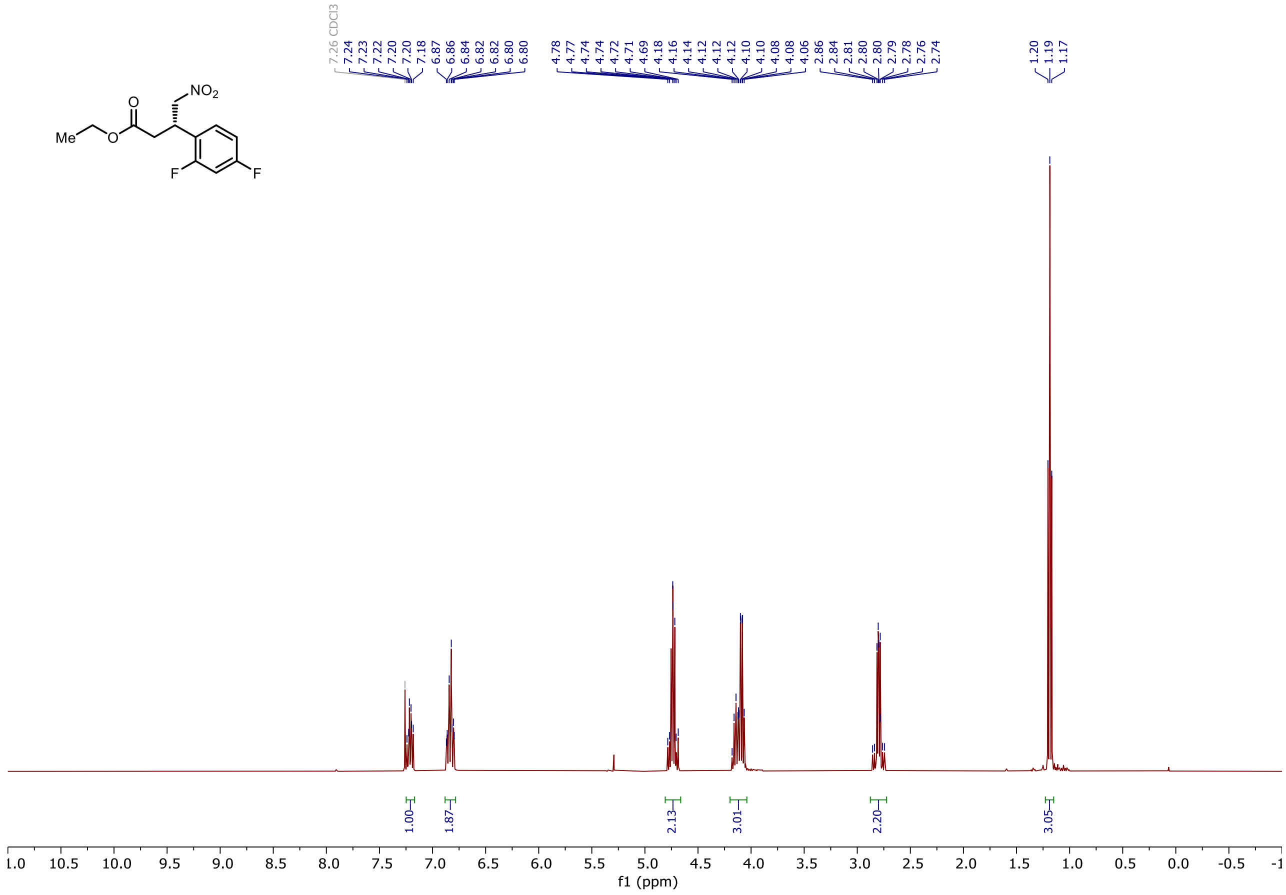

7.26 CDCl3
7.24
7.23
7.22
7.20
7.20
7.18
6.87
6.86
6.84
6.82
6.80
6.80
4.78
4.77
4.74
4.74
4.72
4.71
4.69
4.18
4.16
4.14
4.12
4.12
4.12
4.10
4.10
4.08
4.06
2.86
2.84
2.81
2.80
2.80
2.79
2.78
2.76
2.74
1.20
1.19
1.17
NO2
O
Me
O
F
F
1.00
1.87
2.13
3.01
2.20
3.05
f1 (ppm)

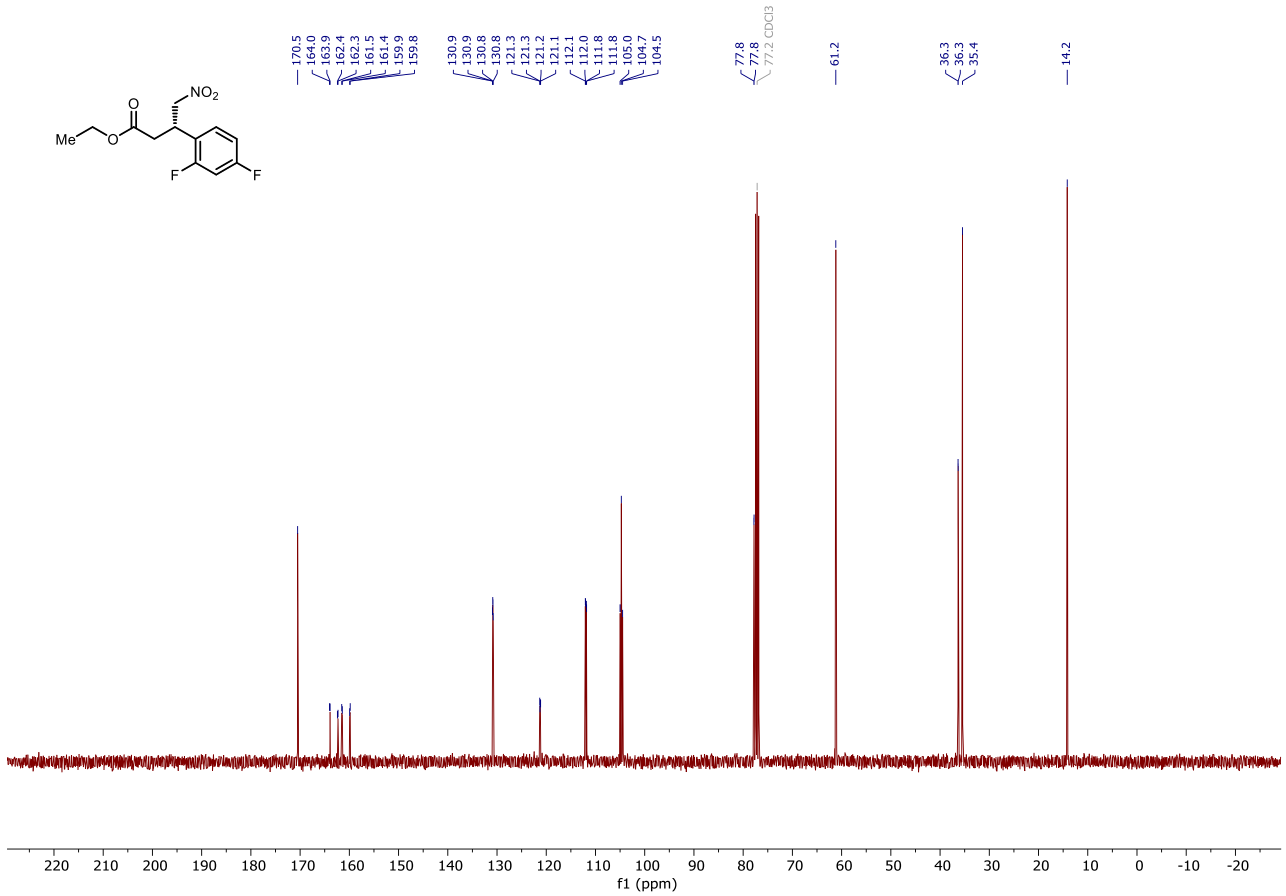

170.5
164.0
163.9
162.4
162.3
161.5
161.4
159.9
159.8
130.9
130.9
130.8
130.8
121.3
121.3
121.2
121.1
112.1
112.0
111.8
111.8
105.0
104.7
104.5
77.8
77.8
77.2 CDCl3
61.2
36.3
36.3
35.4
14.2
NO2
O
Me
O
F
F
f1 (ppm)

[ToC]

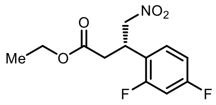

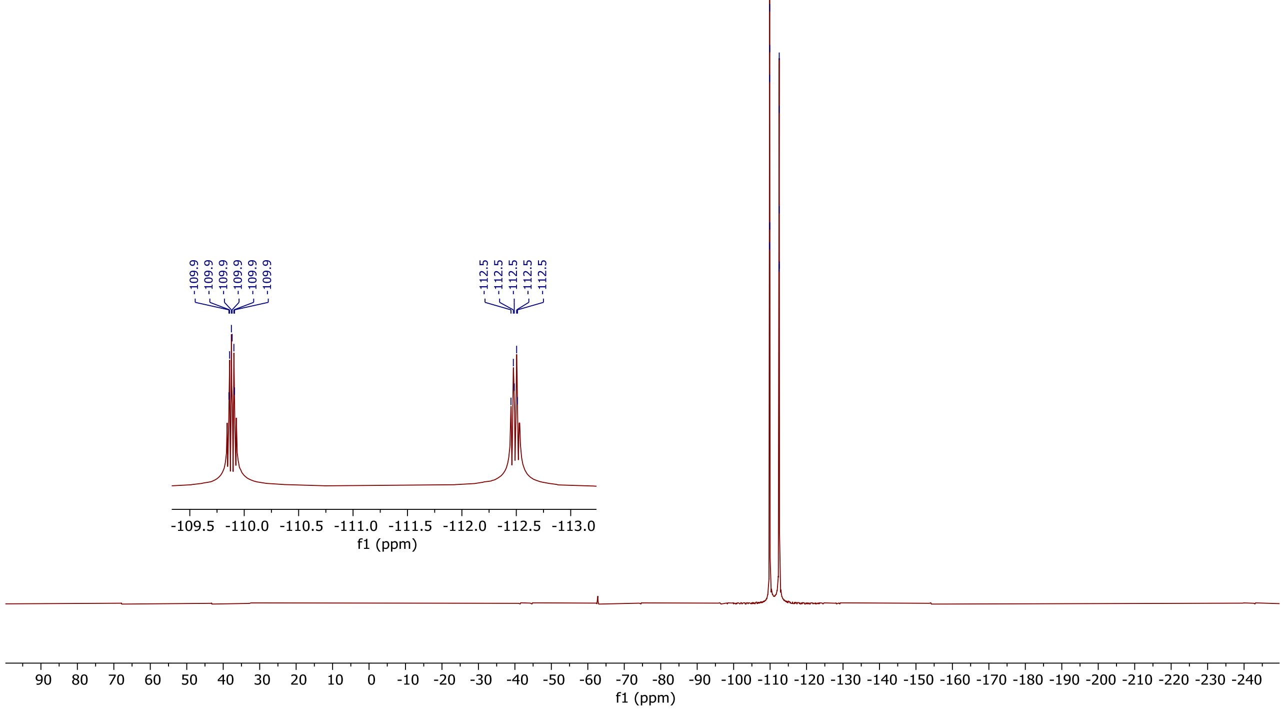

-109.9
-109.9
-109.9
-109.9
-109.9
-109.9
-112.5
-112.5
-112.5
-112.5
-112.5
-112.5
-109.5 -110.0 -110.5 -111.0 -111.5 -112.0 -112.5 -113.0
f1 (ppm)
90 80 70 60 50 40 30 20 10 0 -10 -20 -30 -40 -50 -60 -70 -80 -90 -100 -110 -120 -130 -140 -150 -160 -170 -180 -190 -200 -210 -220 -230 -240
f1 (ppm)

[ToC]

3x

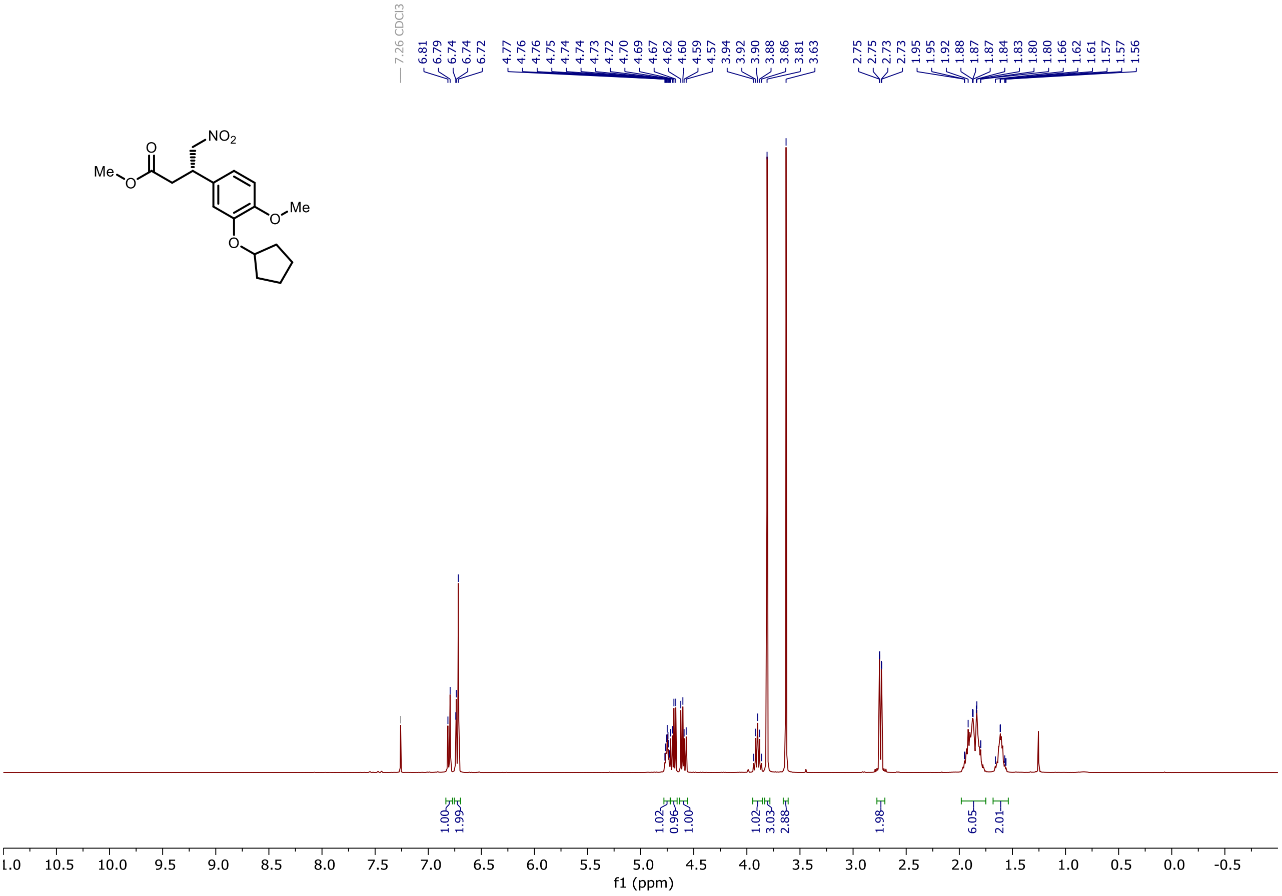
7.26 CDCl3
6.81
6.79
6.74
6.74
6.72
4.77
4.76
4.75
4.74
4.74
4.73
4.72
4.70
4.69
4.67
4.62
4.60
4.59
4.57
3.94
3.92
3.90
3.88
3.86
3.81
3.63
2.75
2.75
2.73
2.73
1.95
1.95
1.92
1.88
1.87
1.87
1.84
1.83
1.80
1.80
1.66
1.62
1.61
1.57
1.56
NO2
O
Me
O
O
Me
O
1.00
1.99
1.02
0.96
1.00
1.02
3.03
2.88
1.98
6.05
2.01
f1 (ppm)

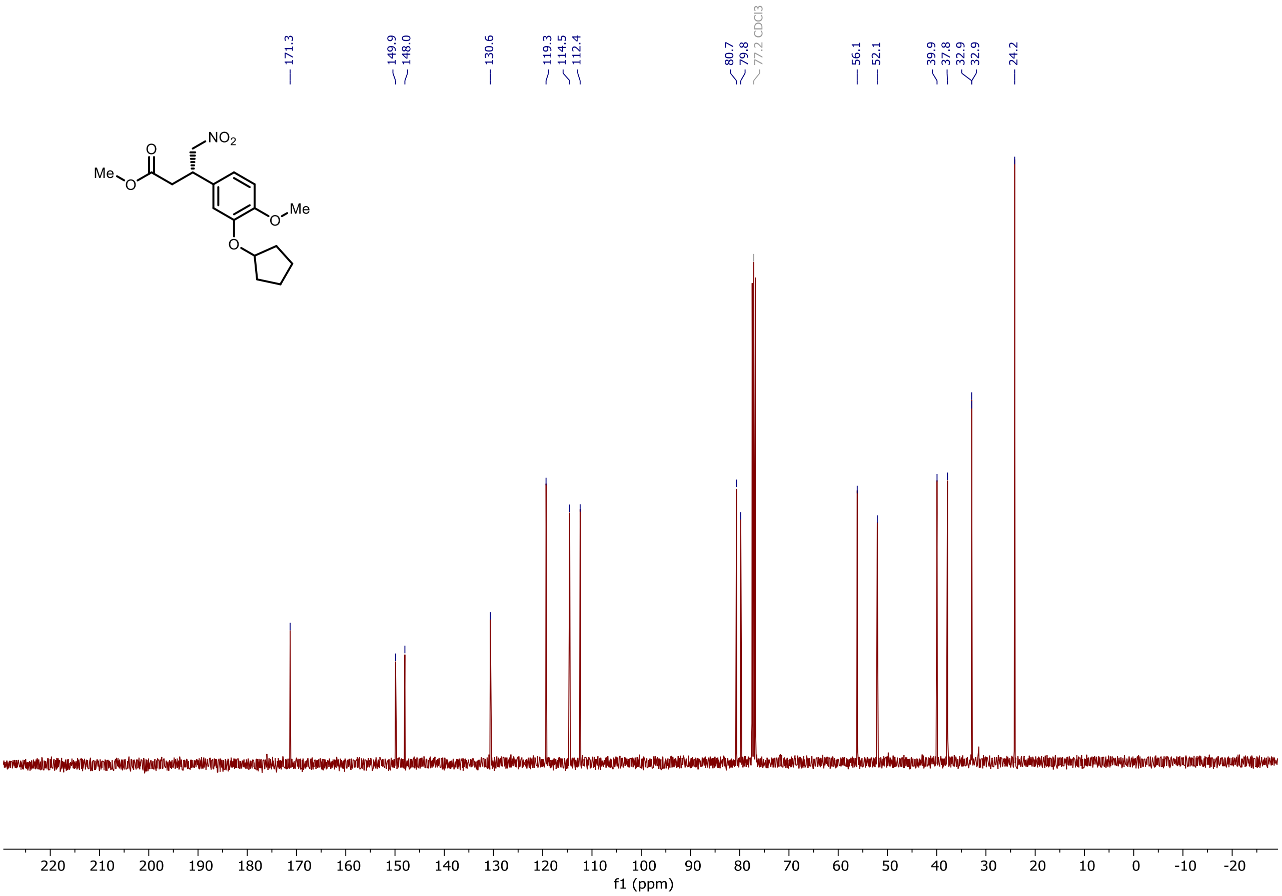
171.3
149.9
148.0
130.6
119.3
114.5
112.4
80.7
79.8
77.2 CDCl3
56.1
52.1
39.9
37.8
32.9
32.9
24.2
NO2
O
Me
O
O
Me
O
f1 (ppm)

[ToC]

3y

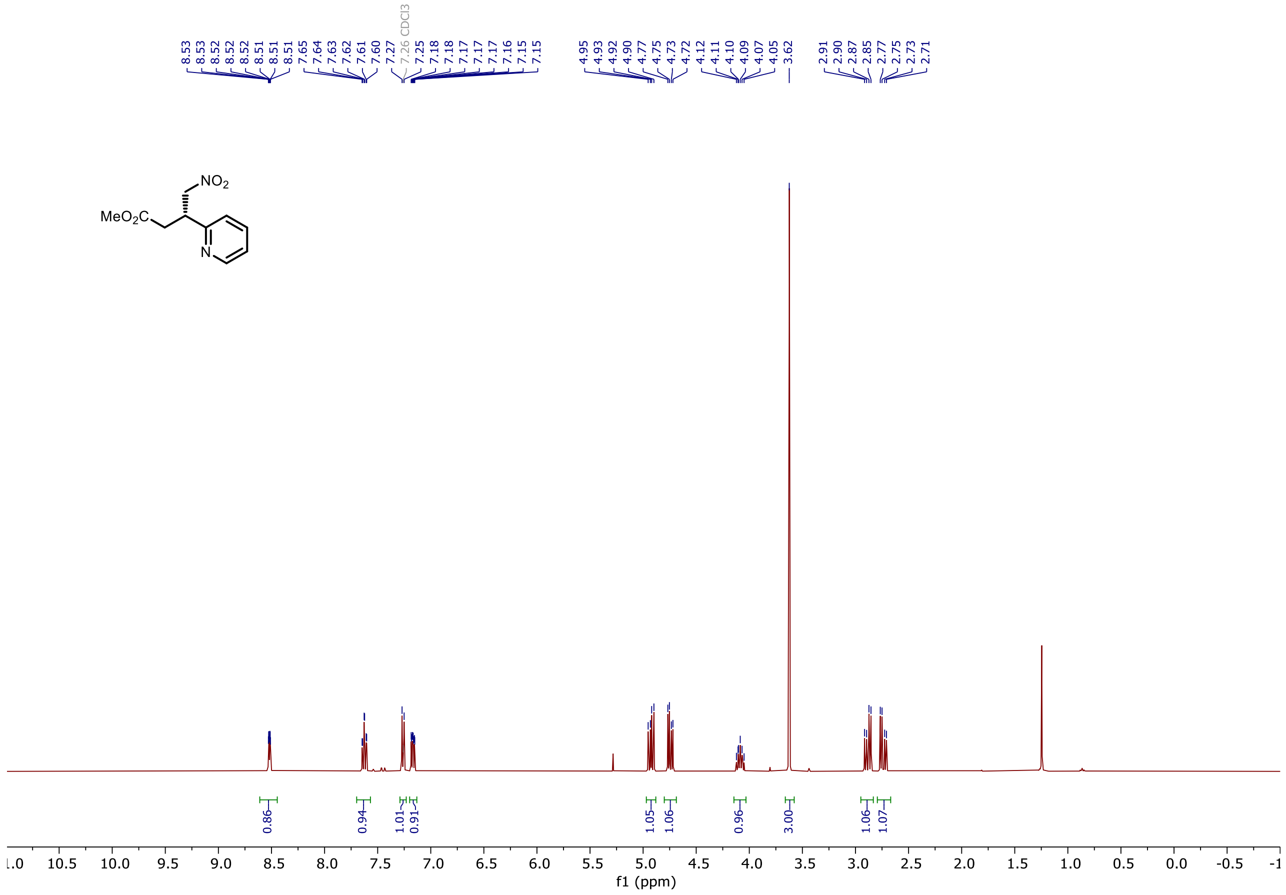

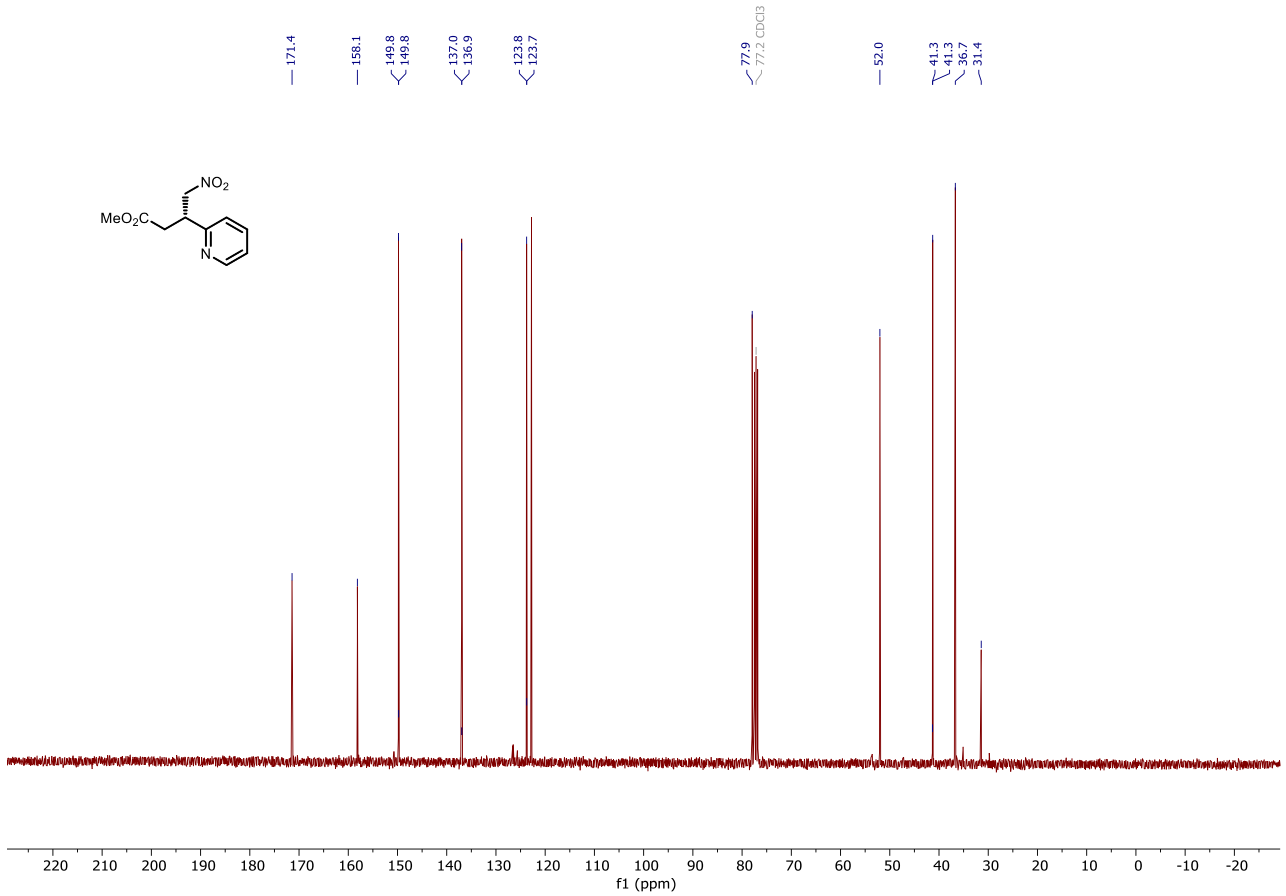

[ToC]

3z

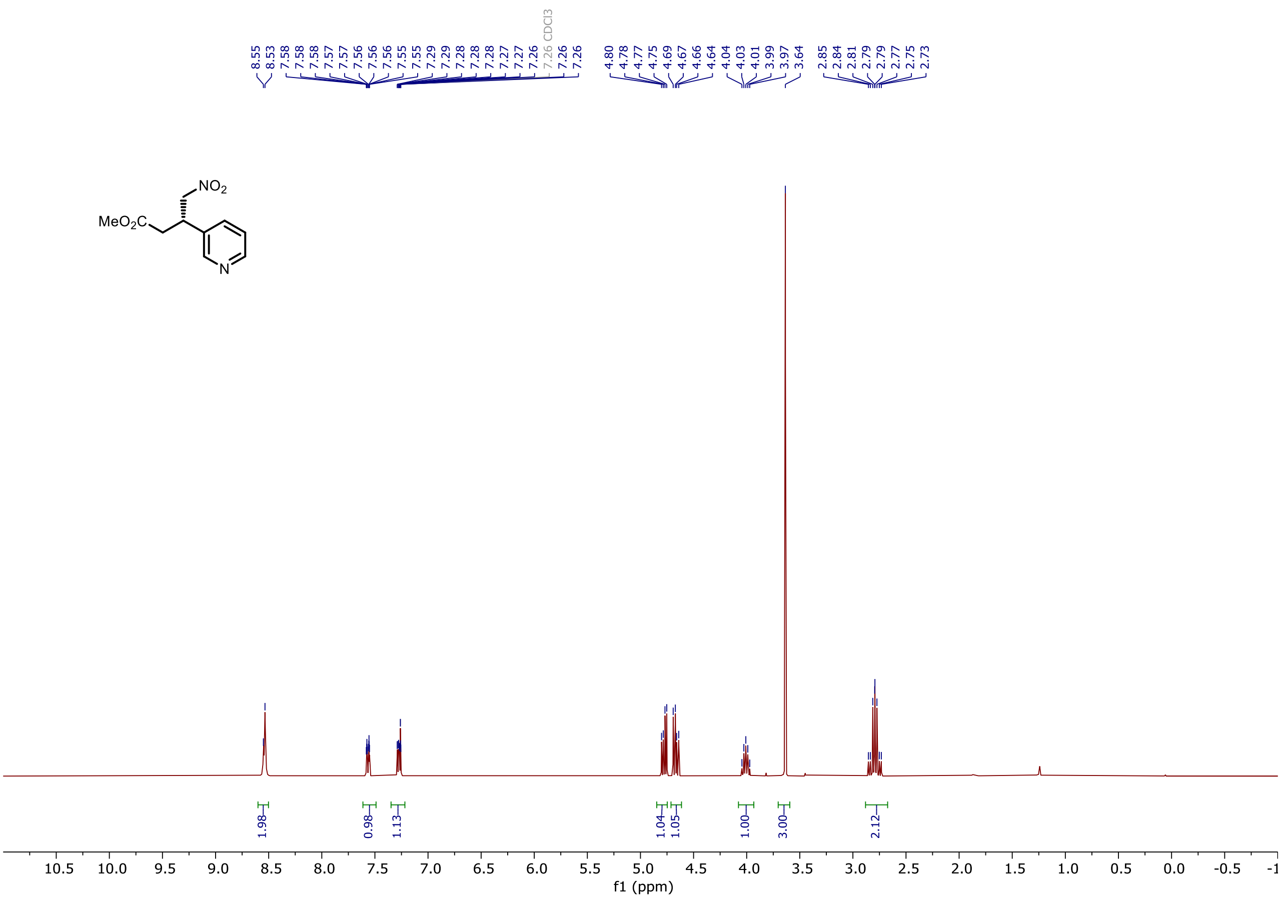

8.55
8.53
7.58
7.58
7.58
7.57
7.57
7.56
7.56
7.56
7.55
7.55
7.29
7.29
7.28
7.28
7.28
7.27
7.27
7.26
7.26 CDCl3
7.26
7.26
4.80
4.78
4.77
4.75
4.69
4.67
4.66
4.64
4.04
4.03
4.01
3.99
3.97
3.64
2.85
2.84
2.81
2.79
2.79
2.77
2.75
2.73
NO2
MeO2C
N
1.98
0.98
1.13
1.04
1.05
1.00
3.00
2.12
f1 (ppm)

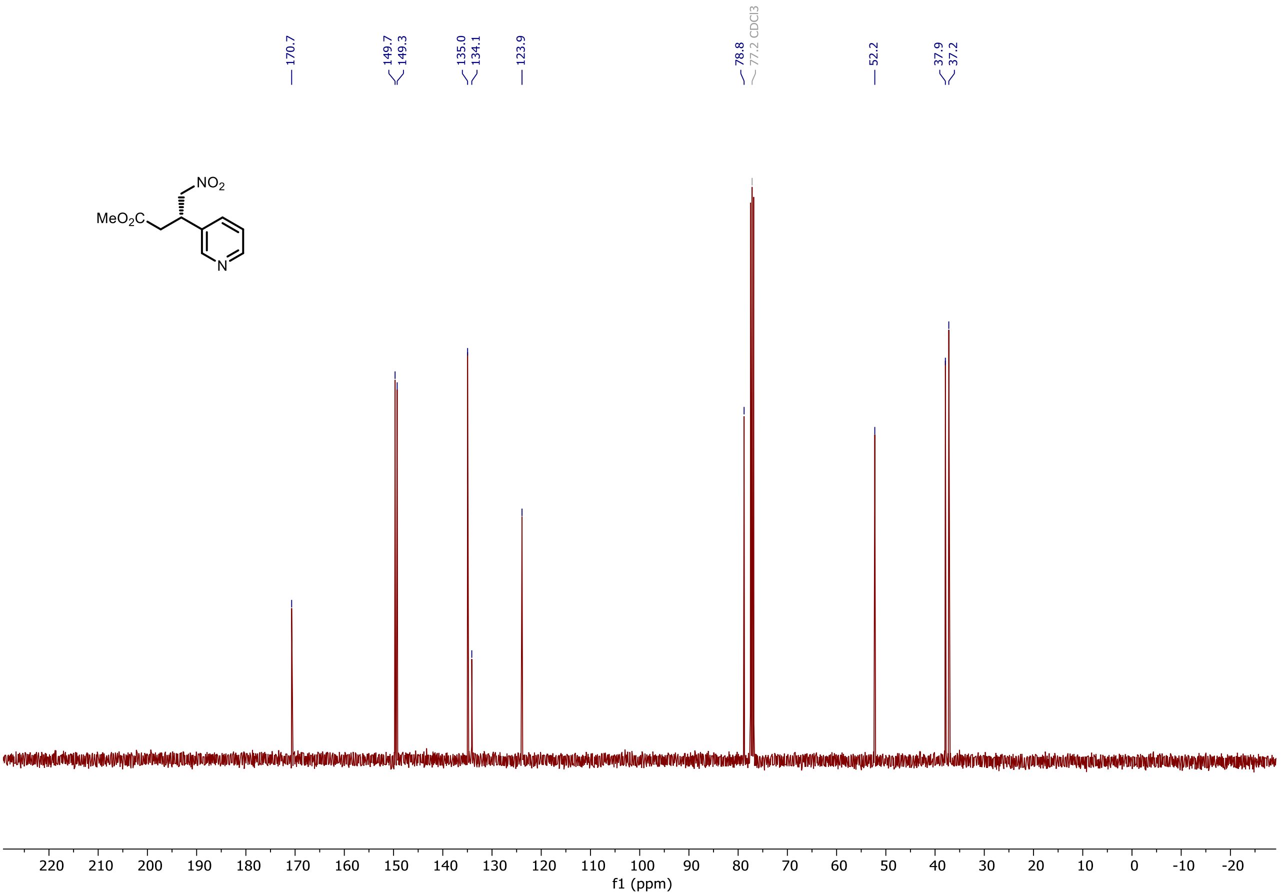

170.7
149.7
149.3
135.0
134.1
123.9
78.8
77.2 CDCl3
52.2
37.9
37.2
NO2
MeO2C
N
f1 (ppm)

[ToC]

3aa

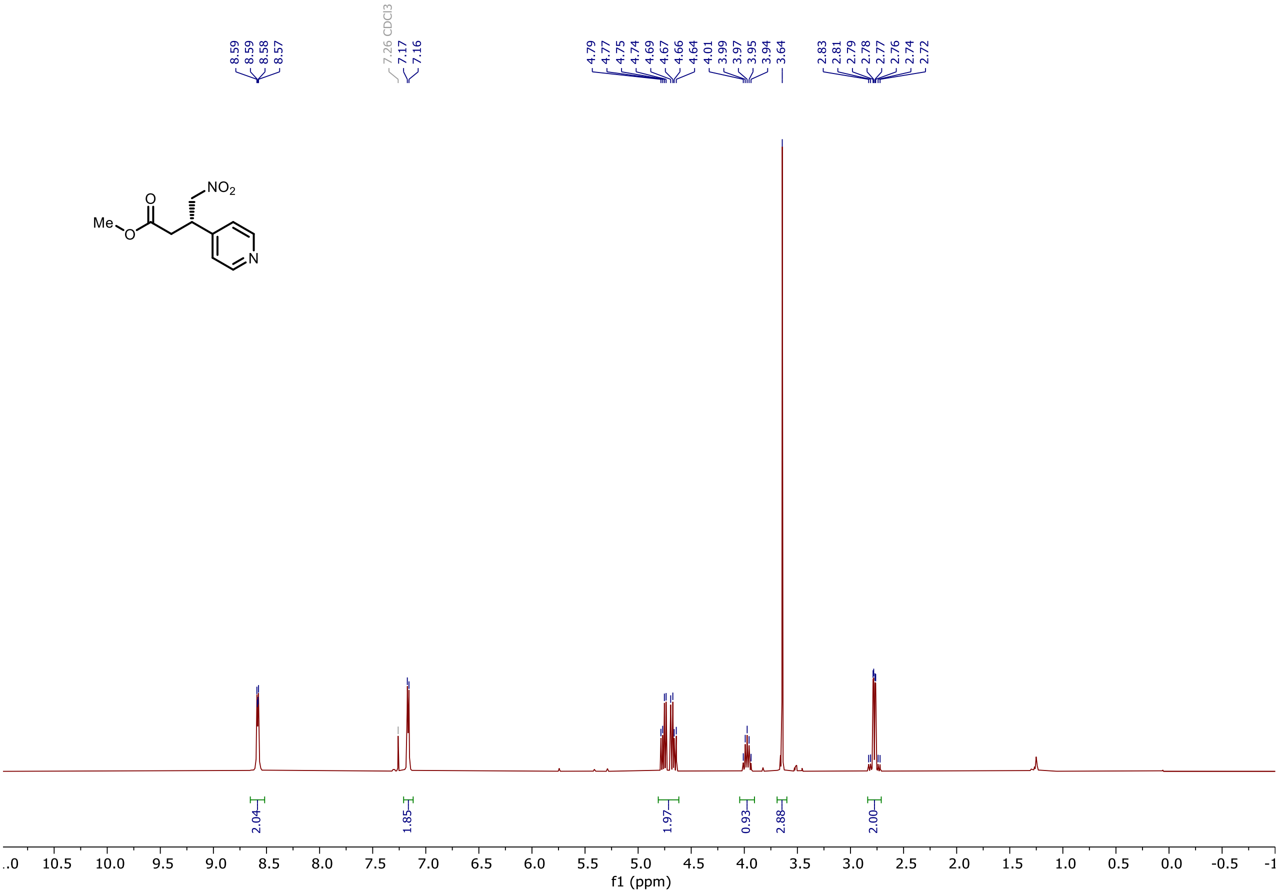

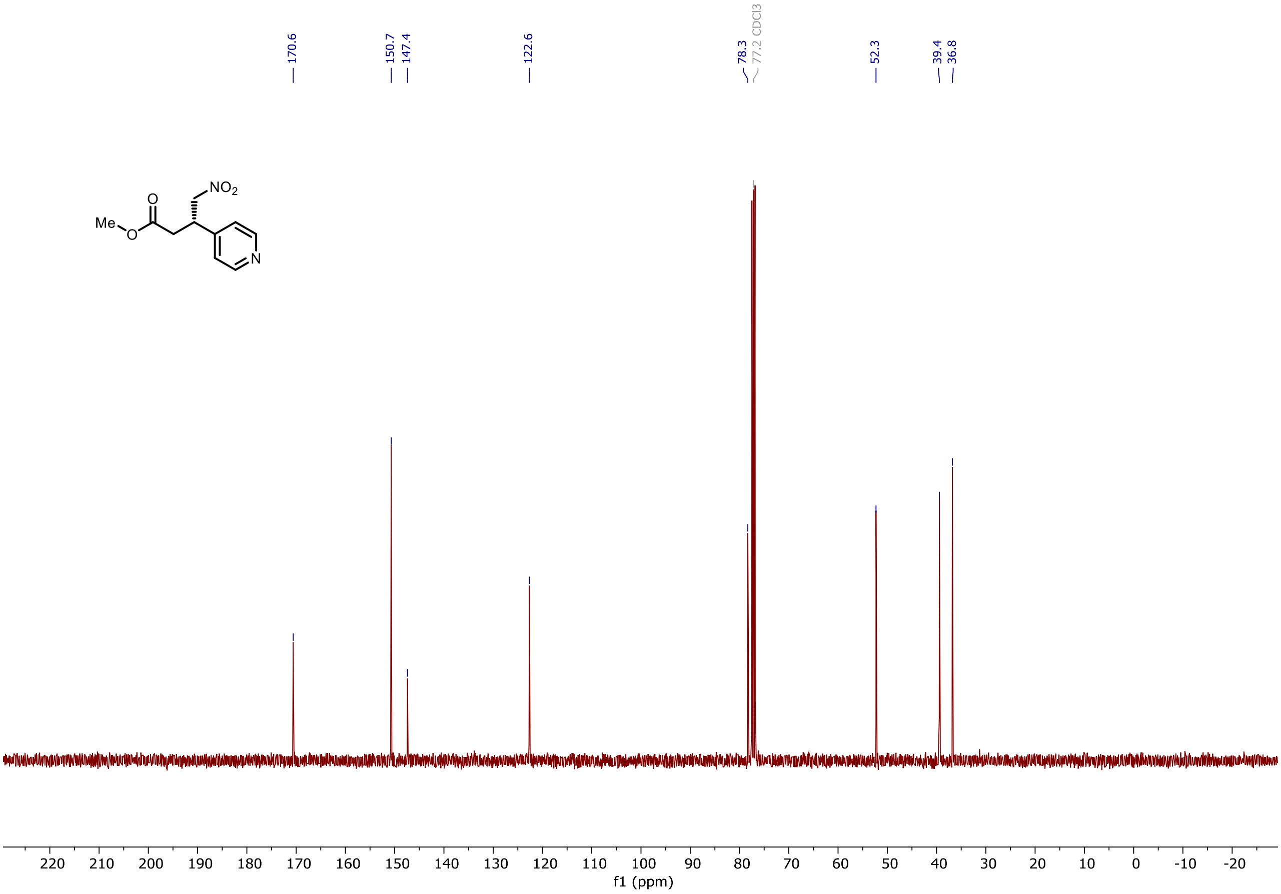

[ToC]

3ab

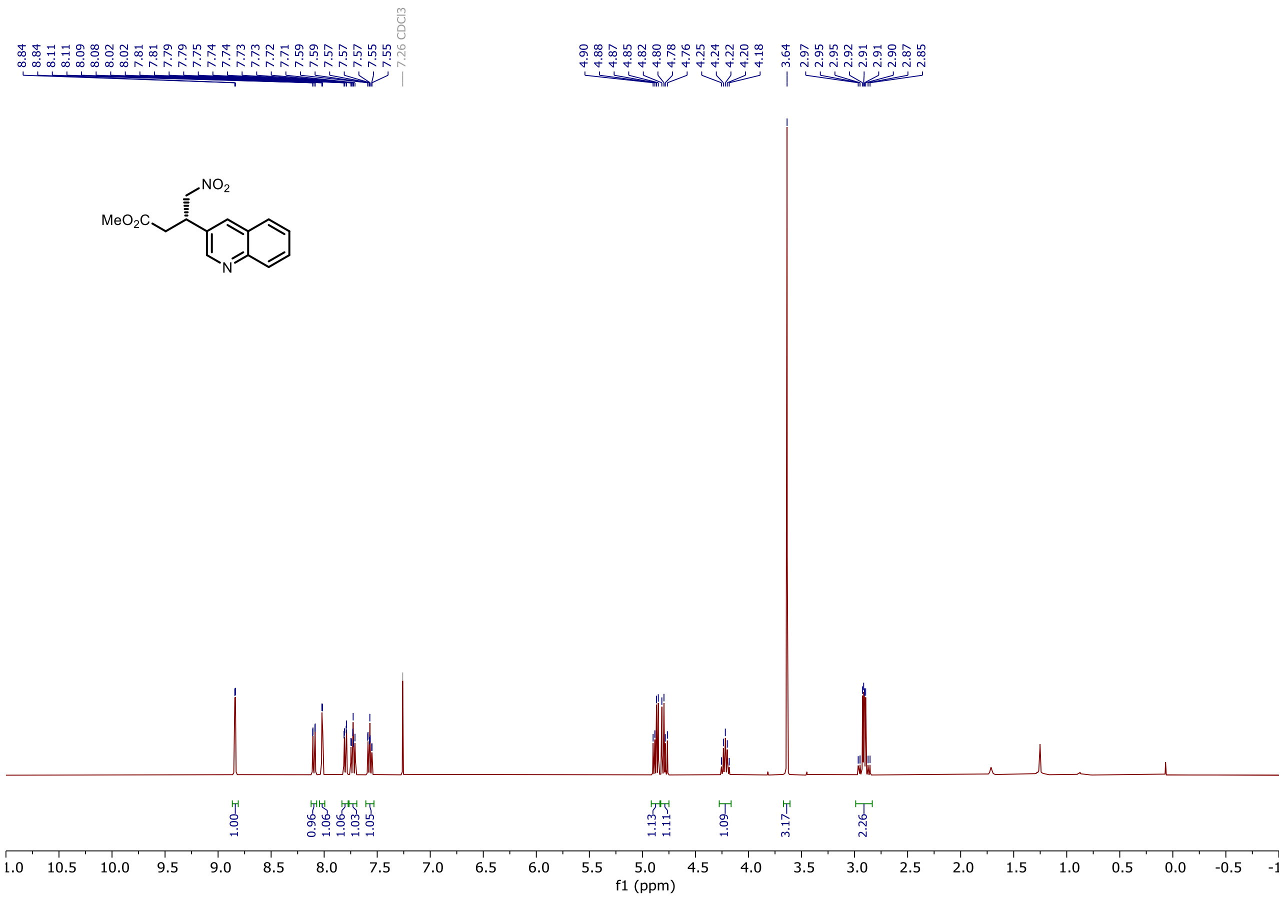
8.84
8.84
8.11
8.11
8.09
8.08
8.02
8.02
7.81
7.81
7.79
7.79
7.75
7.74
7.74
7.73
7.73
7.72
7.71
7.59
7.59
7.57
7.57
7.57
7.55
7.55
7.26 CDCl3
4.90
4.88
4.87
4.85
4.82
4.80
4.78
4.76
4.25
4.24
4.22
4.20
4.18
3.64
2.97
2.95
2.95
2.92
2.91
2.91
2.90
2.87
2.85
NO_2
MeO_2C
N
1.00
0.96
1.06
1.06
1.03
1.05
1.13
1.11
1.09
3.17
2.26
f1 (ppm)

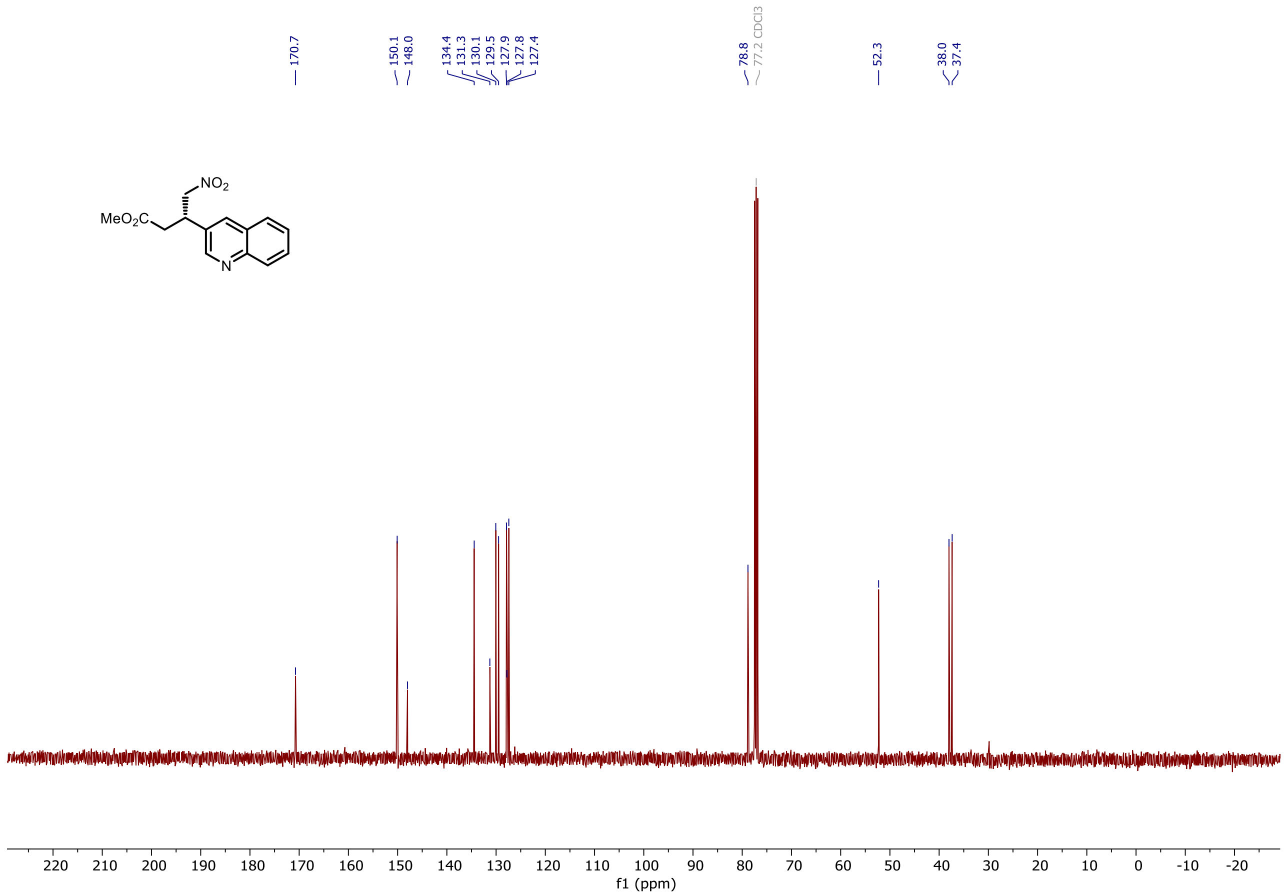
170.7
150.1
148.0
134.4
131.3
130.1
129.5
127.9
127.8
127.4
78.8
77.2 CDCl3
52.3
38.0
37.4
NO_2
MeO_2C
N
f1 (ppm)

[ToC]

3ac

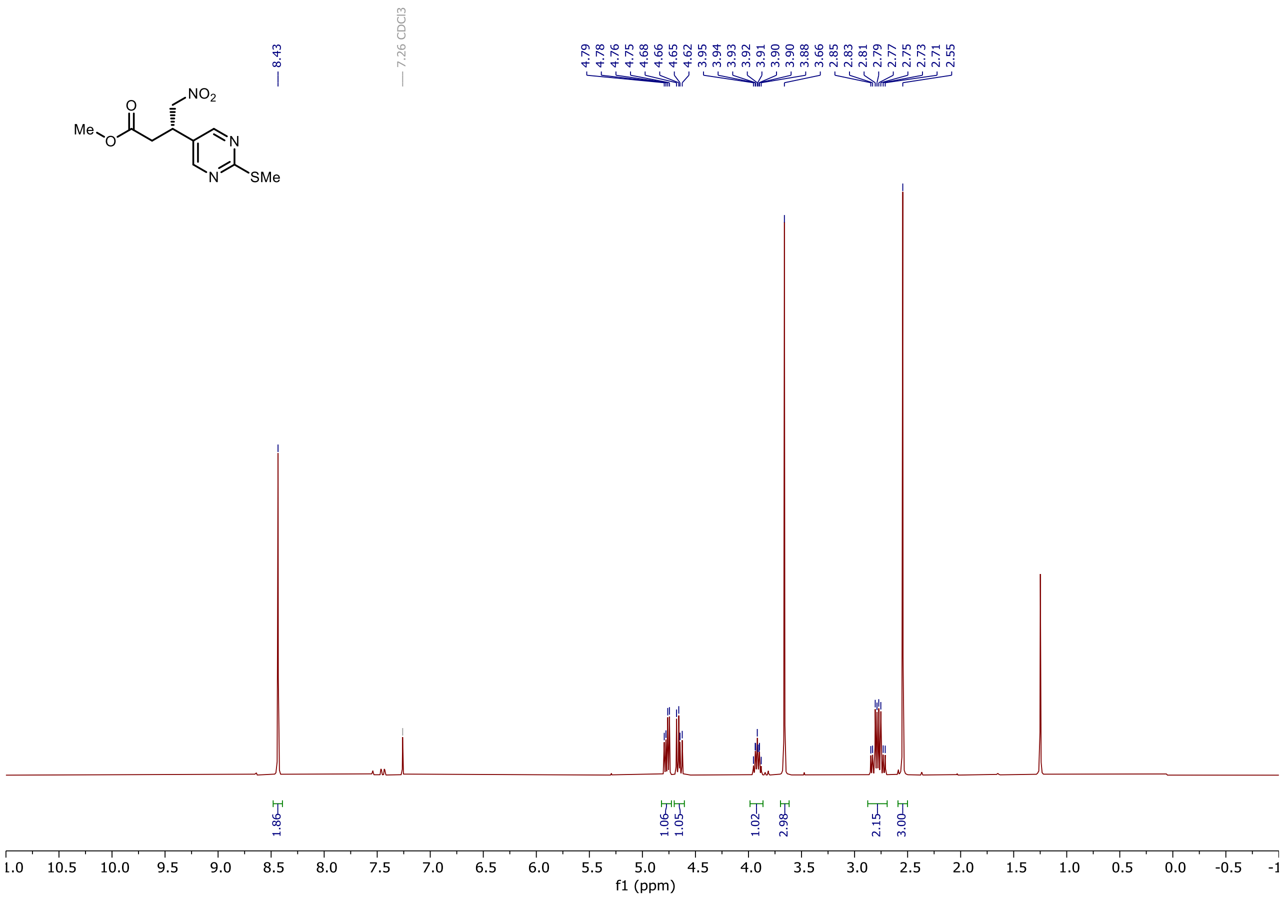

NO2
O
Me
O
N
N
SMe
8.43
7.26 CDCl3
4.79
4.78
4.76
4.75
4.68
4.66
4.65
4.62
3.95
3.94
3.93
3.92
3.91
3.90
3.90
3.88
3.66
2.85
2.83
2.81
2.79
2.77
2.75
2.73
2.71
2.55
1.86
1.06
1.05
1.02
2.98
2.15
3.00
11.0 10.5 10.0 9.5 9.0 8.5 8.0 7.5 7.0 6.5 6.0 5.5 5.0 4.5 4.0 3.5 3.0 2.5 2.0 1.5 1.0 0.5 0.0 -0.5 -1
f1 (ppm)

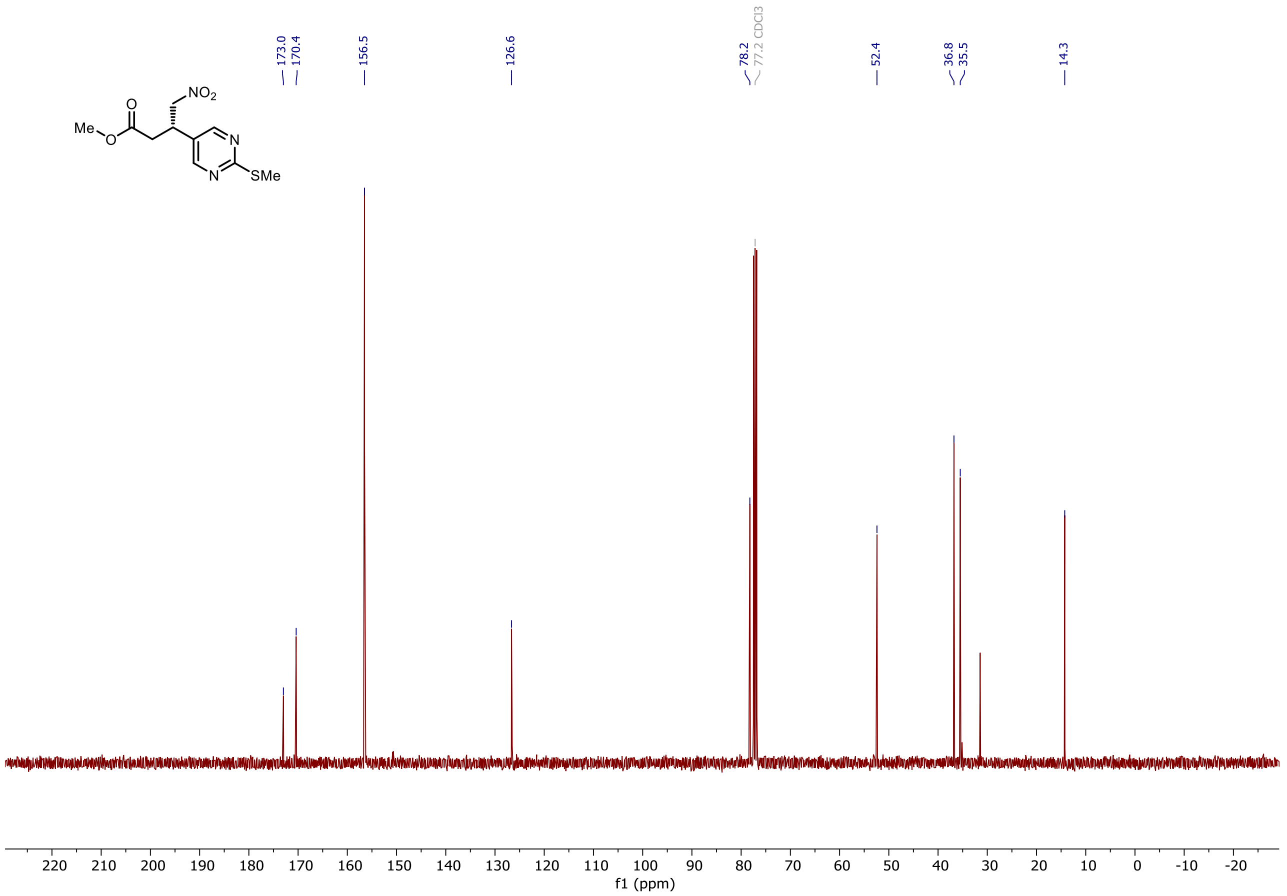

NO2
O
Me
O
N
N
SMe
173.0
170.4
156.5
126.6
78.2
77.2 CDCl3
52.4
36.8
35.5
14.3
220 210 200 190 180 170 160 150 140 130 120 110 100 90 80 70 60 50 40 30 20 10 0 -10 -20
f1 (ppm)

3ad

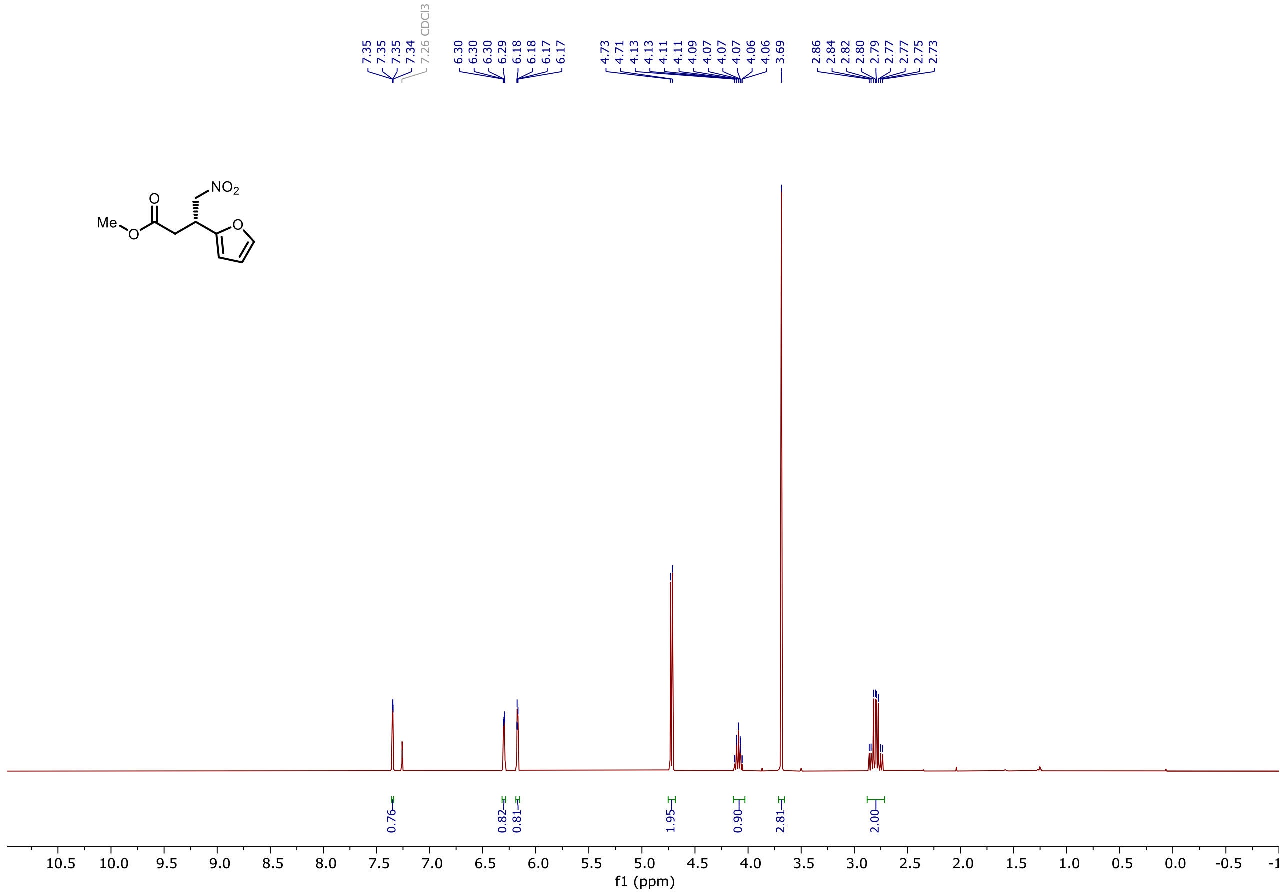

7.35
7.35
7.35
7.34
7.26 CDCl3
6.30
6.30
6.30
6.29
6.18
6.18
6.17
6.17
4.73
4.71
4.13
4.13
4.11
4.11
4.09
4.07
4.07
4.07
4.06
4.06
3.69
2.86
2.84
2.82
2.80
2.79
2.77
2.77
2.75
2.73
NO2
O
Me
O
O
0.76
0.82
0.81
1.95
0.90
2.81
2.00
f1 (ppm)

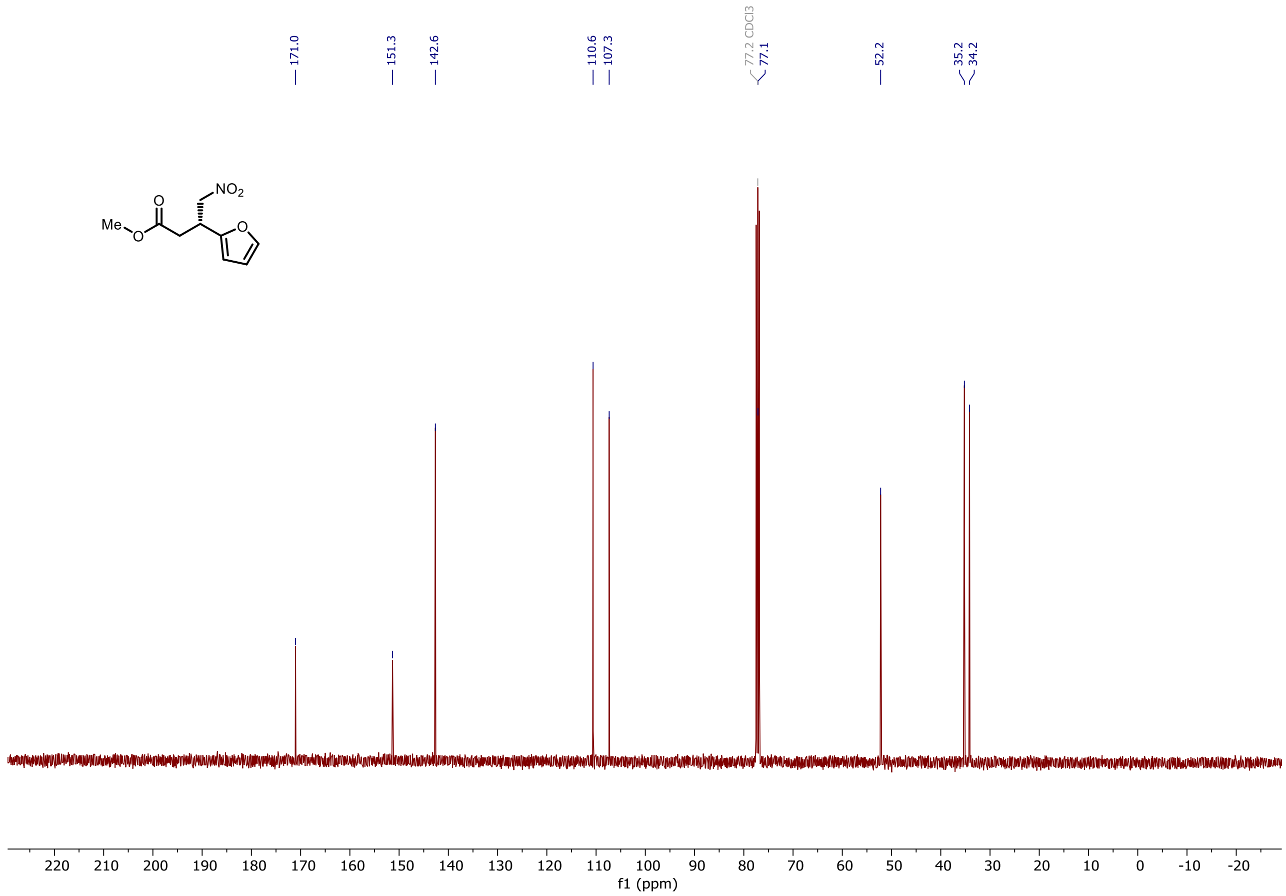

171.0
151.3
142.6
110.6
107.3
77.2 CDCl3
77.1
52.2
35.2
34.2
NO2
O
Me
O
O
f1 (ppm)

[ToC]

3ae

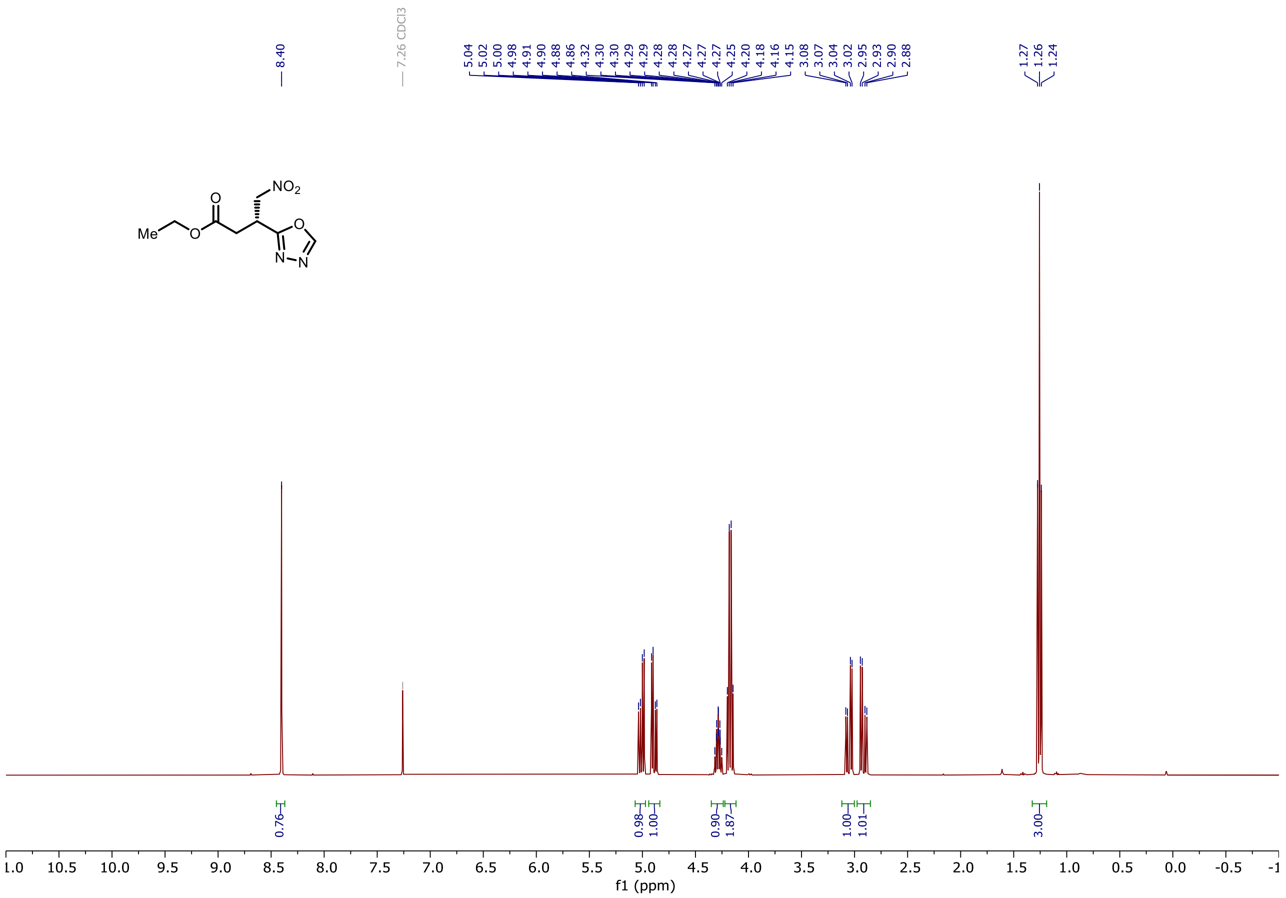

8.40
7.26 CDCl3
5.04 5.02 5.00 4.98 4.91 4.90 4.88 4.86 4.32 4.30 4.30 4.29 4.29 4.28 4.28 4.27 4.27 4.27 4.25 4.20 4.18 4.16 4.15 3.08 3.07 3.04 3.02 2.95 2.93 2.90 2.88
1.27 1.26 1.24
NO_2
O
Me
O
O
N
N
0.76
0.98 1.00
0.90 1.87
1.00 1.01
3.00
f1 (ppm)

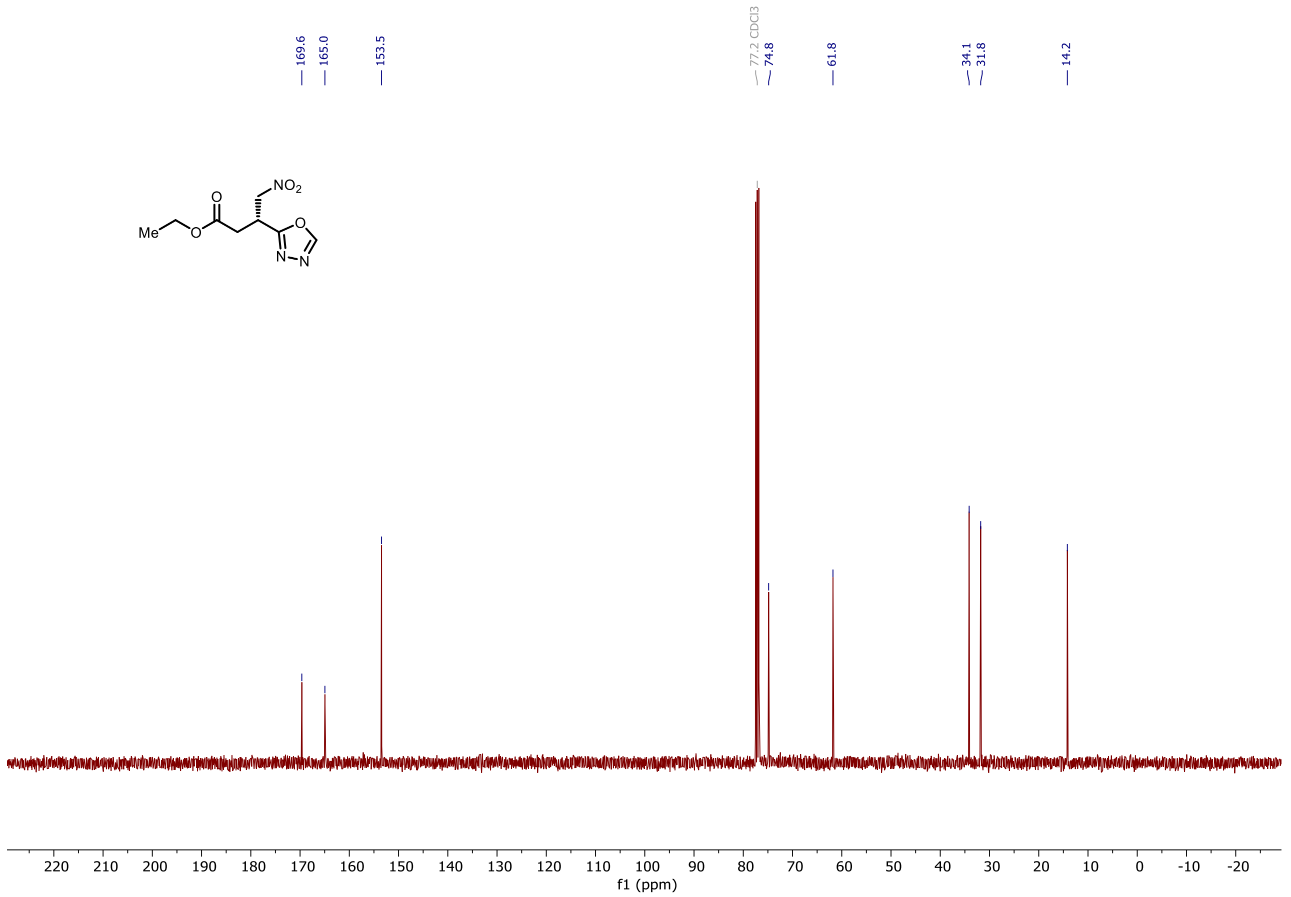

169.6
165.0
153.5
77.2 CDCl3
74.8
61.8
34.1
31.8
14.2
NO_2
O
Me
O
O
N
N
f1 (ppm)

[ToC]

3af

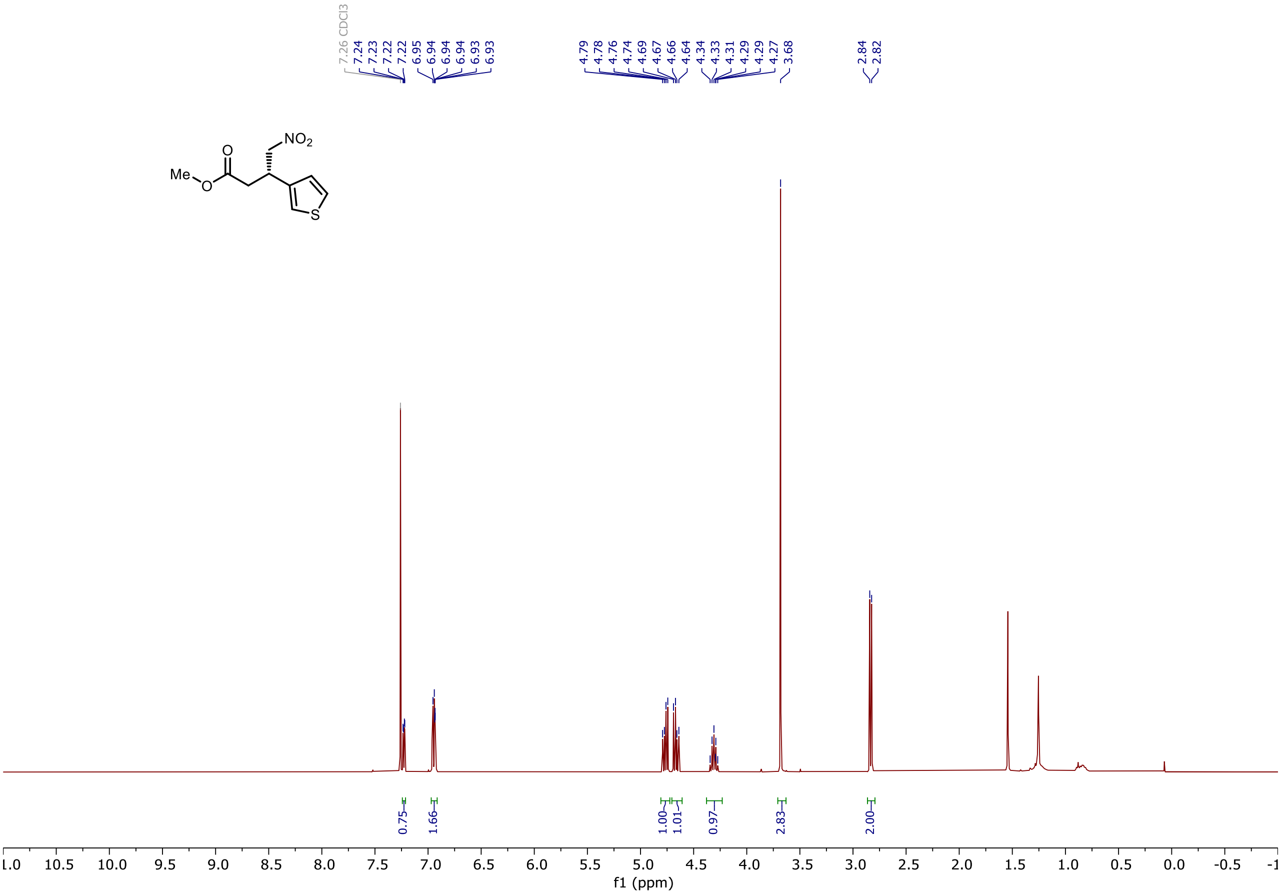
7.26 CDCl3
7.24
7.23
7.22
7.22
6.95
6.94
6.94
6.94
6.93
6.93
4.79
4.78
4.76
4.74
4.69
4.67
4.66
4.64
4.34
4.33
4.31
4.29
4.29
4.27
3.68
2.84
2.82
NO2
O
Me
O
S
0.75
1.66
1.00
1.01
0.97
2.83
2.00
f1 (ppm)

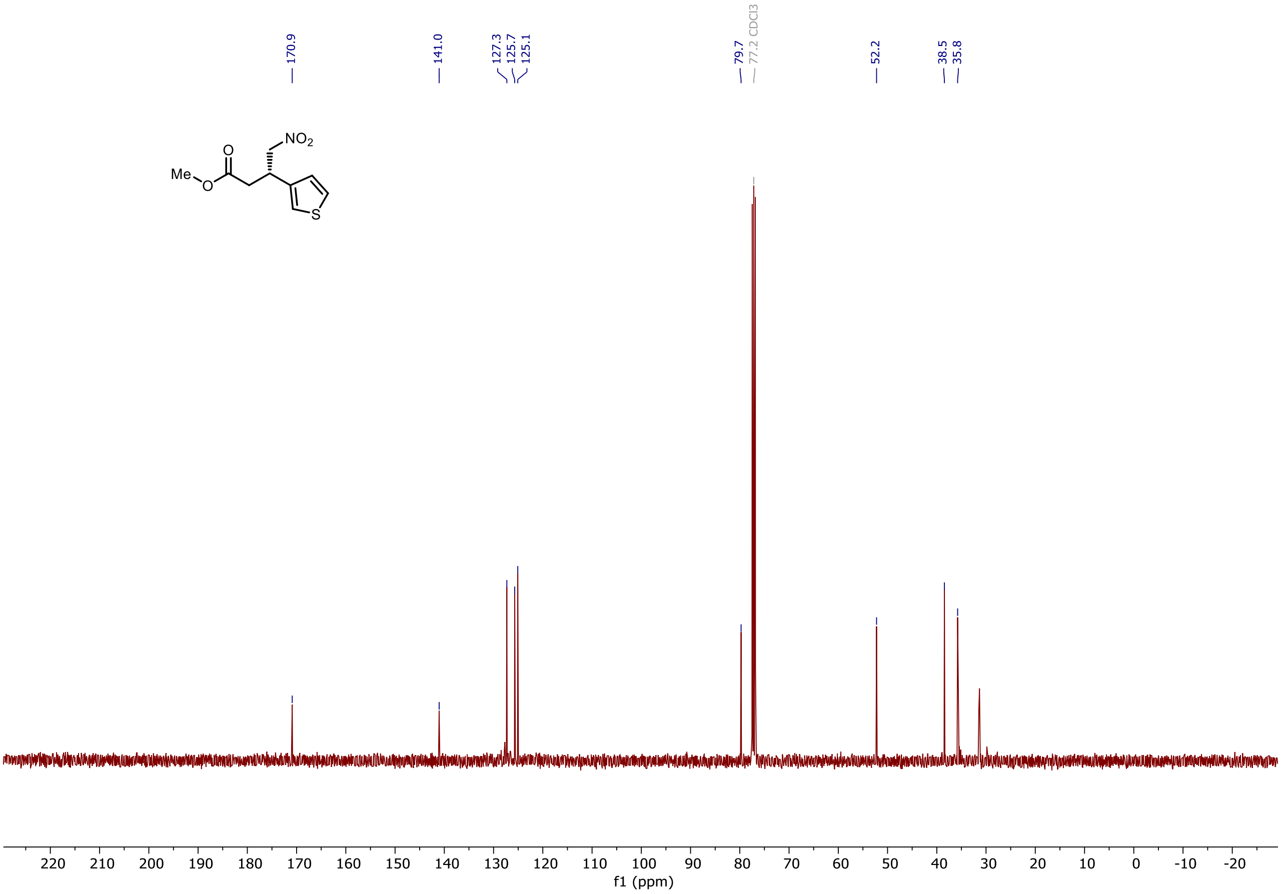
170.9
141.0
127.3
125.7
125.1
79.7
77.2 CDCl3
52.2
38.5
35.8
NO2
O
Me
O
S
f1 (ppm)

[ToC]

3ag

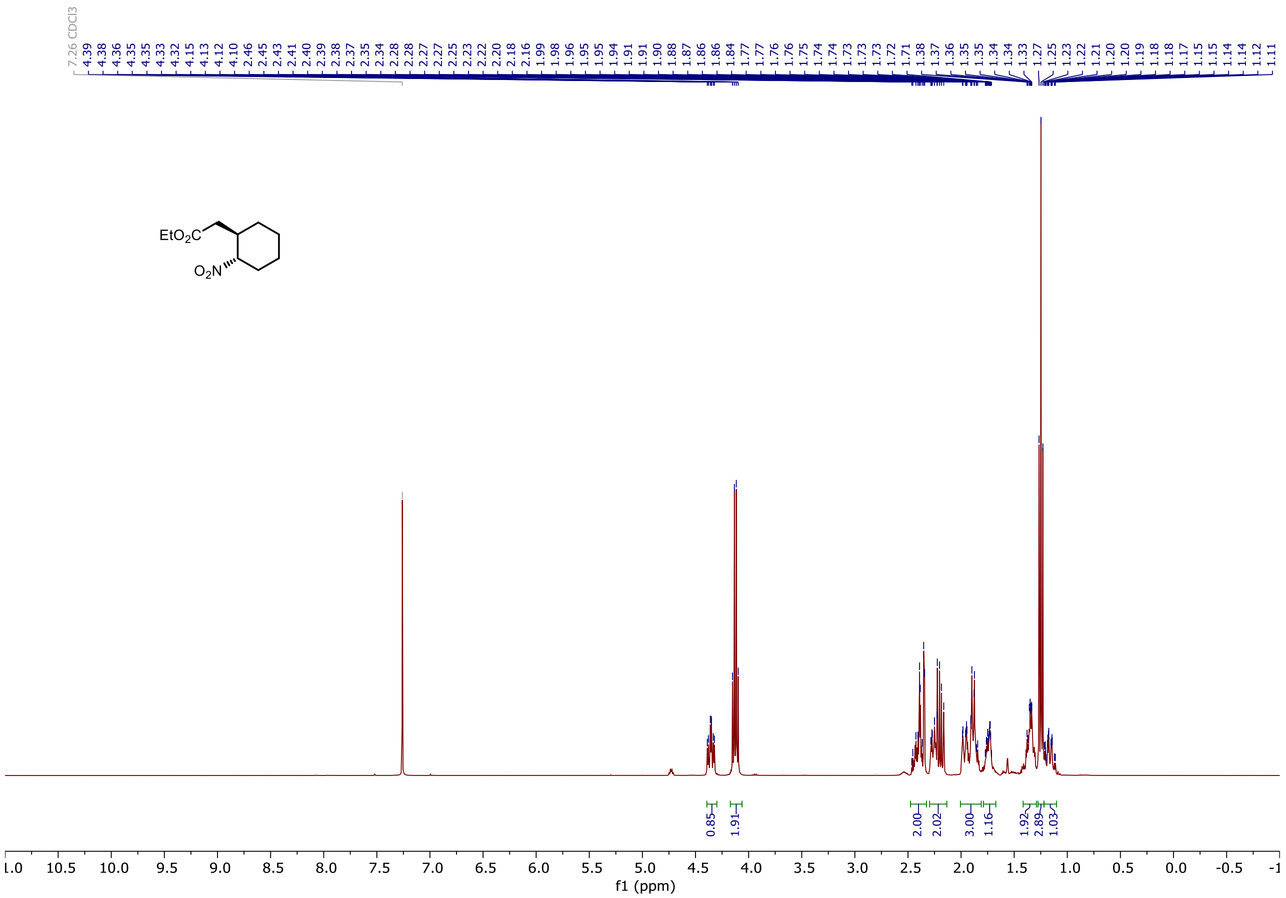

7.26 CDCl3
4.39
4.38
4.36
4.35
4.35
4.33
4.32
4.15
4.13
4.12
4.10
2.46
2.45
2.43
2.41
2.40
2.39
2.38
2.37
2.35
2.34
2.28
2.28
2.27
2.27
2.25
2.23
2.22
2.20
2.18
2.16
1.99
1.98
1.96
1.95
1.95
1.94
1.91
1.91
1.90
1.88
1.87
1.86
1.86
1.84
1.77
1.77
1.76
1.76
1.75
1.74
1.74
1.73
1.73
1.73
1.73
1.72
1.71
1.38
1.37
1.36
1.35
1.35
1.34
1.34
1.33
1.27
1.25
1.23
1.22
1.21
1.20
1.20
1.19
1.18
1.18
1.17
1.15
1.15
1.14
1.14
1.12
1.11
EtO_2C
O_2N
0.85
1.91
2.00
2.02
3.00
1.16
1.92
2.89
1.03
f1 (ppm)

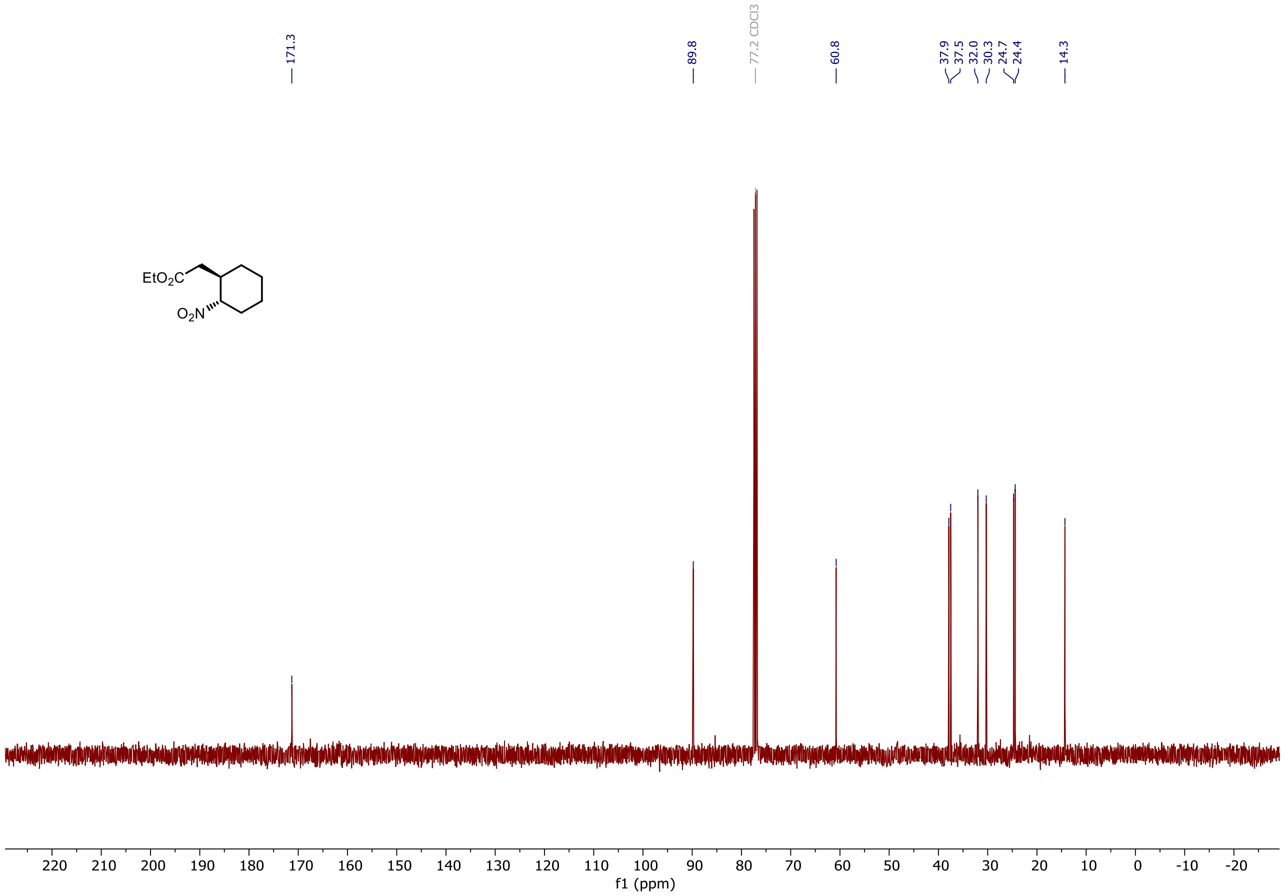

171.3
89.8
77.2 CDCl3
60.8
37.9
37.5
32.0
30.3
24.7
24.4
14.3
EtO_2C
O_2N
f1 (ppm)

[ToC]

γ-Nitroester Derivatives

4a

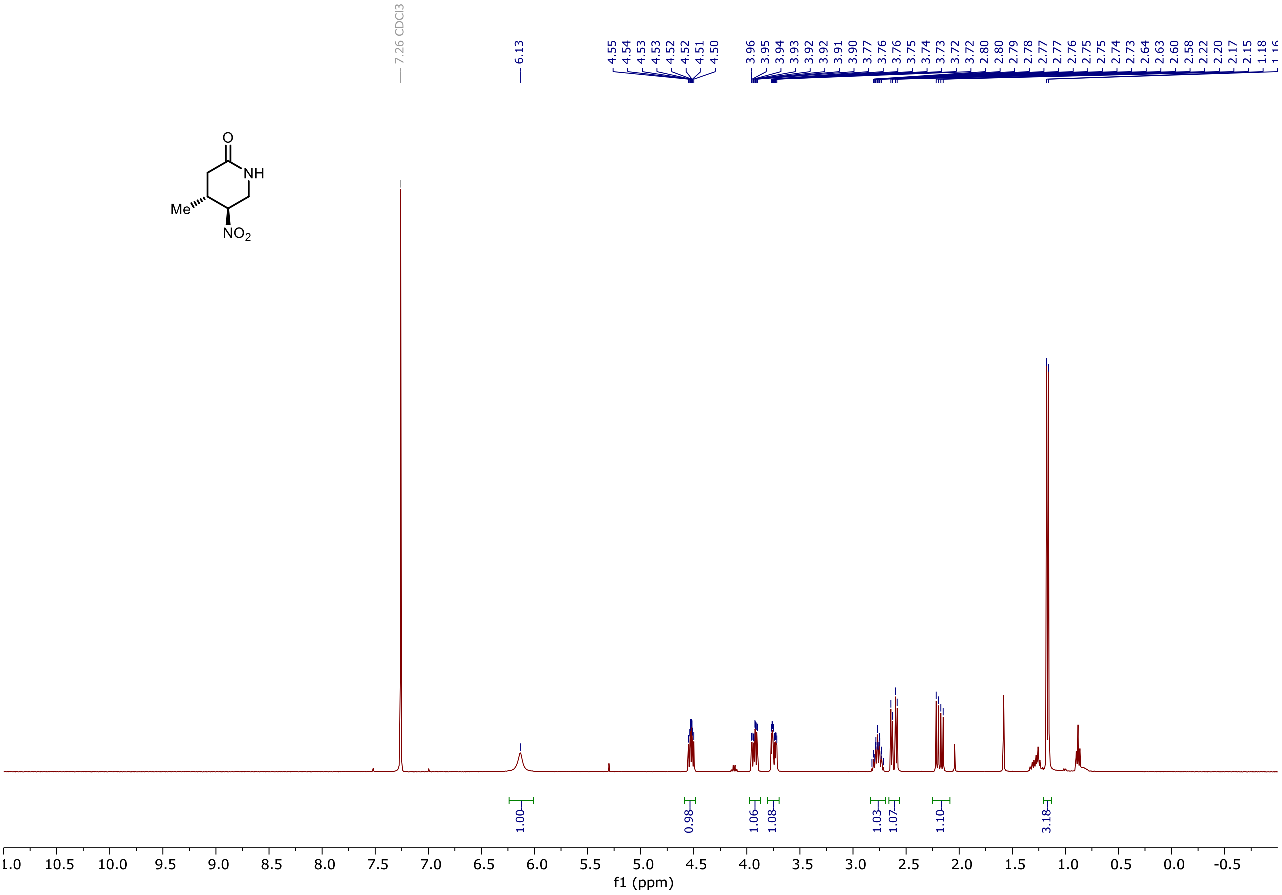

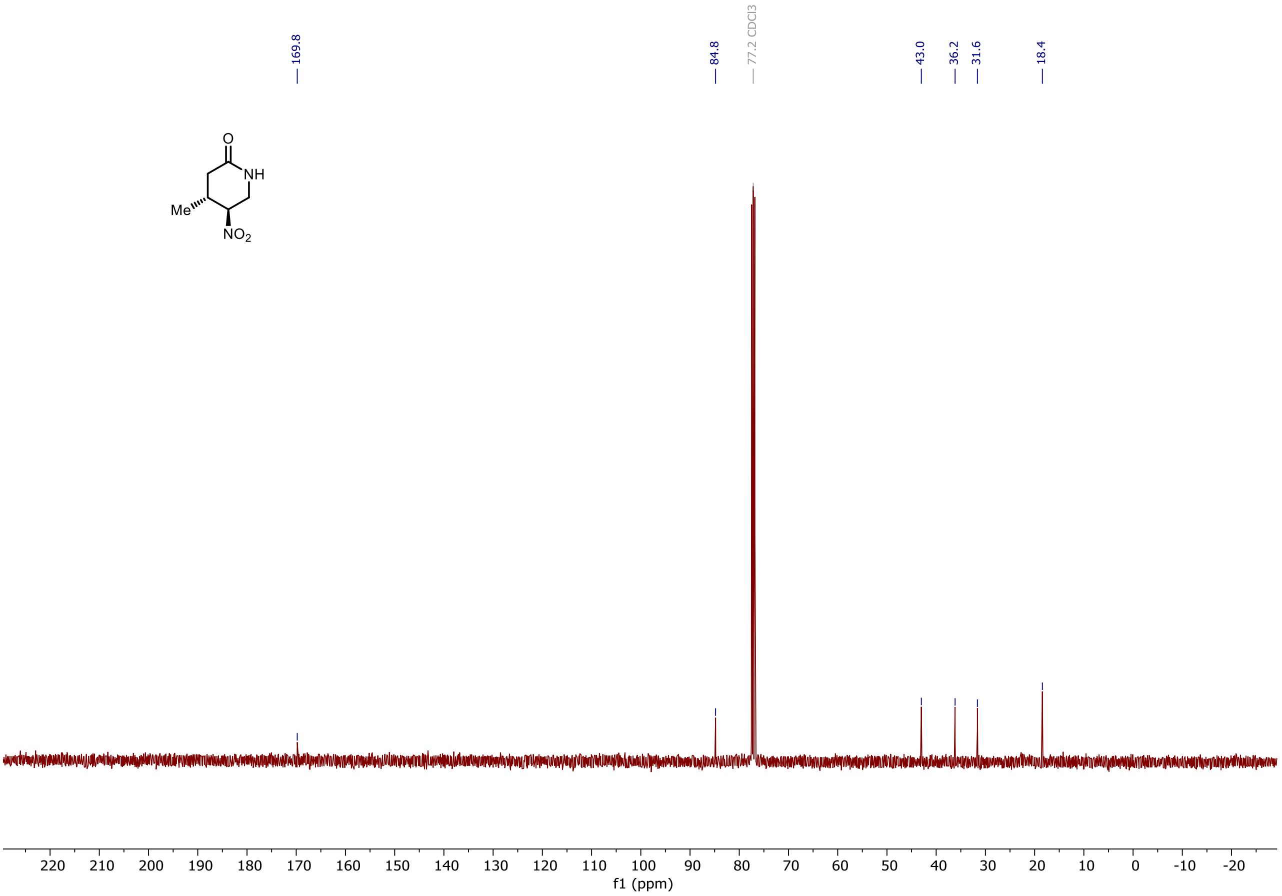

4b

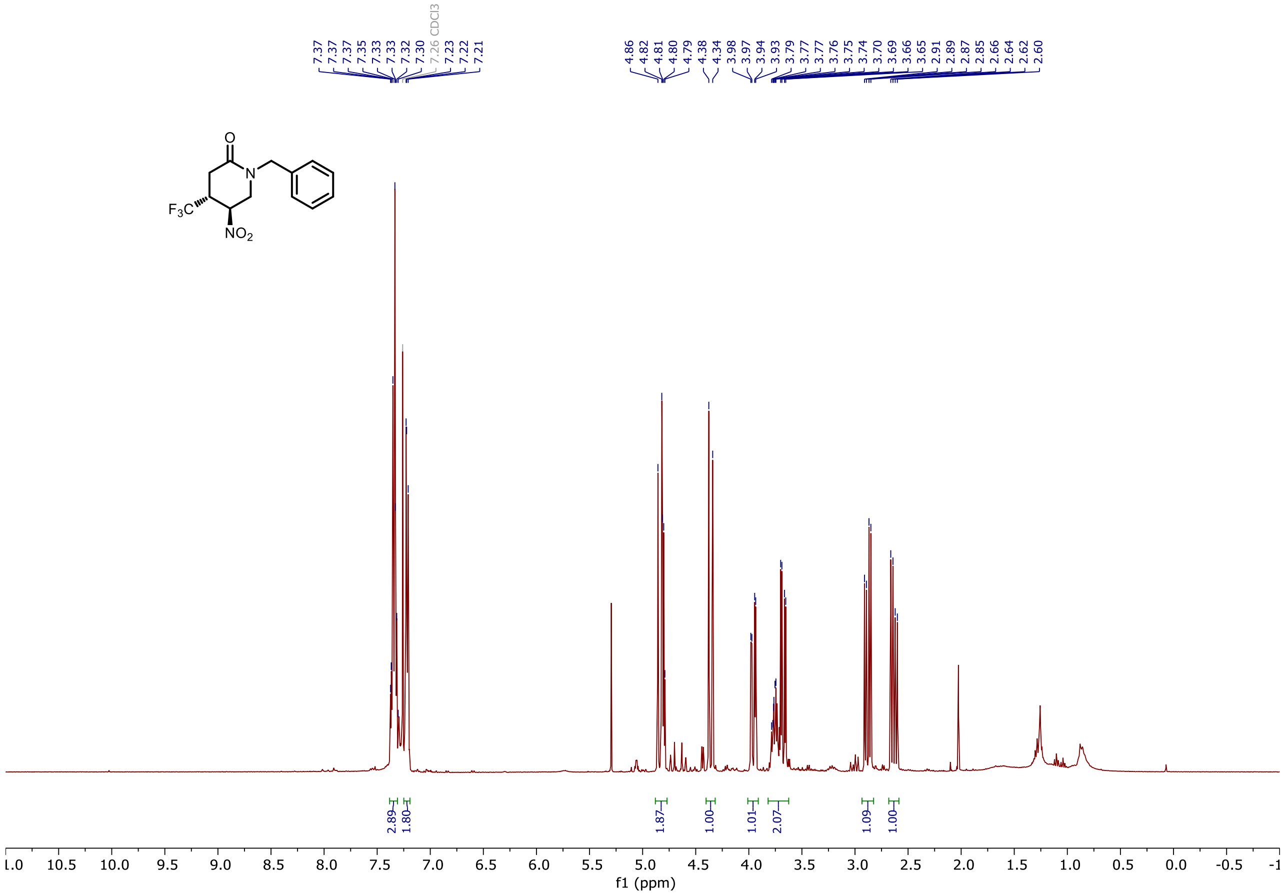
7.37
7.37
7.37
7.35
7.33
7.33
7.32
7.30
7.26 CDCl3
7.23
7.22
7.21
4.86
4.82
4.81
4.80
4.79
4.38
4.34
3.98
3.97
3.94
3.93
3.79
3.77
3.76
3.75
3.74
3.70
3.69
3.66
3.65
2.91
2.89
2.87
2.85
2.66
2.64
2.62
2.60
O
N
F_3C
NO_2
2.89
1.80
1.87
1.00
1.01
2.07
1.09
1.00
f1 (ppm)

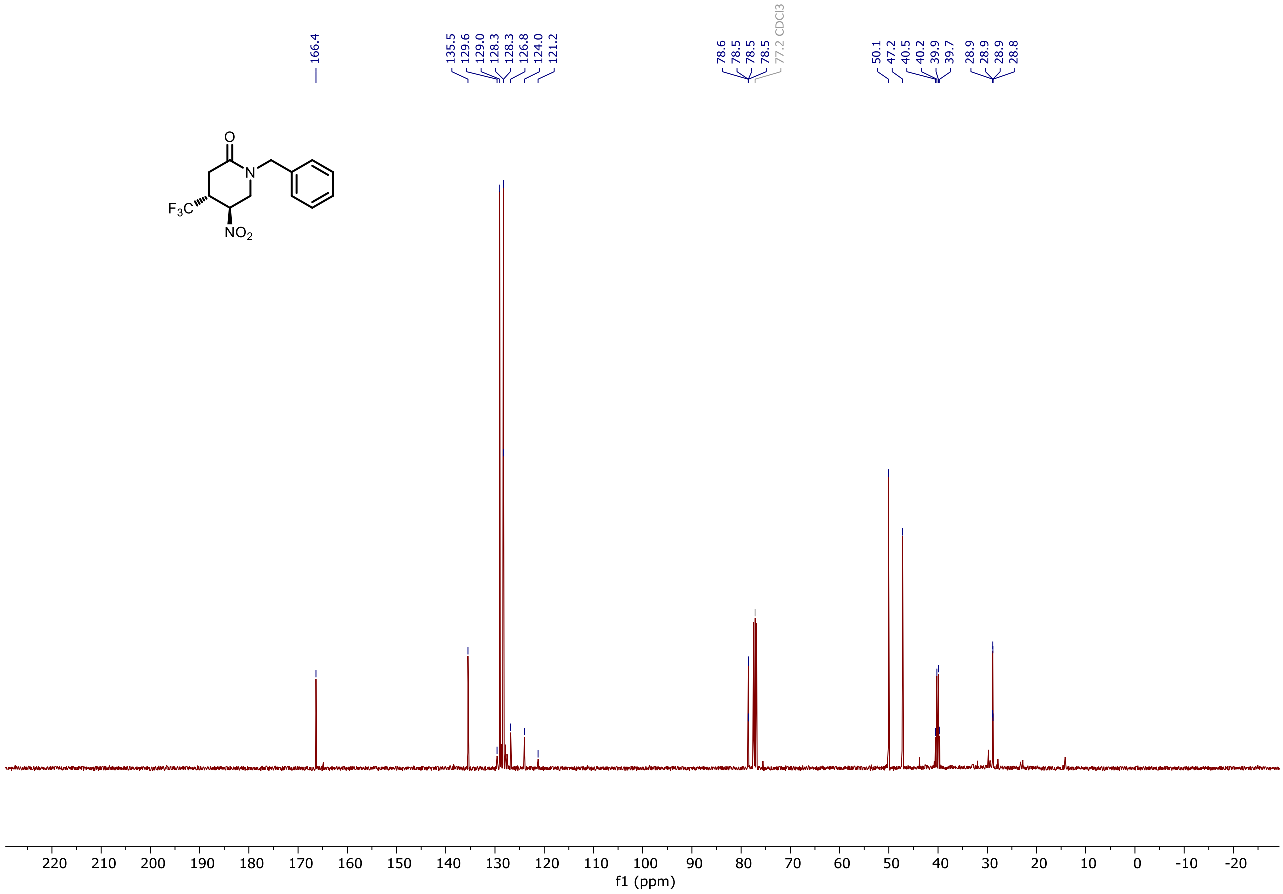
166.4
135.5
129.6
129.0
128.3
128.3
126.8
124.0
121.2
78.6
78.5
78.5
78.5
77.2 CDCl3
50.1
47.2
40.5
40.2
39.9
39.7
28.9
28.9
28.9
28.8
O
N
F_3C
NO_2
f1 (ppm)

[ToC]

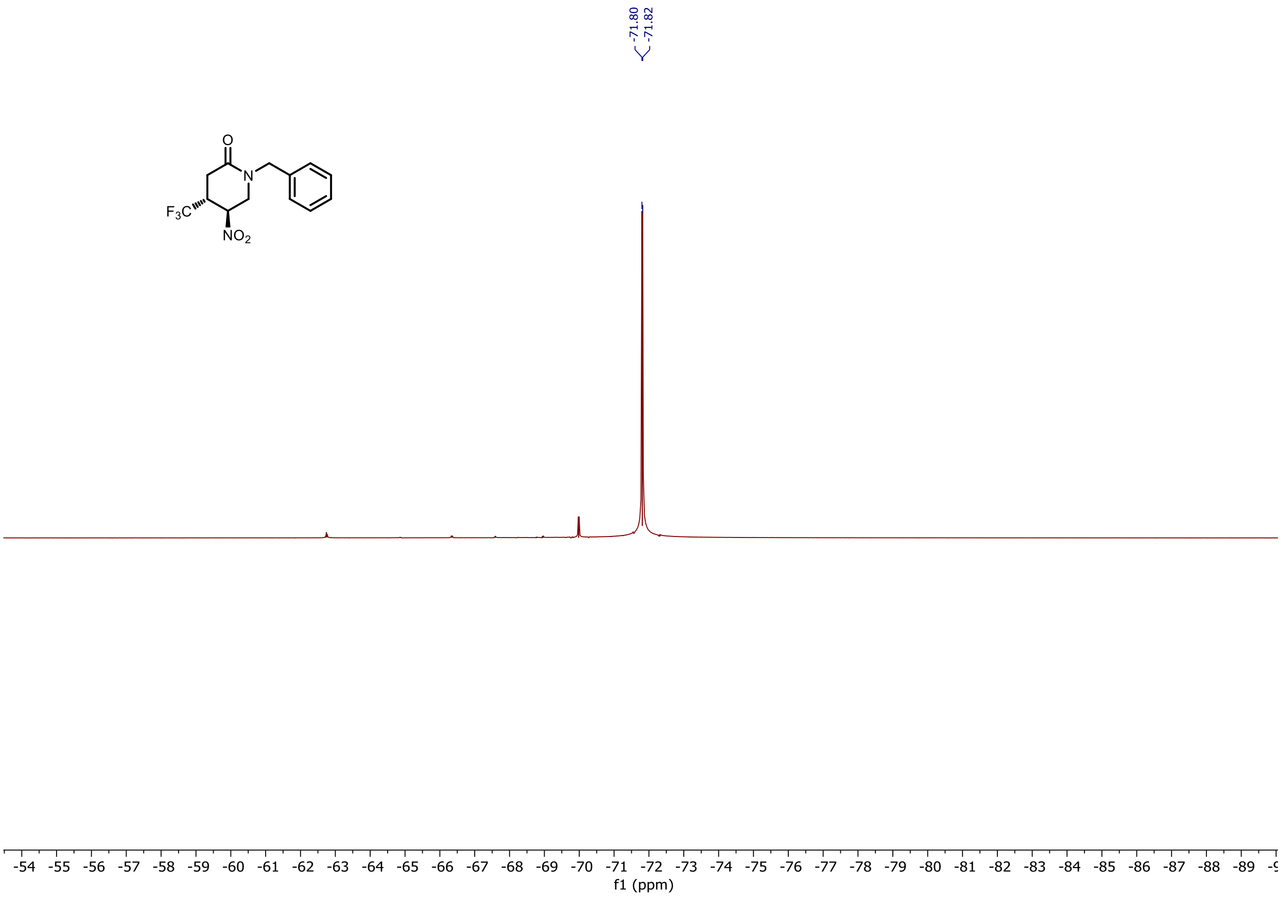

-71.80
-71.82
F3C
NO2
N
O
-54 -55 -56 -57 -58 -59 -60 -61 -62 -63 -64 -65 -66 -67 -68 -69 -70 -71 -72 -73 -74 -75 -76 -77 -78 -79 -80 -81 -82 -83 -84 -85 -86 -87 -88 -89
f1 (ppm)

[ToC]

4c

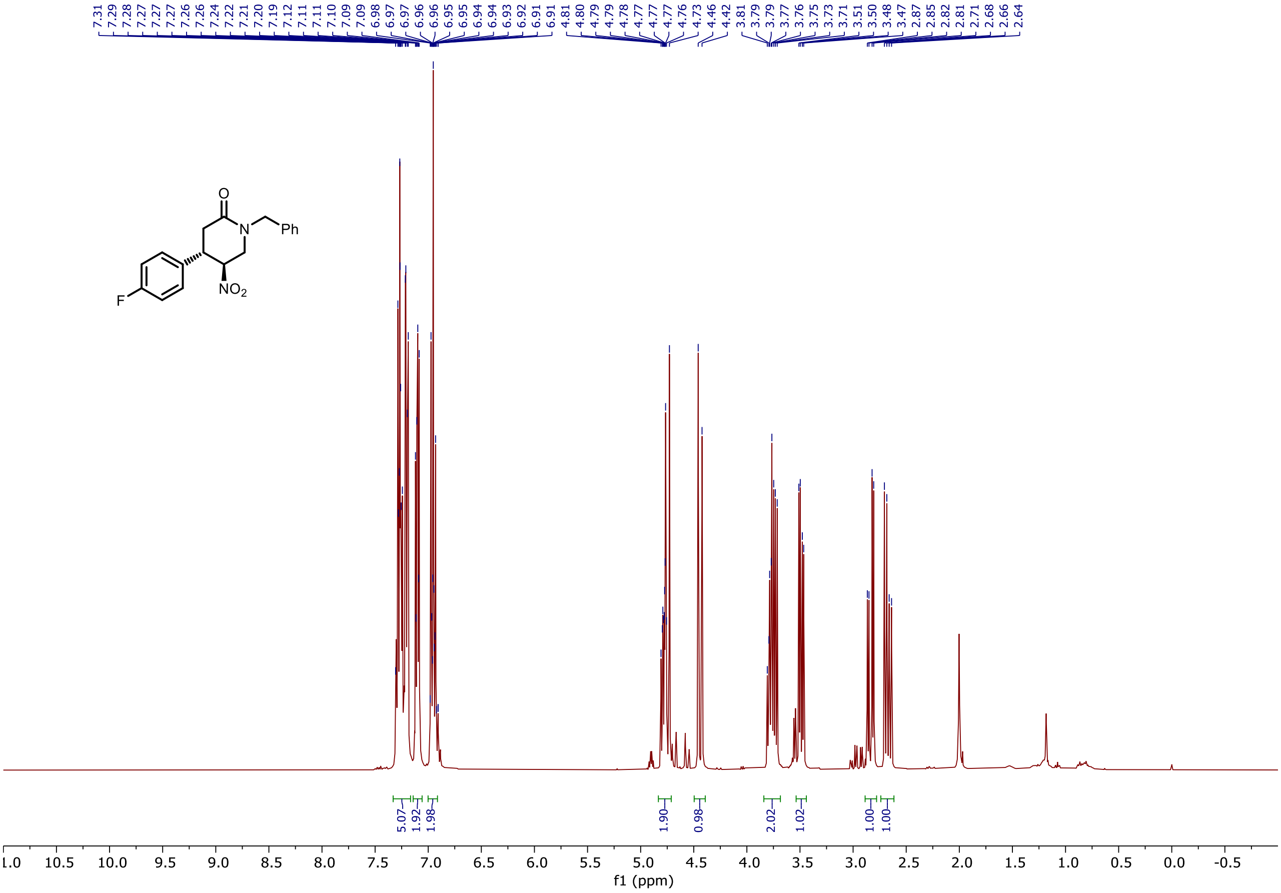

7.31 7.29 7.28 7.27 7.27 7.27 7.26 7.24 7.22 7.21 7.20 7.19 7.12 7.11 7.11 7.10 7.09 6.98 6.97 6.97 6.96 6.96 6.95 6.95 6.94 6.94 6.93 6.92 6.91 6.91 4.81 4.80 4.79 4.79 4.78 4.77 4.77 4.77 4.76 4.73 4.46 4.42 3.81 3.79 3.79 3.77 3.76 3.75 3.73 3.71 3.51 3.50 3.48 3.47 2.87 2.85 2.82 2.81 2.71 2.68 2.66 2.64
O
N
Ph
F
NO_2
5.07 1.92 1.98 1.90 0.98 2.02 1.02 1.00 1.00
f1 (ppm)

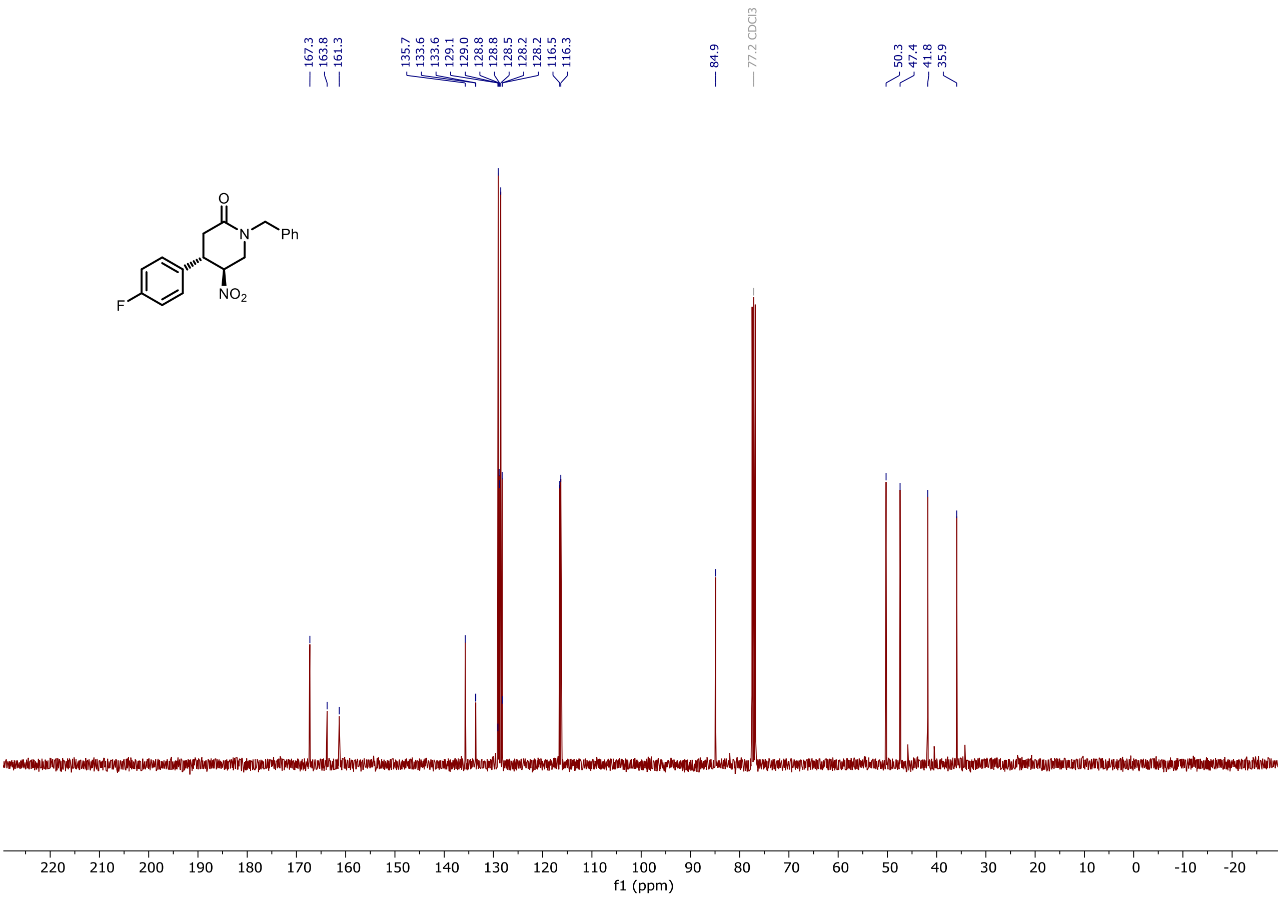

167.3 163.8 161.3 135.7 133.6 133.6 129.1 129.0 128.8 128.8 128.5 128.2 116.5 116.3 84.9 77.2 CDCl3 50.3 47.4 41.8 35.9
O
N
Ph
F
NO_2
f1 (ppm)

[ToC]

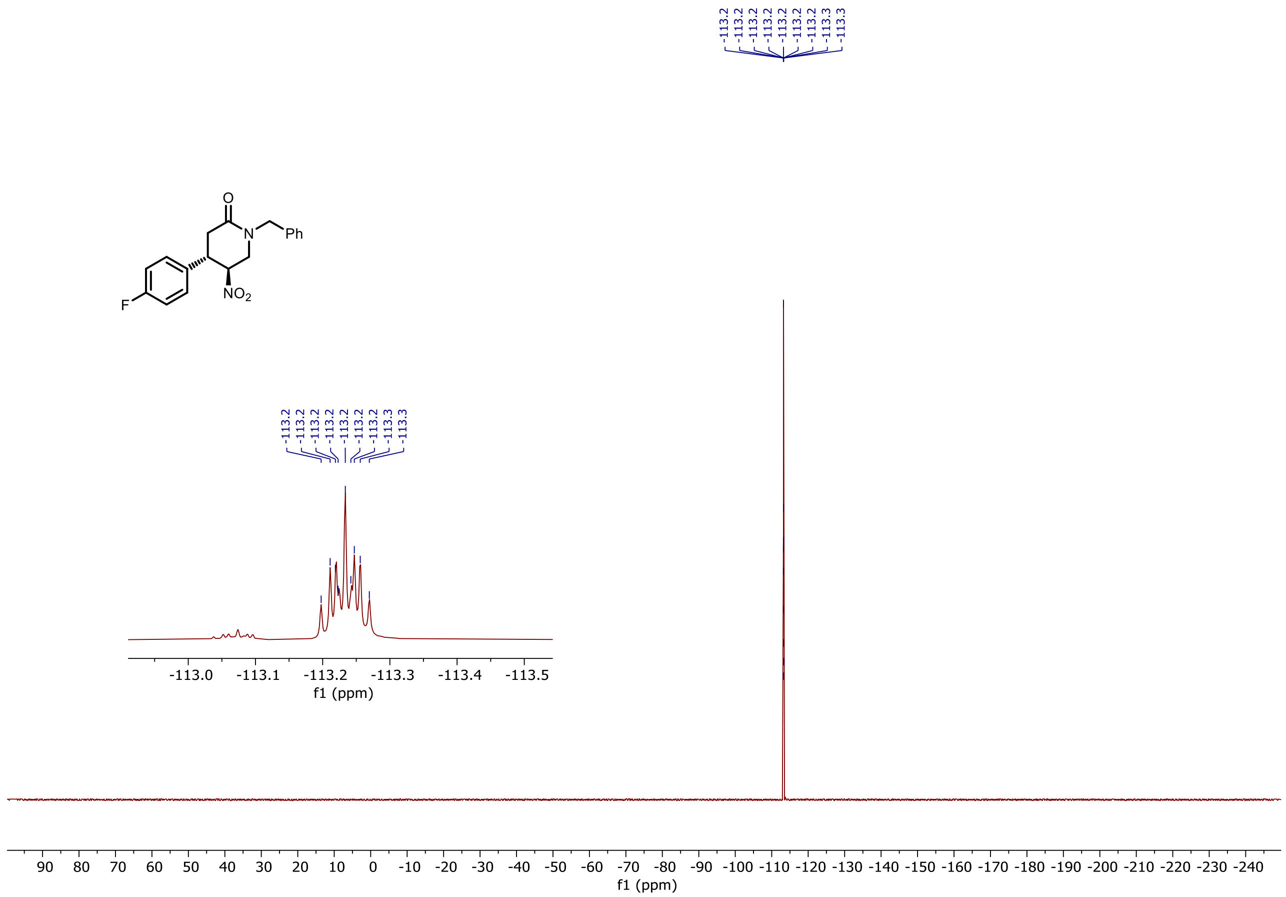

O
N
Ph
F
NO2
-113.2
-113.2
-113.2
-113.2
-113.2
-113.2
-113.2
-113.3
-113.3
-113.0
-113.1
-113.2
-113.3
-113.4
-113.5
f1 (ppm)
90 80 70 60 50 40 30 20 10 0 -10 -20 -30 -40 -50 -60 -70 -80 -90 -100 -110 -120 -130 -140 -150 -160 -170 -180 -190 -200 -210 -220 -230 -240
f1 (ppm)

[ToC]

5a

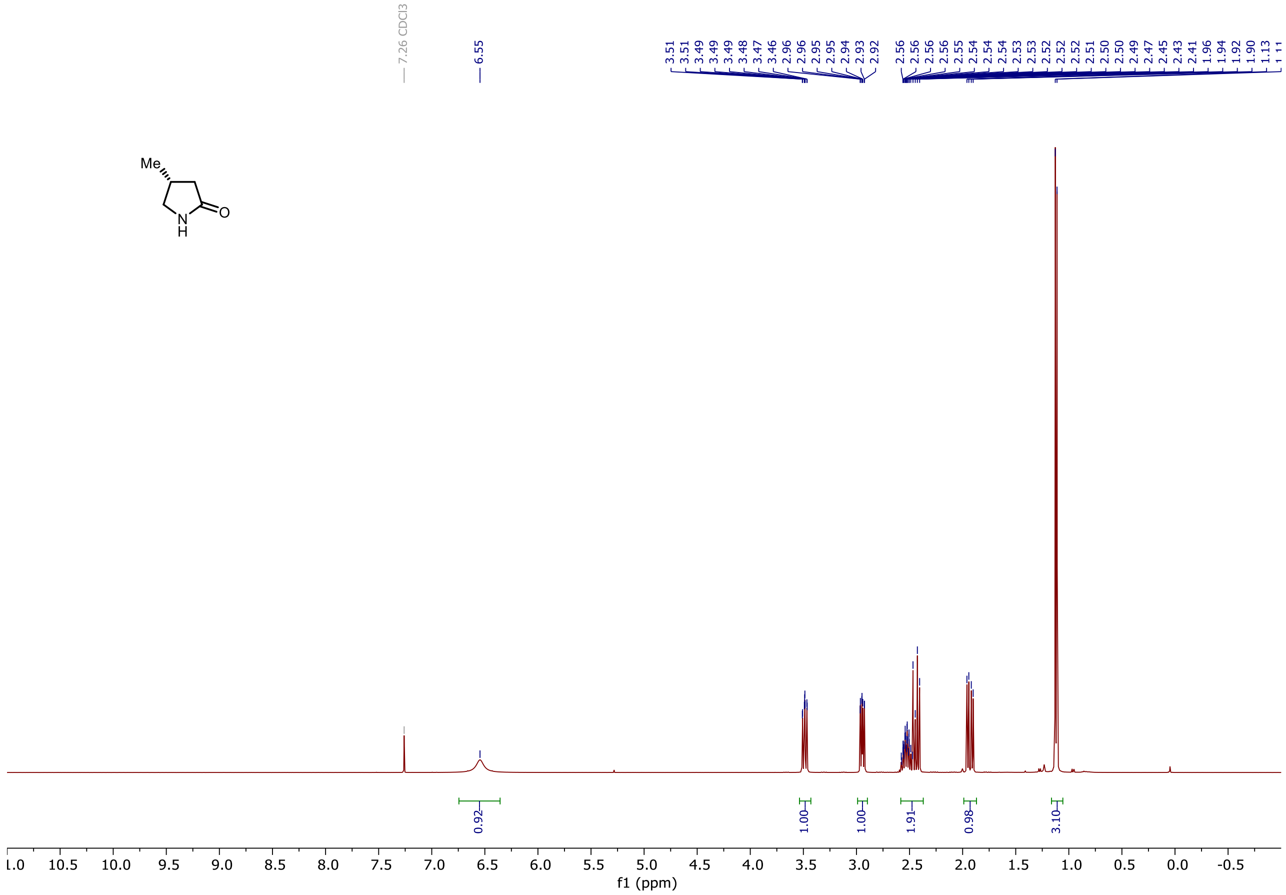

7.26 CDCl3
6.55
0.92
1.00
1.00
1.91
0.98
3.10
Me
N
H
O
f1 (ppm)

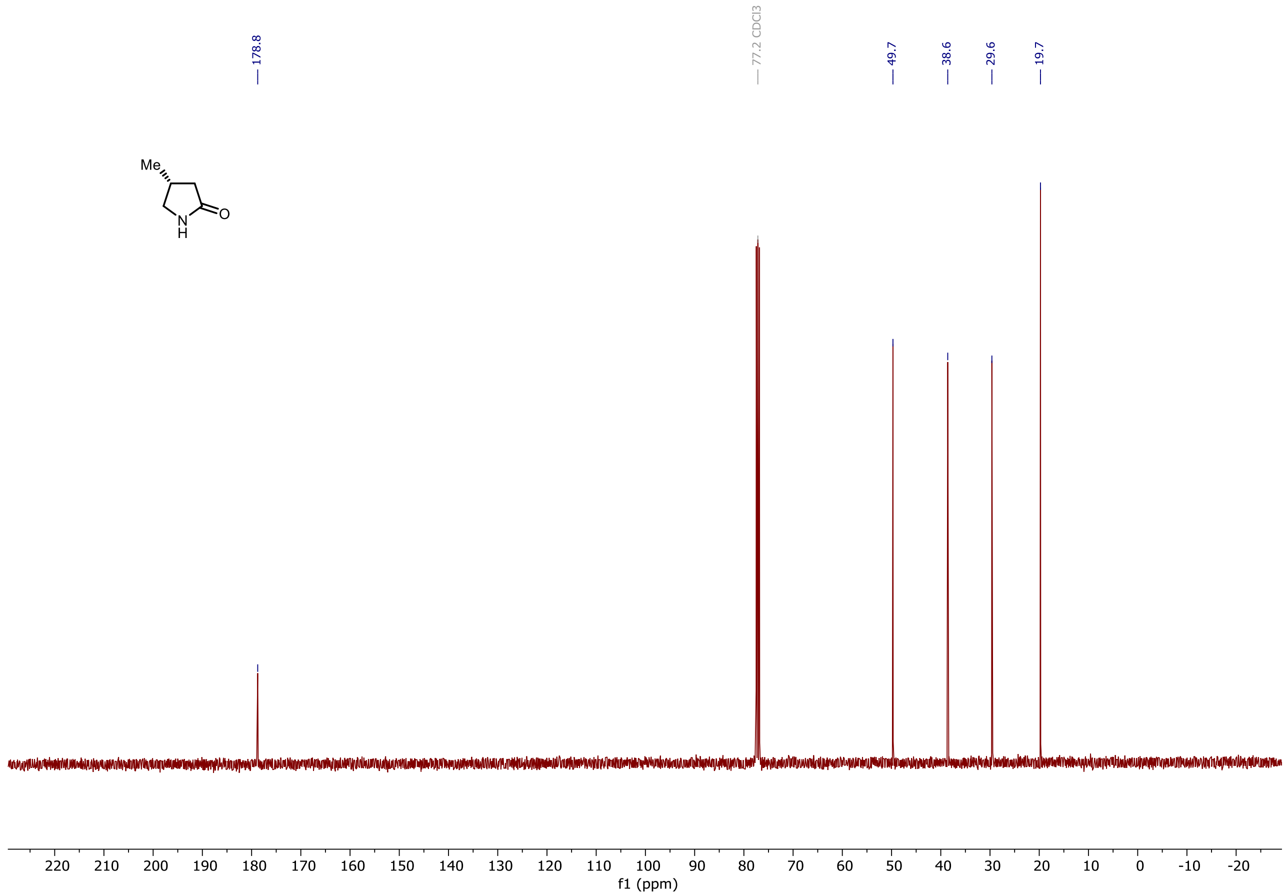

178.8
77.2 CDCl3
49.7
38.6
29.6
19.7
Me
N
H
O
f1 (ppm)

[ToC]

5b

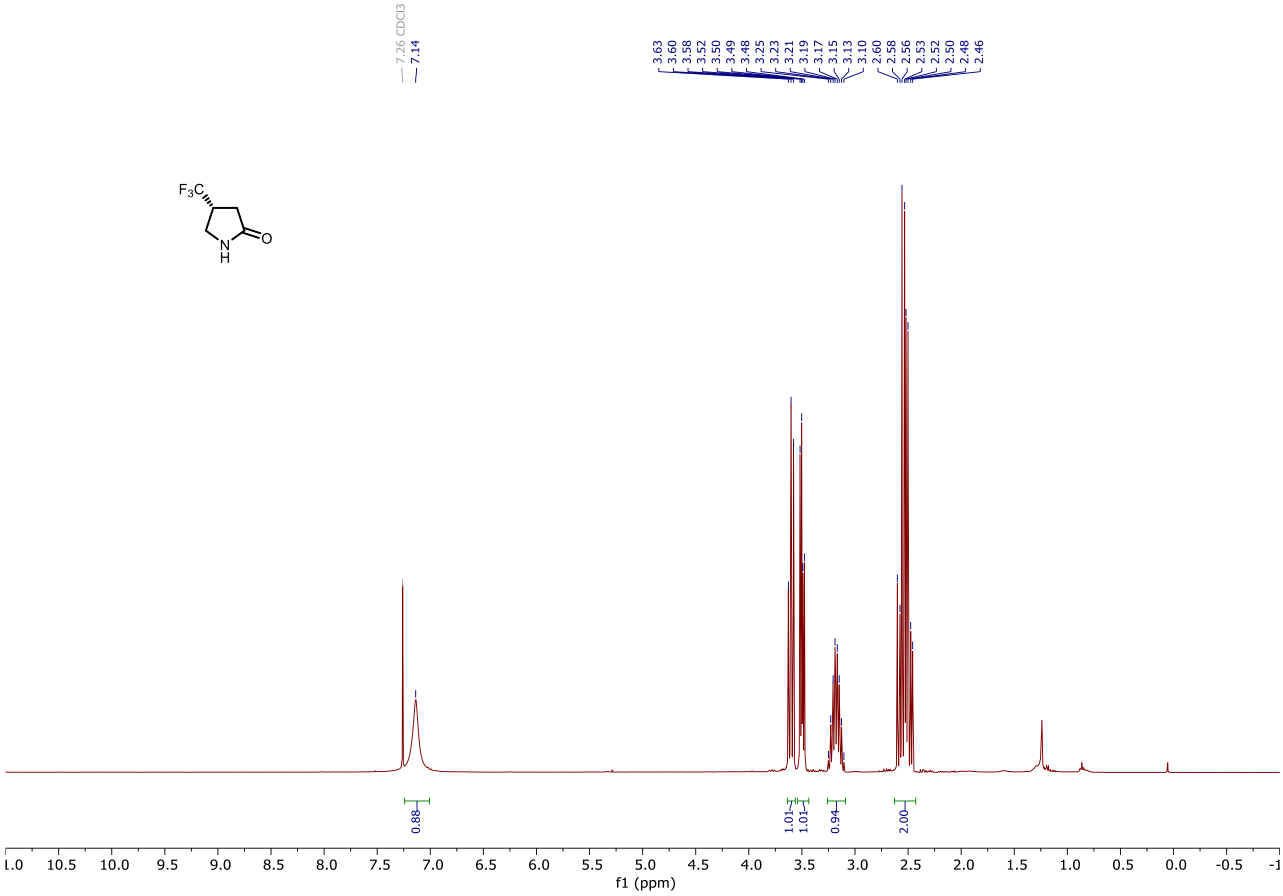

7.26 CDCl3
7.14
3.63
3.60
3.58
3.52
3.50
3.49
3.48
3.25
3.23
3.21
3.19
3.17
3.15
3.13
3.10
2.60
2.58
2.56
2.53
2.52
2.50
2.48
2.46
F_3C
N
H
O
0.88
1.01
1.01
0.94
2.00
f1 (ppm)

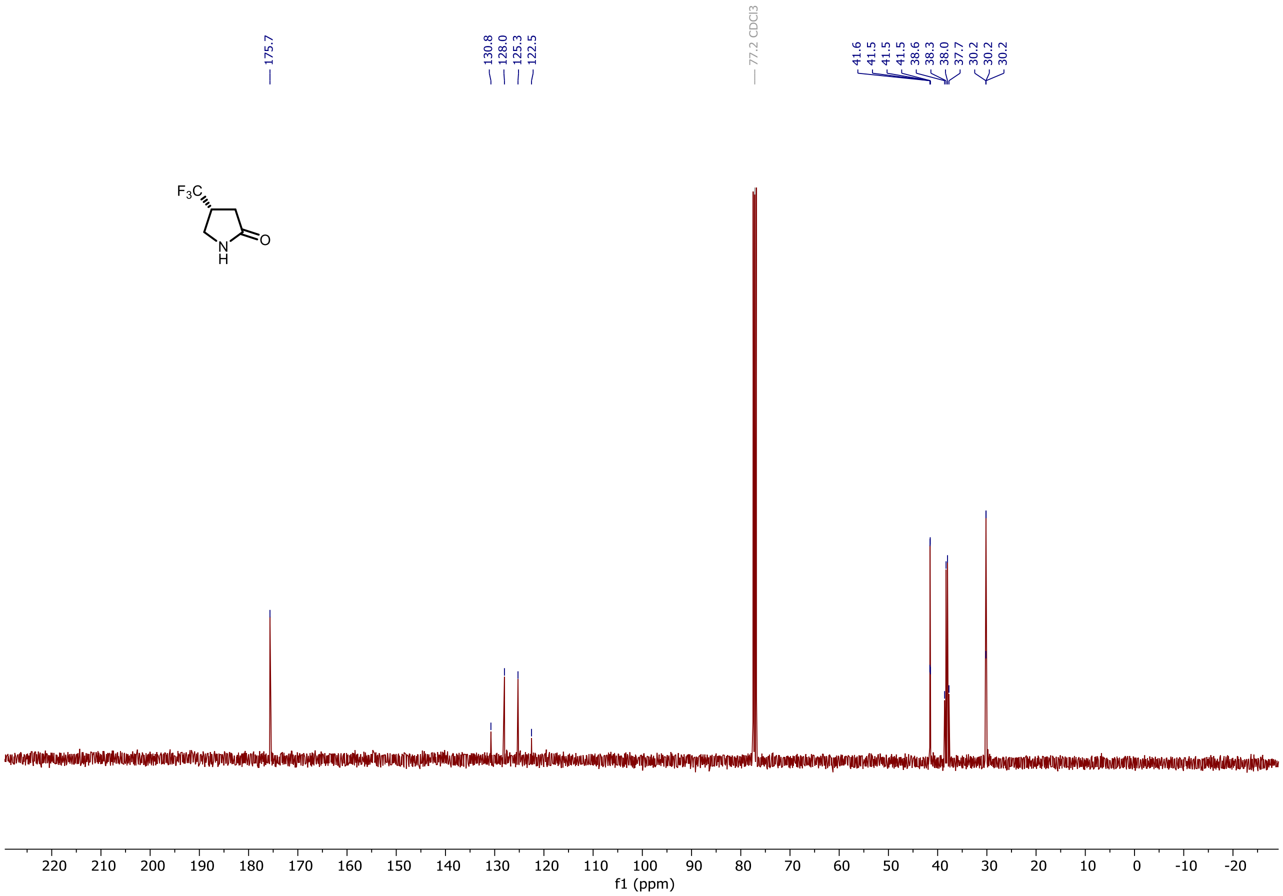

175.7
130.8
128.0
125.3
122.5
77.2 CDCl3
41.6
41.5
41.5
41.5
38.6
38.3
38.0
37.7
30.2
30.2
30.2
F_3C
N
H
O
f1 (ppm)

[ToC]

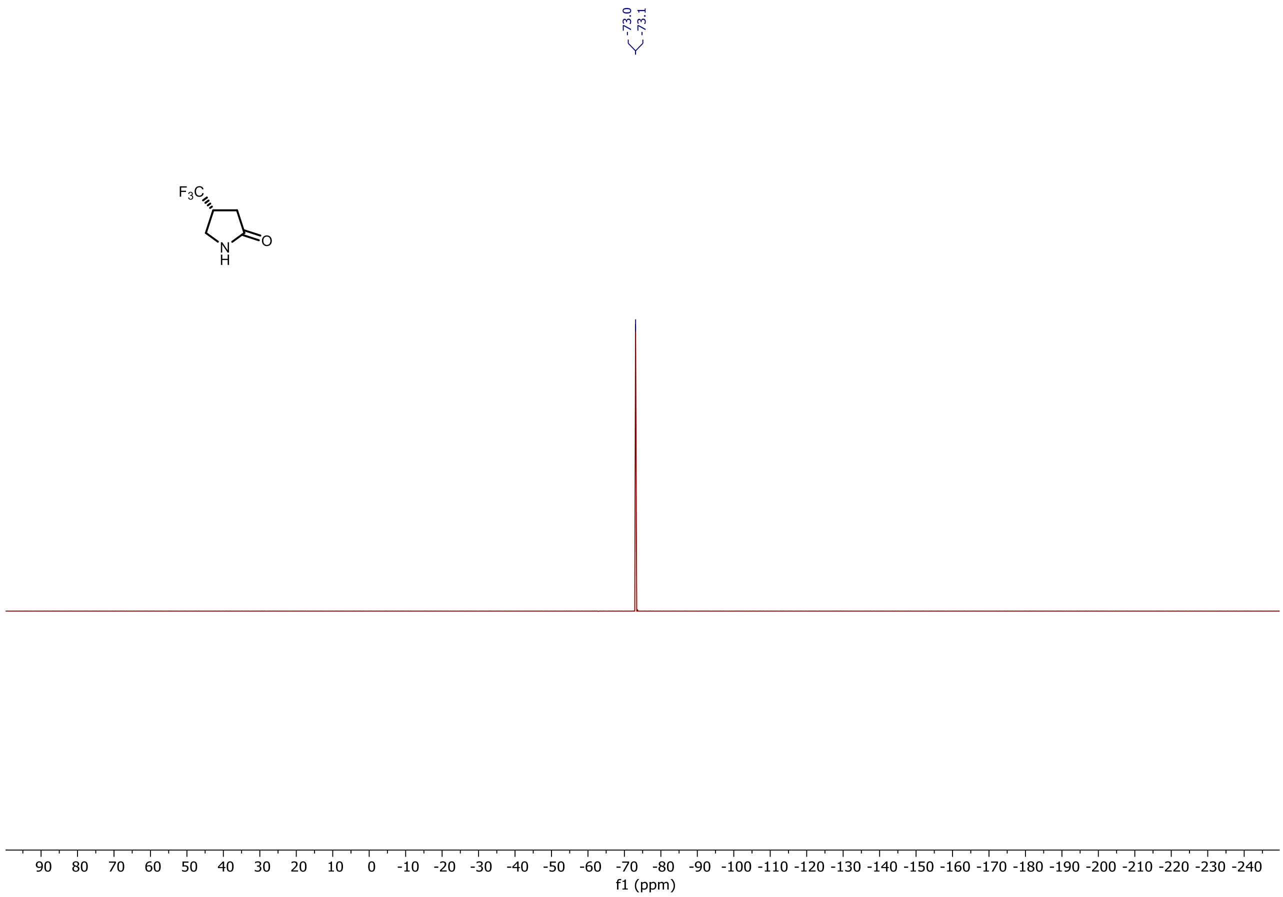

-73.0
-73.1
90 80 70 60 50 40 30 20 10 0 -10 -20 -30 -40 -50 -60 -70 -80 -90 -100 -110 -120 -130 -140 -150 -160 -170 -180 -190 -200 -210 -220 -230 -240
f1 (ppm)

[[ToC]]

5c

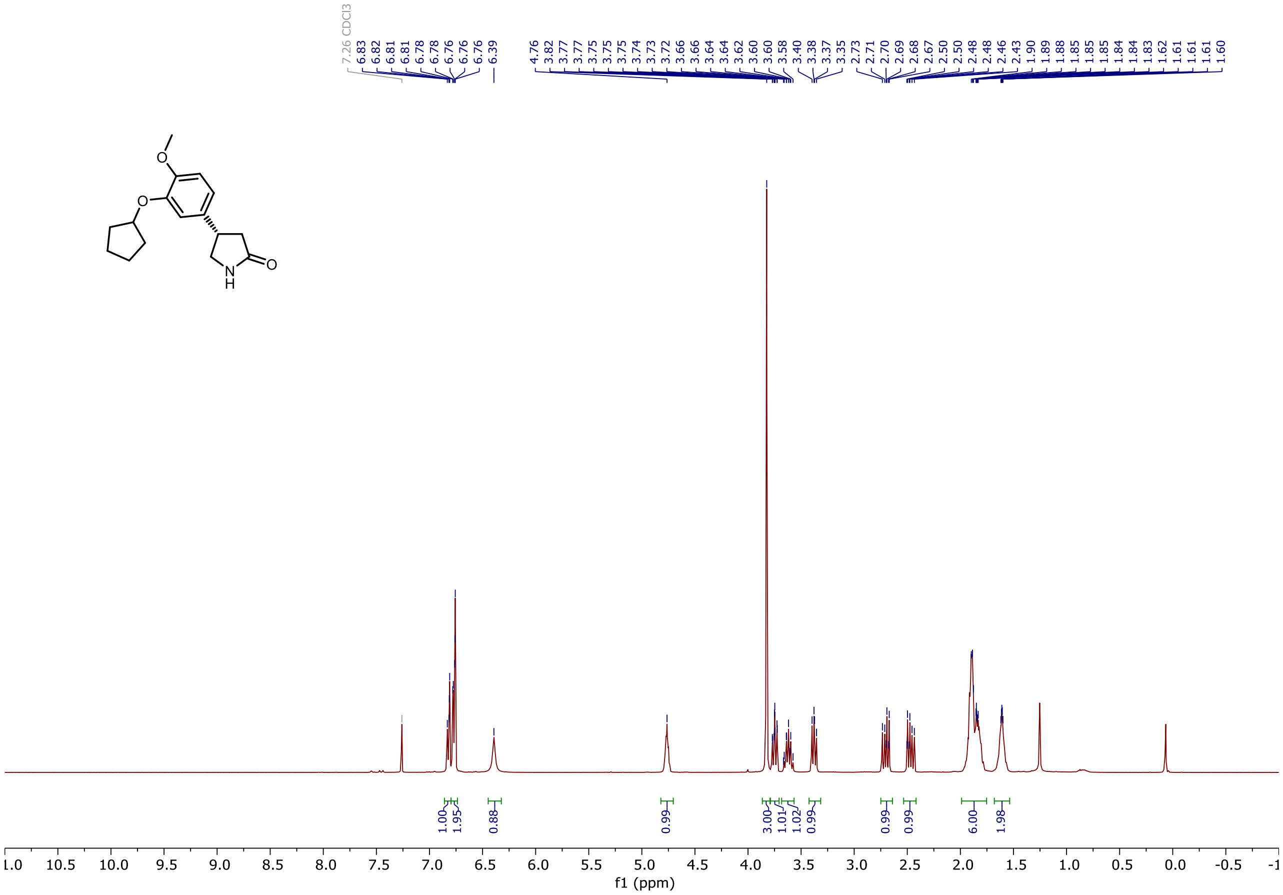

7.26 CDCl3
6.83
6.82
6.81
6.81
6.78
6.78
6.76
6.76
6.76
6.39
4.76
3.82
3.77
3.77
3.75
3.75
3.75
3.74
3.73
3.72
3.66
3.66
3.64
3.64
3.62
3.60
3.60
3.58
3.40
3.38
3.37
3.35
2.73
2.71
2.70
2.69
2.68
2.67
2.50
2.50
2.48
2.48
2.46
2.43
1.90
1.89
1.88
1.85
1.85
1.85
1.84
1.84
1.83
1.62
1.61
1.61
1.61
1.60
O
O
N
H
O
1.00
1.95
0.88
0.99
3.00
1.01
1.02
0.99
0.99
0.99
6.00
1.98
f1 (ppm)

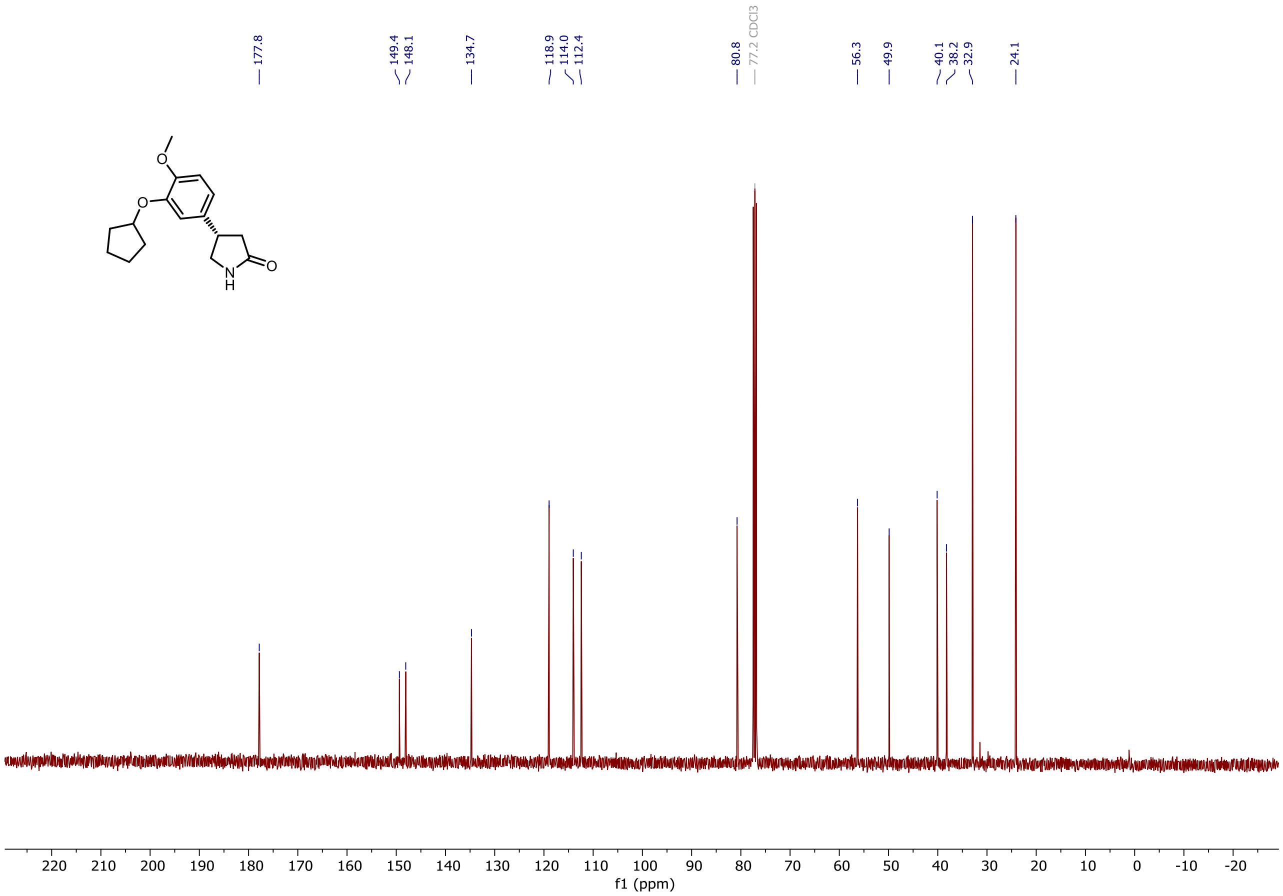

177.8
149.4
148.1
134.7
118.9
114.0
112.4
80.8
77.2 CDCl3
56.3
49.9
40.1
38.2
32.9
24.1
O
O
N
H
O
f1 (ppm)

[ToC]

5d

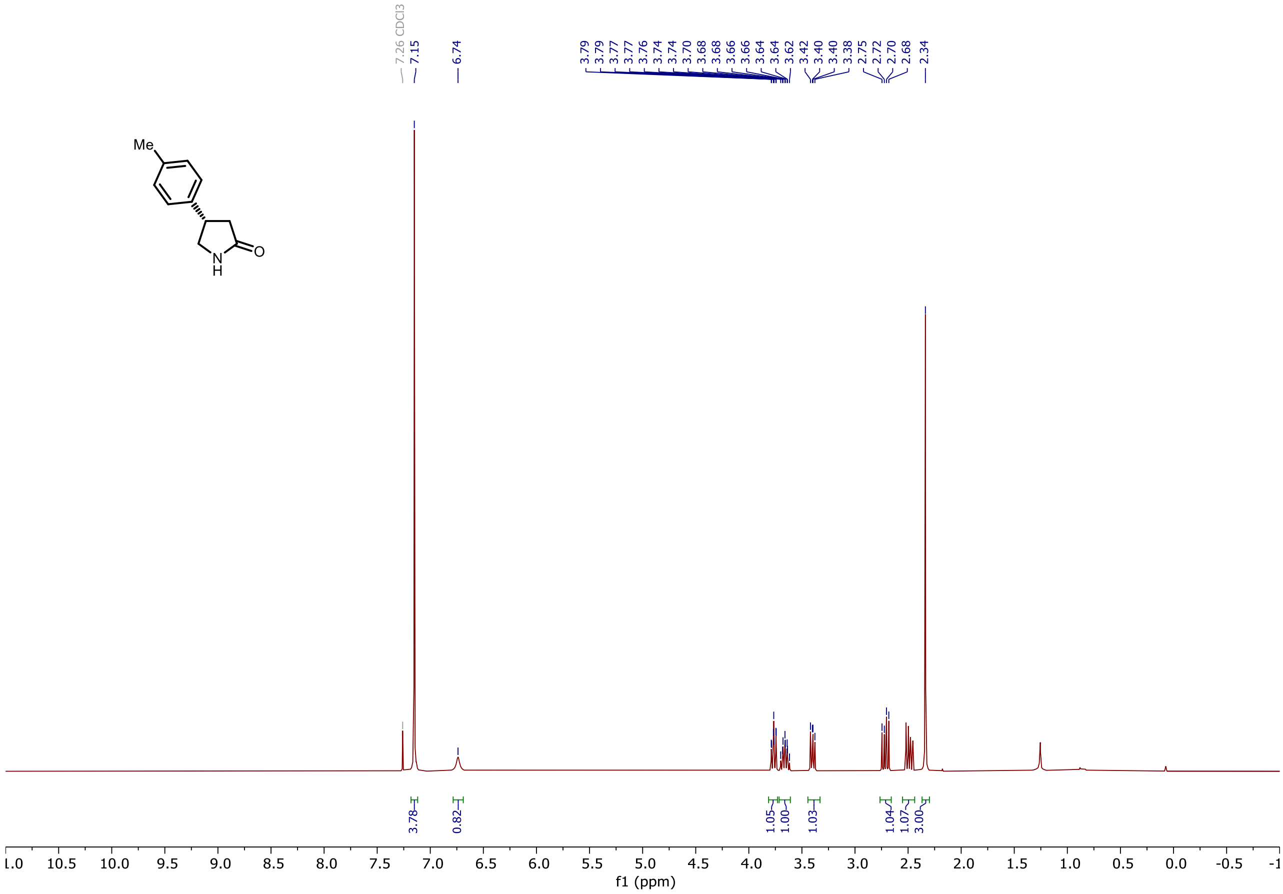

7.26 CDCl3
7.15
6.74
3.79 3.79 3.77 3.76 3.74 3.74 3.70 3.68 3.68 3.66 3.66 3.64 3.64 3.62
3.42 3.40 3.40 3.38
2.75 2.72 2.70 2.68
2.34
Me
N
H
O
3.78
0.82
1.05
1.00
1.03
1.04
1.07
3.00
f1 (ppm)

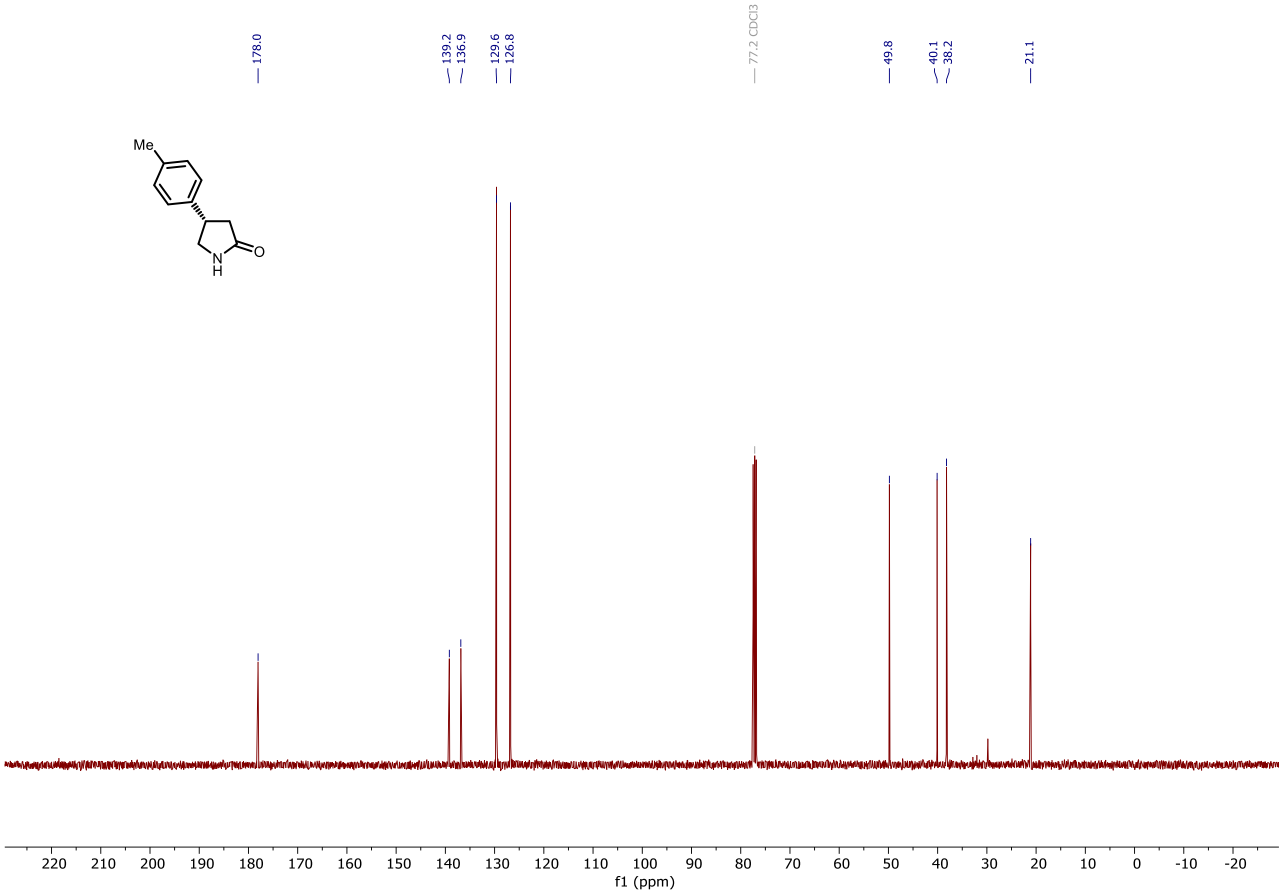

178.0
139.2
136.9
129.6
126.8
77.2 CDCl3
49.8
40.1
38.2
21.1
Me
N
H
O
f1 (ppm)

[ToC]

HPLC and SFC Traces

γ-Nitroesters

3a

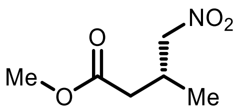

Racemic (CHIRALPAK® AS-H, hexane/IPA = 95/05, 1 mL/min)

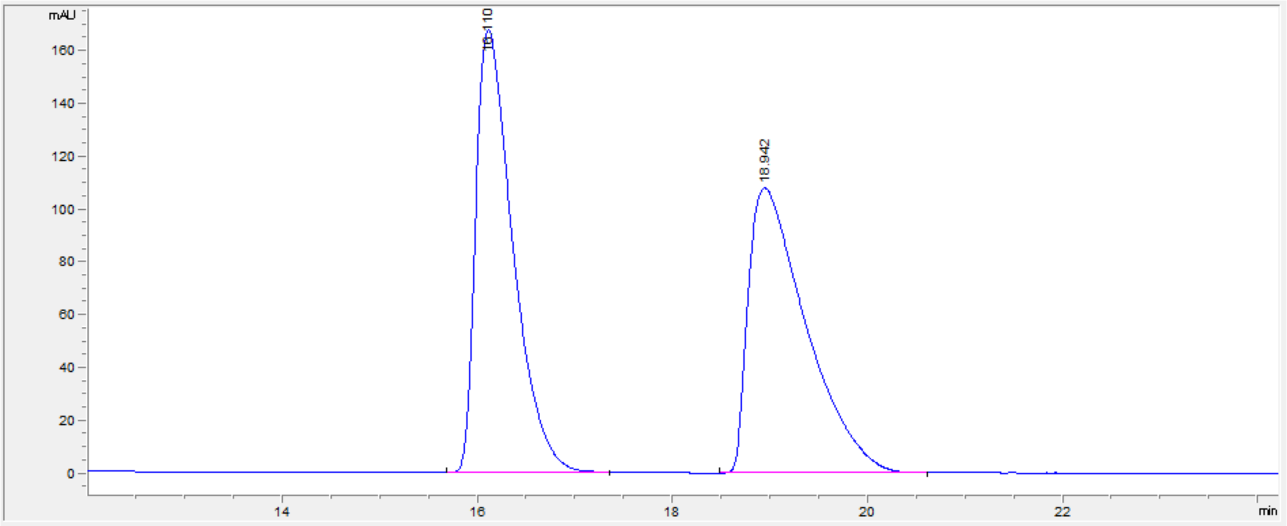

#	Time	Area	Height	Width	Area%	Symmetry
1	16.11	4422.2	167.5	0.402	49.891	0.478
2	18.942	4441.6	107.8	0.6205	50.109	0.366

Enantioenriched (CHIRALPAK® AS-H, hexane/IPA = 95/05, 1 mL/min)

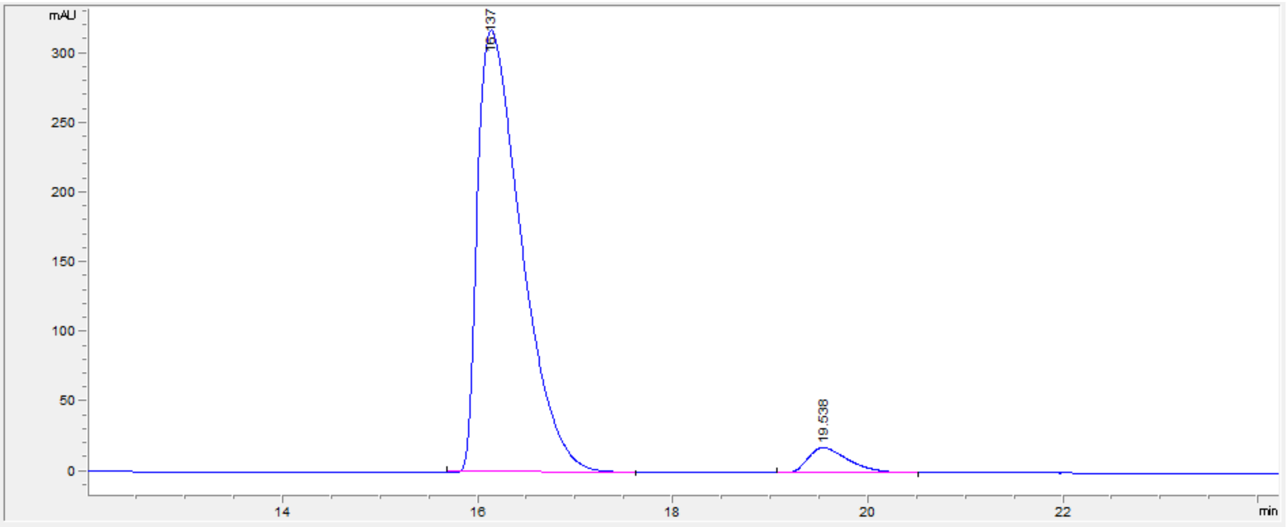

#	Time	Area	Height	Width	Area%	Symmetry
1	16.137	9874.4	317.3	0.479	95.002	0.423
2	19.538	519.4	17.9	0.4356	4.998	0.563

[ToC]

3b

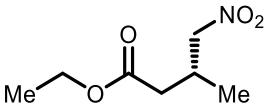

Racemic (CHIRALPAK® AS-H, hexane/IPA = 95/05, 1 mL/min)

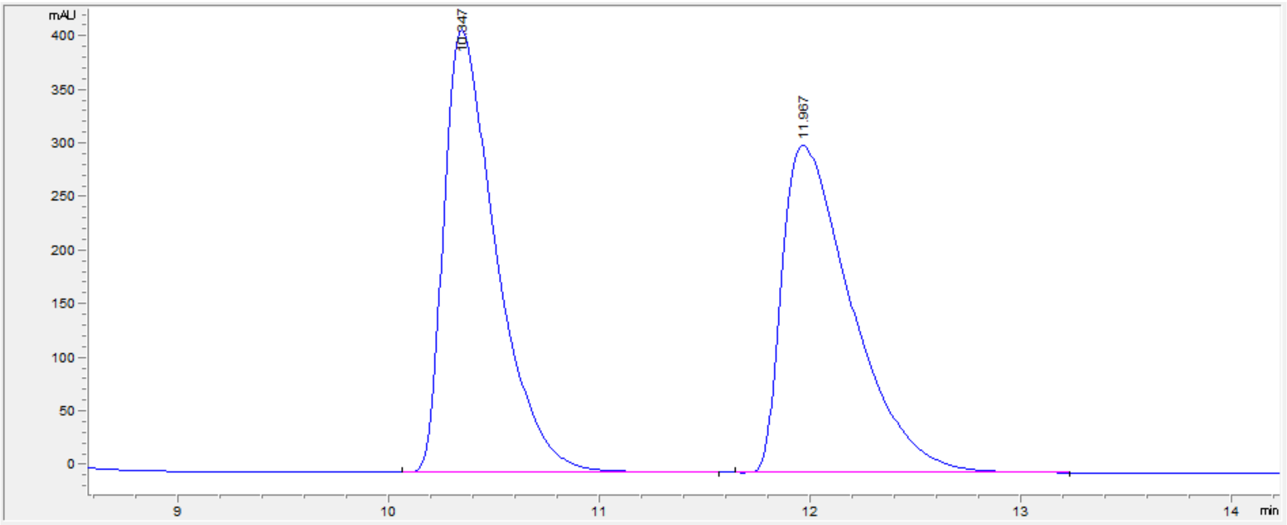

#	Time	Area	Height	Width	Area%	Symmetry
1	10.347	7121.3	412.6	0.2614	50.267	0.492
2	11.967	7045.7	305.4	0.3491	49.733	0.415

Enantioenriched (CHIRALPAK® AS-H, hexane/IPA = 95/05, 1 mL/min)

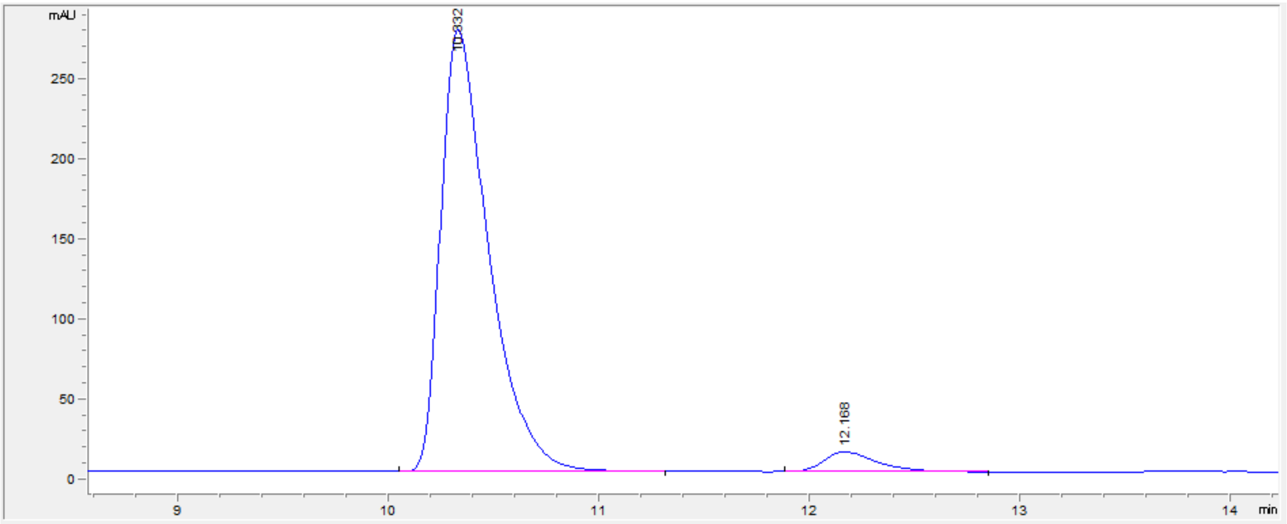

#	Time	Area	Height	Width	Area%	Symmetry
1	10.332	4365.7	275.6	0.2388	95.257	0.53
2	12.168	217.4	12.4	0.2652	4.743	0.617

3c

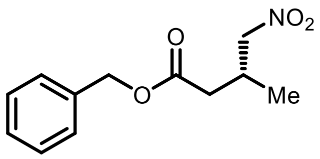

Racemic (CHIRALPAK® IA, hexane/IPA = 99/01, 1 mL/min)

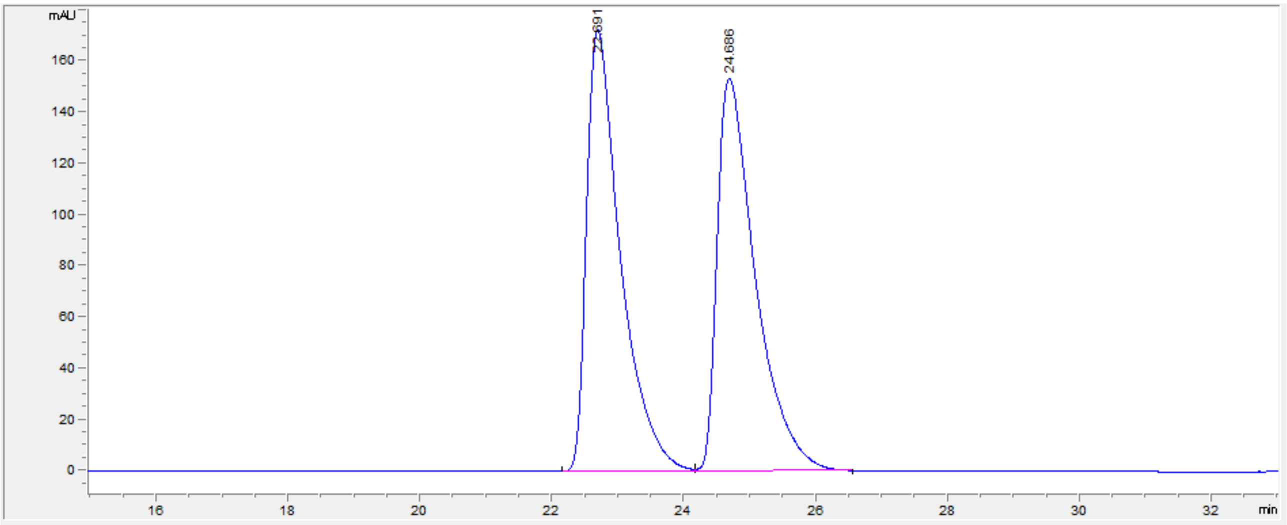

#	Time	Area	Height	Width	Area%	Symmetry
1	22.691	6163.9	172.2	0.5317	49.881	0.451
2	24.686	6193.3	153.2	0.6015	50.119	0.428

Enantioenriched (CHIRALPAK® IA, hexane/IPA = 99/01, 1 mL/min)

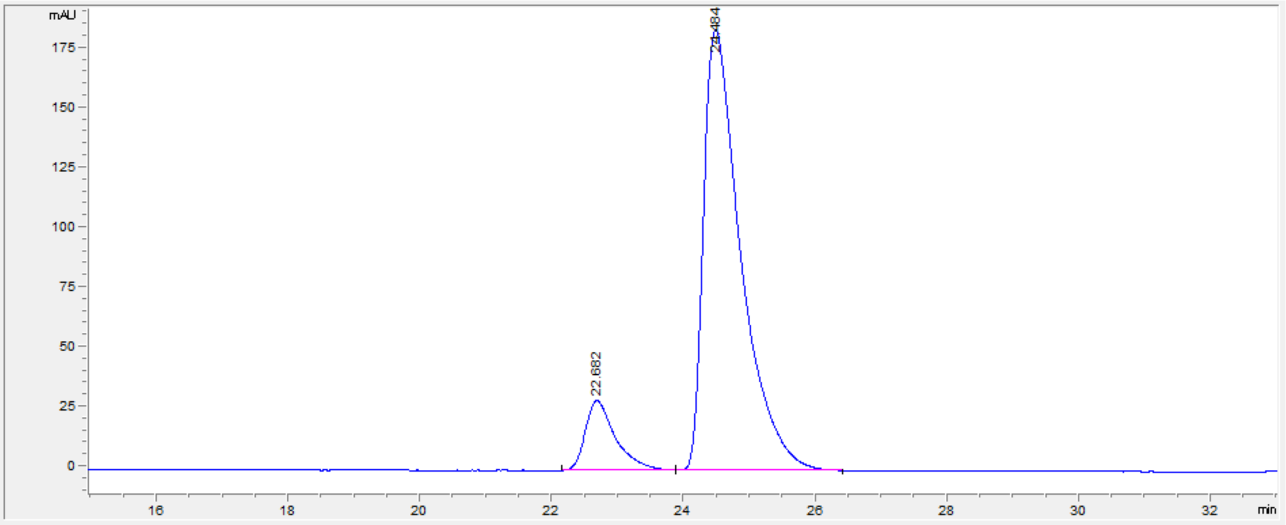

#	Time	Area	Height	Width	Area%	Symmetry
1	22.682	935.5	29.2	0.4759	11.410	0.576
2	24.484	7263.8	184.3	0.5898	88.590	0.438

[ToC]

3e

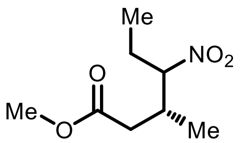

Racemic (CHIRALPAK® IA, hexane/IPA = 98/02, 1 mL/min)

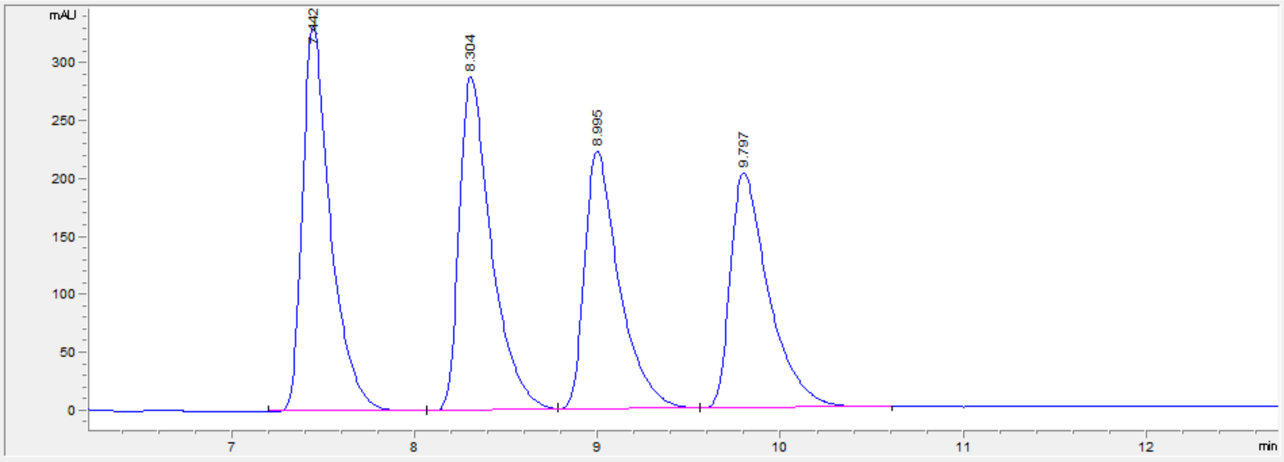

#	Time	Area	Height	Width	Area%	Symmetry
1	7.442	3544.7	331.9	0.1586	27.230	0.577
2	8.304	3529.6	288.2	0.1829	27.114	0.53
3	8.995	2952.5	223.1	0.1964	22.681	0.535
4	9.797	2990.9	203.7	0.2193	22.976	0.504

Enantioenriched (CHIRALPAK® IA, hexane/IPA = 98/02, 1 mL/min)

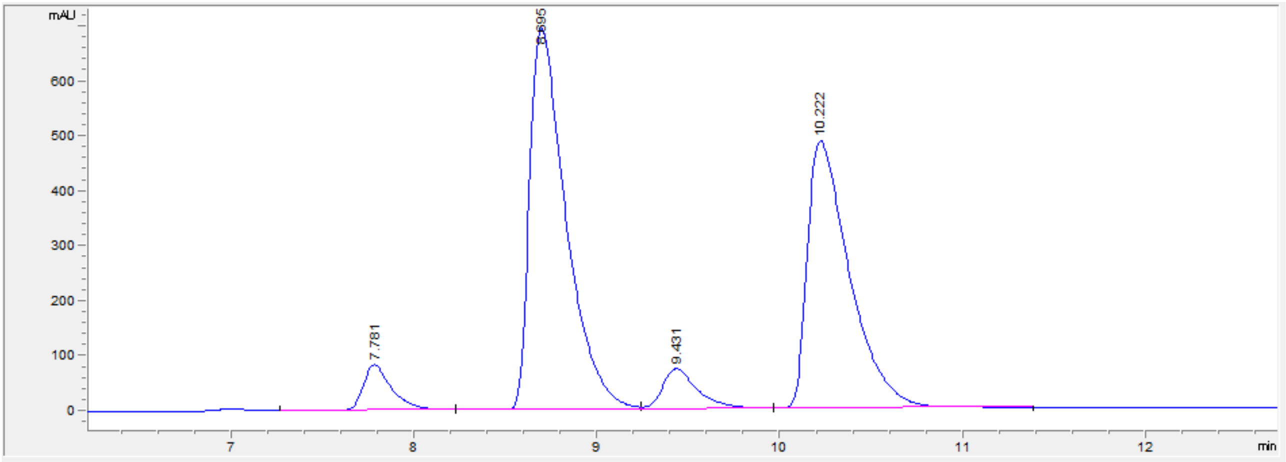

#	Time	Area	Height	Width	Area%	Symmetry
1	7.781	918	83.1	0.1629	4.723	0.604
2	8.695	9624	696.1	0.2073	49.517	0.452
3	9.431	997.2	73.6	0.198	5.131	0.578
4	10.222	7896.6	487.1	0.2451	40.629	0.427

[ToC]

3f

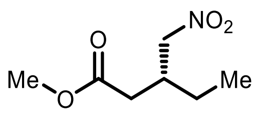

Racemic (CHIRALPAK® IG, from 1% to 20% MeOH in 7 min, then from 20% to 50% in 1 min, 1 mL/min)

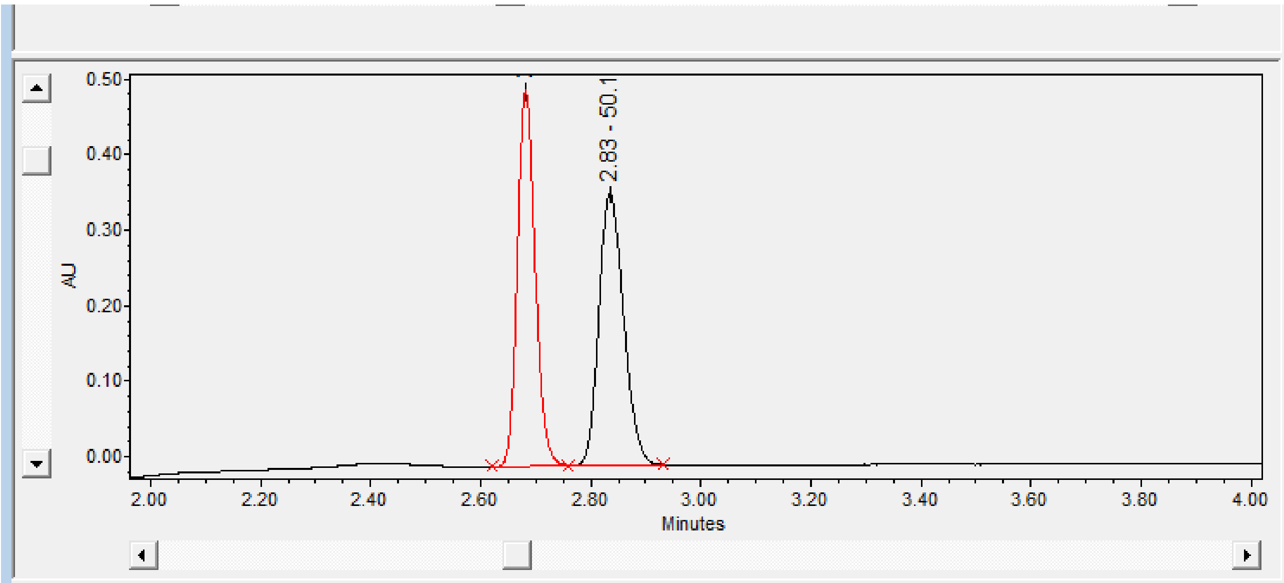

Name	Retention Time (min)	Area (µV*sec)	% Area	Height (µV)	Int Type	Peak Type
1	2.682	1117053	49.92	497068	bv	Unknown
2	2.835	1120832	50.08	358863	vb	Unknown

Enantioenriched (CHIRALPAK® IG, from 1% to 20% MeOH in 7 min, then from 20% to 50% in 1 min, 1 mL/min)

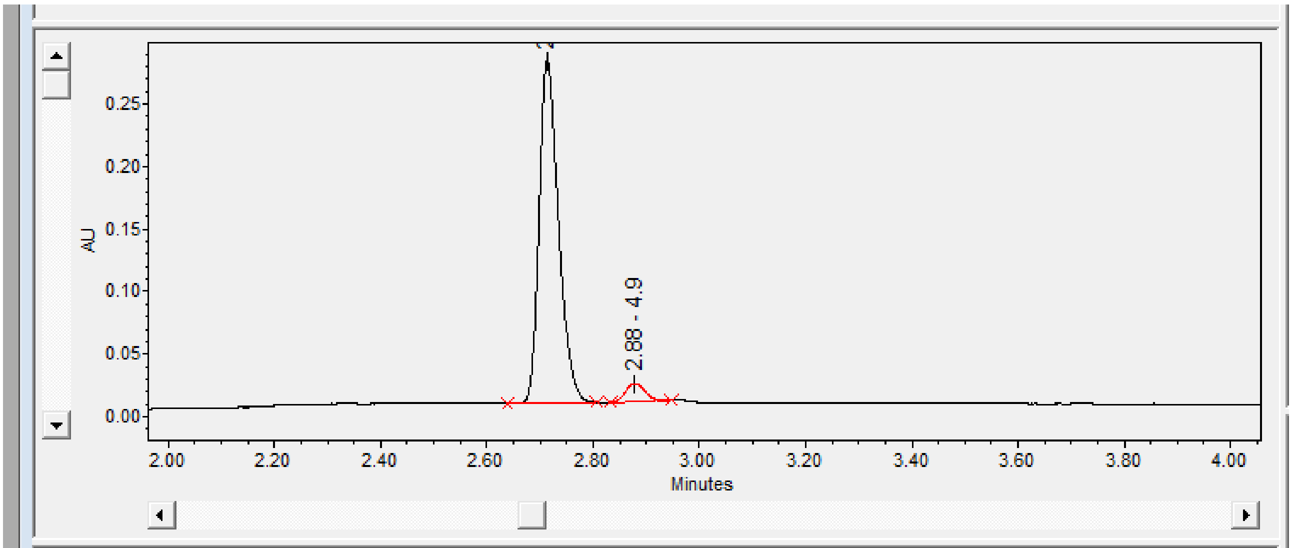

Name	Retention Time (min)	Area (µV*sec)	% Area	Height (µV)	Int Type	Peak Type
1	2.714	695063	95.09	273406	bb	Unknown
2	2.878	35881	4.91	14351	bb	Unknown

3g

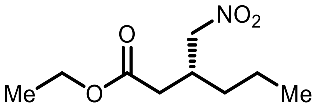

Racemic (CHIRALPAK® IA, hexane/IPA = 99/01, 1 mL/min)

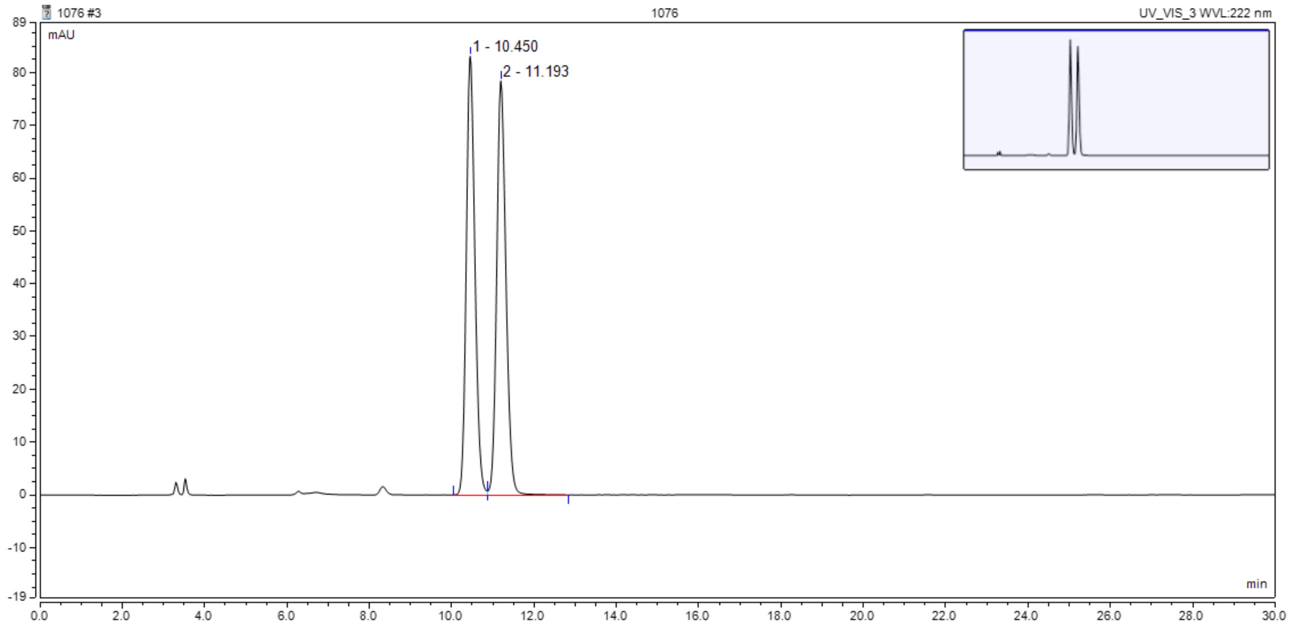

Peak No.	Peak Name	Ret.Time min	Amount n.a.	Rel.Area %	Area mAU*min	Height mAU	Type	Width (50%) min	Asym. EP	Resol. EP	Plates EP
1		10.450	n.a.	49.70	20.1411	83.13	M	0.223	1.16	1.91	12160
2		11.193	n.a.	50.30	20.3869	78.46	M	0.237	1.16	n.a.	12330
Maximum			0.0000	50.30	20.3869	83.13		0.237	1.16	1.91	12330
Minimum			0.0000	49.70	20.1411	78.46		0.223	1.16	1.91	12160
Sum			0.0000	100.00	40.5280	161.60					

Enantioenriched (CHIRALPAK® IA, hexane/IPA = 99/01, 1 mL/min)

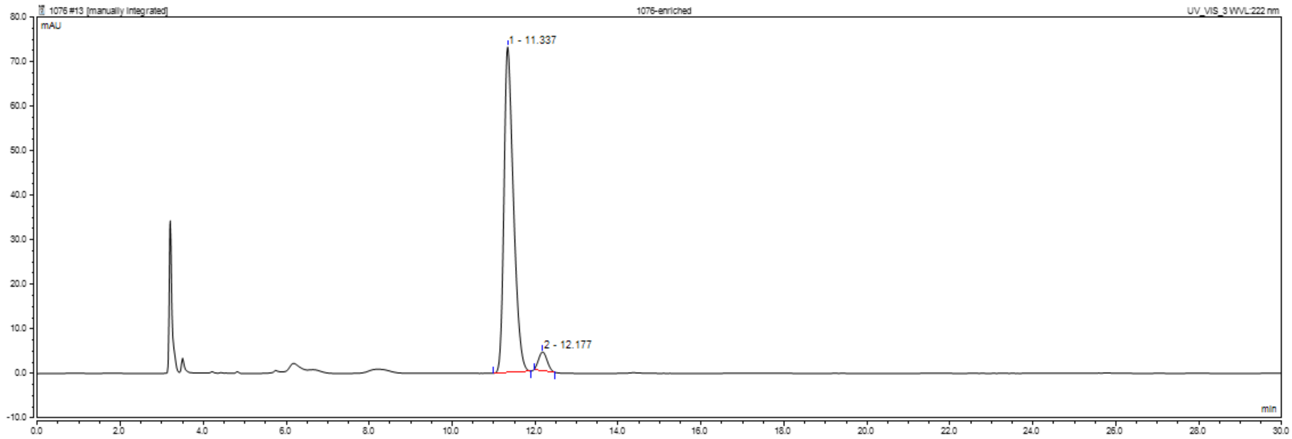

Peak No.	Peak Name	Ret.Time min	Amount n.a.	Rel.Area %	Area mAU*min	Height mAU	Type	Width (50%) min	Asym. EP	Resol. EP	Plates EP
1		11.337	n.a.	95.02	19.2968	73.06	BMB*	0.241	1.40	2.10	12237
2		12.177	n.a.	4.98	1.0107	4.25	BMB*	0.230	1.14	n.a.	15506
Maximum			0.0000	95.02	19.2968	73.06		0.241	1.40	2.10	15506
Minimum			0.0000	4.98	1.0107	4.25		0.230	1.14	2.10	12237
Sum			0.0000	100.00	20.3075	77.31					

3h

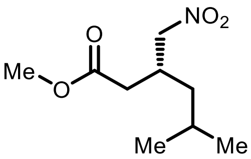

Racemic (CHIRALPAK® AS-H, hexane/IPA = 90/10, 1 mL/min)

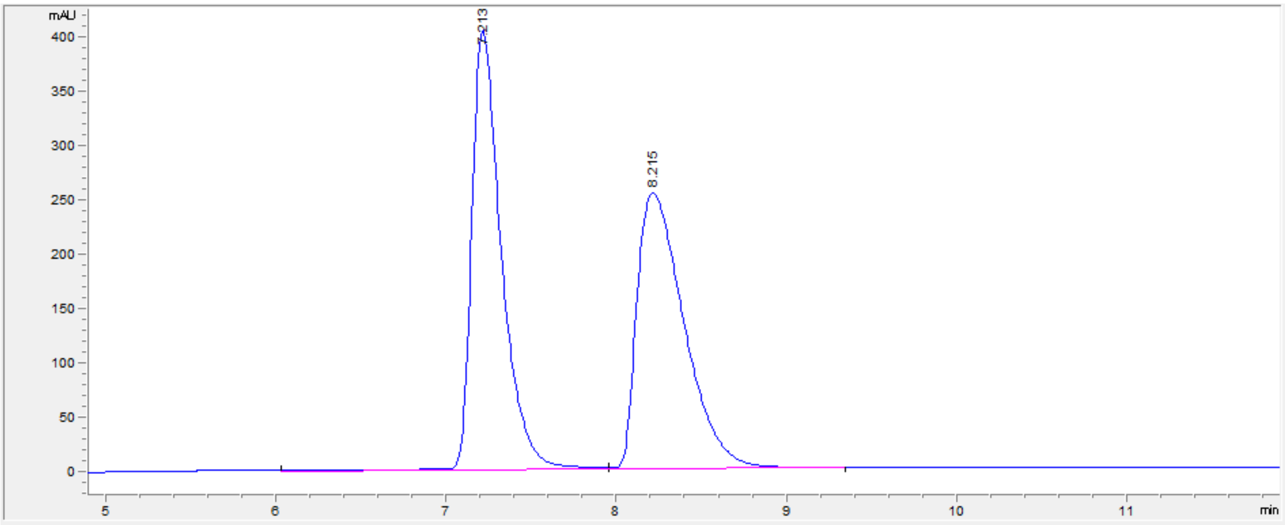

#	Time	Area	Height	Width	Area%	Symmetry
1	7.213	4991.1	405	0.1858	50.271	0.575
2	8.215	4937.4	254.5	0.3023	49.729	0.481

Enantioenriched (CHIRALPAK® AS-H, hexane/IPA = 90/10, 1 mL/min)

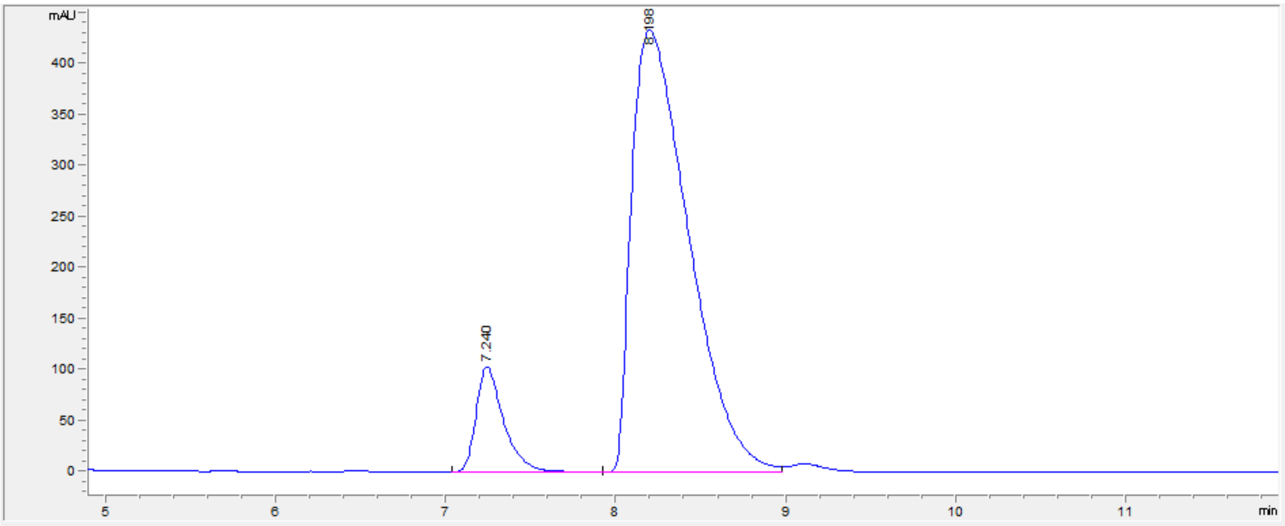

#	Time	Area	Height	Width	Area%	Symmetry
1	7.24	1174.2	103.2	0.1707	10.396	0.601
2	8.198	10120.1	432.8	0.3688	89.604	0.43

[ToC]

3i

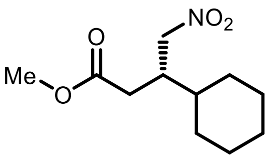

Racemic (CHIRALPAK® AD-H, hexane/IPA = 92/08, 1 mL/min)

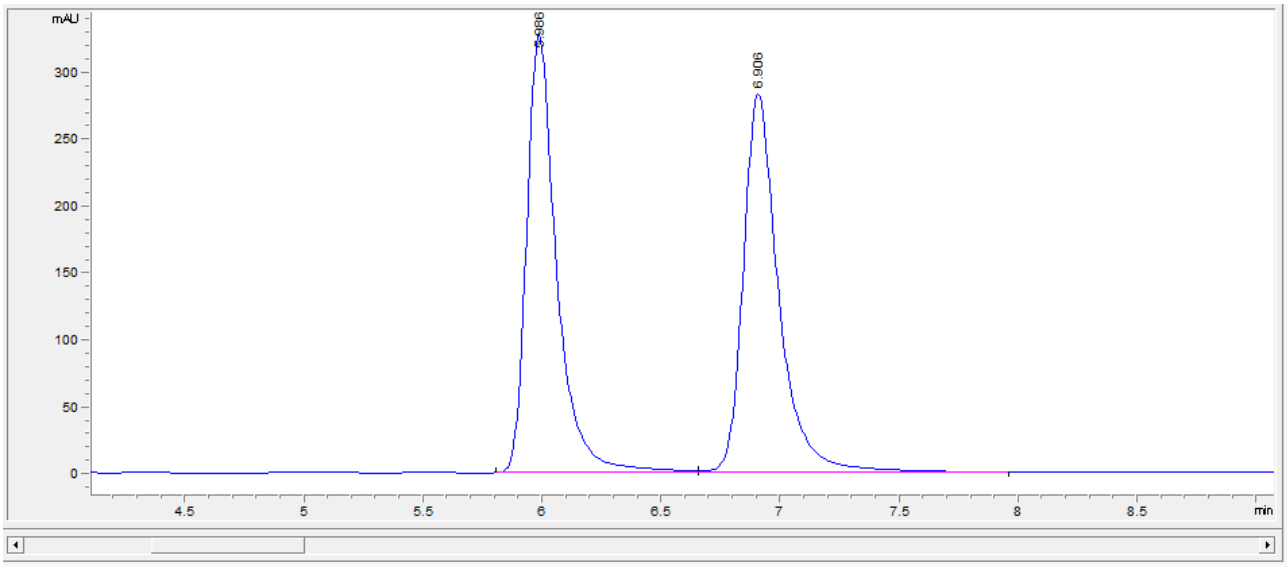

#	Time	Area	Height	Width	Area%	Symmetry
1	5.986	2889.8	328.2	0.1327	49.480	0.625
2	6.906	2950.6	283.2	0.1556	50.520	0.651

Enantioenriched (CHIRALPAK® AD-H, hexane/IPA = 92/08, 1 mL/min)

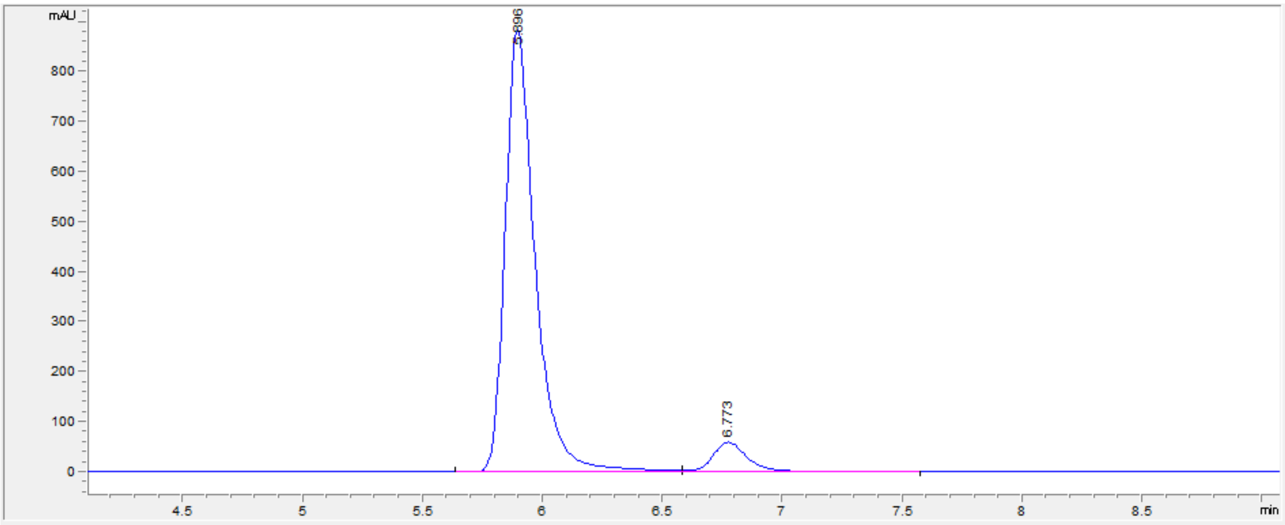

#	Time	Area	Height	Width	Area%	Symmetry
1	5.896	7699.9	884.2	0.1316	92.484	0.645
2	6.773	625.8	59.8	0.1561	7.516	0.697

3j

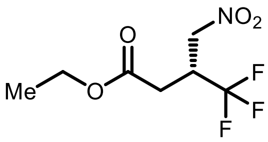

Racemic (CHIRALPAK® IA, hexane/IPA = 98/02, 1 mL/min)

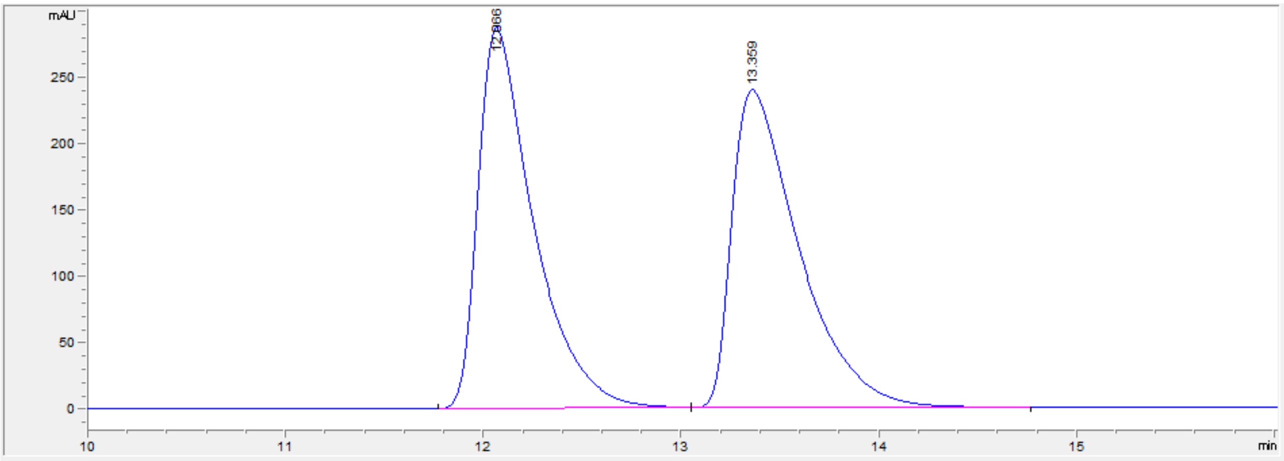

#	Time	Area	Height	Width	Area%	Symmetry
1	12.066	5624.4	287.4	0.2904	49.741	0.467
2	13.359	5682.9	239.9	0.3544	50.259	0.407

Enantioenriched (CHIRALPAK® IA, hexane/IPA = 98/02, 1 mL/min)

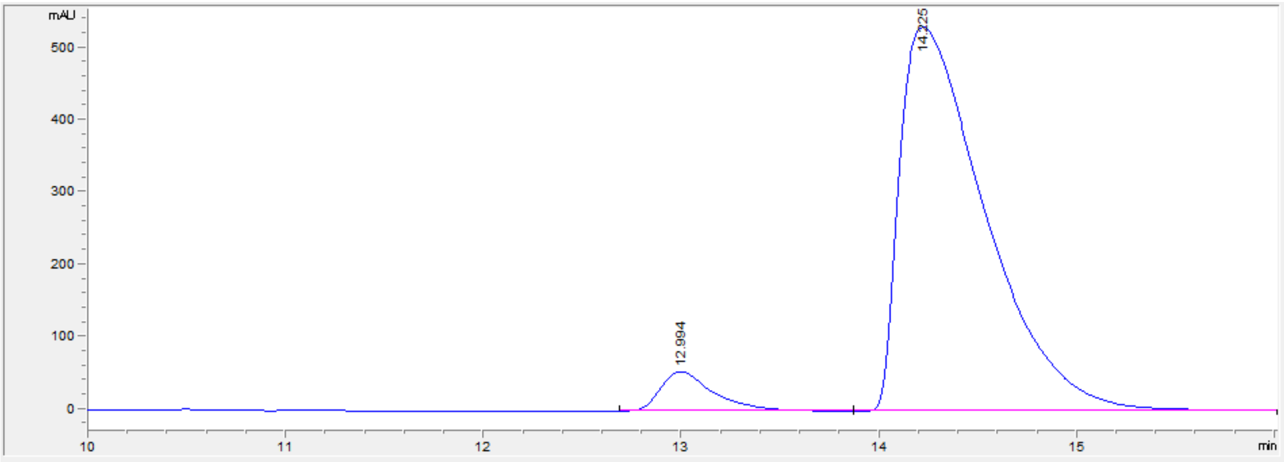

#	Time	Area	Height	Width	Area%	Symmetry
1	12.994	1010.3	54.5	0.2722	5.926	0.552
2	14.225	16039.7	530.4	0.4628	94.074	0.361

[ToC]

3jS

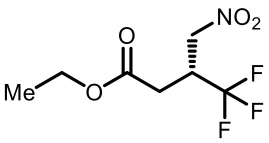

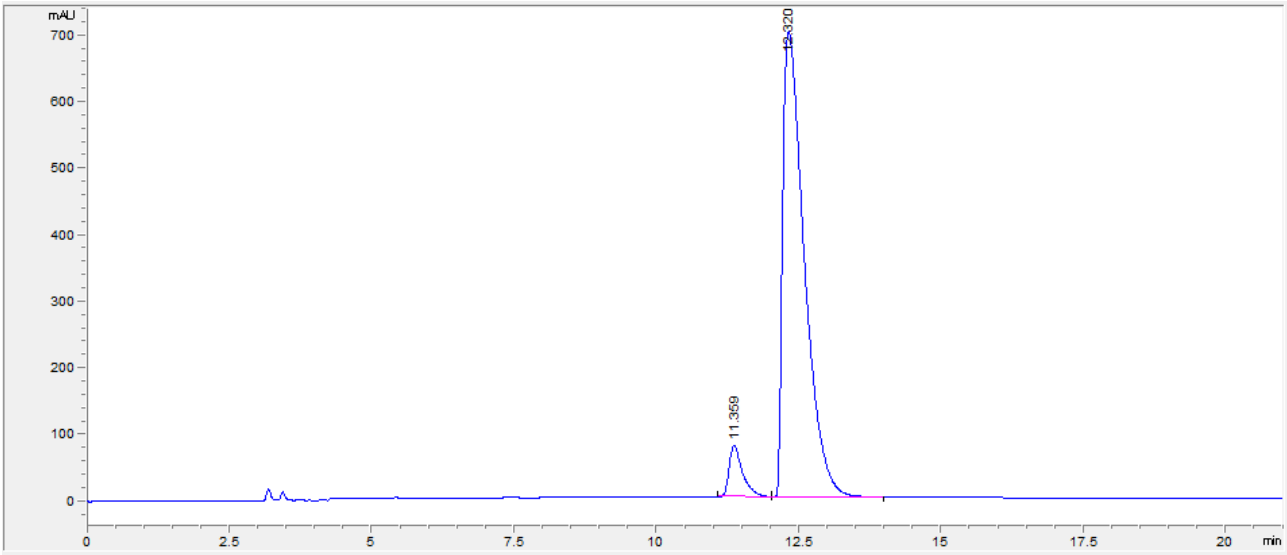

#	Time	Area	Height	Width	Area%	Symmetry
1	11.359	1277.1	77.6	0.2421	6.485	0.538
2	12.32	18415.7	699.2	0.3993	93.515	0.355

[ToC]

3k

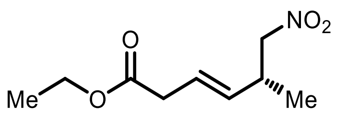

Racemic (CHIRALPAK® AS-H, hexane/IPA = 95/05, 1 mL/min)

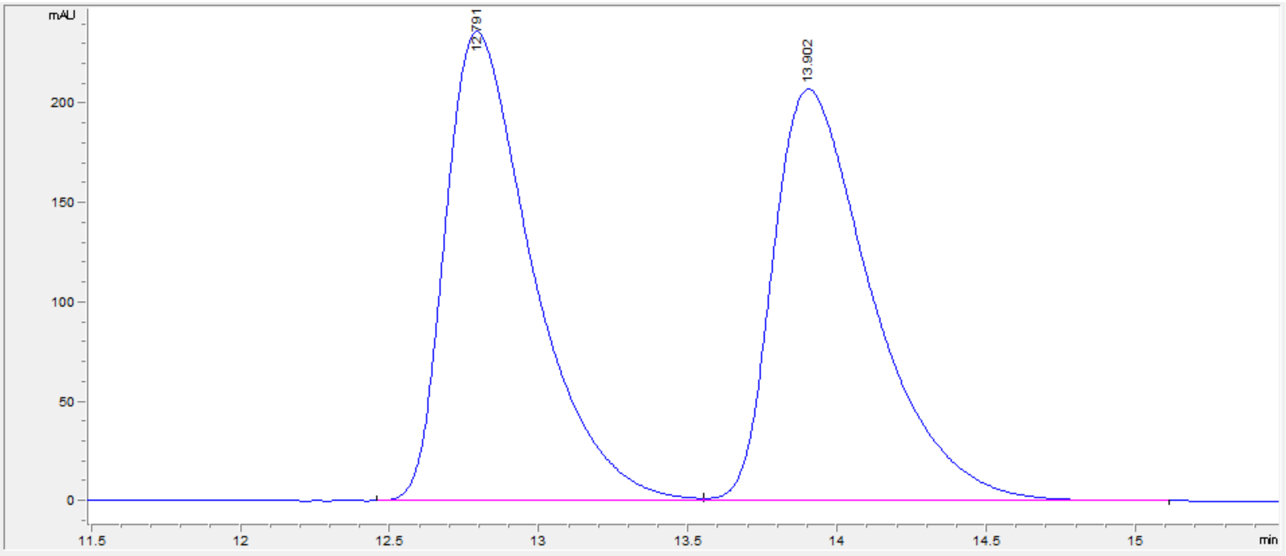

#	Time	Area	Height	Width	Area%	Symmetry
1	12.791	4793.8	236.2	0.3067	50.056	0.53
2	13.902	4783	207.3	0.3491	49.944	0.523

Enantioenriched (CHIRALPAK® AS-H, hexane/IPA = 95/05, 1 mL/min)

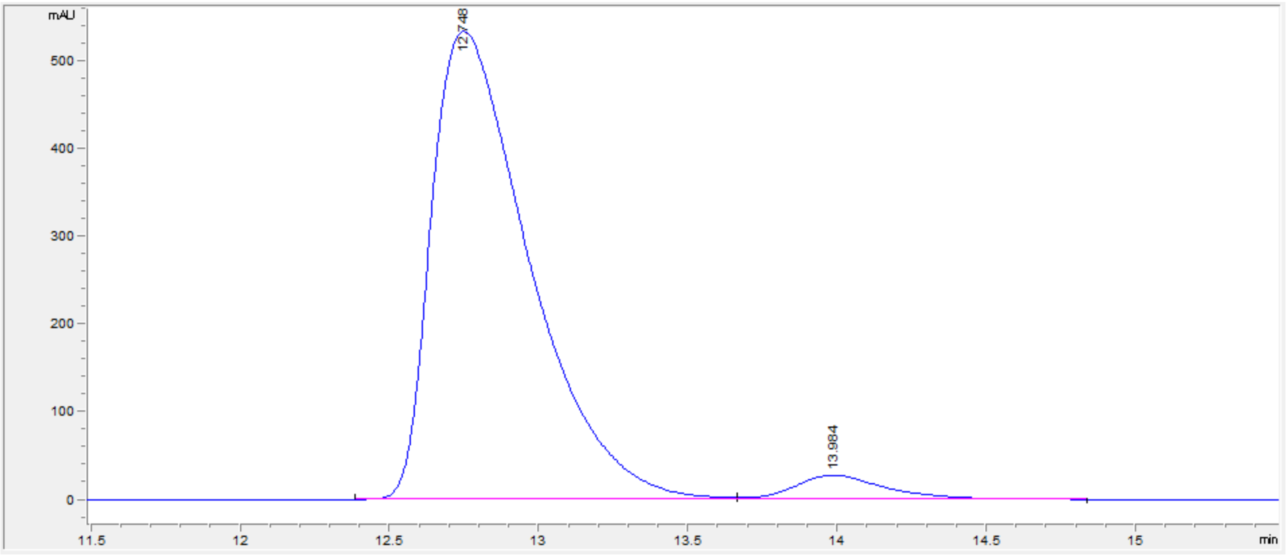

#	Time	Area	Height	Width	Area%	Symmetry
1	12.748	12155.5	533.3	0.3479	95.450	0.462
2	13.984	579.4	27.4	0.3145	4.550	0.679

3l

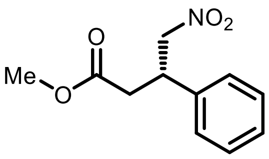

Racemic (CHIRALPAK® AS-H, hexane/IPA = 95/05, 1 mL/min)

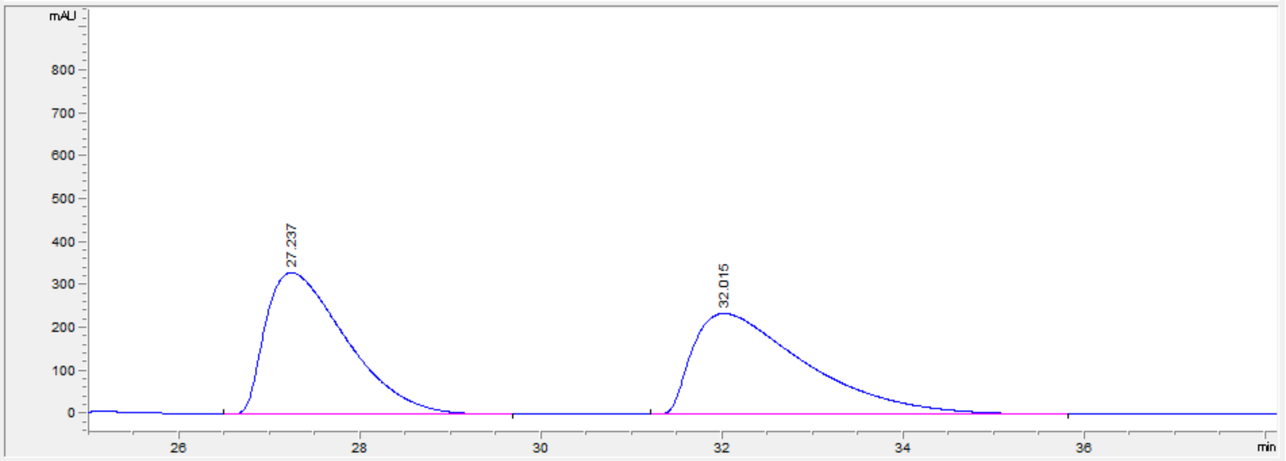

#	Time	Area	Height	Width	Area%	Symmetry
1	27.237	20381	329.6	0.9653	49.881	0.442
2	32.015	20478.2	235.1	1.2924	50.119	0.354

Enantioenriched (CHIRALPAK® AS-H, hexane/IPA = 95/05, 1 mL/min)

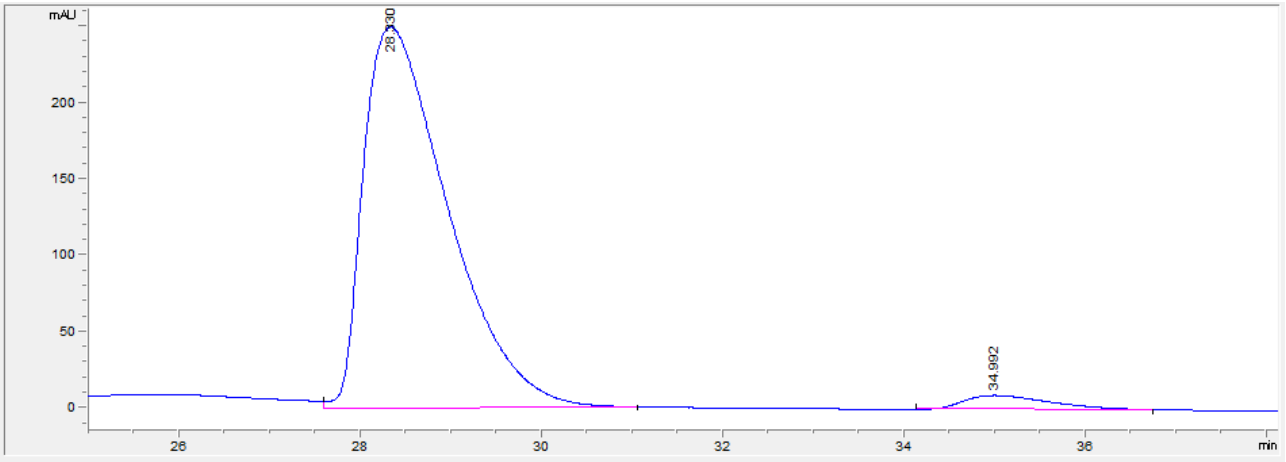

#	Time	Area	Height	Width	Area%	Symmetry
1	28.33	16304.2	250	0.9968	96.346	0.44
2	34.992	618.3	9.3	0.9522	3.654	0.507

3m

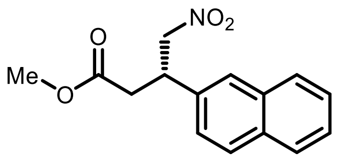

Racemic (CHIRALPAK® AS-H, hexane/IPA = 85/15, 1 mL/min)

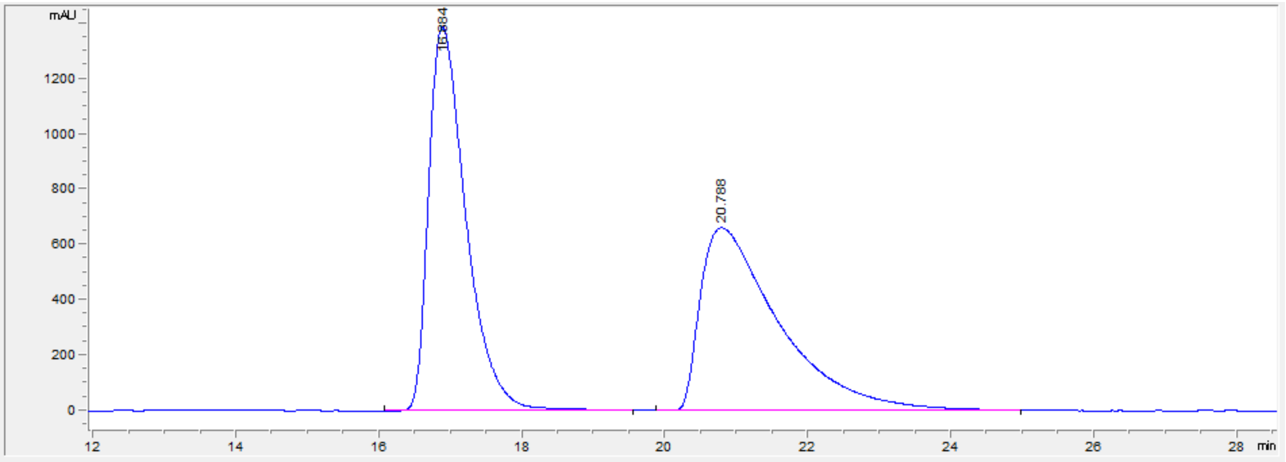

#	Time	Area	Height	Width	Area%	Symmetry
1	16.884	50793.6	1388.9	0.5587	49.888	0.538
2	20.788	51021.2	662.4	1.1207	50.112	0.336

Enantioenriched (CHIRALPAK® AS-H, hexane/IPA = 85/15, 1 mL/min)

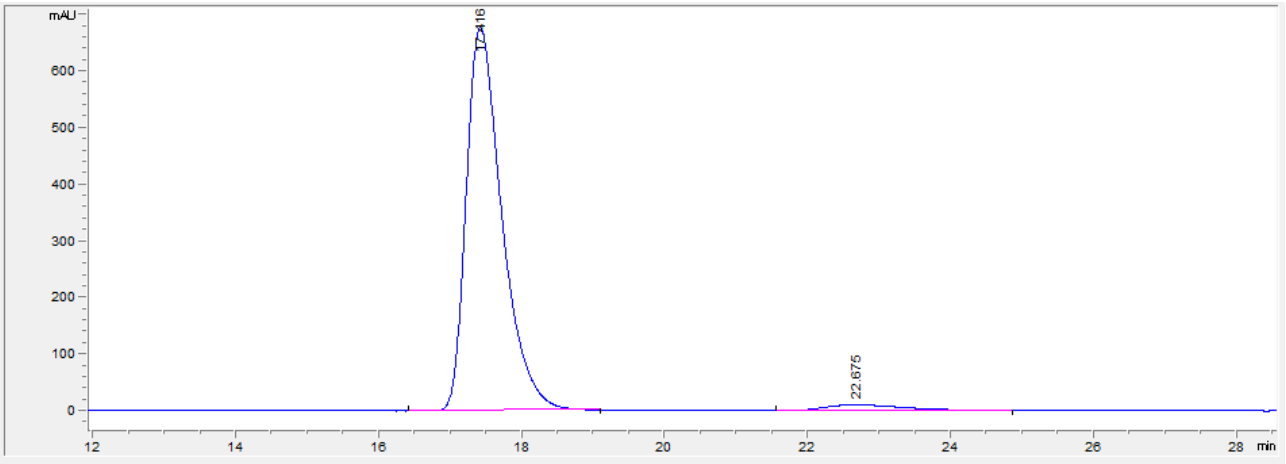

#	Time	Area	Height	Width	Area%	Symmetry
1	17.416	23556.2	676.8	0.532	96.563	0.598
2	22.675	838.4	10.5	0.9478	3.437	0.579

[ToC]

3n

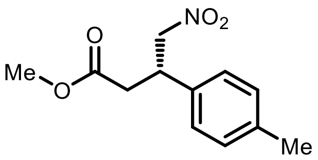

Racemic (CHIRALPAK® AS-H, hexane/IPA = 85/15, 1 mL/min)

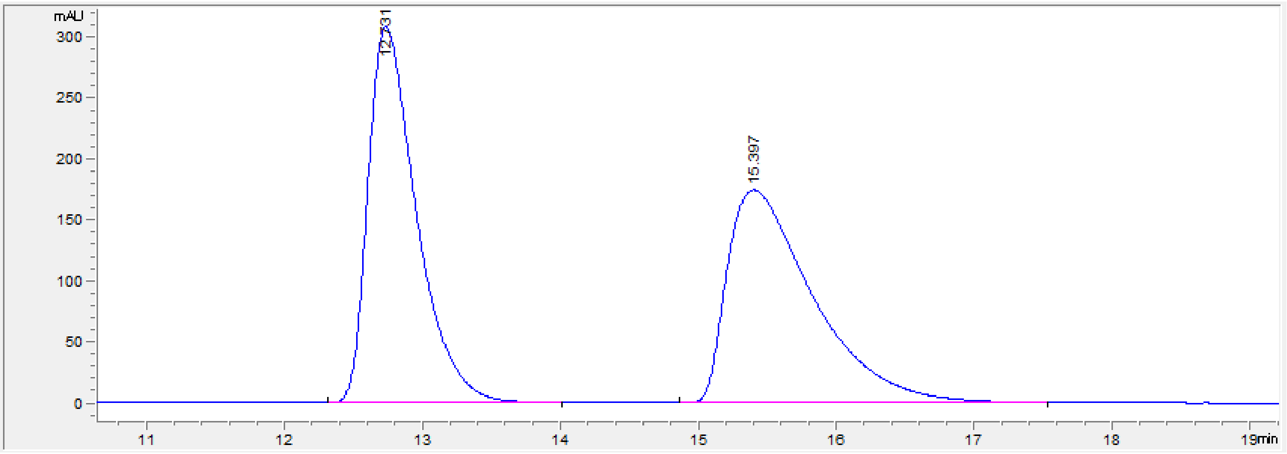

#	Time	Area	Height	Width	Area%	Symmetry
1	12.731	7538.9	308	0.3736	49.978	0.549
2	15.397	7545.6	174.4	0.6527	50.022	0.409

Enantioenriched (CHIRALPAK® AS-H, hexane/IPA = 85/15, 1 mL/min)

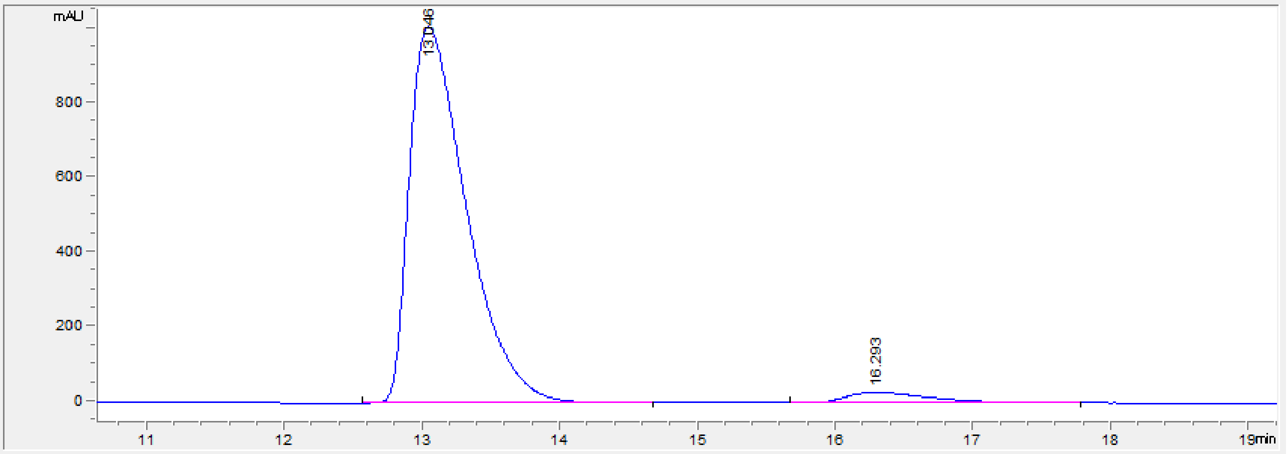

#	Time	Area	Height	Width	Area%	Symmetry
1	13.046	29052.4	1007.1	0.4426	96.244	0.476
2	16.293	1133.8	27	0.6359	3.756	0.506

3o

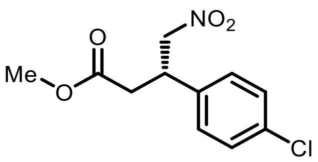

Racemic (CHIRALPAK® AS-H, hexane/IPA = 85/15, 1 mL/min)

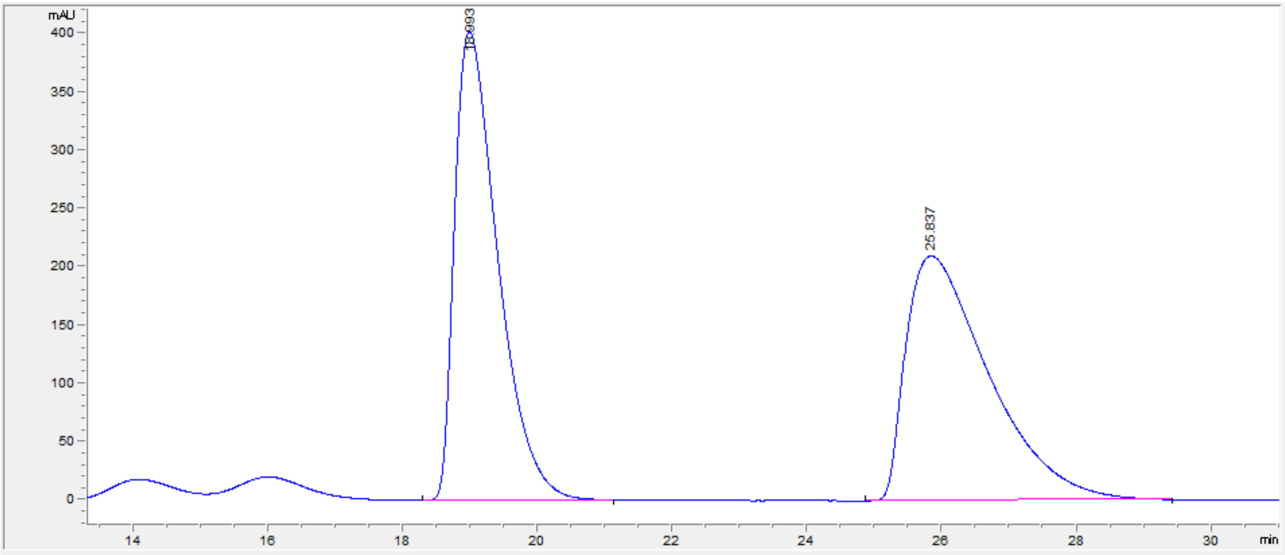

#	Time	Area	Height	Width	Area%	Symmetry
1	18.993	17972.3	402.1	0.6895	50.041	0.482
2	25.837	17942.7	209.8	1.2618	49.959	0.4

Enantioenriched (CHIRALPAK® AS-H, hexane/IPA = 85/15, 1 mL/min)

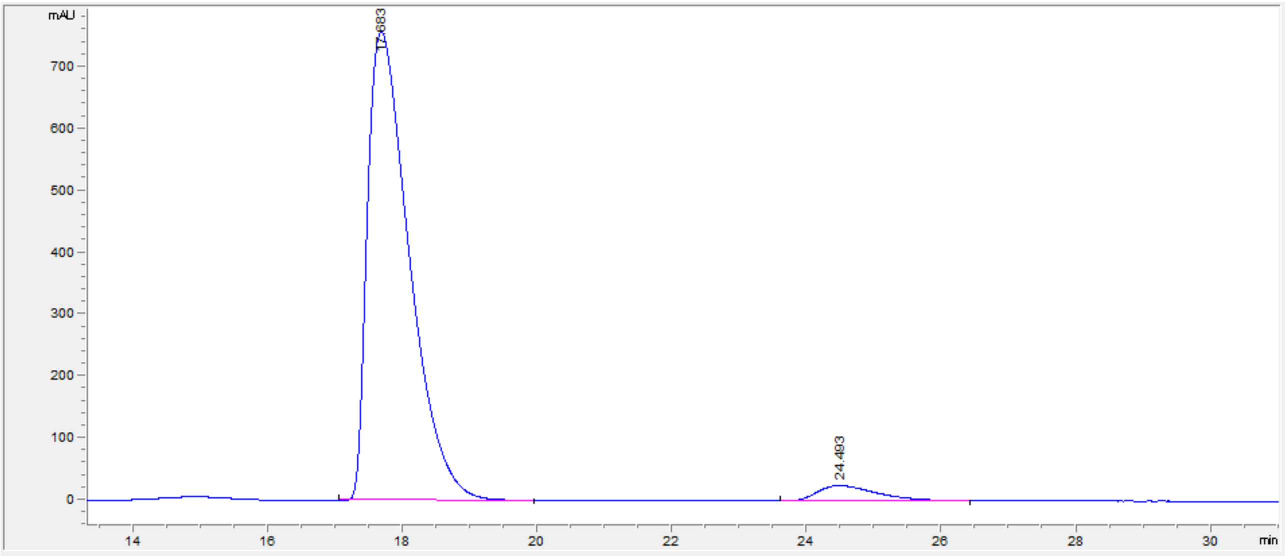

#	Time	Area	Height	Width	Area%	Symmetry
1	17.683	32749.5	758.3	0.6679	95.566	0.451
2	24.493	1519.6	24.8	0.8763	4.434	0.559

[ToC]

3p

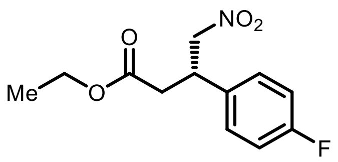

Racemic (CHIRALPAK® AS-H, hexane/IPA = 90/10, 1 mL/min)

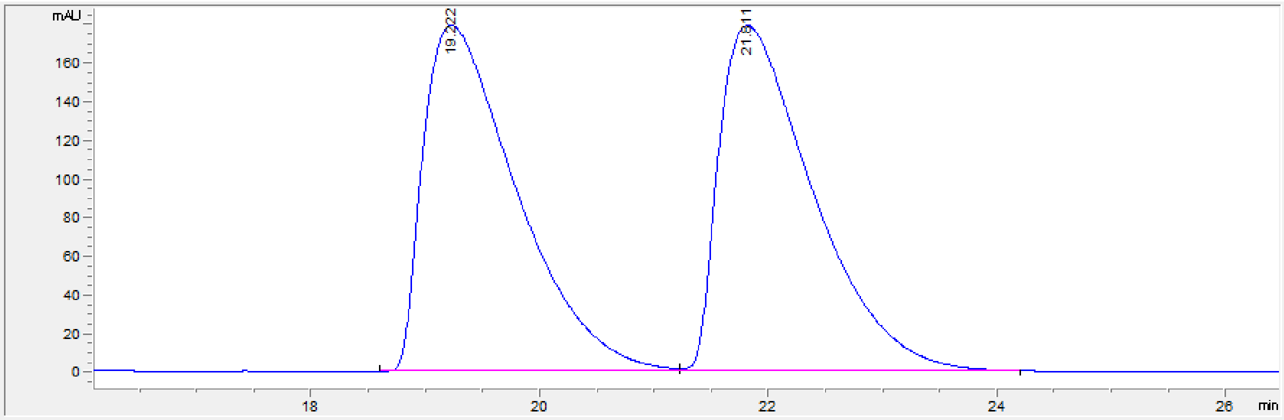

#	Time	Area	Height	Width	Area%	Symmetry
1	19.222	10178	179	0.8635	49.843	0.402
2	21.811	10242	178.9	0.868	50.157	0.405

Enantioenriched (CHIRALPAK® AS-H, hexane/IPA = 90/10, 1 mL/min)

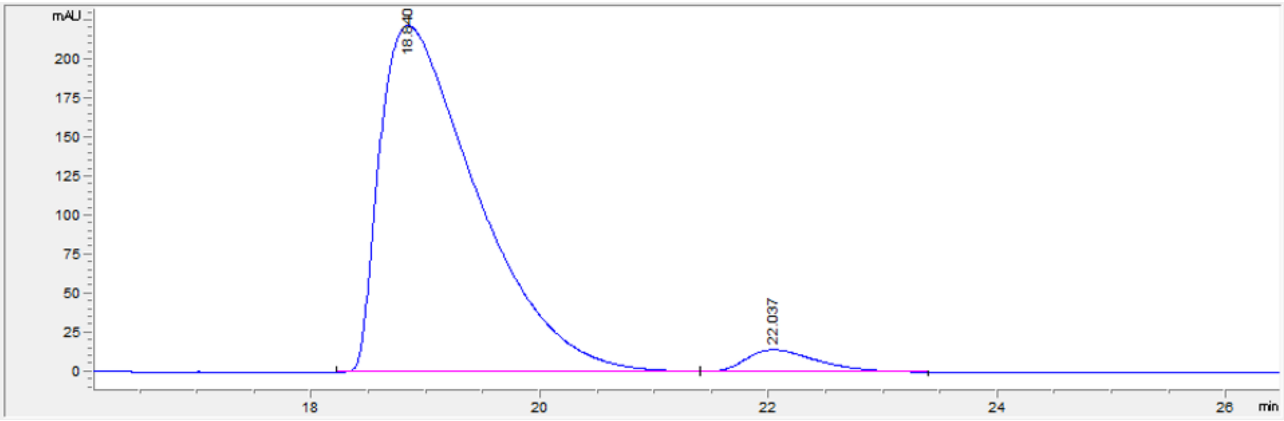

#	Time	Area	Height	Width	Area%	Symmetry
1	18.84	13155.1	222.1	0.8911	95.403	0.376
2	22.037	633.9	14.3	0.6584	4.597	0.594

[ToC]

3q

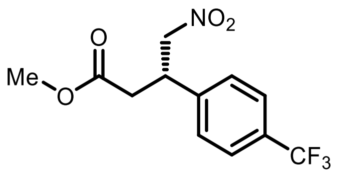

Racemic (CHIRALPAK® AS-H, hexane/IPA = 80/20, 1 mL/min)

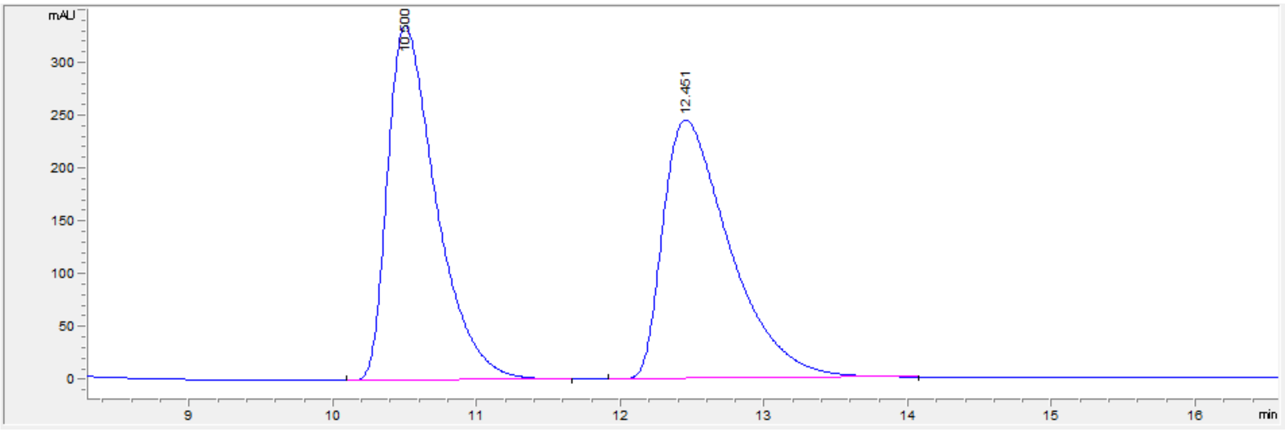

#	Time	Area	Height	Width	Area%	Symmetry
1	10.5	7919.9	335.5	0.3594	49.829	0.534
2	12.451	7974.2	244.9	0.4938	50.171	0.476

Enantioenriched (CHIRALPAK® AS-H, hexane/IPA = 80/20, 1 mL/min)

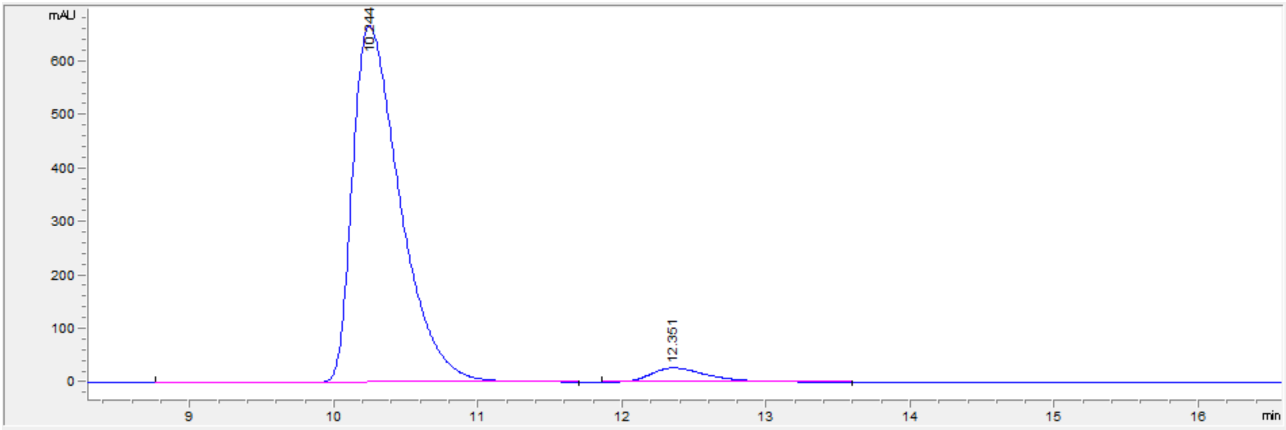

#	Time	Area	Height	Width	Area%	Symmetry
1	10.244	15403.5	667.1	0.3534	95.304	0.525
2	12.351	758.9	26.8	0.4344	4.696	0.612

[ToC]

3s

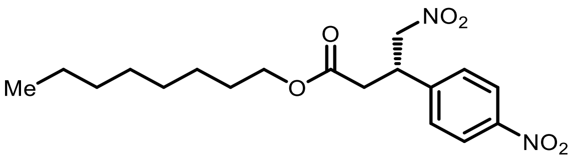

Racemic (CHIRALPAK® AS-H, hexane/IPA = 85/15, 1 mL/min)

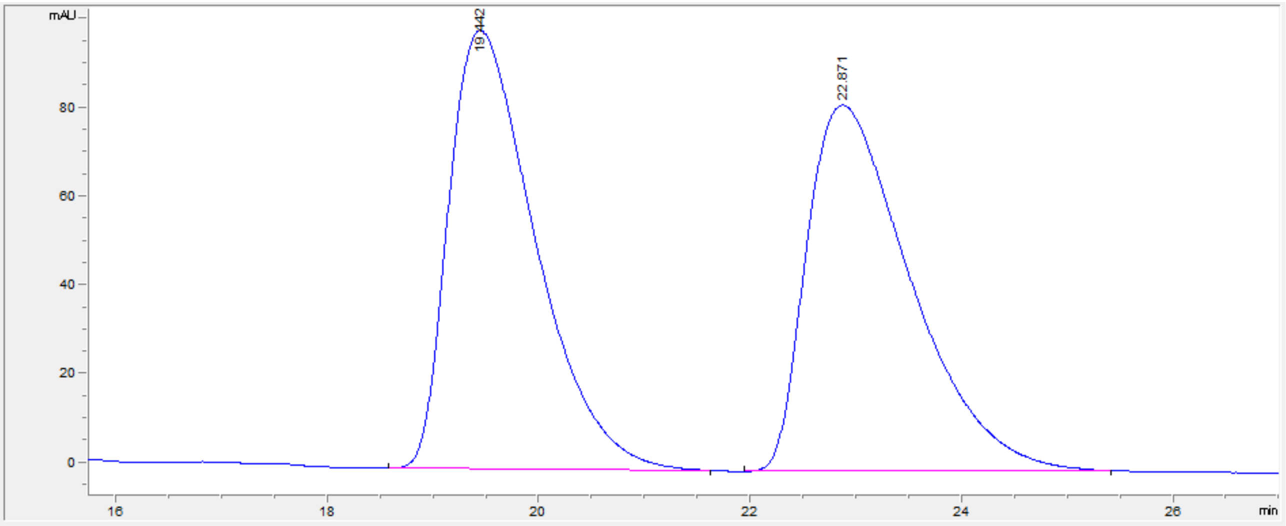

#	Time	Area	Height	Width	Area%	Symmetry
1	19.442	5646.5	98.8	0.8769	49.921	0.533
2	22.871	5664.4	82.5	1.0434	50.079	0.496

Enantioenriched (CHIRALPAK® AS-H, hexane/IPA = 85/15, 1 mL/min)

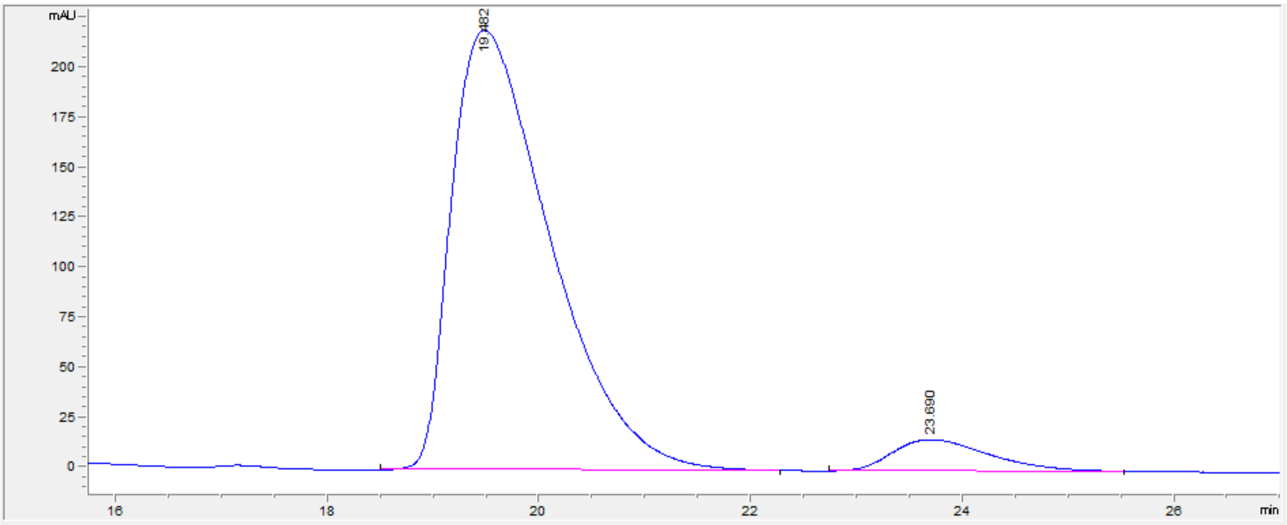

#	Time	Area	Height	Width	Area%	Symmetry
1	19.482	14343.6	219.9	0.9991	93.398	0.465
2	23.69	1013.8	15.8	0.9	6.602	0.595

[ToC]

3t

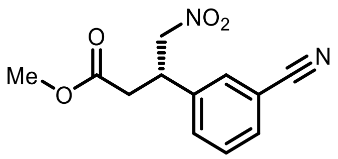

Racemic (CHIRALPAK® IB, hexane/IPA = 80/20, 1 mL/min)

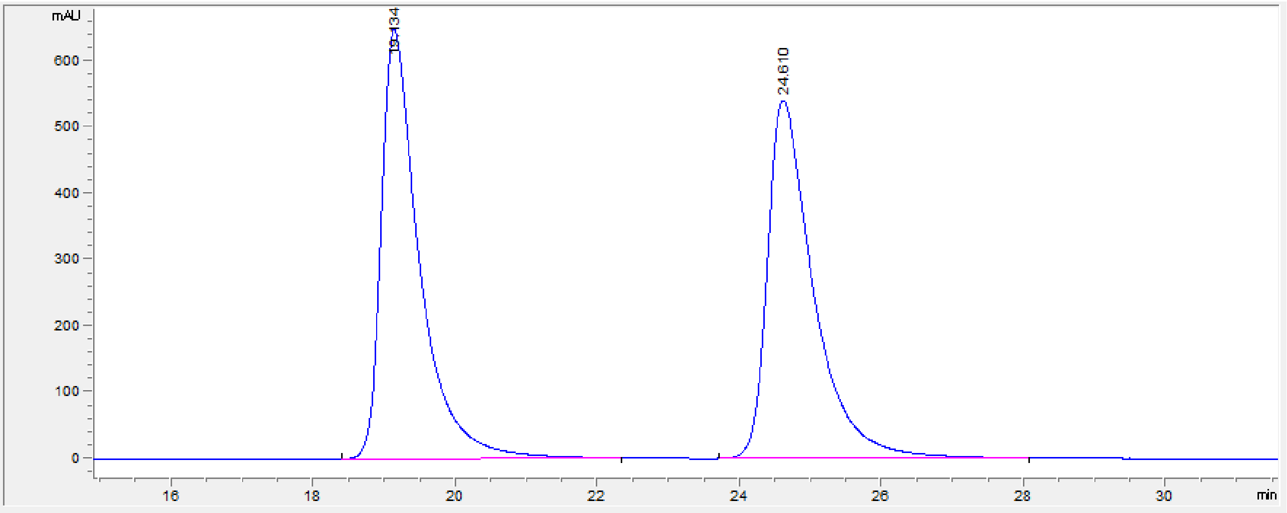

#	Time	Area	Height	Width	Area%	Symmetry
1	19.134	24411	648.3	0.5513	49.970	0.466
2	24.61	24440.5	540.3	0.6658	50.030	0.481

Enantioenriched (CHIRALPAK® AS-H, hexane/IPA = 80/20, 1 mL/min)

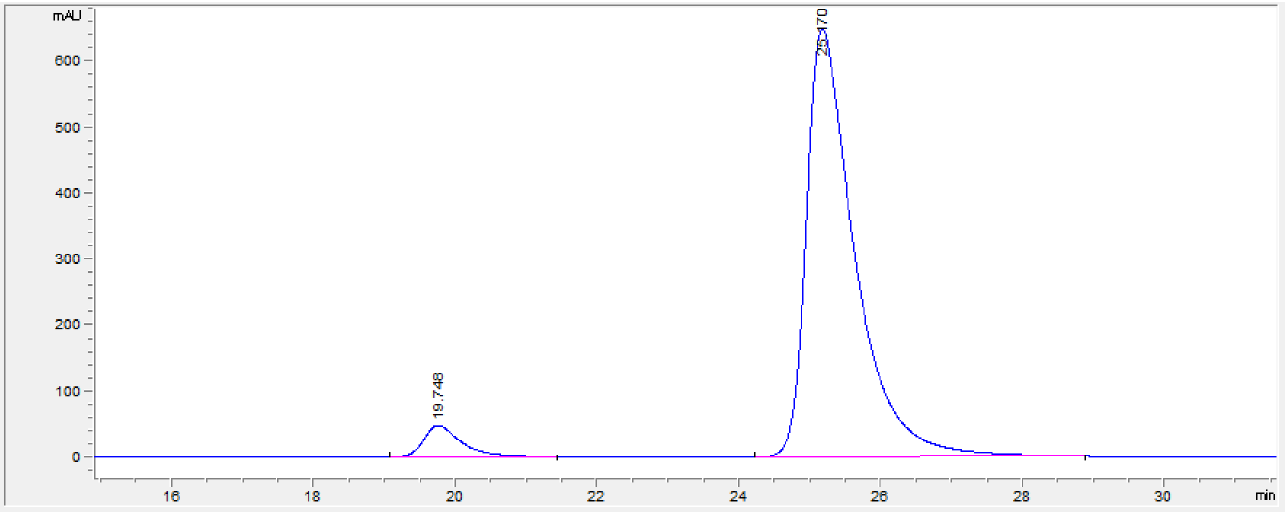

#	Time	Area	Height	Width	Area%	Symmetry
1	19.748	1800.8	47.7	0.556	5.500	0.566
2	25.17	30938	648	0.701	94.500	0.454

[ToC]

3u

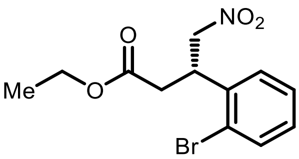

Racemic (CHIRALPAK® IA, hexane/IPA = 95/05, 1 mL/min)

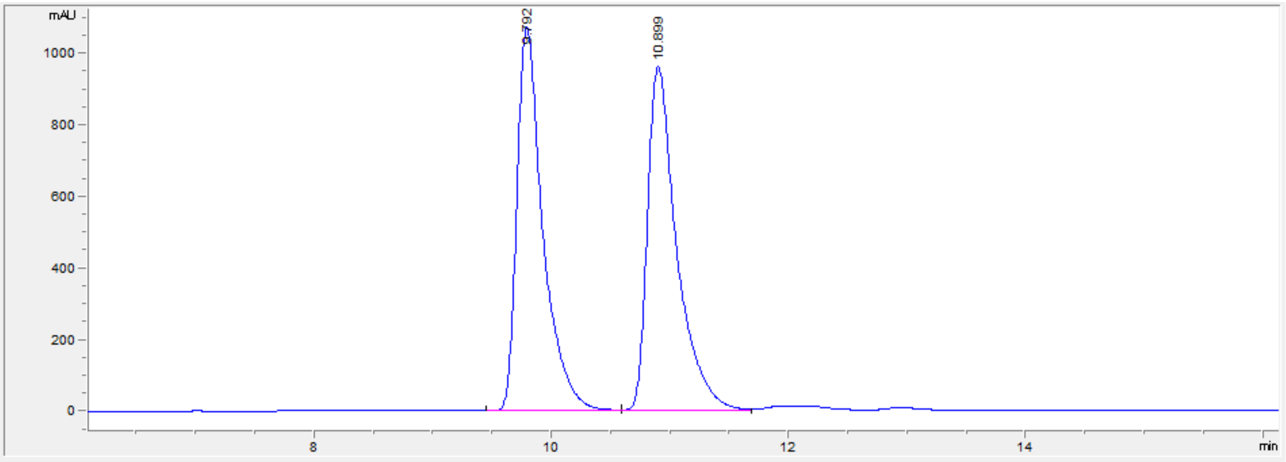

#	Time	Area	Height	Width	Area%	Symmetry
1	9.792	16366	1072.4	0.2261	49.708	0.562
2	10.899	16558	963.4	0.2545	50.292	0.531

Enantioenriched (CHIRALPAK® IA, hexane/IPA = 95/05, 1 mL/min)

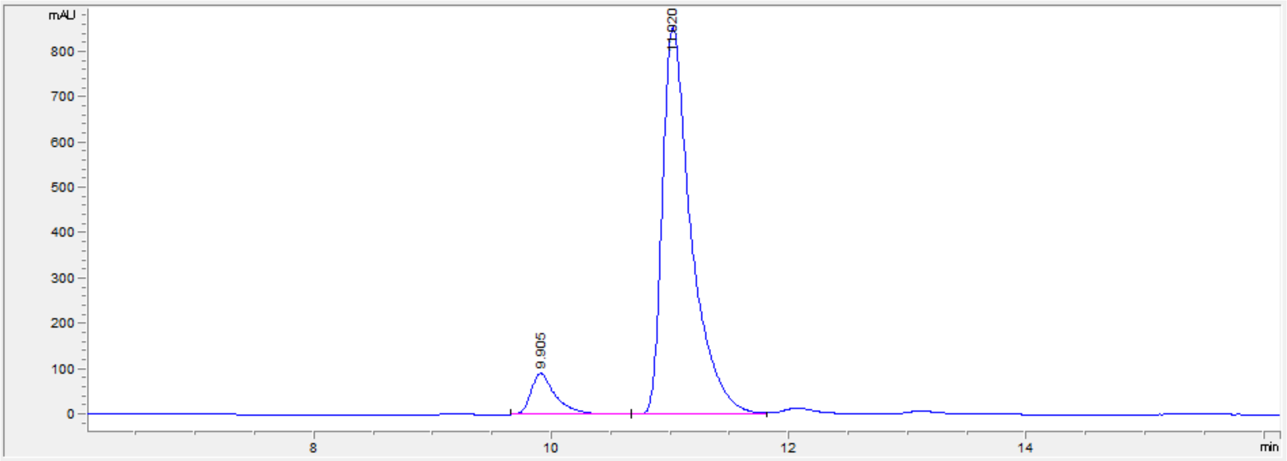

#	Time	Area	Height	Width	Area%	Symmetry
1	9.905	1311.7	90.6	0.2129	8.323	0.575
2	11.02	14448	854.7	0.2492	91.677	0.525

[ToC]

3v

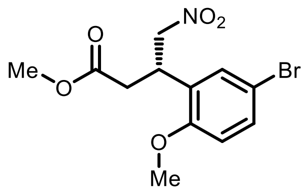

Racemic (CHIRALPAK® IB, hexane/IPA = 80/20, 1 mL/min)

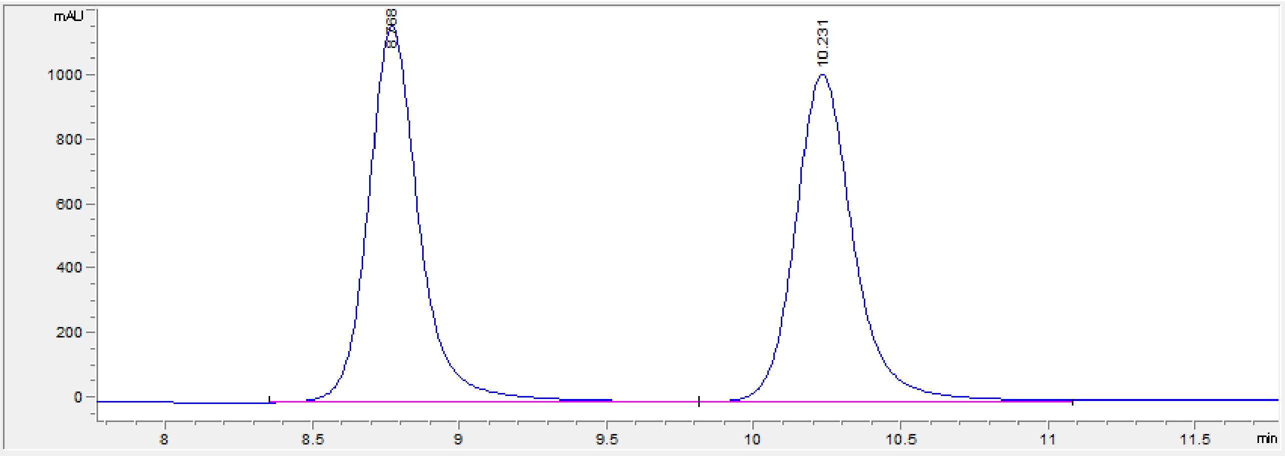

#	Time	Area	Height	Width	Area%	Symmetry
1	8.768	14096.6	1163.3	0.1834	50.060	0.796
2	10.231	14062.9	1017	0.2093	49.940	0.795

Enantioenriched (CHIRALPAK® IB, hexane/IPA = 80/20, 1 mL/min)

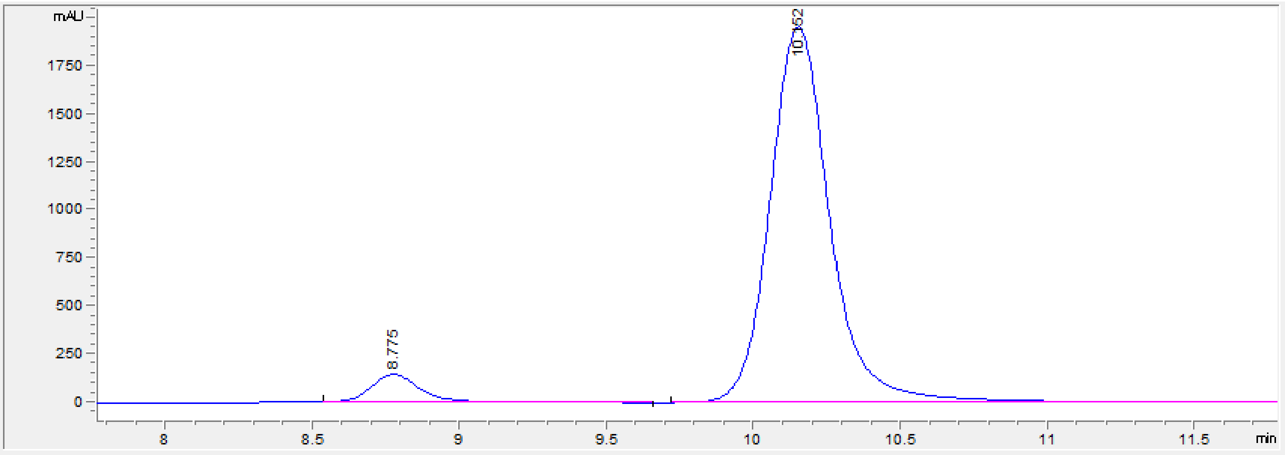

#	Time	Area	Height	Width	Area%	Symmetry
1	8.775	1733.7	144.1	0.1823	5.843	0.778
2	10.152	27938	1954	0.2188	94.157	0.82

[ToC]

3w

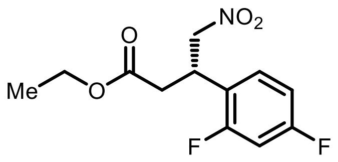

Racemic (CHIRALPAK® AS-H, hexane/IPA = 85/15, 1 mL/min)

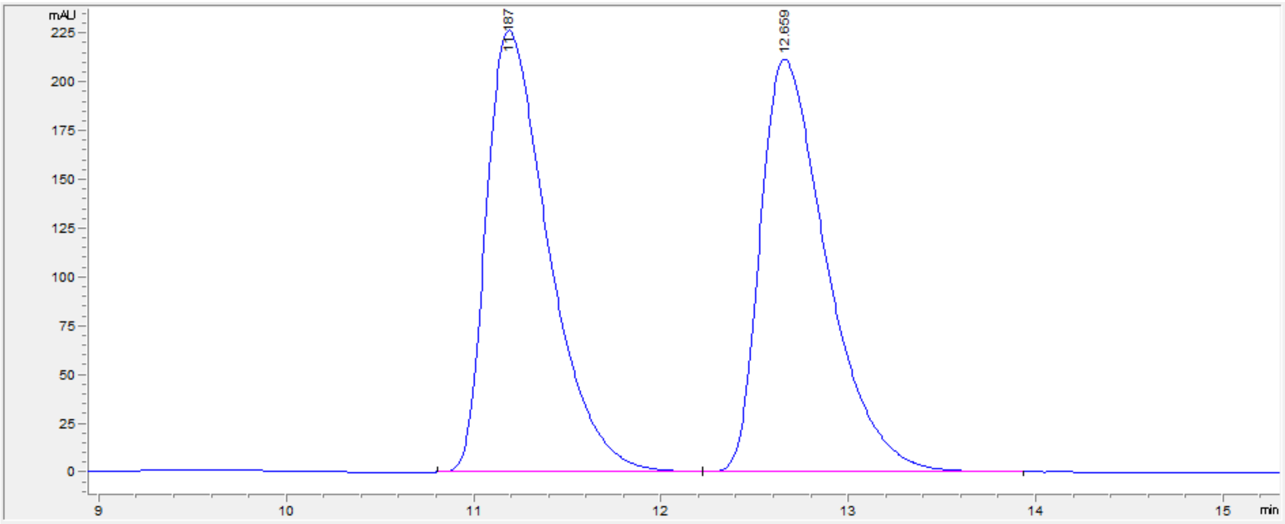

#	Time	Area	Height	Width	Area%	Symmetry
1	11.187	5221.5	226.5	0.351	50.002	0.54
2	12.659	5221	211.6	0.3759	49.998	0.547

Enantioenriched (CHIRALPAK® AS-H, hexane/IPA = 85/15, 1 mL/min)

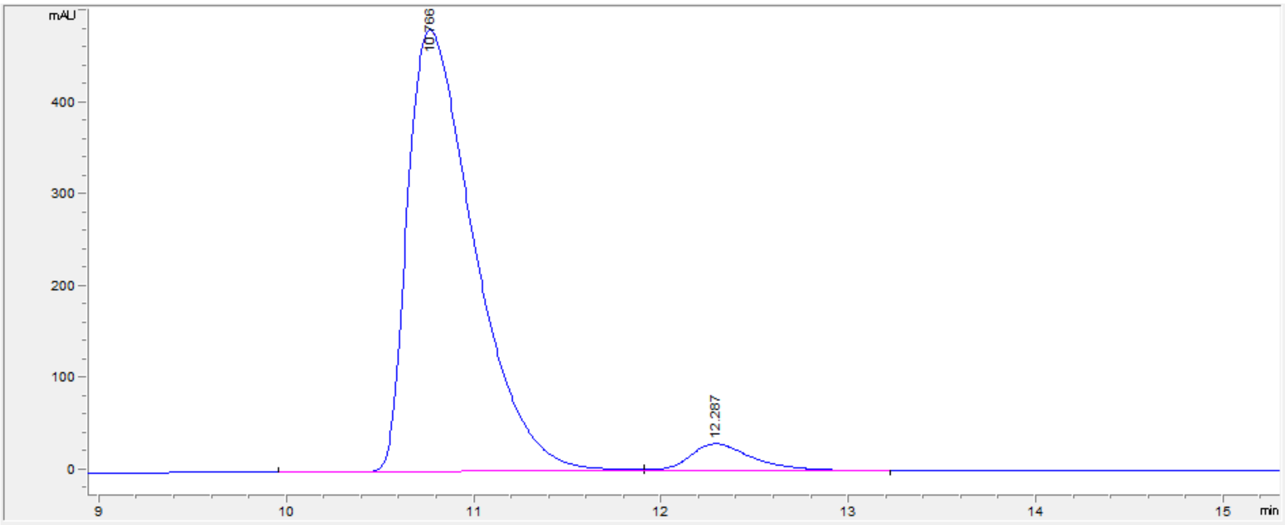

#	Time	Area	Height	Width	Area%	Symmetry
1	10.766	11914.8	480.5	0.3813	94.298	0.478
2	12.287	720.5	29.9	0.3548	5.702	0.648

[ToC]

3x

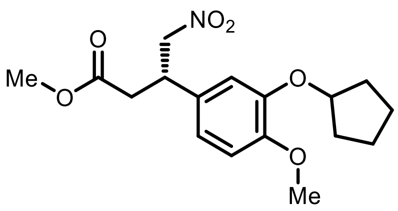

Racemic (CHIRALPAK® AS-H, hexane/IPA = 80/20, 1 mL/min)

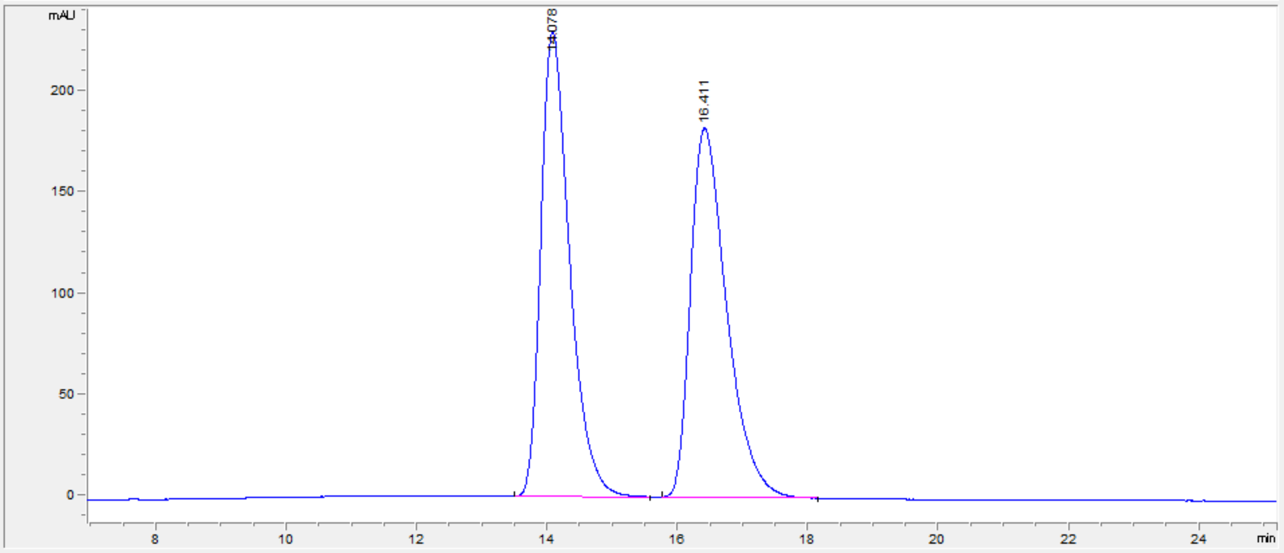

#	Time	Area	Height	Width	Area%	Symmetry
1	14.078	7074.1	229.6	0.4695	50.032	0.64
2	16.411	7065	182.6	0.5875	49.968	0.594

Enantioenriched (CHIRALPAK® AS-H, hexane/IPA = 80/20, 1 mL/min)

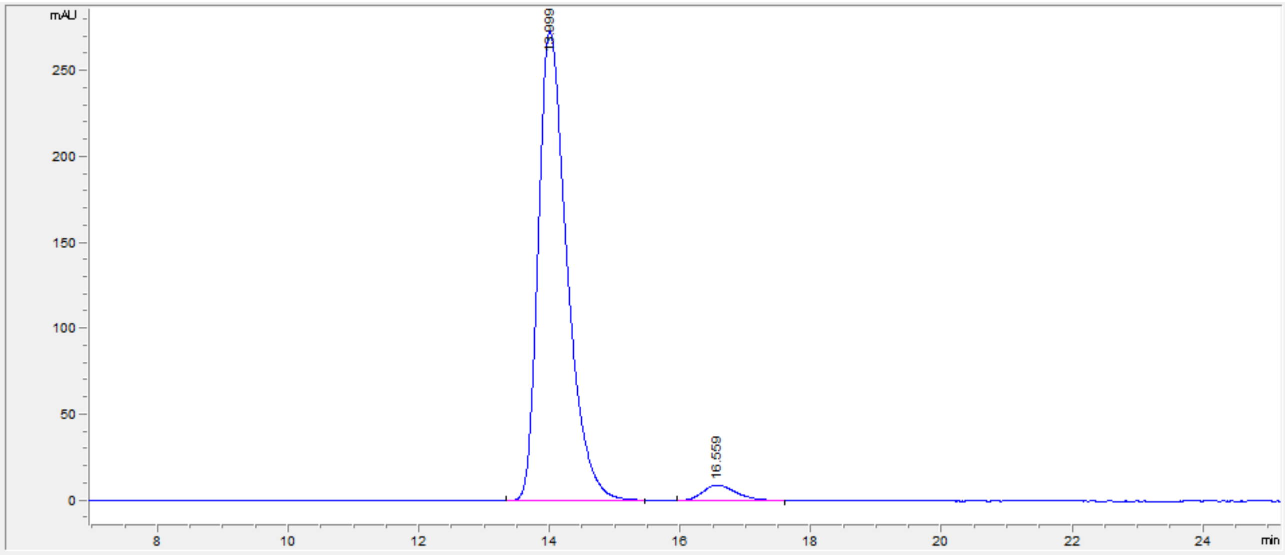

#	Time	Area	Height	Width	Area%	Symmetry
1	13.999	8352.2	272.9	0.465	96.246	0.639
2	16.559	325.8	9	0.5321	3.754	0.708

3y

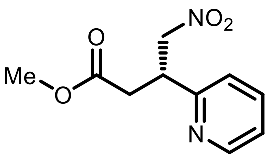

Racemic (CHIRALPAK® OD, hexane/IPA = 85/15, 1 mL/min)

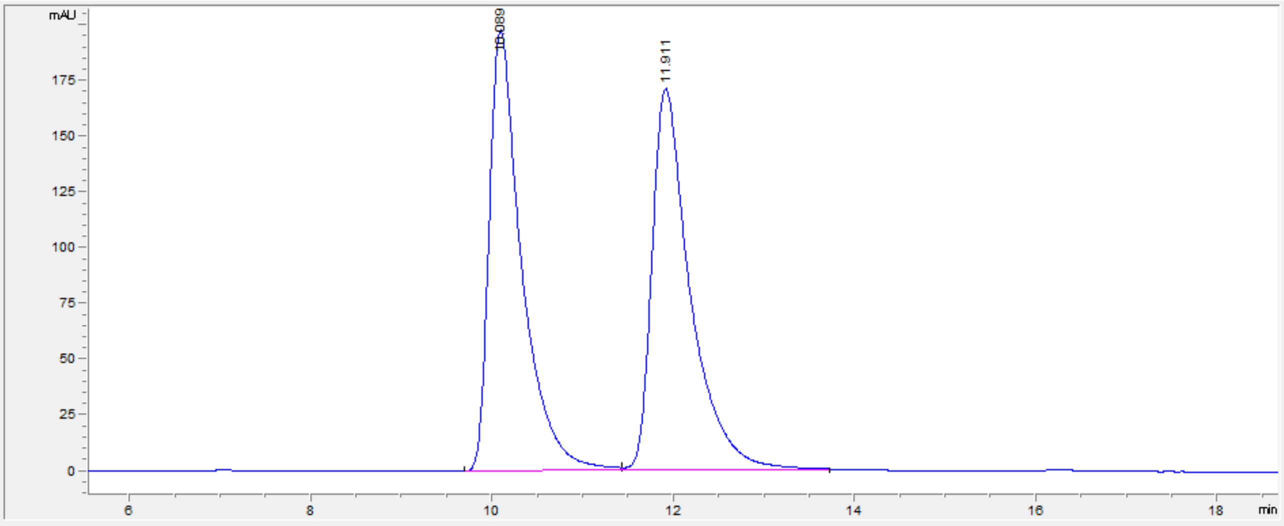

#	Time	Area	Height	Width	Area%	Symmetry
1	10.089	4953.5	197.9	0.366	49.564	0.489
2	11.911	5040.6	171.3	0.4313	50.436	0.508

Enantioenriched (CHIRALPAK® OD, hexane/IPA = 85/15, 1 mL/min)

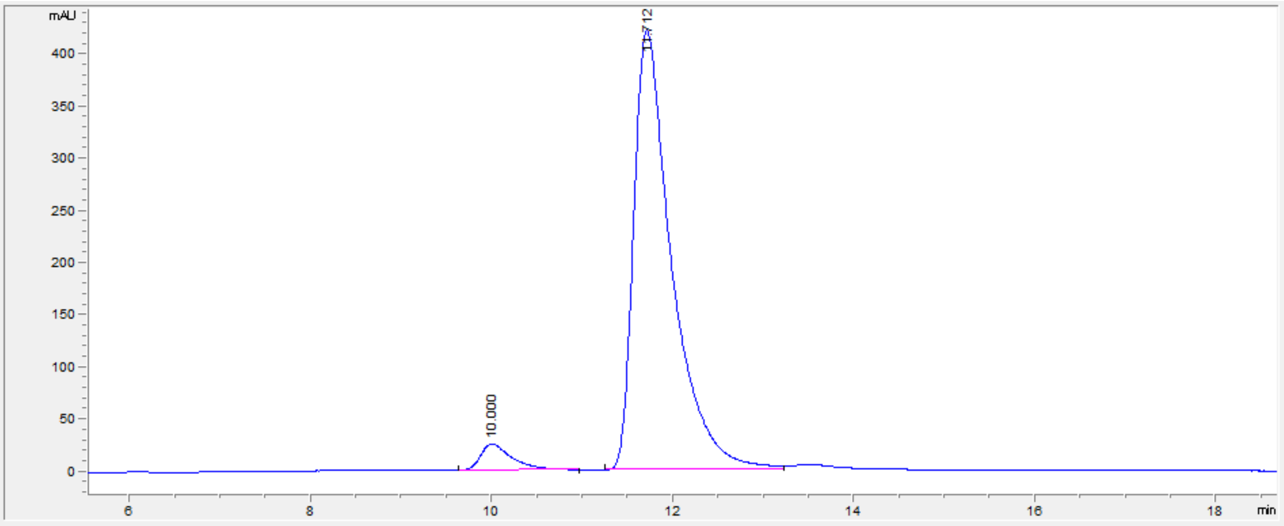

#	Time	Area	Height	Width	Area%	Symmetry
1	10	617.9	25.4	0.3543	4.773	0.512
2	11.712	12326.9	421.4	0.4312	95.227	0.488

[ToC]

3z

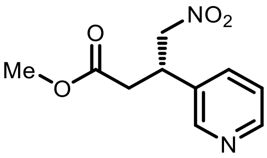

Racemic (CHIRALPAK® AD-H, hexane/IPA = 80/20, 1 mL/min)

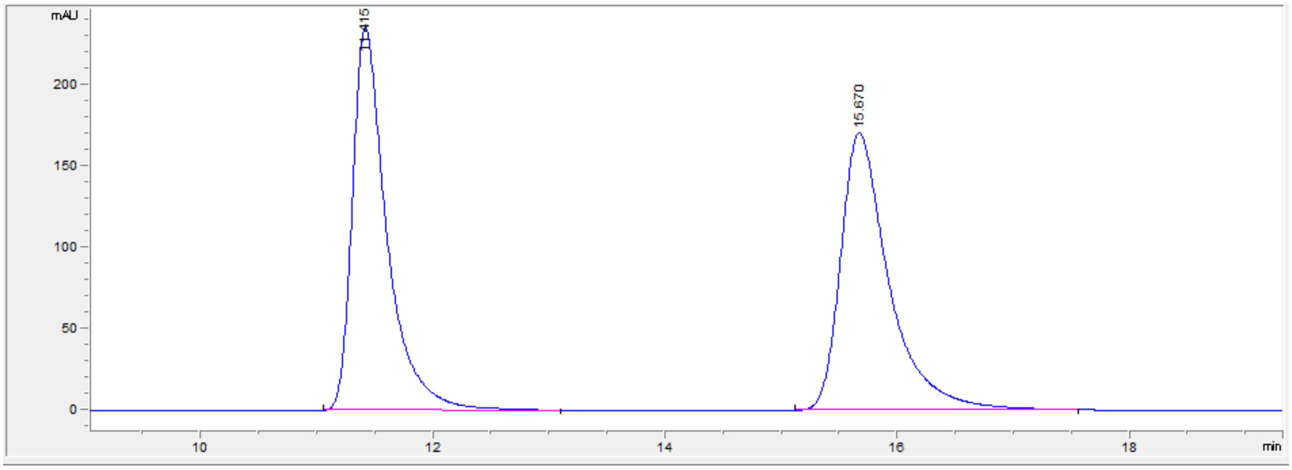

#	Time	Area	Height	Width	Area%	Symmetry
1	11.415	4874.9	235.7	0.3073	49.492	0.549
2	15.67	4974.9	170.8	0.4319	50.508	0.57

Enantioenriched (CHIRALPAK® AD-H, hexane/IPA = 80/20, 1 mL/min)

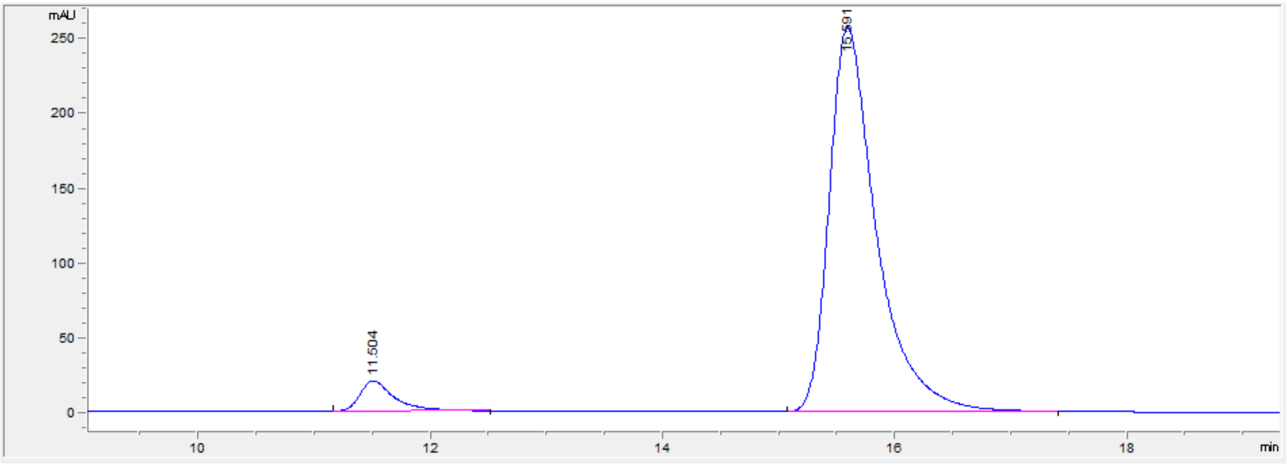

#	Time	Area	Height	Width	Area%	Symmetry
1	11.504	428.8	20.3	0.309	5.572	0.551
2	15.591	7267.6	256.3	0.4188	94.428	0.583

[ToC]

3aa

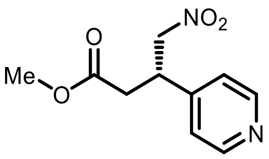

Racemic (CHIRALPAK® AD-H, hexane/IPA = 80/20, 1 mL/min)

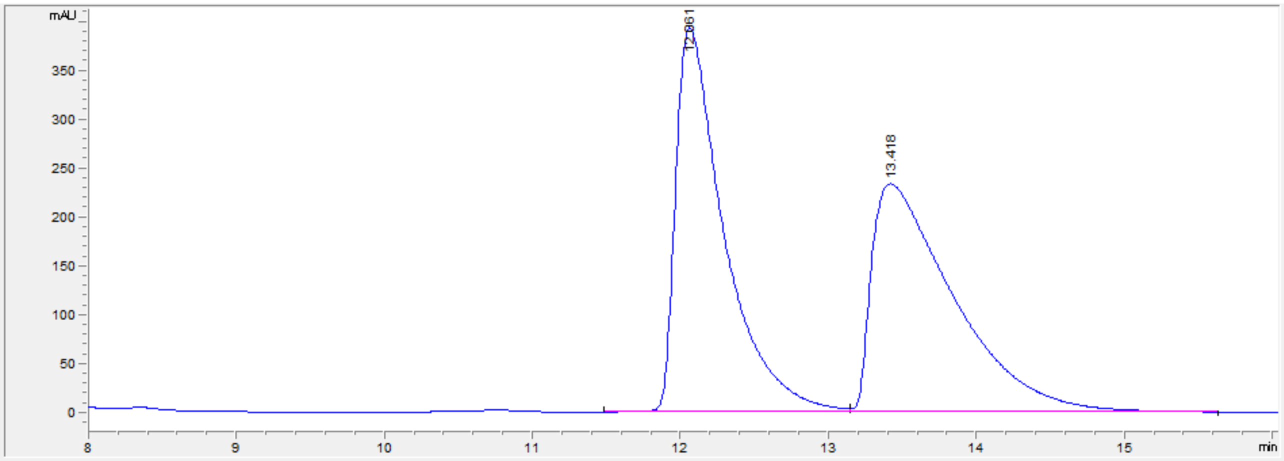

#	Time	Area	Height	Width	Area%	Symmetry
1	12.061	8963.9	393.7	0.3336	49.808	0.38
2	13.418	9033.1	233.8	0.5708	50.192	0.274

Enantioenriched (CHIRALPAK® AD-H, hexane/IPA = 80/20, 1 mL/min)

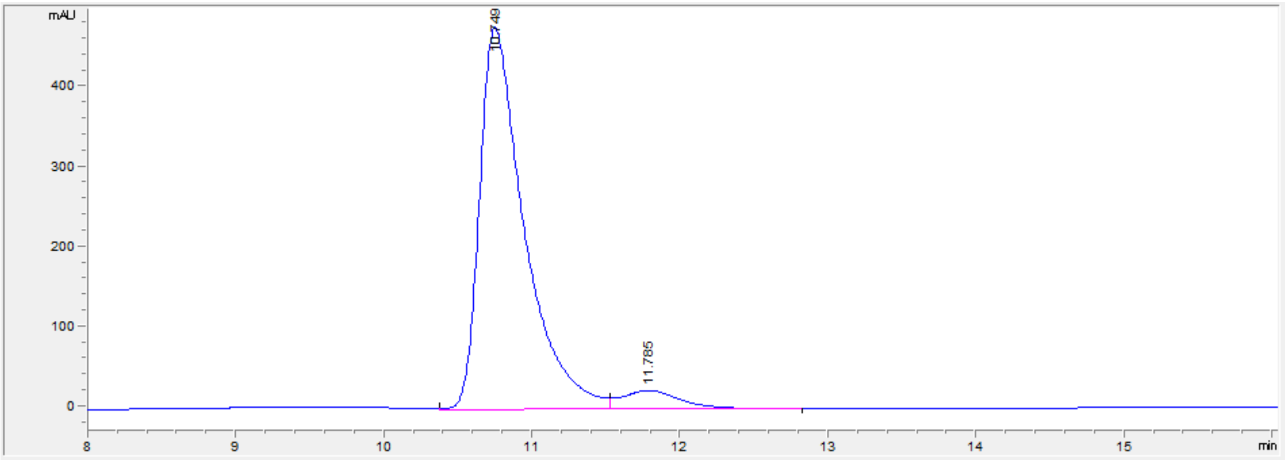

#	Time	Area	Height	Width	Area%	Symmetry
1	10.749	10083.8	478.6	0.3098	93.715	0.485
2	11.785	676.2	24	0.4066	6.285	0.713

[ToC]

3ab

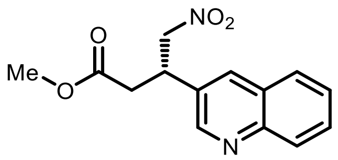

Racemic (CHIRALPAK® IB, hexane/IPA = 70/30, 1 mL/min)

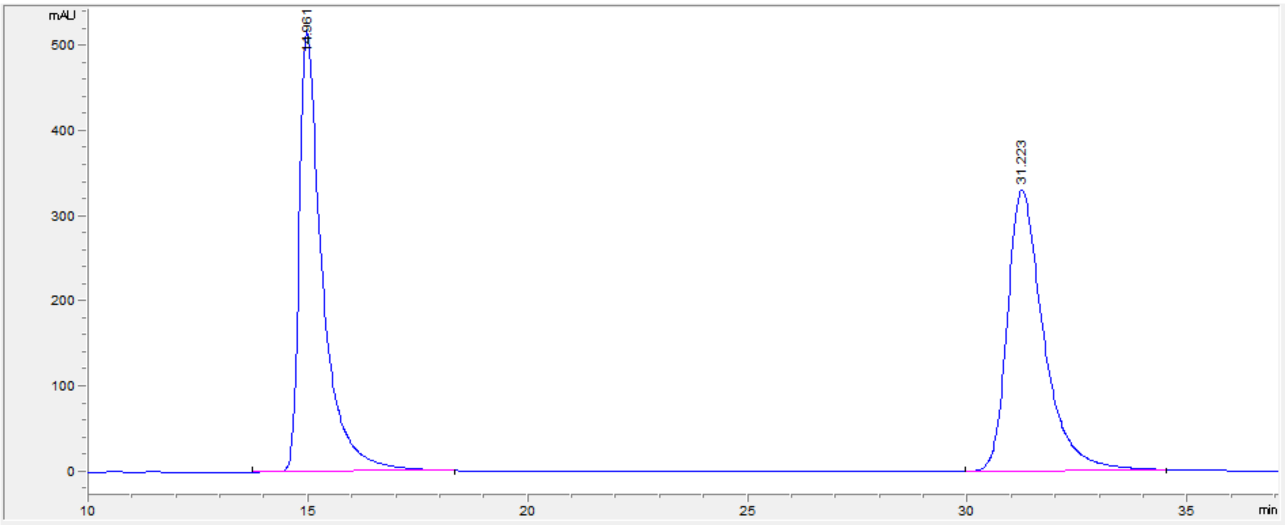

#	Time	Area	Height	Width	Area%	Symmetry
1	14.961	19043.5	518.7	0.5323	50.091	0.44
2	31.223	18974.6	330.3	0.8602	49.909	0.616

Enantioenriched (CHIRALPAK® IB, hexane/IPA = 70/30, 1 mL/min)

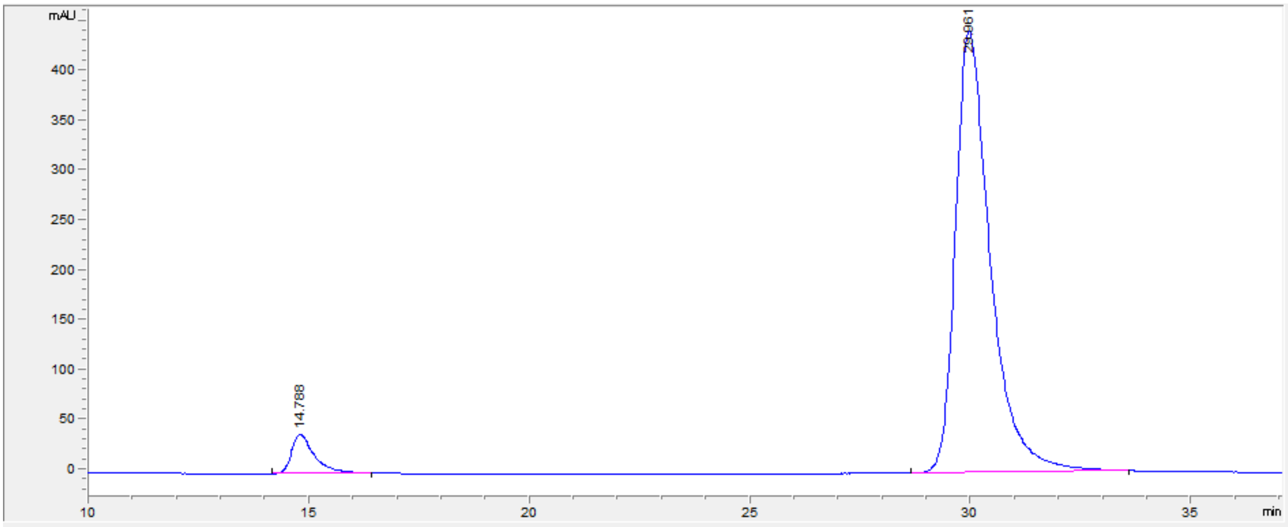

#	Time	Area	Height	Width	Area%	Symmetry
1	14.788	1434.1	39.5	0.5279	5.549	0.536
2	29.961	24410.4	443.7	0.8218	94.451	0.597

[ToC]

3ac

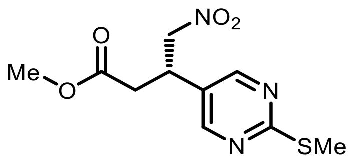

Racemic (CHIRALPAK® OD-H, hexane/IPA = 80/20, 1 mL/min)

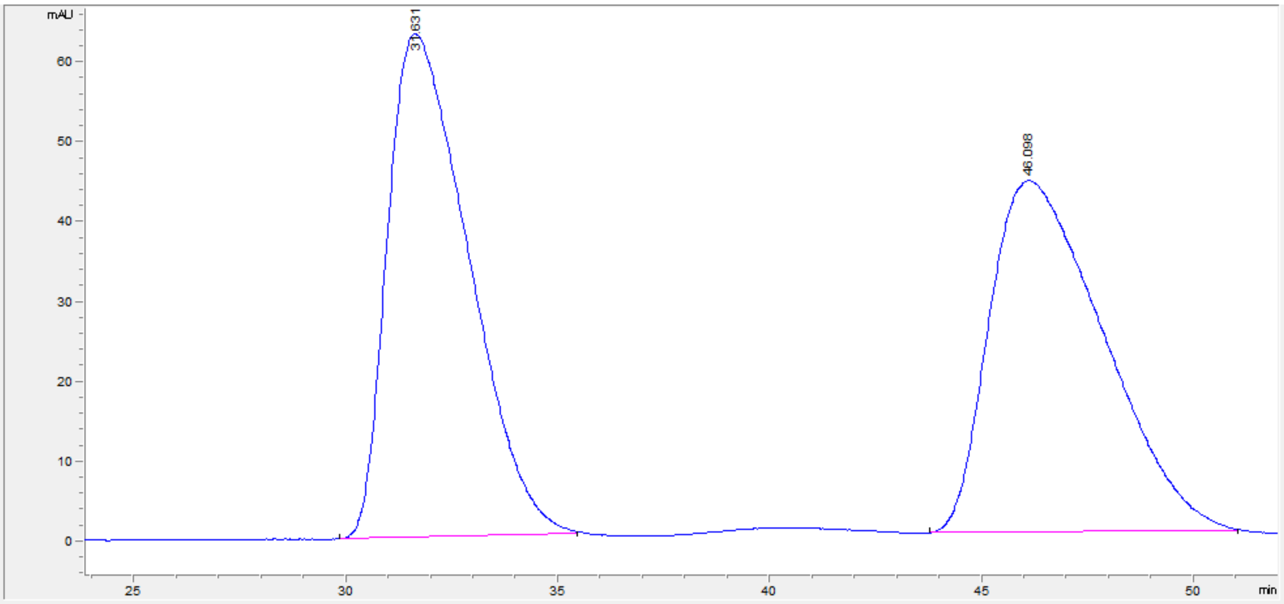

#	Time	Area	Height	Width	Area%	Symmetry
1	31.631	8382.7	63.1	1.5727	50.352	0.516
2	46.098	8265.5	44.1	2.2287	49.648	0.513

Enantioenriched (CHIRALPAK® OD-H, hexane/IPA = 80/20, 1 mL/min)

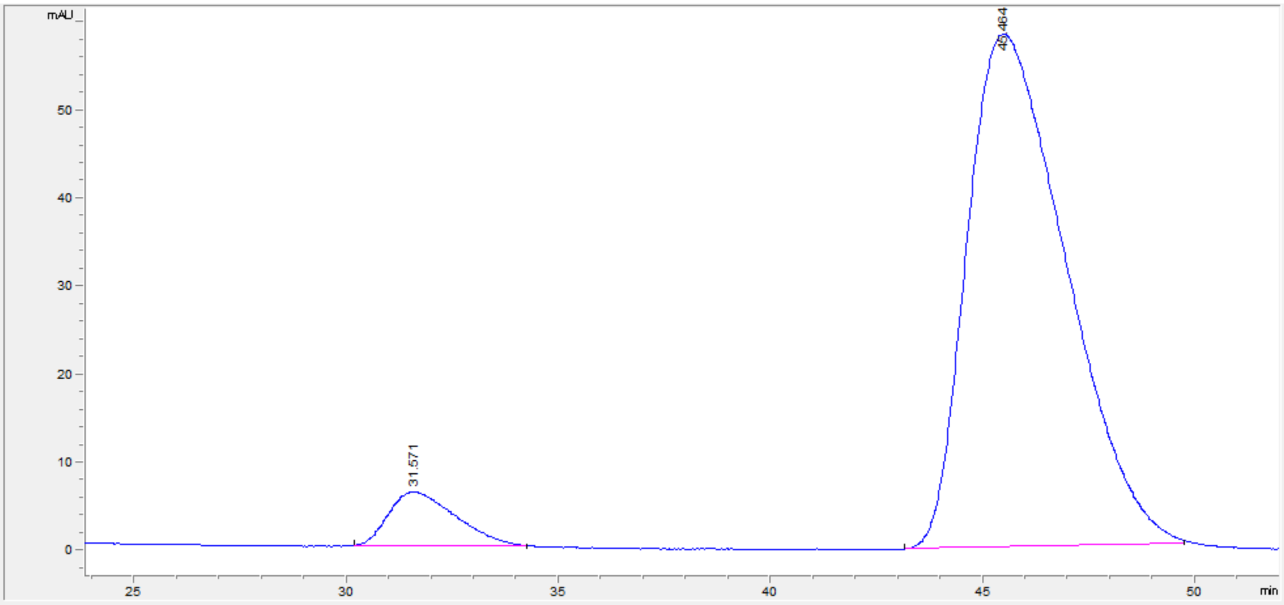

#	Time	Area	Height	Width	Area%	Symmetry
1	31.571	677.7	6.2	1.2953	6.704	0.576
2	45.464	9430.3	58.3	1.902	93.296	0.554

[ToC]

3ad

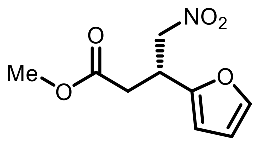

Racemic (CHIRALPAK® AS-H, hexane/IPA = 80/20, 1 mL/min)

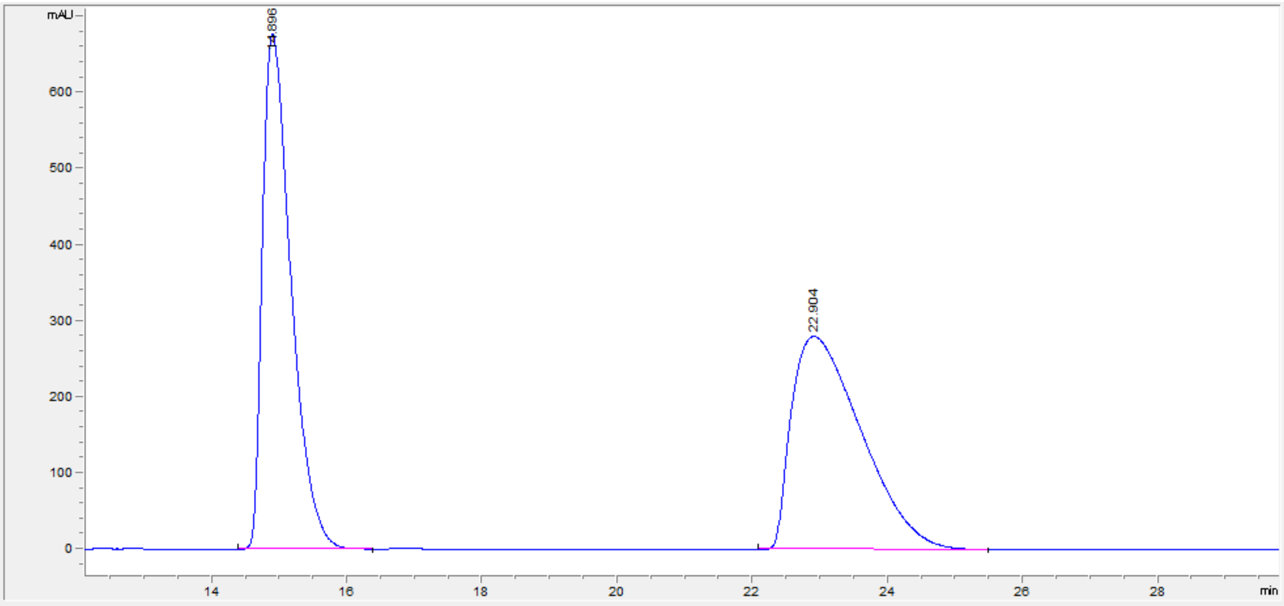

#	Time	Area	Height	Width	Area%	Symmetry
1	14.896	19944.3	677.9	0.4533	49.770	0.503
2	22.904	20128.3	280.4	1.1214	50.230	0.442

Enantioenriched (CHIRALPAK® AS-H, hexane/IPA = 80/20, 1 mL/min)

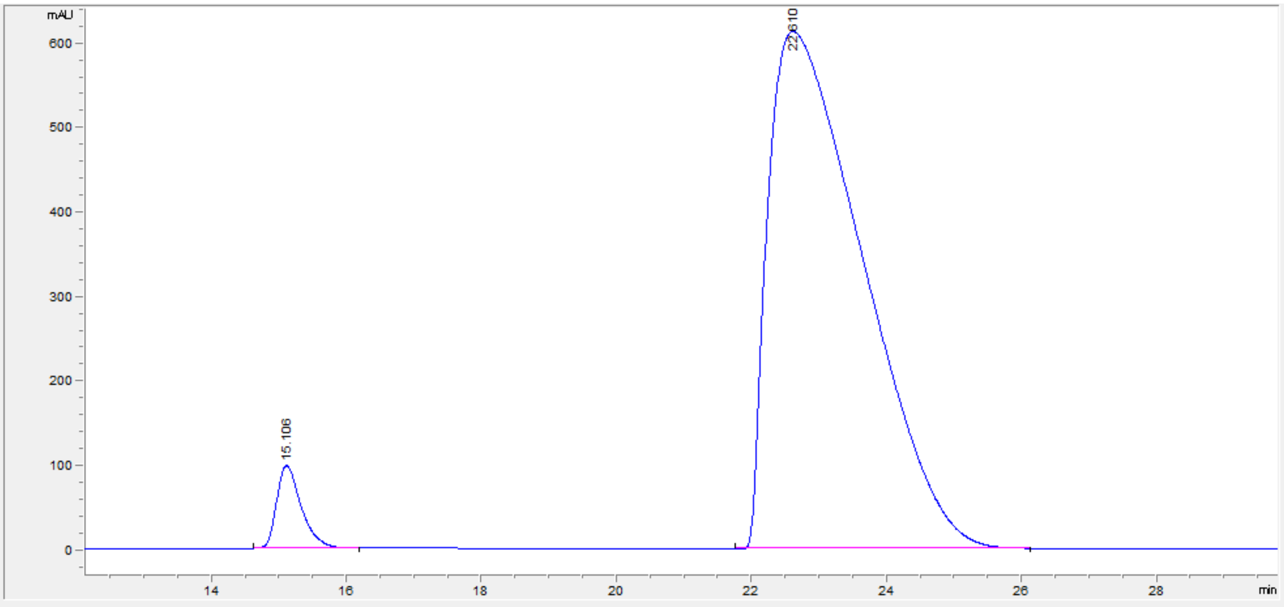

#	Time	Area	Height	Width	Area%	Symmetry
1	15.106	2481	97.8	0.3839	3.993	0.619
2	22.61	59647.1	612.4	1.5242	96.007	0.345

[ToC]

3ae

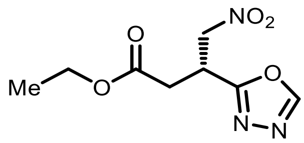

Racemic (CHIRALPAK® IA, hexane/IPA = 90/10, 1 mL/min)

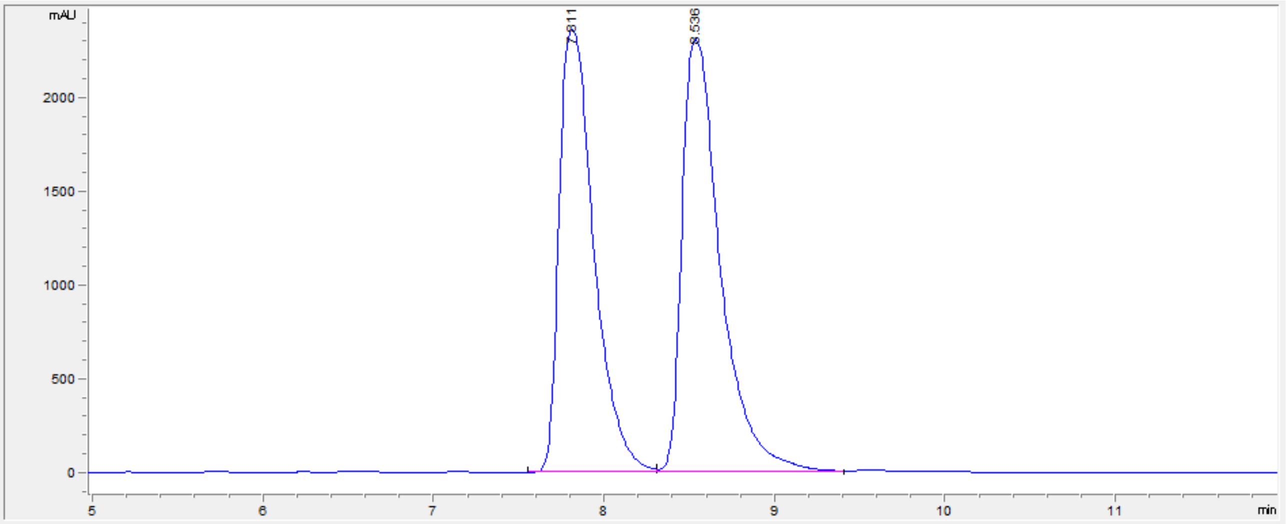

#	Time	Area	Height	Width	Area%	Symmetry
1	7.811	33538.6	2358.4	0.2179	47.967	0.555
2	8.536	36381	2308	0.2379	52.033	0.505

Enantioenriched (CHIRALPAK® IA, hexane/IPA = 90/10, 1 mL/min)

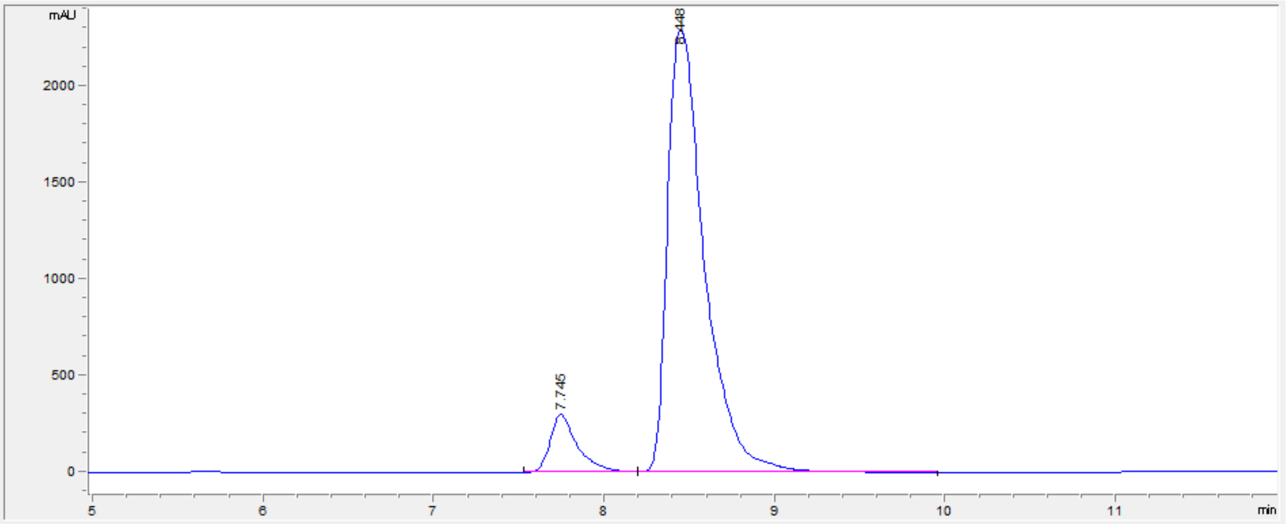

#	Time	Area	Height	Width	Area%	Symmetry
1	7.745	3363.7	300.1	0.1648	8.864	0.596
2	8.448	34582	2293.5	0.2279	91.136	0.512

[ToC]

3af

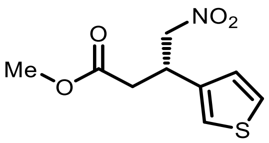

Racemic (CHIRALPAK® OD, hexane/IPA = 85/15, 1 mL/min)

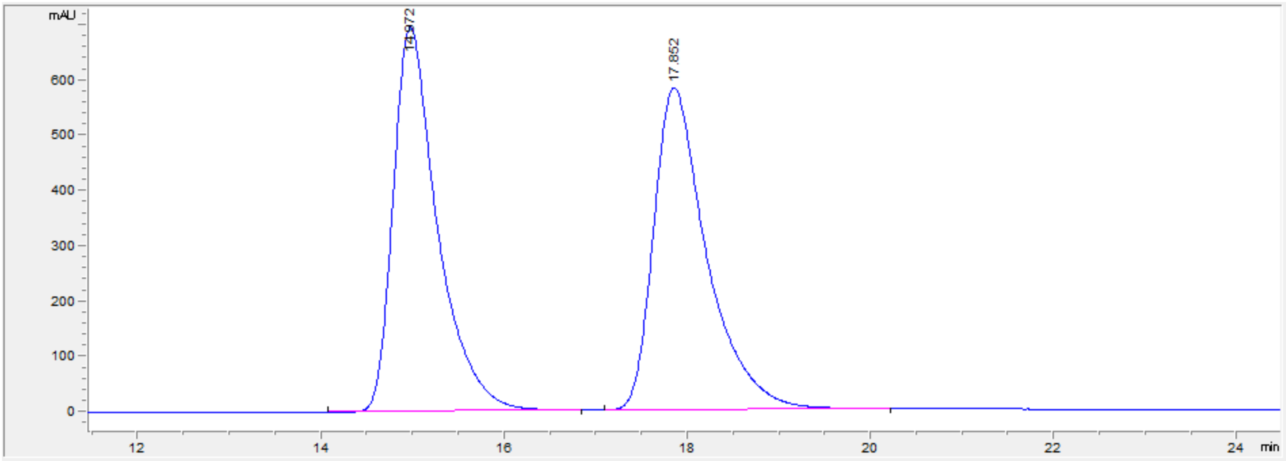

#	Time	Area	Height	Width	Area%	Symmetry
1	14.972	23419.5	695.3	0.499	49.773	0.582
2	17.852	23633	583.7	0.6003	50.227	0.58

Enantioenriched (CHIRALPAK® OD, hexane/IPA = 85/15, 1 mL/min)

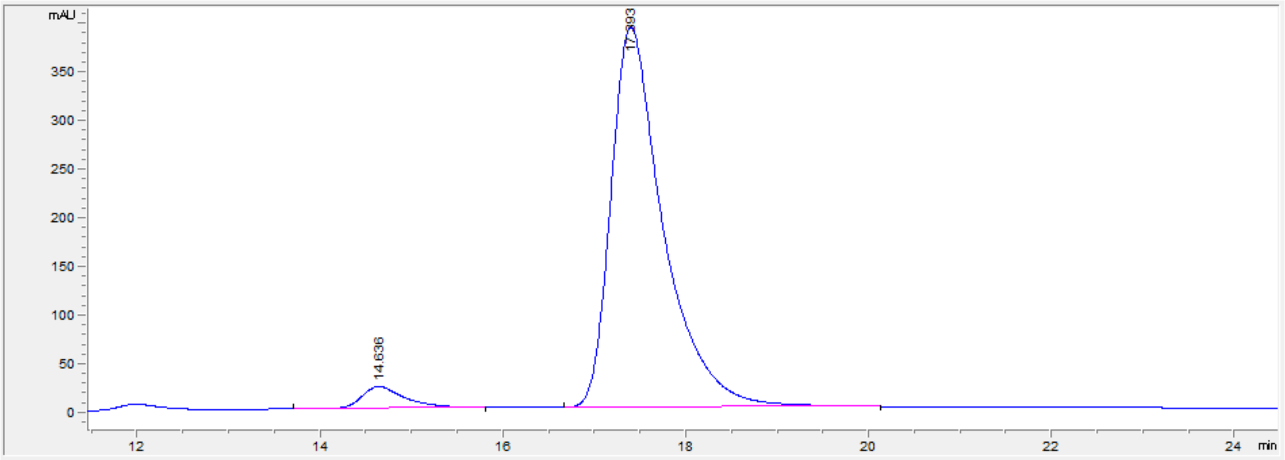

#	Time	Area	Height	Width	Area%	Symmetry
1	14.636	746.6	22.4	0.4927	4.557	0.591
2	17.393	15637.4	390.5	0.5912	95.443	0.561

[ToC]

3ag

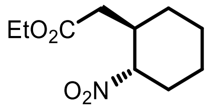

Racemic (SFC CHIRALPAK® IF, 0% MeOH for 3 minutes; then 0% to 10% MeOH over 5 min, then from 10% to 30% MeOH in 0.5 min, then from 30% to 50% MeOH in 0.5 min, then hold 50% MeOH for 2 mins, 1.5 mL/min)

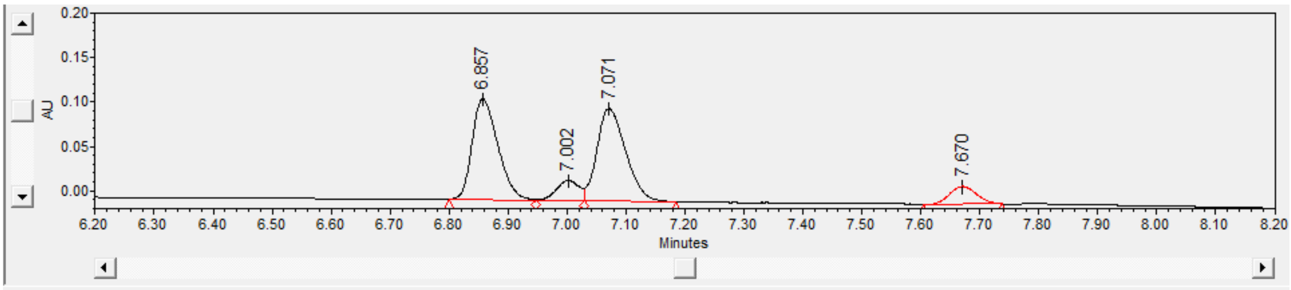

	Name	Retention Time (min)	Purity1 Angle	Purity1 Threshold	PDA/FLR Match1 Spect. Name	PDA/FLR Match1 Angle	PDA/FLR Match1 Threshold	PDA/FLR Match1 Lib. Name	Area (µV*sec)	% Area	Height (µV)	Int Type	Amount	Units	Peak Type	P
1		6.857							341043	41.96	113170	bv			Unknown	
2		7.002							59361	7.30	22342	vv			Unknown	
3		7.071							350098	43.07	104210	vb			Unknown	
4		7.670							62308	7.67	19759	bb			Unknown	

Enantioenriched (SFC CHIRALPAK® IF, 0% MeOH for 3 minutes; then 0% to 10% MeOH over 5 min, then from 10% to 30% MeOH in 0.5 min, then from 30% to 50% MeOH in 0.5 min, then hold 50% MeOH for 2 mins, 1.5 mL/min)

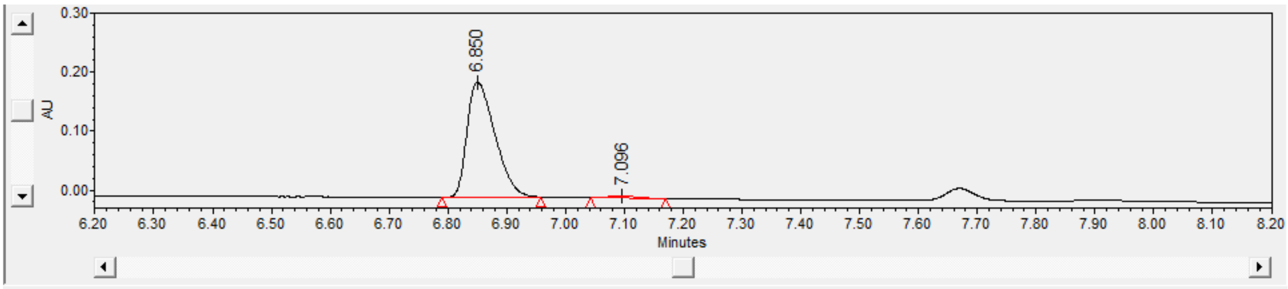

	Name	Retention Time (min)	Purity1 Angle	Purity1 Threshold	PDA/FLR Match1 Spect. Name	PDA/FLR Match1 Angle	PDA/FLR Match1 Threshold	PDA/FLR Match1 Lib. Name	Area (µV*sec)	% Area	Height (µV)	Int Type	Amount	Units	Peak Type	P
1		6.850							642991	98.07	195358	bb			Unknown	
2		7.096							12651	1.93	4226	bb			Unknown	

γ-Nitroester Derivatives

4a

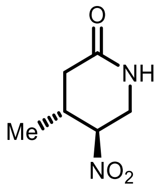

Racemic (CHIRALPAK® AD, hexane/IPA = 85/15, 1 mL/min)

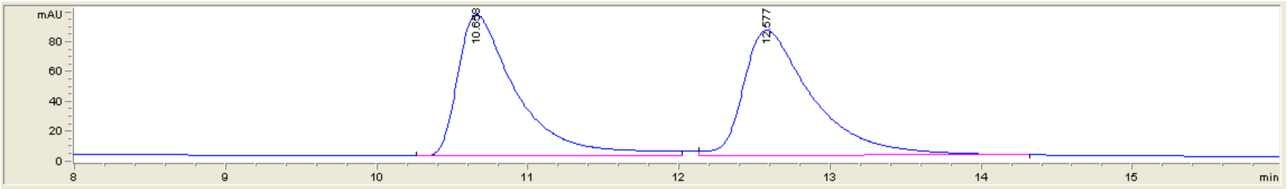

#	Time	Area	Height	Width	Area%	Symmetry
1	10.658	2596.5	94.8	0.3995	48.985	0.424
2	12.577	2704.1	84.2	0.4667	51.015	0.464

Enantioenriched (CHIRALPAK® AD, hexane/IPA = 85/15, 1 mL/min)

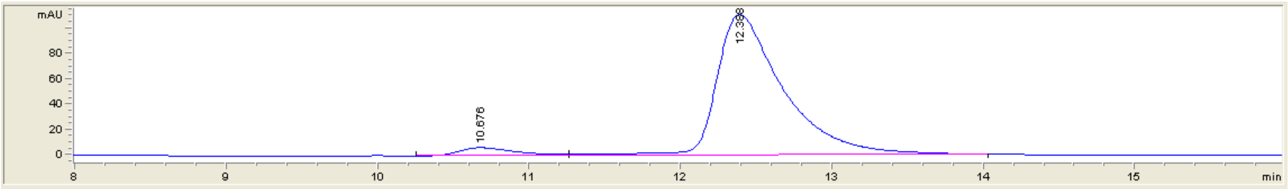

#	Time	Area	Height	Width	Area%	Symmetry
1	10.676	165.8	6.3	0.3907	4.600	0.56
2	12.388	3439.4	110.9	0.4538	95.400	0.495

4b

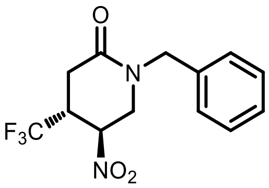

Racemic (CHIRALPAK® AD-H, hexane/IPA = 90/10, 1 mL/min)

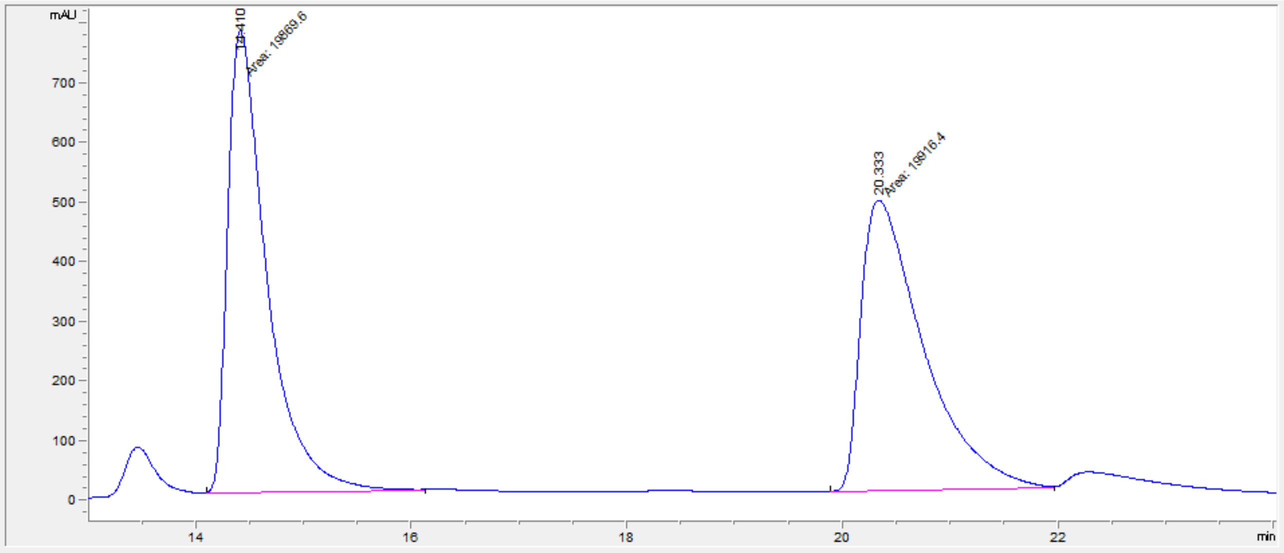

#	Time	Area	Height	Width	Area%	Symmetry
1	14.41	19869.6	775.7	0.4269	49.941	0.452
2	20.333	19916.4	488.8	0.6791	50.059	0.384

Enantioenriched (CHIRALPAK® AD, hexane/IPA = 90/10, 1 mL/min)

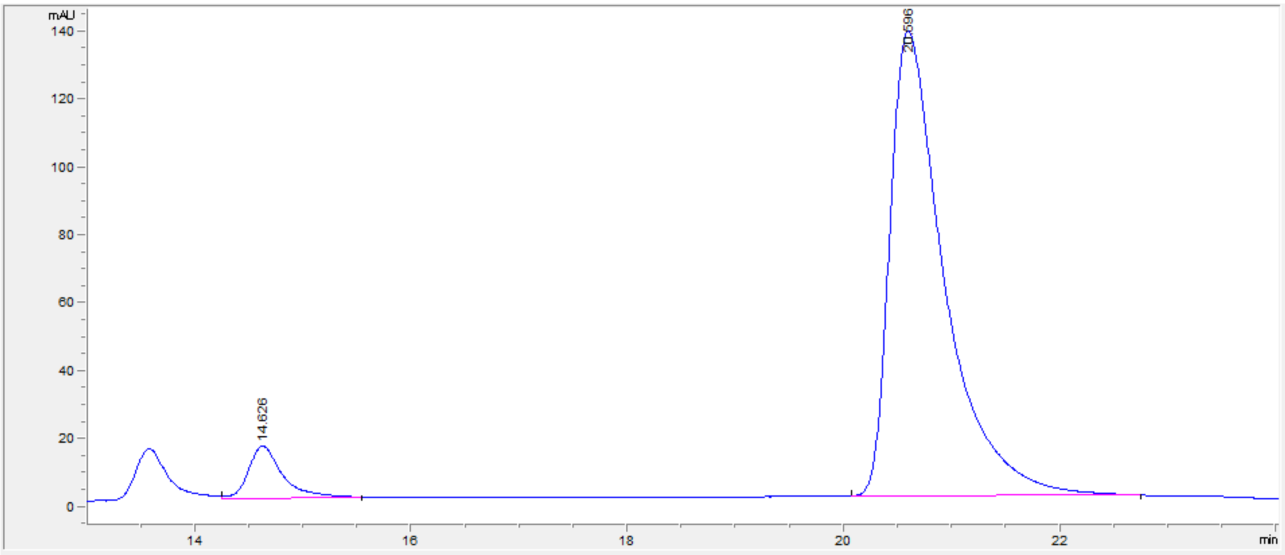

#	Time	Area	Height	Width	Area%	Symmetry
1	14.626	350.9	15.5	0.3359	6.796	0.651
2	20.596	4812.6	136.7	0.5227	93.204	0.458

[ToC]

4c

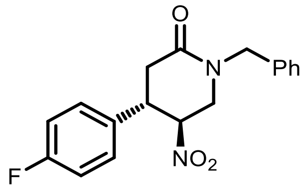

Racemic (CHIRALPAK® AD-H, hexane/IPA = 85/15, 1 mL/min)

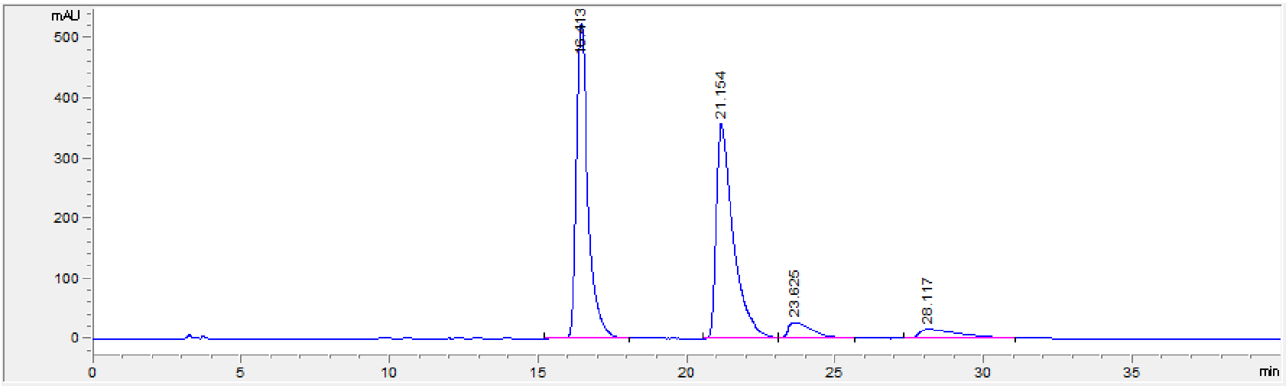

#	Time	Area	Height	Width	Area%	Symmetry
1	16.413	14601.3	523.2	0.4216	45.609	0.596
2	21.154	14589.8	356.6	0.6074	45.573	0.439
3	23.625	1451.7	26.2	0.8151	4.535	0.379
4	28.117	1371.1	14.7	1.2507	4.283	0.28

Enantioenriched (CHIRALPAK® AD-H, hexane/IPA = 85/15, 1 mL/min)

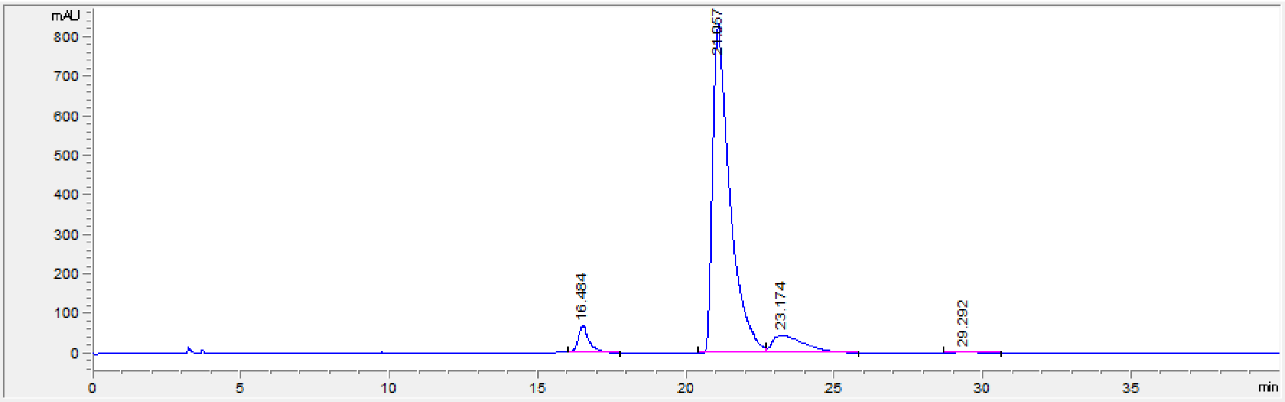

#	Time	Area	Height	Width	Area%	Symmetry
1	16.484	1891.1	67.6	0.4122	4.729	0.628
2	21.057	34632.3	831.4	0.62	86.606	0.435
3	23.174	3334.7	44.1	1.0847	8.339	0.346
4	29.292	130.4	2.5	0.6366	0.326	0.549

[ToC]

5a

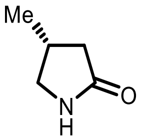

Racemic (CHIRALPAK® IG, from 1% to 20% MeOH in 7 min, then from 20% to 50% in 1 min, 1 mL/min)

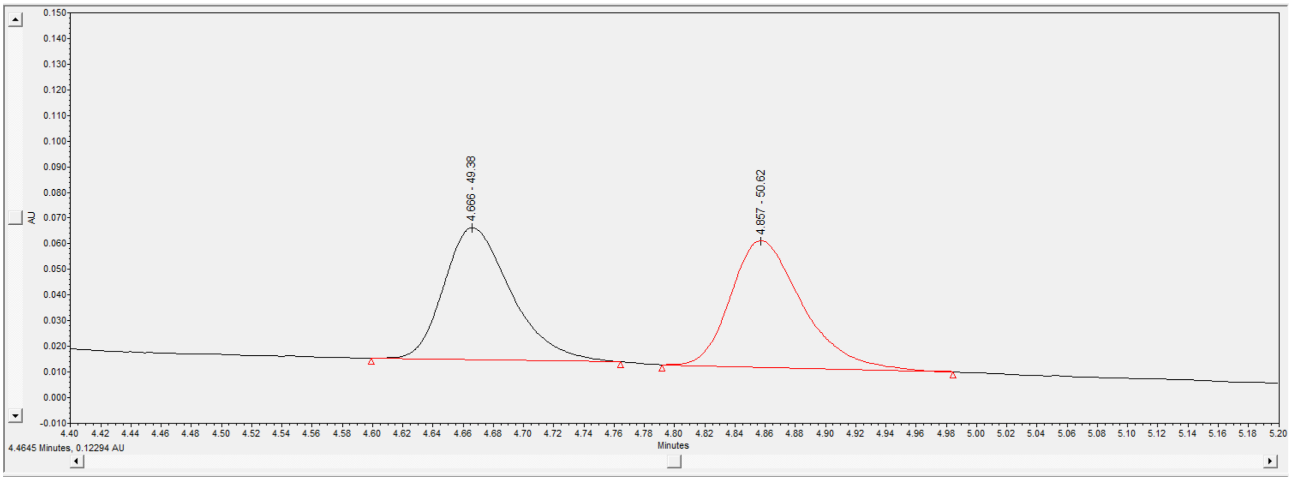

	Name	Retention Time (min)	Purity1 Angle	Purity1 Threshold	PDA/FLR Match1 Spect. Name	PDA/FLR Match1 Angle	PDA/FLR Match1 Threshold	PDA/FLR Match1 Lib. Name	Area (μV*sec)	% Area	Height (μV)	Int Type	Amount	Units	Peak Type	Peak Codes
1		4.666							158047	49.38	51386	bb			Unknown	
2		4.857							162043	50.62	49279	bb			Unknown	

Enantioenriched (CHIRALPAK® IG, from 1% to 20% MeOH in 7 min, then from 20% to 50% in 1 min, 1 mL/min)

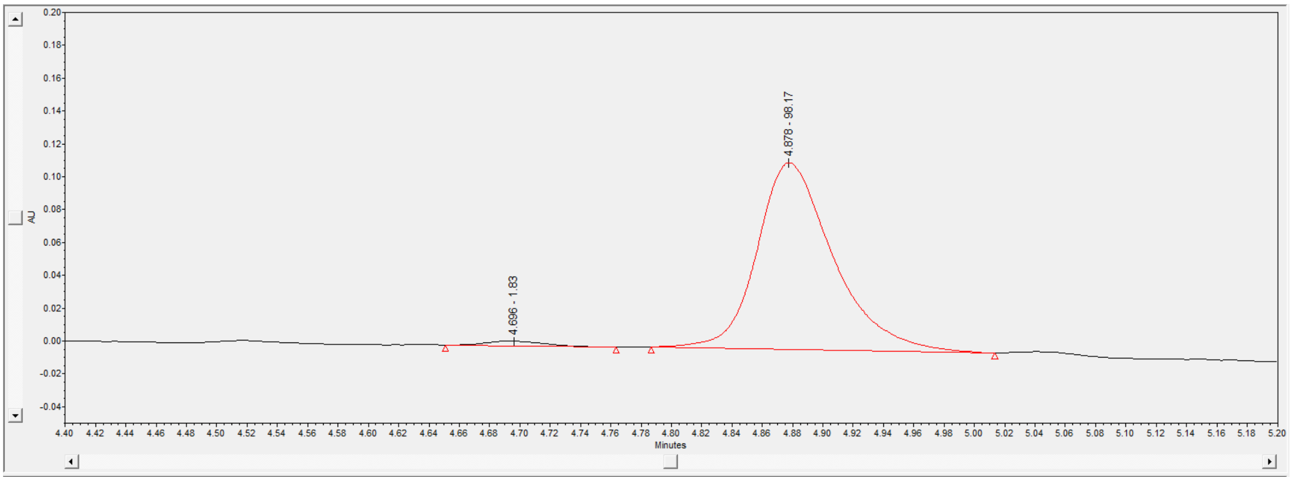

	Name	Retention Time (min)	Purity1 Angle	Purity1 Threshold	PDA/FLR Match1 Spect. Name	PDA/FLR Match1 Angle	PDA/FLR Match1 Threshold	PDA/FLR Match1 Lib. Name	Area (μV*sec)	% Area	Height (μV)	Int Type	Amount	Units	Peak Type	Peak Codes
1		4.696							7456	1.83	2804	bb			Unknown	I08
2		4.878							399139	98.17	113634	bb			Unknown	

[ToC]

5b

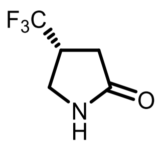

Racemic (CHIRALPAK® AD-H, hexane/IPA = 90/10, 1 mL/min)

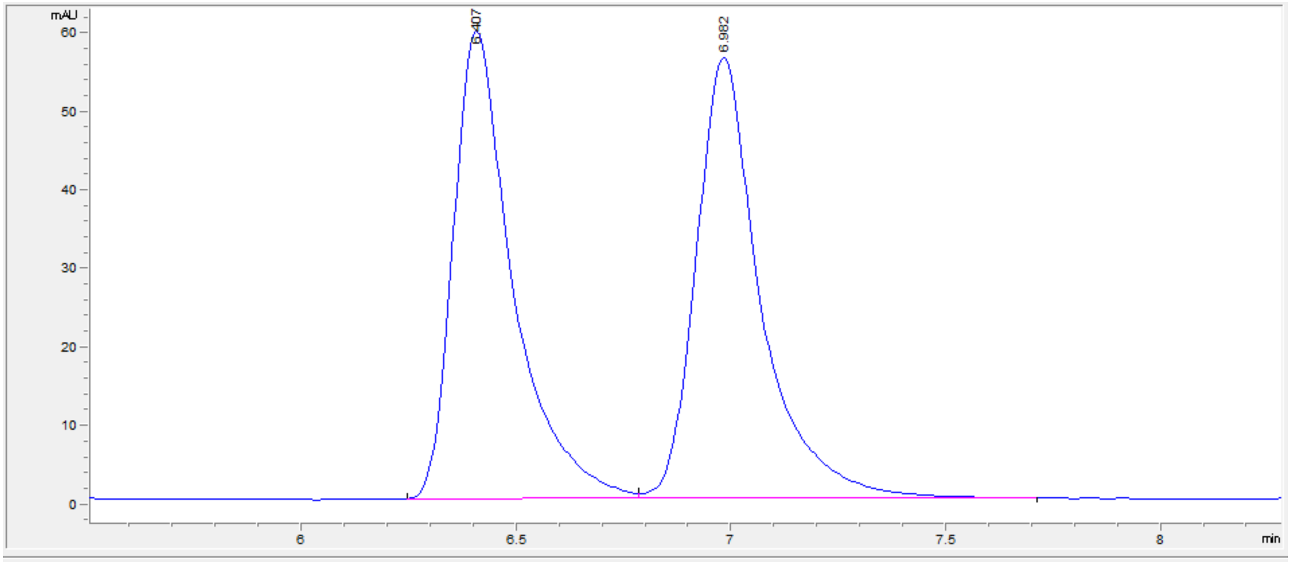

#	Time	Area	Height	Width	Area%	Symmetry
1	6.407	576.3	59.6	0.1449	49.262	0.586
2	6.982	593.6	56.2	0.1552	50.738	0.676

Enantioenriched (CHIRALPAK® AD-H, hexane/IPA = 90/10, 1 mL/min)

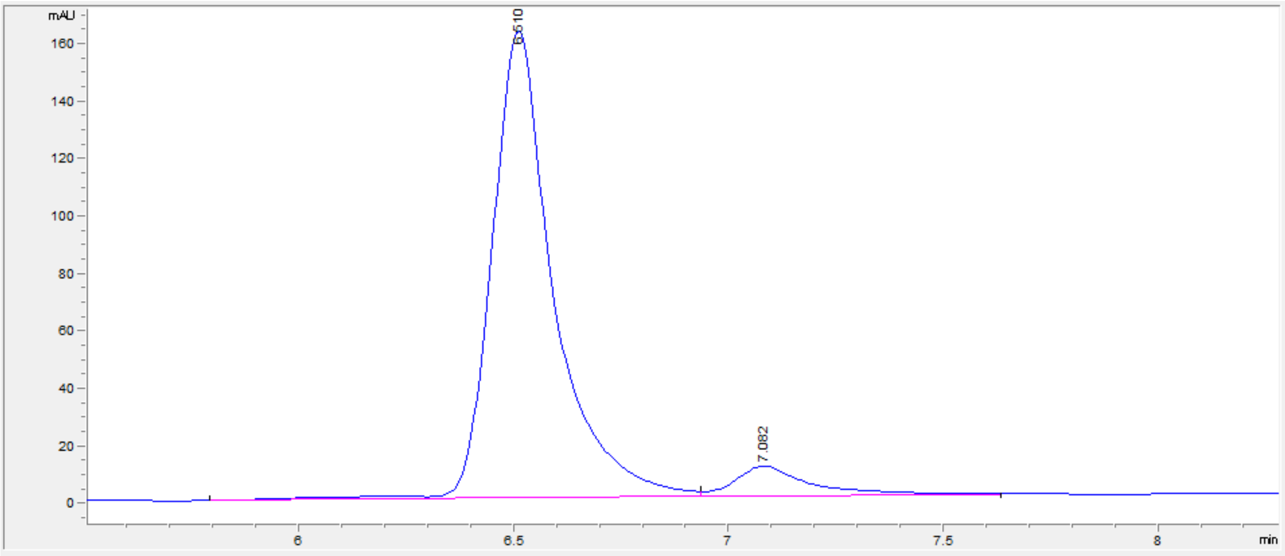

#	Time	Area	Height	Width	Area%	Symmetry
1	6.51	1629.2	162.6	0.1469	92.273	0.706
2	7.082	136.4	10.5	0.1857	7.727	0.52

[ToC]

5c

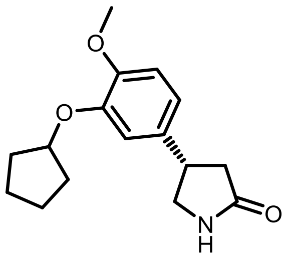

Racemic (CHIRALPAK® IG, from 1% to 20% MeOH in 7 min, then from 20% to 50% in 1 min, 1 mL/min)

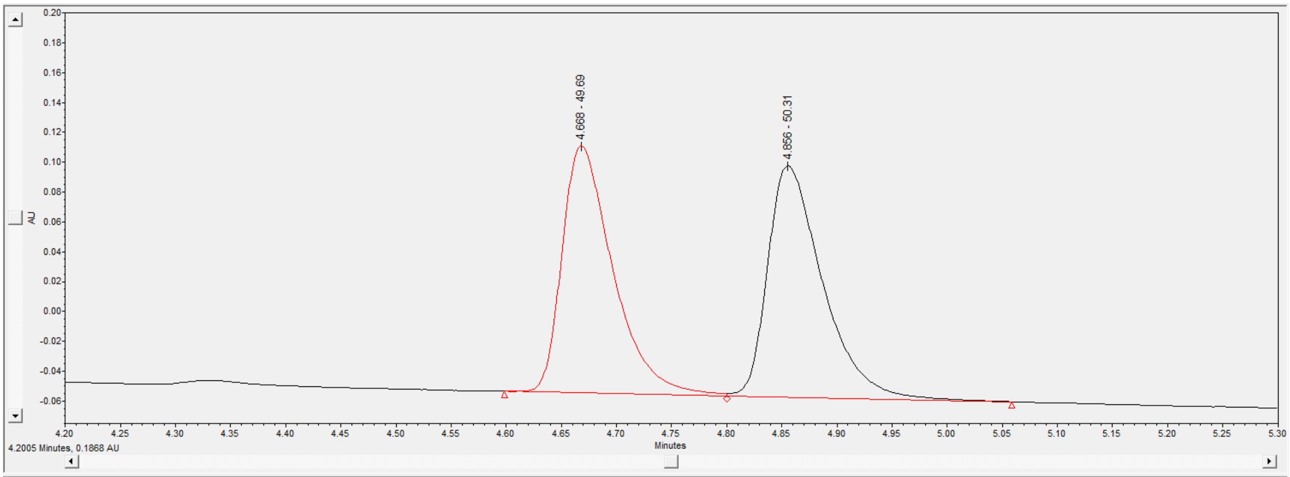

	Name	Retention Time (min)	Purity1 Angle	Purity1 Threshold	PDA/FLR Match1 Spect. Name	PDA/FLR Match1 Angle	PDA/FLR Match1 Threshold	PDA/FLR Match1 Lib. Name	Area (µV*sec)	% Area	Height (µV)	Int Type	Amount	Units	Peak Type	Peak Codes
1		4.668							531468	49.69	165302	bv			Unknown	
2		4.856							538043	50.31	155094	vb			Unknown	

Enantioenriched (CHIRALPAK® IG, from 1% to 20% MeOH in 7 min, then from 20% to 50% in 1 min, 1 mL/min)

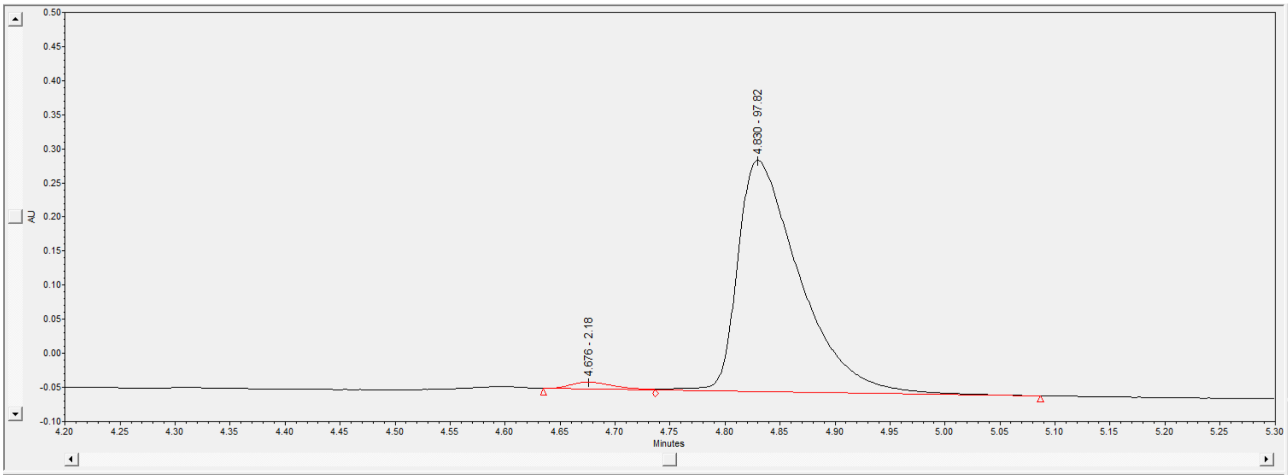

	Name	Retention Time (min)	Purity1 Angle	Purity1 Threshold	PDA/FLR Match1 Spect. Name	PDA/FLR Match1 Angle	PDA/FLR Match1 Threshold	PDA/FLR Match1 Lib. Name	Area (µV*sec)	% Area	Height (µV)	Int Type	Amount	Units	Peak Type	Peak Codes
1		4.676							29644	2.18	10362	bv			Unknown	
2		4.830							1331512	97.82	339254	vb			Unknown	

[ToC]

5d

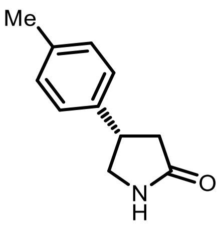

Racemic (CHIRALPAK® AD-H, hexane/IPA = 97/3, 1 mL/min)

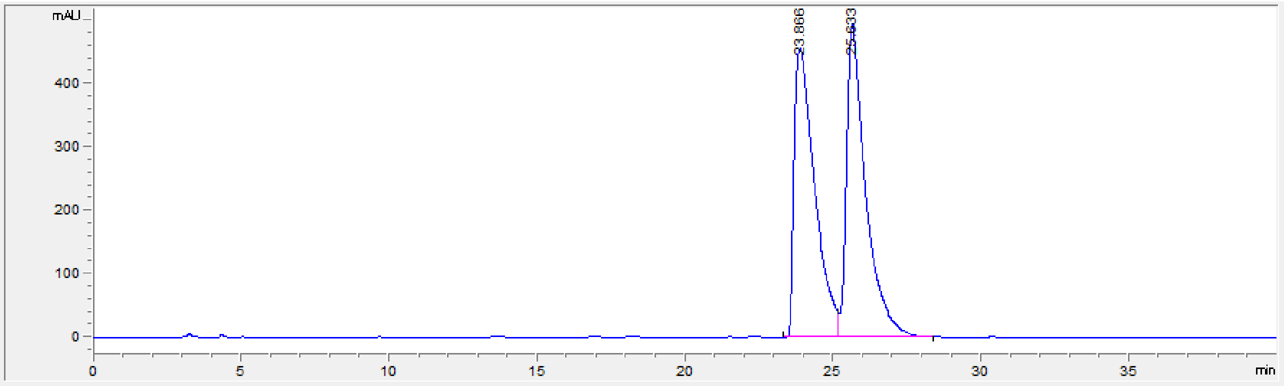

#	Time	Area	Height	Width	Area%	Symmetry
1	23.866	21516.9	454.6	0.7062	48.616	0.34
2	25.633	22742.3	494.4	0.6766	51.384	0.408

Enantioenriched (CHIRALPAK® AD-H, hexane/IPA = 97/3, 1 mL/min)

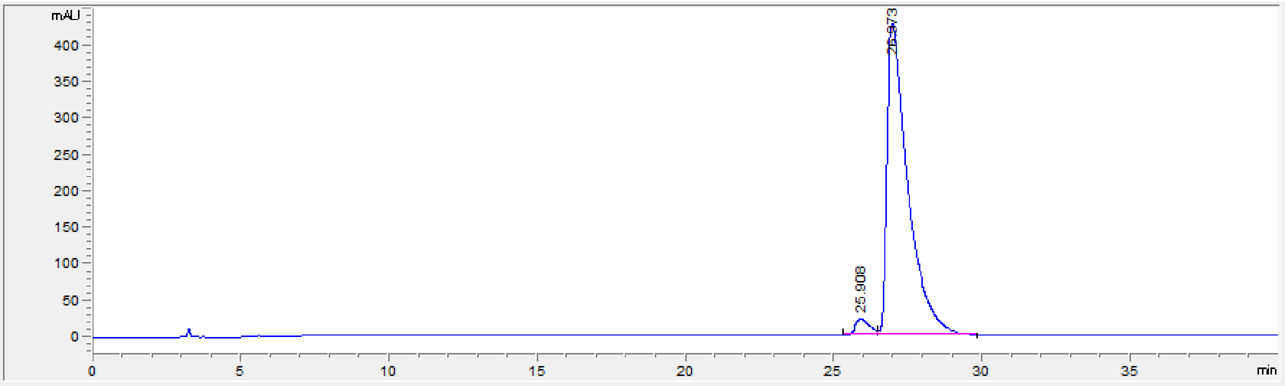

#	Time	Area	Height	Width	Area%	Symmetry
1	25.908	741	22.3	0.5153	3.376	0.61
2	26.973	21206	426.7	0.7297	96.624	0.358

[ToC]

COMPUTATIONAL STUDIES

Computational Methods

All calculations reported in this paper were performed using the Gaussian 16 (G16) program.[24] Equilibrium structures and transition state geometries were optimized using the B3LYP functional and the 6-31G(d) basis set.[25] Dispersion interactions were included using Grimme's DFT-D3 correction.[26] SMD solvent effects were incorporated into all calculations with cyclohexane as the solvent.[27] This level is referred to as SMD(cyclohexane)/B3LYP-D3/6-31G(d). All stationary points have been verified, through vibrational analysis, to be minima (zero imaginary frequencies) or transition state structures (one imaginary frequency). The character of the normal mode associated with the imaginary frequency has been analyzed to ensure it resembles the reaction under consideration. Optimized structures were illustrated using CYLview20.[28] Potential energies were refined by means of single point calculations using the M06-2X functional and the 6-311+G(d,p) basis set.[29] This level is denoted SMD(cyclohexane)/M062X/6-311+G(d,p)//SMD(cyclohexane)/B3LYP-D3/6-31G(d).

Computational Details

The BIMP catalyst has a rich conformational space and we considered potential conformations of the molecule. The conformational space of the freely-rotating side chain that contains the iminophosphorane moiety is named "*right arm* – ***RA***", and there are two possible conformations for ***RA***. In addition, there are two coordination modes for which the substrate may bind to the substrate in the transition structures (TSs, Mode A and Mode B) as originally hypothesized by Pápai and Soós (**Figure S3**).[30] In the case of mode A, the thiourea coordinates to the electrophile (ester) and the protonated iminophosphorane coordinates to the nucleophile (nitronate). In the mode B, the thiourea coordinates to the nucleophile (nitronate) and the protonated iminophosphorane coordinates to the electrophile (ester). Both activation modes were considered in the calculations of the TSs described below.

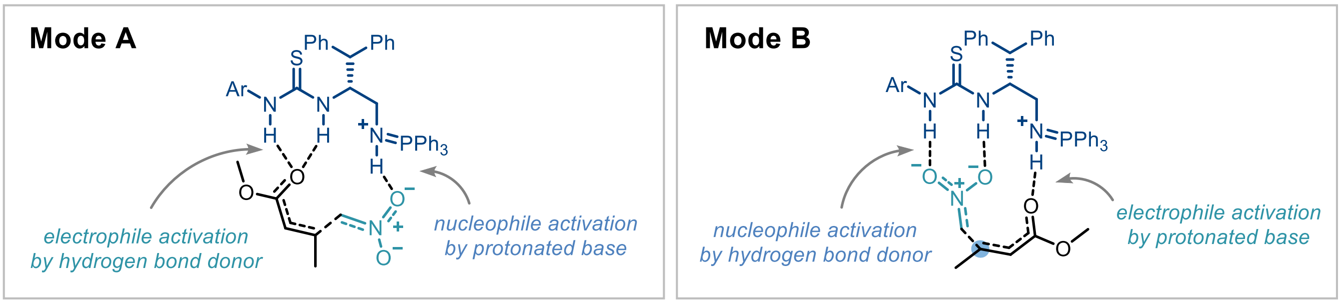

Figure S3. Activation modes of the BIMP catalyst.

In order to differentiate the computed transition states with several confirmations, they are named according to the following figure that includes information of the coordination mode, confirmations of the *right arm*, and the absolute configuration of the product.

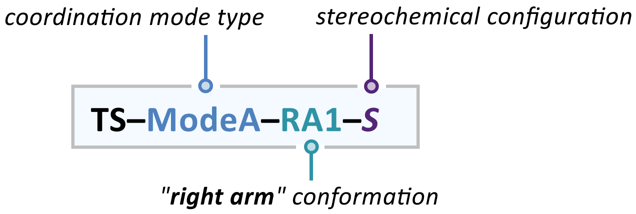

Figure S4. Nomenclature for BIMP-catalyzed Michael addition transition structures.

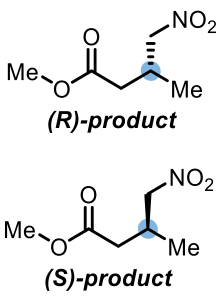

	$\Delta G^‡$	$\Delta E^‡$
TS–ModeA–RA1–*R*	25.6	–6.2
TS–ModeA–RA2–*R*	18.1	–12.4
TS–ModeB–RA1–*R*	**13.6**	**–15.1**
TS–ModeB–RA2–*R*	*n.d.*	*n.d.*
TS–ModeA–RA1–*S*	25.2	–8.9
TS–ModeA–RA2–*S*	*n.d.*	*n.d.*
TS–ModeB–RA1–*S*	**15.3**	**–11.6**
TS–ModeB–RA2–*S*	24.5	–10.0

Figure S5. Relative stability of the BIMP-catalyzed conjugate addition transition structures computed at SMD(cyclohexane)/M062X/6-311+G(d,p)//SMD(cyclohexane)/B3LYP-D3/6-31G(d). Energies (kcal mol^{-1}) are provided in the insert.

[ToC]

Table S1. Energies (electronic energies (*E*), enthalpies (*H*) and Gibbs free energies (G) in Hartrees) and imaginary frequencies (in *i* cm^{-1}) of all stationary points computed at SMD(cyclohexane)/B3LYP-D3/6-31G(d) level of theory are provided. Electronic energies (E^{high} in Hartrees) at SMD(cyclohexane)/M062X/6-311+G(d,p)//SMD(cyclohexane)/B3LYP-D3/6-31G(d) level of theory are also provided in the insert.

structure	***E***	***H***	***G***	E^{high}	***Imag. Freq.***
**1a**	-345.803718	-345.670458	-345.711890	-345.745412	-
**2a**	-245.016801	-244.961181	-244.992960	-244.987382	-
**BIMP**	-4260.180876	-4259.271778	-4259.444443	-4259.757079	-
**Int1**	-4505.220629	-4504.253914	-4504.441671	-4504.759550	-
**TS1**	-4505.206540	-4504.245704	-4504.435251	-4504.748434	1163.8*i*
**Int2**	-4505.229504	-4504.262427	-4504.445890	-4504.777497	-
**Int3**	-4851.059222	-4849.958096	-4850.166543	-4850.538356	-
**TS2-(*R*)**	-4851.042060	-4849.940498	-4850.144128	-4850.513995	324.7*i*
**TS2-(*S*)**	-4851.032460	-4849.930599	-4850.137480	-4850.508403	365.1*i*
**Int4-(*R*)**	-4851.054690	-4849.950841	-4850.154425	-4850.529980	-
**Int4-(*S*)**	-4851.043369	-4849.939296	-4850.142112	-4850.523874	-
**3a**	-590.857103	-590.663937	-590.717982	-590.771582	-
**TS-mode A-RA1-*R***	-4851.030575	-4849.928967	-4850.127856	-4850.499703	286.6*i*
**TS-mode A-RA2-*R***	-4851.041938	-4849.940140	-4850.141282	-4850.509592	325.2*i*
**TS-mode A-RA1-*S***	-4851.031266	-4849.929452	-4850.124765	-4850.504060	383.8*i*
**TS-mode B-RA2-*S***	-4851.039977	-4849.936307	-4850.132894	-4850.505815	385.3*i*

Computational Data

methyl crotonate (1a)

O	1.54288100	-0.76561700	0.00015400
C	2.88590100	-0.25558500	0.00020500
H	3.07038000	0.35279100	0.89102300
H	3.06999900	0.35417500	-0.88973200
H	3.53475200	-1.13306800	-0.00058300
C	0.56702900	0.17369700	-0.00021600
C	-0.76797800	-0.46086400	-0.00043600
O	0.79374900	1.37044400	-0.00020700
C	-1.88226100	0.28203000	0.00021200
H	-0.80071500	-1.54715900	-0.00104300
H	-1.76671400	1.36573300	0.00079600
C	-3.27676800	-0.25452900	0.00014700
H	-3.29891300	-1.34922000	-0.00066300
H	-3.82892900	0.10549600	-0.87879300
H	-3.82843400	0.10414700	0.87995000

2a (MeNO2)

C	1.32511600	0.04422800	0.00000300
N	-0.17020000	0.00016400	0.00001000
H	1.66288500	-0.48576900	0.89224900
H	1.66284400	-0.48528900	-0.89254800
H	1.64325300	1.08453900	0.00025700
O	-0.77873000	1.06651800	-0.00000200
O	-0.68730400	-1.11401800	-0.00000300

BIMP

C	3.50531600	-1.10512200	0.15607000
C	3.90517700	0.22804300	0.00088500
C	2.95948800	1.26196700	0.05646800
C	1.61611200	0.94161500	0.26641000
C	1.19909100	-0.39139800	0.41846000
C	2.15351200	-1.41439000	0.36541400
H	4.94246900	0.45770800	-0.22011000
H	1.58181400	3.26899400	-1.17625900
H	0.87990200	1.73633800	0.34291900
N	-0.18112000	-0.57834900	0.58644000
H	1.84229500	-2.43744200	0.51693400
H	3.20161200	-3.57977800	-0.95287500
C	4.50479100	-2.20229300	0.08156600

C	5.78996300	-2.04286100	0.61904900
C	6.72455200	-3.07625300	0.55181600
C	6.40181100	-4.29156800	-0.04842300
C	5.12363500	-4.45707500	-0.58132800
C	4.18524600	-3.42845600	-0.52197200
H	6.05455700	-1.11925500	1.12260100
C	8.10993200	-2.85274800	1.09590400
H	7.12449200	-5.09803000	-0.08739000
C	4.77879000	-5.74006100	-1.28916800
C	3.36598500	2.68024000	-0.11078400
C	4.59262800	3.14399900	0.38786300
C	4.96651500	4.47872500	0.23536200
C	4.12742000	5.38738100	-0.40790500
C	2.90525000	4.93324000	-0.90192100
C	2.52764700	3.59838700	-0.76188400
H	5.24983100	2.46662100	0.92205800
C	6.31507200	4.93235500	0.72847700
H	4.41855300	6.42526300	-0.51965400
C	1.95096200	5.91131700	-1.52962700
H	-0.70232400	0.28138400	0.44727500
C	-0.97453500	-1.67709400	0.86639500
N	-2.27609100	-1.39612200	0.63597400
S	-0.41478800	-3.17054000	1.42812100
H	-2.51942900	-0.40083300	0.57624300
C	-3.46035500	-2.09847300	1.12567500
C	-4.62559900	-1.05430100	1.04721000
H	-3.29190100	-2.37659500	2.17368000
C	-3.72446300	-3.42954100	0.34222100
H	-5.38752400	-1.36758900	1.76911400
H	-5.07659900	-1.11567100	0.04561800
N	-4.12976500	0.27358600	1.35389900
F	8.71342000	-4.01101700	1.44118700
F	8.09934600	-2.05711600	2.18952000
F	8.90930100	-2.24855500	0.18328600
F	3.45791800	-6.01376300	-1.22774900
F	5.44000100	-6.79645900	-0.76499100
F	5.10821800	-5.68521400	-2.60288000
F	6.71818400	4.22925800	1.80992900
F	6.31311800	6.24009800	1.06976600
F	7.26809200	4.77471000	-0.22047400
F	1.14548000	5.32493700	-2.44495900
F	2.58943500	6.93073400	-2.13937300

F	1.12936800	6.46804400	-0.59752900
H	-3.37491600	-5.85483100	1.41732400
H	-2.79117500	-3.98055300	0.47683200
H	-6.01491000	-3.16931300	-1.29933900
H	-6.08320500	-2.82847600	-3.72746700
P	-4.41908700	1.60957100	0.55972300
H	-3.18801000	3.65823800	-1.23999700
H	-6.33915900	1.06950500	2.61404600
H	-2.91908900	0.48551000	-1.62440300
C	-4.79594700	-4.29145900	1.00397500
C	-6.14187500	-3.91591000	1.15250000
C	-7.06313500	-4.75913400	1.77679000
C	-6.66145400	-5.99977500	2.27315700
C	-5.32789700	-6.38811000	2.13881200
C	-4.41182400	-5.54228300	1.51242700
H	-6.48653700	-2.95766600	0.78156600
H	-8.09804600	-4.44101700	1.87528700
H	-7.37895300	-6.65493200	2.76016400
H	-4.99693800	-7.34991200	2.52219500
C	-3.86009400	-3.22956100	-1.16741200
C	-2.67977500	-3.17265100	-1.92788800
C	-2.71580400	-2.99244200	-3.31047000
C	-3.94097700	-2.86665700	-3.96923500
C	-5.12185600	-2.92251900	-3.22889800
C	-5.08073500	-3.10324700	-1.84443500
H	-1.72371800	-3.29248100	-1.42597800
H	-1.78620000	-2.96452200	-3.87389800
H	-3.97463000	-2.73324000	-5.04762000
C	-5.73775100	2.65225100	1.28763000
C	-5.95943400	3.97923200	0.88704800
C	-6.97915500	4.72497900	1.47608700
C	-7.77874500	4.15369200	2.47110400
C	-7.55498400	2.83789600	2.87953900
C	-6.53453500	2.08819300	2.29119500
H	-5.33565700	4.43068200	0.12014200
H	-7.14777100	5.75206100	1.16411000
H	-8.57103200	4.73811800	2.93109200
H	-8.17003000	2.39598900	3.65863500
C	-2.89444800	2.61839500	0.63373200
C	-2.05137300	2.45931000	1.74657000
C	-0.89120500	3.22594500	1.85964000
C	-0.56101000	4.15150100	0.86493100

[ToC]

C	-1.38873700	4.30333400	-0.24993700
C	-2.55472600	3.54279000	-0.36512900
H	-2.30687400	1.72751700	2.50692700
H	-0.24290300	3.09813900	2.72206200
H	0.33887000	4.75025600	0.95192400
H	-1.11888200	5.00886300	-1.02973100
C	-4.82743700	1.42586100	-1.21395600
C	-6.06964200	1.78651600	-1.75471300
C	-6.34318200	1.54658400	-3.10292700
C	-5.38436600	0.94137700	-3.91597500
C	-4.14904600	0.56646100	-3.38072600
C	-3.87336000	0.80204000	-2.03661800
H	-6.82193700	2.25622700	-1.12921800
H	-7.30735500	1.83156800	-3.51498600
H	-5.60068600	0.75191700	-4.96383800
H	-3.40978800	0.07210300	-4.00183500

Int1

H	-3.91065300	-3.49680800	-0.73960600
C	1.69959000	-6.34906100	-1.47508900
H	4.06579400	-6.93116600	-0.18676300
C	5.93346100	-5.41114900	1.07465200
C	4.45479400	-3.56901100	0.24663900
H	1.58850100	-3.71597100	-1.56474700
F	6.95272100	-4.52527600	1.01493100
C	-3.13735500	-2.61632900	-3.57390500
N	-2.37806500	-1.52940000	-2.89348700
O	-2.92500000	-0.43274000	-2.80123800
F	5.64650500	-5.59072100	2.38602100
F	6.39323100	-6.59352000	0.60809900
F	1.36464700	-6.03035800	-2.74682400
F	2.11255300	-7.63152100	-1.46966900
F	0.54409100	-6.29706300	-0.75513200
F	8.33900600	3.62497700	2.08161600
F	7.73089700	1.57957400	2.53061100
F	8.81949700	2.01593000	0.70178100
F	3.96904900	6.17768900	-0.23303100
O	-1.24283600	-1.78050000	-2.49054200
N	-4.73119100	0.54952700	0.17709100
P	-4.79708400	-0.95187100	0.63493100
C	-4.10989400	1.68373000	0.83779700
C	-3.18582900	2.39959400	-0.16511500

C	-2.72686600	3.82372600	0.27037700
H	-2.30328200	3.69414400	-2.48737000
N	-2.07646800	1.52579600	-0.55520900
C	-0.96021200	1.19626700	0.12064500
S	-0.53193800	1.74552400	1.66608000
N	-0.19112500	0.29376900	-0.58171300
C	1.17287900	0.01541800	-0.41222800
C	2.11893100	1.02938600	-0.24947800
C	3.48123300	0.71555500	-0.18067900
C	3.88673700	-0.62840200	-0.26167000
C	2.94094700	-1.65454000	-0.40290800
C	1.58471100	-1.31258300	-0.48340000
H	-4.23375900	4.79607900	-1.84653300
H	-0.49487300	5.05796400	1.16062000
H	0.82072300	-2.07574100	-0.55023300
H	5.12638300	-2.87433200	0.73887000
H	4.94563000	-0.86714700	-0.25704300
H	3.31891700	3.22584200	-1.22188900
H	1.78037700	2.05439000	-0.16926900
H	-0.65996500	-0.29017800	-1.27018700
H	-2.20985000	1.06248200	-1.44686000
H	1.48605100	5.83134700	-0.11131300
H	-3.76946000	2.50200500	-1.08549600
H	-6.39971200	-3.44657600	0.74852200
H	-2.36323200	3.74131100	1.29579800
H	-4.47329400	-3.06509300	2.78827400
F	5.86783100	6.22435300	-1.28978600
F	4.10788700	5.35613800	-2.24031400
H	-4.88248000	2.40158600	1.14864700
H	-3.52283800	1.44385900	1.73473200
H	-3.09054700	-2.40411200	-4.64493000
H	1.57937100	5.53508700	-2.57189900
H	-0.34746500	4.47905500	-3.75716700
C	-3.91346500	4.78474800	0.28750900
C	-4.37359200	5.29882600	1.50707200
C	-5.46356200	6.17021400	1.55714500
C	-6.11576200	6.54297400	0.38118700
C	-5.66709100	6.03850100	-0.84186800
C	-4.57725100	5.16930000	-0.88637900
H	-3.87113700	5.01040900	2.42744700
H	-5.80106100	6.55685800	2.51548200
H	-6.96416300	7.22145000	0.41564000

H	-6.16538300	6.32462800	-1.76478500
C	-1.55560700	4.33867700	-0.56515800
C	-0.47221300	4.94956100	0.08004800
C	0.64634700	5.38283000	-0.63438300
C	0.70064400	5.21393400	-2.01981100
C	-0.37744500	4.61890000	-2.67927800
C	-1.49112800	4.18480700	-1.95780900
C	-3.26470000	-1.99110600	0.67321300
C	-2.21176800	-1.58527800	1.51070100
C	-1.04128400	-2.33800800	1.59424800
C	-0.90346200	-3.50905400	0.84312300
C	-1.93465000	-3.91319400	-0.00561300
C	-3.10939900	-3.16061500	-0.08888200
H	-2.29236700	-0.67277700	2.09359500
H	-0.23299000	-1.99830900	2.23479300
H	0.00582000	-4.10069700	0.90614100
H	-1.82014100	-4.81745400	-0.59799000
C	-5.51669200	-1.23303700	2.30543700
C	-6.44232700	-0.28157800	2.76066900
C	-7.04573100	-0.42765800	4.00937700
C	-6.73090900	-1.52601300	4.81348900
C	-5.80925600	-2.47614100	4.36823100
C	-5.20043100	-2.33021600	3.12047800
H	-6.67858900	0.57310700	2.13264800
H	-7.75793000	0.31610000	4.35641900
H	-7.19915900	-1.63868900	5.78775900
H	-5.55756600	-3.32749900	4.99508000
C	-5.90434800	-1.79206300	-0.55176900
C	-6.10673700	-1.21583100	-1.81391100
C	-6.92196200	-1.85463100	-2.75042400
C	-7.54038900	-3.06764700	-2.43357400
C	-7.34967400	-3.63815100	-1.17312300
C	-6.53626800	-3.00212400	-0.23346900
H	-5.62841200	-0.26850600	-2.03835900
H	-7.08050500	-1.40025200	-3.72514300
H	-8.17736800	-3.56103100	-3.16297800
H	-7.83884400	-4.57422700	-0.91785100
C	4.46774700	1.81874300	-0.04915900
C	5.65749000	1.65681300	0.67572400
C	6.56135900	2.71101600	0.80773600
C	6.30067700	3.95464700	0.23343400
C	5.11869600	4.12214500	-0.48664100

C	4.21765900	3.06972000	-0.63580000
H	5.86858300	0.71366500	1.16783300
C	7.85943900	2.48573900	1.53544400
H	7.00113800	4.77344400	0.34646800
C	4.77264600	5.46763400	-1.06511100
C	3.30832400	-3.09295300	-0.40969200
C	2.46397300	-4.03825100	-1.01205400
C	2.72330000	-5.40361000	-0.91195900
C	3.85970900	-5.87006600	-0.25655100
C	4.72997600	-4.93445600	0.30569800
H	-2.65901600	-3.56398100	-3.33808400
H	-4.16864500	-2.56224200	-3.22588100

TS1

H	0.26537600	-0.14046700	-2.72641300
C	-4.35918100	-4.71245200	3.72074700
H	-5.60473500	-5.24701200	1.38042300
C	-5.84934300	-3.94838700	-0.99463600
H	2.49655100	-4.12909500	1.17696600
H	3.52384200	-3.08780400	2.28452300
F	-6.37079700	-2.94074100	-1.72716600
C	2.64637500	-3.15740700	1.64357800
N	1.48057500	-2.78261200	2.37545900
O	1.59721300	-1.91929200	3.28905000
F	-4.96034000	-4.59372000	-1.79821000
F	-6.84275600	-4.82434000	-0.73480700
F	-5.46847200	-4.52786800	4.47648900
F	-4.31647300	-6.02801600	3.40821100
F	-3.29587700	-4.44541200	4.50741400
F	-5.87490100	5.16062300	-3.44573800
F	-5.56587000	3.00286800	-3.50719000
F	-7.09180900	3.84724200	-2.21260400
F	-2.01939400	6.82033800	1.15486500
O	0.37645800	-3.22930300	2.04872700
N	3.03579200	-1.28049900	-0.42732700
P	2.64789200	-1.67209800	-1.94401300
C	4.04743100	-0.24227700	-0.21609800
C	4.03278900	0.27626600	1.24170600
C	4.82294600	1.60530900	1.38346500
H	4.81211000	0.23384900	3.83538400
N	2.69266300	0.35378400	1.82067800
C	1.58799300	0.95451600	1.31544300

[ToC]

S	1.60769700	2.07789000	0.05314600
N	0.44300200	0.51908100	1.96361800
C	-0.90396400	0.63482800	1.56621800
C	-1.46998200	1.80393600	1.04743100
C	-2.80652600	1.81003600	0.62142700
C	-3.57802100	0.64508100	0.73462000
C	-3.02894500	-0.51763200	1.28963900
C	-1.69626900	-0.51269700	1.71289600
H	7.00854800	0.29902300	2.43705800
H	4.87937100	4.18301200	2.13806700
H	-1.24087200	-1.42577500	2.08720600
H	-4.59353100	-1.59346300	-0.64294900
H	-4.62112500	0.65238200	0.43356300
H	-2.35400400	4.39399200	1.38162500
H	-0.85621600	2.68589400	0.93765800
H	0.59537900	-0.27410900	2.58699200
H	2.52032200	-0.36408300	2.51926900
H	4.99096400	5.15471900	4.40984000
H	4.53390300	-0.48089700	1.85330500
H	1.53385300	-3.84780500	-3.62196700
H	4.28640800	2.33773500	0.77478600
H	3.34252600	-2.24139900	-4.81213300
F	-3.27724400	7.79569100	-0.33562000
F	-4.12678400	7.16110700	1.56131300
H	5.06309500	-0.60806700	-0.42074500
H	3.86947300	0.60665100	-0.88441600
H	2.79689500	-2.25243600	0.65237100
H	5.02113400	3.66322100	6.40547100
H	4.92893600	1.19574700	6.09108000
C	6.22323900	1.45500300	0.79599800
C	6.52404000	2.01384400	-0.45314000
C	7.78229000	1.83763400	-1.03249600
C	8.76183700	1.09750700	-0.36826100
C	8.47486200	0.54060400	0.88079500
C	7.21743000	0.72041000	1.45725100
H	5.76097900	2.58442000	-0.97728700
H	7.99699700	2.28151000	-2.00141500
H	9.74272200	0.96060000	-0.81586900
H	9.23403700	-0.03015100	1.40975000
C	4.85040900	2.13988900	2.81119200
C	4.89616300	3.52725300	3.00546500
C	4.95996500	4.07521500	4.28619300

C	4.97612900	3.24007700	5.40545300
C	4.92572500	1.85705200	5.22825100
C	4.86290400	1.31362100	3.94332400
C	2.40714900	-0.21788100	-3.01704400
C	3.51916200	0.42582000	-3.58580800
C	3.34979800	1.60622200	-4.30802000
C	2.07296900	2.14858600	-4.47165900
C	0.96522800	1.51243500	-3.90862600
C	1.12916900	0.33693200	-3.17727900
H	4.51452600	0.00808700	-3.46495100
H	4.21419000	2.10155200	-4.74141400
H	1.94234600	3.06853100	-5.03488900
H	-0.02764300	1.93630700	-4.02779300
C	3.91113500	-2.70215200	-2.77601100
C	4.79404300	-3.42401600	-1.96053100
C	5.75896800	-4.25288600	-2.53306400
C	5.84736900	-4.36495200	-3.92224400
C	4.97364400	-3.64434100	-4.74081900
C	4.00968300	-2.81166900	-4.17220900
H	4.72436100	-3.32672700	-0.88044200
H	6.44263500	-4.80706900	-1.89607600
H	6.59989600	-5.00964700	-4.36798000
H	5.04623500	-3.72635700	-5.82170600
C	1.09939900	-2.61207800	-1.89646300
C	0.15216300	-2.29525600	-0.91319700
C	-1.08180200	-2.94393600	-0.90443100
C	-1.38564500	-3.89565300	-1.87788100
C	-0.44075600	-4.21681400	-2.85700600
C	0.80064700	-3.58235700	-2.86681200
H	0.38430800	-1.55412600	-0.15796700
H	-1.80017000	-2.70796500	-0.12981700
H	-2.35741600	-4.38203400	-1.87121500
H	-0.66768800	-4.96644900	-3.60985300
C	-3.39226900	3.04892700	0.04818600
C	-4.29625200	2.98759400	-1.02209300
C	-4.83563900	4.15190700	-1.56900700
C	-4.48739500	5.40409100	-1.06674800
C	-3.58612100	5.47166200	-0.00437200
C	-3.04524100	4.31297700	0.54996400
H	-4.55782100	2.02778400	-1.45440300
C	-5.83771900	4.04364900	-2.68630100
H	-4.89700500	6.30791500	-1.50206400

C	-3.24698000	6.81169600	0.59181100
C	-3.80129900	-1.78322200	1.35414200
C	-3.72830500	-2.62147100	2.47698500
C	-4.38550100	-3.85035400	2.48667700
C	-5.11335800	-4.28087900	1.37701600
C	-5.18514000	-3.45212400	0.25919000
C	-4.54778400	-2.21129300	0.24768800
H	-3.15408500	-2.31259500	3.34348900

Int2

H	0.60716300	-1.57949700	1.10574000
C	2.58573100	-5.28920500	-3.07691600
H	4.54569100	-5.67341800	-1.26752000
C	5.76280900	-4.10598600	0.59767800
H	-1.79252100	-3.71374800	-4.25025800
H	-3.56777000	-3.80674800	-3.56337700
F	6.66227100	-3.12010000	0.81673200
C	-2.59990400	-3.33553900	-3.64127800
N	-2.39976100	-2.22842000	-2.97473700
O	-3.35039900	-1.71574200	-2.23135400
F	5.19963000	-4.39111500	1.79735900
F	6.44747500	-5.20481500	0.21295700
F	3.49027400	-5.76445300	-3.95994200
F	2.13353900	-6.36265200	-2.37488100
F	1.53805800	-4.81388200	-3.78697800
F	6.58742300	4.81980800	2.99692400
F	5.84184600	2.79215500	3.29299400
F	7.43146700	3.16717700	1.86233100
F	2.68805700	6.80205100	-1.43318100
O	-1.25284800	-1.61372300	-3.01640100
N	-3.19934300	-1.12022200	0.54081100
P	-2.25850500	-1.80923000	1.69978500
C	-3.87773300	0.17627500	0.73188000
C	-4.12985300	0.84501600	-0.63422400
C	-4.88832900	2.19026400	-0.50730600
H	-5.14549700	1.12771500	-3.09508100
N	-2.90453400	0.90899400	-1.43003800
C	-1.71073000	1.44017800	-1.05569000
S	-1.52792200	2.66574700	0.10176600
N	-0.67691100	0.83978400	-1.73746000
C	0.69450000	0.81482000	-1.43157400
C	1.45052700	1.91699500	-1.01933600

C	2.80719700	1.75415300	-0.69783200
C	3.41059500	0.49263000	-0.80492400
C	2.66492500	-0.61593900	-1.23367300
C	1.31561400	-0.43679900	-1.54716100
H	-7.14940600	0.92178700	-1.43636900
H	-5.01737700	4.83964600	-0.92847300
H	0.70812600	-1.28143700	-1.84673600
H	4.66234000	-1.68121000	0.30458200
H	4.47117800	0.38629900	-0.59913800
H	2.67720600	4.34109600	-1.56689800
H	0.97227000	2.87939100	-0.90884800
H	-0.96474700	0.00596900	-2.27641400
H	-2.85775700	0.07178900	-2.02408000
H	-5.39842800	6.08011400	-3.03470700
H	-4.76478400	0.15667000	-1.20183700
H	-1.75251000	-4.52877800	2.70941900
H	-4.27640000	2.84191500	0.12023000
H	-1.71738900	-2.21571700	4.58966700
F	4.15342500	7.65028600	-0.05897900
F	4.79538000	6.82434300	-1.96364600
H	-4.83331800	0.02819600	1.24429600
H	-3.25610300	0.83699000	1.34271000
H	-3.17755800	-1.50740900	-0.42155400
H	-5.66807400	4.84180400	-5.18000100
H	-5.53675800	2.35610900	-5.18407200
C	-6.22517400	1.97768700	0.20015700
C	-6.41710500	2.45365300	1.50336400
C	-7.62074300	2.23439400	2.17750300
C	-8.65313400	1.53115600	1.55516400
C	-8.47437400	1.05464400	0.25347400
C	-7.27236700	1.27865500	-0.41725300
H	-5.61385400	2.99917300	1.99348700
H	-7.75067000	2.61583800	3.18721200
H	-9.59176700	1.36082800	2.07598700
H	-9.27544500	0.51298800	-0.24317500
C	-5.07135500	2.89519900	-1.84769400
C	-5.13831400	4.29476100	-1.86180600
C	-5.35410400	4.99403000	-3.04884600
C	-5.50455500	4.30071500	-4.25164800
C	-5.43287100	2.90714100	-4.25279600
C	-5.21729700	2.21053000	-3.06196000
C	-0.96155800	-0.70321400	2.31901800

[ToC]

C	-1.28013900	0.31614800	3.23306900
C	-0.30404600	1.23111500	3.62193600
C	0.99002700	1.13707800	3.10522900
C	1.31200400	0.12127600	2.20464900
C	0.34368300	-0.79911900	1.81016800
H	-2.28381200	0.39558900	3.64012100
H	-0.55786100	2.02357300	4.31954300
H	1.74602500	1.85993900	3.39857600
H	2.31221900	0.04894300	1.79127400
C	-3.29569400	-2.27919500	3.10803900
C	-4.65256200	-2.54728600	2.86970300
C	-5.47224600	-2.95867300	3.91949100
C	-4.94172600	-3.11064800	5.20275800
C	-3.58987200	-2.84992500	5.44158300
C	-2.76415000	-2.43131300	4.39882100
H	-5.05741400	-2.42807100	1.86915100
H	-6.52392600	-3.15742700	3.73550800
H	-5.58315500	-3.43000300	6.01938600
H	-3.17939800	-2.96617900	6.44036700
C	-1.49971400	-3.26995100	0.96264000
C	-0.99076200	-3.18300900	-0.34161500
C	-0.34187100	-4.28013000	-0.90428100
C	-0.18081400	-5.45357700	-0.16525000
C	-0.68844500	-5.53959800	1.13401100
C	-1.35248800	-4.45313700	1.70297300
H	-1.09565700	-2.27900700	-0.92954800
H	0.03275100	-4.20916600	-1.91874500
H	0.34464300	-6.29535300	-0.60494200
H	-0.57098100	-6.45555100	1.70583500
C	3.59207400	2.92161300	-0.22126400
C	4.53397200	2.77957300	0.80715100
C	5.25323300	3.87973300	1.27547300
C	5.05330400	5.14599700	0.73129900
C	4.11634800	5.29414100	-0.29207300
C	3.39433400	4.20053400	-0.76561100
H	4.68775900	1.81001100	1.26928400
C	6.27631300	3.67141700	2.35851800
H	5.60343800	6.00155400	1.10435100
C	3.93277300	6.64326600	-0.93466000
C	3.23777300	-1.98490500	-1.28923200
C	2.72023700	-2.94250800	-2.18055900
C	3.17897700	-4.25512000	-2.15952100

C	4.18870700	-4.64974300	-1.27923000
C	4.72583000	-3.70025900	-0.41483500
C	4.25156200	-2.38663300	-0.40963700
H	1.94996100	-2.66343100	-2.88955200

Int3

H	-0.54941700	0.84233000	1.36786500
C	3.76232000	5.36888100	-1.69171900
H	5.67118100	3.51937800	-2.50580800
H	6.03731600	-0.57930600	-1.06062600
H	2.07977000	2.85421400	-4.47226500
H	3.87614500	2.33294700	-4.11223400
C	4.24558300	4.11175800	-1.04786200
C	2.81353200	2.26848400	-3.93986700
N	2.38584100	1.40829700	-3.04718400
O	3.21922200	0.66651300	-2.37206000
C	5.17795700	3.30083800	-1.56333600
C	5.54557200	2.04089700	-0.90570400
O	6.48912400	1.38342900	-1.59817500
H	3.78400500	3.82688400	-0.10500000
H	2.69827100	5.27421200	-1.94462200
H	3.84658000	6.22058200	-1.00359900
H	4.31335000	5.60088700	-2.60873900
C	6.88483300	0.10717600	-1.06997600
O	5.09574100	1.62427700	0.16016300
O	1.11163900	1.28398500	-2.80054200
N	3.07114300	-0.13285500	1.04664900
P	2.23735400	0.67717900	2.20755300
C	3.28233400	-1.58412100	0.95988700
C	3.30503100	-1.98923300	-0.52892300
C	3.67484100	-3.47831600	-0.76598000
H	3.90387000	-1.92415400	-3.11543900
N	2.10009000	-1.54462600	-1.21085100
C	0.81629400	-1.87198500	-0.96605600
S	0.31481600	-3.13937300	0.04938400
N	-0.03836900	-1.02869500	-1.64873800
C	-1.36285300	-0.68969500	-1.32707400
C	-2.38118000	-1.60724900	-1.04967300
C	-3.66292400	-1.14818300	-0.70573300
C	-3.92631700	0.22946700	-0.65529700
C	-2.91548000	1.15697100	-0.95096800
C	-1.64459200	0.68255800	-1.28548200

H	6.13677600	-2.87920100	-1.83667000
H	3.21261100	-5.93898200	-1.71987700
H	-0.83090500	1.37416600	-1.46696400
H	-4.56361300	2.52818200	0.75522700
H	-4.93209800	0.57580300	-0.43843200
H	-3.45849200	-3.44768800	0.75333000
H	-2.15898100	-2.66485000	-1.06273400
H	0.44393300	-0.26062000	-2.14794100
H	2.29790500	-0.67655000	-1.74225800
H	3.13569300	-6.73481800	-4.06068000
H	4.08876300	-1.38439000	-0.99700400
H	1.78219800	3.36301900	3.33622700
H	2.94770200	-4.07940000	-0.21392600
H	1.57603900	1.07892500	5.08266300
H	7.65907500	-0.26275200	-1.74327600
H	7.27757800	0.20655100	-0.05364400
H	4.22592100	-1.86488500	1.43830800
H	2.46457700	-2.09927800	1.46627200
H	3.75652900	0.44687800	0.54901700
H	3.46458800	-5.12603000	-5.93548300
H	3.85111000	-2.71920800	-5.42931700
C	5.06217700	-3.76698000	-0.19420200
C	5.20151600	-4.41980200	1.03741600
C	6.46277800	-4.63425000	1.59861500
C	7.60933400	-4.19657200	0.93386700
C	7.48472800	-3.55521800	-0.30159400
C	6.22352500	-3.34885000	-0.86107100
H	4.31117900	-4.75764600	1.56305800
H	6.54781900	-5.14563100	2.55417300
H	8.59161000	-4.36267100	1.36831200
H	8.37196300	-3.22525300	-0.83650200
C	3.58410700	-3.88331000	-2.23497600
C	3.35823000	-5.23370700	-2.53494900
C	3.31712200	-5.68302400	-3.85412200
C	3.50088700	-4.78181700	-4.90510100
C	3.72036000	-3.43370600	-4.62055700
C	3.76173200	-2.98528000	-3.29799200
C	0.76977600	-0.25554300	2.69119500
C	0.86760800	-1.30558900	3.61956300
C	-0.26442400	-2.05240200	3.93767800
C	-1.49150100	-1.75498000	3.33909100
C	-1.59028900	-0.71056100	2.41974800

[ToC]

C	-0.46406300	0.04042000	2.09230700
H	1.81876000	-1.53692400	4.09005800
H	-0.18737700	-2.86916700	4.64927800
H	-2.37013700	-2.34472200	3.58460900
H	-2.53667100	-0.48608400	1.93906300
C	3.23295900	1.00402100	3.69131300
C	4.62274100	1.13046300	3.53308800
C	5.41803500	1.43232100	4.63774700
C	4.83615800	1.60831900	5.89556800
C	3.45380600	1.48547600	6.05404400
C	2.64929300	1.18362900	4.95525600
H	5.07610700	1.00440300	2.55412300
H	6.49294800	1.52895200	4.51566100
H	5.46065600	1.84013300	6.75389700
H	3.00140300	1.61958400	7.03240000
C	1.74699600	2.25133900	1.47540500
C	1.46715900	2.29250900	0.10208000
C	0.97067000	3.46605000	-0.46338000
C	0.75896700	4.59425500	0.33163500
C	1.05765800	4.55695200	1.69729900
C	1.55087000	3.38792600	2.27543800
H	1.64563500	1.43678400	-0.53682700
H	0.75995200	3.47621000	-1.52688300
H	0.36214700	5.50248600	-0.11083600
H	0.90439100	5.43805600	2.31377200
C	-4.73269300	-2.12801600	-0.38588800
C	-6.04558500	-1.93143700	-0.83796200
C	-7.05512800	-2.83982800	-0.51916600
C	-6.78335500	-3.96485900	0.25659600
C	-5.47971800	-4.16543400	0.71020400
C	-4.46515200	-3.26341100	0.39485800
H	-6.29099300	-1.04752500	-1.41688500
C	-8.44230300	-2.61766400	-1.05757900
H	-7.57134500	-4.66244600	0.51435000
C	-5.15329100	-5.40780500	1.49534300
C	-3.12449100	2.62307200	-0.85182000
C	-2.38518200	3.50248500	-1.66190200
C	-2.47655700	4.88021800	-1.48699800
C	-3.33831200	5.42537200	-0.53374500
C	-4.10347500	4.56017700	0.24526400
C	-3.99221400	3.17632900	0.09949000
H	-1.72160600	3.10698500	-2.42180900

C	-1.64412500	5.80611600	-2.33185300
H	-3.40665400	6.49861200	-0.39730600
C	-4.98049100	5.11589300	1.33441300
F	-6.07437900	4.34879500	1.54135300
F	-4.32134000	5.18165500	2.51755800
F	-5.40658700	6.36697900	1.05373900
F	-2.35098800	6.36272300	-3.33856600
F	-1.14969100	6.83321400	-1.59117500
F	-0.58264500	5.17783300	-2.88993100
F	-9.38621700	-3.20514700	-0.28988100
F	-8.74604200	-1.30067900	-1.13748300
F	-8.57940200	-3.12365500	-2.30677700
F	-4.12140000	-5.21444700	2.34441400
F	-6.20940500	-5.83167500	2.22646800
F	-4.80552900	-6.43275800	0.68015300

TS2-(R) (TS-mode B-RA1-R)

H	1.70622431	1.13851886	-1.30379034
C	-3.97766369	4.07803986	4.05295266
H	-5.32088669	1.83735986	3.12704166
H	-4.89736169	-0.06428114	-0.93184434
H	-1.41937569	2.77327286	4.48994466
H	-2.88481269	1.71402086	4.94964666
C	-3.55863469	3.07232286	3.00064766
C	-2.17467469	2.05069286	4.20694866
N	-1.72413869	1.07044186	3.38260266
O	-2.48384469	0.08671786	3.13271866
C	-4.48539569	2.15566286	2.51118266
C	-4.41561669	1.62502386	1.19293266
O	-5.43993469	0.74903086	0.92959466
H	-2.82458969	3.42896186	2.27826066
H	-3.12267469	4.55151786	4.54667166
H	-4.56809269	4.87344686	3.57855566
H	-4.60843669	3.61529986	4.82054466
C	-5.70569769	0.48968886	-0.44944834
O	-3.60272369	1.93277086	0.29813066
O	-0.61716069	1.22613686	2.74695266
N	-1.94943269	0.20809286	-1.08810034
P	-1.05701869	1.05745286	-2.17640934
C	-2.33913169	-1.20016414	-1.23400734
C	-2.58449369	-1.79719114	0.16813866
C	-3.03987769	-3.28014814	0.12560366

[ToC]

H	-3.82842969	-2.06935814	2.54098466
N	-1.45018169	-1.55835114	1.05247266
C	-0.14831169	-1.83585814	0.82572566
S	0.41577431	-2.95179114	-0.32121634
N	0.67203831	-1.08228614	1.64482766
C	2.02396431	-0.75941314	1.41907866
C	3.02542331	-1.70253914	1.16875666
C	4.33508031	-1.27971314	0.89604366
C	4.64378131	0.08883186	0.90352966
C	3.65262531	1.04106886	1.18264866
C	2.34905231	0.60290586	1.43776666
H	-5.60800869	-2.50768214	0.75210666
H	-2.59454169	-5.82987314	0.84909266
H	1.55389331	1.32280886	1.59292766
H	5.39666331	2.39011086	-0.43225634
H	5.66861131	0.40850186	0.74132366
H	4.69783631	-3.74817714	2.01184566
H	2.77014331	-2.75236814	1.14348766
H	0.17582831	-0.32215514	2.12992266
H	-1.67318369	-0.90199714	1.80938466
H	-2.89679869	-6.91452314	3.05193266
H	-3.39345969	-1.21260314	0.62001766
H	-0.16025469	3.64629786	-3.19105434
H	-2.24304569	-3.83516514	-0.37597834
H	-0.40830769	1.38391786	-5.08787534
H	-6.60981569	-0.12032114	-0.47009534
H	-5.87665669	1.42431286	-0.99650334
H	-3.24739969	-1.30363514	-1.83746634
H	-1.53423969	-1.74731514	-1.72637034
H	-2.59835369	0.79312686	-0.52832734
H	-3.67401669	-5.57614214	5.00464866
H	-4.13140069	-3.14416114	4.71810566
C	-4.31261569	-3.43213514	-0.70285134
C	-4.26359369	-4.02591014	-1.97101434
C	-5.41435269	-4.14456814	-2.75358834
C	-6.63714769	-3.66993714	-2.27620534
C	-6.69879669	-3.07953614	-1.01119934
C	-5.54709669	-2.96204414	-0.23280134
H	-3.31297469	-4.39746314	-2.34706234
H	-5.35472369	-4.61163914	-3.73346434
H	-7.53556669	-3.76471714	-2.88044134
H	-7.64765869	-2.71438914	-0.62600734

C	-3.20193669	-3.87787814	1.52025866
C	-2.93957569	-5.24283214	1.69713766
C	-3.11019969	-5.85454314	2.93882666
C	-3.54539369	-5.10484614	4.03364266
C	-3.80421869	-3.74312214	3.87213166
C	-3.63500769	-3.13393614	2.62723066
C	0.39151631	0.10641786	-2.67984534
C	0.28458431	-0.92368314	-3.62899234
C	1.40065531	-1.70022814	-3.93276234
C	2.62013331	-1.45454314	-3.29663534
C	2.72870431	-0.42843114	-2.35763934
C	1.61904931	0.35322986	-2.04627934
H	-0.66188469	-1.11909514	-4.12465534
H	1.31589431	-2.50247814	-4.65978434
H	3.48519731	-2.06931614	-3.52872634
H	3.66887731	-0.24398314	-1.84819634
C	-2.01995169	1.50696386	-3.64917234
C	-3.37864769	1.80320786	-3.44855234
C	-4.16727569	2.19442086	-4.52831834
C	-3.60772269	2.29254586	-5.80543934
C	-2.25578269	2.00527886	-6.00513434
C	-1.45766969	1.61313686	-4.92951534
H	-3.80785669	1.74044786	-2.45160934
H	-5.21797269	2.42209786	-4.37341134
H	-4.22651769	2.59424786	-6.64595334
H	-1.82182369	2.08348686	-6.99782034
C	-0.54148969	2.57318386	-1.34522834
C	-0.49978269	2.61881886	0.05446466
C	-0.02587269	3.76680386	0.68915166
C	0.40795431	4.85956986	-0.06326334
C	0.36081031	4.81309986	-1.45992934
C	-0.11722769	3.67465086	-2.10575834
H	-0.82562969	1.78207386	0.65914066
H	0.01142431	3.78849686	1.77276066
H	0.79174831	5.74298786	0.43716266
H	0.69440631	5.66443386	-2.04615834
C	5.38380631	-2.28320814	0.58126766
C	6.34685931	-2.02685614	-0.40447634
C	7.31807831	-2.97757114	-0.72057334
C	7.35474331	-4.20481314	-0.06341334
C	6.39777731	-4.46678414	0.91786266
C	5.42505531	-3.52243214	1.23973266

H	6.32388531	-1.09106714	-0.95325934
C	8.34850031	-2.64066914	-1.76369134
H	8.10206331	-4.94669414	-0.31782834
C	6.46060431	-5.76190314	1.68314066
C	3.93215631	2.49813386	1.15017766
C	3.22919731	3.38008986	1.98756666
C	3.42572331	4.75523586	1.89767766
C	4.34367731	5.29068286	0.99343666
C	5.06019231	4.41953186	0.17576566
C	4.85358031	3.04124486	0.24414066
H	2.51905231	2.99058586	2.70744266
C	2.65678731	5.68869186	2.79216666
H	4.49182931	6.36228086	0.92313766
C	5.99448831	4.97548186	-0.86471934
F	7.01937531	4.13297986	-1.12416034
F	5.35609231	5.18799286	-2.04160434
F	6.52516131	6.15953786	-0.48780834
F	3.39817331	6.12712386	3.83302166
F	2.24728431	6.79355186	2.11608666
F	1.54922131	5.10794286	3.30924966
F	8.96692631	-3.73883414	-2.24749234
F	7.79802031	-1.98772414	-2.81533434
F	9.31164631	-1.82776314	-1.26711934
F	5.24378431	-6.15253014	2.11938666
F	6.96591731	-6.76574614	0.93088366
F	7.25448931	-5.65398314	2.77588566

TS2-(S) (TS-mode B-RA1-S)

H	-3.67016473	-0.08623739	-0.95366556
C	-3.06183173	4.15324561	3.02262644
H	-1.93403373	4.25702461	-0.19566756
H	-1.13762073	4.77010661	1.39732944
H	-2.82254973	2.29095061	1.88918744
C	-6.47110373	1.64613061	-1.36455656
C	-3.18657773	3.31115961	1.76793744
C	-4.32931373	3.43497861	0.95980644
C	-4.80643173	2.36456861	0.16252844
O	-5.88194373	2.70797161	-0.61154456
O	-4.36715073	1.19335661	0.13151344
C	-1.53924873	3.98198561	0.77234144
N	-0.73627973	2.86883261	0.72796144
O	0.02757427	2.59198061	1.70106544

[ToC]

O	-0.89302173	2.02831761	-0.21808756
N	-3.11119573	-0.89353239	-1.26467156
H	-4.86510573	4.37813261	0.90363544
C	-2.82596173	-1.87332739	-0.19927856
C	-2.45939273	-1.12354039	1.09616344
C	-2.72674773	-1.90475739	2.40896144
H	-5.08894173	-0.61448639	1.57546844
N	-1.12721573	-0.53883539	1.00289944
C	0.08880027	-1.11732139	1.12792144
S	0.34098827	-2.79761139	1.19180544
N	1.06274527	-0.14997739	1.19698344
C	2.44488127	-0.16032539	0.99627144
C	3.24875327	-1.30274539	0.90071944
C	4.61909527	-1.18127139	0.62945844
C	5.19236427	0.08669061	0.46782944
C	4.40012927	1.24042761	0.57751344
C	3.03518527	1.10662961	0.83786244
H	-3.79206873	0.46722361	3.32501744
P	-2.28381273	-0.69966039	-2.67367956
H	2.39715227	1.98462961	0.87723544
H	3.76592027	3.53738061	1.90789644
H	6.26335827	0.18109361	0.31993044
H	6.70454627	-1.63513939	-1.06790756
H	2.78914427	-2.27505639	0.99915544
H	-1.11926973	0.43117761	0.67687544
H	0.69126327	0.79910061	1.31439844
H	-3.10146273	-0.24331039	1.13088244
H	-3.71883373	-2.48575439	-0.04511556
H	-2.00978773	-2.53538839	-0.49510256
H	-2.11520873	-2.80881939	2.40274044
H	-2.07213373	4.05495661	3.47809844
H	-3.25693073	5.21275961	2.81989044
H	-3.80729073	3.81620861	3.75480944
H	-7.31895273	2.09278161	-1.88888456
H	-5.76646773	1.23004761	-2.09162056
H	-6.82089573	0.83983461	-0.71017156
H	-2.58903973	2.00378461	-1.64276956
H	-4.41126573	-2.65003039	-2.66044956
H	-0.42254973	1.59673161	-2.82744456
C	-2.61097173	-2.13255339	-3.73200856
C	-1.76809173	-2.44606439	-4.81077456
C	-2.08143373	-3.51783139	-5.64506256

[ToC]

C	-3.22652473	-4.28173439	-5.40361256
C	-4.06501973	-3.97269039	-4.33033256
C	-3.76361873	-2.89698739	-3.49585856
H	-0.86965773	-1.86404539	-4.99121856
H	-1.42797873	-3.76147739	-6.47757756
H	-3.46335073	-5.12107639	-6.05145156
H	-4.95192973	-4.56980839	-4.13984656
C	-3.00649373	0.79257561	-3.39682056
C	-3.58216073	0.76168261	-4.67551156
C	-4.19710173	1.90701861	-5.18127956
C	-4.24414573	3.07413761	-4.41555456
C	-3.66542073	3.10484061	-3.14456756
C	-3.04025373	1.97054561	-2.62928256
H	-3.56217373	-0.14803839	-5.26699856
H	-4.64679273	1.88338961	-6.16965156
H	-4.73519873	3.95997661	-4.80860756
H	-3.71291273	4.00611161	-2.54140156
C	-0.48242673	-0.55090039	-2.54587556
C	0.28333527	-1.70536939	-2.30139156
C	1.66650027	-1.60819439	-2.17484256
C	2.29468727	-0.36694839	-2.29231256
C	1.53868027	0.78123261	-2.53143756
C	0.15389727	0.69599361	-2.65818456
H	-0.19319373	-2.67525839	-2.20470456
H	2.24883227	-2.49790339	-1.95979556
H	3.37119127	-0.29282139	-2.17152756
H	2.02500127	1.74918961	-2.60584256
C	-4.18689073	-2.33526939	2.54226244
C	-4.47153373	-3.55095039	3.18061644
C	-5.78601373	-3.97647139	3.37242744
C	-6.84901773	-3.19137839	2.92080644
C	-6.58059173	-1.98212939	2.27785944
C	-5.26393273	-1.55523839	2.08997944
H	-3.64857573	-4.16644139	3.53689644
H	-5.97892473	-4.92234939	3.87240844
H	-7.87471373	-3.52015139	3.06669244
H	-7.39860573	-1.36216839	1.91859944
C	-2.28254973	-1.05226339	3.59802444
C	-1.18660973	-1.44218739	4.37479344
C	-0.74236373	-0.64693739	5.43405444
C	-1.39055973	0.55194561	5.73119144
C	-2.48964173	0.94746561	4.96437344

[ToC]

C	-2.93232173	0.15087361	3.90894444
H	-0.66334173	-2.36260739	4.13198344
H	0.11535727	-0.96458839	6.02159544
H	-1.04377973	1.17433861	6.55201444
H	-3.00394073	1.87826961	5.18811944
C	4.99175027	2.59099261	0.40210844
C	6.01565927	2.81366261	-0.53092956
C	6.56573127	4.08375961	-0.69734856
C	6.10705427	5.16513761	0.05388344
C	5.08891227	4.95049761	0.98135944
C	4.53732327	3.68190161	1.15881344
H	6.37899327	1.99432161	-1.14187956
C	7.61495927	4.30093261	-1.75392556
H	6.54155727	6.14957561	-0.07238856
C	4.53785327	6.10668661	1.77249044
C	5.45188327	-2.40615639	0.51243244
C	5.20149227	-3.53001239	1.31540944
C	5.97950127	-4.68027439	1.19207344
C	7.02691127	-4.74169739	0.27345244
C	7.28074127	-3.62865439	-0.52600856
C	6.50367927	-2.47613439	-0.41301656
H	4.40767727	-3.50112639	2.05387644
C	5.64596227	-5.89109839	2.02243244
H	7.63060527	-5.63674839	0.18227744
C	8.43939827	-3.64506839	-1.48587456
F	8.43002927	5.33580961	-1.45337956
F	8.39136127	3.20707761	-1.92582856
F	7.05787827	4.57641161	-2.95853856
F	4.18530127	5.73770461	3.02361444
F	5.42781427	7.11803961	1.88139044
F	3.42743127	6.62029661	1.18934044
F	4.71580027	-6.66694839	1.41597244
F	6.72941527	-6.67254539	2.23412344
F	5.14649427	-5.55258939	3.23102044
F	8.18436627	-2.91291239	-2.59510556
F	9.55772927	-3.12509939	-0.92549856
F	8.74791527	-4.89756639	-1.88876056

Int4-(R)

H	-0.83162800	1.16005100	1.56056200
C	4.94367700	3.72983000	-3.91902700
H	5.88571700	1.11819500	-3.25020500

H	5.86661400	0.14864300	1.18318300
H	2.47061900	2.76981800	-4.51524400
H	3.70151700	1.55452100	-5.02763400
C	4.22956000	2.59890600	-3.15178800
C	3.17332300	2.00444500	-4.18669300
N	2.41356400	0.95155300	-3.53396100
O	2.97930600	-0.14346200	-3.40398200
C	5.15977800	1.59175900	-2.59688900
C	5.18627500	1.29748400	-1.25034500
O	6.11825100	0.31027500	-0.90101500
H	3.64662100	3.03168000	-2.33101900
H	4.23631500	4.44966700	-4.35227900
H	5.60777000	4.26990300	-3.23758000
H	5.55849600	3.32414200	-4.73220300
C	6.61932900	0.40032100	0.42859100
O	4.48530200	1.81718800	-0.32220400
O	1.32352000	1.23312700	-2.97942400
N	2.83576700	0.24529900	1.09137200
P	2.01757900	1.17378400	2.16630900
C	3.21200800	-1.16026100	1.29085800
C	3.40427900	-1.81030500	-0.09448300
C	3.81876600	-3.30312100	-0.03557500
H	4.80610000	-2.13372400	-2.39304700
N	2.24685200	-1.58345500	-0.95934200
C	0.94558100	-1.82571500	-0.69277600
S	0.38285900	-2.84404100	0.53803700
N	0.11220600	-1.12640200	-1.55094300
C	-1.23642400	-0.78534600	-1.30528200
C	-2.23593700	-1.72660100	-1.04472600
C	-3.54258900	-1.30192300	-0.76047400
C	-3.84707500	0.06805400	-0.77139200
C	-2.85809500	1.01677100	-1.06419500
C	-1.55485500	0.57648400	-1.32184600
H	6.40870300	-2.50852400	-0.55489500
H	3.17396900	-5.79541600	-0.82550500
H	-0.76081800	1.29690300	-1.48160300
H	-4.54027000	2.41317300	0.57910600
H	-4.86972700	0.39231900	-0.60407100
H	-3.91427900	-3.76828400	-1.87606400
H	-1.98182800	-2.77722500	-1.02547600
H	0.58797800	-0.40834400	-2.10050300
H	2.45303500	-1.02971300	-1.78928800

H	3.44562000	-6.85991300	-3.04240900
H	4.21482400	-1.25624000	-0.57689600
H	0.97778300	3.74453100	3.04699300
H	2.99764500	-3.83494400	0.45129800
H	1.63756200	1.68188400	5.10942000
H	7.43561100	-0.32166300	0.49676400
H	7.00496000	1.40585400	0.64278500
H	4.14724300	-1.24959500	1.85397800
H	2.42498000	-1.67614400	1.84151400
H	3.51028500	0.80364600	0.49993000
H	4.40189700	-5.56068200	-4.94104800
H	5.07314900	-3.18813600	-4.58625600
C	5.06944100	-3.49989800	0.81491700
C	4.98156000	-4.15325300	2.05104800
C	6.11167000	-4.32747500	2.85310700
C	7.35148600	-3.84815100	2.42777000
C	7.45036300	-3.19482600	1.19645100
C	6.32001300	-3.02247000	0.39747100
H	4.01721300	-4.52840300	2.38634100
H	6.02229500	-4.84064500	3.80737700
H	8.23397000	-3.98505300	3.04728100
H	8.41205800	-2.82075600	0.85408500
C	3.97993900	-3.89575000	-1.43362000
C	3.59909100	-5.22648100	-1.64911200
C	3.75152500	-5.82654800	-2.89927900
C	4.28672700	-5.09878000	-3.96386400
C	4.66464300	-3.77009200	-3.76407300
C	4.51395200	-3.17321300	-2.51120600
C	0.63286600	0.25894900	2.87711900
C	0.85324900	-0.69148100	3.88814500
C	-0.21203400	-1.45147800	4.36632400
C	-1.49458900	-1.26669400	3.84366600
C	-1.71539000	-0.32163400	2.84115500
C	-0.65634700	0.44106200	2.35373700
H	1.84864700	-0.83647600	4.29805900
H	-0.03982200	-2.19090300	5.14281700
H	-2.32121600	-1.86584600	4.21483800
H	-2.70825100	-0.18688600	2.42321800
C	3.08267000	1.79578900	3.50195100
C	4.38995100	2.16321900	3.13610000
C	5.25372400	2.67989400	4.09964800
C	4.82296400	2.83390500	5.42060400

C	3.52193000	2.47825800	5.78156100
C	2.64743400	1.96066200	4.82462600
H	4.71959800	2.05939500	2.10415300
H	6.26408900	2.96263800	3.81833400
H	5.50223700	3.23367500	6.16852100
H	3.18624400	2.60064600	6.80744200
C	1.40517500	2.60774800	1.25224800
C	1.36546400	2.59261900	-0.14795400
C	0.85229800	3.69354400	-0.83394600
C	0.37830300	4.80184800	-0.12965400
C	0.41772600	4.81568800	1.26727700
C	0.93535100	3.72419600	1.96148400
H	1.73393100	1.73883800	-0.69992900
H	0.81606500	3.67241600	-1.91759300
H	-0.02849100	5.65234700	-0.66717900
H	0.05016900	5.67816600	1.81558800
C	-4.59494700	-2.30147300	-0.44494700
C	-5.55933400	-2.03983900	0.53892200
C	-6.53537200	-2.98565400	0.85195100
C	-6.57588800	-4.21329400	0.19416100
C	-5.61900500	-4.47966500	-0.78496900
C	-4.64102700	-3.53928500	-1.10445800
H	-5.53236700	-1.10485800	1.08837300
C	-7.57712800	-2.65218100	1.88522000
H	-7.32681900	-4.95196600	0.44796800
C	-5.68516800	-5.77380500	-1.55163700
C	-3.14182100	2.47268000	-1.06380400
C	-2.47901300	3.32503900	-1.96119500
C	-2.68259000	4.70134600	-1.91704200
C	-3.56530700	5.26534700	-0.99551400
C	-4.24012800	4.42229100	-0.11490800
C	-4.02911400	3.04312700	-0.14110500
H	-1.79500300	2.91114600	-2.69295300
C	-1.95544500	5.59911200	-2.88042900
H	-3.71749500	6.33798900	-0.95916600
C	-5.13086200	5.01203500	0.94522100
F	-6.14718500	4.18134300	1.26838500
F	-4.44522300	5.25411700	2.08929800
F	-5.67202500	6.18720600	0.55635300
F	-2.72139200	5.94682300	-3.93726900
F	-1.56886800	6.75470400	-2.28221500
F	-0.83744300	5.01653700	-3.37490300

[ToC]

F	-8.10068500	-3.75927400	2.45495000
F	-7.07106900	-1.88415100	2.87859800
F	-8.60929800	-1.95961400	1.34673400
F	-4.46675900	-6.17416700	-1.97534800
F	-6.20666000	-6.77378200	-0.80590200
F	-6.46587200	-5.65799500	-2.65298900

Int4-(S)

H	4.71853200	-0.04749400	-0.59887000
C	4.14996400	-5.61650200	0.50494400
H	2.32940000	-4.72254500	-1.82614700
H	1.74138000	-5.32491800	-0.23486700
H	3.62071800	-3.53481300	0.63906900
C	7.18162300	-1.29439400	-1.93490600
C	3.73201800	-4.28416900	-0.15202600
C	4.71293500	-3.80996900	-1.15499000
C	5.40231400	-2.61769300	-1.05551300
O	6.39212700	-2.47047800	-2.02363900
O	5.23873500	-1.67101800	-0.21844400
C	2.27034700	-4.52889200	-0.75673000
N	1.47406000	-3.31767800	-0.55882200
O	0.70394600	-3.26763200	0.41943800
O	1.69564100	-2.33481200	-1.28960900
N	4.21959300	0.86114100	-0.47975700
H	5.03428500	-4.51276200	-1.92031100
C	3.86179200	1.16483700	0.91705700
C	3.27001100	-0.10682900	1.55328600
C	3.38613300	-0.20971300	3.09859600
H	5.81343700	-1.01413800	1.91153500
N	1.92193000	-0.35481100	1.04256100
C	0.74846400	0.23634200	1.37025400
S	0.62914900	1.66064400	2.28117900
N	-0.30809700	-0.49954700	0.88147200
C	-1.67169300	-0.23207200	0.72854100
C	-2.26662500	1.02253200	0.90475600
C	-3.63353600	1.20225400	0.65554800
C	-4.41521000	0.11734900	0.23908700
C	-3.83526800	-1.14813800	0.06715900
C	-2.46798000	-1.31156900	0.30887000
H	4.42386600	-2.75922800	2.88836000
P	3.69810200	1.67045500	-1.80158500
H	-2.00151200	-2.27927000	0.14467500

H	-3.65234300	-3.74341600	0.87847500
H	-5.47408900	0.25323700	0.04503700
H	-6.16364400	1.88366200	1.48235400
H	-1.65805500	1.84155400	1.25540600
H	1.87727200	-1.05225400	0.30741300
H	-0.04874200	-1.44958800	0.61140800
H	3.84147900	-0.94334000	1.14787100
H	4.75897000	1.48034000	1.45612200
H	3.13706000	1.98079900	0.95784000
H	2.79671900	0.59509400	3.54177700
H	3.42526000	-5.94906900	1.25882400
H	4.25131700	-6.41067900	-0.24605800
H	5.12372800	-5.49359900	0.98927600
H	7.94231100	-1.38788200	-2.71549800
H	6.59642600	-0.38475800	-2.11874300
H	7.66824100	-1.19869700	-0.95678900
H	3.53245400	-1.21666700	-2.22185500
H	6.03445100	2.69998000	-0.49366100
H	1.41630000	-0.00740800	-2.46432000
C	4.54893400	3.26641700	-1.95184500
C	4.11973100	4.25970300	-2.84925700
C	4.84256500	5.44523900	-2.96914400
C	5.98865100	5.64811300	-2.19495400
C	6.41708500	4.66342100	-1.30312700
C	5.70294200	3.47119700	-1.18199200
H	3.22358300	4.11169100	-3.44444400
H	4.50828900	6.21189400	-3.66199100
H	6.54583900	6.57616500	-2.28687000
H	7.30614200	4.82185700	-0.69990200
C	4.12682700	0.62510300	-3.21492400
C	4.65688200	1.18272700	-4.38969200
C	4.97658100	0.35270300	-5.46249800
C	4.77984600	-1.02774300	-5.36705800
C	4.25639600	-1.58095300	-4.19834300
C	3.92013400	-0.76017500	-3.12424400
H	4.83501900	2.25023000	-4.46464400
H	5.39225700	0.78409600	-6.36859800
H	5.04617500	-1.67124800	-6.20094900
H	4.12673200	-2.65302100	-4.09398700
C	1.90988700	1.99902200	-1.79712300
C	1.38929100	3.24268900	-1.40479600
C	0.01166800	3.46387600	-1.43667900

C	-0.84884300	2.44381500	-1.84382700
C	-0.33833300	1.19292800	-2.20051200
C	1.03429800	0.96697200	-2.18047700
H	2.04874800	4.04065900	-1.08108200
H	-0.38627800	4.42708700	-1.13075900
H	-1.92020400	2.61625100	-1.87455700
H	-1.01069800	0.39162200	-2.49060100
C	4.82390100	-0.04909600	3.58771400
C	5.04381600	0.58190500	4.82050500
C	6.33045200	0.72281800	5.34071200
C	7.42952400	0.23668100	4.62943700
C	7.22540500	-0.38793400	3.39813400
C	5.93654600	-0.53266700	2.87861300
H	4.19201400	0.96192900	5.38014300
H	6.47357900	1.21478900	6.29959100
H	8.43408500	0.34625100	5.03003900
H	8.07252500	-0.76809800	2.83222800
C	2.77223400	-1.53740900	3.54882800
C	1.50202600	-1.56163500	4.13673500
C	0.90150800	-2.77099600	4.49386500
C	1.56701600	-3.97754800	4.27363800
C	2.83817000	-3.96412900	3.69453400
C	3.43387200	-2.75483900	3.33445400
H	0.97290800	-0.62652000	4.29825900
H	-0.08817200	-2.76696000	4.94379000
H	1.10155800	-4.91961800	4.55171700
H	3.37145400	-4.89571100	3.52421800
C	-4.65513400	-2.30322500	-0.37938800
C	-5.68667700	-2.13842300	-1.31605100
C	-6.45797500	-3.22540700	-1.72487300
C	-6.21817600	-4.50278200	-1.21975900
C	-5.19323100	-4.67286600	-0.29071700
C	-4.42144800	-3.58966600	0.12903300
H	-5.88345900	-1.15877000	-1.73817000
C	-7.51642400	-3.02787000	-2.77654500
H	-6.82521000	-5.34363300	-1.53359300
C	-4.87807000	-6.04744100	0.23633800
C	-4.22730200	2.55667200	0.80169000
C	-3.46708000	3.69793000	0.50297900
C	-4.01838100	4.97280000	0.60056600
C	-5.34005900	5.15004900	1.00815100
C	-6.09752200	4.02171000	1.31943500

[ToC]

C	-5.55365200	2.74067900	1.21827300
H	-2.43817300	3.59286600	0.18375500
C	-3.20124200	6.16155700	0.17368400
H	-5.76929600	6.14226600	1.08362400
C	-7.49980000	4.18832500	1.84095300
F	-8.51429500	-3.93200400	-2.66446300
F	-8.07209900	-1.79701300	-2.70730900
F	-7.00710900	-3.15684500	-4.02539200
F	-4.46617200	-6.01054100	1.52322100
F	-5.94590900	-6.87271400	0.17341500
F	-3.88423800	-6.63346400	-0.47463700
F	-3.37614500	6.43741800	-1.14211000
F	-3.53167600	7.27787500	0.85640600
F	-1.87276000	5.95449600	0.34993300
F	-8.29540600	3.15463500	1.48365600
F	-7.52432400	4.25156700	3.19303900
F	-8.07667900	5.32200400	1.38346000

3a

H	-1.49030800	2.42449300	0.88696000
C	-1.43272800	2.08421800	-0.15481500
H	-0.57298200	-1.25014400	0.38993900
H	-1.23767300	-0.10878300	1.59733700
H	-0.54305500	0.50162400	-1.32625800
C	4.09249900	-0.69994700	0.12568600
C	-0.57226100	0.82094200	-0.28010500
C	0.86543200	1.10167400	0.18331300
C	1.84561100	-0.01883500	-0.10591100
O	3.07100000	0.29080800	0.35598300
O	1.58698500	-1.05745200	-0.68103500
C	-1.15662000	-0.33785100	0.53343400
N	-2.55092500	-0.69823700	0.08803400
O	-3.37364100	-0.96240400	0.96099900
O	-2.77117800	-0.73455700	-1.12056200
H	-1.01132900	2.90021500	-0.75234700
H	-2.45192000	1.90600800	-0.51251200
H	5.00385200	-0.28634100	0.55941000
H	3.83102800	-1.64316600	0.61402900
H	4.22473000	-0.87558800	-0.94571000
H	0.90203600	1.32570800	1.25762500
H	1.24515700	2.00127100	-0.31740400

[ToC]

TS-mode A-RA1-R

H	-3.57178700	-0.63094100	0.80720500
C	-4.00720800	-5.73715700	-1.21294500
H	-5.15596500	-4.52800800	1.35436200
H	-6.07829000	-4.20012700	-0.23706600
H	-3.35375700	-3.63432500	-1.09739100
C	0.55649500	-3.45045500	2.00322900
C	-3.43831100	-4.53565900	-0.49081700
C	-2.47529400	-4.71417900	0.49633100
C	-1.47659300	-3.75662900	0.81685800
O	-0.65878800	-4.18164000	1.83018000
O	-1.24539000	-2.66227700	0.26089800
C	-5.28327800	-3.94721100	0.45225600
N	-5.09475300	-2.60869900	0.60411700
O	-5.55580100	-1.79322000	-0.26041900
O	-4.34517300	-2.18815300	1.54266700
N	-3.23844800	0.33727900	0.64308200
H	-2.44707100	-5.64300100	1.05895400
C	-3.66337000	0.85322000	-0.67975400
C	-3.30038000	-0.21939400	-1.71856700
C	-3.67793000	0.10820100	-3.18640200
H	-6.14199000	-0.77542100	-2.18668800
N	-1.90834300	-0.65952400	-1.57928400
C	-0.78742900	0.09920700	-1.59249800
S	-0.67492700	1.61815000	-2.35479100
N	0.24775600	-0.52394400	-0.93505900
C	1.60195800	-0.18887500	-0.76364900
C	2.10219400	1.11896800	-0.73092900
C	3.46613000	1.34921600	-0.50633000
C	4.33256400	0.27094100	-0.28843000
C	3.84072800	-1.04075600	-0.28707200
C	2.48278200	-1.25945200	-0.54247700
H	-4.79020700	-2.42207100	-2.89859400
P	-3.42196900	1.18870400	2.04760100
H	2.10114100	-2.27409100	-0.60473600
H	6.40739200	-1.47025600	-1.14590400
H	5.37660800	0.45393400	-0.05385600
H	2.29237700	3.52073300	0.64042000
H	1.43096600	1.94573100	-0.90830500
H	-1.82970400	-1.53401500	-1.05659500
H	-0.00842600	-1.41431400	-0.50350100
H	-3.88030700	-1.09447700	-1.42152800

H	-4.74310500	1.02175500	-0.70985800
H	-3.15990300	1.79595400	-0.89395400
H	-2.96733100	0.86019600	-3.53200600
H	-4.88807300	-5.48646200	-1.81094800
H	-4.27658000	-6.53720200	-0.51388300
H	-3.24407000	-6.13791700	-1.89415600
H	1.12583500	-3.99057600	2.76259900
H	0.37783300	-2.42061100	2.32864900
H	1.12296000	-3.42861100	1.06767100
H	-1.73152900	-1.18493000	2.21641000
H	-5.91511500	0.01479600	1.35802000
H	-2.61903400	2.82715100	4.29733500
C	-5.10489500	1.85223500	2.18929400
C	-5.39713900	3.13959100	2.66090800
C	-6.72357600	3.56857800	2.71885400
C	-7.75196200	2.72339700	2.29710500
C	-7.45925200	1.44537900	1.81310600
C	-6.13982900	1.00210000	1.75637100
H	-4.59977900	3.80817000	2.96900200
H	-6.95073100	4.56628800	3.08328700
H	-8.78261500	3.06509300	2.33563200
H	-8.25709700	0.79358700	1.46954600
C	-3.12285800	0.00421500	3.37925500
C	-3.78656700	0.14641100	4.60589800
C	-3.53360800	-0.75953700	5.63544300
C	-2.64231600	-1.81508100	5.43266000
C	-1.99334300	-1.96298900	4.20461000
C	-2.21613000	-1.04817500	3.17776300
H	-4.51061100	0.94277100	4.75168700
H	-4.04817400	-0.65191700	6.58592800
H	-2.46391800	-2.53284900	6.22863000
H	-1.33516200	-2.80801700	4.03069000
C	-2.21668600	2.53312500	2.18588800
C	-1.44499900	2.94079100	1.09029000
C	-0.54365600	3.99625700	1.23523200
C	-0.39556200	4.63542800	2.46705700
C	-1.14708500	4.21268600	3.56726300
C	-2.05440700	3.16421700	3.43219700
H	-1.51691400	2.44197400	0.13136300
H	0.04119000	4.31861900	0.38006800
H	0.31424300	5.44876400	2.57084000
H	-1.02072200	4.69382700	4.53280800

[ToC]

C	-5.07304300	0.70742200	-3.34437500
C	-5.22070300	1.88796900	-4.08487900
C	-6.47547800	2.47136800	-4.27199400
C	-7.61002600	1.88292300	-3.71219300
C	-7.47565600	0.70906600	-2.96702100
C	-6.22186700	0.12398100	-2.78634000
H	-4.34029900	2.35522200	-4.51999700
H	-6.56315100	3.38686100	-4.85179000
H	-8.58850400	2.33547200	-3.85169400
H	-8.35082600	0.24437200	-2.51934100
C	-3.47414800	-1.14146600	-4.04380500
C	-2.60720800	-1.09773300	-5.14122400
C	-2.38705200	-2.22995100	-5.92911500
C	-3.03140200	-3.43013600	-5.62733600
C	-3.89794100	-3.48717600	-4.53285200
C	-4.11795700	-2.35398700	-3.74949200
H	-2.08965400	-0.17018200	-5.37267700
H	-1.70610300	-2.17324600	-6.77468600
H	-2.85865700	-4.31389800	-6.23607800
H	-4.40361700	-4.41740300	-4.28549300
C	3.97660800	2.74300000	-0.45974600
C	5.21425200	3.07792400	-1.03086200
C	5.68983400	4.38666100	-0.98181700
C	4.94660300	5.39580500	-0.36765800
C	3.71952900	5.06683800	0.20200000
C	3.23779100	3.75723500	0.16289300
H	5.80173500	2.31432500	-1.52937200
C	6.98843500	4.73066500	-1.66089300
H	5.32116600	6.41135900	-0.32859600
C	2.88981500	6.10782200	0.89974000
C	4.73604300	-2.18393500	0.01906600
C	4.29199500	-3.22352100	0.84737500
C	5.12201900	-4.29977000	1.15420500
C	6.41940100	-4.36351900	0.64857900
C	6.86973100	-3.32846900	-0.17134700
C	6.04253700	-2.25006400	-0.48572200
H	3.29943700	-3.17854500	1.27842400
C	4.57960300	-5.42220900	1.99750000
H	7.06598500	-5.19958000	0.88753600
C	8.28640900	-3.34546500	-0.68044800
F	5.55801000	-6.09359700	2.64175800
F	3.71145900	-4.96966500	2.93566100

F	3.90437400	-6.32536500	1.24919000
F	8.39603000	-2.74079400	-1.88427300
F	9.12358200	-2.69416100	0.16248500
F	8.76207000	-4.60345900	-0.81152600
F	7.60554800	5.77641500	-1.06616800
F	7.85197000	3.69030500	-1.65186800
F	6.79861700	5.07204300	-2.95709000
F	1.64865400	6.20477000	0.35537400
F	2.70145700	5.79595600	2.20814400
F	3.44179900	7.33558100	0.85567400

TS-mode A-RA2-R

H	-3.65897900	-0.45621100	-1.77556100
C	0.51706000	0.62305800	-3.35533700
H	-1.22332400	-2.27615200	-4.52363100
H	-0.98434600	-0.57289200	-5.25379200
H	-0.57423100	-0.87822900	-2.18270600
C	2.04338000	-5.00918400	-1.54339500
C	0.16726000	-0.79562600	-2.97394500
C	1.14503900	-1.79427300	-2.98941400
C	1.01182700	-2.97161700	-2.20962700
O	2.06122800	-3.83783600	-2.36622300
O	0.09904500	-3.24258200	-1.39379300
C	-1.29024100	-1.20174100	-4.42745600
N	-2.41496200	-0.77764600	-3.77695900
O	-2.73720800	0.44927800	-3.76808500
O	-3.04037100	-1.60538600	-3.02997200
N	-4.15315800	-0.33330500	-0.87834900
H	2.01850400	-1.70792600	-3.62802400
C	-4.31488300	-1.61255500	-0.18360400
C	-3.20412100	-1.86194700	0.85485800
C	-3.22364800	-3.34205900	1.35017500
H	-4.94888800	-1.86749500	2.96905500
N	-1.90658000	-1.54875200	0.28863100
C	-0.97620300	-0.72314900	0.84015700
S	-1.31060100	0.45044800	2.01628500
N	0.26383000	-0.98877400	0.33164000
C	1.48568400	-0.30901600	0.39266600
C	1.62682300	1.07553900	0.54133900
C	2.90425100	1.65413400	0.49625400
C	4.03330700	0.84938400	0.28924500
C	3.89938000	-0.53599200	0.12446700

[ToC]

C	2.62488900	-1.10139900	0.19382700
H	-2.96263700	-2.94622400	4.08341100
P	-4.96805500	1.05750300	-0.60373300
H	2.50310100	-2.17595400	0.10521700
H	6.42153100	-0.32898600	1.15409200
H	5.01011700	1.31153600	0.18716600
H	1.32394200	3.59435400	-0.57900000
H	0.74995100	1.68738000	0.70468700
H	-1.57798300	-2.17137200	-0.44920300
H	0.31663200	-1.87515800	-0.17973800
H	-3.33906200	-1.18328200	1.69751200
H	-5.28962400	-1.67752400	0.30250800
H	-4.28085700	-2.38945000	-0.95406700
H	-3.08546400	-3.95961000	0.45203500
H	-0.37576400	1.20672800	-3.59749000
H	1.20494400	0.65161100	-4.20798000
H	1.02082800	1.10295000	-2.50444200
H	2.93849400	-5.56947300	-1.81266000
H	1.14672600	-5.60836500	-1.72942800
H	2.07503000	-4.74863600	-0.47922600
H	-2.41049600	1.65884200	-1.87741500
H	-4.05447300	1.17046900	2.14116100
H	-6.94327400	3.18006000	-1.06380500
C	-5.85426100	0.88800000	0.96310400
C	-7.22688000	0.60094400	0.99050500
C	-7.86764300	0.40898300	2.21471600
C	-7.14463700	0.50294300	3.40583200
C	-5.77499600	0.78186100	3.37846600
C	-5.12301300	0.97306600	2.16129400
H	-7.79105200	0.52925600	0.06608700
H	-8.93036000	0.18664400	2.23614600
H	-7.64854700	0.35783000	4.35743900
H	-5.20977400	0.84733500	4.30350800
C	-3.85469900	2.47988300	-0.48673000
C	-4.20478700	3.58059900	0.31017200
C	-3.36524000	4.69182800	0.36002600
C	-2.18660200	4.71261500	-0.38750100
C	-1.84610800	3.62050000	-1.18927300
C	-2.67351200	2.49976400	-1.24332600
H	-5.11307300	3.56576300	0.90504500
H	-3.62880300	5.53827900	0.98772700
H	-1.53338500	5.57604300	-0.34191400

H	-0.93458700	3.64488300	-1.77892700
C	-6.13469300	1.37865300	-1.95546600
C	-6.15549900	0.52042100	-3.06376600
C	-7.03260800	0.78299200	-4.11744100
C	-7.88410400	1.88712600	-4.06568400
C	-7.85637800	2.74750900	-2.96329400
C	-6.97727700	2.50143100	-1.91194400
H	-5.47879300	-0.32651700	-3.11678200
H	-7.04422700	0.12367500	-4.98038100
H	-8.56616100	2.08492900	-4.88796500
H	-8.51314100	3.61194500	-2.92789100
C	-4.55986900	-3.74226100	1.96390300
C	-5.06633400	-5.02828600	1.73024500
C	-6.27560300	-5.43920800	2.29135000
C	-7.00791000	-4.56466400	3.09738200
C	-6.51722600	-3.28034800	3.33622200
C	-5.30408300	-2.87486300	2.77712900
H	-4.50251200	-5.71465500	1.10263200
H	-6.64771200	-6.44139800	2.09491400
H	-7.95275900	-4.88085300	3.53140000
H	-7.07889900	-2.58473200	3.95410400
C	-2.02770100	-3.62051700	2.26144900
C	-0.84111400	-4.11835100	1.70385100
C	0.29494900	-4.30427100	2.49238700
C	0.26098100	-4.00390700	3.85506300
C	-0.91853800	-3.51664200	4.42064400
C	-2.05251700	-3.32657900	3.62976100
H	-0.78967000	-4.33201600	0.63974400
H	1.20720900	-4.68471800	2.03891700
H	1.14519700	-4.14656200	4.47067500
H	-0.95681100	-3.27810400	5.48039900
C	3.06236600	3.12649200	0.60922800
C	4.13825800	3.68165500	1.31596100
C	4.30859000	5.06461100	1.38608300
C	3.41518200	5.92547300	0.75337600
C	2.33829900	5.37817600	0.05486400
C	2.15695000	3.99839000	-0.01526700
H	4.83717200	3.03142600	1.83118100
C	5.50981100	5.62409800	2.09969300
H	3.55582000	6.99914200	0.79997200
C	1.35492300	6.30697000	-0.60081700
C	5.07436900	-1.40002700	-0.14825900

C	4.96376000	-2.49378700	-1.02158300
C	6.06075700	-3.31980400	-1.26337200
C	7.29278400	-3.07177700	-0.65945600
C	7.40915400	-1.97873000	0.19847400
C	6.31497300	-1.15305800	0.45670400
H	4.02607300	-2.69448300	-1.53027000
C	5.88606600	-4.52754500	-2.14401500
H	8.14287600	-3.71528500	-0.85204500
C	8.74196700	-1.65215200	0.81639700
F	8.60685000	-1.10884900	2.04752900
F	9.52648800	-2.74596200	0.93870900
F	9.43061000	-0.75604800	0.06881500
F	5.01854200	-4.30045800	-3.15197800
F	7.05294900	-4.93554500	-2.68961500
F	5.39415200	-5.58321400	-1.44056300
F	5.29976200	6.88680500	2.53300200
F	5.85043000	4.87611100	3.17298200
F	6.59507900	5.66114100	1.28938300
F	0.44013900	6.77797400	0.28682800
F	1.95990600	7.38826600	-1.13741700
F	0.65729600	5.70004900	-1.59110800

TS-mode A-RA1-S

H	-3.29710200	0.59037500	-0.03218300
C	-4.20469100	4.56519300	2.24704500
H	-4.90808600	1.09748600	2.95093700
H	-5.97056400	2.61375700	3.04850100
H	-3.29368900	2.77025300	1.39034600
C	-0.13309900	0.54578900	4.43747200
C	-3.60854800	3.17708900	2.34866600
C	-2.72163700	2.88050700	3.41864000
C	-1.81664900	1.81486700	3.32892700
O	-1.06702700	1.62393900	4.45782400
O	-1.64627300	1.06459400	2.32084500
C	-5.26562000	2.00883500	2.48939600
N	-5.65864400	1.80792700	1.16614700
O	-6.34586600	2.67016000	0.57239500
O	-5.17175700	0.81428500	0.53713100
N	-2.77670800	0.07030100	-0.74908300
H	-2.79100600	3.41604100	4.36015900
C	-3.28183600	-1.32215400	-0.80599600
C	-3.36492600	-1.84767500	0.64107600

[ToC]

C	-3.88847100	-3.30142500	0.74810300
H	-6.04565000	-1.50331600	0.45573400
N	-2.10693500	-1.60379000	1.36284800
C	-0.85646000	-1.88225600	0.89614500
S	-0.48797100	-3.22800500	-0.06809200
N	0.04011100	-0.92226400	1.29522400
C	1.38081600	-0.69185700	0.95513200
C	2.30943300	-1.68022500	0.60671200
C	3.62455800	-1.32342200	0.27319600
C	4.01599700	0.02066300	0.31407800
C	3.10050200	1.01715900	0.67998600
C	1.78928100	0.65282000	0.99315900
H	-5.66915200	-2.63933000	2.74861200
P	-2.69562100	0.99377000	-2.13006900
H	1.07103400	1.41316700	1.28648500
H	1.71300900	3.21018200	-0.18748900
H	5.03523000	0.29445900	0.06115200
H	5.51227200	-1.18234600	-1.68320300
H	1.99375600	-2.71201700	0.56317700
H	-2.15511900	-0.73758800	1.90269100
H	-0.40628900	-0.10765600	1.73750800
H	-4.07560500	-1.20551600	1.16719700
H	-4.27368500	-1.36807600	-1.26450000
H	-2.59241800	-1.92810300	-1.39669900
H	-3.17729000	-3.93960100	0.21866800
H	-5.04514000	4.59062000	1.54770000
H	-4.54586100	4.92435000	3.22505800
H	-3.43930700	5.26293100	1.88258100
H	0.28137100	0.49532500	5.44697000
H	-0.61597300	-0.40676400	4.19306600
H	0.68083200	0.72793800	3.72534100
H	-1.21673600	1.94203300	0.17491900
H	-5.26418500	2.22519500	-1.61503600
H	-1.90228200	1.45013900	-4.93271100
C	-4.23607300	0.98000600	-3.07765800
C	-4.39425300	0.16832800	-4.21359300
C	-5.64451700	0.06325300	-4.82165700
C	-6.73955800	0.74987500	-4.29299000
C	-6.59014200	1.53553000	-3.14762100
C	-5.34616100	1.65180600	-2.53195800
H	-3.55285400	-0.38473500	-4.61765400
H	-5.76306800	-0.56257200	-5.70129100

H	-7.71363400	0.66032400	-4.76593100
H	-7.44378800	2.04976900	-2.71656800
C	-2.31615000	2.66074800	-1.55077700
C	-2.72711200	3.78818000	-2.27615100
C	-2.32470700	5.05765700	-1.86175600
C	-1.51187100	5.20385000	-0.73583000
C	-1.11045200	4.08055700	-0.00860100
C	-1.50344000	2.80610200	-0.41373200
H	-3.36229000	3.68041300	-3.14992400
H	-2.64661500	5.93171000	-2.42011600
H	-1.18348400	6.18946000	-0.42306900
H	-0.49904300	4.19727400	0.87929800
C	-1.31220200	0.39718600	-3.12916900
C	-0.32178000	-0.38449100	-2.51787100
C	0.80987400	-0.75963600	-3.24099900
C	0.96369500	-0.34477700	-4.56441700
C	-0.01415300	0.45041900	-5.17037100
C	-1.15051300	0.82659200	-4.45757600
H	-0.43075100	-0.70248100	-1.48923800
H	1.56400100	-1.37581900	-2.76115500
H	1.84745700	-0.63575900	-5.12533900
H	0.10885900	0.77959700	-6.19804900
C	-5.25744600	-3.49415800	0.09809400
C	-5.55977500	-4.74530700	-0.45860900
C	-6.80440300	-4.99819800	-1.03444800
C	-7.77620700	-3.99551000	-1.07018300
C	-7.48852900	-2.74432300	-0.52407500
C	-6.24315900	-2.49507100	0.05803600
H	-4.80755100	-5.53054700	-0.43334900
H	-7.01304500	-5.97694400	-1.45912800
H	-8.74558300	-4.18704900	-1.52297300
H	-8.23137900	-1.95118300	-0.55218500
C	-3.88615600	-3.72932600	2.21549600
C	-2.86476100	-4.55473700	2.70017800
C	-2.81981800	-4.91734500	4.04760800
C	-3.79604600	-4.45499100	4.93202500
C	-4.81750500	-3.62837600	4.45805000
C	-4.86192500	-3.27085000	3.11009100
H	-2.09170900	-4.89723800	2.01761300
H	-2.01910200	-5.55978000	4.40564100
H	-3.76285900	-4.73686800	5.98131600
H	-5.58444800	-3.26475200	5.13746200

C	3.51370900	2.44198300	0.73325400
C	4.76199500	2.80016600	1.26447500
C	5.15517100	4.13579300	1.32175000
C	4.31676700	5.14588200	0.85290000
C	3.07732000	4.79510400	0.32178900
C	2.67368800	3.45964400	0.25660900
H	5.41950300	2.03333400	1.65998600
C	6.52331800	4.47966100	1.84731100
H	4.62382800	6.18345600	0.89979100
C	2.14127600	5.86501300	-0.16860600
C	4.59347500	-2.37348000	-0.13518900
C	4.60491200	-3.63081600	0.48804100
C	5.51414100	-4.61089300	0.09324500
C	6.43331000	-4.36589100	-0.92679400
C	6.42542600	-3.11954600	-1.54937300
C	5.51712700	-2.13405800	-1.16252900
H	3.91518800	-3.83990900	1.29854100
C	5.46729000	-5.97059600	0.73817100
H	7.13929600	-5.12997400	-1.22920300
C	7.43762500	-2.80328000	-2.61722600
F	6.58213300	5.74374800	2.32020600
F	6.89868200	3.65149000	2.84766100
F	7.46541400	4.37550100	0.87943500
F	1.14765600	6.11061100	0.72835800
F	2.76750400	7.04031200	-0.38416200
F	1.53897400	5.50583300	-1.32393500
F	4.61049300	-6.79672700	0.09311400
F	6.67711700	-6.57476100	0.72752100
F	5.05680100	-5.90478700	2.02398900
F	6.91621400	-2.01069400	-3.58267000
F	8.50626000	-2.14352500	-2.10928000
F	7.91002300	-3.91707700	-3.21746700

TS-mode B-RA2-S

C	1.93564300	0.89402200	-1.54271800
C	1.59764300	-0.44124800	-1.82479900
C	2.52622500	-1.45703600	-1.57545600
C	3.74914800	-1.14648600	-0.95830600
C	4.05030600	0.18219000	-0.63259700
C	3.15336400	1.21824000	-0.94232500
N	0.29806600	-0.62553600	-2.33109500
C	-0.58897300	-1.66678700	-2.13452200

[ToC]

S	-0.18078300	-3.30268800	-1.99118000
N	-1.86270000	-1.20523900	-2.07617600
C	-3.02133300	-1.96882900	-1.65413200
C	-4.30911600	-1.27684100	-2.19506500
H	-5.03459900	-3.97927200	-2.21001300
C	-3.01673600	-2.11517700	-0.11941200
N	-2.88602500	-0.79996300	0.54392900
H	0.00897500	0.83151800	1.68223800
P	-2.48635200	-0.65903900	2.13761300
H	-5.09057800	-3.05523700	-4.21435600
O	-4.74447100	1.34431400	0.32878300
C	-5.15010400	2.51363800	0.51529000
O	-6.41607600	2.74107200	0.99344500
H	-2.82591000	-0.67175300	5.09038400
H	-4.99986700	4.64718200	0.32169800
H	-2.51158800	2.84489100	0.30834400
H	-1.37398000	4.96152400	-0.55227000
C	-7.24735000	1.60412100	1.21601800
C	-4.43902900	3.71820300	0.29259700
C	-3.07288000	3.71848900	-0.02233200
C	-2.81617700	3.13713300	-1.95598300
N	-1.53224800	2.66018900	-1.94905600
O	-1.33413100	1.48162000	-1.46429500
C	-2.31514600	5.02484300	-0.00365400
H	-2.91927600	5.83184300	-0.43478800
H	-2.07512000	5.30277700	1.02928800
H	-6.70428700	0.79389900	1.71052700
H	-3.57183100	2.36512300	-1.97736200
H	-2.95350900	4.03693600	-2.54236200
O	-0.54974200	3.39918300	-2.21930700
H	1.21768000	1.67207800	-1.78401200
H	5.60423000	2.39703600	-0.68380900
H	4.95960300	0.40110800	-0.08351400
H	6.48845500	-1.09067900	-0.92645500
H	2.28672900	-2.47258500	-1.85549600
H	-0.18404100	0.27653100	-2.36571400
H	-1.95875800	-0.19508000	-1.94136800
C	1.09166700	-2.52923100	1.67537800
C	-0.03289700	-1.73362200	1.48400600
H	-1.83225900	-2.71313000	4.21831900
H	-4.31413600	-0.25763500	-1.78822800
H	0.15687300	-4.13832900	4.52304800

H	2.04067200	-4.02905600	2.89154700
H	1.89707700	-2.47220400	0.95540300
H	-2.15038700	-2.72219400	0.14836600
H	-3.91546900	-2.65049600	0.20211300
H	-3.47307100	-0.02097100	0.21888300
H	-0.09785400	-1.10434700	0.60302400
H	-8.06452500	1.95039200	1.85434200
H	-7.66469000	1.23443600	0.27202000
H	-2.94278000	-2.97443100	-2.07458700
C	-1.99538000	1.04731100	2.49163500
C	-2.90528100	1.93131300	3.09131500
C	-2.51827300	3.24195100	3.36868200
C	-1.23128300	3.67566700	3.04897100
C	-0.32920600	2.80296600	2.43525300
C	-0.70480700	1.49308100	2.15677400
H	-3.91303500	1.60851100	3.32750700
H	-3.23123600	3.92563400	3.81866400
H	-0.93117100	4.69785500	3.25895100
H	0.66228600	3.14723200	2.16385000
C	-3.87185300	-1.07219900	3.23438400
C	-5.09490300	-1.43455800	2.65087800
C	-6.18815500	-1.73819200	3.46311000
C	-6.06828500	-1.67424800	4.85210100
C	-4.85587000	-1.29431800	5.43623500
C	-3.75913600	-0.98825000	4.63274400
H	-5.20818300	-1.45468900	1.57273300
H	-7.13035400	-2.02067700	3.00283000
H	-6.92085400	-1.91153300	5.48223300
H	-4.76700100	-1.22933400	6.51667200
C	-1.08709100	-1.77528700	2.41335600
C	-1.01398100	-2.64722900	3.50992100
C	0.11138000	-3.45517200	3.67996300
C	1.16823300	-3.39328100	2.76883400
C	-5.55971400	-1.95588100	-1.65044200
C	-6.50668600	-1.17858300	-0.97281900
C	-7.64476800	-1.75874400	-0.40790800
C	-7.85188500	-3.13578000	-0.50588200
C	-6.90790300	-3.92516300	-1.16717100
C	-5.77109700	-3.34199300	-1.72899300
H	-6.32487300	-0.11527400	-0.86161500
H	-8.36526800	-1.13260700	0.11298300
H	-8.73549000	-3.59184300	-0.06718800

H	-7.05106100	-5.00007000	-1.23957600
C	-4.25417200	-1.12726500	-3.71671600
C	-3.73068600	0.05418200	-4.26162900
C	-3.61420300	0.22536700	-5.64058500
C	-4.02547700	-0.78808000	-6.50768000
C	-4.55204900	-1.96751300	-5.97970100
C	-4.66490700	-2.13488800	-4.59815200
H	-3.40044100	0.84950400	-3.59948200
H	-3.20315200	1.15124400	-6.03437800
H	-3.93845100	-0.65916500	-7.58322000
H	-4.87980500	-2.76345500	-6.64350500
C	4.68891800	-2.23480100	-0.58224900
C	4.20947400	-3.48903900	-0.17616100
C	5.08259300	-4.48268300	0.26419300
C	6.45904100	-4.26981300	0.28183800
C	6.94637300	-3.03838800	-0.15650800
C	6.07818300	-2.03211200	-0.57769600
H	3.14571700	-3.69663800	-0.18591600
C	4.49599600	-5.75824800	0.80321700
H	7.13614900	-5.04616200	0.61679500
C	8.42567300	-2.76312000	-0.11642400
C	3.45669800	2.63205700	-0.59419600
C	2.42933800	3.56325000	-0.36542500
C	2.73102100	4.88237400	-0.01985000
C	4.04990800	5.31057200	0.10790200
C	5.07311000	4.39310000	-0.13009300
C	4.78432200	3.07681300	-0.48136800
H	1.38754800	3.27289200	-0.46283400
C	1.59323800	5.83994300	0.21292000
H	4.27725800	6.33256000	0.38488900
C	6.50637400	4.84828900	-0.07816400
F	5.40794600	-6.74774800	0.89638400
F	3.98852900	-5.57141500	2.05384500
F	3.47623000	-6.20725300	0.04135300
F	8.78486200	-2.15439000	1.04010400
F	9.15524100	-3.89674900	-0.20638500
F	8.81369300	-1.94738600	-1.12210300
F	6.93450900	5.29147400	-1.28388600
F	6.68120500	5.86380200	0.79746800
F	7.34024300	3.84818900	0.28869700
F	2.01680700	7.07171800	0.56860000
F	0.82481300	5.97846800	-0.88775700

F	0.77044200	5.40405400	1.20639900

[ToC]

References

(1) Pupo, G.; Vicini, A. C.; Ascough, D. M. H.; Ibba, F.; Christensen, K. E.; Thompson, A. L.; Brown, J. M.; Paton, R. S.; Gouverneur, V. Hydrogen Bonding Phase-Transfer Catalysis with Potassium Fluoride: Enantioselective Synthesis of β-Fluoroamines. *J. Am. Chem. Soc.* **2019**, *141*, 2878–2883.

(2) Núñez, M. G.; Farley, A. J. M.; Dixon, D. J. Bifunctional Iminophosphorane Organocatalysts for Enantioselective Synthesis: Application to the Ketimine Nitro-Mannich Reaction. *J. Am. Chem. Soc.* **2013**, *135*, 16348–16351.

(3) Szigeti, M.; Dobi, Z.; Soós, T. The Goldilocks Principle in Phase Labeling. Minimalist and Orthogonal Phase Tagging for Chromatography-Free Mitsunobu Reaction. *J. Org. Chem.* **2018**, *83*, 2869–2874.

(4) Brackeen, M. F.; Stafford, J. A.; Cowan, D. J.; Brown, P. J.; Domanico, P. L.; Feldman, P. L.; Rose, D.; Strickland, A. B.; Veal, J. M.; Verghese, M. Design and Synthesis of Conformationally Constrained Analogs of 4-(3-Butoxy-4-Methoxybenzyl)Imidazolidin-2-One (Ro 20-1724) as Potent Inhibitors of CAMP-Specific Phosphodiesterase. *J. Med. Chem.* **1995**, *38*, 4848–4854.

(5) (a) Matheau-Raven, D.; Dixon, D. J. A One-Pot Synthesis-Functionalization Strategy for Streamlined Access to 2,5-Disubstituted 1,3,4-Oxadiazoles from Carboxylic Acids. *J. Org. Chem.* **2022**, *87*, 12498–12505. (b) Nodes, W. J.; Nutt, D. R.; Chippindale, A. M.; Cobb, A. J. A. Enantioselective Intramolecular Michael Addition of Nitronates onto Conjugated Esters: Access to Cyclic γ-Amino Acids with up to Three Stereocenters. *J. Am. Chem. Soc.* **2009**, *131*, 16016–16017.

(6) Clark, P. G. K.; Vieira, L. C. C.; Tallant, C.; Fedorov, O.; Singleton, D. C.; Rogers, C. M.; Monteiro, O. P.; Bennett, J. M.; Baronio, R.; Müller, S.; Daniels, D. L.; Méndez, J.; Knapp, S.; Brennan, P. E.; Dixon, D. J. LP99: Discovery and Synthesis of the First Selective BRD7/9 Bromodomain Inhibitor. *Angew. Chem. Int. Ed.* **2015**, *54*, 6217–6221.

(7) Feng, X.; Sun, A.; Zhang, S.; Yu, X.; Bao, M. Palladium-Catalyzed Carboxylative Coupling of Benzyl Chlorides with Allyltributylstannane: Remarkable Effect of Palladium Nanoparticles. *Org. Lett.* **2013**, *15*, 108–111.

(8) Li, L.; Stimac, J. C.; Geary, L. M. Synthesis of Olefins via a Wittig Reaction Mediated by Triphenylarsine. *Tetrahedron Lett.* **2017**, *58*, 1379–1381.

(9) Qian, B.; Chen, S.; Wang, T.; Zhang, X.; Bao, H. Iron-Catalyzed Carboamination of Olefins: Synthesis of Amines and Disubstituted β-Amino Acids. *J. Am. Chem. Soc.* **2017**, *139*, 13076–13082.

(10) Youn, S. W.; Kim, B. S.; Jagdale, A. R. Pd-Catalyzed Sequential C–C Bond Formation and Cleavage: Evidence for an Unexpected Generation of Arylpalladium(II) Species. *J. Am. Chem. Soc.* **2012**, *134*, 11308–11311.

(11) Zhang, H.; Huang, X. Ligand-Free Heck Reactions of Aryl Iodides: Significant Acceleration of the Rate through Visible Light Irradiation at Ambient Temperature. *Adv. Synth. Catal.* **2016**, *358*, 3736–3742.

(12) Ojha, S.; Panda, N. Pd-Catalyzed Desulfitative Arylation of Olefins by N -Methoxysulfonamide. *Org. Biomol. Chem.* **2022**, *20*, 1292–1298.

(13) Schmidt, B.; Elizarov, N.; Berger, R.; Petersen, M. From Paracetamol to Rolipram and Derivatives: Application of Deacetylation-Diazotation Sequences and Palladium-Catalyzed Matsuda-Heck Reaction. *Synthesis (Stuttg).* **2013**, *45*, 1174–1180.

(14) Saha, A.; Guin, S.; Ali, W.; Bhattacharya, T.; Sasmal, S.; Goswami, N.; Prakash, G.; Sinha, S. K.; Chandrashekar, H. B.; Panda, S.; Anjana, S. S.; Maiti, D. Photoinduced Regioselective Olefination of Arenes at Proximal and Distal Sites. *J. Am. Chem. Soc.* **2022**, *144*, 1929–1940.

(15) Liu, W.; Wang, D.; Duan, Y.; Zhang, Y.; Bian, F. Palladium Supported on Poly (Ionic Liquid) Entrapped Magnetic Nanoparticles as a Highly Efficient and Reusable Catalyst for the Solvent-Free Heck Reaction. *Tetrahedron Lett.* **2015**, *56*, 1784–1789.

(16) Larionova, N. A.; Ondozabal, J. M.; Cambeiro, X. C. Reduction of Electron-Deficient Alkenes Enabled by a Photoinduced Hydrogen Atom Transfer. *Adv. Synth. Catal.* **2021**, *363*, 558–564.

(17) Felluga, F.; Gombac, V.; Pitacco, G.; Valentin, E. A short and convenient chemoenzymatic synthesis of both enantiomers of 3-phenylGABA and 3-(4-chlorophenyl)GABA (Baclofen). *Tetrahedron: Asymmetry*, **2005**, *16*, 1341–1345.

(18) Jensen, K. L., Poulsen, P. H., Donslund, B. S., Morana, F.; Jørgensen, K. A. Asymmetric Synthesis of γ-Nitroesters by an Organocatalytic One-Pot Strategy. *Org. Lett.*, **2012** *14*, 1516–1519.

(19) Rodríguez, V., Sánchez, M., Quintero, L.; Sartillo-Piscil, F. The 5-exo-trig radical cyclization reaction under reductive and oxidative conditions in the synthesis of optically pure GABA derivatives. *Tetrahedron*, **2004**, *60*, 10809–10815.

(20) Meyers, A. I.; Snyder, L. The Synthesis of Aracemic 4-Substituted Pyrrolidinones and 3-Substituted Pyrrolidines. An Asymmetric Synthesis of (-)-Rolipram. *J. Org. Chem.* **1993**, *58*, 36–42.

(21) Nagy, B. S., Llanes, P., Pericas, M. A., Kappe, C. O.; Ötvös, S. B. Enantioselective Flow Synthesis of Rolipram Enabled by a Telescoped Asymmetric Conjugate Addition–Oxidative Aldehyde Esterification Sequence Using in Situ -Generated Persulfuric Acid as Oxidant. *Org. Lett.*, **2022**, *24*, 1066–1071.

(22) Montoya-Balbás, I., Valentín-Guevara, B., López-Mendoza, E., Linzaga-Elizalde, I., Ordoñez, M.; Román-Bravo, P. Efficient Synthesis of β-Aryl-γ-lactams and Their Resolution with (*S*)-Naproxen: Preparation of (*R*)- and (*S*)-Baclofen. *Molecules*, **2015**, *20*, 22028–22043.

(23) Biswas, K., Gholap, R., Srinivas, P., Kanyal, S.; Sarma, K. Das. β-Substituted γ-butyrolactams from mucochloric acid: synthesis of (±)-baclofen and other γ-aminobutyric acids and useful building blocks. *RSC Advances*, **2014**, *4*, 2538–2545.

(24) Gaussian 16, Revision C.02, M. J. Frisch, G. W. Trucks, H. B. Schlegel, G. E. Scuseria, M. A. Robb, J. R. Cheeseman, G. Scalmani, V. Barone, G. A. Petersson, H. Nakatsuji, X. Li, M. Caricato, A. V. Marenich, J. Bloino, B. G. Janesko, R. Gomperts, B. Mennucci, H. P. Hratchian, J. V. Ortiz, A. F. Izmaylov, J. L. Sonnenberg, D. Williams-Young, F. Ding, F. Lipparini, F. Egidi, J. Goings, B. Peng, A. Petrone, T. Henderson, D. Ranasinghe, V. G. Zakrzewski, J. Gao, N. Rega, G. Zheng, W. Liang, M. Hada, M. Ehara, K. Toyota, R. Fukuda, J. Hasegawa, M. Ishida, T. Nakajima, Y. Honda, O. Kitao, H. Nakai, T. Vreven, K. Throssell, J. A. Montgomery, Jr., J. E. Peralta, F. Ogliaro, M. J. Bearpark, J. J. Heyd, E. N. Brothers, K. N. Kudin, V. N. Staroverov, T. A. Keith, R. Kobayashi, J. Normand, K. Raghavachari, A. P. Rendell, J. C. Burant, S. S. Iyengar, J. Tomasi, M. Cossi, J. M. Millam, M. Klene, C. Adamo, R. Cammi, J. W. Ochterski, R. L. Martin, K. Morokuma, O. Farkas, J. B. Foresman, and D. J. Fox, Gaussian, Inc., Wallingford CT, 2016.

(25) (a) Becke, A. D. *J. Chem. Phys.* **1993**, *98*, 5648−5652. (b) Lee, C.; Yang, W.; Parr, R. G. *Phys. Rev. B* **1988**, *37*, 785−789.

(26) Grimme, S.; Antony, J.; Ehrlich, S.; Krieg, H. *J. Chem. Phys.* **2010**, *132*, 154104.

(27) Marenich, A. V.; Cramer, C. J.; Truhlar, D. G *J. Phys. Chem. B* **2009**, *113*, 6378–6396.

(28) CYLview20; Legault, C. Y., Unversité de Sherbrooke: Sherbrooke, **2020** (http://www.cylview.org).

(29) Zhao, Y.; Truhlar, D.G. *Theor. Chem. Acc.*, **2008**, *120*, 215-241.

(30) (a) Okino, T.; Hoashi, Y.; Takemoto, Y. *J. Am. Chem. Soc.* **2003**, *125*, 12672–12673. (b) Okino, T.; Hoashi, Y.; Furukawa, T.; Xu, X.; Takemoto, Y. *J. Am. Chem. Soc.* **2005**, *127*, 119–125. (c) Hamza, A.; Schubert, G.; Soós, T.; Pápai, I. *J. Am. Chem. Soc.* **2006**, *128*, 13151–13160. (d) Kótai, B.; Kardos, G.; Hamza, A.; Farkas, V.; Pápai, I.; Soós, T. *Chem. Eur. J.* **2014**, *20*, 5631–5639. (e) Su, G.; Thompson, C. J.; Yamazaki, K.; Rozsar, D.; Christensen, K.; Hamlin, T. A.; Dixon, D. J. *Chem. Sci.* **2021**, *12*, 6064–6072. (f) Rozsar, D.; Formica, M.; Yamazaki, K.; Hamlin, T. A.; Dixon, D. J. *J. Am. Chem. Soc.* **2022**, *144*, 1006–1015.
